# Molecular evolution of *Phox*-related regulatory subunits for NADPH oxidase enzymes

**DOI:** 10.1186/1471-2148-7-178

**Published:** 2007-09-27

**Authors:** Tsukasa Kawahara, J David Lambeth

**Affiliations:** 1 Department of Pathology and Laboratory Medicine, Emory University School of Medicine, Atlanta, Georgia, 30322, USA

## Abstract

**Background:**

The reactive oxygen-generating NADPH oxidases (Noxes) function in a variety of biological roles, and can be broadly classified into those that are regulated by subunit interactions and those that are regulated by calcium. The prototypical subunit-regulated Nox, Nox2, is the membrane-associated catalytic subunit of the phagocyte NADPH-oxidase. Nox2 forms a heterodimer with the integral membrane protein, p22*phox*, and this heterodimer binds to the regulatory subunits p47*phox*, p67*phox*, p40*phox *and the small GTPase Rac, triggering superoxide generation. Nox-organizer protein 1 (NOXO1) and Nox-activator 1 (NOXA1), respective homologs of p47*phox *and p67*phox*, together with p22*phox *and Rac, activate Nox1, a non-phagocytic homolog of Nox2. NOXO1 and p22*phox *also regulate Nox3, whereas Nox4 requires only p22*phox*. In this study, we have assembled and analyzed amino acid sequences of Nox regulatory subunit orthologs from vertebrates, a urochordate, an echinoderm, a mollusc, a cnidarian, a choanoflagellate, fungi and a slime mold amoeba to investigate the evolutionary history of these subunits.

**Results:**

Ancestral p47*phox*, p67*phox*, and p22*phox *genes are broadly seen in the metazoa, except for the ecdysozoans. The choanoflagellate *Monosiga brevicollis*, the unicellular organism that is the closest relatives of multicellular animals, encodes early prototypes of p22*phox*, p47*phox *as well as the earliest known Nox2-like ancestor of the Nox1-3 subfamily. p67*phox- *and p47*phox*-like genes are seen in the sea urchin *Strongylocentrotus purpuratus *and the limpet *Lottia gigantea *that also possess Nox2-like co-orthologs of vertebrate Nox1-3. Duplication of primordial p47*phox *and p67*phox *genes occurred in vertebrates, with the duplicated branches evolving into NOXO1 and NOXA1. Analysis of characteristic domains of regulatory subunits suggests a novel view of the evolution of Nox: in fish, p40*phox *participated in regulating both Nox1 and Nox2, but after the appearance of mammals, Nox1 (but not Nox2) became independent of p40*phox*. In the fish *Oryzias latipes*, a NOXO1 ortholog retains an autoinhibitory region that is characteristic of mammalian p47*phox*, and this was subsequently lost from NOXO1 in later vertebrates. Detailed amino acid sequence comparisons identified both putative key residues conserved in characteristic domains and previously unidentified conserved regions. Also, candidate organizer/activator proteins in fungi and amoeba are identified and hypothetical activation models are suggested.

**Conclusion:**

This is the first report to provide the comprehensive view of the molecular evolution of regulatory subunits for Nox enzymes. This approach provides clues for understanding the evolution of biochemical and physiological functions for regulatory-subunit-dependent Nox enzymes.

## Background

Nox enzymes (reactive oxygen-generating NADPH-oxidases) diverged early in evolution into calcium-regulated Noxes (e.g., Nox5 and the Duox enzymes) and Noxes that are activated by binding to regulatory subunits [1,2]. The most extensively studied of the latter is the phagocyte NAPDH-oxidase (*Phox*), whose role in host defense has been documented at length [3-8]. Professional phagocytes such as neutrophils and macrophages produce large amounts of superoxide, with secondary production of other microbicidal reactive oxygen species (ROS). Superoxide is generated by the *Phox*, which consists of the catalytic subunit gp91*phox *(a.k.a. Nox2), along with the regulatory subunits p22*phox*, p67*phox*, p47*phox*, p40*phox*, and the small GTPase, Rac [3,6,9-11]. The importance of the oxidase is demonstrated by the inherited condition, chronic granulomatous disease (CGD), in which absent or mutated *Phox *proteins result in an inability of phagocytes to kill microbes [4,12,13].

Nox2 and p22*phox *are integral membrane proteins that form a heterodimer referred to as flavocytochrome *b*_558_. In resting cells, flavocytochrome *b*558 is inactive and p47*phox*, p67*phox*, and p40*phox *are all present in the cytoplasm. Cell activation is accompanied by phosphorylation of p47*phox *and probably other *Phox*-regulatory subunits [3,10,14-16], and by guanine nucleotide exchange factors that convert Rac-GDP to Rac-GTP [3,17]. These events trigger assembly of these proteins at the membrane, resulting in activation of Nox2. An intricate set of protein-protein interactions facilitates the binding of the regulatory subunits to the membrane components and to each other, and these interactions are mediated by well-characterized modular interaction domains. For example, p47*phox *and p67*phox *bind via a C-terminal proline-rich region (PRR) in p47*phox *and a C-terminal Src homology 3 (SH3) domain in p67*phox *[18]. In the non-activated cell, the p47*phox*-p67*phox *complex fails to bind to flavocytochrome *b*_558_, due to an unusual autoinhibitory mechanism. In the resting cell, tandem SH3 domains in p47*phox *(referred to as the *bis*-SH3 domain) bind to an auto-inhibitory region (AIR), preventing the *bis*-SH3 domain from binding to the PRR of p22*phox*. Upon cell activation, serine residues of the AIR become phosphorylated, releasing the *bis-*SH3 domain so that it can now bind to p22*phox *[19-22]. In addition, p47*phox *possesses another membrane-binding region, the PX domain, which binds to the headgroups of phosphatidylinositols present in the membrane [23-25]. Together, these interactions with membrane lipids and p22*phox *promote the assembly of p47*phox *and p67*phox *with flavocytochrome *b*_558_. Concurrently, Rac-GTP translocates to the membrane where it interacts with the N-terminal tetratricopeptide repeat (TPR) region of p67*phox *[26-28]. An activation domain (AD) in p67*phox*, which is conformationally regulated by Rac binding [29], activates the hydride transfer from NADPH to FAD in Nox2. Subsequent electron transfer through two heme groups permits reduction of oxygen to form superoxide [30,31]. p40*phox *binds through its *Phox*/Bem 1 (PB1) domain to a partner PB1 domain in p67*phox *and facilitates the assembly of p67*phox*-p47*phox *at the membrane through lipid binding via its PX domain [32-34]. Although no CGD patients have been described with mutations in p40*phox*, neutrophils from p40*phox*-knockout mice exhibit CGD-like severe defects in Nox2-derived oxidant-dependent bacterial killing [35], also suggesting that p40*phox *is a crucial component for Nox2 function.

The first homologue of gp91*phox *identified was Nox1, which is abundantly expressed in non-phagocyte cells including gastrointestinal epithelium and vascular smooth muscle [9,36]. Subsequently, additional homologues were identified, and the Nox/Duox family in humans now consists of seven members: Nox1 though Nox5, Duox1 and Duox2 [9,37]. All members of Nox/Duox family contain the six transmembrane α-helical heme-binding domain and an FAD domain containing an NADPH binding site, which together constitute the "Nox domain"[1]. Nox5 and Duox1/2 contain a calcium-binding EF-hand motif. The evolution of these Noxes and their calcium-binding domains were considered in the recent article [1] and are not discussed here. The remaining Noxes, Nox1-4, require p22*phox *for activity, and Nox1-3 all require additional *Phox*-like regulatory subunits, reviewed recently [10,11,37].

Nox1, like gp91*phox*, is activated by regulatory subunits: NOXO1 (Nox-organizer protein 1) is a homologue of p47*phox *and NOXA1 (Nox-activator protein 1) is a homologue of p67*phox *[38-41]. Recent studies have also demonstrated activation of the Nox1 system by Rac1 [42-45]. NOXO1 and NOXA1 are co-expressed with Nox1 in colon epithelium and gastric mucosa [39,43,46], consistent with *in vivo *regulation of Nox1 by these novel subunits. Activation of Nox1 by regulatory subunits differs in two major respects compared to gp91*phox*. First, NOXO1 lacks the AIR and its associated regulatory phosphorylation sites that are present in p47*phox*. Consistent with the domain structure, NOXO1 co-localizes with Nox1/p22*phox *complex constitutively at/near the plasma membrane *in vivo *[40,41]. On the other hand, a recent study revealed that the *bis*-SH3 domain of NOXO1 bind to the extremely C-terminal PRR of NOXO1 [47]. Phosphorylation-independent disruption of the intramolecular interaction facilitates NOXO1 binding to the PRR of p22*phox*. Second, NOXA1 lacks critical basic amino acid residues in its PB1 domain that are important for binding to p40*phox*. NOXA1 does not interact with p40*phox*, making human Nox1 activity independent of p40*phox *[40]. Furthermore, human NOXA1 lacks the central SH3 domain that is present in p67*phox *[38-40], but whose function is unknown. Like Nox1, Nox3 is also regulated by p22*phox *and NOXO1, but in humans does not require NOXA1 [48-51]. In comparison, Nox4 requires p22*phox *for activity, but other known regulatory subunits are not needed [52-54].

Another subfamily of the Noxes is the NoxA/NoxB group, found in a slime mold amoeba and fungi. These fungal Nox genes have been identified in most ascomycetes except for hemiascomycete yeasts, and also in higher basidiomycetes and chytridiomycotes (reviewed in [55]). No Nox gene has been identified in fungi that belong to Zygomycota and Micropordia. These NoxA/NoxB genes are closely related to the subunit-regulated Noxes according to taxonomy [1]. A non-animal homologue of p67*phox*/NOXA1 was described in slime mold amoeba *Dictyostelium discoideum *(*D. discoideum*) [56]. The p67*phox *homolog (referred to here as the Dd-p67-like) of *D. discoideum *possesses the N-terminal TPR region and PB1 domain characteristic of p67*phox*, but lacks the SH3 domain [56]. A p67*phox *homolog Nox-regulator (NOXR) was also reported in some fungi including ascomycetes and higher basidiomycetes [55,57]. The basic motif structure of NOXR is similar to that of *D. discoideum*. A NOXR-deficient mutant of the ascomycete *Epichlöe festucae *shows decreased ROS-generation [57]. Taken together, these data suggest that NOXR regulates NoxA and/or NoxB in the slime mold and fungi, although this has not been tested directly.

Herein, we assembled and analyzed experimentally cloned and computationally predicted amino acid sequences of the *Phox*-related regulatory subunits p47*phox*, NOXO1, p67*phox*, NOXA1, p40*phox*, p22*phox*, and NOXR from 10 vertebrates, one urochordate, one echinoderm, one mollusc, one cnidarian, one choanoflagellate, 13 fungi, and one slime mold amoeba. Using this large set of amino acid sequences, we report: (i) the occurrence and origin of *Phox*-like subunits during evolution, (ii) identification of putative key amino acid residues conserved in regulatory subunits, (iii) the description of putative new modular regions that are conserved among *Phox*-related regulatory subunits, (iv) the molecular evolution of characteristic domains of the *Phox*-related regulatory subunits, and (v) a prediction of novel fungal and amoebal Nox organizer/enhancer subunits based on sequence analysis.

## Results and discussion

### Occurrence of *Phox*-related regulatory subunits

**Figure 1:**
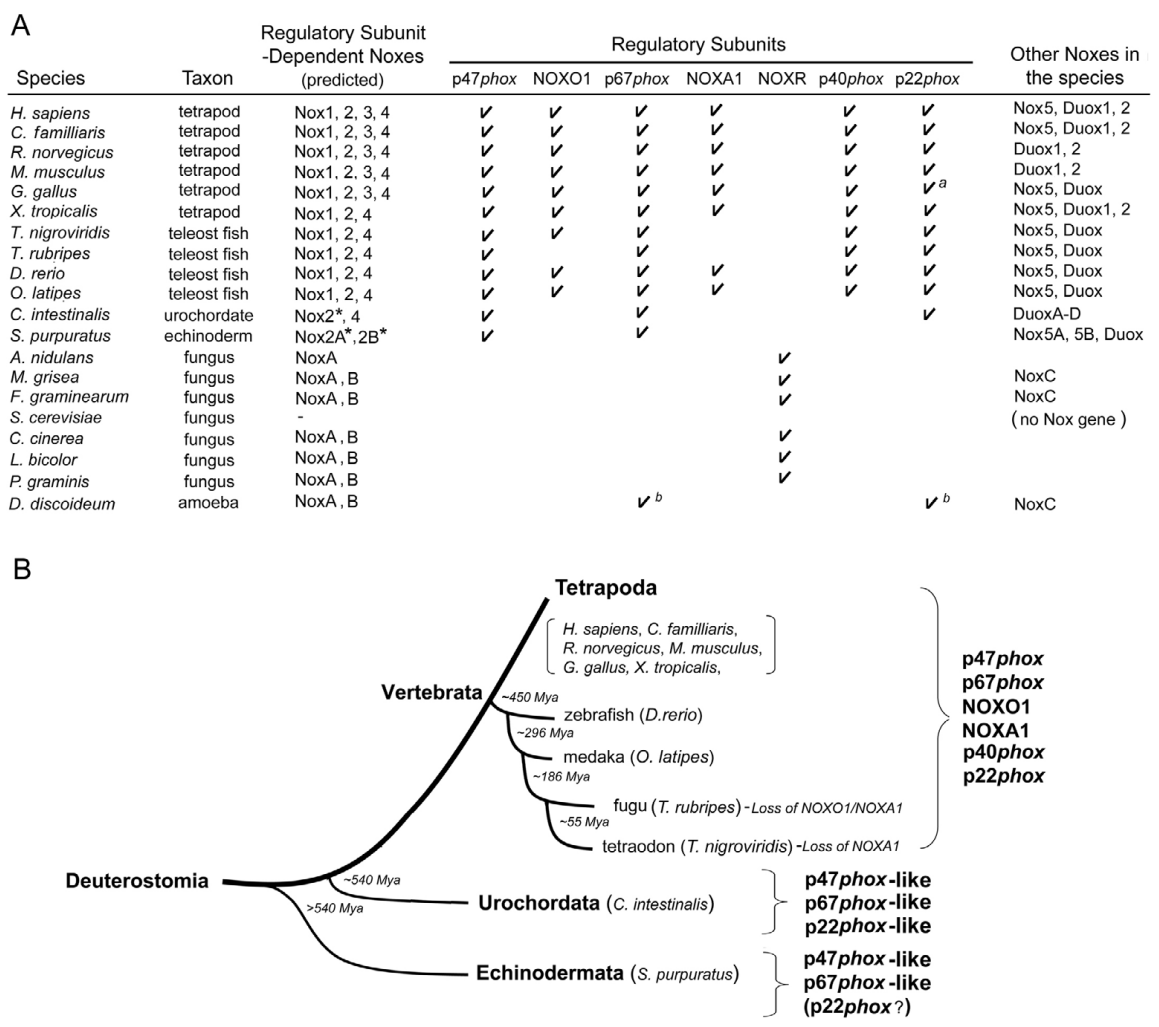
**Relationships between the phylogenic tree of species and the occurrence of *Phox*-reated regulatory subunits**. (*A*) Summary of the occurrence of Nox-regulatory subunit genes in vertebrates, a urochordate, an echinoderm, fungi, and a slime mold amoeba are shown. *Asterisks *indicate that the gene is a co-ortholog ancestor of all members of the vertebrate Nox1-3 subgroup. Superscripted letters represent: (*a*), a partial amino acid sequence predicted from genomic DNA fragment (nucleotides 713622–713565 of GenBank™ No. NW_001471438.1), (*b*) the gene taxonomy is discussed in the text. (*B*) Summary of the occurrence of Nox-regulatory subunit genes within Deuterostomia. The number in the fork of each branch indicates the estimated time in millions of years (Mya, millions of years ago) since a given species diverged. A schematic phylogeny and the estimated time of occurrence of species were created from genomic information [62, 125]. Branch lengths are for illustrative purposes and are not proportional to time since divergence.

Figure 1A summarizes the occurrence of subunit-dependent-like Nox orthologs along with the *Phox*-like regulatory subunit orthologs in the genomes of deutrostomes, fungi, a slime mold amoeba. The tetrapods [human *Homo sapiens *(*H. sapiens*), dog *Canis familliaris *(*C. familliaris*), rat *Rattus norvegicus *(*R. norvegicus*), mouse *Mus musculus *(*M. musculus*), chicken *Gallus gallus *(*G. gallus*), frog *Xenopus tropicalis *(*X. tropicalis*)], and the two teleost fish [zebrafish *Danio rerio *(*D. rerio*), medaka *Oryzias latipes *(*O. latipes*)] have two isologs of Nox organizer proteins (p47*phox *and NOXO1), two isologs of Nox activator proteins (p67*phox *and NOXA1), and single isologs of p40*phox *and p22*phox *(Figure 1A, the sequences are provided in Additional file 1). The pufferfish *Takifugu rubripes *(*T. rubripes*), which has a compact genome size, ~400 megabase (Mb) [58], lacks NOXO1 and NOXA1 although it possesses both Nox1 and Nox2 as well as p22*phox*, p47*phox*, p67*phox*, and p40*phox *(Figure 1A), in contrast to *D. rerio *and *O. latipes *that have less compact genomes (1680–2280 Mb and 870–1100 Mb, respectively, according to the Animal Genome Size Database [59]). Interestingly, *Tetraodon nigroviridis *(*T. nigroviridis*), another species of pufferfish, which also possesses a compact genome, ~340 Mb [60], encodes a NOXO1 ortholog, but not NOXA1 (Figure 1A).

The earlier chordate *Ciona intestinalis *(*C. intestinalis*) possesses a Nox2 ortholog, but not Nox1 or Nox3 [1,61]. The sea urchin *Strongylocentrotus purpuratus *(*S. purpuratus*) belongs to Echinodermata, which diverged early from an ancestor common to urochordates and vertebrates [62]. *S. purpuratus*, possesses two Nox2 genes, Nox2A and Nox2B, and an analysis of the taxonomy Noxes demonstrates that these Nox2 genes are related to the common ancestor gene of vertebrate Nox1, Nox2 and Nox3 [1]. *C. intestinalis *possesses single copies of p47*phox-*, p67*phox-*, and p22*phox*-like genes [61], which presumably regulate its Nox2 ortholog (Figure 1A). The *S. purpuratus *genome possesses single copies of p47*phox*- and p67*phox*-like genes (Figure 1A). No p22*phox *ortholog of *S. purpuratus *was identified using a BLAST search of recently published genome sequences [63]. Because the *S. purpuratus *p47*phox*-like protein has a highly conserved tandem SH3 region that in other species binds to the C-terminal proline-rich region (PRR) of p22*phox*, it seems likely that *S. purpuratus *has an as-yet unidentified p22*phox *homologue.

Using the estimated time of evolution of deuterostomes based on Hox gene clusters to construct a phylogenetic tree [64], we illustrate the occurrence during evolution of the *Phox*-related regulatory subunits(Figure 1B). The phylogenic tree suggests an evolutionary model in which duplication of both the p47*phox *gene and the p67*phox *gene occurred at a time corresponding to the appearance of vertebrates, with the NOXO1 and NOXA1 genes subsequently evolving as distinct regulatory subunits in vertebrates (Figure 1B). This appearance of NOXO1 and NOXA1 coincided with the emergence of Nox1 [1]. The pufferfish *T. rubripes *and *T. nigroviridis *are considered to have evolved from an *O. latipes *ancestor approximately 186 million years ago [64]. This tree suggests that NOXA1 and NOXO1 of *T. rubripes *and NOXA1 of *T. nigroviridis *have been lost during the decrease in genome sizes in these species (Figure 1B). In contrast to the co-appearance of p47*phox *and p67*phox *orthologs with the earliest Nox2 ortholog in *S. purpuratus*, p40*phox *appeared later, in vertebrates, and was not found in the genomes of either *C. intestinalis *or *S. purpuratus *(Figure 1B). The situation of the regulatory subunits of other primitive animals and a choanoflagellate is described below.

A homologue of p67*phox*/NOXA1 occurs in the slime mold amoeba (*D. discoideum*) [56] (Figure 1A). Fungal genomes of *Aspergillus nidulans *(*A. nidulans*), *Magnaporthe grisea *(*M. grisea*), and *Fusarium graminearum *(*F. graminearum*), all members of Ascomycota, also possess NOXR, a homolog of p67*phox *[55] (Figure 1A). However, *Saccharomyces cerevisiae *(*S. cerevisiae*) that also belongs to Ascomycota does not possess any Nox or NOXR orthologs [1,55] (Figure 1A). Some basidiomycetes [*Coprinopsis cinerea *(*C. cinerea*), *Laccaria bicolor *(*L. bicolor*), and *Postia placenta *(*P. placenta*)] also possess NOXR and NoxA/B genes (Figure 1A). The situation of the regulatory subunits of other fungi is described below. Interestingly, the fungi seem to lack precise homologues of p47*phox*/NOXO1 or its binding partner p22*phox *(Figure 1A). Recently, the possibility of p22*phox*-like gene in the *D. discoideum *genome was suggested [56], but it is not clear whether the gene represents an ancestral p22*phox*, a later adaptation or a functionally unrelated protein. We discuss the possibilities below based on a comparison of the amino acid sequences of amoebal p22*phox *and other p22*phox *orthologs. In addition, we identified other predicted proteins encoded in fungal and amoebal genomes that may function as (a) binding partner(s) of the Nox activator proteins (described below), suggesting the early evolutionary origin not only of the activator protein, but also possibly of a rudimentary organizer subunit.

### Synteny of NOXO1 ad NOXA1 genes in vertebrates

**Figure 2:**
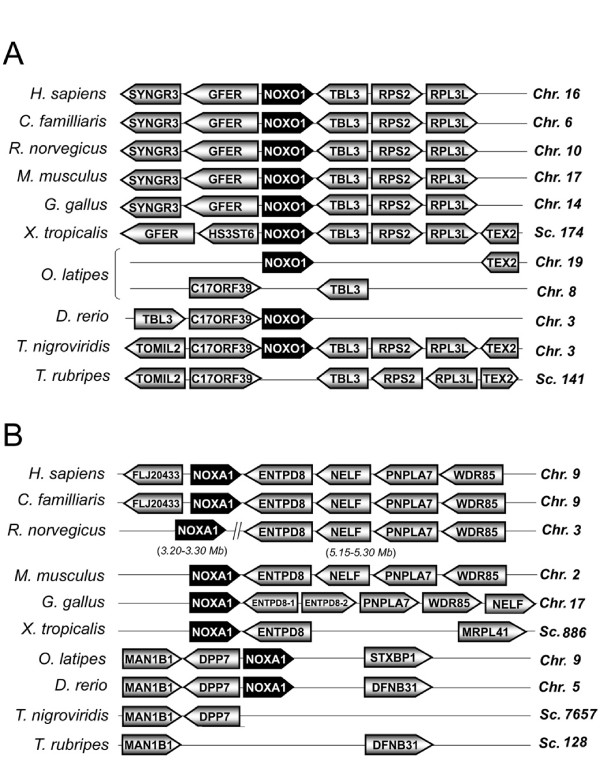
**Syntenies of NOXO1 and NOXA1 genes**. Syntenies of the indicated vertebrate NOXO1 (*A*) and NOXA1 (*B*) genes are shown. Genes are aligned in columns to illustrate orthology. Chromosome (Chr) or scaffold (Sc) number is indicated on the right. Abbreviations of gene names are shown in Additional file 2.

An extensive BLAST search has failed to identify the NOXA1 gene in the pufferfish *T. rubripes *and *T. nigroviridis *genomes, as indicated in Figure 1. In addition to the absence of NOXA1, *T. rubripes *seems to have lost the NOXO1 gene. To substantiate that the NOXO1 and NOXA1 genes were indeed absent, we compared chromosomal synteny (the preserved chromosomal order of genes among organisms) of NOXO1 in vertebrates. The human NOXO1 gene is preceded by SYNGR3 and GFER genes on chromosome 16 (Figure 2A, abbreviations of gene names are shown in Additional file 2), and followed by TBL3, RPS2 and RPL3L. Synteny was preserved in mammals and the chicken *G. gallus*. The frog *X. tropicalis *lacks SYNGR3, but other markers are preserved. Among fish, *T. nigroviridis *conserved part of the mammalian synteny: NOXO1, TBL3, RPS2, and RPL3L and instead of SYNGR3 and GFER, C17orf39 is positioned next to NOXO1. Synteny of the *T. rubripes *genome was very similar to that of *T. nigroviridis*, although it lacks a NOXO1 ortholog (Figure 2A). Two fishes, *D. rerio *and *O. latipes*, genomes possess NOXO1 genes, but with greater variations in synteny;although TBL3 and C17orf39 were present in the same chromosome as NOXO1, other linked markers were not seen (Figure 2A). The gene markers C17orf39 and TBL3 of *O. latipes *are present on a separate chromosome from that of NOXO1. Thus, the synteny analysis of the *T. rubripes *genomes shows that the NOXO1 gene was indeed lost during evolution.

The synteny of genetic markers surrounding NOXA1 was highly conserved in *H. sapiens*, *C. familliaris*, *M. musculus, G. gallus *(Figure 2B). Both NOXA1 and the linked markers of *R. norvegicus *were present in chromosome 3, with a long insert between NOXA1and ENTPD8 (Figure 2B). In the genome of *X. tropicalis*, synteny was more varied in that NOXA1 and ENTPD8 were present in the same scaffold, but other markers were not seen. Synteny of fish NOXA1 was preserved among the class but different from other vertebrates. *O. latipes *and *D. rerio *NOXA1 genes are preceded by MAN1B1 and DPP7 on chromosome 9 and 5, respectively (Figure 2B). *T. nigroviridis *possesses two markers MAN1B1 and DPP7, but lacks the NOXA1 ortholog. Synteny of *T. rubripes *is similar to that of *D. rerio*, but a NOXA1 ortholog is absent. Thus, the synteny of NOXA1 had considerable variation, and this analysis indicates that NOXA1 orthologs were lost during the evolution of the two pufferfish genomes.

### Identification of putative critical amino acids conserved among p47*phox*/NOXO1 orthologs

**Figure 3:**
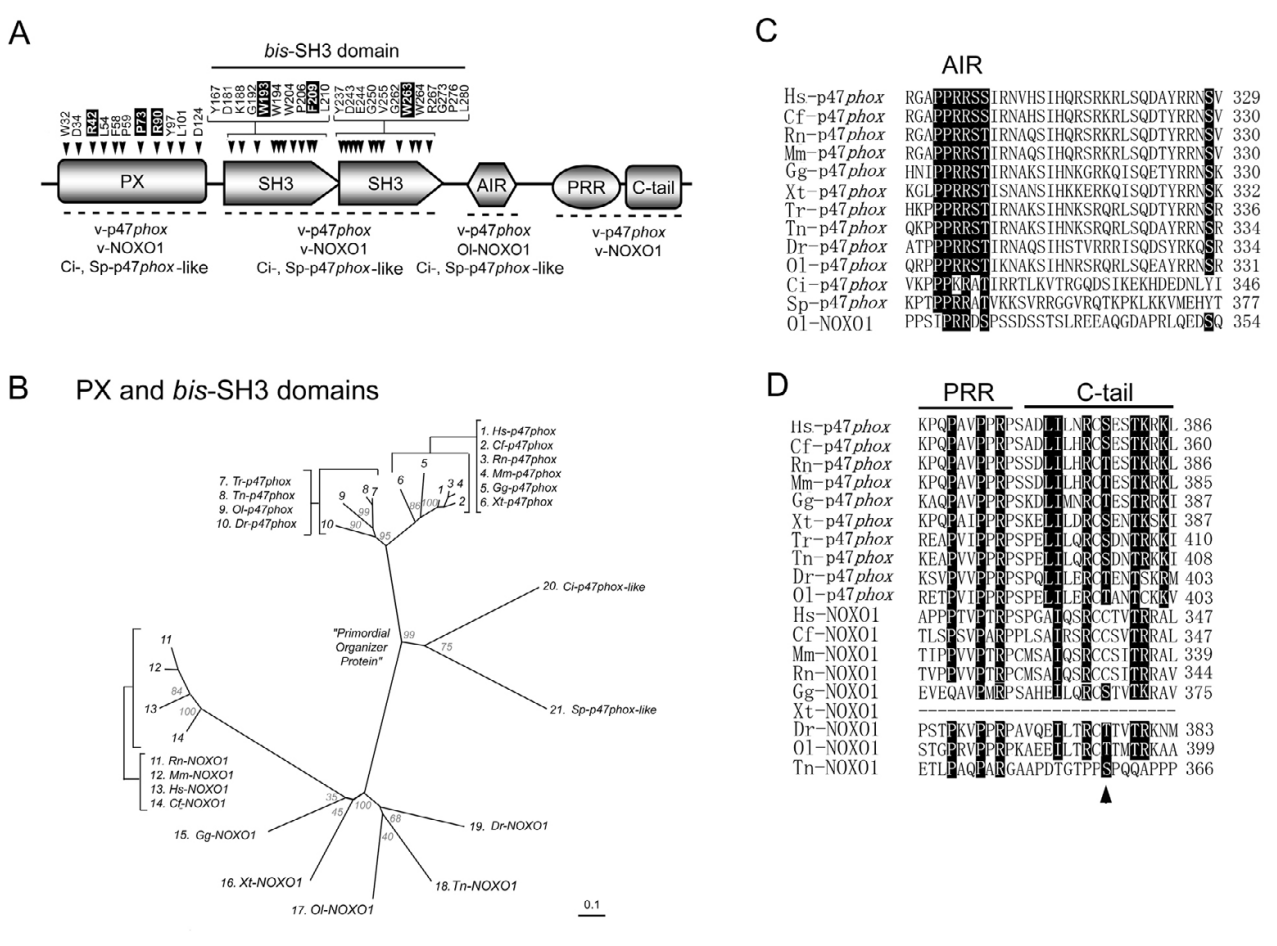
**Identification of highly conserved residues of Nox organizer proteins**. Species names were abbreviated as follows: v-, vertebrate; Hs, *H. sapiens*; Cf, *C. familliaris*; Rn, *R. norvegicus*; Mm, *M. musculus*; Gg, *G. gallus*; Xt, *X. tropicalis*, Dr, *D. rerio*; Tr, *T. rubripes*; Tn, *T. nigroviridis*; Ol, *O. latipes*; Ci, *C. intestinalis*; Sp, *S. purpuratus*. (*A*) Schematic domain structures of Nox organizer are shown; PX, *Phox *homology; SH3, the Src homology 3; AIR, autoinhibitory region; PRR, proline-rich region; C-tail, C-terminal tail region. Orthologs that possess these domains are shown below the domains. Amino acid residues indicate conserved amino acids in all Nox-organizers. Residue numbers correspond to those of the human p47*phox *protein sequence. Letters in *solid boxes *indicate the residues that have been previously proven by mutational analyses to be essential for the human Nox2 activation [24, 68, 126]. (*B*) Molecular taxonomy was created comparing by the sequence spanning the PX and *bis*-SH3 domains. (*C*) Alignment of amino acid sequences of the AIR. Letters in *solid boxes *indicate residues previously shown to be essential for human Nox2 activation, determined by mutational analyses [20] and also key residues responsible for interactions with the *bis*-SH3 domain, shown by X-ray crystallography [21]. (*D*) Alignment of amino acid sequences of the PRR and C-tail regions. Letters in *solid boxes *indicate the residues that have been shown by mutational analyses to be important for binding to p67*phox *[18, 72]. *An arrow head *indicates a residue corresponding to a phosphorylation site of human p47*phox *[19].

The PX domain of p47*phox *binds with highest affinity to phosphatidylinositol 3,4-bisphosphate [PtdIns(3,4)P_2_], and with lower but significant affinity to PtdIns(3,4,5)P_3_, PtdIns(3,5)P_2 _and PtdIns(3)P [23-25]. The PX domain of human NOXO1 shows a different lipid binding specificity, with highest affinity for PtdIns(3,5)P_2_, PtdIns(5)P, and PtdIns(4)P and lower affinity for PtdIns(3)P and PtdIns(3,4)P_2 _[41,65]. Both PX and *bis*-SH3 domains are seen in all p47*phox*/NOXO1 orthologs of deuterostomes (Figure 3A). Comparison of the sequences of the organizer proteins allowed identification of amino acid residues that are highly conserved (Figure 3A). Eleven highly conserved residues were identified in the PX domain, and 22 in the *bis*-SH3 domains (arrows in Figure 3A). Arg-42, Pro-73, and Arg-90 of the PX-domain and Trp-193, Phe-209, and Trp-263 of the *bis*-SH3 domain (human-p47*phox *numbering, black boxes in Figure 3A) were previously shown by mutational analyses to be essential for the activation of human Nox2 by p47*phox *[24,66-68].

While the PX and *bis*-SH3 domains are shared among all p47*phox *and NOXO1 orthologs including those of *S. purpuratus *and *C. intestinalis*, the molecular taxonomy of the regions (excluding the variable C-terminal regulator docking regions) reveals that vertebrate p47*phox *and NOXO1 families form distinct subgroups (Figure 3B), likely accounting, for example, for differences in lipid binding specificities [24,41]. The phylogenic tree supports an evolutionary model in which the p47*phox*-like proteins of *S. purpuratus *and *C. intestinalis *(#21 and 20 of Figure 3B, respectively) branch from a root that is closely related to the primordial ancestors of both p47*phox *(#1–10 of Figure 3B) and NOXO1 (#11–19 of Figure 3B).

To maintain p47*phox *strictly in an inactive state that cannot bind to p22*phox*, human p47*phox *masks its *bis*-SH3 domain with the AIR region [21,68,69], and this region is absent in human NOXO1 [38-41]. Therefore Nox2 activity is considered more tightly controlled than Nox1 activity. Most residues essential for the function of the AIR (e.g., Pro-299, Pro-300, Arg-301, Arg-302, Ser-303, Ser- 304 and Ser-328 of human p47*phox*), are well conserved among all vertebrate p47*phox *proteins (black boxes of Figure 3C). Interestingly *C. intestinalis *and *S. purpuratus *p47*phox *proteins possessed the AIR-like region (Figure 3C). *S. purpuratus *possesses two Nox2-like proteins Nox2A and 2B that are co-orthologs of vertebrate Noxes 1–3; the presence of a p47*phox *ortholog harboring the AIR suggests that this primitive Nox system functions analogously to the mammalian Nox2 system. All NOXO1 orthologs lack the AIR, with one exception, the teleost fish *O. latipes*, which retains the predicted AIR in both the p47*phox *and the NOXO1 genes (Figure 3C). Thus, this analysis supports an evolutionary model in which the NOXO1 "AIR-independent" Nox1 and Nox3 activation systems originated from the p47*phox*-like "AIR-dependent" Nox2 regulatory system. The *O. latipes *NOXO1, which contains the AIR, represents an evolutionary remnant reflecting the transition from AIR-dependence to -independence.

The heterodimerization of human p47*phox *and p67*phox *is dependent on the interaction between the C-terminal PRR of p47*phox *and the C-terminal SH3 domain of p67*phox *[40,70-72]. Both of these regions were conserved in vertebrate p47*phox *and p67*phox *orthologs, but not in orthologs of lower organisms (Figure 3A). The alignment of vertebrate p47*phox *and NOXO1 amino acid sequences demonstrates that in addition to the PRR, the C-terminal region immediately following the PRR ("C-tail" in Figure 3D) was also highly conserved. The PRR of p47*phox *isologs possesses four strictly conserved residues (Pro-363, Pro-366, Arg-368, Pro-369; human-p47*phox *numbering), and the C-tail region had an additional four identical amino acids (Ile-374, Arg-377, Cys-378, and Thr-382; human-p47*phox *numbering) (Figure 3D). Consistent with these identities, the essential binding roles of three residues (Pro-363, Pro-366, Arg-368, indicated by black boxes of Figure 3D) were previously demonstrated by mutational analyses [18]. The C-tail region is thought to enhance the interaction between the PRR of p47*phox *and the SH3 domain of p67*phox *[18], since simultaneous mutation of six of these residues (Leu-373, Ile-374, Arg-377, Thr-382, Lys-383, and Lys-385; human-p47*phox *numbering, indicated by black boxes of Figure 3D) significantly decreased the binding of the p47*phox *PRR to the p67*phox *SH3 domain [18,72]. Four of these residues (Ile-374, Arg-377, Thr-382, and Lys-383) are highly conserved among both p47*phox *and NOXO1 proteins, but two other residues (Leu-373 and Lys-385; human-p47*phox *numbering) were conserved only in p47*phox *orthologs (Figure 3D). This suggests that the binding affinities of p47*phox *and NOXO1 toward their respective activator protein SH3 domains may differ due to differences in these residues. Ser-379 in the C-tail region of human p47*phox *is a phosphorylation site [19]; the phosphorylation of which negatively regulates binding of the PRR and C-tail regions to the p67*phox *SH3 domain [72]. The threonine or serine residue is conserved at the position in all vertebrate p47*phox *and *G. gallus*, *D. rerio *and *O. latipes *NOXO1 (indicated by *an arrow head *in Figure 3D), indicating that NOXO1 proteins of these species retain this feature of p47*phox *which is subsequently lost in higher vertebrate NOXO1.

### *T. rubripes *C17orf39 ortholog as a candidate NOXO1-like protein

**Figure 4:**
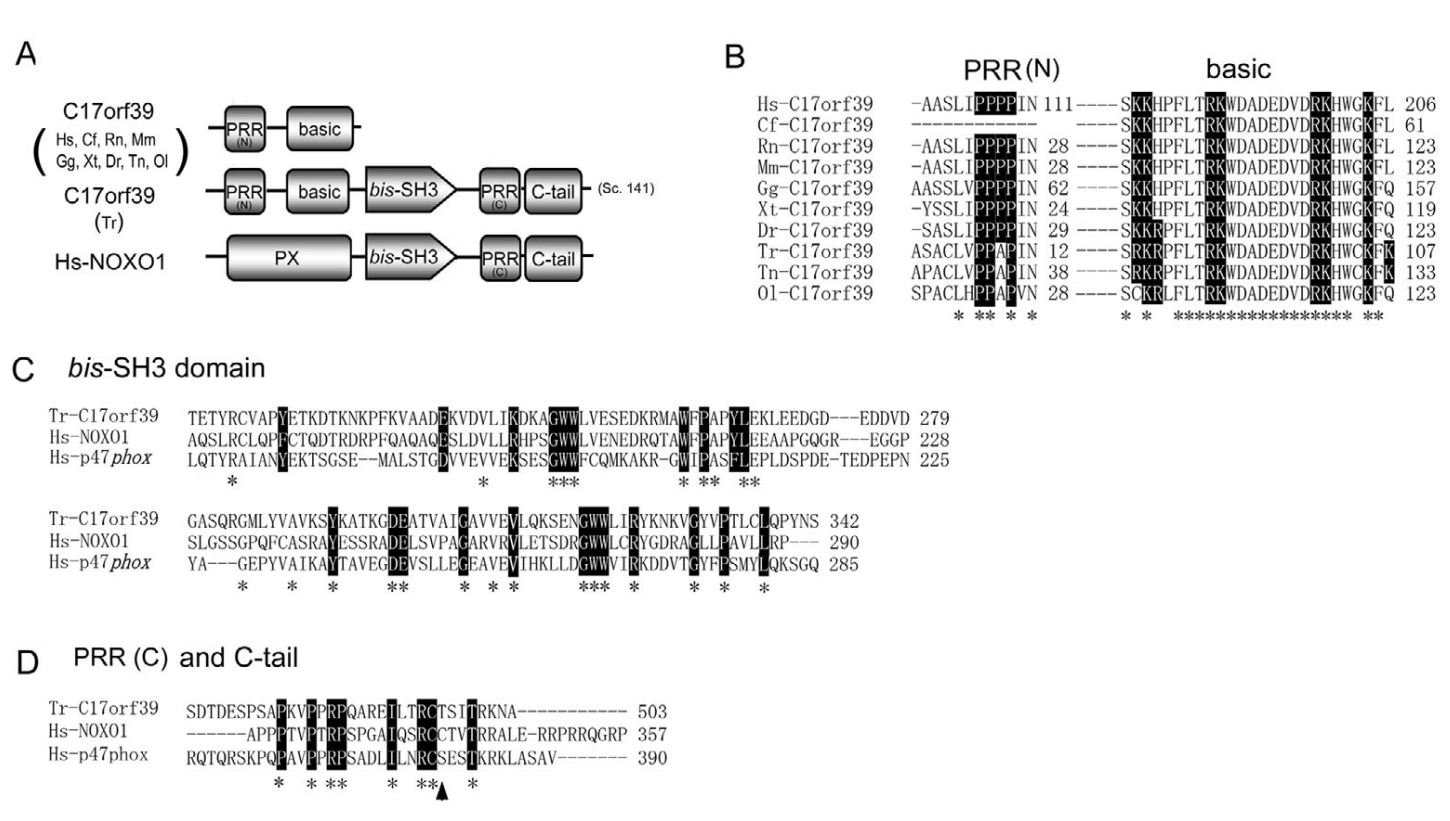
**Hypothetical function of *T. rubripes *C17orf39 protein as a Nox organizer protein**. (*A*) Schematic domain structures of C17orf39 and NOXO1. Names of orthologs that possess domains are shown to the left of structures. *T. rubripes *C17orf39 was located in scaffold 141 (*Sc 141*). Abbreviations of species names and most domains are shown in Figure 3; "basic" indicates a lysine/arginine-rich polybasic region. (*B*) Alignments of the PRR and basic regions of C17orf39. Letters in *solid boxes *indicate conserved the proline residues in the PRR and lysines and arginines in the basic region. (*C*) Comparison of *bis*-SH3 domain among *T. rubripes *C17orf39, human NOXO1, and human p47*phox*. (*D*) Comparison of PRR and C-tail regions among *T. rubripes *C17orf39, human NOXO1, and human p47*phox*. Letters in *solid boxes *indicate the evolutionally conserved residues that have been identified in Figure 3 (*C*, *D*). *An arrow head *indicates a residue corresponding to a phosphorylation site of human p47*phox*. Identical residues are shown by *asterisks *(*B*-*D*).

The NOXO1 gene seems to have been lost from the *T. rubripes *genome, as shown in Figure 2A. On the other hand, orthologs of chromosome 17 open reading frame 39 (C17orf39) gene predicted by the ensembl transcript model [73] are present adjacent to the NOXO1 genes in the *D. rerio *and *T. nigroviridis *genomes (Figure 2A). Amino acid sequences of these C17orf39 orthologs showed conserved PRR and polybasic regions (Figure. 4B, respectively, indicated by black boxes). In addition to the PRR and polybasic region, the *T. rubripes *C17orf39 ortholog has an extended region that encodes domains analogous to the C-terminal regions of NOXO1; these contain the *bis*-SH3 domain, PRR and C-tail region (Figure 4A). The sequence of *T. rubripes *C17orf39 possesses the 22 residues that are conserved in *bis*-SH3 domains of p47*phox *and NOXO1 (Figure 4C) and the eight key residues conserved in the PRR and C-tail regions (Figure 4D). A phosphorylation site corresponding to Ser-379 residue of human p47*phox *is also conserved in the *T. rubripes *C17orf39 (Figure 4D). Although the function of C17orf39 has not been established in any species, its NOXO1-like domain structure suggests that the C17orf39 ortholog of *T. rubripes *may function in place of the authentic NOXO1.

### Identification of putative critical amino acids conserved among p67*phox*/NOXA1 orthologs

**Figure 5:**
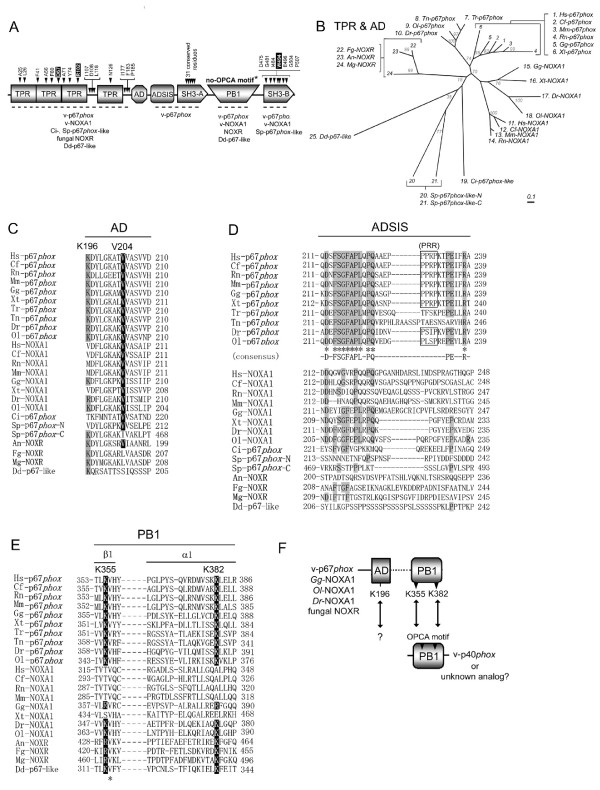
**Identification of highly conserved residues of Nox activator proteins**. Abbreviations of species names are shown in Figure 3. (*A*) Schematic domains structures of p67*phox *are shown; TPR, the tetratricopeptide repeats; AD, activation domain; ADSIS, AD-SH3 Intervening Sequence; PB1, *Phox*/Bem 1; OPCA motif, OPR/PC/AID motif. Abbreviations of other domain names are given in Figure 3. Names of orthologs that possess certain domains are shown below the domains. Amino acid residues indicate conserved amino acids in all indicated proteins, and the residue numbers correspond to that of human p67*phox*.*An asterisk *indicates that fungal NOXR possess the OPCA motif. Letters in *solid boxes *indicate residues previously proven by mutational analyses [27] and by X-ray crystallographic structure analysis [28] to be important for binding to Rac (TRP) or p47*phox *(SH3-B). (*B*) Molecular taxonomy was built by the length corresponding to the TPR and AD domains. Species names are abbreviated as shown in Figure 3 and as follows: An, *A. nidulans*; Mg, *M. grisea*; Fg, *F. graminearum*. (*C*-*E*) Alignments of the AD, ADSIS, and PB1 domains are shown. (*C*) Letters in *a solid box *indicate residues that have been proven by mutational analyses to be essential for activation of human Nox2 [30]. Letters in *a gray box *indicate basic residues that might correlate with a functional PB1 domain. (*D*) Amino acid residues identical among vertebrate p67*phox *ortholog sequences are indicated by *asterisks*. Letters in *gray boxes *indicate amino acid residues conserved in the putative ADSIS region. (*E*) Letters in *solid boxes *indicate the key basic residues required to bind to another PB1 domain, as shown by X-ray crystallographic structural analysis [32]. K355 and K382 indicate lysine residues of the human p67*phox*. "β1" and "α1" indicate the first β-sheet and the first α-helical structures of PB1 domain [32]. (*F*) Proposed model for a cooperation of a basic residue of AD (K196 of the human p67*phox *sequence) with a functional PB1 domain.

Alignment of the amino acid sequences of the 24 Nox-activator proteins (including amoebal and fungal isologs) identified 13 conserved amino acids in the TPR domains (Figure 5A). This includes two residues that have been identified as essential for binding human p67*phox *to Rac, as determined by the crystal structure of the complex [21] (Figure 5A, filled boxes). The AD was first identified by mutagenesis of p67*phox *[30]. Val-204 of human p67*phox *and the corresponding residue Val-205 of human NOXA1 are crucial for respective activation of Nox2 and Nox1 [30,41]. All orthologs of p67*phox *and NOXA1, and one fungal NOXR (*A. nidulans*-NOXR) conserve this valine residue (Figure 5C). The two fungal NOXR (*M. grisea *and *F. graminearum*) retained the hydrophobic amino acid leucine instead of valine at this position (Figure 5C). Similarly, the second AD-like region of *S. purpuratus *p67*phox*-like protein (Sp-p67*phox*-C) conserved an isoleucine residue at this position. In contrast, Dd-p67-like protein lacked a typical AD sequence (Figure 5C). The N-terminal region containing the TPR and AD can be considered as the "Nox-activating region", a module that cooperates with Rac to activate Nox enzymatic activity [11,29-31,74]. Taxonomy of the "Nox-activating region" (corresponding to residues 1–212 of human p67*phox*) reveals distinct subgroups corresponding to NOXA1 and to p67*phox*, and shows that the NOXR proteins of fungi also represent a distinct subgroup (Figure 5B). A fourth rather diverse subgroup is comprised of p67*phox*-like proteins that include those of *S. purpuratus *and *C. intestinalis *(numbers 19–21 in Figure 5B). Taken together with the taxonomy of p47*phox *(Figure 3B) and of Noxes [1], these data support an evolutionary model in which duplication of both the p67*phox *and p47*phox *genes occurred in vertebrates at a time corresponding to the appearance of Nox1. In contrast to fungal NOXR, Dd-p67-like protein appeared to branch from a root shared with sea urchin-p67*phox*-like genes (Figure 5B); however, the bootstrap value was only 35% raising uncertainty as to which subgroup the amoebal p67-like protein belongs. Nevertheless, amoebal p67-like protein is clearly distinct from the NOXR group.

In contrast to the C-terminal SH3 domain (SH3-B) of activator proteins which is seen in all vertebrate activator proteins, the central SH3 domain (SH3-A) was seen only in vertebrate p67*phox *(Figure 5A). Comparison of sequences of all vertebrate p67*phox *orthologs identified 31 highly conserved amino acids in this SH3 domain (Figure 5A and Additional file 3). In addition, a region between AD and SH3-A (termed here the ADSIS region for " AD-SH3 Intervening Sequence") was strictly conserved among vertebrate p67*phox *isologs, but was not seen in others, such as NOXA1 and NOXR. A consensus sequence was identified in this region (Figure 5D). A PRR-like region (residues 227–231 of human p67*phox*) was seen in ADSIS region, but it does not conserve in all vertebrate p67*phox *isologs based on the deduced sequences (Figure 5D). The role of the putative region is currently unknown, but may involve a function related to SH3-A because it coincides with the presence of SH3-A. Despite the high conservation of SH3-A among vertebrate p67*phox *orthologs, the role of this domain is not defined [10,75].

The PB1 domain was present in all vertebrate p67*phox*, in vertebrate NOXA1 and in fungal NOXR and amoebal p67-like protein, but not in *C. intestinalis *and *S. purpuratus *p67*phox*-like proteins (Figure 5A). The crystal structure of the heterodimer between the PB1 domains of p67*phox *and p40*phox *domain demonstrated that Lys-355 and Lys-382 in human p67*phox *are essential to bind the PB1 domain of p40*phox *[32,76]. Residues corresponding to Lys-355 and Lys-382 of human p67*phox *are conserved not only in the vertebrate p67*phox*, but also in the NOXA1 orthologs of chicken (*G. gallus*) and fish (*D. rerio *and *O. latipes*) (Figure 5E). This suggests that in addition to its well-described role in activating Nox2, p40*phox *probably also participates in activating Nox1 in chicken and fish (Figure 5E). On the other hand, NOXA1 of *X. tropicalis *has lost both of these p40*phox*-interacting residues. Thus, these data support an evolutionary model in which p40*phox *originally participated in regulating *both *Nox1 and Nox2 in vertebrates, but after the appearance of mammals, Nox1 (but not Nox2) became independent of p40*phox*. The lack of a PB1 domain in both *C. intestinalis *and *S. purpuratus *p67*phox*-like proteins is consistent with the absence of p40*phox *orthologs in these species (Figure 1B).

The analysis of PB1 domain sequences in Nox activators also suggests an unexpected relationship between the AD and the PB1 domain. AD regions of all vertebrate p67*phox *contain a positively charged residue, *e.g*., Lys-196 of h-p67*phox *(indicated by *gray boxes *in Figure 5C). This basic residue is also completely conserved in NOXA1 orthologs of *G. gallus, D. rerio *and *O. latipes *(Figure 5C), which retain functional PB1 domains (Figure 5E), but not in the other NOXA1 and p67*phox*-like proteins (Figure 5C) (with the single exception of the C-terminal AD-like region of *S. purpuratus *p67*phox*-like protein). Interestingly, the fungal NOXR, which possess these key basic residues in their PB1 domains (Figure 5E), also conserves this basic residue in their AD regions (Figure 5C). Thus, the presence of a functional PB1 domain correlates strongly with the presence of a positively charged residue corresponding to position 196 in the human p67*phox *sequence, and may point to the functional cooperation of these two regions, e.g. in binding to p40*phox *or other functionally analogous proteins in fungi (Figure 5F). Alternatively, the positively charged residue in AD region might be related to the tail-to-tail interaction between vertebrate p47*phox *and p67*phox *(and NOXO1-NOXA1 of early vertebrates) because a sequence analysis of Nox organizers that is shown in Figure 3D suggests that the conserved residue also co-occurs with the phosphorylation site in the C-tail region of all vertebrate p47*phox *and early vertebrate NOXO1. Thus, co-appearance and co-disappearance of evolutionarily conserved residue provides a hint to find an unknown relationship between regulatory-subunits.

SH3-B was seen in all vertebrate p67*phox *and NOXA1 orthologs and in *S. purpuratus *p67*phox*-like protein (Figure 5A). Among these orthologs, seven conserved amino acid residues in SH3-B were identified (Figure 5A), and the residues include a tryptophan (Trp-494; human p67*phox*- numbering) that was previously shown to be crucial for interaction with the C-terminal PRR of Nox organizer proteins [40].

### Identification of putative critical amino acids conserved among p40*phox *orthologs

**Figure 6:**
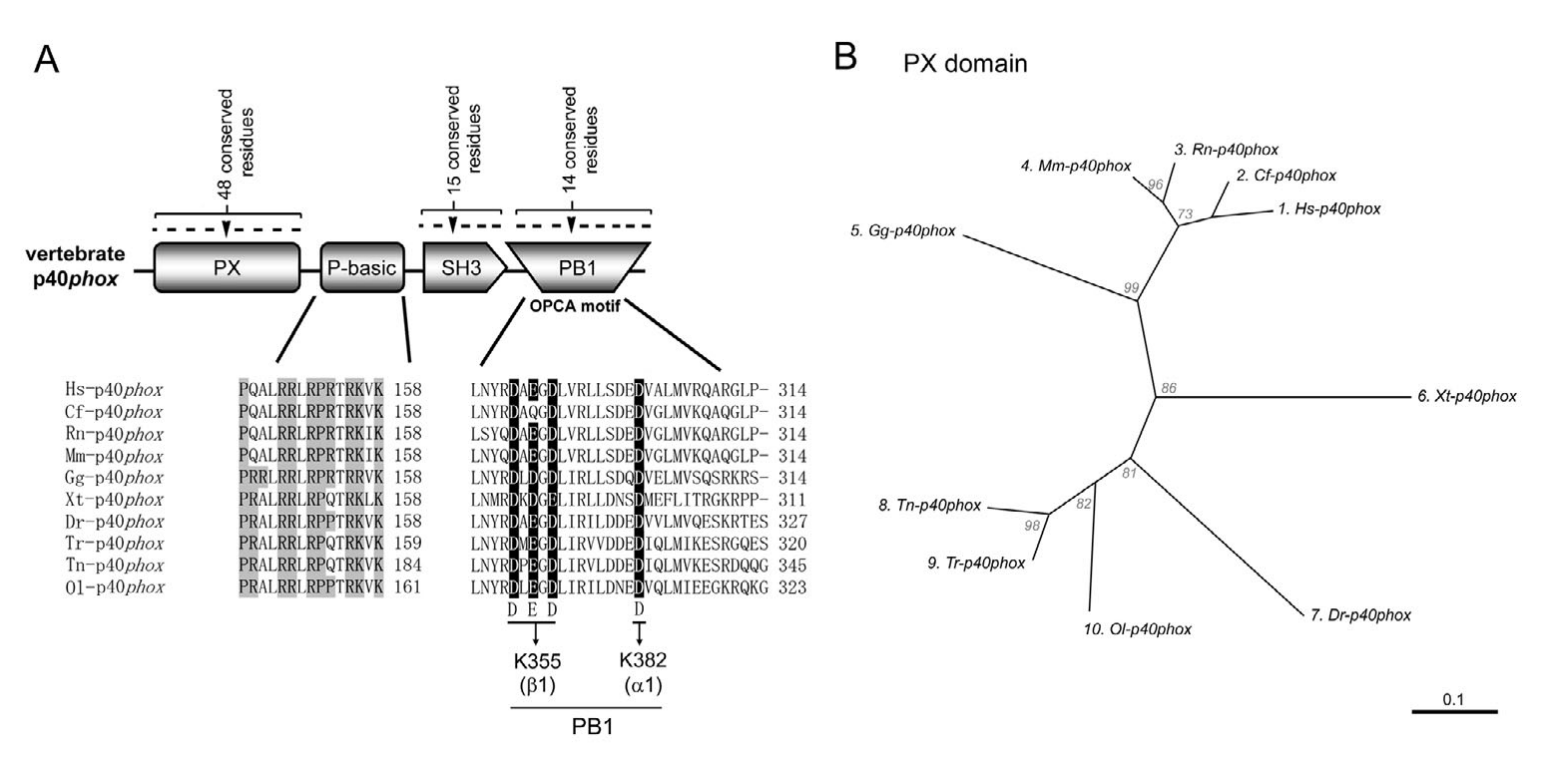
**Identification of highly conserved residues of p40*phox***. (*A*) A schematic structure of p40*phox *domains is shown; proline-basic region, P-basic. Abbreviations of domain names are described in legends to other Figures. Letters in *gray boxes *indicate highly conserved proline and basic amino acid residues in the P-basic region. Letters in *solid boxes *indicate key acidic residues of OPCA motif that have been demonstrated by X-ray crystallographic structure [32]. K355 and K382 indicate two residues of human p67*phox *that interact with the OPCA motif. (*B*) Molecular taxonomy was generated by the alignment of amino acid sequences of PX domain. Species name abbreviations are given in Figure 3.

Mammalian p40*phox *is considered to be an enhancer of Nox2 activation [33,35,77,78]. p40*phox *orthologs emerged in vertebrates (see Figure 1B) coincident with the appearance of the PB1 domain of p67*phox*-like proteins. Fungal NOXR and amoebal p67-like proteins also possess the PB1 domains (Figure 5E), but their genomes do not encode a distinct p40*phox *ortholog. The protein sequences of vertebrate p40*phox *orthologs are highly conserved; the canonical PX, SH3, and PB1 domains are all seen among vertebrate p40*phox *orthologs (Figure 6A). Alignment of ten vertebrate p40*phox *orthologs showed 48 conserved amino acid residues in the PX domain (see Additional file 3), including Phe-35, Arg-57, Tyr-59, Leu-65, Arg-105 (human p40*phox *numbering) that have been demonstrated to be essential for phospholipid-binding [23,24,79,80]. Phylogenic analysis using the trimmed PX domain sequences (Figure 6B) does not reveal obvious sub-families. Recently a novel intramolecular interaction in p40*phox *between the PX and PB1 domains was reported [81]. The interface between the PX and PB1 domains consists of a hydrophobic pocket by Phe-35 of the PX domain and Val-257, Pro-265, Leu-273 and Phe-320 of the PB1 domain and ionic interactions between His-38, Arg-58, and Arg-60 of the PX domain and Glu-259 and Asp-269 of the PB1 domain (human p40*phox *numbering) [82]. The residue corresponding to the Leu-273 is much varied among vertebrate p40*phox *proteins (see Additional file 3), suggesting that the residue is less crucial than that previously proposed if the interaction is important in other vertebrates than human. The residue to His-38 is often replaced by tyrosine. The majority of other residues are conserved, but there are some exceptions: residues corresponding to Arg-60 in *X. tropicalis *p40*phox *and to Glu-259 and Asp-269 in rat p40*phox *(see Additional file 3).

To the best of our knowledge, a role for the SH3 domain of p40*phox *has not been defined [10]. Eighteen amino acids are conserved in the SH3 domain (see Additional file 3). Between the PX and SH3 domains, we identified a new conserved region of 15 amino acids that is rich in proline, arginine and lysine, and is therefore designated the "proline-basic (P-basic) domain" (Figure 6A). This putative region is not essential for PtdIns(3)P-binding by the p40*phox *PX domain [25,80]. An intramolecular interaction between the PX and PB1 domains seem not to be related to P-basic region according to the structure of full length p40*phox *[81,82]. The structural analysis demonstrates that P-basic region forms a separate motif and is situated closely to the PX domain [82], indicating that it might support the cell membrane-binding function of p40*phox *together with the PX-domain. Alternatively, the P-basic region might interact with the SH3 domain of p40*phox *in a manner similar to the interaction between the AIR and the tandem SH3 of human p47*phox*. Interestingly, Thr-154 of human p40*phox*, located in the center of P-basic region, has been identified as a phosphorylation site [16], and this residue is entirely conserved among all vertebrate p40*phox *orthologs (Figure 6A).

The PB1 domain of human p40*phox *harbors acidic amino acids forming the "OPCA (OPR/PC/AID) motif". An OPCA motif contains four distinctive acidic residues that are essential for binding to conserved basic residues in the p67*phox *PB1 domain [32]. The four acidic residues, Asp-289, Glu-291, Asp-293 and Asp-302 (human p40*phox *numbering) are conserved in all orthologs with the exception of a glutamine rather than a glutamic acid in the position of *C. familliaris *p40*phox *that corresponds to Glu-291 of human p40*phox *(Figure 6A).

### Identification of putative critical amino acids conserved among p22*phox *orthologs

**Figure 7:**
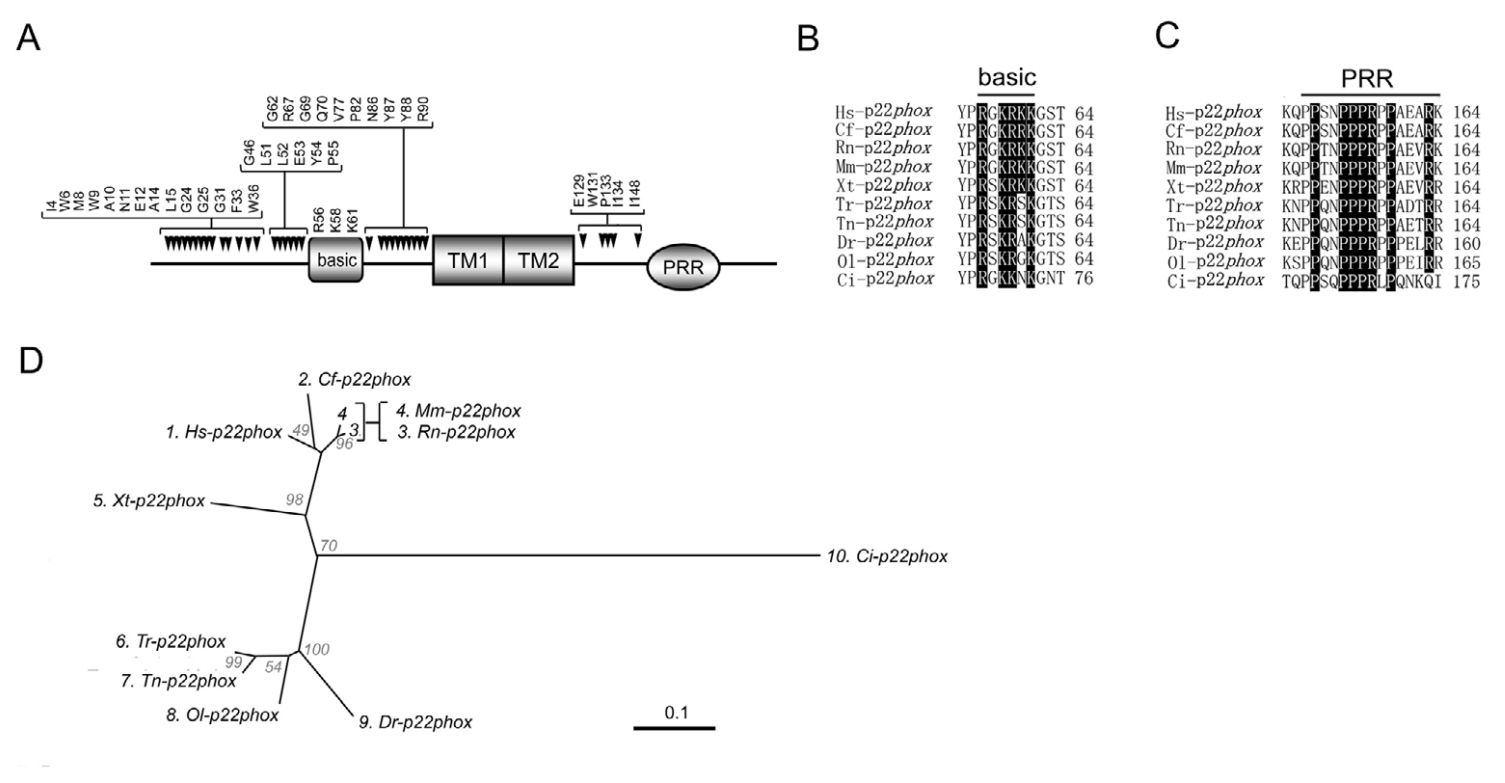
**Identification of highly conserved residues of p22*phox***. (*A*) Schematic domains structures of p22*phox *are shown; TM1 and TM2 represent the predicted 1^st ^and 2^nd ^transmembrane region; PRR, proline-rich region; basic, a lysine- and arginine-rich conserved domain. Indicated above the schematic structure are putative key amino acid residues that are conserved in p22*phox *orthologs, and the residue numbers correspond to those of human p22*phox*. Detailed alignment of the polybasic region *(B) *and the PRR *(C) *are shown. Letters in *solid boxes *indicate the residues that have been proven by mutational analyses to be important for binding to p47*phox *and/or activation of human Nox2 [53, 75, 127, 128]. (*D*) Molecular taxonomy was generated based on the alignment of amino acid sequences of full-length p22*phox*. Species name abbreviations are given in Figure 3.

Human p22*phox *contains two predicted transmembrane regions [83] and forms a stabilizing heterocomplex with Nox2 as well as Nox1, Nox3, and Nox4 [51-53]. The PRR of p22*phox *is the only motif in this protein whose role in Nox activation has been demonstrated [3,10]. The PRR is highly conserved among all p22*phox *orthologs (Figure 7A, the sequences are provided in Additional file 4), consistent with its role as a docking site for the *bis*-SH3 domain of organizer proteins. In addition, a total of 38 amino acids were identical in all p22*phox *sequences (Figure 7A), suggesting important additional functions, particularly for the N-terminal regions preceding TM1. Within these conserved residues, we identified a region rich in arginine and lysine (Figure 7B), and is therefore designated the polybasic region (indicated as "basic" in Figure 7A). A short sequence containing multiple basic amino acid residues has been proposed to contribute to recruitment of proteins to membrane sub-regions and to function in receptor-internalization via interaction with phospholipids [84-86]. According to our preliminary data, mutation in the polybasic region of p22*phox *did not affect extracellular ROS production from Nox2-transfected human embryonic kidney 293 cells (T. Kawahara and J. D. Lambeth, unpublished observations). This implies that the putative conserved region might function in a role unrelated to the enzymatic activity of Nox2, *e.g*. in phagocytosis or receptor-mediated endocytosis.

As shown in Figure 1, a p22*phox*-like protein was not identified in *S. purpuratus*, but because the genome sequence of this organism is incomplete and because a p47*phox*-like protein with a conserved *bis*-SH3 domain is present, we suggest that this organism probably possesses an undiscovered p22*phox*-like protein, or a protein that serves an analogous function. Our analysis did not provide convincing evidence for a p22*phox *isolog in slime mold amoeba. Lardy *et al*. argued that this organism may possess a membrane-associated p22*phox-*like protein (GenBank™ accession number AY221170); predicted p22*phox*-like gene-null *D. discoideum *mutant is unable to produce spores, similar to *nox*-null mutants [56]. However according to our analysis, this protein is highly divergent in amino acid sequence compared with the consensus sequence obtained from all other organisms, showing disagreement in 24 out of the 38 conserved residues shown in Figure 7A. Thus, further investigation is needed to judge whether the gene emerged as a later adaptation or whether it represents a primitive p22*phox *gene.

### Molecular evolution of domains of Nox organizers and activators in Deuterostomia

**Figure 8:**
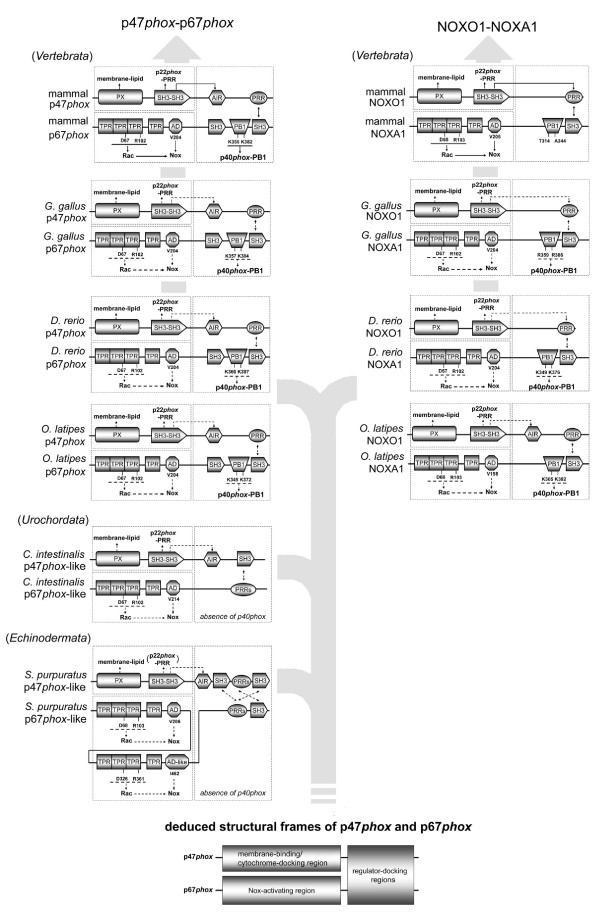
**Evolution of domains of p47*phox */NOXO1 and p67*phox */NOXA1 orthologs**. Shown is a schematic representation of identified domains and known or predicted inter- and intra-molecular interactions among p47*phox*/NOXO1 and p67*phox*/NOXA1 orthologs in "mammals" (*H. sapiens*), *G. gallus*, *D. rerio*, *O. latipes*, *C. intestinalis*, and *S. purpuratus*. *Solid lines *indicate interactions that have been demonstrated experimentally. *Broken lines *indicate predicted or proposed interactions based on sequences analysis and correspondence to experimentally demonstrated interactions in other species. The inferior diagram indicates deduced generalized structural features of p47*phox- *and p67*phox-*like proteins. Abbreviations of domain names are given in legends to other Figures.

As described above, comparisons of the amino acid sequences of the Nox regulatory subunit orthologs revealed certain features of the evolution of characteristic domains. Some key residues and domains are summarized in Figure 8, which shows the proposed evolution of organizer and activator proteins. The p47*phox *orthologs of all vertebrates (top four left-hand panels in Figure 8) have identical domain structures, which include the PX domain, *bis*-SH3 domain, AIR, and PRR. On the other hand, the domain composition of NOXO1 (right-hand panels in Figure 8) changed during the evolution from fish to tetrapods: the NOXO1 orthologs of tetrapods and fish all possessed the PX domain, *bis*-SH3 domain, and PRR. However, the predicted AIR (including key conserved residues, for details, see Figure 3C) is also present in *O. latipes *but is absent in *D. rerio *and tetrapods (right-hand panels in Figure 8). Despite the presence of the AIR in *O. latipes *NOXO1, it is clearly distinguished by sequence homology and synteny from a p47*phox *isolog, as shown in Figure 3B. Thus, initially, following gene duplication from p47*phox*, NOXO1 retained the AIR regulatory feature of p47*phox*, and this feature was subsequently lost.

Like vertebrate isologs of p47*phox*, the p47*phox*-like proteins of *C. intestinalis *and *S. purpuratus *possess the PX domain, *bis-*SH3 domain, and AIR. However, these isologs differ considerably in the C-terminal regions that, in higher forms, interact with p67*phox *isologs. The C-terminus of the *C. intestinalis *p47*phox *lacks the PRR, but motif scanning by PROSITE [87] demonstrates the p47*phox*-like protein contains, instead, additional SH3 domain [residues 340–399 of Ci-p47*phox *protein, GenBank™ No. NM_001033828]. The *S. purpuratus *p47*phox*-like protein also lacked the C-terminal PRR, but instead had two additional SH3 domains (residues 367–430 and 561–618 of Sp-p47*phox*, GenBank™ No. XP_001183696) separated by two PRRs (residues 453–460 and 476–484 of Sp-p47*phox*) (bottom two left-hand panels in Figure 8).

All vertebrate orthologs of p67*phox *have the same domains seen in human p67*phox *(Figure 8, left panels). Importantly, all p67*phox *and NOXA1 proteins possess the AD region, which has been shown to be critical for activating superoxide generation in Nox2 and Nox1 [30,31]. The TPR motif is present in all species, and the two critical residues for Rac-binding, Asp-67 and Arg-102 of human p67*phox *[21], are conserved among all vertebrate isologs of p67*phox *and NOXA1 (top four panels in Figure 8), supporting the idea that binding to Rac is a conserved feature of vertebrate p67*phox *andNOXA1. The N-terminal domain structures of *C. intestinalis *and *S. purpuratus *p67*phox*-like proteins are similar to those of vertebrate p67*phox *and NOXA1 (Figure 8). Both p67*phox*-like proteins possess a TPR domain retaining the two critical Rac-binding residues (Figure 8). In addition, *S. purpuratus *p67*phox*-like protein possesses a second set of TPR and AD-like domains. Both TRP domains retain conserved essential Rac-binding residues, but the second AD-like domain was not well conserved as shown in Figure 5C. Thus, Rac binding is likely to be an evolutionarily conserved feature among all p67*phox*/NOXA1 proteins.

In mammals, binding between the PB1 domains of p67*phox *and p40*phox *has been shown to mediate heterodimer formation. The crystal structure of the heterodimer of the PB1 domains from p67*phox *and p40*phox *[32] demonstrated that Lys-355 and Lys-382 in human p67*phox *are essential to bind to the PB1 domain of p40*phox*, and mutation of Lys-355 of human p67*phox *abolished binding to p40*phox *[76]. These crucial residues are entirely conserved in the PB1 domains of all vertebrate p67*phox *proteins, which imply that they all bind to p40*phox *(top four left-hand panels in Figure 8). In contrast to vertebrate p67*phox*, mammalian NOXA1 does not retain these two key amino acids (a top right-hand panel of Figure 8), explaining the failure of NOXA1 to bind to p40*phox *[40]. However, the chicken *G. gallus*, and the fish *D. rerio *and *O. latipes *NOXA1 completely conserve these two residues, and an OPCA motif of p40*phox *(Figure 6), which is essential for binding to the p67*phox *PB1 domain are highly conserved. The conservations of key residues suggest that in these fish, binding to p40*phox *may be retained (from the second to fourth panels in right hand of Figure 8). Thus, the loss of function of p40*phox *can be traced in the evolution of NOXA1. Both *C. intestinalis *and *S. purpuratus *p67*phox*-like proteins lack a PB1 domain, consistent with the absence of p40*phox *orthologs.

The PX-domain of human p40*phox *specifically binds to PtdIns(3)P [23,24,79,80]. Therefore, p40*phox *accumulates during phagocytosis at early endosomes, which are rich in PtdIns(3)P [35,81,88]. *T. rubripes *p40*phox *is expressed in a wide range of tissues including the gastrointestinal tissues [89], while Nox2 expression is restricted to the blood and kidney. Although Nox1 expression in fish or the other non-mammal organisms is not clear, our analysis suggest that Nox1 of fish and birds might function, along with the p40*phox *orthologs, in endocytosis, *e.g*., in receptor-internalization. This novel relationship between Nox1 and p40*phox *may provide a clue to understanding the physiological role of Nox1 in vertebrates.

The vertebrate p67*phox *and NOXA1 orthologs all have the C-terminal SH3 domain that, in human, mediates binding to the PRR of p47*phox *and NOXO1, respectively. This supports the idea that all of the vertebrate forms of these proteins mediate protein interactions via tail-to-tail binding between the SH3 domain of the activator protein and the PRR of the organizer protein. We further suggest that even though the structures of the C-termini of p47*phox*- and p67*phox*-like proteins in *C. intestinalis *and in *S. purpuratus *differ from the vertebrate proteins, the same sort of tail-to-tail binding of these p67*phox*-like proteins to the corresponding p47*phox*-like proteins occurs as diagrammed at the bottom of Figure 8. For example, we suggest that the C-terminal putative PRRs of *C. intestinalis *p67*phox*-like proteins (residues 256–348 of Ci-p67*phox *protein, GenBank™ No. NM_001033827) binds to the C-terminal SH3 domain of the p47*phox*-like protein as indicated by a broken line in Figure 8, reversing the mode of interaction seen in the vertebrate proteins but accomplishing the same function. Similarly, SH3 domains and putative PRRs in the C-terminus of *S. purpuratus *p47*phox*-like protein may interact with the PRRs and the SH3 domains in the C-terminus of *S. purpura*tus p67*phox*, respectively. Thus, while domains in the C-termini of p47*phox *and p67*phox *orthologs are not conserved during evolution, the model shown in Figure 8 suggests that the "docking function" has been retained. The model suggests three functional modules within the organizer protein/activator protein complex: 1), a "membrane-binding/cytochrome docking region" on the organizer protein that is comprised of the phospholipids-binding PX domain and the tandem SH3 region that binds to the PRR of p22*phox*; 2) a "Nox-activating region" that contains the Rac-binding TPR domain and the AD, which together cooperate to stimulate the activity of the Nox catalytic domain; and 3) a "regulator-docking region" which uses evolutionarily variable but paired docking modules that link the "membrane-binding/cytochrome docking region" of p47*phox *to the "Nox-activating region" of p67*phox *(bottom panel in Figure 8).

### Occurrence and origin of *Phox*-like regulatory subunits during evolution of Metazoa and Choanoflagellata

**Figure 9:**
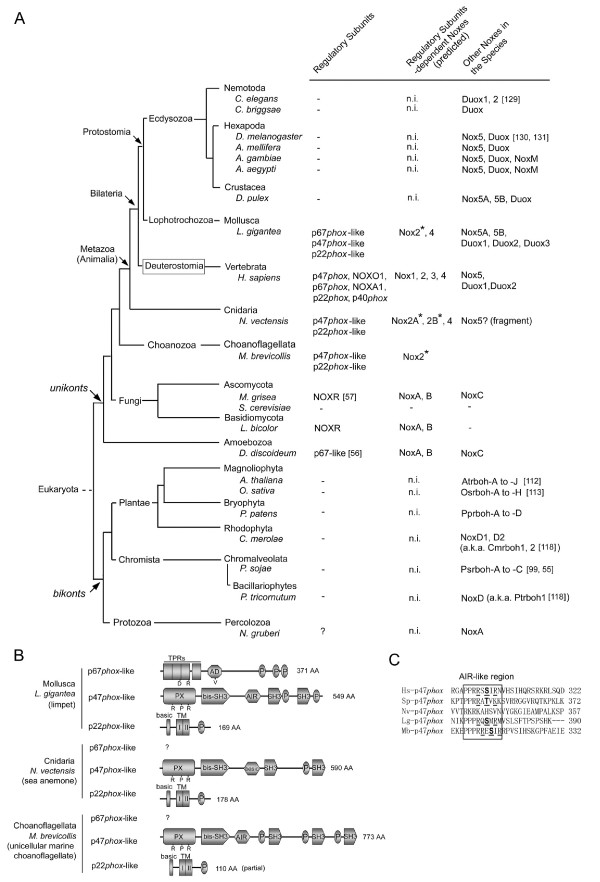
**Relationship between the evolution of eukaryotes and the occurrence of *Phox *-related regulatory subunits**. (*A*) A phylogeny of eukaryotes was created based largely on genetic information [90, 100]. Branch lengths are for illustrative purposes and are not proportional to phylogenetic distances of groups. Details of deusterostomes are shown in Figure 1. "n.i." indicates none identified or reported. "co-ortho. of v-Nox1-3" indicates that the gene is a co-ortholog ancestor of all members of the vertebrate Nox1-3 subgroup. The number in a parenthesis (e.g. [129, 130, 131]) indicates a reference number. (*B*) Predicted domains structures of newly identified putative regulatory subunit-like proteins are shown; P, proline-rich region. The sequences are provided in Additional file 7. Abbreviations of domain names are described in legends to other Figures. (C) Sequences of putative AIR-like region are shown. *Underlines *indicate residues that agree with a consensus sequence of protein kinase C substrate. *Bold letters *indicate predicted phosphorylation sites in AIR-like regions. Abbreviations: Hs, *H. sapiens*; Sp, *S. purpuratus*; *Nv, N. vectensis*; Lg, *L. gigantea*; Mb, *M. brevicollis*.

In addition to regulatory subunit-dependent Nox proteins of the Deuterostomia, we assembled predicted regulatory subunits and the subunit-regulated Nox isolog sequences present in the genomes of following species: the sea anemone *Nematostella vectensis *(*N. vectensis*), the limpet *Lottia gigantea *(*L. gigantea*), the marine choanoflagellate *Monosiga brevicollis *(*M. brevicollis*) (see Additional files 5, 6, 7, 8). According to the recent phylogeny based on small ribosomal subunit gene and other molecular tools [90], the bilaterian animals consist of three clades: Deuterostomia, Lophotrochozoa, and Ecdysozoa. A gastropod snail, the limpet *L. gigantea*, belongs to the Lophotrochozoa, and p67*phox*, p47*phox*, p22*phox*-like genes are found in this genome (Figure 9A). The predicted motif structures by the Pfam program are shown in Figure 9B; characteristic motifs of p67*phox *(TPR domain and AD-like region) and p47*phox *(PX domain and AIR-like region) as well as the key residues (detailed alignments are shown in Additional file 8) are conserved. The genome of *L. gigantea *also possesses an ancestral Nox2 gene as a co-ortholog of vertebrate Nox1-3 (Figure 9A and see Additional file 6) like Nox2 isologs in primitive deuterostomes [1]. On the other hand, any distinct regulatory subunit genes were not found in seven species that belongs to Ecdysozoa including two nemotodes (*Caenorhabditis elegans *and *Caenorhabditis briggsae*), four insects (*Drosophila melanogaster*, *Apis mellifera*, *Anopheles gambiae*, *Aedes aegypt*), and one non-insect arthropod (the waterflea *Daphnia pulex*) (Figure 9A).

Before the emergence of bilateral animals, cnidarians split from the main animal lineage and the genome retained many of the features present in last common ancestor of animals [90]. The starlet sea anemone *N. vectensis *that belongs to the Cnidaria, conserves p47*phox*-, p22*phox*-like genes, and two ancestral Nox2 genes, Nox2A and 2B (Figure 9A). The characteristic motifs of the putative regulatory subunits are predicted by the Pfam program and shown in Figure 9B (alignments are shown in Additional file 8). *N. vectensis *p47*phox *possesses PX and *bis*-SH3 domains. The isolog also possesses a putative polybasic region within the sequence corresponding to the AIR of the human p47*phox *gene. A polybasic region is a class III consensus motif for binding to a SH3 domain [91]; therefore, this may implicate the polybasic region as a prototype of the AIR. Although the current version of the *N. vectensis *genome does not encode an obvious p67*phox*-like gene, the presence of multiple SH3 domains in *N. vectensis *p47*phox *points to an unidentified p67*phox *ortholog or an unknown PRR-containing binding partner. Although cnidarians have no specialized immune cells, the *N. vectensis *genome possesses orthologous genes to Toll/Toll-like receptors, complement C3-like molecules, and a redox-sensitive transcription factor nuclear factor-κB that are orthologs of key components of innate immunity in vertebrates [92]. Further study needs to identify an original role of *Phox*-type NADPH oxidase in the primitive multicellular animals.

One clear implication of these genomic and detailed sequence analyses described above is that the hypothetical ancestor of primitive animals might conserve the *Phox*-type NADPH-oxidase system. In the middle of the nineteenth century, a striking structural resemblance between the collar cells, so-called "choanocytes", of primitive animal sponges and a group of protists the choanoflagellates has been noted ([93], reviewed in [94]). A line of recent phylogenic analyses suggests that the metazoa including sponges, cnidarians, and other animals constitutes a monophyletic clade closely related to the Choanoflagellata [90,95,96]; therefore, the choanoflagellates are considered to be the organisms that are close in evolution to the all modern animals. The unicellular marine choanoflagellate *M. brevicollis *has been reported as a closer relatives to the hypothetical ancestor of all multicellular animals based on the comparative analyses of animal-specific genes [97,98]. Interestingly, the genome of *M. brevicollis *possesses p47*phox*- and p22*phox*-like genes (Figures 9A,B), but a BLAST searching in the current *M. brevicollis *genomic database failed to identify a dinstinct p67*phox *ortholog. Furthermore, Nox2-like gene is present in the *M. brevicollis *genome sequence (Figure 9A). Interestingly, a taxonomy of Nox domains demonstrates that *M. brevicollis *Nox2-like gene diverges from the monophyletic clade of all metazoan Nox2 genes containing other ancestral Nox2 and all vertebrate Nox1-3, but it differs from Nox4 and fungal NoxA/NoxB subfamilies (see Additional file 6). The *M. brevicollis *p47*phox*-like protein possesses AIR-like region that conserves a serine residue consistent with a consensus motif of a putative substrate of protein kinase C (Figure 9C); therefore, this implies that ROS-generating activity of the most primitive Nox2-like protein is also strictly regulated by phosphorylation as seen in mammalian phagocyte NADPH-oxidase. The physiological function of the primordial Nox2 enzyme in *M. brevicollis *is unknown, but the early appearance of the *Phox*-like Nox system in the unicellular flagellate suggests the presence of a fundamental and essential role of the regulatory subunit-dependent Nox in a unicellular living.

Figure 9A also summarizes the occurrence of Nox4, Nox5, and Duox isologs in genomes of the metazoa and the choanoflagellate, adding to information published previously [1]. Duox orthologs are present in all analyzed genomes of the Bilateria (Figure 9A). Nox5 genes occur broadly in the genomes of Metazoa, but not in Nematoda. Nox4 genes appear after the emergence of chordates, but is not seen in the echinoderm *S. purpuratus *[1] (Figure 1A). Interestingly, gene search that covers a wide range demonstrates that Nox4-like genes are also present in a lophotrochozoan (*L. gigantea*), a cnidarian (*N. vectensis*), but not in the ecdysozoans (see Figure 9A, and the taxonomy of Nox is shown in Additional file 6). The choanoflagellate *M. brevicolli *do not possess Nox4, Nox5, and Duox orthologs, at least based on the current genomic database. In the kingdom Plantae, the moss *Physcomitrella patens *(*P. patens*) possess double EF-hand- containing Noxes like the higher land plants *Arabidopsis thaliana *(*A. thaliana*) and *Oryza sativa *(*O. sativa*) (Figure 9A). Despite a kingdom distinct from plants, the oomycete *Phytophthora sojae *(*P. sojae*) also possesses single or double EF-hand(s)-containing Noxes [55,99], at least three genes (Figure 9A). Here we referred to them as *P. patens *and *P. sojae *rboh (respiratory burst oxidase homolog) to represent the remarkable resemblance to the higher plant Noxes, so-called rboh, in the respects of taxonomy of the Nox domains (Additional file 6). In the kingdoms Plantae and Chomista, a distinct regulatory subunit was not seen. However, higher plant genomes possess a novel protein family conserved domain structures similar to p67*phox *as descried below. *Naegleria gruberi *(*N. gruberi*) is a widespread soil and freshwater amoeboflagellate that belongs to the phylum Perocolozoa, the kingdom Protozoa [100]. Although *N. gruberi *is a free-living organism, it is not related to species of the phylum Amoebozoa with sequenced genomes, such as *D. discoideum *and *Entamoeba histolytica*, but it is close to pathogenic relatives (e.g. *Naegleria fowleri*) that can cause amoebic meningitis. We identified single Nox gene of *N. gruberi *from the genome (Figure 9A), together with five putative *Fre *ortholog genes that have similar domain structures to that of Nox domain (see Additional file 9). According to a phylogenetic taxonomy of Nox domain, the *N. gruberi *Nox is close to *D. discoideum *NoxA/NoxB proteins (see Additional file 6); therefore, it is referred to as *N. gruberi *NoxA (Figure 9A). Nox regulatory subunit gene has not been found in the current version of genomic database, but it might contain a prototype of regulatory subunits according to the Nox taxonomy.

### SH3PXD2 as a p47*phox *family member

**Figure 10:**
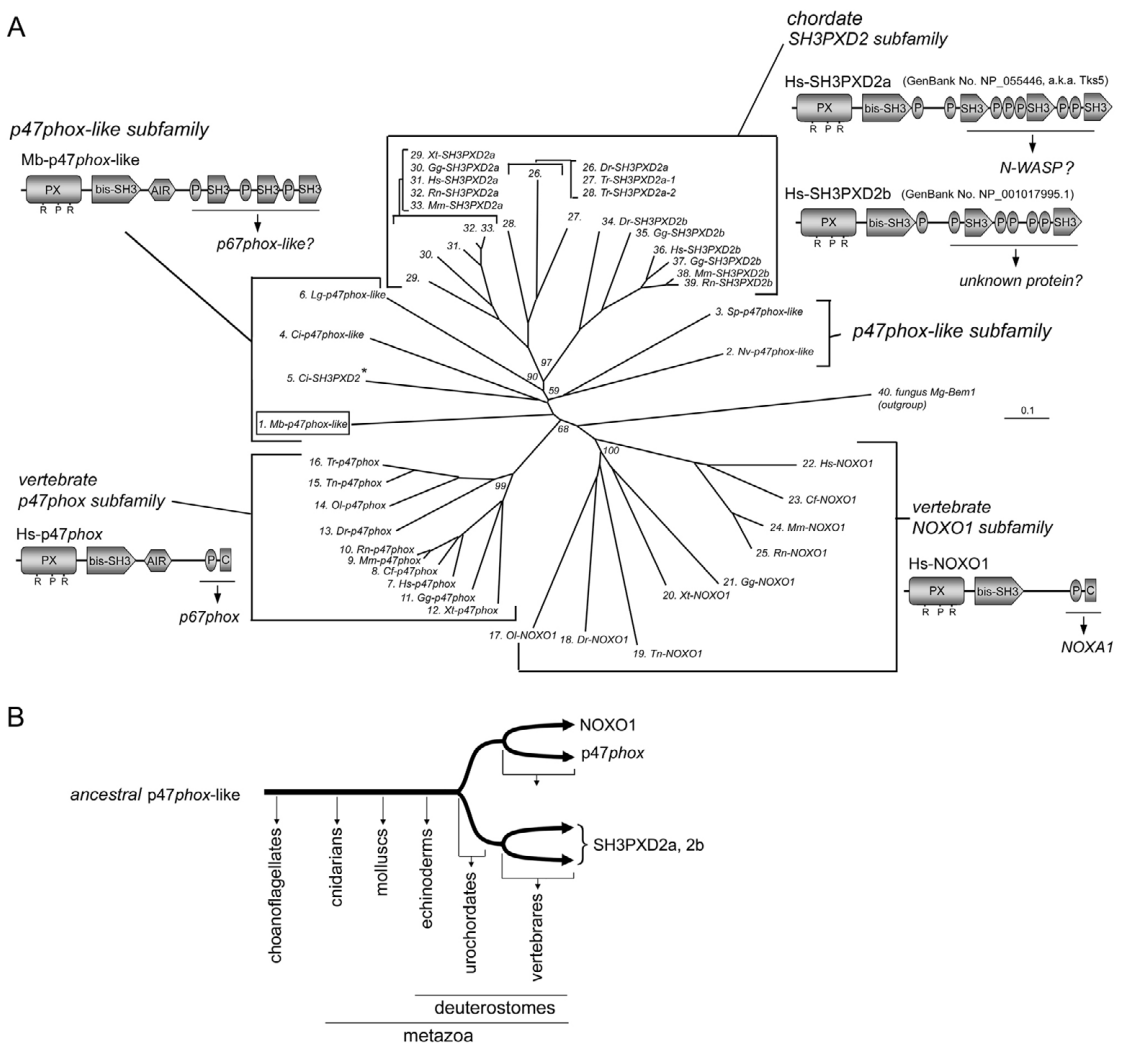
**Evolution of a vast family of p47*phox *proteins**. (A) Molecular taxonomy of the p47*phox *and related proteins is shown. The sequences of SH3PXD2 orthologs are provided in Additional file 10. Predicted domains structures of *H. sapiens *(p47*phox*, NOXO1, SH3PXD2a, 2b) and *M. brevicollis *(p47*phox*-like) proteins are shown near to each subfamily: P, proline-rich region; C, C-tail region. An *asterisk *indicates the appearance of a *C. intestinalis *SH3PXD2 branch from a common root of the p47*phox*-like protein. Abbreviations of species names and other domains are given in other Figures. (B) A schematic drawing to show a hypothetical evolution of an expanded p47*phox*-like gene family. Branches are for illustrative purposes and are not proportional to rates of divergence.

As shown in Figure 9, a choanoflagellate and primitive animals possess p47*phox*-like genes as well as ancestral Nox2 and PRR-containing p22*phox *genes. On the other hand, further BLASTP search in the animal genomes using the *M. brevicolli *p47*phox*-like protein sequence suggested the presence of a protein family proteins related to the primitive p47*phox *in higher animals, encoding the **"**SH3 and PX domains 2" protein (SH3PXD2) (sequences and alignments are provided in Additional files 10 and 11, respectively). The encoded protein is composed of an N-terminal PX domain and a central *bis*-SH3 domain, plus two or three additional SH3 domains in the C-terminal half of the molecule (see the motif structure in Figure 10A). The SH3PXD2 gene is present in a urochordate, but was not seen in an echinoderm and other primitive metazoan species (Figures 10A and 10B). In vertebrates, two isozymes SH3PXD2a and 2b were found (Figure 10A). Interestingly the *M. brevicollis *p47*phox*-like gene and other primitive animal p47*phox*-like genes are related more closely to the SH3PXD2 gene subfamily than to the vertebrate p47*phox *and NOXO1 subfamilies (Figure 10A). The physiological function of SH3PXD2a is suggested by only a few papers; human SH3PXD2a protein (a.k.a. Tks5) is involved in the protease-driven invasion of cancer cells and the neurotoxicity induced by amyloid-beta [101,102]. A protein binding to SH3PXD2a is also little known, but recently N-WASP (Wiskott-Aldrich Syndrome protein) was proposed as a candidate that binds to the third SH3 domain of the human SH3PXD2a [101]. The physiological role as well as the binding partner of SH3PXD2b is unknown. However, the clear phylogenetic relationship of the SH3PXD2 gene family to the larger family of Nox-regulatory organizing proteins suggests that the genes might be involved in regulating Nox activity. Alternatively, SH3PXD2 proteins may have diverged to perform unrelated functions (e.g., to regulate other enzymes or signaling pathways). Thus, the *M. brevilcollis *p47*phox*-like protein evolved from a hypothetical p47*phox *ancestor, and the SH3PXD2 genes diverged from the lineage of the p47*phox*-like gene following the emergence of chordates but prior to NOXO1 (Figure 10B). The vertebrate p47*phox *and NOXO1 were somehow able to eliminate the additional SH3 domains in the "regulator-docking region", as proposed in Figure 8.

### What is the regulatory subunit binding partner for fungal NOXR?

**Figure 11:**
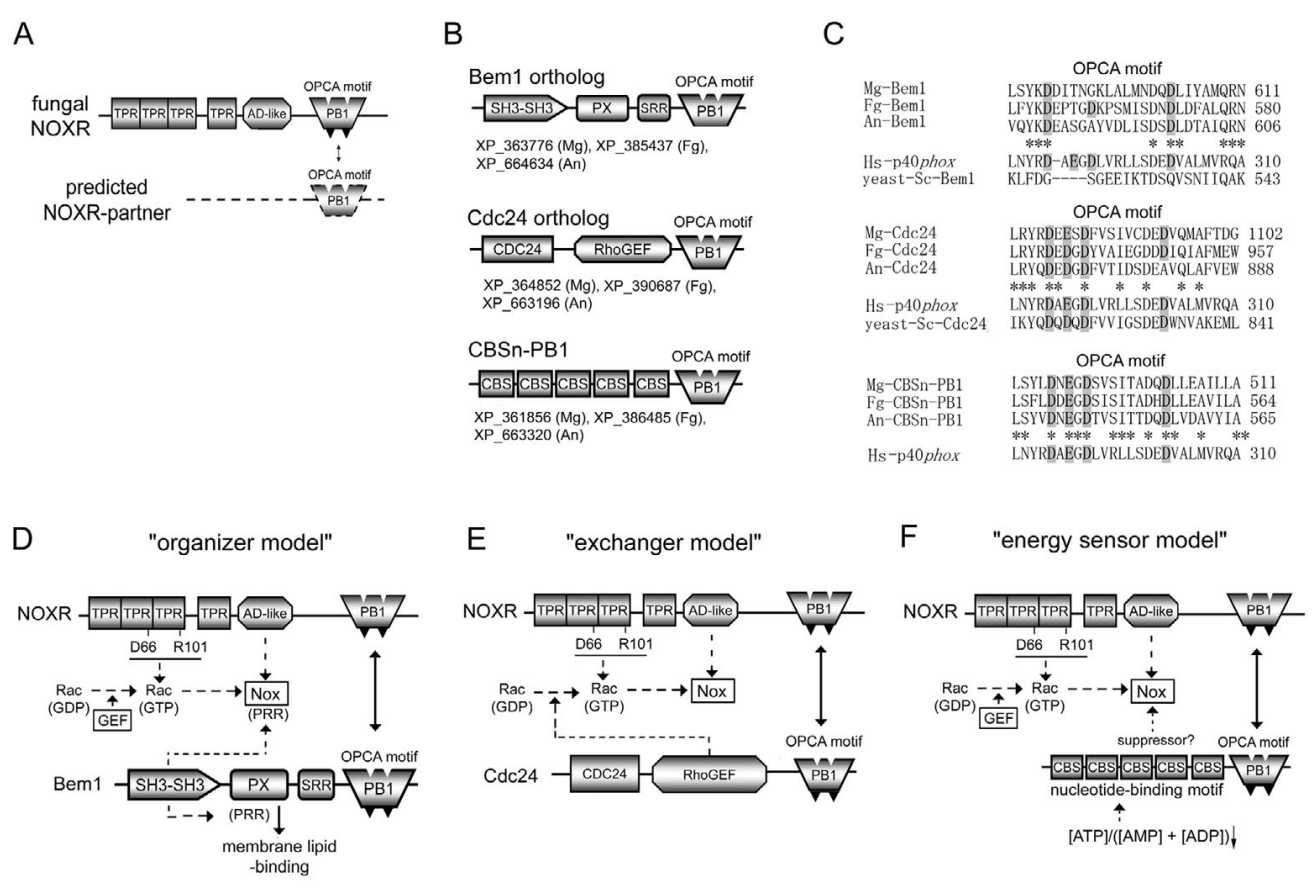
**Predicted binding partners of fungal NOXR and activation models of fungal Nox**. (*A*) A hypothetical domain structure of a predicted NOXR partner is shown. (*B*) Domain structures of fungal proteins with PB1 domains meeting criteria for possible NOXR-partners are shown, along with their GenBank™ accession numbers. Abbreviations: An, *A. nidulans*; Mg, *M. grisea*; Fg, *F. graminearum*. (*C*) Aligned OPCA motifs of candidate NOXR partners are compared with the corresponding region in p40*phox *and the yeast isolog (the first and second alignments). Amino acid residues conserved among fungal ortholog sequences are indicated by *asterisks*. (*D*) A proposed model for activation of fungal Nox by NOXR and Bem1 protein. (*E*) A proposed model for activation of fungal Nox by NOXR and Cdc24. (*F*) A proposed model for activation of fungal Nox by CBSn-PB1 proteins. RhoGEF, Rho guanine nucleotide exchange factor; CBS, cystathionine beta-synthase; Abbreviations of other domain names are described in legends to other Figures.

Fungi (*A. nidulans*, *M. grisea*, and *F. graminearum*) belong to Ascomycota [55] and possess the NOXR gene as a p67*phox *ortholog (Figure 9A). NOXR encodes a PB1 domain homologous to that in p67*phox*, but these organisms do not possess a p40*phox *ortholog (see Figure 1A). As shown in Figure 5E, two important basic residues in the PB1 domains are conserved in all NOXR proteins. The conservation of these residues implies the existence of an unknown NOXR binding partner(s) containing a PB1 domain with an OPCA motif (Figure 11A). A BLAST search of a database of computationally predicted amino acid sequences [103] from the genomes of *A. nidulans*, *M. grisea*, and *F. graminearum *was performed using as a query sequence the PB1 domain of human p40*phox *(residues 237–339; GenBank™ No. NP_000622). In addition to NOXR, three sequences harboring PB1 domains were identified in the fungi. These were: "SH3-SH3-PX-SRR-PB1", "CDC24-RhoGEF-PB1" and "(CBS)n-PB1" proteins (Figure 11B and see Additional file 12), so-named for their domain structures in sequence from N- to C-termini. SRR indicates a serine-rich region. The three predicted proteins were encoded in all three fungal genomes, and all three possessed acidic OPCA motifs in their PB1 domains (Figure 11C).

The "SH3-SH3-PX-SRR-PB1" protein contains a similar domain structure to yeast *S. cerevisiae *Bem1p (Bud Emergence 1, GenBank™ accession number NP_009759), but differs in that the PB1 domain of *S. cerevisiae *Bem1p does not contain the OPCA motif [76]. A speculative model for the regulation of fungal Nox by the Bem1 ortholog is shown in Figure 11D, wherein Bem1 ortholog is proposed to play a role as an organizer protein, analogous to the roles of p47*phox *and p40*phox *in mammals. According to this model, PB1-PB1 interaction mediates the linkage between the "regulator-docking regions" suggested in Figure 8. PX-domain of the *S. cerevisiae *Bem1 facilitates a protein to anchor to a membrane lipid layer [104]. Fungi do not have a PRR-containing p22*phox *ortholog (see Figure 1A), but NoxA and NoxB in the three fungi has a distinctive highly conserved a class II PRR motif [91] (e.g. 423–428 residues of Mg-NoxA) and class III (e.g. 447–452 residues of Mg-NoxB) near the predicted NADPH-binding sub-regions [1]. Thus the hypothetical functions of the *bis-*SH3 and PX domains indicate the analogous function as a "membrane-binding/cytochrome docking region" of Nox organizer. Because *S. cerevisiae *Bem1 is involved in establishment of cell polarity [105], the proposed interaction between fungal NOXR and Bem1 orthologs may point to a function of Nox-derived ROS in cell polarity.

A second model is suggested by the "CDC24-RhoGEF-PB1" protein, which is an ortholog of *S. cerevisiae *Cdc24p (GenBank™ accession number AAA82871). According to this "exchanger model", the PB1-mediated heterodimer formation localizes the RhoGEF domain which then catalyzes the conversion of Rac-GDP to the active Rac-GTP (Figure 11E). The model is supported by a recent report showed that fungal NOXR binds to the fungal Rac homologue in the GTP form [57].

An "energy sensor" function for NOXR is shown in Figure 11F, and is based on the occurrence of the CBS (cystathione beta-synthase) domain in the putative binding partner. The CBS domain senses cellular energy status by binding adenosine nucleotides; it occurs in a range of proteins including AMP-activated protein kinase, which is activated by AMP and inhibited by ATP via the CBS domain [106,107]. Such a function might be related to the requirement of Nox enzyme for reduced pyridine nucleotide, thus affecting cellular energetics.

**Figure 12:**
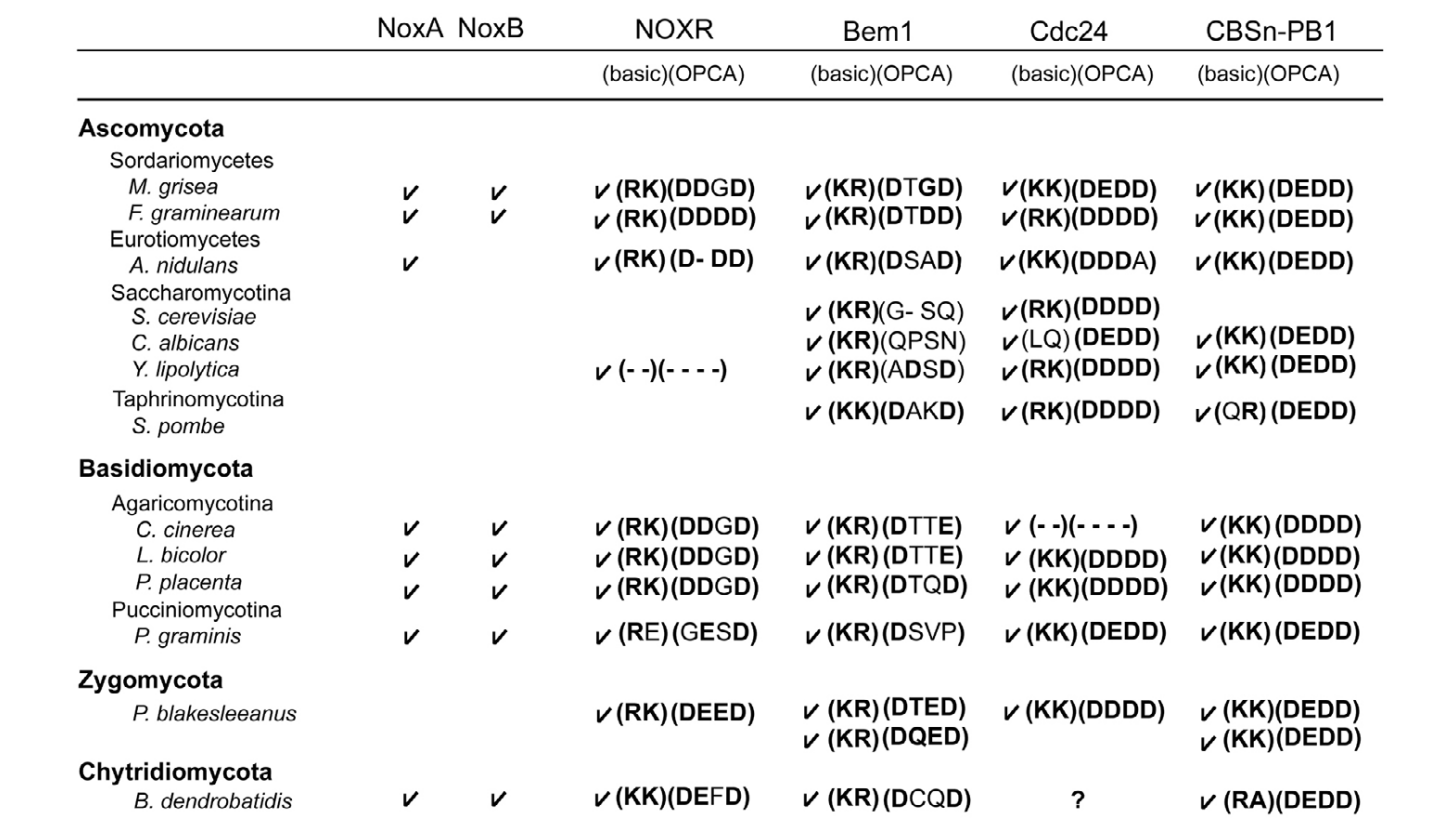
**Conservations of NOXR, Bem1, Cdc24, and CBSn-PB1 genes and characteristic residues of PB1 domains in fungal genomes**. (*A*) "basic" and "OPCA" in parenthesis indicate key residues that are required to form a dimer between two PB1 domains; two "basic" residues and four in "OPCA" motif residues correspond to Lys-355 and Lys-382 of human p67*phox *and Asp-289, Glu-291, Asp-293 and Asp-302 of human p40*phox*, respectively. Divisions of Fungi are shown in *bold*.

Fungal Nox and NOXR isologs are seen not only in fungi belonging to Ascomycota, but also some basidiomycetes [55]. To explore the likelihood of the fungal Nox activation mechanisms suggested in Figure 11, the occurrence of NOXR, Bem1, Cdc24, and CBSn-PB1 isologs were compared in 13 fungal genomes spanning the four fungal divisions: Ascomycota, Basidiomycota, Zygomycota, and Chytridiomycota (sequences are shown in Additional file 12). Bem1, Cdc24, and CBSn-PB1 genes are present in most of the fungal genomes, with the exception of Cdc24 in *Batrachochytrium dendrobatidis *(*B. dendrobatidis*), and these proteins all conserved the PB1 domains with some exceptions; *Y. lipolytica *NOXR and *Coprinopsis cinerea *Cdc24 isologs lack the PB1 domain (Figure 12). In addition to the absence of Nox and NOXR isolog genes in *S. cerevisiae*, the Nox and NOXR isologs are absent in the genomes of *Candida albicans, Yarrowia lipolytica *(*Y. lipolytica*) and *Schizosaccharomyces pombe *(*S. pombe*) [55]. A Nox gene is absent in the genomes of *Y. lipolytica *and *Phycomyces blakesleeanus *(*P. blakesleeanus*) despite the presence of NOXR isologs [55]. The *P. blakesleeanus *genome possesses two Bem1 and CBS-PB1 genes and all conserved the PB1 domains. We investigated the conservation of two basic residues of the PB1 domain corresponding to the Lys-355 and -382 residues of human p67*phox *and the four acidic residues in OPCA motif corresponding to the Asp-289, Glu-291, and Asp-293, -302 residues of human p40*phox*. Comparison of the PB1 domains in these fungal proteins by alignment and analyses by the Pfam program demonstrate that the two basic and four acidic residues are conserved in the fungal NOXR, Bem1, Cdc24, and CBSn-PB1 with a few exceptions; the OPCA motifs in Bem1 isologs of *S. cerevisiae *and *C. albicans *has been lost (Figure 12 and the sequences and detailed alignments are shown in Additional file 13 and 14, respectively). The high conservation of the motif strongly implies that these PB1 domains mediate functionally important oligomerizations. Future functional studies will clarify the roles of these proteins in fungal Nox functions.

### Predicted binding partners for *D. discoideum *p67-like protein

**Figure 13:**
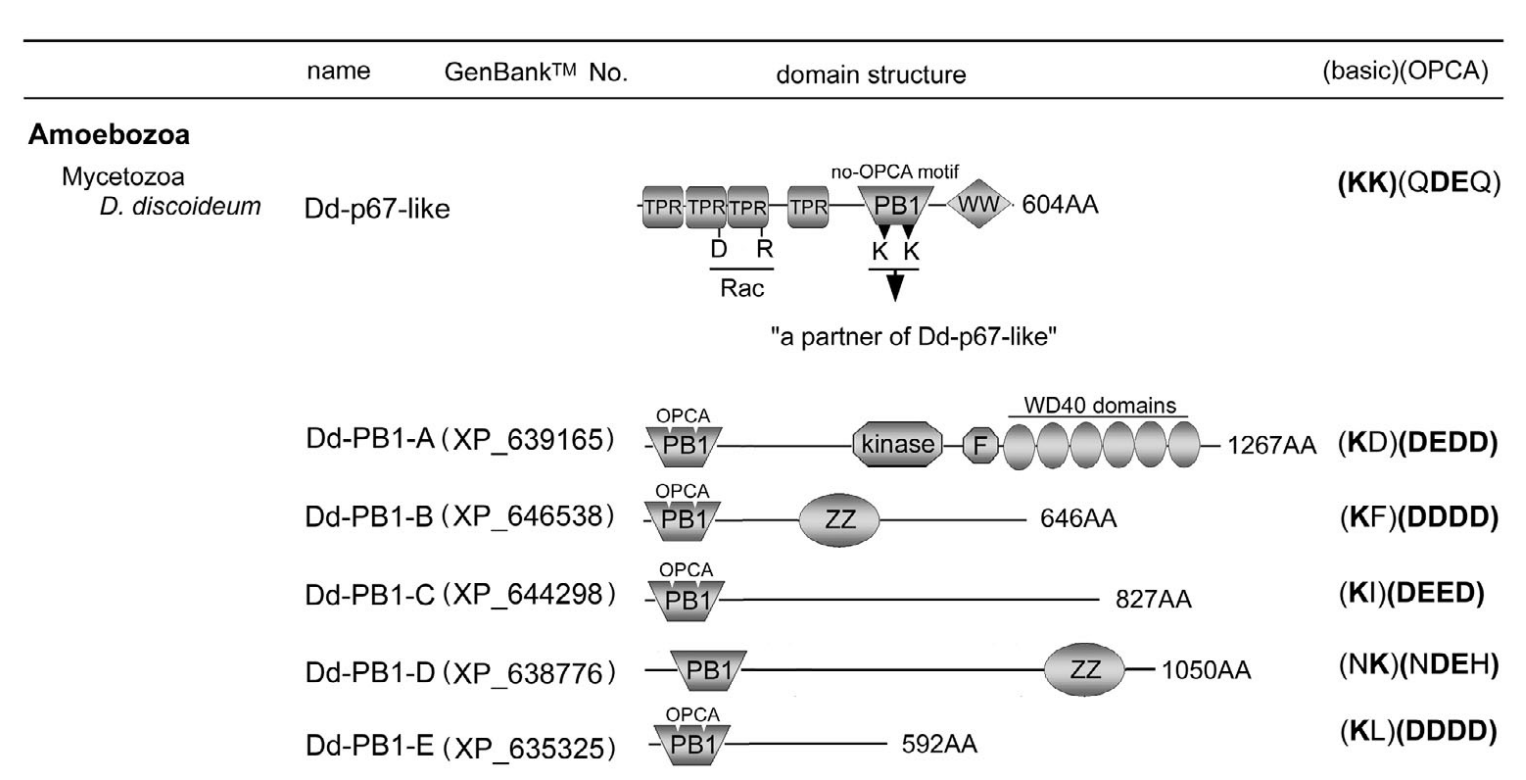
**Predicted candidate binding partners of *D. discoideum *p67-like protein**. Predicted domain structures of Dd-p67-like protein and candidates of binding partners are shown. "basic" and "OPCA" in *parenthesis *indicate six characteristic residues of PB1 domain as shown in Figure 12. Abbreviations: kinase, protein serine/threonine kinase domain; F, F-box; WD40, WD40 repeat; WW, WW domain; ZZ, ZZ type zinc-finger.

Like fungal NOXRs, the PB1 domain of *D. discoideum *p67-like protein also possesses two basic residues, Lys-313 and Lys-340 residues, as shown in Figure 5E. A BLAST search demonstrates that the *D. discoideum *genome possesses five predicted PB1 domain-containing proteins (referred to here as "Dd-PB1-A to -E"). All except Dd-PB1-D conserve the OPCA motif (Figure 13, and see details in Additional file 15). Dd-PB1-A protein (GenBank No. XP_639165) is partially identical to *D. discoideum *MEK kinase α (GenBank No. AAC97114) [108]. It is possible that the serine/threonine kinase domain might phosphorylate the amoebal Noxes or the p67-like protein to stimulate ROS generation, analogous to other regulatory phosphorylations of Nox and their regulatory subunits [20,109-111]. Another interesting point of the Dd-PB1-A protein is to contain the F-box and a WD40 repeat regions that function as a ubiquitin ligase component E3. Further investigations to identify a binding partner will lead to understand functional relationships between Nox and the Dd-PB1 proteins.

### Do p67*phox*-like genes exist in plants?

The land plants, *A. thaliana *and *O. sativa *possess the multiple EF-hand-containing Nox proteins, At-rboh-A to -J [1,112,113] and Os-rboh-A [114] to -H, respectively. However, there is no report of *Phox*-related regulatory subunit homologs in plant genomes. Unexpectedly BLAST search using PB1 domain sequences demonstrates that *A. thaliana *possesses a unique family of genes encoding four proteins that possess both TPR and PB1 domains (GenBank accession No. NP_194935, NP_180101, NP_197536, NP_564794). The *O. sativa *genome also possesses a similar gene (GenBank accession No. BAD31284). Herein, we refer to this family as "hypothetical p67*phox*-like (hypo-p67-L)". The expressions of at least the two identified genes, At-hypo-p67-L1 and -L4, were confirmed according to the EST database [115].

**Figure 14:**
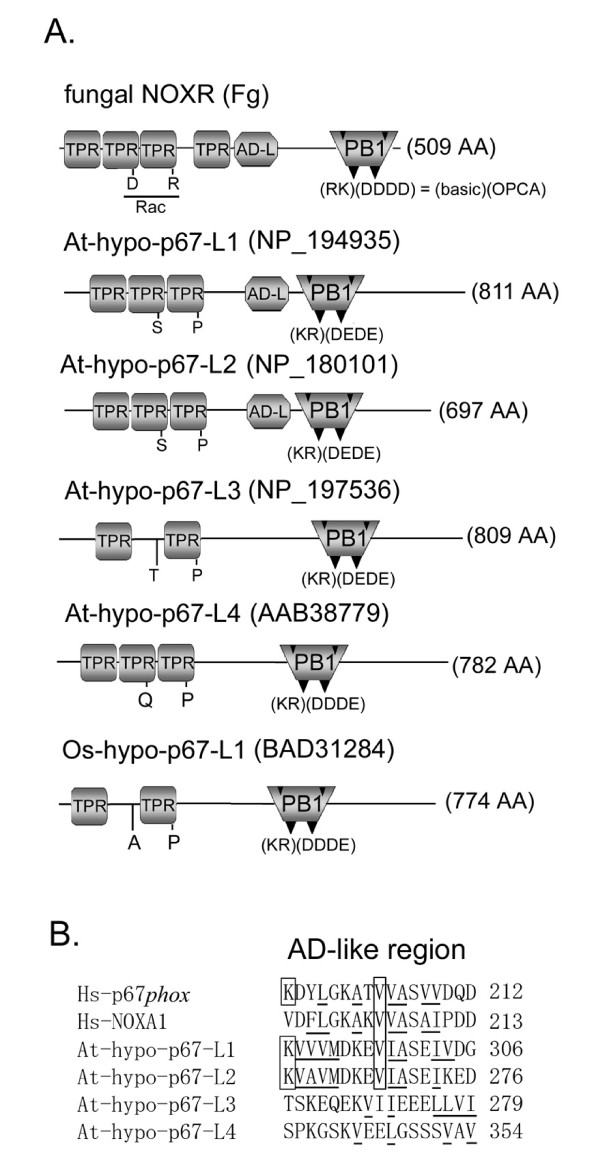
**Predicted p67*phox- *like proteins in higher plants**. (A) Predicted domain structures of hypothetical p67*phox*-like proteins (At-hypo-p67-L1 to -L4, Os-hypo-p67-L1) of *A. thaliana *and *O. sativa*, and a fungal *F. graminearum *(Fg-) NOXR are shown, along with their GenBank™ accession numbers. "basic" and "OPCA" in parenthesis indicate six characteristic residues of PB1 domains as shown in Figure 12. "D" and "R" below the TPR region of Fg-NOXR represent two conserved amino acid residues that are important for binding to Rac as indicated in Figure 5. (B) Alignments of the putative AD-like regions are shown (see details in Additional file 17). *Boxes *indicate lysine and valine residues corresponding to the Lys-196 and Val-204 of human p67*phox*, respectively. *Underlines *in alignment indicates amino acid with a hydropholic side group.

Domain structures of the *A. thaliana *and *O. sativa *hypo-p67-L proteins are shown in Figure 14A (sequences are provided in Additional file 16). The six residues characteristic of the PB1 domains are conserved in all sequences (Figure 14A, and see the alignment in Additional file 17). This predicts that the hypo-p67-L proteins will form a heteromeric or homomeric complex via PB1-PB1-interaction. In addition to the hypo-p67-L gene family, the *A. thaliana *genome possessed at least 27 isolated genes encoding the PB1 domain [e.g. protein kinase family protein (GenBank No. NP_200569), CBSn-PB1 ortholog (GenBank No. NP_181191), see Additional file 18]. Alignment of putative AD-like domains of the *A. thaliana *proteins with human p67*phox *and NOXA1 show that the *A. thaliana *hypo-p67-L1 and -L2 proteins conserve the functionally critical valine residue (Figure 14B). Moreover, a lysine residue corresponding to Lys-196 of human p67*phox *that is correlated to the appearance of functional PB1 domain is also conserved in two of them (Figure 14B). On the other hand, the sequences do not conserve residues in the TPR domain known to be essential for binding to Rac [10]. Biological functions for this putative family are unknown and further investigations are needed.

## Conclusion

In summary, we reported here an exhaustive analysis of the *Phox*-related regulatory subunits for Nox enzymes from a total of 32 species in the Eukaryota including 10 vertebrates. The present study, viewed in the context of the evolution of Noxes [1], reveals that regulatory subunits co-evolved with their respective Noxes; Nox2 with p67*phox*/p47*phox*; Nox1 with NOXO1/NOXA1; ancestral Nox2 as co-ortholog of vertebrate Nox1-3 with p67*phox*/p47*phox*-like proteins; fungal and amoebal NoxA/B with NOXR (and possibly new components). By comparing amino acid sequences of the regulatory subunits, putative key amino acid residues and conserved region previously undefined were identified, e.g. ADSIS. These conserved residues are likely to be important in conserved catalytic or regulatory functions of Nox proteins, although, the detailed biochemical roles of most of these residues are not yet fully understood. The analysis of the *Phox*-related regulatory subunits points to a novel evolutionary history of Nox regulatory proteins. The earliest Nox2 and p47*phox*- and p22*phox*-like genes are appearing in the unicellular choanoflagellate before the emergence of the metazoa. Duplication of primordial p47*phox*- and p67*phox*-like genes occurred in vertebrates, resulting in the emergence of NOXO1-p47*phox *and NOXA1-p67*phox *genes, respectively. Supporting this evolutionary origin, the NOXO1 protein in the fish *O. latipes *still retains the p47*phox*-characteristic "AIR". A predicted motif structure of ancestral p47*phox *suggests a greatly expanded family of p47*phox *including SH3PXD2 in the animal lineages. Detailed sequence analyses proposed an evolution model that p40*phox *originally participated in regulating both Nox1 and Nox2 in early vertebrates, but in mammals the regulation of Nox1 became independent of p40*phox*. Thus, this report provides keys to understand the evolutionary history of Nox and the regulatory subunits, and provides new information that may be useful in elucidating new molecular details of the activation mechanisms of Nox enzymes including the human orthologs.

Note: While we were in the process of revising this manuscript, a review article about structure and function of the PB1 domain appeared [116]. The review will help to understand the structural features of the PB1 domain that are often mentioned in the present study. In the article, authors also described the presence of the hypothetical genes coding TPR and PB1 domain-containing proteins in the *A. thaliana *genomes and argued about a possibility of *A. thaliana *p67*phox*.

## Methods

### Gene identification

Homologues/orthologs of Nox/Duox and regulatory subunits sequences were assembled from the following species: *H. sapiens*, (human), *C. familliaris *(dog), *R. norvegicus *(rat), *M. musculus *(mouse), *G. gallus *(chicken), *X. tropicalis *(frog), *D. rerio *(zebrafish), *T. rubripes *(fugu), *T. nigroviridis *(tetraodon), *O. latipes *(medaka), *C. intestinalis *(ascidian), *S. purpuratus *(sea urchin), *D. pulex *(waterflea), C.*briggsae *(nematode), *C. elegans *(nematode), *N. vectensis *(sea anemone), *L. gigantea *(gastropod snail), *M. brevicollis *(choanoflagellate), *D. discoideum *(slime mold amoeba), *C. merolae *(red alga), *A. nidulans *(fungus-An), *M. grisea *(fungus-Mg), *F. graminearum *(fungus-Fg), *S. cerevisiae *(fungus-Sc), *C. albicans *(fungus-Ca), *Y. lipolytica *(fungus-Yl), *S. pombe *(fungus-Sp), *Coprinopsis cinerea *(*C. cinerea*, fungus-Cc), *L. bicolor *(fungus-Lb), *P. placenta *(fungus-Pp), *Puccinia graminis *(*P. graminis*, fungus-Pg), *P. blakesleeanus *(fungus-Pb), *B. dendrobatidis *(fungus-Bd), *P. sojae *(oomycete), *Phaeodactylum tricornutum *(*P. tricornutum*, diatom), *Physcomitrella patens *(*P. patens *moss), *A. thaliana *(land plant), and *O. sativa *(land plant). Using NCBI-HomoloGene [117], existing homologues/orthologs of regulatory subunits were assembled: p47*phox*, NOXO1, p67*phox*, NOXA1, p40*phox *and p22*phox *orthologs of human, dog, mouse, rat, and p47*phox *and p67*phox *and NOXO1 orthologs of chicken. In addition, we assembled amino acid sequences that have been cloned and reported: p47*phox*, p67*phox*, and p22*phox *of *T. rubripes *[89] and *C. intestinalis *[61]; p67-like protein of *D. discoideum *[56]; NOXR of fungi including *A. nidulans, M. grisea*, *F. graminearum *[55,57]. Based on these sequences, BLASTP searches were performed to obtain predicted regulatory subunits, related Nox genes (including genes that has been reported such as *P. sojae *rboh [99], *C. merolae *rboh1/2 [118], *P. tricornutum *rboh1 [118]), new regulator-binging partners, using the NCBI protein database for vertebrates, *C. intestinalis, C. briggsae, D. discoideum, A. thaliana*, *O. sativa *[103], *S. purpuratus *[63], and for fungi (*A. nidulans*, *M. grisea*, *F. graminearum*, *S. cerevisiae, C. albicans, Y. lipolytica, S. pombe, C. cinerea*) [119], the Doe Joint Genome Institute database for *L. bicolor, P. placenta*, *D. pulex*, *N. vectensis*, *L. gigantea*, *M. brevicollis, P. blakesleeanus, P. sojae, P. tricornutum*, *P. patens *[120], and the Broad Institute FGI database for *P. graminis *and *B. dendrobatidis *[121]. Sequences that had more than 50% identity to the sequence of the closest template were chosen as orthologs or isologs. Assembled sequences, including newly defined, were annotated based on molecular taxonomy analysis of the domains characteristic in each regulator. Characteristic motifs of the regulatory subunits are predicted by the Pfam program [122]. All amino acid sequences and the accession numbers of assembled and analyzed regulator orthologs are listed in Additional material (see Additional files 1, 5, 7, 9, 10, 12, 13, 15, 16, 18).

### Phylogenic analysis and synteny

Multiple sequence alignment and phylogenetic analyses were carried out with ClustalW [123] and trees were reconstructed by the neighbor-joining method as previously described [1]. Amino acid sequences of regulatory subunits were trimmed and aligned to the domains indicated in each figure. Bootstrap values of 1,000 replications are shown at the branches as percentages. Equivalent to 0.1 amino acid substitutions per site indicate evolutionary distances as inferior bars. To elucidate the synteny, we used the Ensembl AlignSliceView program [124]. Conserved marker genes among the genomes of vertebrates are defined (see Additional file 2).

## Abbreviations

Nox, NADPH oxidase; Duox, dual oxidase; ROS, reactive oxygen species; TM, transmembrane; NOXO1, Nox Organizer 1; NOXA1, Nox Activator 1: TPR, the tetratricopeptide repeats; AD, activation domain; SH3, Src homology 3; PX, *Phox *homology domain; PRR, proline-rich region; P-basic, proline-basic region; PB1, *Phox*/Bem 1; OPCA motif, OPR/PC/AID motif; ADSIS, AD-SH3 Intervening Sequence; SH3PXD2, SH3 and PX domains 2; SRR, serine-rich region; RhoGEF, Rho guanine nucleotide exchange factor; CBS, cystathionine beta-synthase.

## Competing interests

The author(s) declares that there are no competing interests.

## Authors' contributions

The majority of the work here described was carried out by TK. JDL critically revised the manuscript for important intellectual content and data analysis. Both authors read and approved the final manuscript.

## Supplementary Material

Additional file 1 **Amino acid sequences of Nox regulatory subunits of 12 dueterostomes, three fungi, and a slime mold amoeba; p47*phox*, NOXO1, p67*phox*, NOXA1, NOXR, p40*phox*, p22*phox***. Amino acid sequences Nox and Duox proteins of *H. sapiens*, *C. familliaris*, *R. norvegicus*, *M. musculus*, *G. gallus*, *X. tropicalis*, *D. rerio*, *T. rubripes*, *T. nigroviridis*, *O. latipes, C. intestinalis*, *S. purpuratus*, *A. nidulans*, *M. grisea*, *F. graminearum *and *D. discoideum *are provided. Click here for file

Additional file 2 **Abbreviations of gene names in Figure **2. Names of the marker genes used in Figures 2 are provided. Click here for file

Additional file 3 **Alignment of characterized domains of Nox regulatory subunits**. Alignments of (i) PX and tandem SH3 domains of Nox organizers of deuterostomes, (ii) TPR and activation domains of Nox activators of deuterostomes and fungi (*A. nidulans*, *M. grisea*, *F. graminearum*) and amoeba, (iii) the N-terminal SH3 domain of vertebrate p67*phox*, (iv) PB1 domain of p67*phox *and NOXA1 of vertebrates and NOXR of fungi (*A. nidulans*, *M. grisea*, *F. graminearum*) and amoeba, (v) the C-terminal SH3 domain of vertebrate p67*phox*, vertebrate NOXA1 and *S. purpuratus *p67*phox*-like protein, (vi) PX domain of vertebrate p40*phox*, (vii) Proline (P)-basic, SH3 and PB1 domain of vertebrate p40*phox*. Click here for file

Additional file 4 **Alignment of full-length p22*phox *(vertebrate p22*phox *and *C. intestinalis *p22*phox*-like protein)**. Alignments of p22*phox *of chordates. Click here for file

Additional file 5 **Amino acid sequences of Nox/Duox proteins**. Amino acid sequences Nox and Duox proteins of the waterflea *D. pulex*, the gastropod snail-limpet *L. gigantea*, the nematode *C. briggsae*, the sea anemone *N. vectensis*, the choanoflagellate *M. brevicollis*, the fungi (*C. cinerea*, *L. bicolor*, *P. placenta*, *P. graminis*, *P. blackesleeanus*, and *B. dendrobatidis*), the plant *O. sativa*, and the red alga *C. merolae *are provided. Click here for file

Additional file 6 **Molecular taxonomy of Nox and Duox proteins containing newly identified sequences**. molecular taxonomy built by amino acid sequences of Nox and Duox proteins of *D. pulex*, *L. gigantea*, C.*briggsae*, *N. vectensis*, *M. brevicollis, C. intestinalis*, *S. purpuratus*, and *H. sapiens *is shown. Click here for file

Additional file 7 **Amino acid sequences of putative Nox regulatory subunits of *L. gigantea*, *N. vectensis, M. brevicollis***. Amino acid sequences of putative regulatory subunit proteins of *L. gigantea *(p47*phox-*, p67*phox-*, p22*phox*-like proteins), *N. vectensis *(p47*phox-*, p22*phox*-like proteins), and *M. brevicollis *(p47*phox-*, p22*phox*-like proteins) are provided. Click here for file

Additional file 8 **Alignments of putative regulatory subunit proteins of *L. gigantea*(p47*phox-*, p67*phox-*, p22*phox*-like proteins), *N. vectensis *(p47*phox-*, p22*phox*-like proteins), and *M. brevicollis *(p47*phox-*, p22*phox*-like proteins)**. Alignment of p67*phox*-like protein (*L. gigantea *and *H. sapiens*), p47*phox*-like protein (*L. gigantea, N. vectensis*, *M. brevicollis, S. purpuratus*, and *H. sapiens*), p22*phox*-like proteins (*L. gigantea, N. vectensis*, *M. brevicollis*, and *H. sapiens*) are provided. Click here for file

Additional file 9 **Amino acid sequences of Fre of an amoeboflagellate**. Amino acid sequences of predicted Fre proteins of the amoeboflagellate *Naegleria gruberi *are provided. Click here for file

Additional file 10 **Amino acid sequences of SH3PXD2 proteins**. Amino acid sequences vertebrate and urochordate SH3 and PX domains 2 (SH3PXD2) proteins are provided. Alignments of PX and *bis*-SH3 domains of SH3PXD2, p47*phox*, NOXO1, p47*phox*-like proteins are shown. Click here for file

Additional file 11 **Alignment of vertebrate p47*phox*, vertebrate NOXO1, p47*phox*-like protein, and chordate SH3PXD2 proteins**. Alignments of SH3PXD2a and 2b of *H. sapiens*, *C. familliaris*, *R. norvegicus*, *M. musculus*, *G. gallus*, *X. tropicalis*, *D. rerio*, *T. rubripes*, *T. nigroviridis*, *O. latipes*, and *C. intestinalis *with other p47*phox *family proteins are shown. Click here for file

Additional file 12 **Amino acid sequences and alignment of predicted fungal NOXR partner: Bem1, Cdc24, CBSn-PB1**. Amino acid sequences Bem1, Cdc24, CBSn-PB1 of *A. nidulans*, *M. grisea *and *F. graminearum *are provided. Alignments of each gene family are shown. Click here for file

Additional file 13 **Amino acid sequences of NOXR, Bem1, Cdc24, and CBSn-PB1 proteins of other fungi than *M. grisea*, *F. graminearum*, and *A. nidulans***. Amino acid sequences Bem1, Cdc24, and CBSn-PB1 proteins of *S. cerevisiae*, *C. albicans*, *Y. lipolytica*, *S. pombe*, *C. cinerea*, *L. bicolor*, *P. placenta*, *P. graminis*, *P. blakesleeanus*, and *B. dendrobatidis *are provided. Click here for file

Additional file 14 **Alignments of fungal NOXR, Bem1, Cdc24, and CBSn-PB1 amino acid sequences**. Alignments of fungal NOXR, Bem1, Cdc24, and CBSn-PB1 of *A. nidulans*, *M. grisea, F. graminearum S. cerevisiae*, *C. albicans*, *Y. lipolytica*, *S. pombe*, *C. cinerea*, *L. bicolor*, *P. placenta*, *P. graminis*, *P. blakesleeanus*, and *B. dendrobatidis *are provided. Click here for file

Additional file 15 **Amino acid sequences of PB1 domain-containing proteins of *D. discoideum *and alignment of PB1 domains**. Amino acid sequences of PB1 domain-containing proteins of the slime mold amoeba *D. discoideum *(Dd-PB1-A to -E) and an alignment of the PB1 domians are provided. Click here for file

Additional file 16 **Amino acid sequences of hypothetical p67*phox*-like (hypo-p67-L) of *Arabidopsis thaliana*and *Oryzae sativa***. Sequences of *A. thaliana *and *O. sativa *proteins that contain the TPR and PB1 domains (At-hypo-p67-L1 to L4 and Or-hypo-p67-L1) are shown. Click here for file

Additional file 17 **Alignment of hypothetical p67*phox*-like (hypo-p67-L) of *Arabidopsis thaliana *and *Oryza sativa***. (i) Alignment of four hypo-p67-L1 to -L4 of *A. thaliana*, (ii) comparison of TPR and AD domain between the *A. thaliana *and *O. sativa *proteins (At-hypo-p67-L1-4 and Os-hypo-p67-L1) and human p67*phox *(1–212 residues) and NOXA1 (1–213 residues) proteins are shown. Click here for file

Additional file 18 **ID numbers of hypothetical PB1 domain-containing genes of *Arabidopsis thaliana*,except for hypo-p67-L genes**. a total of 27 ID numbers of *Arabidopsis thaliana *genes are shown. Click here for file
