# Supplementary material for: Molecular evolution of Phox-related regulatory subunits for NADPH oxidase enzymes
Source: BMC Evol Biol. 2007 Sep 27;7:178. doi: 10.1186/1471-2148-7-178 (PMC2121648; doi:10.1186/1471-2148-7-178)
Supplement: Additional file 1 — Amino acid sequences of Nox regulatory subunits of 12 dueterostomes, three fungi, and a slime mold amoeba; p47phox, NOXO1, p67phox, NOXA1, NOXR, p40phox, p22phox. Amino acid sequences Nox and Duox proteins of H. sapiens, C. familliaris, R. norvegicus, M. musculus, G. gallus, X. tropicalis, D. rerio, T. rubripes, T. nigroviridis, O. latipes, C. intestinalis, S. purpuratus, A. nidulans, M. grisea, F. graminearum and D. discoideum are provided. [file 1471-2148-7-178-S1.doc]

**Additional File 1**

**Amino acid sequences of Nox regulatory subunits of 12 dueterostomes, three fungi, and a slime mold amoeba: p47*phox*, NOXO1, p67*phox*, NOXA1, NOXR, p40*phox*, p22*phox.*** To describe species of sequences, we used the following naming: human-Hs (as *H. sapiens*), dog-Cf (*C. familliaris*), rat-Rn (*R. norvegicus*), mouse-Mm (*M. musculus*), chicken-Gg (*G. gallus*), frog-Xt (*X. tropicalis*), zebrafish-Dr (*D. rerio*), fugu-Tr (*T. rubripes*), tetraodon-Tn (*T. nigroviridis*), medaka-Ol (*O. latipes*)*,* ascidian-Ci (*C. intestinalis*), sea urchin-Sp (*S. purpuratus*), amoeba-Dd (*D. discoideum*), fungus-An (*A. nidulans*), fungus-Mg (*M. grisea*) and fungus-Fg (*F. graminearum*). Sequences are available from the indicated servers: http://www.ncbi.nlm.nih.gov/ (GenBank^TM^ numbering genes), http://www.ensembl.org (Ensembl numbering genes) or http://www.ddbj.nig.ac.jp/searches-e.html (DDBJ number gene).

>human-Hs-p47*phox*: GenBank^TM^ No. NM_000256

MGDTFIRHIALLGFEKRFVPSQHYVYMFLVKWQDLSEKVVYRRFTEIYEFHKTLKEMFPIEAGAINPENRIIPHLPAPKWFDGQRAAENRQGTLTEYCGTLMSLPTKISRCPHLLDFFKVRPDDLKLPTDNQTKKPETYLMPKDGKSTATDITGPIILQTYRAIANYEKTSGSEMALSTGDVVEVVEKSESGWWFCQMKAKRGWIPASFLEPLDSPDETEDPEPNYAGEPYVAIKAYTAVEGDEVSLLEGEAVEVIHKLLDGWWVIRKDDVTGYFPSMYLQKSGQDVSQAQRQIKRGAPPRRSSIRNVHSIHQRSRKRLSQDAYRRNSVRFLQQRRRQARPGPQSPGSPLEEERQTQRSKPQPAVPPRPSADLILNRCSESTKRKLASAV

>dog-Cf-p47*phox*: DDBJ^TM^ No. BR000287

MGDTFIRHIALLGFEKRFVPSQHYVYMFLVKWHDLSEKVVYRRFTEIYEFHKMLKEMFPIEAGDINPENRIIPHLPAPRWFDGQRAAESRQGTLTEYYNTLMGLPVKISRCPQLLDFFRVRPDDLKLPTDSQVKKPETYLVPKDGKSSVTDITGPIILQTYRAIADFEKTSSSQMALATGDVVDVVEKSESGWWFCQTKTKRGWVPASYLEPLDSPDEAEDPEPNYEGEPYVTIKAYTAEMEDEMSLQEGEAIEVIHKLLDGWWVVRKDDITGYFPSMYLQKSGQDAAQAHRQIKSRGAPPRRSSIRNAHSIHQRSRKRLSQDTYRRNSVPTQPGKPQPAVPPRPSADLILHRCSESTKRKLASSV

>mouse-Mm-p47*phox*: GenBank^TM^ No. NM_010876

MGDTFIRHIALLGFEKRFIPSQHYVYMFLVKWQDLSEKVVYRKFTEIYEFHKMLKEMFPIEAGEIHTENRVIPHLPAPRWFDGQRAAESRQGTLTEYFNGLMGLPVKISRCPHLLDFFKVRPDDLKLPTDSQAKKPETYLVPKDGKNNVADITGPIILQTYRAIADYEKSSGTEMTVATGDVVDVVEKSESGWWFCQMKTKRGWVPASYLEPLDSPDEAEDPDPNYAGEPYVTIKAYAAVEEDEMSLSEGEAIEVIHKLLDGWWVVRKGDITGYFPSMYLQKAGEEITQAQRQIRGRGAPPRRSTIRNAQSIHQRSRKRLSQDTYRRNSVRFLQQRRRPGRPGPQSTDGTKDNPSTPRVKPQPAVPPRPSSDLILHRCTESTKRKLTSAV

>zebrafish-Dr-p47*phox*: Ensembl No. **ENSDARG00000033735**

MAETYVRHVELLGFEKRFFPSQHYVYMLLVKWSDQSEKLVYRRYPEVHTLHKTLKEMFPIEAGDIDEKDRIIPTLPAPKWLDNQKTTETRQATLAEYCRSLLNLPANISRCQLIRDFFKMRPEDETPPAPHPYKRNETFIMSTNRVRSNTTSEITGPIILETYRVIADYSKSSKYELTLKMGDMVDIVEKSPNGWWFCQCESRRGWVPASYLEPLDGADESEEPEPNYAGELYKTTRGYKAVEQDEMTLEAGVIIEVIHKLLDGWWVVRKGEETGFYPSMFLCRTGEKKEVDAERDVVRRATPPPRRSTIRNAQSIHSTVRRRISQDSYRKQSRRFLQQRGRLNSHSRIGTRSPLQERRTNKNIEKSSAPQAEDEDKSVPVVPPRPSPQLILERCTENTSKRMSMQEA

>medaka-Ol-p47*phox*: Ensembl No. ENSORLP00000024397

MEDVYVRHVELLGIEKRFFPTQHFVYMLMVKWSDQSEKLIYRSYPEIHTFHKSLKDMFPIEAGQIEAKDRIIPKLPAPRWLESQKSRENRKTTLVDYCHLLVSLPPHISRCKELSNFFKVRPEDENPPAPNITKRNQTFVVSKEPAQGTAAEISGPIILDTYRVIADFEKTSKHELNLHDGDLVEILEKNSNGWWFCQCEAKRGWVPASYLEPLDGPEEAEEAEPDYEGELHVTTNAYKAEQDDEISLDLGETVEVIHKLLDGWWVVRKGDETGHFPSMFLTKASKRIQTLRTNLHGQRPPPRRSTIKNAKSIHNRSRQRLSQEAYRRNSRRYLQQKGGRLASPHRSSRGSGKSPLTERKNHDNIPELHGSETETKRETPVIPPRPSPELILERCTANTCKKVSIHRSQSGSSSQNQDQ

>chicken-Gg-p47*phox*: GenBank^TM^ No. NM_001030709

VGDTFIRHIELLRYEKRFFPSQHYVYMFLVKWNDLSEKLIYRRFTDIYEFHKALKEMFPIESGDINAENRIIPHLPAPKWFDGQRSTQSRQGTLAEYCYTLVNLPHKISRCRHVVSFFEVRPDDMNPVTDSQIRKPEVFLLPKDAKKNTSDITGPIVLQTYRAIADYEKSSKSEMAVKAGDAVDVVEKSETGWWFCQLKTKRGWVPAAYLEPMDGPDESEEQEPNYAGELYVVQKSYTAVEEDELTLKEGDTIEVIHKLLDGWWVIRKDETTGYYPSMYLQKSGEVNSPEKSGLRNHNIPPRRSTIRNAKSIHNKGRKQISQETYRRNSKKYMQNRRNMRGNLQNKDIISEKNEQEENKSKAQPAVPPRPSKDLIMNRCTESTRRKI

>rat-Rn-p47*phox*: GenBank^TM^ No. NM_053734

MGDTFIRHIALLGFEKRFVPSQHYVYMFLVKWQDLSEKVVYRKFTEIYEFHKMLKEMFPIEAGEIHTENRVIPHLPAPRWYDGQRAAESRQGTLTEYFNSLMGLPMKISRCPHLLNFFKVRPDDLKLPNDSQVKKPETYLTAKDGKNNVADITGPIILQTYRAIADYEKGSKTEMTVATGDVVDVVEKSESGWWFCQMKTKRGWVPASYLEPLDSPDEAEDPDPNYAGEPYVTIKAYAAVEEDEVSLSEGEAIEVIHKLLDGWWVVRKGDITGYFPSMYLQKAGEEITQAQRQIRSRGAPPRRSTIRNAQSIHQRSRKRLSQDTYRRNSVRFLQQRRRPARPGPQSPDSKDNPSTPRAKPQPAVPPRPSSDLILHRCTESTKRKLTSAV

>frog-Xt-p47*phox*: Ensembl No. **ENSXETG00000018448**

MTEPHIRHIQLLGFEKRFIPSQHYVYMFMVKWQDLTEKLVYRKFTEIYEFHKSLKEMFPIEAGDISKEHRTIPHLPAPKWFDGLRSTENRQVTLSDYFSSLLSLPPKISRCPHVLNFFQVRSDDVNPVANNTNGRKPETFLLKVDTAKKNVSDITGPIILQSYRVIADYEKNSKSELAAKNGDVVEIVEKSENGWWFCQLRNKRGWMPAAYLEPLDGPDESEEQDPNYEGDLHITTKDYSGELDDELSLQEGENVEVIHKLLDGWWVVRKGSITGYFPAMYLQKSGETAPANENPSKRKGLPPRRSTISNANSIHKKERKQISQDTYRRNSKKYLKQRQSIVDTKSPIITEENKEEESKSKPQPAIPPRPSKELILDRCSENTKSKI

>tetraodon-Tn-p47*phox*: DDBJ^TM^ No. BR000288

MADTYVRHVQLLGFEKRFFPSQHYVYMLLVKWSDLTEKLIYRTYPEIYTFHKALKEMFPIEAGKIEKRDRIIPSLSAPPWLDSQKSTETRQTSLSDYCQALVNLPPHISRCTHLTSLFKVRPEDENPAAPNTLKRNETFVVSRDLARGNASEISGPIILDMYRAIADYTKTTKYEINLLAGDQVEIVEKNQNGWWFCQMDSKRGWVPASYLEPLDGPEESEEADPDYEGSELFITIKAYKAEQEDEISLDLGESIEVIHKLLDGWWVVRKGEGTGYFPSMFLQKASKRAQAEAARNHLQGQKPPPRRSTIRNAKSIHNKSRQRLSQDAYRRNSRRYLQQKGGQRDLQNKYARTAAKSPLQERKNQGNIPEESGTASEGEPKKEAPVVPPRPSPELILQRCSDNTRKKIS

>fugu-Tr-p47*phox*: GenBank^TM^ No. AB099897

MAETYVRHVQLLGFEKRFFPSQHYVYMLLVKWSDLTEKLIYRTYPEIYTFHKSLKEMFPIEAGKIEKRDRIIPSLSAPPWLDSQKSTETRQTTLSDYCHSLVNLPPHISRCTHLTGFFTVRPEDENPPSPNILKRNETFVVSKDLARGNVSEISGPIILDMYRAIADYTKTTKYEINLHAGDQVEIVEKNQNGWWFCQCDSKRGWVPASYLEPLDGPEESEEAEPDYGGSPCELYITIKAYKAEQEDEITLDLGESIEVIHKLLDGWWVVRKGEQMGYFPSMFLQKANKREQSESSRANVQGHKPPPRRSTIRNAKSIHNKSRQRLSQDTYRRNSRRYLQQKGGQLVKPDPYPRNVAKSPLRERRNQGNIPEESSTISENEGKREAPVIPPRPSPELILQRCSDNTRKKISIHKSSSASTSKPGQA

>ascidian-Ci-p47*phox*: GenBank^TM^ No. NM_001033828

MVNRTLKSVKVIDIEKRRLPSKHYVYLIEIKWSDGSLCTVGRRFSAFFMMHMTLLEKFPLEGGQKDPSRRILPFLPGKILFKRSHTRDVTLKRLGSISEYCESLLLLPEHISQCDTILRFFETSSSDIARDTKEKTQSTAVSQIIQDITGPIELETYIAIADYKAEAKTQISLHSGETVEVVEKSESGWWLVCNTYGSNGWVPGAYLEKEDGSEEDLVTEKAAVGQGTWYVATSHYDATSNDEISFPMGAALEVLQVNLEGWWLARYNSNEGWVPGSYLEKSRRTYSWATDTAPTESVPGAAESVKKSTLALVKPPPKRATIRRTLKVTRGQDSIKEKHDEDNLYITLFDFDSSIEDGLSFKAGQIVKVIEQSDNGWWLATLNGAEGWVPSSYLESKTTEAEPARNSNPGFSAKMVLPKIEETGAGRSYDPGQAKQKLIGSPLKLKLEGKERNTPFQLQAK

>sea urchin-Sp-p47*phox*: GenBank^TM^ No. XP_001183696

MGKRTVVNANVTDIEKRREPTKHYVYIIHVTWSDGSVNVVYRRYSTFFDFQNKLLSKFPEEAGANNPSSRCIPFLPGKKLFGRSHIREVALKRLSPIDEYCTALVKLPGKISDSKEVINFFTPTPEDVSPPSPDGGGESTRGRADIGNISEPIQAEQYIVVADYKKQQKNEVELTAGDLVEVFEKNDNGWWFVTVHDQHGWAPGTFLQNPDGQEEEDEETLIPGNDESYITNNAYQGQAEDEISFETGVVVTVIQKSLDGWWKVSYQGKQGWAPATFLQIYKGPSGVTPKHPTQSIGNVMLLKSGSDSKPKPGPGPGSSGRPQPPVQPRDEPGQLYSNYDAEVKPTPPRRATVKKSVRRGGVRQTKPKLKKVMEHYTTDSFQGAAGEGSISFESGQKVEVLEENDGGWWYVKMNGQEGWAPSNYIEKREVSSRINGKPSLGNLDEETGEDKVPPVLPARPAFGSGGSGAFKPVAKKSTPPPVDRSNSPSFTNRKAVTNNGRGGGDGVRRGSGGVGAGGNMMAALKKQLEKSSVGESSSPAIPARPGVAPKLSKPTAQPYCNRDAAYITTSSYVNDENDGLSFEEGQRVEVIKKDDSGWWSVRIGNTEGWVPNTFLEKI

>human-Hs-NOXO1: GenBank^TM^ No. NM_172167

MAGPRYPVSVQGAALVQIKRLQTFAFSVRWSDGSDTFVRRSWDEFRQLKKTLKETFPVEAGLLRRSDRVLPKLLDAPLLGRVGRTSRGLARLQLLETYSRRLLATAERVARSPTITGFFAPQPLDLEPALPPGSRVILPTPEEQPLSRAAGRLSIHSLEAQSLRCLQPFCTQDTRDRPFQAQAQESLDVLLRHPSGWWLVENEDRQTAWFPAPYLEEAAPGQGREGGPSLGSSGPQFCASRAYESSRADELSVPAGARVRVLETSDRGWWLCRYGDRAGLLPAVLLRPEGLGALLSGTGFRGGDDPAGEARGFPEPSQATAPPPTVPTRPSPGAIQSRCCTVTRRALERRPRRQGRPRGCVDSVPHPTTEQ

>dog-Cf-NOXO1: GenBank^TM^ No. XM_547183

MAGSRHPVSVRAAALVQTGRLQTFAFSVCWSDGSDTFVRRSWAEFKELHKTLKEAFPVEAGLLRRSDRILPKLPDTSLLVRGGRTGRGLARLRLLDTYTRALLAAAEQLSRSAVLTGFFEPQPVDLEPVLPPGSLVILPTPEEPHRRPPHSPAICSLEAQSLRCLQPFSTQDTQGWPFHARAQEVIDVLLRHPSGWWLVANEEQQMAWFPAPYLEEAAPDREGTTLRSSGSQFCASQAYESSHADELSVPAGARVSVLETSDRGWWLCRFRGRSGLLPAVLLQPEGLGALLSGPGLHREANSKEDRGGEAQRTPEACQATTLSPSVPARPPLSAIRSRCCSVTRRALASKYPPRAGQ

>chicken-Gg-NOXO1: Ensembl No. ENSGALT00000008938

YMMFVSWSDQNNILIYRTLEEFKRFHKELKRKFPIESGSLRRSDRTIPRFKDINGKQKKSGKINRSLERLKLLETYTQELLKVDAKISQGEDVIQFFKAQTQDLDPCFPEDSVVIMPSEIGGEKKKEVQQQQLSITYPQVSQSYRCIETFETKDTKNKTFKVAKKEIVEVLLKDMTGWWLVENADKQIAWFPASYLEQISAHKDIQNVESSDEEGSLYFVMRAYEAQKADELSLNKGVVVEVVRRSDNGWWLIRYNGRKGYMPSMCLQAYKNPHHRLQTIMNSGLHISTPNLCSPSPALQPLRDSTARDCTSGDGSDEDLESDSSSLSSGSAPSGVLSWKPDLSRSLPEVEQAVPMRPSAHEILQRCSTVTKRAVQQSA

>mouse-Mm-NOXO1: GenBank^TM^ No. NM_027988

MASPRHPVSAHAVALVQMDRLQTFAFSVCWSDNSDTFVRRSWDEFRQLQKTLKKTFPVEAGLLRRSEQVLPKLPDAPLLTRRGHTGRGLVRLRLLDTYVQALLATSEHILRSSALHGFFVPKPLDLEPMLPPGSLVILPTPEEPLSQPRGSLDIHSLEAQSIPCVQPFHTLDIRDRPFHTKAQEILDILLRHPSGWWLVENKDQQVAWFPAPYLEEVATCQGQESGLALQGSGRQFCTTQAYEGSRSDELSVPSGARVHVLETSDRGWWLCRYNGRTGLLPAMSLQPEGLGSLLGRPGFPDSAGADKVAEDRTIPPVVPTRPCMSAIQSRCCSITRRALGQEQGTRVPR

>rat-Rn-NOXO1: GenBank^TM^ No. XM_220221

MASPRHPVSAHAVALVQMERLQTFAFSVCWSDNSDTFARRSWEEFRQLQKTLKKIFPVEAGLLQRSERVLPKLPGQACRNAPLLTRRGHTGRGLLRLRLLETYVRSLLATSQHIVTSSTLNSFFAPKPLDLEPMLPPGSLVILPTPEEPLSQPIGSLAIHSLEAQSMRCLQPFHTLDTKDRPFHTQAKEILDILLRHPSGWWLVENKDQQTAWFPAPYLEEIATGQGQESGMAVQGSGRQFCATQAYEGSRPDELSVPSGARVHVLETSDRGWWLCRYNGQTGLLPAVLLQPEGLGSLLGRPGLPDSGGADKVTEGRTVPPVVPTRPCMSAIQSRCCSITRRAVGQEQRTQVPP

>frog-Xt-NOXO1: Ensembl No ENSXETT00000015702

WSDHNEILIYRTFEDFKKLNRQLKKKFPLEAGLFRKSDNLLPKLKDVPIFRKNRTTNRFIERLRLLEKYSQELLRTDGKISQCDLVLKFFTPSNNDLNPKFPENSLVMMTSDSKDQKEQKKPLPEAPAIHPIVSQQYICMEDYETKDTKNRPFKVKRHELVGVLIKENTGWWLVENEEKHLAWFPAPYLKDVDNSEDTDSGTSEDEGVLYYAAKAYEAMNSDEVSITVGVLVEVIEKSNNGWWLIR

>zebrafish-Dr-NOXO1: DDBJ^TM^ No. BR000290

LIKLFIRRVEHRNMDINIHSSPPQLYMTTVLWSDGNEITVYRSLEDFKKMHRQLKKKFPPSNPFKRSARIVPEFKGNKWSGSKSVLRMKALEEYCGQLLKSDAQVCRSSELIQFLLPKAHDLNADFAKNCIVIMPSDVTLGSSKAESNSGVTQPFVTETYRCIANYETKDTKNRPFKVEVDETVDVLIKDQKGWWLVENESKHLAWFPAPYLERAEMADDGPDEMDNESFQSAGVFYVATKAYKATNSDELSVELGSVLEVLQKSDNGWWIVRYNRKAGYVPSMYLQPHNNPRILLKSTQKEISRSTLDLAQLQHPQTLQDSRLRELSRSQGNLLLQPAETDIMDKQKSDPCLNKMPSTPKVPPRPAVQEILTRCTTVTRKNMQ

>tetraodon-Tn-NOXO1: DDBJ^TM^ No. BR000291

METQRYPISARLVGVLHKEKSKVGAHKSQVVLFHARFNQQLCPLQMYMTSVLWSDHNEIVVYRTFQDFRKMHKLKRSGKKKSPTRSLVRLKFLQKYCNELLSCEPRVSQSADLIQFFHPNAQDLEPEFSKNRQEEVKAEAGHGSVGNVTQPFVTVTYRCVSQYETKDTKNKPFKVAADEKVDVLIKDKAGWWLVENEEKRMAWFPAPYLEKLEEDGDEDDTDGTRTLYLTAKNYKASKGDEISVAVGAVVEVLQKSDSGWWLIRYQGKVGYVPTLCLQPYNRPQVRLNGAAFPGLQQQSNKLSSSRGNLLQLPSAGRSPSPQQPHADGRQRSHSLNALLETLPAQPARGAAPDTGTPPSPQQAPPPVIRPQYPRGQNDAQRLRSQHLQGSRNPQGSTQTPGPGDPHQVHHHHPQECQQRRSVAHPTRDTESVKLPLARFSCSFRLHA

>medaka-Ol-NOXO1: Ensembl No ENSORLP00000003961

PADTHMSLSVSAPGSDKWLLPSDDRKYTFMVSGIWSDGSEIIIYRSFKDFKKFHDQLKKQFPNLTPFRKEDRMLPKFNGKARRSLKQKGSKKSVKQMEFLESYCDKLLKCDPNVTQSSEVTRFFTPKDQDLQPDFTKNSSLLLVYGRPGALRSSGAGNVTHPFVTQTYRCVAPYETRDTKNRPFKVAVDEKLDVLIKDPAGWWLVESENKRLAWFPAPYLEVLDGEDEDDEGNLGGSLYCAVRSYSTKKNDEVPLSIGSVVEVLRKSDDGWWLIRFNGKVGYIPAMYLQPYNNPRTGLHGQHNKIHTSTLNLSTMKDQQVPPSIPRRDSPSSDSSTSLREEAQGDAPRLQEDSQSSGEAASDRRSTNSSGFDESTGPRVPPRPKAEEILTRCTTMTRKAALATKTR

>human-Hs-p67*phox*: GenBank^TM^ No. NM_000433

MSLVEAISLWNEGVLAADKKDWKGALDAFSAVQDPHSRICFNIGCMYTILKNMTEAEKAFTRSINRDKHLAVAYFQRGMLYYQTEKYDLAIKDLKEALIQLRGNQLIDYKILGLQFKLFACEVLYNIAFMYAKKEEWKKAEEQLALATSMKSEPRHSKIDKAMECVWKQKLYEPVVIPVGKLFRPNERQVAQLAKKDYLGKATVVASVVDQDSFSGFAPLQPQAAEPPPRPKTPEIFRALEGEAHRVLFGFVPETKEELQVMPGNIVFVLKKGNDNWATVMFNGQKGLVPCNYLEPVELRIHPQQQPQEESSPQSDIPAPPSSKAPGRPQLSPGQKQKEEPKEVKLSVPMPYTLKVHYKYTVVMKTQPGLPYSQVRDMVSKKLELRLEHTKLSYRPRDSNELVPLSEDSMKDAWGQVKNYCLTLWCENTVGDQGFPDEPKESEKADANNQTTEPQLKKGSQVEALFSYEATQPEDLEFQEGDIILVLSKVNEEWLEGECKGKVGIFPKVFVEDCATTDLESTRREV

>mouse-Mm-p67*phox*: GenBank^TM^ No. NM_010877

MSLAEAIRLWNEGVLAADKKDWKGALEAFSEVQDPHSRICFNIGCVNTILENLQAAEQAFTKSINRDKHSAVAYFQRGMLYYRMEKYDLAIKDLKEALTQLRGNQLIDYKILGLQFKLFACEVLYNIALMHAKKEEWKKAEEQLALATNMKSEPRHSKIDKAMESIWKQKLFEPVVIPVGRLFRPNERQVAQLAKKDYLGKATVVASVVHQDNFSGFAPLQPQSAEPPPRPKTPEIFRALEGEAHRVLFGFVPETPEELQVMPGNIVFVLKKGSDNWATVMFNGQKGLVPCNYLEPVELRIHPQSQPQEDTSPESDIPPPPNSSPPGRLQLSPGHKQKEPKELKLSVPMPYMLKVHYKYTVVMETRLGLPYSQLRNMVSKKLALSPEHTKLSYRRRDSHELLLLSEESMKDAWGQVKNYCLTLWCEHTVGDQGLIDEPIQRENSDASKQTTEPQPKEGTQVVAIFSYEAAQPEDLEFVEGDVILVLSHVNEEWLEGECKGKVGIFPKAFVEGCAAKNLEGIPREV

>dog-Cf-p67*phox*: DDBJ^TM^ No. BR000293

MSLAEAISLWNEGVLAADKKDWKGALEAFAAVQDPHSRICFNVGCMHTILGNMPEAEKAFTRSINKDKHLAVAYFQRGMLYYHMEKYDAAIKDLKEALTQLRGNQLIDYKILGLQFKLFACEVLYNIAFMYAKNEEWKKAEEHLALAMSMKSEPRHTKIDKAMECVWKQKLYEPVVIPMGRLFRPNERQVAQLAKKDYLGKATVVASVVDQDSFSGFAPLQPQAAEPPPRPKTPEIFRALEGEAHRVLFGFVPETPEELQVMPGNIVFVLKKGNDNWATVMFNGQKGLVPCNYLEPVELRIHSQQQPQEEASPESDIPAPPSSSAPGRPQLPPGWSSFCFLSPQDVKLRVPMPYTVKVHYKYTVVMEIQAGLPYSQLRDMVAKKLELLPEHTKLSYRPPDSHELEPLSEDNMKAAWGQVRNYCLTLWCENTVGDQGFPDEPQESEKSEANNQTTEPTLKEGGHVVALFTYEATQPEDLEFQQGDIIQIISMVNEDWLEGECKGKIGIFPKAFVEEHATTDLESSPRGV

>zebrafish-Dr-p67*phox*: DDBJ^TM^ No. BR000296

MSFVSTLRQWDEAVACVEQRDPDAALRIFLSIEEKNSKIAFNIGCLCLNNSDLDEAEKAFDGSIGKDEHLAVAFFQRGVTFYKKEKFEESLLDFQQAFKQLRGNQLIDYTPLGLRYKLYACEVLHNIGLAQAQLGKWEKAQENLLTALSLRADAKFSHIDHALDAILKHKLFPLVEVRAGLLFKPNKKYVAELEKRDYLGKAKVVASVVPADEFSGFAPLQPQIDNVPSIPKVPEVLRVLEGEPHTVLYEFVPETKEELAVLPGNIVFVLHRGTDNWASVVFNEKRGLVPYNFLEPLDIVTMTSKPVETEALNENDDIPAPPRRAAPSRPVAPEGLKTVNAKLYSPQTVNTREFSGCVVKVHFQFTIAIAIAHGQPYGVILQMISSKLKLPASTLTLRYAKEGSAERVIIEDSEMEAVWNSAKDGRLTLWCSVTEGKSASHAKVVALYSYESSTPEDLEFKQGNVITVLSKVNDEWLEGQCNGKIGIFPSSFVEPLNGDPH

>chicken-Gg-p67*phox*: DDBJ^TM^ No. BR000295

MSLVETIRLWQEGVCAADGKEWGAALKAFTAVQNPPAKICFNIGCTHLVLGQLAEAEEAFTQSISCDKHLAVAYFQRGTVFYKRHNHEMALKDFKEALAQLRGNQLIDYKILGLRYRLFACEILYNIALVYATMENWKKAEEHLTLAMSSKSEPQHNKIDRAMEAILKQKLYEPVAIPTGKLFRPNEKQVAQLEKKDYLGKAMVVASVVDKDSFSGFAPLQPQASGPPPRPKTPEILRALEGQPHRVLYEFIPETAEELQVLPGNIVFVLKKEKDNWATVMFNGKKGIVPCNFLEPMELQHKLHVQDEAPLEPDIPEPPSSTAPRPRRPAPGQEKPDTPIHHLQESEPDIPKPYVLKVHYKYTVAMQVKPDLSYKELLGLVCDKLELQPEHTMLRYKSAASGELVPLSAQNLEEAWSHSKDQCLTVWCDCTEGEGFLPDSKPEEPQQAAAETGPTQVVAQYSYEATQPEDLEFQAGDVILVLSKVNEDWLEGQCNGKIGIFPSAFVRDGNTKDP

>rat-Rn-p67*phox*: DDBJ^TM^ No. BR000294

MSLAEAIRLWNEGVQAADKKDWKGALEAFSEVQDPHSRICFNIGCMYTILDNLQEAEQAFTKSINRDKHLAVAYFQRGMLYYSMEKYLLCVYCLREALVTFRKKQVLSPKPEGLQFLLQRSPVLYNIALMHAKKEEWKKAEEQLALATNMKSEPRHSKIDKAMESIWVSVVVDGPCCLAQYHVQRASHKQVPVLSKDLLGEETVVASVVHQDNFSGFAPLQPQSAEPPPRPKTPEIFRALEGEAHRVLFGFVPETPEELQVMPGNIVFVLKKGSDNWATVMFNGQKGLVPCNYLEPVELRIHPQSQPQEDTSLESDIPPPPNSSPPERLQLSPGHKQKEEPKEVKRSVPMPYMLKVHYKYTVVMETQLGLPYSQLRNMVSKKLELLPEHTKLSYQRRDSPELLLLSEESMKDAWAQVKNYCLTLWCEHTVGDQGFVDEPKEKENSDADNRTTEPQPKEGTQVVAIFSYDATQPEDLEFVEGDVILVLSHVNEEWLEGECKGKIGIFPKAFVEGCAAKNLEGTPREV

>frog-Xt-p67*phox*: DDBJ^TM^ No. BC099979

MALVEIMRLWSEGVAAAENEDWNGALKSFTSITDPRSKICFNIGCCHLVLGDLEKAEKAFTLTIERDMHLAVGYFQRGFVFFQRGKYSLALQDWTRAYTEMRGNQLIDYKILGLIFKLYSCEILHNIALTHAKEGKWAKAEESILLALSQKVELRHNTKLEKAMEDILKEKVFAAVKIPKGRIFQPNERLVEQLEKKDYLGKALVVASVVDKDSFSGFAPLQPQASNPPPRPKTPEILRTLQGEPHRVLFEFNPETAEEMQVLPGNIVFVLKKGDDNWATVVFNGKKGIVPCNYLEPVELRFQSAQQTGVQSELDSPTNRPQQSDVPAPPDATPPQLLKNTKEAVAVASYLVKVYYKYTVAIQISSKLPFADLLTLISSKLQLLPSRMKLSFKEDQDDVLLNEENTEKAWSLATDNCLKLKCTEVQVRQARSLYCSTMQGYFKAQGKYPIALFEYEATQPEDLPFCKGDIIKILSHVSEDWWEGECQGRMGIFPKVFTEE

>tetraodon-Tn-p67*phox*: DDBJ^TM^ No. BR000297

MSFLDTLRQWDNACTVADGQDFSAALQVFLSIQEPNSKICFNIGCLHLLNEDLSAAEKAFDSSISKDEHLAVAFFQRAITFYKMSRQTHRPCHFQQTFKELRGNQLIDYGALGLRYKLTACEVLHNMALAEAQLGRWEKAQESLVKALDYSSDSKLGAIDKALQATLKQKLFKLTGFPSKVLFKPNKRYVAELEKKDYLCKAKVVASVVPQDEFSGFAPLMPQVRPHLRAASSPTAESNSARVHRALEGEPHTVLYEFVPETSDELAVVPGNVVFVLQKGADNWASVVFNERRGLVPYNYLERLEISLAAKLKDVRTCQPGKPRPPSQQPPTRPERNLLTLFSGLEFQEAQLVDDSYVVKVRFTFTFAVIVPRGSSYATLAQKVGEKLSVPADAVILSLSSEAAEEDVINGSTDMEAVWGRASGRCITLWCRLAEQTSETEPRRETFLLALHTYDSPNPEDLTFQQGDKILLLSKVNQDWLEGQCHGNTGIFPAAFVEEVSVSE

>fugu-Tr-p67*phox*: GenBank^TM^ No. NM_001032682

MSFLDTLKQWDKASTVADRQEFSEALEIFLSIEEPNSKIYFNIGCLHLLNEDLKDAEKAFDSSICKDEHLAVAFFQRAITFYKMTRQEYSLADFQQTLKELRGNQLIDYGALGLRYKLNACEVLHNIALTEAQMGHWEKAQESLVKALDYRTESKLGIIDNALQATLKQKLFKLIGFPSKVLFKPNKHYVAELEKKDYLGKAKVVASVVPQDEFSGFAPLMPQVESGQTFSKPEPELLRALEGEPHTVLYKFVPETSDELAVVPGNVVFVLQKGADNWASVVFNERRGLVPYNYLERLEITMASKQNNVQSRPPSRQPPTRPERKSGLPPCADDRRNTQMKESELADDSCVVKVRYTFTFAVLVPRGSSYATLAEKISEKLSVPANAIVLSLSSEATEQNVIDGGTDMEGVWSRVSGRCITLWCRLAQTNERVQKESSLLALHSYDSSNPEDLSFHQGDRITLLSKVNQDWLEGEFNGNTGIFPAAFVEEVPANG

>medaka-Ol-p67*phox*: Ensembl No. ENSORLP00000006791

MSFVDTLRQWDDGVTCADKQDFSEALRILLAIPEPNSKICFNIGCLHLLNQNLDDAEKAFDCSIRKDEHLAVAFFQRGITFYKKMRYEESIGDFQRAFKTLRGNNLIDYKALGLRYILYACEVLHNMALAEAQLGNWEKAEQNLRKALDYKTDAKLSVIEKAQQAVLKEKLFKLVEFPSKMMFKPNKHYVAELEKKDYLGKAKVVASVNPQDDFSGFAPLQPQVEDGPLSPREPEVLRALEGEPHTVLFEFVPETCDELAVKPGNMVFVLQKGADNWAYVIFNGRTGLVPYNYLERQEISLALKQKKTEKNSRRNELKPPRKTDQRAVPGPGSGSKVPDSLYIVKVHFTFTFAVSIPRESSYEVLIRKISKKVKLPSDNISLSFSLDSSGQRAIDANTDMETVWSQVRAGRITLWCKEKKNTYGFESSNLNSPPYILGFQQEEGEETHKVALHSYQSANPEDLNFKEGDEITVLARINQDWLEGRCNGSTGIFPASFVNFCSKPRQL

>ascidian-Ci-p67*phox*: GenBank^TM^ No. NM_001033827

MASKHAALLQQEGVEASEENDWFKALSIFQQVPNPSSVIWFNIGCCHLQVQQYLKAENAFSQSIAKDKYLVAGYFQLAVSQTHLGNYAEAIDNFSSALSSLRGNPFIDYKQLNMLCKISACDIRLNLALLHIFSGDVPKAREILNEAMSMPHDEDKMKNCKSALDALVNENWVYFGESALERLVRLSSSCLFRPSKTKMEGLKSGTKFMNTATVVSATNDEYSFVGFVGPKKMQQQREKEELFPINAGQSSHSIVPPPLPSVAPPRRTRSNPPTLPPPVPPGNSPSKPKPEVKDLTKLPPKPLKPLDKKLAMPKPIPKPANSKVLSSPTMSPKSSPGKRSPPVRPAKPSVNSYLQCSLSLSLSLPKNKIGPHDNFLIELSSKLDHLVKALVAPNFSDNYQMTLATIGAAQEITSSTWRETYLDAVRSKKLSINITPLRSKELPKTPKPTEPNGSNIQRPAITEAALKPAVAKKPNQNWPPAPPEDADIYVDANNAASLIPEANEDNIYAEAVFGV

>sea urchin-Sp-p67*phox*: GenBank^TM^ No. XM_001186087

MDKQTHKDIIQVWHEGVQTFDKGNVDAALQAFLGIGDPSAKILFNIGHLEMSQYRFGEGEVNFTAAVEKDPHMALAHFQLGMVYFHLHRYNESRNSYEKAKVCLRGNRFIDYRQLGFVHKLYECEVLYNLALTYATMMNNSEYALELLDEAKKVSVEARHGPLIEKAQKRLLLYEPVVLPSNQLFRPPKSKVVGLKNVDYLGKPKVVSELPESSNNNNETNFQPSPNSFRPIYDDFSDDDDLSPTFNGGSSPTPSTVTKMTHKDTIATWYEGVLAFERGESSEALNQLNSIVDPSAKILYNIGILQKSLGQLEDANSILKEVVSRDPHLAIGHFQSGVVLGLLGRGDDAWHAFEKARETLRGKMINYKPLGLQYKLHKCEVLHNQAWAYNELDQRDWARNQLEEARECKADPRHEKIEDSYRAFTSGRSFKLYELPKPLLFKPPKSKIDNLDRKDYLGKAKIVAKLPTVRKRSSTPPPLKTSSSLGVPVLSPRSKTPTPSSAPSKALPVPKTQSLSSPGLPDRPVPSRQPRPSLPDTQAPLMMPVVTVESLQNSKPPSRPPPSQSGGFNFDKVLPEAPAPSRGALSSSVPQRPLPVTPGRKMSSELAPSRPLPSPSRLSPVPNRPLPASPGKNNSSDLPPSRPLPSPTRLSPVPDRRGKKNSPELPPSRQLPSPSRSSPVPDRPLPQRPVPTKSSDAPLSRPLPSPKRSSPPPPDRPLPGPPRKSASPDLPQRPLILAPSDSDVPSRPLFLADRPVPARPGNASPELPSRPVPPPPGTDEPDLPSRPAPIASRISDLSSRTVPLSPNAGRKLPGIPVTKNPFFDMSNRQVPALPTEGPQDDLPSRPPPPKPTQSMPELPPRPVPSPPIKQLSLDSLSTRPVPPPPGSNTEPELPPRPKPSGGNLLQDRPIPPPPSSSHAINLLNPGRRSSSPGLPDLPPPPPPKKAPSPNPPRKNINNNHVASYGGKLAGNKAKLVKIVSDSPPVVRRAAAVTVTVQKEAKPTPVERSKPATADKPAAAGKQPPILAFKPELKQRVAKGAGPSKPGQRKTALVIASHETDVEGEVSVSEGDLITITGEVGDWLEVEVKGRKGRVPRSCVKDFSKPRV

>human-Hs-NOXA1: GenBank^TM^ No. NM_006647

MASLGDLVRAWHLGAQAVDRGDWARALHLFSGVPAPPARLCFNAGCVHLLAGDPEAALRAFDQAVTKDTCMAVGFFQRGVANFQLARFQEALSDFWLALEQLRGHAAIDYTQLGLRFKLQAWEVLHNVASAQCQLGLWTEAASSLREAMSKWPEGSLNGLDSALDQVQRRGSLPPRQVPRGEVFRPHRWHLKHLEPVDFLGKAKVVASAIPDDQGWGVRPQQPQGPGANHDARSLIMDSPRAGTHQGPLDAETEVGADRCTSTAYQEQRPQVEQVGKQAPLSPGLPAMGGPGPGPCEDPAGAGGAGAGGSEPLVTVTVQCAFTVALRARRGADLSSLRALLGQALPHQAQLGQLSYLAPGEDGHWVPIPEEESLQRAWQDAAACPRGLQLQCRGAGGRPVLYQVVAQHSYSAQGPEDLGFRQGDTVDVLCEEPDVPLAVDQAWLEGHCDGRIGIFPKCFVVPAGPRMSGAPGRLPRSQQGDQP

>dog-Cf-NOXA1: DDBJ^TM^ BR000298

MPSLGDLVHDWHRGVQAVARGDWGCALRLFSGDPDPPAKMCFNLGCVHLLAGDPEAALRAFDQAVTKDTCMAVGFFQRGVANFQLERFQEALSDFRLALAQLRGNAAIDYTQLGLRFKLKTWEVLFNVGAAQCALGLWAEAAGSLEEALCKGPEGAGEDLHAALAQVQKQATLQLRQVPRGEVFRPHRRHVEHLEPVDFLGKAKVVSSAIPDDHLQGSRPQQRQVSGAPSSQPPNGPGDPSCGASLACSPHPSAPRPSQLGPQRAALGWTEPGGCLSASSQVVATGGPESLVTVTVQCAFTLALKVPWGAGLPHLRTLLSQALPLQAQHGQLSYRDPSHEARWVALPGEEALQGAWRDTAASPRGLQLQCRAAGSRPVLYQAVAQHNYCAQGPEDLDLRQGDMVDVLCAGQGPDVPLHVVDPAWLEGHCDGRIGIFPKCFVVPAG

>mouse-Mm-NOXA1: GenBank^TM^ No. NM_172204

MSSLGDQIRDWHRGVLAVAREDWDSALCFFSDVREPLARMYFNRGCVHLMAGDPEAALRAFDQAVTKDTCMAVGFLQRGVANFQLQRFQEAVSDFQLALAQLRDNAVIDYTQLGLNFKLQAWEVLYNMASAQCQAGLWTKAANTLVEAISKWPEGAQDILDIAMDKVQKQVPLQLQQVPKGEVFQPPRRYLKHLEPMDFLGKAKVVASVIPDDHNAQPQQRSQAEHAGHQPSSSMCKRVLSTTGGHTSPGLYDSLLASRRPGPGPSEVSSGSEGAATKDPESLVTVTVQCHFTVPLKVPRGTGLSSFQTLLAQALLHQTQTGQLSYKAPGEERSWIPISTEESLQSIWRNVPVGPGGLQLQCQGVWGRPVLYQVVAQYNYRAQRPEDLDFHQGDTVDVLCEVDEAWLEGHRDGCVGIFPKCFVVPAGAYVEAMLVLGPQPGDQN

>rat-Rn-NOXA1: DDBJ^TM^ No. BR000299

MSSLGDQIRDWHRGVLAVAREDWDSALCFFSDVREPLAKMYFNMGCVHLMAGDPEAALRAFDQAVTKDTCMAVGFLQRGVANFQLQRLQEAVSDFQLALAQLRGNAAIDYTQLGLDFKLQAWEVLYNMASVQCQAGLWTKAANTLVEAISKRPEGAQDTLEAAMDKVQKQVPLQLRQVPKGEVFQPPRRYLKHLEPMDFLGKAKVVASVIPDDHNSDIQPQQSSQVEQAGLQSSSPVCKRVLSTRGGHMSPGLWDSLLATGGPVPGPSEDSSSAEGTATKDPESLVTVTVQCHFTVPLKVPRGTDLSSFRTLLSQALLQQTQKGQFSYKARGEDRAWVPISTEDSLQSVWRNVPVSPRGLQLQCRGAWGRPVLYQVVAQYDYRAQRPEDLDFRQGDTVDVLCEVDEAWLEGHRDGRVGIFPKCFVVPAATCVEALPVPEPQPGEQH

>chichen-Gg-NOXA1: Ensembl No. ENSGAL00000008952

MAYRELLRRWHQAALAADGGDWDAALETLCGIEEPPARICFNVGCMHLRAGRLRDALRAFDETVMKDNSLAVGYFQRGFVCLQLEMYEEALSDYHMAFSHLRKNPFIDYKQLGLRHILYAWEVLYSTAATQCRLQQWQEARDTLEKAVVWRPEGRSATLALALERVQNHQFLEPMQVPPGEFFRPRKKEVEQLDSKDFLGKPKVISSIIPNDEYIGFEPLRPQEMGAERGCRICPVFLSRDRESGYYRVLSHYYPEGTEKLAVKASSLVFVLARGANGWATAIHDGQKLHIPTSLLEPASKMDKWPSDSTEKIGDGIPLPPAQVPPSRLHMLPCHYGRTESPLTHREASSSTDRPAVLRVRCECTVVVRAGEVPSVPALRALLRERFGQQAERGRLSYRHLDGKELGAVSGEEDLEKMWQQLTDGRITLCCQDSDSHSGRPILYRMLAQHSYSAQGPGDLEFSKGDVLDILSEVNEDWLEGHCNGKTGIFPKCFATQTSCAAFP

>frog-Xt-NOXA1: GenBank^TM^ No. BC075351

MHYKEVVRRWHEGVVAAEGKDYDAALRSFTAIEDPPSRIWFNVGGIYLLRGDLPRALEAYDKSLAQDPCLAVGYYQRGYLQFKLGRYEKALSDCHLALSNLRNNSFIDYKQLGLRHVLFSWEAQYNMAAVLCSLGRWESAEEKLKETLQGDGRNAKLDWALDQVQRRSLLQPMSVPEGEFFRPRKQEVEQLNSVDFLGKPTVISSVVPNDQYSGFEPLRPQQPGFYEPCRDAMQCREAGYHRVVVHYYPENSNEVAVKANSVLFVLNKDGDWATAIHDGQKILIPTSFLEPTNPPKADIKKMNNGIPLPPMKTPPTRPNVRPGMEPLTGVQAGAPVPPQPAGGAAEPYKIKALPVGMEPIVEVAVPVQRSVPTHKETGNVPLGNDASLVEDAGRTIIKPKGESAPEPGPFPTRQTGPDNGPMATPVPTDDDKLVLSVHAEFTVNMTVSKAITYPELQGALREELRKHGEQMANHLSYRDPESRGLTPVTGSKDWQEVLKLSRANQVTLCCKETTLCAGRPVLYRMRAQYDYLAQGPADLSFQQGDLISILSEVNGEWLEGHCHRGIGIFPKCFAQRAEGI

>zebrafish-Dr-NOXA1: GenBank^TM^ No. XM_679087

MLYIELIRLWDEAVKAIDIRDWQGALSKLNQITDHNCRTMFVVASTHIALGQVDLAIKALDRVIAKDSCLAVGFFQRSAVHMMANRLEEALSDCIWAQKYMRENPVIDYKQLGLRYKLYSWQVLYNAAAVHSRLQQWDKARDILLAASQERGAGRSNLIDTALEAISRKDVLEPLLLPEGEVFRPRKLEVDQLKPRDFLGEAKVITSMIPNDDFRGFDPLRPQKPGYYEPKVEDGQDSRYMIMKSAYVAKGAGELTVPAGAEVFVYSDDDRDGLAVIIYDGKELANGIPHPPALKPPNRPQLPSQRSLELHPSSAGASHTTPPTTSSSTRIPPQQIQTSPQESGSVVVKVHYTYTMALRVPAETPFRDLQEKIAQKLGQPAMNIRLRHRRPGTRVLTPLNGDDGLDCLEGVAESGRAQIWCQNEDPLANRTILYQMVALYDYNAQGPEDLEFSEGDTIDILSEVNEEWLEGHVAGNIGIFPQSFAHRDTDSISGASTD

>medaka-Ol-NOXA1: Ensembl No. ENSORLP00000022190

MLYAELLKLWDESVQAMDSRDWQGALEKLQQIQEPTSRILFNAATAHLALGHLDMALKCLDLTISKDEHLAVGFFQRAAVNMQLERLEDALSDCIWAQKHMRKNTVIDYKQLGLRFKLYSWQVLHNAAAAYCRMGQWEQATEVLLQGGGAGHLEAALDSIERRELPTPLTVPESLVFRPRKQEVEQLQKKDFLGKAKVISSLIPNDDFGGFEPLRQQVSFSPQRPGFYEPKADRAQESRYMRVRTPYMARAPEQLTVPGGVVVFLFGEEDRDGMINVIYDGQRGLLPVFLLEPAEVNAPKAKNDNQRHRAVAFSRQSLLISASPSADLSAPLPLSKVEDEPEACASQKDEMVETCPPEEGSVVVKVHYTYTVAVTVPLNTPYHELKQRIAQKLGHPASELCLRHKQQGSHLLSPLSGEPGGTVQDLAVAGRATLWCQRFQMLVLSLMSGVRCLEEQKDDPLVNRHILYQMVALYNYDAQGPEDLEFSEGDTIDILSEVNEEWLEGHCAGNVGIFPSCFAYRENSSLYP

>amoeba-Dd-p67-like: GenBank^TM^ No. AY221172 (initially termed Dd-p67phox)

MLKQTIKKWNQSIERYESGNVGEALTILTSIEQSTSKINYNIGVMYIKSNNFRNAIEYFNRSVEQDKYLASSYYMRAIAHHMNGELNHAIVDYDETISKLRGHEYIDYKQLGLDHKLLLAEVLFNKALALGRAGSSVALQATQCFSQPSDSQEFKNQCKKIQDGSQLNFSTRPIPLSLLFKPPKVSDAPQKQRSATTSSIQSSSPSTPMSSSPPSYILKGPSSPPSSSSPSSSSPSLSSSSSPKLPPTPKPSFGSSPPPSSSSSSSSSSSSSSSSISPLTNKTLPPKPPPLPSKKLPSRPISCVIQDVKITLKVFYKDRRLIQIPVPCNLSTFIQKIELKFEITISDKFSLSFQLDGEENEINSQVQLDKMICMEINEINVKDIIPSPSPSPSPSPDKTNNSTSSYSSSSSSSSSSSSSSSSSSYDNKPKSSFIPKTTTRPILPPTTTTTTSTSNNFNRNATLPKKFGSTPSSPSFSSPSSSSSGGGGGPPIPTRGSPSISLLKQQNQTQSINIPPKVPTSSRPKMTQSHSPPSSSPLSSYSTSFQSVSSPSLSSSYNGSTSSYGGFSSSRPPPTPYPYQVLYTDSNEKYYLNTETNETFWELP

>fungus-Fg-NOXR: GenBank^TM^ No. FG04123.1

MSLKQEIETWVAALGRYDNNEFEEALNEFGKIGDTSKILFNMGVIHATLGEHEKAVESYQRAIRLDQYLAVAYFQQGVSNFLLGDFEEALANFNDTLLYLRGNAMIDYAQLGLLFKLYSCEVLFNRGLCYIYLQQMDAGMQDFSYAVKEKVVEDHNVIDDAINEQAEGYTVFSIPVGVVYRPNEAKVRNLKTKDYLGKARLVAASDRANAFTGFAGSEIKNAGKLEVKDDRPADNISFAATNLVKPGLSSRRQQSEPPNGRNVFPPTPPPENERPSRAASVRNQKPQLAKLNIQQAEPNRRYEKAASPADARRPMPRSASTTRTPLQREPPPLQLRPKQIPEETGSPEDVYAMYSATDGYRNSRGSAGSRRLRPQQYSEEEDASDYEGTINENDFEMIGQRRGPGSVSGSRNSRRTEVTKIRVKVHADEVKLIMITPDTRFETLSDKVRDKFNIKRRFKIKVKDDDMPNGDMITVGDQDDLEMVIDSVKDEARKQRTETGKMEIWILQL

>fungus-Mg-NOXR: GenBank^TM^ No. XP_359497

MSLKAELETWAAALKAYDEEDFEKSLDLFSRIADSSKILTNIGLIYATLGEHEAAVQRFIEATNFDQYLAVAYFQCGVSNFLLARYELAYKDFEEALLYLRGNQAINYEQLGLKFRLFSAEVLFNKGLAQIYMGRAQEGLADMEEARREKATDEHNVIDDAIQERGEGYTVFSIPVGVLYRPSEKKLKNSMQKDYMGKAKLVAASDPNDIFTTFTGSTRLKQGISPSGVFIDRPDIESAVIPSVTRSASVPSSTAPSRQPADGVRLAGVERSRTAMNPPQSARLASNKGPPPRPPMSAGASIGRSNTNITPSRPSPNATIGGPVRGLSVRRPGNASPGNSSPPRAPPKDDARLTEFYDDYLDSYGGDAPIPPIAQPGPDRIAAWARTNANPNYPLVRSGSRSAPTSQYTPSSYGGGGSLRRKVTKRNNPRAPSRVQSTYEEEEEGYVSGEYEDGPFELTLIRVKLHYQDDTRGMTLTPDTPFADFMDKVTAKFGKQINGLGLKFKDEDGGKVTLRDESDYELAIETARESAKGKPEGRLELWCMDL

>fungus-An-NOXR: GenBank^TM^ No.EAA58021

MSLKQEIETWVQALEHFDNQEYDLALRSFAAIADTSKILFNCGVIYATLGEHEKAVECYQGAVGLDQYLAIAYFQEGVSNFLLGDFEEALANFNDTLLYLRGNTYIDYEQLGLKFRLYSCEVLFNRGLCYIYLQQIGPGMQDLEYASKEKYSRSRLTPQGYTVFSIPVGVVYRPNEAKVKNLKTKDYLGKSRVIAANRLSTPADTSQRSVDSVPFATSHLVQKNLTSRSRQQSEPPMHRNLFPPTPPPDADKASLSSTGSNGTVRAQPGKAQRPPKLDLDRPGAQPAGRSTTDLTAPEKPRLGTIRTASEPRGQSRQPRGYAPERHVRSSDGYGHRRGASDHGFGVSNGHSDDAYGMYGEARAMTLANGGRPFQQQGYIDEEEEYGSSPCDEDLVPDASFELMGSRPRARSCSRGPARGYSRRPEVRRFRVKVHSFEDTRYILIPPTIEFAEFETRIREKFGFQMALKIKMQDEGDMITMVDQEDLDLLLMASREIARREGSEMGKMEIWVEERRMI

>human-Hs-p40*phox*: GenBank^TM^ No. NP_000622

MAVAQQLRAESDFEQLPDDVAISANIADIEEKRGFTSHFVFVIEVKTKGGSKYLIYRRYRQFHALQSKLEERFGPDSKSSALACTLPTLPAKVYVGVKQEIAEMRIPALNAYMKSLLSLPVWVLMDEDVRIFFYQSPYDSEQVPQALRRLRPRTRKVKSVSPQGNSVDRMAAPRAEALFDFTGNSKLELNFKAGDVIFLLSRINKDWLEGTVRGATGIFPLSFVKILKDFPEEDDPTNWLRCYYYEDTISTIKDIAVEEDLSSTPLLKDLLELTRREFQREDIALNYRDAEGDLVRLLSDEDVALMVRQARGLPSQKRLFPWKLHITQKDNYRVYNTMP

>dog-Cf-p40*phox*: GenBank^TM^ No. **XP_538398.1**

MALAQQLRAESDFEQLPDNIAISANIADIEEKRGFTSYFVFVIEVKTKGGSKYLIYRRYRQFHALQSKLEERFGPENKSNPFTCSLPTLPAKVYVGVKQEIAEMRIPALNAYMKSLLSLPIWVLMDEDVRIFFYQSPYDSEQVPQALRRLRPRTRKVKNESPQDAIFDRMAAPRAEALFDFTGNSKLELNFKAGDVIILLKGSSFFLPQGTVRGATGIFPQSFVKILKDFPEEEDPTNWLRCYYYEDTISTTKDIAVEEELSSTPLFKDLMQLMRREFQREDIALNYRDAQGDLVRLLSDEDVGLMVKQAQGLPSQKHLFPWKLHITQKDDYRVYNTVP

>mouse-Mm-p40*phox*: GenBank^TM^ No. NM_008677

MALAQQLRSESDFEQLPDDVAVSANIADIEEKRGFTSHFVFVIEVKTKGGSKYLIYRRYRQFYALQSKLEERFGPESKNSPFTCNLPTLPAKVYMGAKQEIAETRIPALNAYMKNLLSLPVCVLMDPDVRIFFYQSAYDAEQVPQALRRLRPRTRKIKGVSPQGAIMDRMEAPRAEALFDFTGNSKLELSFKAGDVIFLLSKINKDWLEGTSQGATGIFPGSFVKILKDFPEDEDTTNWLRCYFYEDTGKTIKDIAVEEDLSSTPLFKDLLALMRREFQREDIALSYQDAEGDLVRLLSDEDVGLMVKQARGLPSQKRLFPWKLHVTQKDNYSVYNTVP

>rat-Rn-p40*phox*: Ensembl No. ENSRNOP00000009544

MALAQQLRSESDFEQLPDDVAISANIADIEEKRGFTSHFVFVIEVKTKGGSKYLIYRRYRQFYALQSKLEERFGPESKNSPFTCSLPTLPAKVYMGVKQEIAETRIPALNAYMKNLLSLPVCVLMDPDVRIFFYQSAYDAEQVPQALRRLRPRTRKIKGVTPQGPSMDRMEAPRAEALFDFTGNSKLELSFKAGDVIFLLSKINKDWLEVRQRGRAEVFSSTLVFPLGASTEPGHRNHIGCPMFALEATVVTTTVIRGSVDHCPVFWKVLLSSMSREFQREDIALNYQDAEGDLVRLLSDEDVGLMVKQAQGLPSQKRLFPWKLHVTQKDNYGVYNTVP

>frog-Xt-p40*phox*: GenBank^TM^ No. NM_001030521

MSLPRQLRDESDFEQLPEDVPVFAHIADVEERRGFSLYYTFVIEVKTKGSSKYFIYRRYSQFFTLHAKLEENYGPDNGIAPYICTLPELPPKIFVGNKKDIAETRIPLLNGYMKGLLNSPTWLLLDEDLRMFYYQTLSDSEGIPRALRRLRPQTRKLKKDSQPISDVDRPRAEALFDFKGNAPMELNLKCGDLIYLLSWVNREWLEGTVGNRTGIFPASFVRIIKNLPELLYQVSLLRCYFHDHDRCLIRDISLEEDVGKCPSYKELLDLIRNQFPDAEVALNMRDKDGELIRLLDNSDMEFLITRGKRPPRAKNYFPWELHVTHEDDLEAYKTEA

>chicken-Gg-p40*phox*: GenBank^TM^ No. NM_001039272

MSLPRQLREKSDFDQFPDDVPVSANIADIEEKKGFTNYYMFVIEVKIKSGGRYLIFRRYREFYALHTKLEERYGGESKNSAFTCTLPVLPGKVYVGAKREIAENRIPILNIYMKNLLCLPVWVLMDEEVRLFFYHSNFDSEQVPRRLRRLRPRTRRVKSISSQLPVLDRVAAPRAEALFDFSGTSKLELSFKKGDLIYLLSRVNKDWLEGTVNDATGIFPSAFVKIIKDLPQQEDTVNKIRCYYYDETVSTIRDISVEENLSSIPLFKDLMELIKQEFDQHDIVLNYRDLDGDLIRLLSDQDVELMVSQSRKRSSEKHFFPWKLHITHKDDFSVYSTSPGIGDTQTVRAT

>zebrafish-Dr-p40*phox*: GenBank^TM^ No. CAK10863

MSLPQQLRDESDFDQLPDNIPVTATIADIEEKKGFIVYFSFVIEVKTKGNSKYLIYRRYREFFALHQSLELKYTAEAQSGYYNCQLPTLPGKVFMGNKKEIAESRIPELNNYMKRLLCLPTWVLLDDLIRMFFYQTESDSQQVPRALRRLRPPTRKVKTVKPKTDLLSAPRAEAVFDFSGSGRLELSLKAGDVIFLLRRVNADWLEGTVRDRTGIFPESFVKIIKALPENESDEEGGASRNSSRAQGSYSCLHCYFLQPEGIETRDICVQEDLSIQPSYEELLSRMRDVFHVDDIALNYRDAEGDLIRILDDEDVVLMVQESKRTESKVKRPVNQFPWELLVTHAKDLTVYNTEY

>fugu-Tr-p40*phox*: GenBank^TM^ No. **NP_001027719.1**

MSLQQLRDESDFDQLPHNVAVTATIADIEEKKGFIDYYRFVIEVKTKGGSKYFIYRRYREFFTLHQTLESKYSPEESDRQGQNTCPLPPLPGKVFLGNKREIAEGRIPELNTYMKRLLGLPAWFLLDDTLRMFFYQTDQDSQQQPRALRRLRPQTRKVKTIKPKMDLFSSPRAEVMFDFRGNGKEELNLKKGEVIFLLQRVNADWLEGTVNNQTGIFPQSFVKIIKPLPDSNTEGEGEGHTYSCLRCFLLTPSGVDTRDVCVEEELTTQPTYKDLLFHMRNVFKVNDIALNYRDMEGDLIRVVDDEDIQLMIKESRGQESKIKRPVNQFPWELYVTLASDFSVYNTEL

>tetraodon-Tn-p40*phox*: Ensembl No. GSTENG00034841001

SDFDQLPHNVPVTATIADIEEKKGFIDHYRFVIEVKTKGGSKYFIYRRYREFFTLHQNLESKYSPEDAEKPGPNTCLLPPLPGKIFIGNKKEIAEGRIPELNTYMKVISAGWALRMDGCRMARSDSPTTFHQSPTIPSPPRLLGLPVWFLLDDTLRMFFYQTDQDSQQQPRALRRLRPQTRKVKTVKPKMDLFSSPRAEVMFDFRGNGKEELNLKKGEVIFLLQRVNADWLEGTVNNQTGIFPQSFVKIIKPLPDVSAEGEDEGHTYSCLRCFLLSPAGVDTRDVCVEEDLTTQPTHKDLLTLMRNVFKVNDIALNYRDPEGDLIRVLDDEDIQLMVKESRDQQGKVKRPVNQFPWELHFASVTVSSPTRGDTSAVLSPLTPERRQMQVGVLLSVTQRTEERGIDFHFTNILGRETDTGVLRWDPQPALQEKLRRLLFSVPPASSLVCVKCGQADCAAGLLCGSGWLPATSSLPAHTGMQSQTTNSPFVSQHIYTVTHTRQGPAQVRPAATGTRPADKPGSSEEHAQPGLPELRLPH

>medaka-Ol-p40*phox*: GenBank^TM^ No. **Q15080**

MSQPQRLRDESDFDQLPGNIPVSATIADTEEKRGFIDYFRFVIEVKTKGGSKYLIYRRYKEFFNLHQTLEAKYSPGDLERPGPNTCVLPSLPGKIYIGNKQEIAESRIPELNTYMKRLLHLPAWLLLDETLRIFFYQTDEDSQIQPRALRRLRPPTRKVKTVEPQKMDLFSSPRAEAMFDFRGNSKAELNLKRGEVIFLLRRVNADWLEGTVNNQTGIFPESYVKIIKALPESDSESNGGGHTYSCLRCFLLTPSGVETRDVCVQEDLSIQPTYNELLSRMRNVFKVEDIALNYRDLEGDLIRILDNEDVQLMIEEGKRQKGKVKRPVNQFSWELHVTKAFDLSVYNFEA

>human-Hs-p22*phox*: GenBank^TM^ No. NM_000101

MGQIEWAMWANEQALASGLILITGGIVATAGRFTQWYFGAYSIVAGVFVCLLEYPRGKRKKGSTMERWGQKYMTAVVKLFGPFTRNYYVRAVLHLLLSVPAGFLLATILGTACLAIASGIYLLAAVRGEQWTPIEPKPRERPQIGGTIKQPPSNPPPRPPAEARKKPSEEEAAVAAGGPPGGPQVNPIPVTDEVV
>dog-Cf-p22*phox*: DDBJ^TM^ No. BR000286

MGQIEWAMWANEQALASGLILIMGGIVATAGQFTKWYFGAYSIGAGVFVCLLEYPRGKRRKGSTMERCGQKYMTKVVKVFGPLSRNYYIRAFLHLGLSVPAGFLLATILGTACLAIASSIYLLAAYHGEQWIPIEPQPKERPQVGGTIKQPPSNPPPRPPAEARKKPSEEEEAAGAVGVSGGPQENPVPVIDEVV

>mouse-Mm-p22*phox*: GenBank^TM^ No. NM_007806

MGQIEWAMWANEQALASGLILITGGIVATAGRFTQWYFGAYSIAAGVLICLLEYPRGKRKKGSTMERCGQKYLTSVVKLFGPLTRNYYVRAALHFLLSVPAGFLLATILGTVCLAIASVIYLLAAIRGEQWTPIEPKPKERPQVGGTIKQPPTNPPPRPPAEVRKKPSEGEEEAASAGGPQVNPMPVTDEVV

>rat-Rn-p22*phox*: GenBank^TM^ No. NM_024160.1

MGQIEWAMWANEQALASGLILITGGIVATAGRFTQWYFGAYSIVAGVLICLLEYPRGKRKKGSTMERCGQKYLTAVVKLFGPLTRNYYVRAVLHLLLSVPAGFLLATILGTVCLAIASVIYLLAAIRGEQWTPIEPKPKERPQVGGTIKQPPTNPPPRPPAEVRKKPSEAEEEAASAGGPQVNPIPVTDEVV

>chichen-Gg-p22*phox* (partial): GenBank^TM^ No. NW_001471438.1

MGQIEWAMWANEQALAAGL

>frog-Xt-p22*phox*: Ensembl No. **ENSXETG00000017431**

MGQIEWAMWANEQALASGLILLAGGIVAVAGQFKGWQFGAYGVAAGVFITLLEYPRSKRKKGSTMERCGQKYLAAVVKLFGPLTRNYYVRAILHAGLAVPGGFILSTILGTVCLGIASIIYFLAAIRGEEWRPIEKQAEPKPRAGETIKRPPENPPPRPPAEVRRKQADEVSVGGGHVNPIPVTDNV

>tetraodon-Tn-p22*phox*: DDBJ^TM^ BR000303

MGKIEWAMWANEQALASGFILLAGGIVGVAGRFRGWEFAAYAVAAGVFVCLLEYPRSKRSKGTSVERPGQHCFTVCVKAFGPVTKNYYVRAVLHAAICVPGGFMLATVLGCVCLGIASIIYLVAAIRGEHWEPILPKKEIQKPVAESIKNPPQNPPPRPPAETRRKRVDDLEAAAYDNP

>zebrafish-Dr-p22*phox*: GenBank^TM^ No. BC056702

MAKIEWAMWANEQALAAGLIYLTGGIVGVAGQFRGWQFAAFGIAAGVFVCLLEYPRSKRGKGTSIERSGQYCFTVCVKSFGPLTRNYYVRAFLHAALCVPGGFMLATVLGCVCLGMASLIYLSAPIHGEHWEPILHIETKKRLGESIKEPPQNPPPRPPPELRRKKADNLDAAAYDNPMSVTINE

>fugu-Tr-p22*phox*: GenBank^TM^ No. AB099895

MGKIEWAMWANEQALASGFILLTGGVVGVAGQFRGWQFAAYAVAAGVLVCLLEYPRSKRSKGTSVERPGQRCFTVCVKAFGPVTRNYYVRAVLHAAICVPGGFMLATVLGCVCLGIASIIYLVAAIRGEHWEPILPSKEIRKPVAESIKNPPQNPPPRPPADTRRKRVEDLEAASYDNPISVTANE

>medaka-Ol-p22*phox*: Ensembl No. ENSORLP00000011738

MGRIEWAMWANEQALAAGLILLAGGIVGVAGQFRGWEFASYAIAAGVFVCLLEYPRSKRAKGTSVERTGQYCFTVCVKAFGPVTRNYYVRAFLHAALCVPGGFMLATVLGCVCLGIASLIYLAAAVRGEHWEPILPRKDTRKPVAESIKSPPQNPPPRPPPEIRRKQAKDVEGAAYDNPMSVTDDE

>ascidian-Ci-p22*phox*: GenBank^TM^ No. AK114374

MPSSSSNIRSIQWGMWANETALLGSYVLTLGGIIGIVGGLLKNFMFWLPIGIYGVVFGILVGLLEYPRGKKNKGNTLLRSGQSCFSTMVNKLPFVSNYYFRAIAYFIVCIPGIISVPTFLGSVCVIVGSGIYLGAALHKERWNPIESRPQVPSTSNDITQPPSQPPPRLPQNKQI
