# Supplementary material for: Molecular evolution of Phox-related regulatory subunits for NADPH oxidase enzymes
Source: BMC Evol Biol. 2007 Sep 27;7:178. doi: 10.1186/1471-2148-7-178 (PMC2121648; doi:10.1186/1471-2148-7-178)
Supplement: Additional file 2 — Abbreviations of gene names in Figure 2. Names of the marker genes used in Figures 2 are provided. [file 1471-2148-7-178-S2.pdf]

Additional File 2

Abbreviation of gene names in Fig. 2.

[Fig. 2A]

SYNGR-3 (synaptogyrin 3), GFER (growth factor, augmenter of liver regeneration), TBL3 (transducin beta-like 3), RPS2 (40S ribosomal protein S2), RP3L (60S ribosomal protein L3-like), TEX2 (testis expressed 2), HS3ST6 (heparan sulfate 3-O-sulfotransferase 6), TOM1L2 (Target of Myb-like protein 2), C17ORF39 (chromosome 17 open reading frame 39).

[Fig. 2B]

FLJ20433 (hypothetical protein FLJ20433), ENTPD8 (ectonucleoside triphosphate diphosphohydrolase 8), NELF (nasal embryonic LHRH factor), PNPLA7 (patatin-like phospholipase domain containing 7), WDR85 (WD repeat domain 85), ARRDC-1 (arrestin domain containing 1), MAN1B1 (mannosidase-alpha class 1B, member 1), DPP7 (Dipeptidyl peptidase 7), STXBP-1 (Syntaxin-binding protein 1), DFNB31 (deafness, autosomal recessive 31), MRPL41 (mitochondrial ribosomal protein L41).
