# Supplementary material for: Molecular evolution of Phox-related regulatory subunits for NADPH oxidase enzymes
Source: BMC Evol Biol. 2007 Sep 27;7:178. doi: 10.1186/1471-2148-7-178 (PMC2121648; doi:10.1186/1471-2148-7-178)
Supplement: Additional file 3 — Alignment of characterized domains of Nox regulatory subunits. Alignments of (i) PX and tandem SH3 domains of Nox organizers of deuterostomes, (ii) TPR and activation domains of Nox activators of deuterostomes and fungi (A. nidulans, M. grisea, F. graminearum) and amoeba, (iii) the N-terminal SH3 domain of vertebrate p67phox, (iv) PB1 domain of p67phox and NOXA1 of vertebrates and NOXR of fungi (A. nidulans, M. grisea, F. graminearum) and amoeba, (v) the C-terminal SH3 domain of vertebrate p67phox, vertebrate NOXA1 and S. purpuratus p67phox-like protein, (vi) PX domain of vertebrate p40phox, (vii) Proline (P)-basic, SH3 and PB1 domain of vertebrate p40phox. [file 1471-2148-7-178-S3.pdf]

Additional File 3

Alignment of characterized domains of Nox regulatory subunits.

We used the following naming: Hs (*H. sapiens*), Cf (*C. familliaris*), Rn (*R. norvegicus*), Mm (*M. musculus*), Gg (*G. gallus*), Xt (*X. tropicalis*), Dr (*D. rerio*), Tr (*T. rubripes*), Tn (*T. nigroviridis*), Ol (*O. latipes*), Ci (*C. intestinalis*), Sp (*S. purpuratus*), Dd (*D. discoideum*), An (*A. nidulans*), Mg (*M. grisea*), Fg (*F. graminearum*).

PX and tandem SH3 domains of Nox organizers (vertebrate p47*phox*, vertebrate NOXO1, *C. intestinalis* and *S. purpuratus* p47*phox*-like proteins).

Gray boxes indicate the amino acid sequences corresponding to the PX (residues 3-132), bis-SH3 (residues 151-285) domains of human p47*phox*.

```
                                PX-domain
Hs-p47phox      -------MGDTFIRHIALLGFEKRFVPSQHY--------------VYMFLVKWQDLSEKV 39
Cf-p47phox      -------MGDTFIRHIALLGFEKRFVPSQHY--------------VYMFLVKWHDLSEKV 39
Mm-p47phox      -------MGDTFIRHIALLGFEKRFIPSQHY--------------VYMFLVKWQDLSEKV 39
Rn-p47phox      -------MGDTFIRHIALLGFEKRFVPSQHY--------------VYMFLVKWQDLSEKV 39
Gg-p47phox      -------VGDTFIRHIELLRYEKRFFPSQHY--------------VYMFLVKWNDLSEKL 39
Xt-p47phox      -------MTEPHIRHIQLLGFEKRFIPSQHY--------------VYMFMVKWQDLTEKL 39
Tn-p47phox      -------MADTYVRHVQLLGFEKRFFPSQHY--------------VYMLLVKWSDLTEKL 39
Tr-p47phox      -------MAETYVRHVQLLGFEKRFFPSQHY--------------VYMLLVKWSDLTEKL 39
Ol-p47phox      -------MEDVYVRHVELLGIEKRFFPTQHF--------------VYMLMVKWSDQSEKL 39
Dr-p47phox      -------MAETYVRHVELLGFEKRFFPSQHY--------------VYMLLVKWSDQSEKL 39
Ci-p47phox      -------MVNRTLKSVKVIDIEKRRLPSKHY--------------VYLIEIKWSDGSLCT 39
Sp-p47phox      -------MGKRTVVNANVTDIEKRREPTKHY--------------VYIIHVTWSDGSVNV 39
Dr-NOXO1        -------LIKLFIRRVEHRNMDINIHSSPPQ--------------LYMTTVLWSDGNEIT 39
Ol-NOXO1        ------PADTHMSLSVSAPGSDKWLLPSDDRKY------------TFMVSGIWSDGSEII 42
Tn-NOXO1        METQRYPISARLVGVLHKEKSKVGAHKSQVVLFHARFNQQLCPLQMYMTSVLWSDHNEIV 60
Gg-NOXO1        ----------------------------------------------YMMFVSWSDQNNIL 14
Xt-NOXO1        ----------------------------------------------------WSDHNEIL 8
Hs-NOXO1        MAGPRYPVSVQGAALVQIKRLQT-----------------------FAFSVRWSDGSDTF 37
Cf-NOXO1        MAGSRHPVSVRAAALVQTGRLQT-----------------------FAFSVCWSDGSDTF 37
Mm-NOXO1        MASPRHPVSAHAVALVQMDRLQT-----------------------FAFSVCWSDNSDTF 37
```

```
Rn-NOXO1            MASPRHPVSAHAVALVQMERLQT-----------------------FAFSVCWSDNSDTF 37
                                                                    * *

Hs-p47phox          VYRRFTEIYEFHKTLKEMFPIEAGAINPENRIIPHLPAPK-----WFDGQRA---AENRQ 91
Cf-p47phox          VYRRFTEIYEFHKMLKEMFPIEAGDINPENRIIPHLPAPR-----WFDGQRA---AESRQ 91
Mm-p47phox          VYRKFTEIYEFHKMLKEMFPIEAGEIHTENRVIPHLPAPR-----WFDGQRA---AESRQ 91
Rn-p47phox          VYRKFTEIYEFHKMLKEMFPIEAGEIHTENRVIPHLPAPR-----WYDGQRA---AESRQ 91
Gg-p47phox          IYRRFTDIYEFHKALKEMFPIESGDINAENRIIPHLPAPK-----WFDGQRS---TQSRQ 91
Xt-p47phox          VYRKFTEIYEFHKSLKEMFPIEAGDISKEHRTIPHLPAPK-----WFDGLRS---TENRQ 91
Tn-p47phox          IYRTYPEIYTFHKALKEMFPIEAGKIEKRDRIIPSLSAPP-----WLDSQKS---TETRQ 91
Tr-p47phox          IYRTYPEIYTFHKSLKEMFPIEAGKIEKRDRIIPSLSAPP-----WLDSQKS---TETRQ 91
Ol-p47phox          IYRSYPEIHTFHKSLKDMFPIEAGQIEAKDRIIPKLPAPR-----WLESQKS---RENRK 91
Dr-p47phox          VYRRYPEVHTLHKTLKEMFPIEAGDIDEKDRIIPTLPAPK-----WLDNQKT---TETRQ 91
Ci-p47phox          VGRRFSAFFMMHMTLLEKFPLEGGQKDPSRRILPFLPGKI-----LFKRSHTRDVTLKRL 94
Sp-p47phox          VYRRYSTFFDFQNKLLSKFPEEAGANNPSSRCIPFLPGKK-----LFGRSHIREVALKRL 94
Dr-NOXO1            VYRSLEDFKKMHRQLKKKFPPSN-PFKRSARIVPEFKGN---------KWSGSK-SVLRM 88
Ol-NOXO1            IYRSFKDFKKFHDQLKKQFPNLT-PFRKEDRMLPKFNGKAR----RSLKQKGSKKSVKQM 97
Tn-NOXO1            VYRTFQDFRKMH-KLKRSGKKKS-PTR----------------------------SLVRL 90
Gg-NOXO1            IYRTLEEFKRFHKELKRKFPIESGSLRRSDRTIPRFKDING----KQKKSGKINRSLERL 70
Xt-NOXO1            IYRTFEDFKKLNRQLKKKFPLEAGLFRKSDNLLPKLKDVP-----IFRKNRTTNRFIERL 63
Hs-NOXO1            VRRSWDEFRQLKKTLKETFPVEAGLLRRSDRVLPKLL-----DAPLLGRVGRTSRGLARL 92
Cf-NOXO1            VRRSWAEFKELHKTLKEAFPVEAGLLRRSDRILPKLP-----DTSLLVRGGRTGRGLARL 92
Mm-NOXO1            VRRSWDEFRQLQKTLKKTFPVEAGLLRRSEQVLPKLP-----DAPLLTRRGHTGRGLVRL 92
Rn-NOXO1            ARRSWEEFRQLQKTLKKIFPVEAGLLQRSERVLPKLPGQACRNAPLLTRRGHTGRGLLRL 97
                      *           *   **            *                        *

Hs-p47phox          GTLTEYCGTLMSLPTKISRCPHLLDFFKVRPDDLKLPTD--NQTKKPETYLMPKD-GKS- 147
Cf-p47phox          GTLTEYYNTLMGLPVKISRCPQLLDFFRVRPDDLKLPTD--SQVKKPETYLVPKD-GKS- 147
Mm-p47phox          GTLTEYFNGLMGLPVKISRCPHLLDFFKVRPDDLKLPTD--SQAKKPETYLVPKD-GKN- 147
Rn-p47phox          GTLTEYFNSLMGLPMKISRCPHLLNFFKVRPDDLKLPND--SQVKKPETYLTAKD-GKN- 147
Gg-p47phox          GTLAEYCYTLVNLPHKISRCRHVVSFFEVRPDDMNPVTD--SQIRKPEVFLLPKD-AKK- 147
Xt-p47phox          VTLSDYFSSLLSLPPKISRCPHVLNFFQVRSDDVNPVANN-TNGRKPETFLLKVDTAKK- 149
Tn-p47phox          TSLSDYCQALVNLPPHISRCTHLTSLFKVRPEDENPAAP--NTLKRNETFVVSRDLARG- 148
Tr-p47phox          TTLSDYCHSLVNLPPHISRCTHLTGFFTVRPEDENPPSP--NILKRNETFVVSKDLARG- 148
Ol-p47phox          TTLVDYCHLLVSLPPHISRCKELSNFFKVRPEDENPPAP--NITKRNQTFVVSKEPAQG- 148
Dr-p47phox          ATLAEYCRSLLNLPANISRCQLIRDFFKMRPEDETPPAP--HPYKRNETFIMSTNRVRSN 149
Ci-p47phox          GSISEYCESLLLLPEHISQCDTILRFFETSSSDIARDTK----------EKT-QSTAVSQ 143
Sp-p47phox          SPIDEYCTALVKLPGKISDSKEVINFFTPTPEDVSPPSP----------DGGGESTRGRA 144
Dr-NOXO1            KALEEYCGQLLKSDAQVCRSSELIQFLLPKAHDLNADFAKNCIVIMPSDVTLGSSKA--E 146
Ol-NOXO1            EFLESYCDKLLKCDPNVTQSSEVTRFFTPKDQDLQPDFTKNSSLLLVYGR-PGALRS--S 154
Tn-NOXO1            KFLQKYCNELLSCEPRVSQSADLIQFFHPNAQDLEPEFSKNRQEEVKAEAGHGSVG---- 146
Gg-NOXO1            KLLETYTQELLKVDAKISQGEDVIQFFKAQTQDLDPCFPEDSVVIMPSEIGGEKKKEVQQ 130
Xt-NOXO1            RLLEKYSQELLRTDGKISQCDLVLKFFTPSNNDLNPKFPENSLVMMTSDSKDQKEQKKPL 123
Hs-NOXO1            QLLETYSRRLLATAERVARSPTITGFFAPQPLDLEPALPPGSRVILPTPEEQPLSRA--- 149
Cf-NOXO1            RLLDTYTRALLAAAEQLSRSAVLTGFFEPQPVDLEPVLPPGSLVILPTPEE-PHRRP--- 148
Mm-NOXO1            RLLDTYVQALLATSEHILRSSALHGFFVPKPLDLEPMLPPGSLVILPTPEE-PLSQP--- 148
Rn-NOXO1            RLLETYVRSLLATSQHIVTSSTLNSFFAPKPLDLEPMLPPGSLVILPTPEE-PLSQP--- 153
                         *   *                      *

                       bis-SH3 domains
Hs-p47phox          TATDITGPIILQTYRAIANYEKTSGSE--MALSTGDVVEVVEKSESGWWFCQMKAKR-GW 204
```

```
Cf-p47phox      SVTDITGPIILQTYRAIADFEKTSSSQ--MALATGDVVDVVEKSESGWWFCQTKTKR-GW 204
Mm-p47phox      NVADITGPIILQTYRAIADYEKSSGTE--MTVATGDVVDVVEKSESGWWFCQMKTKR-GW 204
Rn-p47phox      NVADITGPIILQTYRAIADYEKGSKTE--MTVATGDVVDVVEKSESGWWFCQMKTKR-GW 204
Gg-p47phox      NTSDITGPIVLQTYRAIADYEKSSKSE--MAVKAGDAVDVVEKSETGWWFCQLKTKR-GW 204
Xt-p47phox      NVSDITGPIILQSYRVIADYEKNSKSE--LAAKNGDVVEIVEKSENGWWFCQLRNKR-GW 206
Tn-p47phox      NASEISGPIILDMYRAIADYTKTTKYE--INLLAGDQVEIVEKNQNGWWFCQMDSKR-GW 205
Tr-p47phox      NVSEISGPIILDMYRAIADYTKTTKYE--INLHAGDQVEIVEKNQNGWWFCQCDSKR-GW 205
Ol-p47phox      TAAEISGPIILDTYRVIADFEKTSKHE--LNLHDGDLVEILEKNSNGWWFCQCEAKR-GW 205
Dr-p47phox      TTSEITGPIILETYRVIADYSKSSKYE--LTLKMGDMVDIVEKSPNGWWFCQCESRR-GW 206
Ci-p47phox      IIQDITGPIELETYIAIADYKAEAKTQ--ISLHSGETVEVVEKSESGWWLVCNTYGSNGW 201
Sp-p47phox      DIGNISEPIQAEQYIVVADYKKQQKNE--VELTAGDLVEVFEKNDNGWWFVT-VHDQHGW 201
Dr-NOXO1        SNSGVTQPFVTETYRCIANYETKDTKNRPFKVEVDETVDVLIKDQKGWWLVENESKHLAW 206
Ol-NOXO1        GAGNVTHPFVTQTYRCVAPYETRDTKNRPFKVAVDEKLDVLIKDPAGWWLVESENKRLAW 214
Tn-NOXO1        ---NVTQPFVTVTYRCVSQYETKDTKNKPFKVAADEKVDVLIKDKAGWWLVENEEKRMAW 203
Gg-NOXO1        QQLSITYPQVSQSYRCIETFETKDTKNKTFKVAKKEIVEVLLKDMTGWWLVENADKQIAW 190
Xt-NOXO1        PEAPAIHPIVSQQYICMEDYETKDTKNRPFKVKRHELVGVLIKENTGWWLVENEEKHLAW 183
Hs-NOXO1        AGRLSIHSLEAQSLRCLQPFCTQDTRDRPFQAQAQESLDVLLRHPSGWWLVENEDRQTAW 209
Cf-NOXO1        PHSPAICSLEAQSLRCLQPFSTQDTQGWPFHARAQEVIDVLLRHPSGWWLVANEEQQMAW 208
Mm-NOXO1        RGSLDIHSLEAQSIPCVQPFHTLDIRDRPFHTKAQEILDILLRHPSGWWLVENKDQQVAW 208
Rn-NOXO1        IGSLAIHSLEAQSMRCLQPFHTLDTKDRPFHTQAKEILDILLRHPSGWWLVENKDQQTAW 213
                                   *               *      *   ***          *

Hs-p47phox      IPASFLEPLD-SPDETEDPEPNYAG---EPYVAIKAYTAVEGDEVSLLEGEAVEVIHKLL 260
Cf-p47phox      VPASYLEPLD-SPDEAEDPEPNYEG---EPYVTIKAYTAEMEDEMSLQEGEAIEVIHKLL 260
Mm-p47phox      VPASYLEPLD-SPDEAEDPDPNYAG---EPYVTIKAYAAVEEDEMSLSEGEAIEVIHKLL 260
Rn-p47phox      VPASYLEPLD-SPDEAEDPDPNYAG---EPYVTIKAYAAVEEDEVSLSEGEAIEVIHKLL 260
Gg-p47phox      VPAAYLEPMD-GPDESEEQEPNYAG---ELYVVQKSYTAVEEDELTLKEGDTIEVIHKLL 260
Xt-p47phox      MPAAYLEPLD-GPDESEEQDPNYEG---DLHITTKDYSGELDDELSLQEGENVEVIHKLL 262
Tn-p47phox      VPASYLEPLD-GPEESEEADPDYEGS--ELFITIKAYKAEQEDEISLDLGESIEVIHKLL 262
Tr-p47phox      VPASYLEPLD-GPEESEEAEPDYGGSPCELYITIKAYKAEQEDEITLDLGESIEVIHKLL 264
Ol-p47phox      VPASYLEPLD-GPEEAEEAEPDYEG---ELHVTTNAYKAEQDDEISLDLGETVEVIHKLL 261
Dr-p47phox      VPASYLEPLD-GADESEEPEPNYAG---ELYKTTRGYKAVEQDEMTLEAGVIIEVIHKLL 262
Ci-p47phox      VPGAYLEKED-GSEEDLVTEKAAVGQG-TWYVATSHYDATSNDEISFPMGAALEVLQVNL 259
Sp-p47phox      APGTFLQNPD-GQEEEDE-ETLIPGND-ESYITNNAYQGQAEDEISFETGVVVTVIQKSL 258
Dr-NOXO1        FPAPYLERAEMADDGPDEMDNESFQSAGVFYVATKAYKATNSDELSVELGSVLEVLQKSD 266
Ol-NOXO1        FPAPYLE----VLDGEDEDDEGNLG--GSLYCAVRSYSTKKNDEVPLSIGSVVEVLRKSD 268
Tn-NOXO1        FPAPYLEK---LEEDGDEDDTDGTR---TLYLTAKNYKASKGDEISVAVGAVVEVLQKSD 257
Gg-NOXO1        FPASYLEQ---ISAHKDIQNVESSDEEGSLYFVMRAYEAQKADELSLNKGVVVEVVRRSD 247
Xt-NOXO1        FPAPYLKD---VDNSEDTDSG-TSEDEGVLYYAAKAYEAMNSDEVSITVGVLVEVIEKSN 239
Hs-NOXO1        FPAPYLEE---AAPGQGREGGPSLGSSGPQFCASRAYESSRADELSVPAGARVRVLETSD 266
Cf-NOXO1        FPAPYLEE---AAP--DREG-TTLRSSGSQFCASQAYESSHADELSVPAGARVSVLETSD 262
Mm-NOXO1        FPAPYLEE---VATCQGQESGLALQGSGRQFCTTQAYEGSRSDELSVPSGARVHVLETSD 265
Rn-NOXO1        FPAPYLEE---IATGQGQESGMAVQGSGRQFCATQAYEGSRPDELSVPSGARVHVLETSD 270
                 *  **                              *     **     *    *

Hs-p47phox      DGWWVIRKDDVTGYFPSMYLQKSGQ 285
Cf-p47phox      DGWWVVRKDDITGYFPSMYLQKSGQ 285
Mm-p47phox      DGWWVVRKGDITGYFPSMYLQKAGE 285
Rn-p47phox      DGWWVVRKGDITGYFPSMYLQKAGE 285
Gg-p47phox      DGWWVIRKDETTGYYPSMYLQKSGE 285
Xt-p47phox      DGWWVVRKGSITGYFPAMYLQKSGE 287
Tn-p47phox      DGWWVVRKGEGTGYFPSMFLQKASK 287
```

```
Tr-p47phox          DGWWVVRKGEQMGYFPSMFLQKANK 289
Ol-p47phox          DGWWVVRKGDETGHFPSMFLTKASK 286
Dr-p47phox          DGWWVVRKGEETGFYPSMFLCRTGE 287
Ci-p47phox          EGWWLARYNSNEGWVPGSYLEKSRR 284
Sp-p47phox          DGWWKVSYQGKQGWAPATFLQIYKG 283
Dr-NOXO1            NGWWIVRYNRKAGYVPSMYLQPHNN 291
Ol-NOXO1            DGWWLIRFNGKVGYIPAMYLQPYNN 293
Tn-NOXO1            SGWWLIRYQGKVGYVPTLCLQPYNR 282
Gg-NOXO1            NGWWLIRYNGRKGYMPSMCLQAYKN 272
Xt-NOXO1            NGWWLIR------------------ 246
Hs-NOXO1            RGWWLCRYGDRAGLLPAVLLRPEGL 291
Cf-NOXO1            RGWWLCRFRGRSGLLPAVLLQPEGL 287
Mm-NOXO1            RGWWLCRYNGRTGLLPAMSLQPEGL 290
Rn-NOXO1            RGWWLCRYNGQTGLLPAVLLQPEGL 295
                     ***  *     *  *   *
```

TPR and activation domains of Nox Activators (vertebrate p67*phox*, vertebrate NOXA1, *C. intestinalis* and *S. purpuratus* p67*phox*-like proteins, Dd-p67-like, and fungal NOXR).

Gray boxes indicate the amino acid residues corresponding to the tetratricopeptide repeat (TPR, residues 6-153), activation domain (AD, 196-210 residues) of human p67*phox*.

```
                           TPR region
Hs-p67phox          ---MSLVEAISLWNEGVLAADKKDWKGALDAFSAVQDPHSRICFNIGCMYTILKNMTEAE 57
Cf-p67phox          ---MSLAEAISLWNEGVLAADKKDWKGALEAFAAVQDPHSRICFNVGCMHTILGNMPEAE 57
Mm-p67phox          ---MSLAEAIRLWNEGVLAADKKDWKGALEAFSEVQDPHSRICFNIGCVNTILENLQAAE 57
Rn-p67phox          ---MSLAEAIRLWNEGVQAADKKDWKGALEAFSEVQDPHSRICFNIGCMYTILDNLQEAE 57
Gg-p67phox          ---MSLVETIRLWQEGVCAADGKEWGAALKAFTAVQNPPAKICFNIGCTHLVLGQLAEAE 57
Xt-p67phox          ---MALVEIMRLWSEGVAAAENEDWNGALKSFTSITDPRSKICFNIGCCHLVLGDLEKAE 57
Tn-p67phox          ---MSFLDTLRQWDNACTVADGQDFSAALQVFLSIQEPNSKICFNIGCLHLLNEDLSAAE 57
Tr-p67phox          ---MSFLDTLKQWDKASTVADRQEFSEALEIFLSIEEPNSKIYFNIGCLHLLNEDLKDAE 57
Ol-p67phox          ---MSFVDTLRQWDDGVTCADKQDFSEALRILLAIPEPNSKICFNIGCLHLLNQNLDDAE 57
Dr-p67phox          ---MSFVSTLRQWDEAVACVEQRDPDAALRIFLSIEEKNSKIAFNIGCLCLNNSDLDEAE 57
Hs-NOXA1            --MASLGDLVRAWHLGAQAVDRGDWARALHLFSGVPAPPARLCFNAGCVHLLAGDPEAAL 58
Cf-NOXA1            --MPSLGDLVHDWHRGVQAVARGDWGCALRLFSGDPDPPAKMCFNLGCVHLLAGDPEAAL 58
Mm-NOXA1            --MSSLGDQIRDWHRGVLAVAREDWDSALCFFSDVREPLARMYFNRGCVHLMAGDPEAAL 58
Rn-NOXA1            --MSSLGDQIRDWHRGVLAVAREDWDSALCFFSDVREPLAKMYFNMGCVHLMAGDPEAAL 58
Gg-NOXA1            ---MAYRELLRRWHQAALAADGGDWDAALETLCGIEEPPARICFNVGCMHLRAGRLRDAL 57
Dr-NOXA1            ---MLYIELIRLWDEAVKAIDIRDWQGALSKLNQITDHNCRTMFVVASTHIALGQVDLAI 57
Ol-NOXA1            ---MLYAELLKLWDESVQAMDSRDWQGALEKLQQIQEPTSRILFNAATAHLALGHLDMAL 57
```

```
Xt-NOXA1                ---MHYKEVVRRWHEGVVAAEGKDYDAALRSFTAIEDPPSRIWFNVGGIYLLRGDLPRAL 57
Ci-p67phox              ---MASKHAALLQQEGVEASEENDWFKALSIFQQVPNPSSVIWFNIGCCHLQVQQYLKAE 57
Sp-p67phox-N            MDKQTHKDIIQVWHEGVQTFDKGNVDAALQAFLGIGDPSAKILFNIGHLEMSQYRFGEGE 60
Sp-p67phox-C            ---MTHKDTIATWYEGVLAFERGESSEALNQLNSIVDPSAKILYNIGILQKSLGGQLEDAN 316
Fg-NOXR                 ---MSLKQEIETWVAALGRYDNNEFEEALNEFGKIGD-TSKILFNMGVIHATLGEHEKAV 56
An-NOXR                 ---MSLKQEIETWVQALEHFDNQEYDLALRSFAAIAD-TSKILFNCGVIYATLGEHEKAV 56
Mg-NOXR                 ---MSLKAELETWAAALKAYDEEDFEKSLDLFSRIAD-SSKILTNIGLIYATLGEHEAAV 56
Dd-p67-like             ----MLKQTIKKWNQSIERYESGNVGEALTILTSIEQSTSKINYNIGVMYIKSNNFRNAI 56
                                                   **              *              *

Hs-p67phox              KAFTRSINRDKHLAVAYFQRGMLYYQTEKYDLAIKDLKEALIQLRGNQLIDYKILGLQFK 117
Cf-p67phox              KAFTRSINKDKHLAVAYFQRGMLYYHMEKYDAAIKDLKEALTQLRGNQLIDYKILGLQFK 117
Mm-p67phox              QAFTKSINRDKHSAVAYFQRGMLYYRMEKYDLAIKDLKEALTQLRGNQLIDYKILGLQFK 117
Rn-p67phox              QAFTKSINRDKHLAVAYFQRGMLYYSMEKYLLCVYCLREALVTFRKKQVLSPKPEGLQFL 117
Gg-p67phox              EAFTQSISCDKHLAVAYFQRGTVFYKRHNHEMALKDFKEALAQLRGNQLIDYKILGLRYR 117
Xt-p67phox              KAFTLTIERDMHLAVGYFQRGFVFFQRGKYSLALQDWTRAYTEMRGNQLIDYKILGLIFK 117
Tn-p67phox              KAFDSSISKDEHLAVAFFQRAITFYKMSRQTHRPCHFQQTFKELRGNQLIDYGALGLRYK 117
Tr-p67phox              KAFDSSICKDEHLAVAFFQRAITFYKMTRQEYSLADFQQTLKELRGNQLIDYGALGLRYK 117
Ol-p67phox              KAFDCSIRKDEHLAVAFFQRGITFYKKMRYEESIGDFQRAFKTLRGNNLIDYKALGLRYI 117
Dr-p67phox              KAFDGSIGKDEHLAVAFFQRGVTFYKKEKFEESLLDFQQAFKQLRGNQLIDYTPLGLRYK 117
Hs-NOXA1                RAFDQAVTKDTCMAVGFFQRGVANFQLARFQEALSDFWLALEQLRGHAAIDYTQLGLRFK 118
Cf-NOXA1                RAFDQAVTKDTCMAVGFFQRGVANFQLERFQEALSDFRLALAQLRGNAAIDYTQLGLRFK 118
Mm-NOXA1                RAFDQAVTKDTCMAVGFLQRGVANFQLQRFQEAVSDFQLALAQLRDNAVIDYTQLGLNFK 118
Rn-NOXA1                RAFDQAVTKDTCMAVGFLQRGVANFQLQRLQEAVSDFQLALAQLRGNAAIDYTQLGLDFK 118
Gg-NOXA1                RAFDETVMKDNSLAVGYFQRGFVCLQLEMYEEALSDYHMAFSHLRKNPFIDYKQLGLRHI 117
Dr-NOXA1                KALDRVIAKDSCLAVGFFQRSAVHMMANRLEEALSDCIWAQKYMRENPVIDYKQLGLRYK 117
Ol-NOXA1                KCLDLTISKDEHLAVGFFQRAAVNMQLERLEDALSDCIWAQKHMRKNTVIDYKQLGLRFK 117
Xt-NOXA1                EAYDKSLAQDPCLAVGYYQRGYLQFKLGRYEKALSDCHLALSNLRNNSFIDYKQLGLRHV 117
Ci-p67phox              NAFSQSIAKDKYLVAGYFQLAVSQTHLGNYAEAIDNFSSALSSLRGNPFIDYKQLNMLCK 117
Sp-p67phox-N            VNFTAAVEKDPHMALAHFQLGMVYFHLHRYNESRNSYEKAKVCLRGNRFIDYRQLGFVHK 120
Sp-p67phox-C            SILKEVVSRDPHLAIGHFQSGVVLGLLGRGDDAWHAFEKARETLRG-KMINYKPLGLQYK 375
Fg-NOXR                 ESYQRAIRLDQYLAVAYFQQGVSNFLLGDFEEALANFNDTLLYLRGNAMIDYAQLGLLFK 116
An-NOXR                 ECYQGAVGLDQYLAIAYFQEGVSNFLLGDFEEALANFNDTLLYLRGNTYIDYEQLGLKFR 116
Mg-NOXR                 QRFIEATNFDQYLAVAYFQCGVSNFLLARYELAYKDFEEALLYLRGNQAINYEQLGLKFR 116
Dd-p67-like             EYFNRSVEQDKYLASSYYMRAIAHHMNGELNHAIVDYDETISKLRGHEYIDYKQLGLDHK 116
                          *      *   *  *                           *    **

Hs-p67phox              LFACEVLYNIAFMYAKKEE-WKKAEEQLALATSMKSEPRH-SKIDKAMECVWKQ------ 169
Cf-p67phox              LFACEVLYNIAFMYAKNEE-WKKAEEHLALAMSMKSEPRH-TKIDKAMECVWKQ------ 169
Mm-p67phox              LFACEVLYNIALMHAKKEE-WKKAEEQLALATNMKSEPRH-SKIDKAMESIWKQ------ 169
Rn-p67phox              LQRSPVLYNIALMHAKKEE-WKKAEEQLALATNMKSEPRH-SKIDKAMESIWVS------ 169
Gg-p67phox              LFACEILYNIALVYATMEN-WKKAEEHLTLAMSSKSEPQH-NKIDRAMEAILKQ------ 169
Xt-p67phox              LYSCEILHNIALTHAKEGK-WAKAEESILLALSQKVELRHNTKLEKAMEDILKE------ 170
Tn-p67phox              LTACEVLHNMALAEAQLGR-WEKAQESLVKALDYSSDSKL-GAIDKALQATLKQ------ 169
Tr-p67phox              LNACEVLHNIALTEAQMGH-WEKAQESLVKALDYRTESKL-GIIDNALQATLKQ------ 169
Ol-p67phox              LYACEVLHNMALAEAQLGN-WEKAEQNLRKALDYKTDAKL-SVIEKAQQAVLKE------ 169
Dr-p67phox              LYACEVLHNIGLAQAQLGK-WEKAQENLLTALSLRADAKF-SHIDHALDAILKH------ 169
Hs-NOXA1                LQAWEVLHNVASAQCQLGL-WTEAASSLREAMSKWPEGSL-NGLDSALDQVQRR------ 170
Cf-NOXA1                LKTWEVLFNVGAAQCALGL-WAEAAGSLEEALCKGPEGAG-EDLHAALAQVQKQ------ 170
Mm-NOXA1                LQAWEVLYNMASAQCQAGL-WTKAANTLVEAISKWPEGAQ-DILDIAMDKVQKQ------ 170
rat-NOXA1               LQAWEVLYNMASVQCQAGL-WTKAANTLVEAISKRPEGAQ-DTLEAAMDKVQKQ------ 170
Gg-NOXA1                LYAWEVLYSTAATQCRLQQ-WQEARDTLEKAVVWRPEGRS-ATLALALERVQNH------ 169
```

```
Dr-NOXA1            LYSWQVLYNAAAVHSRLQQ-WDKARDILLAASQERGAGRS-NLIDTALEAISRK------ 169
Ol-NOXA1            LYSWQVLHNAAAAYCRMGQ-WEQATEVLLQGG---GAG----HLEAALDSIERR------ 163
Xt-NOXA1            LFSWEAQYNMAAVLCSLGR-WESAEEKLKETLQ--GDGRN-AKLDWALDQVQRR------ 167
Ci-p67phox          ISACDIRLNLALLHIFSGD-VPKAREILNEAMSMPHDEDKMKNCKSALDALVNENWVYFG 176
Sp-p67phox-N        LYECEVLYNLALTYATMMNNSEYALELLDEAKKVSVEARHGPLIEKAQKRLLLY------ 174
Sp-p67phox-C        LHKCEVLHNQAWAYN-ELDQRDWARNQLEEARECKADPRHE--KIEDSYRAFTSGRSFK- 431
Fg-NOXR             LYSCEVLFNRGLCYIYLQQ-MDAGMQDFSYAVKEKVVEDHNVIDDAINEQAEGYT----- 170
An-NOXR             LYSCEVLFNRGLCYIYLQQ-IGPGMQDLEYASKEKYSRSR--------LTPQGYT----- 162
Mg-NOXR             LFSAEVLFNKGLAQIYMGR-AQEGLADMEEARREKATDEHNVIDDAIQERGEGYT----- 170
Dd-p67-like         LLLAEVLFNKALALGRAGS--SVALQATQCFSQPSDSQEFKNQCKKIQDGSQLN------ 168
                    *       *
```

```
                                                  AD
Hs-p67phox          --KLYE-PVVIPVGKLFRPNERQVAQLAKKDYLGKATVVASVVDQD--------- 212
Cf-p67phox          --KLYE-PVVIPMGRLFRPNERQVAQLAKKDYLGKATVVASVVDQD--------- 212
Mm-p67phox          --KLFE-PVVIPVGRLFRPNERQVAQLAKKDYLGKATVVASVVHQD--------- 212
Rn-p67phox          --VVVDGPCCLAQYHVQRASHKQVPVLS-KDLLGEETVVASVVHQD--------- 212
Gg-p67phox          --KLYE-PVAIPTGKLFRPNEKQVAQLEKKDYLGKAMVVASVVDKD--------- 212
Xt-p67phox          --KVFA-AVKIPKGRIFQPNERLVEQLEKKDYLGKALVVASVVDKD--------- 213
Tn-p67phox          --KLFK-LTGFPSKVLFKPNKRYVAELEKKDYLCKAKVVASVVPQD--------- 212
Tr-p67phox          --KLFK-LIGFPSKVLFKPNKHYVAELEKKDYLGKAKVVASVVPQD--------- 212
Ol-p67phox          --KLFK-LVEFPSKMMFKPNKHYVAELEKKDYLGKAKVVASVNPQD--------- 212
Dr-p67phox          --KLFP-LVEVRAGLLFKPNKKYVAELEKRDYLGKAKVVASVVPAD--------- 212
Hs-NOXA1            --GSLP-PRQVPRGEVFRPHRWHLKHLEPVDFLGKAKVVASAIPDD--------- 213
Cf-NOXA1            --ATLQ-LRQVPRGEVFRPHRRHVEHLEPVDFLGKAKVVSSAIPDD--------- 213
Mm-NOXA1            --VPLQ-LQQVPKGEVFQPPRRYLKHLEPMDFLGKAKVVASVIPDD--------- 213
Rn-NOXA1            --VPLQ-LRQVPKGEVFQPPRRYLKHLEPMDFLGKAKVVASVIPDD--------- 213
Gg-NOXA1            --QFLE-PMQVPPGEFFRPRKKEVEQLDSKDFLGKPKVISSIIPND--------- 212
Dr-NOXA1            --DVLE-PLLLPEGEVFRPRKLEVDQLKPRDFLGEAKVITSMIPND--------- 212
Ol-NOXA1            --ELPT-PLTVPESLVFRPRKQEVEQLQKKDFLGKAKVISSLIPND--------- 206
Xt-NOXA1            --SLLQ-PMSVPEGEFFRPRKQEVEQLNSVDFLGKPTVISSVVPND--------- 210
Ci-p67phox          ESALERLVRLSSSCLFRPSKTKMEGLKSGTKFMNTATVVSATNDEY--------- 222
Sp-p67phox-N        ------EPVVLPSNQLFRPPKSKVVGLKNVDYLGKPKVVSELPESS--------- 214
Sp-p67phox-C        ------LYELPKPLLFKPPKSKIDNLDR-KDYLGKAKIVAKLPTVR--------- 470
Fg-NOXR             -------VFSIPVGVVYRPNEAKVRNLKTKDYLGKARLVAASDRAN--------- 209
An-NOXR             -------VFSIPVGVVYRPNEAKVKNLKTKDYLGKSRVIAANRLSTPAD------ 204
Mg-NOXR             -------VFSIPVGVLYRPSEKKLKNSMQKDYMGKAKLVAASDPNDIFT------ 212
Dd-p67-like         -----FSTRPIPLSLLFKPPK--VSDAPQKQRSATTSSIQSSSPSTPMSSSPPSY 216
                              *     * *
```

The N-terminal SH3 domain of Nox Activators (vertebrate p67*phox*)

The amino acid sequences corresponding to the N-terminal SH3 (SH3-A) domain (residues 240-299) of human p67*phox* were aligned.

```
Hs-p67phox          LEGEAHRVLFGFVPETKEELQVMPGNIVFVLKKGNDNWATVMFNGQKGLVPCNYLEPVEL 299
Cf-p67phox          LEGEAHRVLFGFVPETPEELQVMPGNIVFVLKKGNDNWATVMFNGQKGLVPCNYLEPVEL 299
```

```
Rn-p67phox          LEGEAHRVLFGFVPETPEELQVMPGNIVFVLKKGSDNWATVMFNGQKGLVPCNYLEPVEL 299
Mm-p67phox          LEGEAHRVLFGFVPETPEELQVMPGNIVFVLKKGSDNWATVMFNGQKGLVPCNYLEPVEL 299
Gg-p67phox          LEGQPHRVLYEFIPETAEELQVLPGNIVFVLKKEKDNWATVMFNGKKGIVPCNFLEPMEL 299
Xt-p67phox          LQGEPHRVLFEFNPETAEEMQVLPGNIVFVLKKGDDNWATVVFNGKKGIVPCNYLEPVEL 300
Tr-p67phox          LEGEPHTVLYKFVPETSDELAVVPGNVVFVLQKGADNWASVVFNERRGLVPYNYLERLEI 300
Tn-p67phox          LEGEPHTVLYEFVPETSDELAVVPGNVVFVLQKGADNWASVVFNERRGLVPYNYLERLEI 306
Dr-p67phox          LEGEPHTVLYEFVPETKEELAVLPGNIVFVLHRGTDNWASVVFNEKRGLVPYNFLEPLDI 299
Ol-p67phox          LEGEPHTVLFEFVPETCDELAVKPGNMVFVLQKGADNWAYVIFNGRTGLVPYNYLERQEI 299
                    * *  * ** * ***  *  * *** ****    **** * **    * ** * **
```

PB1 domain of Nox Activators (vertebrate p67*phox*, vertebrate NOXA1, fungal NOXR and Dd-p67-like).

The amino acid residues corresponding to the Phox/Bem1 (PB1) domain (residues 352-429) of human p67*phox* were aligned. *Red* and *blue letters* indicate the amino acid residues corresponding to the positions of two basic amino acid residues (K355 and K382) of PB1 domains of human p67*phox* and four acidic amino acid residues (D289, D291, D293 and D302) of OPCA motif of human p40*phox* PB1 domain, respectively.

```
Hs-p67phox          --YTLKVHY---KYTVVMKTQPGLPYSQVRDMVSKKLELRLEHTKLSYRPRDSN-EL-VPLS 406
Cf-p67phox          --YTVKVHY---KYTVVMEIQAGLPYSQLRDMVAKKLELLPEHTKLSYRPPDSH-EL-EPLS 408
Rn-p67phox          --YMLKVHY---KYTVVMETQLGLPYSQLRNMVSKKLELLPEHTKLSYQRRDSP-EL-LLLS 406
Mm-p67phox          --YMLKVHY---KYTVVMETRLGLPYSQLRNMVSKKLALSPEHTKLSYRRRDSH-EL-LLLS 405
Gg-p67phox          --YVLKVHY---KYTVAMQVKPDLSYKELLGLVCDKLELQPEHTMLRYKSAASG-EL-VPLS 408
Xt-p67phox          --YLVKVYY---KYTVAIQISSKLPFADLLTLISSKLQLLPSRMKLSFK--EDQ-DD-VLLN 402
Tr-p67phox          --CVVKVRY---TFTFAVLVPRGSSYATLAEKISEKLSVPANAIVLSLSSEATE-QN-VIDG 404
Tn-p67phox          --YVVKVRF---TFTFAVIVPRGSSYATLAQKVGEKLSVPADAVILSLSSEAAE-ED-VING 411
Dr-p67phox          --CVVKVHF---QFTIAIAIAHGQPYGVILQMISSKLKLPASTLTLRYAKEGSA-ER-VIIE 411
Ol-p67phox          --YIVKVHF---TFTFAVSIPRESSYEVLIRKISKKVKLPSDNISLSFSLDSSG-QR-AIDA 396
Hs-NOXA1            --VTVTVQC---AFTVALRARRGADLSSLRALLGQALPHQAQLGQLSYLAPGED-GHWVPIP 369
Cf-NOXA1            --VTVTVQC---AFTLALKVPWGAGLPHLRTLLSQALPLQAQHGQLSYRDPSHE-ARWVALP 347
Rn-NOXA1            --VTVTVQC---HFTVPLKVPRGTDLSSFRTLLSQALLQQTQKGQFSYKARGED-RAWVPIS 339
Mm-NOXA1            --VTVTVQC---HFTVPLKVPRGTGLSSFQTLLAQALLHQTQTGQLSYKAPGEE-RSWIPIS 341
Gg-NOXA1            RPAVLRVRC---ECTVVVRAGEVPSVPALRALLRERFGQQAERGRLSYRHLDGK--ELGAVS 410
Xt-NOXA1            --LVLSVHA---EFTVNMTVSKAITYPELQGALREELRKHGEQMANHLSYRDPESRGLTPVT 489
Dr-NOXA1            --VVVKVHY---TYTMALRVPAETPFRDLQEKIAQKLGQPAMNIRLRHRRPGTR-VL-TPLN 400
Ol-NOXA1            --VVVKVHY---TYTVAVTVPLNTPYHELKQRIAQKLGHPASELCLRHKQQGSH-LL-SPLS 416
An-NOXR             --RRFRVKVHSFEDTRYILIPPTIEFAEFETRIREKFGFQMA-LKIKMQDE----GDMITMV 481
Fg-NOXR             --TKIRVKVHA-DEVKLIMITPDTRFETLSDKVRDKFNIKRR-FKIKVKDDDMPNGDMITVG 486
```

```
Mg-NOXR          --TLIRVKLHYQDDTRGMTLTPDTPFADFMDKVTAKFGKQINGLGLKFKDED---GGKVTLR 515
Dd-p67-like      --ITLKVFYK---DRRLIQIPVPCNLSTFIQKIELKFEITISDKFSLSFQLD---GEENEIN 363
```

```
Hs-p67phox       -EDSMKDAWGQV---------KNYCLTLWCENT--- 429
Cf-p67phox       -EDNMKAAWGQV---------RNYCLTLWCENT--- 431
Rn-p67phox       -EESMKDAWAQV---------KNYCLTLWCEHT--- 429
Mm-p67phox       -EESMKDAWGQV---------KNYCLTLWCEHT--- 428
Gg-p67phox       -AQNLEEAWSHS---------KDQCLTVWCDCT--- 431
Xt-p67phox       -EENTEKAWSLA---------TDNCLKLKCT----- 423
Tr-p67phox       -GTDMEGVWSRV---------SGRCITLWCR----- 425
Tn-p67phox       -STDMEAVWGRA---------SGRCITLWCR----- 432
Dr-p67phox       -DSEMEAVWNSA---------KDGRLTLWCS----- 432
Ol-p67phox       -NTDMETVWSQV---------RAGRITLWCKEK--- 419
Hs-NOXA1         EEESLQRAWQDA---------AACPRGLQLQ----- 391
Cf-NOXA1         GEEALQGAWRDT---------AASPRGLQLQ----- 369
Rn-NOXA1         TEDSLQSVWRNV---------PVSPRGLQLQ----- 363
Mm-NOXA1         TEESLQSIWRNV---------PVGPGGLQLQ----- 361
Gg-NOXA1         GEEDLEKMWQQL---------TDGRITLCCQ----- 432
Xt-NOXA1         GSKDWQEVLKLS---------RANQVTLCCK----- 511
Dr-NOXA1         GDDGLDCLEGVA---------ESGRAQIWCQ----- 422
Ol-NOXA1         GEPG-GTVQDLA---------VAGRATLWCQRF--- 439
An-NOXR          DQEDLDLLLMASREIARREGSEMGKMEIWVEERPMI 517
Fg-NOXR          DQDDLEMVIDSVKDEARKQRTETGKMEIWILQL--- 509
Mg-NOXR          DESDYELAIETARESAK--GKPEGRLELWCMDL--- 546
Dd-p67-like      SQVQLDKMICMEINEINVKDIIPSPSPSPSPSP--- 390
```

The C-terminal SH3 domain of Nox Activators (vertebrate p67*phox*, vertebrate NOXA1 and *S. purpuratus* p67*phox*-like protein).

The amino acid residues corresponding to the C-terminal SH3 (SH3-B) domain (residues 457-526) of human p67*phox* were aligned.

```
Hs-p67phox       KKGSQVEALFSYEATQPEDLEFQEGDIILVLSKV----------NEEWLEGECKGKVGIF 506
Cf-p67phox       KEGGHVVALFTYEATQPEDLEFQQGDIIQIISMV----------NEDWLEGECKGKIGIF 508
Rn-p67phox       KEGTQVVAIFSYDATQPEDLEFVEGDVILVLSHV----------NEEWLEGECKGKIGIF 506
Mm-p67phox       KEGTQVVAIFSYEAAQPEDLEFVEGDVILVLSHV----------NEEWLEGECKGKVGIF 505
Xt-p67phox       AQGKYPIALFEYEATQPEDLPFCKGDIIKILSHV----------SEDWWEGECQGRMGIF 493
Gg-p67phox       ---TQVVAQYSYEATQPEDLEFQAGDVILVLSKV----------NEDWLEGQCNGKIGIF 501
Tr-p67phox       -KESSLLALHSYDSSNPEDLSFHQGDRITLLSKV----------NQDWLEGEFNGNTGIF 483
Tn-p67phox       RRETFLLALHTYDSPNPEDLTFQQGDKILLLSKV----------NQDWLEGQCHGNTGIF 492
```

```
Dr-p67phox             ---AKVVALYSYESSTPEDLEFKQGNVITVLSKV----------NDEWLEGQCNGKIGIF 488
Ol-p67phox             GEETHKVALHSYQSANPEDLNFKEGDEITVLARI----------NQDWLEGRCNGSTGIF 493
Hs-NOXA1               PVLYQVVAQHSYSAQGPEDLGFRQGDTVDVLCEE--PDVPL-AVDQAWLEGHCDGRIGIF 455
Cf-NOXA1               PVLYQAVAQHNYCAQGPEDLDLRQGDMVDVLCAGQGPDVPLHVVDPAWLEGHCDGRIGIF 436
Rn-NOXA1               PVLYQVVAQYDYRAQRPEDLDFRQGDTVDVLCEV----------DEAWLEGHRDGRVGIF 420
Mm-NOXA1               PVLYQVVAQYNYRAQRPEDLDFHQGDTVDVLCEV----------DEAWLEGHRDGCVGIF 418
Gg-NOXA1               PILYRMLAQHSYSAQGPGDLEFSKGDVLDILSEV----------NEDWLEGHCNGKTGIF 490
Xt-NOXA1               PVLYRMRAQYDYLAQGPADLSFQQGDLISILSEV----------NGEWLEGHCHRGIGIF 569
Dr-NOXA1               TILYQMVALYDYNAQGPEDLEFSEGDTIDILSEV----------NEEWLEGHVAGNIGIF 480
Ol-NOXA1               HILYQMVALYNYDAQGPEDLEFSEGDTIDILSEV----------NEEWLEGHCAGNVGIF 514
Sp-67phox              GQRKTALVIASHETDVEGEVSVSEGDLITITGEV-----------GDWLEVEVKGRKGRV 1094
                                          *     *    *                  * *       *
```

```
Hs-p67phox             PKVFVEDCATTDLESTRREV-------- 526
Cf-p67phox             PKAFVEEHATTDLESSPRGV-------- 528
Rn-p67phox             PKAFVEGCAAKNLEGTPREV-------- 526
Mm-p67phox             PKAFVEGCAAKNLEGIPREV-------- 525
Gg-p67phox             PSAFVRDGNTKDP--------------- 518
Xt-p67phox             PKVFTEE--------------------- 500
Tr-p67phox             PAAFVEEVPANG---------------- 495
Tn-p67phox             PAAFVEEVSVSE---------------- 504
Dr-p67phox             PSSFVEPLNGDPH--------------- 501
Ol-p67phox             PASFVNFCSKPRQL-------------- 505
Hs-NOXA1               PKCFVVPAGPRMSGAPGRLPRSQQGDQP 483
Cf-NOXA1               PKCFVVPAG------------------- 445
Rn-NOXA1               PKCFVVPAATCVEALP--VPEPQPGEQH 446
Mm-NOXA1               PKCFVVPAGAYVEAML--VLGPQPGDQN 444
Gg-NOXA1               PKCFATQTSCAAFP-------------- 504
Xt-NOXA1               PKCFAQR-AEGI---------------- 580
Dr-NOXA1               PQSFAHRDTDSISGASTD---------- 498
Ol-NOXA1               PSCFAYRENSSLYP-------------- 528
Sp-67phox              PRSCVKDFSKPRV--------------- 1106
                       *
```

PX domain of p40*phox* (vertebrate p40*phox*).

The amino acid sequences corresponding to the PX domain (residues 19-140 of human p40*phox*) were aligned.

```
Hs-p40phox             DVAISANIADIEEKRGFTSHFVFVIEVKTKGGSKYLIYRRYRQFHALQSKLEERFGPDSK 78
Cf-p40phox             NIAISANIADIEEKRGFTSYFVFVIEVKTKGGSKYLIYRRYRQFHALQSKLEERFGPENK 78
Mm-p40phox             DVAVSANIADIEEKRGFTSHFVFVIEVKTKGGSKYLIYRRYRQFYALQSKLEERFGPESK 78
Rn-p40phox             DVAISANIADIEEKRGFTSHFVFVIEVKTKGGSKYLIYRRYRQFYALQSKLEERFGPESK 78
Gg-p40phox             DVPVSANIADIEEKKGFTNYYMFVIEVKIKSGGRYLIFRRYREFYALHTKLEERYGGESK 78
Xt-p40phox             DVPVFAHIADVEERRGFSLYYTFVIEVKTKGSSKYFIYRRYSQFFTLHAKLEENYGP--D 76
Tr-p40phox             NVAVTATIADIEEKKGFIDYYRFVIEVKTKGGSKYFIYRRYREFFTLHQTLESKYSPEES 77
Tn-p40phox             NVPVTATIADIEEKKGFIDHYRFVIEVKTKGGSKYFIYRRYREFFTLHQNLESKYSPEDA 68
```

```
Ol-p40phox        NIPVSATIADTEEKRGFIDYFRFVIEVKTKGGSKYLIYRRYKEFFNLHQTLEAKYSPGDL 78
Dr-p40phox        NIPVTATIADIEEKKGFIVYFSFVIEVKTEGNSKYLIYRRYREFFALHQSLELKYTAEAQ 78
                       * *** ** **     ******     * * ***  * *   **

Hs-p40phox        S--SALACTLPTLPAKVYVGVKQEIAEMRIPALNAYMK---------------------- 114
Cf-p40phox        S--NPFTCSLPTLPAKVYVGVKQEIAEMRIPALNAYMK---------------------- 114
Mm-p40phox        N--SPFTCNLPTLPAKVYMGAKQEIAETRIPALNAYMK---------------------- 114
Rn-p40phox        N--SPFTCSLPTLPAKVYMGVKQEIAETRIPALNAYMK---------------------- 114
Gg-p40phox        N--SAFTCTLPVLPGKVYVGAKREIAENRIPILNIYMK---------------------- 114
Xt-p40phox        NGIAPYICTLPELPPKIFVGNKKDIAETRIPLLNGYMK---------------------- 114
Tr-p40phox        DRQGQNTCPLPPLPGKVFLGNKREIAEGRIPELNTYMK---------------------- 115
Tn-p40phox        EKPGPNTCLLPPLPGKIFIGNKKEIAEGRIPELNTYMKVISAGWALRMDGCRMARSDSPT 128
Ol-p40phox        ERPGPNTCVLPSLPGKIYIGNKQEIAESRIPELNTYMK---------------------- 116
Dr-p40phox        S--GYYNCQLPTLPGKVFMGNKKEIAESRIPELNNYMK---------------------- 114
                         * ** ** *   * *  *** *** ** ***

Hs-p40phox        ------------SLLSLPVWVLMDEDVRIFFYQSPYDS 140
Cf-p40phox        ------------SLLSLPIWVLMDEDVRIFFYQSPYDS 140
Mm-p40phox        ------------NLLSLPVCVLMDPDVRIFFYQSAYDA 140
Rn-p40phox        ------------NLLSLPVCVLMDPDVRIFFYQSAYDA 140
Gg-p40phox        ------------NLLCLPVWVLMDEEVRLFFYHSNFDS 140
Xt-p40phox        ------------GLLNSPTWLLLDEDLRMFYYQTLSDS 140
Tr-p40phox        ------------RLLGLPAWFLLDDTLRMFFYQTDQDS 141
Tn-p40phox        TFHQSPTIPSPPRLLGLPVWFLLDDTLRMFFYQTDQDS 166
Ol-p40phox        ------------RLLHLPAWLLLDETLRIFFYQTDEDS 142
Dr-p40phox        ------------RLLCLPTWVLLDDLIQCFSTRLSPTA 140
                              **  *   * *    *
```

Proline (P)-basic, SH3 and PB1 domains of p40*phox* (vertebrate p40*phox*).

The amino acid sequences corresponding to region from proline-basic (P-basic) region to PB1 domain (residues 144-339 of human p40*phox*) were aligned. *Gray boxes* indicate the amino acid sequences corresponding to the P-basic region (residues 144-158), SH3 domain (residues 170-229), and PB1 domain (residues 237-339) of human p40*phox*.

```
                  P-basic region           SH3 domain
Hs-p40phox        PQALRRLRPRTRKVKSVSPQGNSVDRMAAPRAEALFDFTGNSKLELNFKAGDVIFLLSRIN 204
Cf-p40phox        PQALRRLRPRTRKVKNESPQDAIFDRMAAPRAEALFDFTGNSKLELNFKAGDVIILLKGSS 204
Mm-p40phox        PQALRRLRPRTRKIKGVSPQGAIMDRMEAPRAEALFDFTGNSKLELSFKAGDVIFLLSKIN 204
Rn-p40phox        PQALRRLRPRTRKIKGVTPQGPSMDRMEAPRAEALFDFTGNSKLELSFKAGDVIFLLSKIN 204
Gg-p40phox        PRRLRRLRPRTRRVKSISSQLPVLDRVAAPRAEALFDFSGTSKLELSFKKGDLIYLLSRVN 204
Tr-p40phox        PRALRRLRPQTRKVKTIKP---KMDLFSSPRAEVMFDFRGNGKEELNLKKGEVIFLLQRVN 202
```

```
Tn-p40phox        PRALRRLRPQTRKVKTVKP---KMDLFSSPRAEVMFDFRGNGKEELNLKKGEVIFLLQRVN 227
Ol-p40phox        PRALRRLRPPTRKVKTVEPQ--KMDLFSSPRAEAMFDFRGNSKAELNLKRGEVIFLLRRVN 204
Dr-p40phox        PRALRRLRPPTRKVKTVKP---KTDLLSAPRAEAVFDFSGSGRLELSLKAGDVIFLLRRVN 201
Xt-p40phox        PRALRRLRPQTRKLKKDSQ---PISDVDRPRAEALFDFKGNAPMELNLKCGDLIYLLSWVN 201
                  *  ****** **  *              ****  *** *    **  * *  * **

                                                                PB1 domain
Hs-p40phox        KDWLEGTVRGATGIFPLSFVKILKDFP------EEDD----------PTNWLRCYYYEDTI 249
Cf-p40phox        FFLPQGTVRGATGIFPQSFVKILKDFP------EEED----------PTNWLRCYYYEDTI 249
Mm-p40phox        KDWLEGTSQGATGIFPGSFVKILKDFP------EDED----------TTNWLRCYFYEDTG 249
Rn-p40phox        KDWLEVRQRGRAEVFSSTLVFPLGAST------EPG-----------HRNHIGCPMFALEA 248
Gg-p40phox        KDWLEGTVNDATGIFPSAFVKIIKDLP------QQED----------TVNKIRCYYYDETV 249
Tr-p40phox        ADWLEGTVNNQTGIFPQSFVKIIKPLPDSNTEGEGEG---------HTYSCLRCFLLTPSG 254
Tn-p40phox        ADWLEGTVNNQTGIFPQSFVKIIKPLPDVSAEGEDEG---------HTYSCLRCFLLSPAG 279
Ol-p40phox        ADWLEGTVNNQTGIFPESYVKIIKALPESDSESNGGG---------HTYSCLRCFLLTPSG 256
Dr-p40phox        NADWLEGTVRDRTGIFPESFVKIIKALPENESDEEGGASRNSSRAQGGSYSCLHCYFLQPEG 261
Xt-p40phox        REWLEGTVGNRTGIFPASFVRIIKNLPELLYQ----------------VSLLRCYFHDHDR 246
                                                                       *

Hs-p40phox        STIKDIAVEEDLSSTPLL-KDLLELTRREFQREDIALNYRDAEGDLVRLLSDEDVALMVR 308
Cf-p40phox        STTKDIAVEEELSSTPLF-KDLMQLMRREFQREDIALNYRDAQGDLVRLLSDEDVGLMVK 308
Mm-p40phox        KTIKDIAVEEDLSSTPLF-KDLLALMRREFQREDIALSYQDAEGDLVRLLSDEDVGLMVK 308
Rn-p40phox        TVVTTTVIRGSVDHCPVFWKVLLSSMSREFQREDIALNYQDAEGDLVRLLSDEDVGLMVK 308
Gg-p40phox        STIRDISVEENLSSIPLF-KDLMELIKQEFDQHDIVLNYRDLDGDLIRLLSDQDVELMVS 308
Tr-p40phox        VDTRDVCVEEELTTQPTY-KDLLFHMRNVFKVNDIALNYRDMEGDLIRVVDDEDIQLMIK 313
Tn-p40phox        VDTRDVCVEEDLTTQPTH-KDLLTLMRNVFKVNDIALNYRDPEGDLIRVLDDEDIQLMVK 338
Ol-p40phox        VETRDVCVQEDLSIQPTY-NELLSRMRNVFKVEDIALNYRDLEGDLIRILDNEDVQLMIE 315
Dr-p40phox        IETRDICVQEDLSIQPSY-EELLSRMRDVFHVDDIALNYRDAEGDLIRILDDEDVVLMVQ 320
Xt-p40phox        CLIRDISLEEDVGKCPSY-KELLDLIRNQFPDAEVALNMRDKDGELIRLLDNSDMEFLIT 305
                                 *     *       *      *   *  * * *     *

Hs-p40phox        QARGLPSQ----KRLFPWKLH---ITQKD----NYRVYNTMP------------------ 339
Cf-p40phox        QAQGLPSQ----KHLFPWKLH---ITQKD----DYRVYNTVP------------------ 339
Mm-p40phox        QARGLPSQ----KRLFPWKLH---VTQKD----NYSVYNTVP------------------ 339
Rn-p40phox        QAQGLPSQ----KRLFPWKLH---VTQKD----NYGVYNTVP------------------ 339
Gg-p40phox        QSRKRSSE----KHFFPWKLH---ITHKD----DFSVYSTSPGIGDTQTVRAT------- 350
Tr-p40phox        ESRGQESKIKRPVNQFPWELY---VTLAS----DFSVYNTEL------------------ 348
Tn-p40phox        ESRDQQGKVKRPVNQFPWELHFASVTVSSPTRGDTSAVLSPLTPERRQMQVGVLLSVTQR 398
Ol-p40phox        EGKRQKGKVKRPVNQFSWELH---VTKAF----DLSVYNFEA------------------ 350
Dr-p40phox        ESKRTESKVKRPVNQFPWELL---VTHAK----DLTVYNTEY------------------ 355
Xt-p40phox        RGKRPP----RAKNYFPWELH---VTHED----DLEAYKTEA------------------ 336
                                 * * *     *
```
