# Supplementary material for: Molecular evolution of Phox-related regulatory subunits for NADPH oxidase enzymes
Source: BMC Evol Biol. 2007 Sep 27;7:178. doi: 10.1186/1471-2148-7-178 (PMC2121648; doi:10.1186/1471-2148-7-178)
Supplement: Additional file 4 — Alignment of full-length p22phox (vertebrate p22phox and C. intestinalis p22phox-like protein). Alignments of p22phox of chordates. [file 1471-2148-7-178-S4.pdf]

Additional File 4

Alignment of full-length p22*phox* (vertebrate p22*phox* and *C. intestinalis* p22*phox*-like protein).

Gray boxes indicate the amino acid sequences corresponding to the polybasic region (basic, residues 55-61), predicted 1st and 2nd transmenbrane segments (TM1-TM2, residues 91-120) and proline-rich region (PRR, residues 151-164) of human p22*phox*. Abbreviations: Hs (*H. sapiens*), Cf (*C. familliaris*), Rn (*R. norvegicus*), Mm (*M. musculus*), Gg (*G. gallus*), Xt (*X. tropicalis*), Dr (*D. rerio*), Tr (*T. rubripes*), Tn (*T. nigroviridis*), Ol (*O. latipes*), and Ci (*C. intestinalis*).

```
Hs-p22phox        -------MGQIEWAMWANEQALASGLILITGGIVATAG----RFTQWY-FGAYSIVAGVF 48
Cf-p22phox        -------MGQIEWAMWANEQALASGLILIMGGIVATAG----QFTKWY-FGAYSIGAGVF 48
Mm-p22phox        -------MGQIEWAMWANEQALASGLILITGGIVATAG----RFTQWY-FGAYSIAAGVL 48
Rn-p22phox        -------MGQIEWAMWANEQALASGLILITGGIVATAG----RFTQWY-FGAYSIVAGVL 48
Xt-p22phox        -------MGQIEWAMWANEQALASGLILLAGGIVAVAG----QFKGWQ-FGAYGVAAGVF 48
Tn-p22phox        -------MGKIEWAMWANEQALASGFILLAGGIVGVAG----RFRGWE-FAAYAVAAGVF 48
Tr-p22phox        -------MGKIEWAMWANEQALASGFILLTGGVVGVAG----QFRGWQ-FAAYAVAAGVL 48
Ol-p22phox        -------MGRIEWAMWANEQALAAGLILLAGGIVGVAG----QFRGWE-FASYAIAAGVF 48
Dr-p22phox        -------MAKIEWAMWANEQALAAGLIYLTGGIVGVAG----QFRGWQ-FAAFGIAAGVF 48
Ci-p22phox        MPSSSSNIRSIQWGMWANETALLGSYVLTLGGIIGIVGGLLKNFMFWLPIGIYGVVFGIL 60
                            * * ***** **        **     *     *  *          *

                        basic                               TM1-TM2
Hs-p22phox        VCLLEYPRGKRKKGSTMERWGQKYMTAVVKLFGPFTRNYYVRAVLHLLLSVPAGFLLATI 108
Cf-p22phox        VCLLEYPRGKRRKGSTMERCGQKYMTKVVKVFGPLSRNYYIRAFLHLGLSVPAGFLLATI 108
Mm-p22phox        ICLLEYPRGKRKKGSTMERCGQKYLTSVVKLFGPLTRNYYVRAALHFLLSVPAGFLLATI 108
Rn-p22phox        ICLLEYPRGKRKKGSTMERCGQKYLTAVVKLFGPLTRNYYVRAVLHLLLSVPAGFLLATI 108
Xt-p22phox        ITLLEYPRSKRKKGSTMERCGQKYLAAVVKLFGPLTRNYYVRAILHAGLAVPGGFILSTI 108
Tn-p22phox        VCLLEYPRSKRSKGTSVERPGQHCFTVCVKAFGPVTKNYYVRAVLHAAICVPGGFMLATV 108
Tr-p22phox        VCLLEYPRSKRSKGTSVERPGQRCFTVCVKAFGPVTRNYYVRAVLHAAICVPGGFMLATV 108
Ol-p22phox        VCLLEYPRSKRAKGTSVERTGQYCFTVCVKAFGPVTRNYYVRAFLHAALCVPGGFMLATV 108
Dr-p22phox        VCLLEYPRSKRGKGTSIERSGQYCFTVCVKSFGPLTRNYYVRAFLHAALCVPGGFMLATV 108
Ci-p22phox        VGLLEYPRGKKNKGNTLLRSGQSCFSTMVNKL-PFVSNYYFRAIAYFIVCIPGIISVPTF 119
                    ****** *  **    * **      *    *   *** **        *      *
```

```
                                                                PRR
Hs-p22phox          LGTACLAIASGIYLLAAVRGEQWTPIEPKPRERPQIGGTIKQPPSNPPPRPPAEARKKPS 168
Cf-p22phox          LGTACLAIASSIYLLAAYHGEQWIPIEPQPKERPQVGGTIKQPPSNPPPRPPAEARKKPS 168
Mm-p22phox          LGTVCLAIASVIYLLAAIRGEQWTPIEPKPKERPQVGGTIKQPPTNPPPRPPAEVRKKPS 168
Rn-p22phox          LGTVCLAIASVIYLLAAIRGEQWTPIEPKPKERPQVGGTIKQPPTNPPPRPPAEVRKKPS 168
Xt-p22phox          LGTVCLGIASIIYFLAAIRGEEWRPIEKQAEPKPRAGETIKRPPENPPPRPPAEVRRK-- 166
Tn-p22phox          LGCVCLGIASIIYLVAAIRGEHWEPILPKKEIQKPVAESIKNPPQNPPPRPPAETRRKRV 168
Tr-p22phox          LGCVCLGIASIIYLVAAIRGEHWEPILPSKEIRKPVAESIKNPPQNPPPRPPADTRRKRV 168
Ol-p22phox          LGCVCLGIASLIYLAAAVRGEHWEPILPRKDTRKPVAESIKSPPQNPPPRPPPEIRRKQA 168
Dr-p22phox          LGCVCLGMASLIYLSAPIHGEHWEPILH-IETKKRLGESIKEPPQNPPPRPPPELRRKKA 167
Ci-p22phox          LGSVCVIVGSGIYLGAALHKERWNPIESRPQVP-STSNDITQPPSQPPPRLPQNKQI--- 175
                    **  *    * **  *    * * **             *  **  **** *

Hs-p22phox           EEEAAVAAGGPPGGPQVNPIPVTDEVV 195
Cf-p22phox           EEEEAAGAVGVSGGPQENPVPVIDEVV 195
Mm-p22phox           EGEEEAASAG---GPQVNPMPVTDEVV 192
Rn-p22phox           EAEEEAASAG---GPQVNPIPVTDEVV 192
Xt-p22phox           --QADEVSVG---GGHVNPIPVTDNV- 187
Tn-p22phox           DDLEAAAYDN--------P-------- 179
Tr-p22phox           EDLEAASYDN--------PISVTANE- 186
Ol-p22phox           KDVEGAAYDN--------PMSVTDDE- 186
Dr-p22phox           DNLDAAAYDN--------PMSVTINE- 185
Ci-p22phox           ---------------------------
```
