# Supplementary material for: Molecular evolution of Phox-related regulatory subunits for NADPH oxidase enzymes
Source: BMC Evol Biol. 2007 Sep 27;7:178. doi: 10.1186/1471-2148-7-178 (PMC2121648; doi:10.1186/1471-2148-7-178)
Supplement: Additional file 5 — Amino acid sequences of Nox/Duox proteins. Amino acid sequences Nox and Duox proteins of the waterflea D. pulex, the gastropod snail-limpet L. gigantea, the nematode C. briggsae, the sea anemone N. vectensis, the choanoflagellate M. brevicollis, the fungi (C. cinerea, L. bicolor, P. placenta, P. graminis, P. blackesleeanus, and B. dendrobatidis), the plant O. sativa, and the red alga C. merolae are provided. [file 1471-2148-7-178-S5.doc]

**Additional File 5**

# Amino acid sequences of Nox/Duox proteins*.* In addition to analyzed sequences that previously reported (1), we searched for Nox/Duox ortholog sequences from databases of eukaryote genome sequences. The following naming was used to describe species of the sequences: waterflea*-*Dp (*D. pulex*), gastropod snail-limpet-Lg (*L. gigantea*), nematode-Cb (C. *briggsae*), sea anemone-Nv (*N. vectensis*), choanoflagellate-Mb (**M. brevicollis),** fungus-Cc (*C. cinerea*), fungus-Lb (*L. bicolor*), fungus-Pp (*P. placenta*), fungus-Pg (*P. graminis*), fungus-Pb (*P. blackesleeanus*), fungus-Bd (*B. dendrobatidis*), plant-Os (*O. sativa*), moss-Pp (**P. patens),** red alga-Cm (**C. merolae**), **oomycete-Ps (***P. sojae*), diatom-Pt (**P. tricornutum),** and amoeboflagellate-Ng (**N. gruberi)**. The long inserted region in Cm- and Pt-NoxD is indicated by underlines and it was trimmed prior to phylogenetic analysis. Sequences are obtained from the indicated servers: GenBank^TM^ numbering genes (http://www.ncbi.nlm.nih.gov/), the DOE Joint Genome Institute (JGI) database (http://genome.jgi-psf.org/euk_home.html), the Fungal Genome Initiative (FGI) by the Broad Institute (http://www.broad.mit.edu/cgi-bin/annotation/fgi/blast_page.cgi), the **Cyanidioschyzon merolae** Genome Project (CMGP) database (http://merolae.biol.s.u-tokyo.ac.jp/blast/blast.html).

Reference

1. Kawahara, T., Quinn, M.T., Lambeth, J.D. Molecular evolution of the reactive oxygen-generating NADPH oxidase (Nox/Duox) family of enzymes. BMC Evol Biol. (2007) 7:109.

>waterflea-Dp-Nox5A: JGI database jgi|Dappu1|55974|e_gw1.53.109.1

LLSKLFKLIDFNGTKNVSVQSVIVFIMDLSLPSKNHRMLRFESIDELFSSTNGRTELTLDDFKKIIPSKDAFFAERVFHILDKDGSKGISTSELRLGLEQLCSQSAEDRVRFLFQIYDGDGDGLIQLHDLRSVLRACTEENKMNFSDEQLDQLALALYEDAVDSVDDGNISAVNGLEFEHLKAQLEKHPGLIDELSFSLERFLLPSTVPRVRKSRWSASVFWSYLRNNAAFLAFLVAFLLVNIALFASRAYQYRQETPAYVIARACGQVLNFNCAFVVVLMLHHCITWIRQTKLAVFLPLDQHVYLHKLCGFTIIFFGTLHTAMHMINFSNELVSPVTNQTHSTEEWLFTTIPGLFGLIPGVANLTGWGLCVILGVMIPCSLPFVRKTGYFELFYWTHFLHYPFWALLILHGPHFWYWLVGPGALFIILEKLLRLRNRMTGKGRTFINYAILLPSSVTHLVCRKPPNFNFHAGDYVYLNIPAIASYEWHPFTISSAPELPDLIWFHIRGVGGWTNKLYEYFKQEQIKQYEFEHHSLRPASPFVSSDQSAFQTNVSFGNQSATIASAKNATENQSPFGWTKTYRPYNPQISITLSEVSTSESPSGAQSVAHTGYRYMRHPPEIINLPVPVEDPRNDDLPHAMWKKFNRKKGPRDTFGPETLRAHKTNINLNYIQLCLSKYFMYKRKKNIIQGESSVRDNRPNGITTTSDAIPDPKQMVEGVNIYLPLEITMDGPYGTPSSHIFRAQHAVLIAAGIGVTPYASILQSIMHRYYASLQKCPNCRYSWANQMPDSIMNLKKVDFFWINRDQRSFEWFVHVLSQLEMEQAEMDSALGRFLDLHMYITSALKKTDLKAVGLQMALDLLYAEEKRDLVTGLKTRTNAGRPNWDKVFQKLIDEDKGNVTVFYCGPPELSKVLKKKCNKFGFNFHKEIF

>waterflea-Dp-Nox5B: JGI database jgi|Dappu1|107862|fgenesh1_pg.C_scaffold_53000035

MADADPLKVGSTHPNEKALLTKSPAPSYDAMEVAEALYPALQLEDSGKTNWLSQAQQRTQALASLESLCNSYCAKEHKSAFSPDSFCQLFSQESVLQALFSLFDHQSKGQLVLSDFIESLKEKLGAEGTEFVDLLDTLWYVFTNETDADFETFCRIFKSKGVLIKLYALIDKNKADQVSVNQVTAFFTECTSQNEHRGSDICDPRRAEALFRSIAKDDSQMLEFEDFKKLIPSKNPFFAKRVFCIFDKSGSDSISLAEFREGMQQFCGKSDEDKVRCLFQIYDENGDGMIKLSELKAVLKACVEENGMKFSEMQLDELTTALYDDARDSSDDVNRASAKGLSFEELKTQMTKHPGLLENLSISLDRLLQPNAEKKIQSQSVFQSFSSYAKNNVPFIIFVLAYAIVNLGLIVSRGIQYKEENVFYILARCGGQALNFNCAFILVLMLRHCITLLRQIGCGYFLPLDLHVYLHKVCGGVVVVLSAVHTLMHLINFPLNIADKVESPVTKETHSAIEWLFTTEPGLFGLVPGLANLTGWALVGILIIMGLCSLPCVRKSGSFEVFYWTHLLYIPFWVLLILHGPNFWYWFIGPGILFFIEGTGRFRLRVTGKGRTFISSALLLPSRVTHLVIRKPENFNFNPGDYVFVKIPAITASEWHPFTISSAPELPDVMWLHIRCAGGWTNKLYDYFEREQAKLCLKQSAQNQQHNGGAGEPACRHCQRILGNDASFSTQNIMKGVRRLTRTFSNKNPVDKYDQLESCRMTLRPLKNSDQLNGAPDDPSSSITFNRAQSPALNRDPYDEGILRDKQHHRYPHHHQQPRAMSILVPIEETPGKSKTGSEVRKRATPRASYAPESMRKKPAQFTSRDENRSAHQPNSEKPHSVEGVHIYYPLEIAIDGPYGTPSSHIFRAQHAVLVAAGIGVTPFASILQSIMHRYYVSRQSCPRCHHSWVSQMPDSIMNLKKVDFFWINREQRSFEWFVEVLSQLEVEQAEMGSALGRFLDLHMYITSALKKTDLKAVGLQMALDLIHAKDKRDLVTGLKTRTNAGRPNWDKIFQKLVDEDKGKVTVFYCGPPQLAKELRKKCNEFDFGFSKEIF

>waterflea-Dp-Duox: JGI database jgi|Dappu1|53683|e_gw1.37.62.1

MGTYEKPRYDGWYNNLAHPDWGSVDSHLTRKVPATYGDGVYMMGGVNRPNARKLSELFMKGPDGLGSVMNRTALFVFFGQMVSSEILMASESGCPIEMHKIDIEKCDEMYDAECTGKKFMPFHRAWYDHKTGQSPNSPREQINRMTSWIDGSFIYSTSEAWVNAMRSFTNGTFKSGDSEGMPPRNKDRVPIFTAPAPHIMRMASPEKMLLLGDPRTNQNPAILAIGVVFFRFHNVIAGKIQQEHPEWSDEEVFQRARRVVVATLQNIVVYEYLPALIGESLGEYEGYKADVHPGISHVFQSAAFRFGHTMIPPGLYRRDGQCNFRLGPNETPAIRLCSAWWDAVEILVNNSVEEFILGMASQLAEREDSLLCSDVRNKLFGPMEFSRRDLGVLNIIRGRDTGLPDYNTARRSFHLSPITNWTDINPALAIQQPKLFPKLAELYGNDLGNVDVYIGGMLESTNGPGPLFTAVIKEQLGRIRDADRFWFENSDNGMFNASEIEEIRQTTFYDVILSTMNISEREIQRNVFFFRDGDPCPQPTQLNASLLTPCNFLAGYDYFAGSEGPYIYGCLLLAFVPIIAAGAGYGVVKLQNSKRRKLKARREENNNGKSVDKMVVKEWLHLNHKRLVKVKFGPEEAFYTVNRKGEKLRKVNFKGVELLSIEVTQDARKKPMLLVRVPRDYDLVLEFDSTASRNRFLHKLETFLTSHNKHLEQIPTYREQMLSNAETRERREMRLEHFFREAYALTFGPKPGEKRKMEEVTGDAIIVMRTSLSRSEFASALGMKGDDVFVKMMFNIVDKDGDGRISFQEFLDTVVLFSKGRTEDKLRIIFDMCDKDHNGVIDKGELSEMLRSLVEIARTNNSLNDDQVTELIDGMFQSAGLEHKDALTYDDFKLMMREYHGDFIAIGLDCKGAKQNYLDTSTNVARMTSFHIDAVQERHRNTILRKWDELTTFLEANRQNIFYLFVFYVVTIALFVDRFIHYSFMSEHTDLRHIMGVGIAITRGSAAALSFCYSLLLLTMSRNLLTKLKEFSVQQYIPLDSHIQFHKICALTAFFFSMLHTVGHVVNFYHVSTQPIEHLRCLSKEISLPSDYKPTITYWLFQTLTGLTGVLLFIVMIIIFVFAHPIIRKKAYNFFWMTHSLYIVLYILSVLHGLGRLTAEPNFWVFLIGPAIVYTLDKIISLRTKFMGLDIIETVLLPSDVLKVKFYRPPNFKYLSGQWIRLSCTGVKPEEFHSFTLTSAPHENFLSCHIKAQGPWTWKLRNYFDPSNFNPKDMDPKIQLDGPFGGGNQDWYKFEVAVMVGGGIGVTPYASILNDLVFGTSTNRYSGVACKKVYFLWTCPSHRHFEWFIDVLRDIESKDVTNVLEMHIFITQFFHKFDLRTTMLYICENHFQRISKRSMFTGLKAVNHFGRPDMTSFLKFVQKKHSYVSKIGVFSCGPRPLTKSIMSACEEVNQVRKLPYFIHHFENFG

>snail-Lg-Nox2: JGI database jgi|Lotgi1|175333|fgenesh2_pg.C_sca_269000003

MGEWIVNELPKWIVVTVWLLINTGIFIGTFFSYKNDIEYFYLRLIVGDALCFARASAACLNFNCFLILLPVCRNFISFLRGTCARCHRARRQLHRQITYHKYIAYMICLQTAIHIAAHCFDFEFLIEAYDSPTKSIQAITNLDTSNNGTWLNPVRVQGTDATREVFKTIAGVSGVIITLCLILIVSSSTETIRRWYFELFWYTHHLFIIFLIGFVIHGIQGIIRHQTNVSSHNPEKCYTRHDEWGTSPDCKIPQFAGSSPKSWIWILIPVIIFIIERGIRFYRSLQQVVITKVVKHPSNVFELQIRKKSFYADPGQYIFLHCPSISLLEWHPFTLTSAPGEETITVHVRRVGDWTEKLAKSCHVDEGEFQEAWKMPKIAIDGPYGTSTEDCFRFDVAVLIGTGIGVTPFASVLRHVWKKYSTRRNELQLKYVHFYWVCPSTSSFEWLQELLNSLESQMAEMGSANFLSHNIYLTSGWDSNQAKNIVLHNDEERDAVTGLQHKTHYGRPQWDKIFSDLAVFHKGMKLGVFFCGPKSLSTSLHSLCNKHSTESVGTRFYYNKENF

>snail-Lg-Nox4 : JGI database jgi|Lotgi1|155376|fgenesh2_pg.C_sca_8000164

MAYQVSVASWIRRHGQKYFLLIVWICGCLFLFYRTFYYFKDDPSFFYVRQILGVSMCISRGTASILNLNCGLILLPTCRCILTFITYLNSKVRISTIRVILESCKGFHKVCAITIILSSVIHVIGHIFNALKYSLYYNYQYPEVNGASYPNQNPIFIILWTVSGVTGLLMALILVLIVTSSYKHIRDSNYEVFYYTHRLIFIFYGLLLIHAVKESWVWIVVPLILYIVDGIIRIFNRKTTTITKITAYTGDVIELQLKCDGLKAIPGQYVLLKCPRISLFEWHPYSITKCPDKSSSGFNLCLRHKGDWSGNIHKAFNTELHYEDIVRSPELKVDGPYSSPLCEMNQSKLAVCIAGGIGVTPFISFLLKLRYKVGKLKKLYFIWIVRDLSHLAWFMEDILLSHQLLERKFPNSLEVQFYVTNSDSTTEQRHFSEDSLNFIQPRTMFHRPDWANVLSSVSHLHCRKTVDVFVCGPKQLIYDVRKITLKCCNKTNRFLLFEESF

>snail-Lg-Nox5A : JGI database jgi|Lotgi1|159226|fgenesh2_pg.C_sca_19000263

MANLELDPNCDEDCQWLEHMEAQFKKIAGEDNKIDLKEFKGALKIKESFFATRFFELFDNDKSGAIDLEELMRGLRLVIKGDYISKLKFLFDVYDADGNGSIDEAELRTVIQSCVDESSLSVSEADMNKLISVMFQSTDEDKSGLISFDELKTELDKHPGVMENLTISAAHWLRPPKPKKNTRACRYFTLKYWRNNRKEGVLFLLYFFINICLFALNCFLYRKSNVYIIIARGCGMCLNFNCAFILMTMLRKTITYIRTFKNLHFLPLDQHIEMHKLTGIMIAVFTVIHTLAHIGNAVVVAEDFGVTVWEFIFTTKANIGWVLGFAPLSGVILDVILIVMIICSMPFIRRSGHFQVFYWTHILYVPFWILCILHATNFWYWFIIPGIIFIIESLNRSKYIKKAAYGATYITNVYLLPYRVIHLVISRPPKWRYRPGDYVFIQIPAIAKYEWHPFTISSAPEQEGTFWLHIRSAGHWTNKLYDYFDSYDPNTYQYGTFNKGFGLDVMEAGGTIEEGTYRRLSSTGNTDQLKTTKPMSKIVRVKIYIDGPFGTSAREIFDTEHAVLVGSGIGVTPFASILQSILHRFQSATRTCPKCQHSFYDQRGPGMKIKRVDFIWINRDQKNFEWFVRMLTDIEVEQANYDKFGRIIHMHMYMTSALSKTDMKGLGLQMALELLHTKEHRDVLTGLRTRTHAGRPNFDELFGEIKQNKAGKVKVFLCGPQALAKDLKEKSDKFGFDFTKENF

>snail-Lg-Nox5B : JGI database jgi|Lotgi1|179958|fgenesh2_pm.C_sca_99000001

MQGTMRETGQSDTQWLQWVQNQFNNLAGNEGHITYKRFKSVLKIKESFFAEKFFDFFDNDGDEKIQLFELMDGLRTLTRGSTTDKIRFLFNLYDLDGNGHIDIDELKTVLQSCVNESSLKMSEIDVDRLTEVLFDSADTDCSGEITFEKLQTVLEQHPEVMKNLTISAAEWLKPSKTEKKRELPRYLQSNYIRNNLKKIIFFIIYFMTNIGLYAYGAYSYRESNNAIIIARGSGMCLNFNCMFVLVLMLRKCITWLRTWQYVYCILPLDQHILFHKTTGIMIAVHSLIHTMGHIGNAVIVSETNNITAVEVIFTTKANLGWLLGSAPITGVLLCPILIVMVICSMSFVRRSGHFEIFYYTHMLYIPFWILCIIHASNFWKWFIAPGLIFFVESILRSKPMKLATHGRTFIKEVNLLPSGVTHLVISRPPNFDFKPGDYLFIQIPMLAKYEWHPFTISSAPEMEGFIWLHIRSAGYWTKSLYDFFEKYGSDQRRKSTITGQINKAFHDGLDKIERGIKEVSTSFTHRQSKGTYLFLRKITAEVYLDGPYGTSTREIFQTEHAVLIGSGIGITPYASILQSIIYRFNAITRTCPKCHHCFNEPLAGSMMKLKKVDFVWINRDQRAFEWFTSLLTQLELEQVKNGSHLDKILNLHMYMTAAVQKADMKGIGLQVALDLIHEKEQRDLLTGLRTRTKAGRPDFNELFKSIAAQKHGKVKVFFCGAPVVGKAIKEHCAKFKFGFRKENF

>snail-Lg-Duox1: JGI database jgi|Lotgi1|71226|gw1.58.83.1

DGYYNNLLHPDWGAIDGQLLRPSRPDYSDSVFEPSGTDRPNPFTVSDTAHFGEPGNGSARGRNSLLVFFGQQVVEEIMDSQRPGCPREFFNVKVPTGHPIYDREGKGNIEMPLLRTRFDQRTGYSPNNPRQQLNEITPYIDGGLMYGTSKAWTDTLRSFKNGELLAEGNEEDIKLSFPNQNDIRLPMANPPPPREHILKPVNRFYRLGNPRGNENPMLLAFGVMWFRWHNVVARKLKLQHPTWNDEQLFNRARQFVIAHHQNIVMYEWLPKWISIYENGTEENISDYSGYKPNVHPGISQEFQAAALRFGHTLVPSGIFTRSVDIVDNGQCKVTSNIITNGTGHNVPYTGIRVCTSFWNSQVPLETEEPGIDSVFLGMIHTLSEKEDHIIVTDLRGNVFGPLDFNQRDLAAINIQRGRDHGLPGYNEIRQTYGLNRLGNWSDINPDLTEILEKLRELYDNDTAPDNLDIFTGGLLETTDDGLGPLFKAITIEQFERIRDGDRFWFENRNNGIFSDAEIEEIKQITLKDIIDNTTNIGADQMSNSVFFYLFIDDTIAECNCMAPFNRTAIDNGIHEECVDLSTYDYFSGSEASFALTFVALGLCVPLTIGIMIFMAKRKEKKMAEANRRRPARSDTTDPNKFSATEWVGIKSGERNVKVELDRNRKKIMVTDRTGKTLRLIDLRRTDRVHIRISDDKDQSVISVRVPGETDLILRFIGLSERQTIVGNIEKFLQDLSINRERHEFTEATIMKDAVTFDDRKKLLDTFFKVICLQSALSDLGIENLDLTTANEIRKIKLTRTEFAEALGLQPDSLFVRNMFLLVDRSKDGFVSFEEFLSMFVTMASGGAEDKAKLLFNMYDLKRKGELTRRDFTKMIRSMMDMADATLDDSRVDTFLDHMFEQAGIANKQTMTFDDFKKIFASEEHGEILQNATLAFDKDIVLFITVEKKSGNGNSNLLMRKKTVINQYRDKTFHPQSRNRESKIHIEATKKQIPMGKFQTKIYEITRYIENYRLHIFWFILFNLVTLGIFIERAYYYSIEREHAGLRRLAGYGVSVTRGAASVQMFLYVSLLVTMSRNTLTFFRETFLHRFIPFDAFHAMHKYTALLALIFTAMHIIGHGINLFHISTQASSDLNCYFREYFRATDVLATFHYWAFTTITGITGVILTLIIIVMYVFATEYGRRHLFNAFWFTHNFYVFLYIFLILHGIGRLVQDPLFPYFFLGPLVVFVLDKMVSLSRNKVEIVVKKAEILPSGVTNLVFKRPLNFDYKSGQWVRIACLDLGQSEYHPFTLTSAPQEENLSLHIRAVGPWTTNIRRVYDINNIVSEKGFPKLYLDGPFGEGHQDWYRYPVAVLVGGGIGVTPFASILKDLVQKSKMKVKFPCQKVYFLWVTRTQKSFEWMTDIIREVELGDVNNLVSVHIFITQFQQKFDLRTTMLYICERHFQKVAGQSLFTGLRATTHFGRPKFQDFLLSLAHEHDGVPQVGVFSCGPPPMTSNVDKACSSLNKLEGPTYVHHFENF

>snail-Lg-Duox2: JGI database jgi|Lotgi1|112284|e_gw1.15.217.1

MLCYLLGNLYKFNPANPFLNPGCLKCLRHAFFFFSVVNAAYTRSDEIELHPLNGYYNNLFNPDWGISGNQLRRLSPPDYSDGAYEPSGVSRPNPMVISDTIHNGPSGKASTTGRNALFVFFGQLVADEMISTGNPGCPPEYTNIPIPEGHKYRALATEMTYQRSGYNAKTGNSPNRPRQQQNGVTSFLDAGFLYGTTKFFTDQIREFRNGRLKASDQTSTRFPAKNDIRLPLVNAAVPRDHTLKPATRFFRIGNARGHMNPYLLSLQIVWYRWHNQVAEQLHAANTGWKDERIFTEARKRVTAHFQKIVINDWLKFLLSTNDSSPLPPYKGYQNSVHPGATQEFQVAMEAISHTMTPGGVFTLSDTCQISPTTVLNRQNEGVKVEAVRLCNTYWDPQDVVENNFESIIRGMVYTRAEKEDLDVVADIREYYPGPLEFSRRDQVAVTIQQGRDHGLPSYNTVRTSLRLSPLESWFDFSSGNSTVCKINTSLMINALVDLFGTTDDLDLYTGGLLEIQDNNPGQLFRAIIVDQFTRIRDGDRFWYENPNNGLFTPTEIADINRITFSDILRNVTSSPGQILPDAFNYAPDSCQCRDPPNIDSNNDTRYDQCSQLELYDYFSGSEVSFALSFLALFLVIPGSFMVLMLLIRMRKKKFEAKSKKITLKQKSANCYIAEEWLGMKSENRKVKVEFQQDRKKINVKNCRGQNVRFINLRQVPKIQIKLSYDKQFNLVLVRVPGEVDLILRFESLTERQEFVVDLEKFGGELNIEITRTEQAEKVILTTSNNRDDRQKILDAFFRVVCLQVQFFIELDFENEKSRFPLFHKQASEVINIKLTRTEFAEALGLQPSSIFVRNVFLLADKDKDGFLCFREFLDMFAIFARGSAEEKARLMFNVYDIKRKGILNRRDFEKMIKSLLDLSEAKLNDDKVKELIEVMYSQAGLNNRDTMNFDDFKKVFASDEYKTTFQSATLADQGEKRLNWSFPTREFGLCSQSVQDTYNIYNDDLDSYPPNGLFNIIHVHYRGKREKGVINRKNRRSKVTLESEATPVKGPTGIQEKIYAITKYIEIYRLHIFWVLLYTLVTCGIFIERAYYYAEGREHAGLRRLSGAWTTAMIRGSASVIMFTYVSLLVTMCRNTITLLRETVLHRFIPFDSAVAFHKYIAVLAMIGTIVHIFGHAVNLYCVVTQPPQDVSCLFREYFRGSHEIATFHYWAYQTITGLAGVVVTIVIFIMYVFATQFARRNHFTAFWLTHSLYLTVYPLTILHGIGVLVQAPIFPYYLFGPLVLFVFDKLASLSRNRVEIRVKKVSILPSDVTELVFRRPANFDYRSGQWVRIACLKLGKGEYHPFTLTSAPHEENLSLHIRAVGPWTKNLRKIYEFYSIGNKPLPLLYVDGPFGEGHQDWFTFDVAVLVGGGIGVTPFASILKDIAFKSKAGIKINCKKVYFVWITRSQKHFEWLIDIIRDVEEQDVLEIVNVHIFITQFTQKYDLRTTMLYICERNFQKVENKSLFTGLRAVTHFGRPKMEDFLHSLHHEYPEVERFGVFSCGPGPMTNSVQAACTLLNSIEGPTYCHHFENF

>snail-Lg-Duox3: JGI database jgi|Lotgi1|71249|gw1.61.117.1

FDGYFNNEQRPEVGSAGRHIARNITTNYRDHTYFPSGWDRPNPRSISNILFKGPTGLPSYHRRSALFSFFGQFVMREVLDTDDTTCPVEVIQTHVPRCDPDFDPDCHGNQVMPYERSAYDKRTGQSPNNPRKQINKASCFIDGSVVYGTNAVRSSYLREPGTGKLYCEDIWGKFPKLNDVQMAFTNFPGQKHKYKDQSKLWRMGDTHIYENPGLLSLGLVFFRYHNYQADQIMLKNPQMSNNEVFERARRWVIATLQKIIMYDWLPLLLNENLSMFVFSVGYKPRVQSEVTDIFDAAAIQYIQTLIPPAIYQRTNKCEFLKVGKSKAGRLCNSYWDSKDVVNVNGLEKILLGLTSQIAEREDHVIVEDLRSKFYGPLFYSRHDSAMLTILKGRDYGLPDYNTARVKMGLEPIKNWTDVNPELNSTLISELKMLHHDKLDKIDIFTGGLLETTRNGPGELFRHIIIDQFLRLRDGDRFWFENKQNGIFSEEEIKEIHKITLNNVIIWTSNEIEAEHLDRDAFTVSKGKYCYFSDHPCPQPEQLTEDQLDECPKHVGYDFFSGSEIPYIIIWTCMGLIPIGKTEFCIFTRMLRKGSCENAQCFADFETGNDESFVELENEDFHDTCEWRCKKEPSRNIELHLTSKGSIEIFSLSGSHLRCIKLVEHMTMTIYLSINKGSNVMLIQIPKEYDLVLVFNSELNRNVFDEDLSQFLASYEIETDLKEKKLKDIYSMAMTKEKRNKQLEKFFKTVFTEAFKLDYDPAFEQNQLDMKTQSKEILEMELSKEEFAEALAVKPDSDFINHFFSLIDADRNGYISFREFLHAVVLFSKGSCQDKLQTIFYMYDAEGSGRMNRYNVCKMFSSLLELAQSNVEQDEVEELVDSLCDKAGVASDQDLCFENFCQIFAPQMDKLWNASIDWKGRLIVIHVISWSLILLSRTAELLPLIKIHTSLYIKIIQRNYQVSEGEIRHRHSVPNEKNNASFKNFTQVSVREHYTPFKAKVKVIKHFIENYRQHIFFLVLVFGIVLGLFAERFYYYTVEREHSGLMRLMSYGISITRGAAAAMSFTFSLLLLTMCRNTITYLRSTFLNMFIPFDSHISFHKVIAWTALFFSGLHVIGYSFNFYHLATQPTKFLCIFDSIVFRADKLPTFSFWLFGNMTGFTGVLLVVVLCIMYVFATQTARSHIFNLFWLTHKLFIIMYVLVILHGASIIVQKPLFFAYFIGPAALFTLDKMISLSRKKTEINIVKAINLPSDVTMLEFKRPPKFEYKSGQWVRIACLSHGSNEYHPFTLTSAPHEDTLKVHIRALGPWTWNIRQTFDLENLKDNPYPKLFLDGPYGAGQQDWYQYEVSLLVGAGIGVTPYASILKDFVHMTSIKNTYKVKCQKLYFIWVTGSQRHFEWLIDILREVEEIDERGMVSIDIFITQFFQNFDLRTAMLYVFEEHFQKMTGGKSVYTGLKATTHFGRPQLNNIMTAVNRAHPMTRKIGVFSCGPPGVTKGVERACVDASKSTKAIFEHHYENF

>nematode-Cb-Duox: GenBank No. CAE61283.1

NEKSLSSGLQQNEEFQRYDGWYNNLANKEWGSAGSRLHRDARSAYSDGVYSVNNSLPSARELSDTLFKGESGIPNTRGCTTLLAFFSQVVAYEIMQSNGVSCPLETLKIQVPLCDSVFDKECEGKTQIPFTRAKYDKETGNGLNSPREQINERTSWIDGSFIYGTTQPWVSSLRSFKQGRLAEGVPGYPPLNNPHIPLNNPAPPQVHRLMSPDRLFMLGDSRVNENPGLLSFGLILFRWHNYNANQIHRKHPEWTDEQIFQAARRLVIASMQKIIAYDFVPALLGPDVRLSNYTKYMPHVPPGISHAFGAAAFRFPHSIVPPAMLLRKRGNKCEFRTEVGGYPALRLCQNWWNAQDIVKEYSVDEIILGMASQIAERDDNIVVEDLRDYIFGPMHFSRLDVVASSIMRGRDNGIPPYNELRRTFGLAPKTWETINEDFYKKHTDKVQKLKALYGGNILYLDAYIGGMLEGGENGPGELFKEIIKDQFTRIRDGDRFWFENKLNGIFTEEEVQMIHGITLRDIIKATTDIDEGMLQDDVFFFKEGDPCPQPFQVNTTGLEPCVPFMQSTYWTDNDTTYVFTLIGLACVPLICYGIGRYLVNRRIAIGHNSACDSLTTDFLNDACNPKGDVYCVNALEWLQEEYIRQVRIEIENTTLTVKKPRGGILRKIRFVAEQDIDVFHSTPNPEAMHGPFVMLSQKNNHPLVIRLPSDRDLSRFLDQIREAATGINAKVNVADEENQVLLDQAVTKERRQDRLDQFFRESYAKAFNDDELRDTETSIDSTSDDILNETISREELASAMGMKADNEFVKRMFAMTAKHDEDSLSFNEFLKVLREFVNAPQKEKLQTLFKMCDLDGKNKVLRRDLAELVKSLNQTAGVNINEGTQMRLFNEVLHSSGVSNDAKYLTYEDFNALFSDIPDKQPVGLPFNRKTFQPSIGDSSSLNSFAVVDRSINNSAPMTLVHQVCAFLETYRQHVFIVFCFVAINLVLFFERFWHYRYMTENRDLRRVMGAGIAITRGAAGALSFCMALILLTVCRNIITLLRETVISQYIPFDSAIAFHKIVALFAAFWATLHTVGHCVNFYHVGTQSQEGLACLFQEAFFGSNFLPSISYWFYGTITGLTGIALVTVMCIIYVFALPCFIKRAYHAFRLTHLLNIAFYALTILHGLPKLLDSPKFGYYVLGPVIIFVIDRIIGLMQYYKKLEIVSAEILPSDIIYIEFRRPRTFQYKSGQWITVSSPSISCTFNESHAFSIASSPQDETVKLYIKAVGPWTWKLRSELLRAQNTGSPYPLIHLKGPYGDGNQEWMNYEVAIMVGGGIGVTPYASTLNDLVQLTASDSFHKVRCQKVYFLWVCPSHKNYEWFVDVLKNVENQDRRGILETHIFVTQLFHKFDLRTTMLYICEKHFRATNAGMSMFTGLHAKNHFGRPNFKTFFQFIQNEHKEQSEIGVFSCGPNNLNEKIAEGCAEANRQRDAPSFAHRFETF

>sea anemone-Nv-Nox2A: JGI database, jgi|Nemve1|168974|estExt_gwp.C_1170131

MANLGNDVARYIVLIIWMGINIYLFIDVYYKLATTKENMYLNKLVKFGLALARAPAMVLNFNCMLILLTMCRNLLSFIRGSCKCCSFMLRLLDKHHVTFHKYIAYMICLQTAIHCIAHIFNVEFLILAWQKGGIYTKLCQLEDVGNETYINPIRDPNADPITVLWTIVSGVTGAVITLALILMLSSSTELIRRSYFEVFWFNHHCFVIFYIGLVLHGVQGIIRYQSNVDKHDPEICMNHTLWYNHPKCMEPPKFVPFGMNTWRWVVGPMFLYCIERLIRFVRSHQAIQIIKVVKHPSNVIEIKMKKAGFRPEVGQYIFLQCPKISKLEWHPFTLTSAPEEDTFSVHIRIVGDWTGDLAKLCGYKGQKLQSVNEMPRLALDGPFGTASIDVFKYGVGMYIGAGIGVTPFASVLKSIWYRYNENPSDLNIKKVYFFWICNDTNAFEWFTDLLKILEEKMIDSGNAGFLEYNIYLTRGWGANMARDIYLREDEVEDPITRLRQKTRFGRPEWNKIFNDIGNKHPKTDIGVFFCGPKALSHTLHKMSNAHSRDGDGAKFYYNKENF

>sea anemone-Nv-Nox2B: JGI database, jgi|Nemve1|113989|e_gw.122.48.1

MNFYFSQIIWAVINIILFATTYHNYGVTKKYTYLREVIKNGLPIARGAAMVLNFNCMLILLSMCRNLNSLIRRHCKSTCMAPVIRVLDKSITFHKYIAYTICFFTIVHVGAHCYNFENLIDSWSKENEIDAKLSQLDGADNWVNPIRSEMSVGKRGSKFAGVTGAVITLCLVVMVSSSTELIRRSYFEVFWYSHHLFIIFFAGLVAHGCGEILRYQTNMDKHDPDVCRKQENLATWGVKDPCRVLPEFEAAGAMTWKIVLLPMVIYFLERCLRFWRSMQQVKVVKVVVKHPSRVVEIQMKKPGFVCEAGQYVFLQVPKISQLEWHPFTLTSAPEEDYFSLHIRVVGNWTTDLANQLGAGNQQIAIDQMPRIAVDGPYGTASTDVFRYEVVMCIGAGIGVTPFASILKSIWYRHNQDLENLRVKKVYFYWICRDTFAFEWFSDLLKHIEIQMDEMNMPGFIQINIFLTGWDKKLANQVVMERSADRDPITGLFARTKYGRPEWAKIFNEVAEAHNQTSVGVFFCGPSGLSHELHKMCTQSCDETKGVRFFYNKENF

>sea anemone-Nv-Nox4: JGI database, jgi|Nemve1|88712|e_gw.19.15.1

YFFFQVLWTGPFIVIFWQTYNKYSNCPEYFYLHNMLGNSLSWSRGAAAVLWFSCSLMLLPMCRNLLAFVRNTLCKSSRRLRRLLDKHIWFHKACAITTIIAAVVHTVAHLINGKRFSENYSTDHPPLNFAKNRDQDPLEFVMLSVAGFTGMGMMLVLLIMIAASTPIVRNRSYEVFWFTHHAFIAFYLMLAVHGLGGVIKHQTNLAAHTPGCKVPLNDSMDTPFNMTSLPLCREAEFAPDQPQVYHKSNTQVYQKPYHTVYCKCYHTGIPQSWKLLILPLSIYLIDRLIRVVRGYQEVTVIKVVNHPCGVIELHMKKSGFYAEPGQFVYVRCHSVARFEWHPFTLTKCPSSKDDSFSIHIKRTGDWTKSLSDQKPLLAALDDSGSLVAAPSVRSAVLSVDGPYGSPCMDVEEYRVSMCIATGIGVTPFAALITRIRSQIIHAQRPPRPHRLYFVWICREVGALQWFADLIHETSRQLWELNRPDFLTCLFYITSKGQKEKICINHTQSSWFDARLTHGRPDWFQIFRRVSQENPKTCVGVFYCGARGPSSMLRRCCQRMYKNGASFVFNKEVSA

>sea anemone-Nv-Nox5-like (flagment): JGI database, jgi|Nemve1|28174|gw.127.63.1

FEYKAGQYCFLCVPGVSMFEWHPFSISSSPHEATVSLHIRVLGDWTQQLYDYVKDTRPINVYIDGPYGAPGVDVDGDRYKVFLFVSGGIGITPMQSICNDILHQRRRGRDIRKVIFVWSVRD

>choanoflagellate-Mb-Nox2: JGI database, jgi|Monbr1|33523|estExt_fgenesh2_pg.C_200105)

MQFWWQQGLTFRQWFVNDGPMYTFVLLWMCANVGLFFYTFSFYTQDKYNYLRRIVHFGLPVARGAASVLNLNCALVLLPVCRNLVNFCRGIFESKRSIRRLFDKNILFHKWCAYVICVFASIHICAHFFNVNNLVEDGTYGRILRDGTPMSQEEVLFTTVAGGTGVGITVPLILMVTTASQQIRRSYFELFWYTHHLFVVFYVCLCLHGYSGFVERQDNPDTYPISLDRGVCTSRDDVLVSNGCASWNAVVSALANQNSSLTEPNLCIPGGVDVGDEEDAFCCPCRLVTQAVLQRGQAATWVWVIGPLILYILERLYRWYKSQTRRLRILKVVKHKDSVPVMEIQFQKVPTKAGQYVFINCPKINSLEWHPITLTSCPELDYVSVHIRLVGDWTTKLADACGFYEDNPKVGSELPYICIDGPFGTASEDMYHYPVAMLIGAGIGVTPFASLLKELYFRKSNPSAYPSFKTQKVYFYWMCPGFDAWGWFASLLIDLEDKLEQLGVPDFLEIRVFTTRGWSQDDAAKIMLQEDESGDSIVRDAETGRALRHKMNFGRPNWDSEFTSVANTHAGNNIGLFFCGPKVLSSQLHVTCNKFTSERAAEGTKFYYNKENF

>plant-Os-rboh-A: GenBank No. CAF33404 (initially termed RbohAOsp)

MRGGASSGPQRWGSAGTTPRSLSTGSSPRGSDDRSSDDGEELVEVTLDLQDDDTIVLRSVEPAAAAAAGVGAGAGAASARGELTGGPSSSSSRSRSPSIRRSSSHRLLQFSQELKAEAMARARQFSQDLTKRFGRSHSRSEAQAPSGLESALAARAARRQRAQLDRTRSGAHKALRGLRFISSNKANNAWMEVQANFDRLARDGYLSRSDFAECIGMTESKEFALELFDTLSRRRQMKVDTINKDELREIWQQITDNSFDSRLQIFFEMVDKNADGRITEAEVKEIIMLSASANKLSRLKEQAEEYAALIMEELDPEGLGYIELWQLETLLLQKDTYMNYSQALSYTSQALSQNLAGLRKKSSIRKISTSLSYYFEDNWKRLWVLALWIGIMAGLFTWKFMQYRNRYVFDVMGYCVTTAKGAAETLKLNMAIILLPVCRNTITWLRSTRAARALPFDDNINFHKTIAAAIVVGIILHAGNHLVCDFPRLIKSSDEKYAPLGQYFGEIKPTYFTLVKGVEGITGVIMVVCMIIAFTLATRWFRRSLVKLPRPFDKLTGFNAFWYSHHLFIIVYIALIVHGECLYLIHVWYRRTTWMYLSVPVCLYVGERILRFFRSGSYSVRLLKVAIYPGNVLTLQMSKPPTFRYKSGQYMFVQCPAVSPFEWHPFSITSAPGDDYLSIHVRQLGDWTRELKRVFAAACEPPAGGKSGLLRADETTKKILPKLLIDGPYGSPAQDYSKYDVLLLVGLGIGATPFISILKDLLNNIIKMEEEEDASTDLYPPMGRNKPHVDLGTLMTITSRPKKILKTTNAYFYWVTREQGSFDWFKGVMNEIADLDQRNIIEMHNYLTSVYEEGDARSALITMLQALNHAKNGVDIVSGTKVRTHFARPNWRKVLSKISSKHPYAKIGVFYCGAPVLAQELSKLCHEFNGKCTTKFEFHKEHF

>plant-Os-rboh-B: GenBank No. NP_001056115

MRAGIGSGSGGGTTPVRPRWGSGVTTPRSLSTGSSPRGSDRSSDDGEELVEVTLDLQEDDTIVLRSVEPAAGGAAVASSSGASPSAVAPPRRAEPPGGVASRSRSPAMRRTSSHRLLQFSQELKAEAMARARQFSQDLTKRFTRTQSTTTAPPGIESALAARAERRQRAQLDRTKSGAQRAIRGLRFISGPNKASNAWIEVQANFDRLARDGYLSRDDFPQCIGMTESKEFAMELFDTLSRRRQMQVDKINKEELREIWQQITDNSFDSRLQIFFDMVDKNADGHITEAEVKEIIMLSASANKLSRLKEQAEEYAALIMEELDPEQLGYIELWQLETLLLQKDTYVNYSQALSYTSQALSQNLAGLRKRSPIRKISTKLSYYLEDNWKRLWVLALWIGIMAGLFIWKFIQYRHRYVFNVMGYCVTTAKGAAETLKLNMAIILLPVCRNTITWLRNTRAARALPFDDNINFHKTIAAAIVVGVILHGGLHLVCDFPRLIGSSEEKYAPLGKYFGETKPTYLTLVKGVEGITGVIMLVCMIIAFTLATRWFRRSLVKLPKPFDKLTGFNAFWYSHHLFIIVYISLVIHGEWLYLIRIWYKRTTWMYLAVPVCLYVGERTLRFFRSGSYSVRLLKVAIYPGNVLTLQMSKPPTFRYKSGQYMFVQCPAVSPFEWHPFSITSAPGDDYLSIHVRQLGDWTRELKRVFSAACEPPVGGKSGLLRADETTKKALPKLLIDGPYGSPAQDYSKYDVLLLVGLGIGATPFISILKDLINSIIKMEEEEEASGDLYPPIGRNKAHVDLDTLMRITSKPKRVLKTTNAYFYWVTREQGSFDWFKGVMNEIAELDQRNIIEMHNYLTSVYEEGDARSALITMLQALNHAKNGVDIVSGTKVRTHFARPNFKKVLSKIASKHPYAKIGVFYCGAPVLAQELSDLCHDFNGRCTSKFEFHKEHF

>plant-Os-rboh-C: GenBank No. NP_001043020

MADLEAGMVAAATDQGNSTRSQDDAATLIPNSGNLGSSNRSTKTARFKDDDELVEITLDVQRDSVAIQEVRGVDEGGSGHGTGFDGLPLVSPSSKSGKLTSKLRQVTNGLKMKSSSRKAPSPQAQQSAKRVRKRLDRTKSSAAVALKGLQFVTAKVGNDGWAAVEKRFNQLQVDGVLLRSRFGKCIGMDGSDEFAVQMFDSLARKRGIVKQVLTKDELKDFYEQLTDQGFDNRLRTFFDMVDKNADGRLTAEEVKEIIALSASANKLSKIKERADEYTALIMEELDPTNLGYIEMEDLEALLLQSPSEAAARSTTTHSSKLSKALSMKLASNKEMSPVRHYWQQFMYFLEENWKRSWVMTLWISICIALFIWKFIQYRNRAVFGIMGYCVTTAKGAAETLKFNMALVLLPVCRNTITWIRSKTQVGAVVPFNDNINFHKVIAAGVAVGVALHAGAHLTCDFPRLLHASDAQYELMKPFFGEKRPPNYWWFVKGTEGWTGVVMVVLMAIAFTLAQPWFRRNKLKDSNPLKKMTGFNAFWFTHHLFVIVYTLLFVHGTCLYLSRKWYKKTTWMYLAVPVVLYVSERILRLFRSHDAVGIQKVAVYPGNVLALYMSKPPGFRYRSGQYIFIKCTAVSPYEWHPFSITSAPGDDYLSVHIRTRGDWTSRLRTVFSEACRPPTEGESGLLRADLSKGITDEKARFPKLLVDGPYGAPAQDYREYDVLLLIGLGIGATPLISIVKDVLNHIQGEGSVGTTEPESSSKAKKKPFMTKRAYFYWVTREEGSFEWFRGVMNEVSEKDKDGVIELHNHCSSVYQEGDARSALIVMLQELQHAKKGVDILSGTSVKTHFARPNWRSVFKKVAVSHENQRVGVFYCGEPVLVPQLRQLSADFTHKTNTRFDFHKENF

>plant-Os-rboh-D: GenBank No. NP_001063267

MWTPSRGSNAARRSGHRRIADYLADDQTTNTDTSDNESYTTAYGDEFFAAAAAAAGSGGGGMLPAFLADQGDLVEVMLELDEESMVVRSVTPTSATLYGGGGGQMPQPLPPPLRTPEGGGGARSLSRCSSTSSRIRKKFAWLRSPSPSPSPRPPTPAELQREAAMAARERRRIQARLNRSSTGAKRALKGLRFISRTTGTVQAAELWRRVEDRFNALARDGLLSRDDFGECIGMVDSKEFAVGIFDALARRRRQNLERITREELYDFWLQISDQSFDARLQIFFDMVDTNVDGRITREEVQELIVLSASANKLAKLKEQAEEYASLIMEELDPENLGYIELWQLEALLLQRDSYMNYSRPLSTASGAQWSQNLGGAAVAAGAAAATGGGAHAAVAARGGQQQQQQQEGRRGGWGVRKAAARVRVAAEENWRRAWVVALWFAAMASLFVWKFVQYRRTPAFRVMGYCLPTAKGAAETLKLNMALVLLPVCRNTLTWLRSSWARFFVPFDDNITFHKMIATAIVVGITLHAGNHLACDFPRLIASGPEEYRLVADAFGPEKPTYVGLLSGVEGITGVAMVVLMTVSFTLATHPFRKGEKGGSGGGAAATVLPTVARLPSPFNRLAGFNAFWYSHHLLGIVYALLIAHGYFLFLVRRWYLKTTWMYISVPLMLYVGERMLRALRSNAYAVKILKVCLLPGNVLTITMSKPYGFRYRSGQYIFLQCPTISPFEWHPFSITSAPGDDYLSVHIRTNGDWTQELKRIFVENYFSPHLNRRASFSELGATEPRSLPRLLVDGPYGAPAQDFRNYDVLLLVGLGIGATPFISILRDLLNNIKLAEELMDLAMETSRSEDSANSFSVSTASSNKKRAYRTSRAHFYWVTREPLSFEWFKGVMNEVAEMDKKGVIELHNYLTSVYEERDARTTLLSMVQALNHAKHGVDIVSGTRVRTHFARPNWKEVFTRIASKHPNSTVGVFYCGAPTLAKELKTLSHEMSHRTGTRFHFHKEYF

>plant-Os-rboh-E: GenBank No. NP_001044725

MASPYDHQSPHAQHPSGLPRPPGAGAGAAAGGFARGLMKQPSRLASGVRQFASRVSMKVPEGVGGMRPGGGRMTRMQSSAQVGLRGLRFLDKTSGGKEGWKSVERRFDEMNRNGRLPKESFGKCIGMGDSKEFAGELFVALARRRNLEPEDGITKEQLKEFWEEMTDQNFDSRLRIFFDMCDKNGDGMLTEDEVKEVIILSASANKLAKLKGHAATYASLIMEELDPDDRGYIEIWQLETLLRGMVSAQAAPEKMKRTTSSLARTMIPSRYRSPLKRHVSRTVDFVHENWKRIWLVALWLAVNVGLFAYKFEQYERRAAFQVMGHCVCVAKGAAEVLKLNMALILLPVCRNTLTTLRSTALSHVIPFDDNINFHKVIAATIAAATAVHTLAHVTCDFPRLINCPSDKFMATLGPNFGYRQPTYADLLESAPGVTGILMIIIMSFSFTLATHSFRRSVVKLPSPLHHLAGFNAFWYAHHLLVLAYVLLVVHSYFIFLTREWYKKTTWMYLIVPVLFYACERTIRKVRENNYRVSIVKAAIYPGNVLSLHMKKPPGFKYKSGMYLFVKCPDVSPFEWHPFSITSAPGDDYLSVHIRTLGDWTTELRNLFGKACEAQVTSKKATLSRLETTVVADAQTEDTRFPKVLIDGPYGAPAQNYKKYDILLLIGLGIGATPFISILKDLLNNIKSNEEVESIHGSEIGSFKNNGPGRAYFYWVTREQGSFEWFKGVMNDVAESDHNNIIEMHNYLTSVYEEGDARSALIAMVQSLQHAKNGVDIVSGSRIRTHFARPNWRKVFSDLANAHKNSRIGVFYCGSPTLTKQLKDLSKEFSQTTTTRFHFHKENF

>plant-Os-rboh-F: GenBank No. NP_001055783

MAGDYVDVPLGGGGQSTLPPVAPLKKQPSRLASGMKRLASMVPDTMKLKRTHSSAQPALRGLRFLDKTSAGKDGWKNVEKRFDEMSADGRLPQESFAKCIGMADSKEFASEVFVALARRRSIKPEDGITKEQLKEFWEELTDQNFDSRLRIFFDMCDKNGDGQLTEDEVKEVIVLSAAANKLAKLKSHAATYASLIMEELDPDHRGYIEIWQLETLLRGMVTAQGPPEKVKLASASLARTMVPSSHRSPMQRRFNKTVDFIHENWKRIWVLSLWAILNIALFMYKFVQYSRRDAFQVMGYCVCIAKGAAETLKLNMAVILLPVCRNTLTRLRSTALSKVVPFDDNINFHKVIALTIAIGAATHTLAHVTCDFPRLVSCPRDKFEATLGPYFNYVQPTYSSLVASTPGWTGILMILIMSFSFTLATHSFRRSVVKLPSPLHHLAGFNAFWYAHHLLVIAYILLVLHSYFIFLTKQWYNRTTWMFLAVPVLFYSCERTIRRVRESSYGVTVIKAAIYPGNVLSIHMNKPSSFKYKSGMYMFVKCPDVSPFEWHPFSITSAPGDDYLSVHIRTLGDWTTELRNLFGKACEAQVSSKKATLARLETTIIADGLKEETCFPKVFIDGPFGAPAQNYKKYDILLLIGLGIGATPFISILKDLLNNIKSNGDVQSTHDAELGCTFKSNGPGRAYFYWVTREQGSFEWFKGVMNDVAESDHDNVIEMHNYLTSVYEEGDARSALIAMVQSLQHAKNGVDIVSGSKIRTHFARPNWRKVFSDLANAHQNSRIGVFYCGSPTLTKMLRDLSLEFSQTTTTRFHFHKENF

>plant-Os-rboh-G: GenBank No. NP_001066954

MASREESGNGGGGGATPAADYRSSDSRSSSRRSTRFKEDNEYVEITLDVKGDDTVAIQSIRNGADMPEVALLARGLAQQPPPSAAPGPGGLSSRLKAVRTELRRIASWKFPSGVLSGGGGGGDAPGNGNDRRPRLDRSMTGAARALRGLQFLNSSAVTNGWPEVEKRFERLAVDGFLLRSRFGQCIGMVGSEEFAVQIFDSLARRRGITAQLLTKDQLREFWEQLSDPGFDAKLQTFFDMVDKNADGQITEEELKEVLTLTASANKLSKILERVDEYTALIMEELDPDQLGYIDSTRVAVSAILNSDTVHGHLPFPKISNLESLLLLPPSQAPSKLVTHSSNISQLISQKLVPTHDRNPLRRGLRRLSYFMEDNWKRVWVMALWLAINAGLFTWKFMAYKRHPTFDVMGYCVCVAKGGAETTKFNMALILLPVCRNTITWLRSRTKLGAVIPFNDNINFHKVVAGGVVVGVALHGVTHLTCDFPRLLHASDAAYEPMKKYFGQTRIPDYWWFVRGVEGITGVIMVVLMAIAYTLAHPWFRRSKLSDSNPLKRLSGFNMFWYSHHLFVIVYIAFVVHGVCLYINRTWWKQTTWMYLAIPILLYAGERIFRALRSHGFTTVRIEKVAIYPGNVIAIHMTKPHGFKYKSGQYIYVNCGEICRPPMNGQSGLLRADCMSMEHHSRFPKLLIDGPYGAPAQDYWKYDVLLLIGLGIGATPLISIVKDVLNHIYDDPESAASPHTTNGGGAAAAARRAFMTKRVYFYWCTREEGSFEWFRGVMNEVADRDAGRELIELHNHCTSVYEEGDARSALVTMLQALHHAKNGVDVVSGTRVRTHFARPSWRDVFKRVAVNHQGQRVGVFFCGDQALTPELRRLAQDFSHKTTTKFVFHKENF

>plant-Os-rboh-H: GenBank No. NP_001061956

MWTPSRGSASGRRATGHRRIADYLADDRTEASTENGSFNTAYSDELFAPTSSSAGGDGVGGMLPAFLADQSDLVEVMLELDEESMVVRSVTPTTGALYGPTSLAGGGAAHTPPGSGRSLSRCSSTSSRIRKKFAWLRSPSPAPAPRAPTPSEPPPPREAAMAARERRRIQARLNRSRSGARRALKGLRFISRTTGSAEAAELWTRVEHRFNALSRDGLLSRDNFGDCIGKQAKPSSMSMARRARARSRDDTAYGAGIGAGMEDSKEFAVGIFDALARRRRQELERISKEELYDFWLIVLSASANKLSKLKEQAEEYASLIMEELDPEDLGYIELWQLEALLLQRDAYMNYSRPLSSGSTAQWSQNLGGGGGGQQGGQGQGQGQSEGRRNDWRRRWSPRRAAARAQVAAEENWRRAWVLALWFAAMAGLFAWKFVQYRRTPAFRVMGYCLPTAKGAAETLKLNMALVLLPVCRNTLTWLRSSWARFFVPFDDSITFHKVESSSGFKWAPPGRSNQCSVAGEPAGTEDPRPGRLRVKAPVIQKSPRPTGLPLSCGATIIATAIALGICTHAGTHLACDFPRLIGSSREEYELLLSGFFGASRPTYRGLLAGVEGVTGIVMVVLMVVSFTLATRPLRKREAPRLPFPLGHLAGFNAFWYSHHLLIVVYLLLLVHGWFMFLVTKWHQRTTWMYIAVPLMLYVGERTLRAFRSKAYAVKILKVCLLPGNVLTITMSKPYGFRYRSGQYIFLQCPTISPFEWHPFSITSAPGDDYISVHIQTRGDWTQELKRIFVENYFVPSVPRRASFGALGMAEQKSPPRLLVDGPYGAPAQDFRNYDVLLLVGLGIGATPFISILRDLLNNIKLADELMDLAMETSRSDDSANSFSVSTASSNKRRAYRTSRAHFYWVTREPGSFEWFKGVMNEVAEMDKKGVIELHNYLTSVYEERDARSTLLSMVQALNHAKHGVDIVSGTRVRTHFARPNWKEVFTRIASKHPNSTVGVFYCGKPTLAKELKKLSLDMSHKTTTRFHFHKEYF

>red alga-Cm-NoxD1: CMGP-database gnl|CMER|CMG089C (initially termed Cmrboh1)

MNKAKQNESNLDPSLTERTQIWNIHLVENGLWYSIVLLFFCAMSWFFAYGVRYVIVDLRPSGILRYAGSIARGMGFACTFCSLVLPLTINRTLATVLYRTPLYDLLCVERWIPDMHTIVGTSLAATGWIHGIAQIVNYAASVFVFRGGLFTGPAALPATMLFVTGVVLMVLLVPLLGFGVETVRRKWFRLFWWTHRPIAVLVYICLVFHGLRGGRPWTVYFFAGPVFLYIIDRLYQLSRATVTPRRILVLSLAQANSNIVKLSVERRNAVYLPGQYFKIMIIDTRKGCGMPADAWHPFTAASAPSCDSDRITFYIAAVGKWTRRLHSLAEAAVAEAATTTADGLDLAPERICMTTIEAYRVLLAGPYGAPAQSHLHFPYQLLIGSGVGATPMVSILREICSARAPAKLAGIADASDDDIRGIRAGTQHGTLDTLSKQETSAQLLDRIRKSGILSMYMKERVGIFQTETRIDRLRAFVLSSLWLFSLLWMLYISLTIYVIANAFDANVALVADFTSSAVLLFLFSFTIASDMLVTLDPPRWRYVGTPRGFVSLSVCGLLVADLVVGATLFGIEQHSSHYMDGAGNAMRIIALVLFCIKSIMFALTLRIFYLPRPWRIQLRRVRHLGRKASALLSRRGNGLGTAVAQQRCVQSDDKVSHVEQQEQQEQQEQQEQQEQQEQQEQQEQQEQQEQQEQQEQQEQQEQQEQRLYANVFSLTFVWVVREYADLWGIQGLFDLAQTWTPDLIPRVRVHIFLTRGEIPASILEAGQSTKSILQFHQGRPRFETFIEDLLDLRDPYEFERLFGNIIQVKQFNRVSGFSNDVQRGIFFCGNPALGASVRRAMHKVQRQPERLGTKKPILFIQERF

>red alga-Cm-NoxD2: CMGP-database gnl|CMER|CMR353C (initially termed Cmrboh2)

MQRRAAVDAQECHDTRFSPNSGGAFGRAPNLAAAAAIQSTPRHRRLSHVRIALVPRSFCFLDEVWMKPSLPPVPLGACAITTMRPRNRIRAQLDLWNERVGESGLFFSLLVAYWCSQAWFFVYGVKYALSLALPSDWSRYMLIFGRGFGYVATFNALLLPITVNRTLTNILYRLPLYDIFSVARWLPDLHAIIARWFTIAGWIHGILLSVAYGVGTLPFRGGFLPFSNTIPTTMVFVTGCALMAFLLAIVLLSLGSVRRRVYRVFWASHVPLAILTYAALVFHGLRGGRLWSVYFFGLPVVLYILDRLYHSFAAAFTPHRVLSVELSHKNSNVVRLVLERRGRKFVAGQYFKLAWKFHVCSHLAVAGEWHPFTVASSPLLDKDRIIFFIAATGKWTNALRALALQQHQQQHDENLEHASSTSPTPKSLATEAMPHAVDKSSITTCLPINRRWILLSGPYGAPAQSHQNFAHQLLIGTGVGASPMFSIVQELCGRATPCDNNNNKDASLECSDVSTGGDLENGTLASVSDTTSPTRESTDLPNFEKLKASTTANDSSSEVVPSVQALPPVLAARLGTLRGDTFLDRFSAAVLSNLWLFSILWICLADLTVSICASAFSHIVAFIGSLALFVVILTMICLTVIADMRLSVNPPRWHYVLLLRGILLLLWFAVALTNAVLCSLLIVEYFLASNDRMASISKSSEWLFVMVVTPVHITIFVLLLFFFFLPRSWTWRTTVTAKVFRRCLQGSWDHQQRSNKVVSDARNAEWNAFSHIRTVTFVWVVRYVEDLWFLEKLYELASQQDASDPCRPRLRIHVHITRLGHDDADPVSSRLPANCGEILQFHCGRPDFGEYVAELVNEDHDTTTPLAGRGKPGAPASIRRFQRTRAFSNKVHHGIFFCGGRSVVRSVHQSIRAVAAARAARGLPQRNVFFMKENF

>fungus-Cc-NoxA: Broad Institute FGI database: CC1G_10525.1

MAETWFQREFLQARRLTFNVLFYGLHLAIFAYGWYSQATNQRLAGLNTLQWSVWVSRGAGLVLALDGGLILIPMLRNIIRVVRPKLTWLFPADENIWFHRQVAYSMAFWSVVHTTAHYVNFINVERTQIRKQTAQMIHYTQPGGITGHFMLLIMVVMYTTAHQKIRKQCFEAFWYTHHLAFFFMLGLYTHATGCFVRDSVHPNLTSTFPFYSTENCIGYLSWRFIIWPGIIYFGERVWREIRARRATRLSKVLVHPSGAMELRIVKPFKYTAGQWLFLQVPELSRFQWHPFTITSAPEDPYVSVHIRQVGDFTNALGERLGVGPAAVASMTKAAVKGAEKDGPSLSRGDFVEIDPATMSITLPQVRIDGPYGAPAEDVFNNEVAVLVGAGIGVTPFASILKHIWYRQKKGKLGSLRRVEFFWVCRDAPSFGWFQSLLQEVEAAQADPNFLRINIYLTQKINEDMLWNIAVNDAGADYDPLTLLRSRTMFGRPDWNSIYSRIRQAVEGGQYIPGATAQLKTKVGTYFCGPSVLAKAIKEATIKNTNANVEFSFAKEHF

>fungus-Cc-NoxB :Broad Institute FGI database CC1G_09830.1)

MAFRNSGFDFSDGKVTGPNATIAQHPDLADKLSTGSNQPVHLQRNKTERRRLQGLQRTNTFTAAPRTTQGEMGWKQRWDVWMINEGGRQLFFGTWIFLHLLVAVFGFMHYQLKDNSEGARSVFGITFPIARTAALVLHVDVIFILLPVCRNFISLLRRTPLNDIIPFDKNITFHKATAWSIVIGSAVHTLAHMVNFTKLALSIPNMTTGQRIGAFMAANFATGPGLTGWIMWLALGIMVWFAIEKRRRAHFERFWYSHHLFIVFFINWQLHGMFCMIKPDRPPYCSFNTIGVFWRYWLVGGVIWIWERILREIRSRHRTHIHKVIQHPSSVMEVQIKKEKTTTRAGQYIFLSCPEISYFQWHPFTLTSAPEEDFISVHIRVAGDWTTAFSKALGCDFERKKKGDDGAVAKPAAVPPPINRPLPRVMVDGPFGSASEDFLKYETVLLVGGGIGVTPFASILKHIWYRMNNLTDAKPTRLSKVYFTWVIKDFGTAEWFHSLLHAIEEEDTQNRIEINIYLTAKLKEDEVNNIIVQDVGAEKDAITSLRAPTHFGRPNWDRVFGSLCEKHPETDVGVFFCGPAPLSKTLHSMCNKYSTPKGTRFFYGKENF

>fungus-Lb-NoxA: JGI database jgi|Lacbi1|243586|e_gww1.1.1127.1

MGESWFRREFMVPRRLAFNVLFYGAHLSLFIYGWYSQATNQRLAGLNSLKYSVWTSRGAGLVLAFDGGLILLPMLRNIIRIIRPKLTWAFPADENIWFHRQVAYSLAFWAMVHTTAHYVNFINVERTQIRKQTALQIHYTQPGGITGHFMLLIMVLMYSTAHQKIRQQCFEAFWYTHHLAFFFMLGLYTHATGCFVRDTVDPDYIPTFPFYSTEHCLGYLSWRFIIWPGIIYFGERVWREIRARRATRLSKVLVHPSGAMELRIIKPSFKYTAGQWLFIQIPELSRFQWHPFTITSAPEDPYVSIHIRQVGDFTRGLGDRLGVGPSDKMSGMSRGDYVELDPAGSSIALPSVRIDGPYGAPAEDVFGAEVAILIGAGIGVTPFASILKHIWYRQKKGKLGSLRRVEFFWVCRDAPSFGWFQSLLQEVEAAQADRELHVLAANFLRINVYLTQKISEDMLWNIAVNDAGAEYDPLTLLRSRTMFGRPDWMTIFGQMKQAIEGGQYLPGSTSQLKTKVATYFCGPGALAKAIKDATVSHTNSNVEFTFAKEHF

>fungus-Lb-NoxB: JGI database jgi|Lacbi1|178541|estExt_Genewise1_worm.C_70328

MKKPVEPKTLREKFSIWMINEGGRQLFFGTWIFLHLLVAVFGFMNYQLKDNLVDARALFGVTFTIARTAALILHIDVIFILLPVCRNFVSLLRRTPLNDIIPFDKNITLHKATAWSIVAGTVVHVVAHMVNFYKLAMADTDAKTTGQRVLAFLEANFNTGPGVTGWIMTVSLGIMVFFASEKRRRAHFERFWYSHHLFVVFFINWQLHGMFCMIKPDRPPYCSSNTIGVFWRYWLIGGVIWISERVLREIRSRHRTYVSKVIQHPSNVMELQIKKEKTTTRAGQYIFLSCPEISYFQWHPFTLTSAPEEDYISVHIRVVGDFTQALAKAMGCDFEKKGKEETPAGGKVVGTNVNPSVNRLLPRVMVDGPFGSASEDFLKYETVLLVGAGIGVTPFASILKSIWYRMNNFNNSKPTRLSKVYFTWVIRDFGVAEWFHSLLHAIEEQDTQGRIEINIYLTAKIKEDDMNNIILQDVGAEKDAITSLRAPTHFGRPNWDRVFGSIAEKHPETDVGVFFCGPAVLSKQLHQMSNKYSDPKGTRFFFGKENF

>fungus-Pp-NoxA: JGI database jgi|Pospl1|109236|estExt_Genewise1Plus.C_480175

MGESWFRREFLAPRRLIFNVLFFGLQVSFFSYGWWAQATNNKLAALNALQWSVWVSRGAGLVLAFLAACLLLPMLRNCIRVIRPKVAFLFPADENIWFHRQAAYCLAFWSMVHTTAHYVNFINVERTQVRQEYALQIHYTQAGGITGHFMLLCMVLMYTTAHHKVRQQCFEAFWYTHHLAFFFFIALWTHADGCFVRDSTNAAYIDTFPFYNAEFCLGYESWRFTIWPGIIYFGERVWREVRARRATRLSKVLVHPSGAMELRIVKPSFKYVAGQWLFIQVPEVSKFQWHPFTITSAPEDPYVSVHIRQVGDFTQALGDRVGAGPSVVAAMTKAAMIGSEKDDSVYGTRGDFVELDSGARPLPTVRIDGPYGAPAEDVFNVEVAVLVGAGIGVTPFASILKHIWYRQKKGNLQSLKRVEFFWVCRDAPSFGWFQTLLQEVEEAQVDPNFLRINIYLTQKIGEDMLWNIALNDAGAEYDPLTLLRTRTMFGRPDWKNIYARMKQAIEMGQYLPGTKEQLKTKVGTYFCGPGVMAKAIKEACVASTTPNINFTFAKEHF

>fungus-Pp-NoxB :JGI database jgi|Pospl1|108206|estExt_fgenesh3_pm.C_1080006

MAANWTKRSSDLDFSDGKLPLAQHPDMDNPFADQGMQPGHLQRNKSERRRLQPLQRSNTVGTIASTPYKPETWIDRWRLWMINEGGKRLFFTFFILLHVLVIVFGFLYYDTSDDLKGARKTFGITYPIARSAALVLHVEVAFILLPICRNFISLLRRTPLNSYIPFDKNITFHKAVAWGICLFTFIHIAAHMVNFARLAFADPLAKTPGERFVVWLIANFTTGPGVTGWIMTAALAIMVWYAVEKRRRANFEKFWYTHHLFIVFFLNWQLHGMFCMIQPDREPFCTWNSIGVFWRYWLTGGVIFTFERILREVRSRHRTYISKVIQHPSNVMELQIKKEKTTTRAGQYIFLSCPEISYFQWHPFTLTSAPEEDYISVHIRIVGDFTRELAAAVGCDFDSKEKGVEAGGKLIGTNTNPPVNRVLPRVMVDGPFGSASEDFLNYETVLLVGAGIGVTPFASILKSIWYRMNNLNNSKPTRLSKVYFTWVIRDFGSAEWFHSLLQAIEEQDTQNRIEINIYLTAKIKEDDMNNIIVQDVGAEKDAITSLRAPTHYGRPNWDRVFSSIAEKHPETDVGVFFCGPAVLSKQLHISCNKYSSPSGTKFFFGKENF

>fungus-Pg-NoxA : Broad Institute FGI database PGTG_01933.2

MTDCEKCYTCAIQDNKLKELHRGLEGAPESEVEQCHWPNKASGLVSHRSNGIGVPGSHEDGSLKGFHVCMGRRLMPLSRWVRTFYTSLQKPYAPFKTEDRMARLSRQVAYTTLFFTAIHTTAHYVNMFHVETTQIRPEKAIEIMYSETGPLTGHIMLFIMVLMYTTASTKIRTQCFEAFWYTHHLAFFWALCLYTHAAGCFVRGALPDHKAQCLGYNSVYVTVWSGLAYFCDRVFREIRGRGRSEISAVLIHPSGTVEIRMLKAGFKYVPGQWIFFQMPEVSRFQWHPFTISSAPDDPYISIHVRQVGDFTKAVGTRLGATPQLMATLNQPSEFSVEDCGEFHDITTIRSRDLPLVRIDGPYGSPAQDVFKCEVAILIGAGIGVTPFSSILKNIYYMQAQGKLGLLRKVQFIWINKEITSFSWFKTLLKNLEDIQHDYSFLRMDMYLTGSMDEDTISNVVLNTGAHKFDSLTGLKSQTHFGKPHWKKDVFDPIRKAIHSGDWYERDISGTTKVGCFYCGPRPLAKTLEQECRQATTDKVKFEFHKERSVDSSTFMNPLECA

>fungus-Pg-NoxB : Broad Institute FGI database PGTG_04320.2

MAPPRGSAGAPSRLGIGNERRQYEKEFYNPEFEESQVPLTPVDRRSPIVANFTAPRFNRNLTERVRLQGLMGGSDLSSRKGDSEFSDDNRLSFSSNGPVPQLPRSTTKFDVERGLILTSFTTRFKSWMVNEGSRKFFVWTWIFIHIITFVFAFLNFLLKDNLTQARATFNITYPIARASALVLHVDVAFILLPICRNFITLLRRSALNQVIPFEKNITFHKFTGFALAFFSAIHILAHMVNFGQLAVRTQTGIVGFIGANFLTGPGATGWIMTLSLGIIVWYAREKPRRAKFERFWYSHHLFIVFFSAWQLHGMFCMIQPDRPPYCSFNQIGVFWKYWLVGGTIFIWERVLREVRSRHKTYISKVIQHPSNVCEVQIKKEKTTTRAGQYIFLNCPEVSYWQWHPFTLTSAPEEDYISVHIRCVGDFTMEFAEALGCDFSRNKEKSNAGRPTVLPPATNRVLPRVMVDGPFGSASEDVFKFEVVMLVGGGIGVTPFASVLKSIWYKLNFPSASKQGSPIRLQKVYFFWVCRDFDSFEWFKSLLSAIEEQDVDRRVELHTYITQKLKDDDINNIIVSDVGGNRDAITQLRSPTHYGRPNWDRIFNSVRETHPATDVGVFFCGPGPLGHSLHLQCNKWTGGTDNDTRFFWGKENF

>fungus-Bd-NoxA: Broad Institute FGI database: BDEG_06864.1

MVEITARLVAFHVLFWGFHIALFVYGFFKQKNDPELQVLNSIGPSVTISRGAGLVLGVDCAALLIPVCRNIVRFFRSSFLNKYIPIDSNLYFHKWLAYSMLFFSLLHTNAHYTNFFFVETKLPSLGLKAWMIHYLAWSGATGHIMLIIMFFMYTSAKSDVKTKNFEYFWYTHHLFVPFYFCLFFHSFGCFVKSSTTGKCKGYNTNYGTVPIFCVYIAERLLREYRARLPTVLSKVIFHSGNTMELRIEKPSFQYMPGQYLFLNIPSISAFQWHPFTISSSPEEGFVSIHIRIVGDWTKNAAKMLGCYEQDIEKRMDLPEIRIDGPYGAPAEDLYNYKVAVLVGAGIGVTPAASLLKSVWYRYYRKASMPLKKVYFVWINRDKEAFGWFQSLLASLEETIPRSFLEIHTYLTGNLAVDDIQNILLNSDMDVDPLTELQSRTHYGRPAWSQLLNGIKLNVADVRDPQVEVGIFYCGPGALAKVIKKHADQASDNRVKFVLRKEHF

>fungus-Bd-NoxB: Broad Institute FGI database BDEG_07125.1

MPNSRPQQTSFQNELSLGAVRPRQRKSGWMVTFNNWMVNEGRSRLFFALFIASQLGYFAWSYYQLWTSPTLVTFRTVLQHGLPMARAAANVINLDCGIILFTFDKNITFHIWIAYSIAFWTFVHVIAHYFNYNNVRIALGVSAEYLSLVSGPGLTGQVISVSFFLIFTSAMEAVRRKYFEIFWFTHHLFLVFFGALLMHGSFCFIKGDSGDPCRGGPMFWKFWVGSAAFYLIERLWREISGRRKTYIFKVVQHPSKVVEVQIKKNGWKMQAGQYIFICCPEIGLFEWHPFTLTSSPHEEFLSIHIRVVGDWTEKFAERVGCRFGGSSDNMPAPDTLPYVMVDGPYGSASEDVFDYEAAVLVGAGIGVTPFASILKTIWFRINNPTRAVPLKKVYFFWICRDKDAFEWFQDLLSTIEDENISNFLEIHTYLTQKLKISEVKNIVINDGEDGRDAITGLKSRTQYGRPNWDQIFEALRVKHRATDIGVFFCGPKVLSRTLHHTCNKWTEATEDGTRFYYGKENF

>moss-Pp-rboh-A: JGI database jgi|Phypa1_1|146280|e_gw1.241.71.1

MSDSKEFASELFDALVRRKGIEVESITKDELYEYWLQVADRSFDARMQIFLGLCDKDLDGRITGEEVKQVIMLSASANKLSKLKDQAAEYAALIMEELDVDRVGYIELSQLETLMRQSILGLGREVTVNYTHVLTTRTERSKIIKLAEKCVHFFLDNWKRIWILALWMSVMAALFAWKFLQYRDRSSWWIMGDCLCVAKGAAETLKLNMALILLPVCRNTLTRLRSTRLWKIIPFDDNLDFHKASQIVAGGMAAGVFLHVGCHATCDLPRFVNADKETFFKHLGDHFDTQPTYSDILNMSVGYSGVIMLVIMIIAFLLATHWFRRSLVKLPWPFHRLMGFNAFWYSHHLFVIVYALLLLHGTKLLLPSPWHERTTWMYIAVPLLLYAGERLLRMYRTNSSKVDVIKAAIYSGNVLAIHMSKPEGFKYKSGMYLFLQCPEISSFEWHPFSITSAPEDPFLSVHIRTLGDWTGEMMKIFADACGGRMRLQTVNNYGFILVGHLTYRSCCHRFPKLYIDGPYGAPAQDYLKYDVLLLVGLGIGATPFISILKDMLHHTRNDSSGHSYPVLTTKAKRKPKAYFYWITREQGSFDWFRGVMREVEEIDNKRSIEMHNYLTSVYEEGDARSTLVMMLQALHHAKNGVDLVSGTRARTHFARPNWKSVFSGLTATHQDKRIGVFYCGPAALANELENLSRSYTQTSSTKFSFHKENF

>moss-Pp-rboh-B: JGI database jgi|Phypa1_1|66252|fgenesh1_pg.scaffold_10000011

MKESKEFALELFDALVRRKGEKVNSISKDGLYQFWLEITDKSFDARMQMFFDLCDKDLDGRISGEEVKQVIMLSASANKLSKLKEQAAEYAALIMEELDVNRNGYIELSHLETLLRESVLPGHGKEATMNNHQLLIKRSKRSRIRSFAEKTRNCFQDNWKRLWILALWMMAMTGLFIWKFFQYREHAAFPIMGNCLCVAKGAAETLKLNMALVLLPVCRNTLTRLRSTRLGKIIPFDDNLDFHKETGGIAAGVFIHGVCHITCDIPKFVESGDDKFFKYLGDEFEKHPTYANIAVMPVAITGILMVVFMIIAFLLATHWFRRSMVKLPWPLQRLTGFNAFWYSHHLFVIVYALLMVHSIKLLLAGPWYKRTTWMYIAIPLLLYAGERMLRLYRTNSSKVEIVKAAIYTGNVLAIHITKPQGFKYKSGMYLFLQCPQISSFEWHPFSITSAPGDPFLSVHIRTLGDWTAEMKKIFSEACGGRTCLQTINNYGLSGELTLAARFPKLFVDGPYGAPAQDYLKYDVLLLVGLGIGATPFISILKDMLHHTRYDLSDPVMKPCDRESSYFYWVTREQGSFDWFRGIMREVEEIDNKESIEMHNYLTSVYEEGDARSTLVTMLQSLYHAKNGVDLVSGTRARTHFARPNWKDVFANLTFTHPEKRIGVFYCGPAALVNELETLSRTYTKESSTKFSFHKENF

>moss-Pp-rboh-C: JGI database jgi|Phypa1_1|206166|estExt_Genewise1.C_320034

MLHRSDFAECIGMKDSKEFANELFDALVRRQGVEVESIGKDELYLHWLQIADKSFDARMQLFFDLCDKDLDGRITGEEVKQLIMLSASANKLSKLKDQAAEYAALIMEGLHVDGAEYITLSQLKTLMSGSVQGFGRETTVNNNQTLTPKSKRSRMMGVAEKSAYFLLDNCKRIWILALWISVMVGLFAWKFLQYRNRSAWWVMGDCLCVAKGAAETLKLNMALILLPVCRNTITRLRSTRLRRIIPFDDNLDFHKIVAGGIAAGVILHATCHITCDIPRFVEADKEKFFKYLGDDFDFQPTYSDILRMSVGYSGLIMVVLMILAFLLATHWFRQSLVKLPWPFHRLTGFNAFWYSHHLFAIVYAFLLLHGSKLLLPNSILERSTWIYIAVPLVLYAGERFLRMYRTNSSKVDVIKAAIYTGNVLAIHMSKPEGFKYKSGMYLFLQCPEISSFEWHPFSITSAPEDPFLSVHIRTLGDWTAELKRIFSDACGGRMRLQTVNNYGLSGELTLAARFPKLYIDGPYGAPAQDYLNYDVLLLVGLGIGATPFISILKDLLHHTINESLGHSDPSLTPDLRPMESPRTRSKKKAKRNPKAYFYWMTREQGSFDWFRGVMREVEEIDHKGSIEMHNYLTSVYEEGDARSTLVIMLQALHHAKNGVDLVSGTRARTHFARPNWKSVFSKLAATHQEKRIGVFYCGPVTLANELENLSRTYTQKSSTKFSFHKENF

>moss-Pp-rboh-D: JGI database jgi|Phypa1_1|204103|estExt_Genewise1.C_180103

MQKSTSRAEYALQAVRCISKATATDDEKKLWEKVEQRFVKLADADSMLPRSNFAECIGMKESKEFANELFDALVRCKGEKVNSISKQELYQYWLVIANKSFDGRMQMFFALCDKDLDGRITAEEVKQVIVLSASANKLSVLKEQASEYAALIMEELDVNRNGYIELSEFESLMRGSVMAANGKEPTVQYNQMLKQKSKRTKVQDATGKAKHFLHDYWKRLWILALWVMAMTGLFTWKFLQYRHHEAYAVMGECLCVAKGAAETLKLNMALILLPVCRNMLTRLRSTRLGKLIPFDENLDFHKASLLPDPEHCIAAAIAVGVFVHGTLHITCDIPKFVNCDDEKFFEALGDQFDKHPTYADIAVTPVAITGILMVVLMAIAMLLATHWIRRSLVKFPWPLHRLTGFNTFWYSHHLFVIVYALLLVHSIKLLLAGSWYKRTIWMYTAVPLVLYASERFLRLYRTNYSKVEVVKAAVYTGNVLAIHMTKPAGFKYKSGMYLFLQCPAISSFEWHPFSITSAPDDPFLSVHIRTLGDWTTEMRKIFSDSLGGKTRLQAINDYGLSGELTLAPRFPKLYIDGPYGAPAQDYLKYDVLLLVGLGIGATPFISILKDMLHHSRNDSVSDLSSSPDLNPTGSPPPKKKAKRDPKAYFYWVTREQGSFDWFRGIMREVEEIDNKELIEMHNYLTSVYEEGDARSTLVTMLQSLHHAKNGVDVVSGTRARTHFARPNWKNVFTNMADTHPNKRIGVFYCGPASLVNELKTLSKAFTKKSTKFFFHKENF

>diatom-Pt-NoxD: JGI database jgi|Phatr2|35161|fgenesh1_pg.C_chr_7000082

MVTVKASSRPTTSSPADHHHVSRWAKSEAFFFNQGANLAFLGLMLALNILMLVWGTYEFTGTRFVTDSDILRVTLPIARAGGRVVTWNAALIFLSGSKYLWTWLRQSPLHLGFPIDNVMPYYHKMIAWTIIFMGCVVHTIPQVINYATKELRIEGGPIWLWGDGLATKQLLVTGIFLFWIFAGFFMTTLERVRRTTWGFRLFWVAHMISITCVLPLLIIHGTIRGKPILMYTASLPIAIYIVDSFMRRMMYKTVQAGVVRFQAFGGEGQGGGEKVVELILRSSAYKYRPGQYAEIQIPELSRYEWHPFTIASAPNSDNTVSFCIKALGRWTNGLFDLADTFQGGDVEEPKAPLSMTVNLRGPFGAPAQNYLNYRHLVVIGSGIGVTPLLSVWAYLVRATKGLVKKVPPPSTSEFTAPSSSFISEDMDLPFDEVAEDRLLACVDVNAVDVIEMDRPFKTLRGKLAYYASTLETMTVNICLFLFSLFVEVTVFSVWLFGFGKEAALIQIIASTIALIIFGSKVALSLVVYGGRYVRSLVFALEMGIVLLDGAALATAVSTRHSPSKPEAISYFVFYGAFIALHSVRIFHIFYATARPPHTEEPTRGVDAIHSVTGIWVAKKYEDMSFAAPSLVRTLPGLSKVFSLRLFATRDKEEDLVHRNPLADHGPDHILTAGRPDWDALLAEALEKAHTSHPEGDAVGVFFCGAPAIARTLQRTAHKVTAQHHYTVRRATGTACRCRVLVHKENF

>oomycete-Ps-rboh-A: JGI database jgi|Physo1_1|142989|estExt_fgenesh1_pg.C_1260035

MQTDYAILESNTPVECTQIHHHDFEAEKKTLQKLPPTLPMPKILGSVIRSSPPNVETRPRASSLVAMLGGSGAKTAMGNSRLATSRVGELNTSHSSCLSSGSVLNMSSHPSMTSVSNISRPSRLMNKAELHDLKDQLWQGSMSGGLSKAMDASAVSSDDCANPRAAGQLSFFTQAFNASSLPGSTHAERTSMAFIPLPRESVAMMVDAVRVSALEDDEKCRFLFEMFDVEHRGVLSKEGVRAFVEATFATNGVEFLGAFDYDAVVGKVFGRCRQQDKMSYSEFKSVFGAVVAESDDDKSKEALGRLTVIVQTHNPKEFVPNTTTTNNNNNERGGRWYRINKFCRRYKAEIFWLTLYFLLMVAVFIAKASRFAFDPAVGNCPRVAKGFAEICLVNTMFVLLPMCRNFVTGLRTLPVVVNHLPIDHHIEFHKICGVVLLLAFLGHTAAWLVIVVYVRTVPLAVWKQSRYHHLAFVRDENLLLFALRVPIWTGVAMLLCAAIAAPLCLEKVRRGKFNLFWVSHMLFIPFLVLMAFHGFARWVAAPQAHYWVLPPIVIYLVEKRYRMTQMFGGQTQIAHVQLSKEAVAVFMRKPKAFSKRQRFLPGMYVFVNVPTISKFEWHPFTISSAPEDKFISLHIQRSGDWTRALYDNLQQHQASRVEDQGSPVTSPYPTVFLDGPIGAPAQDYSRYREVVLIGAGIGVTPFASILRSIMHQWESYRCPQCGHVRFPPSFQLRKIYFYWVMREQEALTWFTNTMNQLSQMDADNRLEIHNFFSSVKNEEVIAPLQALQNFIHDTEGQDIISGLQTRQRTRFGRPDWNAELSRVAQNHRRLEPLEDGDGEREEIGVFFCGPKPLGNVIDEQCARLNQTVPDVEFAFHSENF

>oomycete-Ps-rboh-B: JGI database jgi|Physo1_1|134197|estExt_fgenesh1_pg.C_300108

MDRQNRGQVEGGAAARQPADELKITPGQNLQPFVDISTPSPHARQFQHKPNSKSSNSRSGSRSNSRVNGRSNGRSNSKRGSMKDKRVPGPPPPPPPPSFGHPSIGSSHPSMVSSFRSTAKSNFMDSSQFINPSATNTHIIGCGPPKMSANCKSSRGPRRRPNVQAMLGASMVRQSLARASNLSSFAGSRYGPQPGSDVVSVPVLGDCNFEQKYTVAMSMDEHAMHANDPSYTPMEKLHALRDVMSSITSKGGYIERETFSQTFELNGDDFDGYATGNGAIDGNAILIDAVMDLNVDGEEKLRFIFETLDPDNSGYVIEDQIVQLLESNFSAAHLDVVGTDFKTVAKLMFRKAQVKNESMTYEQFKTVFAPYINDSYNLRKSAPVYTAPIRPKSKFGAWYSANKLRIWWLLFYAILNNIAFWVKWFSYDVDPAIGWGLRIARANAQVAMVNCVFVLLPMCRSITQVMKRSRILWRYIPFDDSIAFHKIAGSVLLTAGLIHTIAHVFNEIYLYLVATPEEIKRSIFVTRHVSSFVNGERPPFITMLQSLPVWTGVILLVITCISFPLAAIPKFRQGKFNVFWYSHMLFGPFLLVLCFHGACSWLARSSSYIWITPPFLIYLIERRFRYAKMFAAPVRIMEAMELDGTVALFMEKPRRFVYRPGMYMFVNCPLISSHEWHPFTISSAPGDNYISVHIRVCGDWTQALARVIADCHERKVLYPDIHLDGPVGAPTQDYHRYKTVICVGGGIGVTPFASILKDVVHLWEANRCPNCSHVRHPGSFKIQKLYFHWVTRGQESLSWFEETMNQIAEMDRDNVIETHQYLSTLKGSENTSQLKMFQEFVHEQTGKDFVSGLSTKQLTHFGRPDWDKVFSEAKANHPGEEVGVFYCGPHALEEILDNTCKRYSSSDPNGTIFDFHSEKFS

>oomycete-Ps-rboh-C: JGI database jgi|Physo1_1|134195|estExt_fgenesh1_pg.C_300106

MDRSRKEPGRGKPKNDLKITPAKTQALIDVSTPSPNAPRFGDKSTPMMSTMVNMNQNPPLRKPATANFMD

SSKFMSMNSTTTPAVGNCRGSGNNRRSHRRRANVDAMMRSNMVRQSLTLSASSVSMTSTGPRMKCRVAPP

APKDDMYGPPPGSDVVTVPIPGDCNFEQKYNVAMSMDEKAYTPMEKLEALRDAMGNITDKDGYIERETFS

HAFEVDGNDFDGYATSNGTIDGNAILIDAVMKLGVNAEQKLRFIFDTLDPDNTGYVVEDQIVQLLESNFS

SARIDVVGMKFQTVAKLMFRKARVQNDAMTFEQFCNVFEPYVTDSYHLEEAKPVYSAPIRPKSKFGQWYS

DNKLRIWWLLFYFIVNNIAFWVKWFMYEVDPAIGWGLRIARANAQVAMLNCVFVLLPMCRSITQVMKRSK

FLWRIIPFDDHIAFHKISGSVLLIAGLIHTIAHVFNEIYLYLIATPDEVKRSIFVTRHVSTFVNGERPPF

TTMLQALPVWTGVILLVITCISFPLAAIPKFRQGKFNLFWYSHMLFGPFLVVLAFHGATSWLARCSAYIW

ITPPFLMYLIERRFRYAKMFAAPVRIMEAMELDSTVALFIEKPRRFVYRPGMYLYINCPQISSHEWHPFT

ISSAPGDNYISIHIRVCGDWTDAMARVIADCHACNLQYPDVYLDGPVGAPTQDYHRYKTVICIGGGIGVT

PFASILKDVVHLWEDNRCLNCNHVRHPSSFQIQKLYFHWVTRGQESLSWFEGTMKQIAQMDRDNVIETHQ

YLSTVVLGGEENTSQLKMFQEFVHEQTGKDFVSGMDTKQLTHFGRPDWERVFSDASAKHPGEEVGVFCCG

PHELEEVLANVCKKYTSSKADGTVFDFHSEKFA

>amoeboflagellate-Ng-NoxA: JGI database jgi|Naegr1|80514|estExt_fgeneshNG_pg.C_380016

MGALSNCCDFFFYGCFANGFSSESISSVRKFLLKWKKRFTNRHEFMNYWSAEGQKLVVVLIWVILNLFLFTEAFVRYYYALLLVNITGVTVVPFYVALARGFGQLLNFNCALLILPTMRTLLNVFRSLKFGSIFPLDKNLVFHRYLAYWIVVCVIGHTLGHYLNYACCWQLYNSARTPLEACWWNKYGTTGNVLCIVMFIMYAASAKSYRRTKNFTVFWYAHHLFVVFFVLLLVHGKQFWAWFFIPMALYALERVMRNLRGSEMTIVKRVHCLASRVIHLELEKPSFRYESGQYCFLNCPMISQHEWHPFTISSAPEEEFLQFHIRCVGDWTNTLMDVFNPAQRPTVEINKPTTPDGTDYLIRVDGPFGTCAEYCFDFEYVMLIAAGIGVTPYSSLLKHFKFRLDAAASGQAPPLKIRRASFYWINRDEGSWEWFSDILNQLETENPEFFDIHTYMTGMLKADDVKKIMFTSSEYQTQSNTAGNVQQSGKVDFMVRALHKYDAQSSDEISLDRNDIVVVSEQDESGWWTGTNTTTKQTGLFPGNFVVKVDHVTKMADSSKRNFGRPNWDAEFSDIRRYVEKNSSGAKKPNVGVFVCGPGGLSKQVYSYAVDKSKNSTVQFVFHKENF
