# Supplementary material for: Molecular evolution of Phox-related regulatory subunits for NADPH oxidase enzymes
Source: BMC Evol Biol. 2007 Sep 27;7:178. doi: 10.1186/1471-2148-7-178 (PMC2121648; doi:10.1186/1471-2148-7-178)
Supplement: Additional file 6 — Molecular taxonomy of Nox and Duox proteins containing newly identified sequences. molecular taxonomy built by amino acid sequences of Nox and Duox proteins of D. pulex, L. gigantea, C.briggsae, N. vectensis, M. brevicollis, C. intestinalis, S. purpuratus, and H. sapiens is shown. [file 1471-2148-7-178-S6.pdf]

Additional File 6

Molecular taxonomy of Nox domains of the selected Nox and Duox protein sequences containing newly identified sequences. The following naming was used to describe species of the sequences: *Caenorhabditis briggsae* (nemotaode-Cb), *Daphnia pulex* (waterflea-Dp), *Lottia gigantea* (gastropod snail-limpet-Lg), *Homo sapiens* (human-Hs), *Strongylocentrotus purpuratus* (sea urchin-Sp), *Ciona intestinalis* (ascidian-Ci), *Nematostella vectensis* (sea anemone-Nv), *Monosiga brevicollis* (choanoflagellate-Mb), *Magnaporthe grisea* (fungus-Mg), *Fusarium graminearum* (fungus-Fg), *Coprinopsis cinerea* (fungus-Cc), *Laccaria bicolor* (fungus-Lb), *Postia placenta* (fungus-Pp), *Puccinia graminis* (fungus-Pg), *Phycomyces blakesleeanus* (fungus-Pb), *Batrachochytrium dendrobatidis* (fungus-Bd), *Dictyostelium discoideum* (slime mold amoeba-Dd), *Arabidopsis thaliana* (plant-At), *Oryza sativa* (plant-Os), *Physcomitrella patens* (moss-Pp), *Cyanidioschyzon merolae* (red alga-Cm), *Phytophthora sojae* (oomycete-Ps), *Phaeodactylum tricornutum* (diatom-Pt), *Naegleria gruberi* (amoeboflagellate-Ng). Two Fre genes (*Aspergillus nidulans*-An and *Saccharomyces cerevisiae*-Sc) are used as references. The An-Fre1 protein (GenBank No. AAN61438) is defined as an out-group. Evolutionary distances (inferior bar) are equivalent to 0.1 amino acid substitution per site.

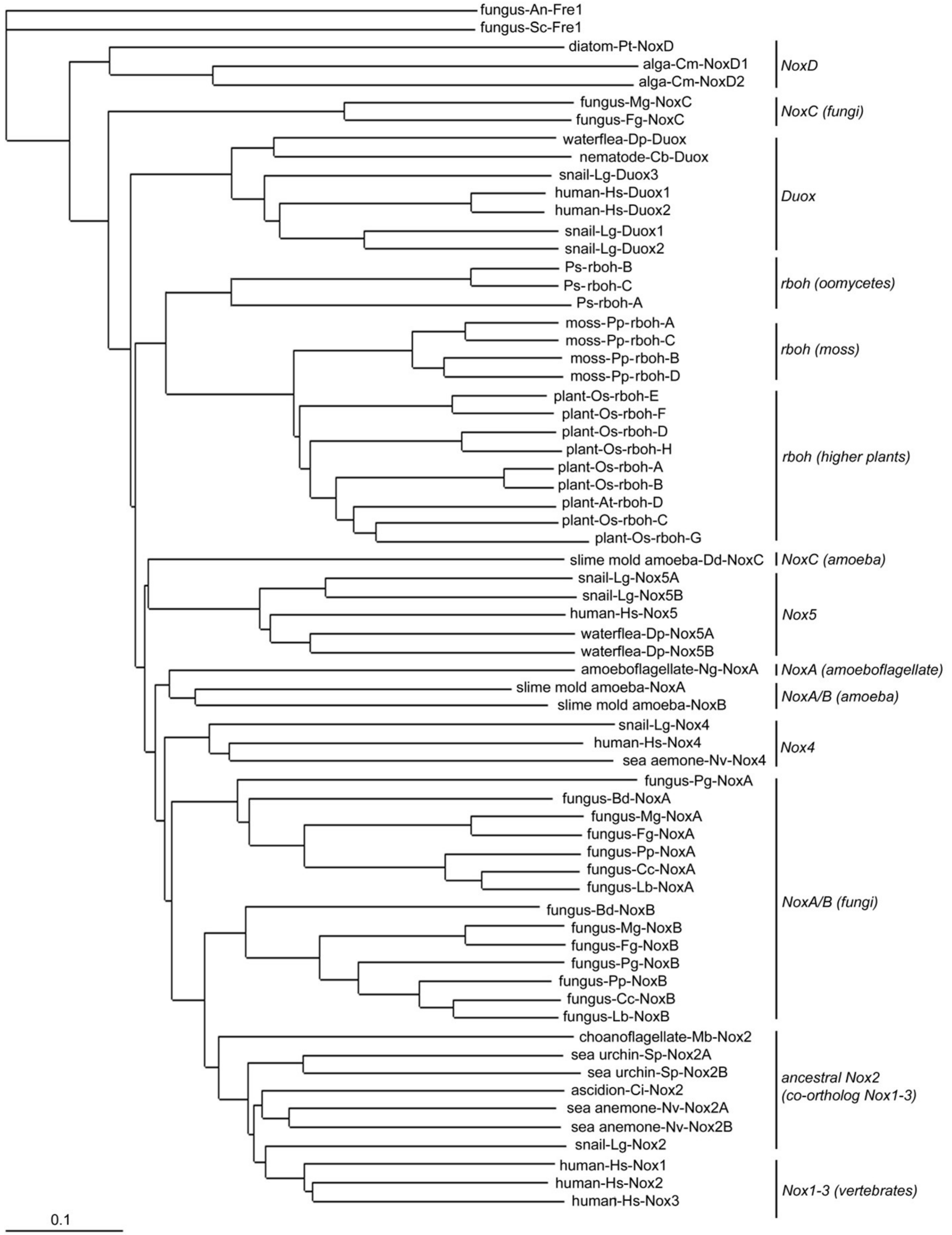
fungus-An-Fre1
fungus-Sc-Fre1
diatom-Pt-NoxD
alga-Cm-NoxD1
alga-Cm-NoxD2
NoxD
fungus-Mg-NoxC
fungus-Fg-NoxC
NoxC (fungi)
waterflea-Dp-Duox
nematode-Cb-Duox
snail-Lg-Duox3
human-Hs-Duox1
human-Hs-Duox2
snail-Lg-Duox1
snail-Lg-Duox2
Duox
Ps-rboh-B
Ps-rboh-C
Ps-rboh-A
rboh (oomycetes)
moss-Pp-rboh-A
moss-Pp-rboh-C
moss-Pp-rboh-B
moss-Pp-rboh-D
rboh (moss)
plant-Os-rboh-E
plant-Os-rboh-F
plant-Os-rboh-D
plant-Os-rboh-H
plant-Os-rboh-A
plant-Os-rboh-B
plant-At-rboh-D
plant-Os-rboh-C
plant-Os-rboh-G
rboh (higher plants)
slime mold amoeba-Dd-NoxC
NoxC (amoeba)
snail-Lg-Nox5A
snail-Lg-Nox5B
human-Hs-Nox5
waterflea-Dp-Nox5A
waterflea-Dp-Nox5B
Nox5
amoeboflagellate-Ng-NoxA
NoxA (amoeboflagellate)
slime mold amoeba-NoxA
slime mold amoeba-NoxB
NoxA/B (amoeba)
snail-Lg-Nox4
human-Hs-Nox4
sea aemone-Nv-Nox4
Nox4
fungus-Pg-NoxA
fungus-Bd-NoxA
fungus-Mg-NoxA
fungus-Fg-NoxA
fungus-Pp-NoxA
fungus-Cc-NoxA
fungus-Lb-NoxA
fungus-Bd-NoxB
fungus-Mg-NoxB
fungus-Fg-NoxB
fungus-Pg-NoxB
fungus-Pp-NoxB
fungus-Cc-NoxB
fungus-Lb-NoxB
NoxA/B (fungi)
choanoflagellate-Mb-Nox2
sea urchin-Sp-Nox2A
sea urchin-Sp-Nox2B
ascidion-Ci-Nox2
sea anemone-Nv-Nox2A
sea anemone-Nv-Nox2B
snail-Lg-Nox2
ancestral Nox2
(co-ortholog Nox1-3)
human-Hs-Nox1
human-Hs-Nox2
human-Hs-Nox3
Nox1-3 (vertebrates)
0.1

Alignment of Nox domain

```
Dp-Nox5A        ------------SRWSASVFWSYLRNNAAFLAFLVAFLLVNIALFASRAYQYRQET---- 44
Dp-Nox5B        ------------SQSVFQSFSSYAKNNVPFIIFVLAYAIVNLGLIVSRGIQYKEEN---- 44
Hs-Nox5         -----------------------WHNHRSQLFCLATYAGLHVLLFGLAASAHRDLG---- 33
Lg-Nox5A        -------------RACRYFTLKYWRNNRKEGVLFLLYFFINICLFALNCFLYRKSN---- 43
Lg-Nox5B        -------------ELPRYLQSNYIRNNLKKIIFFIIYFMTNIGLYAYGAYSYRESN---- 43
Dd-NoxC         ----------------FHSLKRYLKIEGSKLFFISLFFIINSILVITSFLNVHANNKRAI 44
Mg-NoxA         ---------------MSVGEFLAKQLTAQKLFFNISFWGFHIGIFAYGWYKQFSDPRLAG 45
Fg-NoxA         -----------MGSQLGFVELIKKQFVPGKLLYHFLFWTFHWGIFAYGWWKQAVDPRLAG 49
Cc-NoxA         ---------------MAETWFQREFLQARRLTFNVLFYGLHLAIFAYGWYSQATNQRLAG 45
Lb-NoxA         ---------------MGESWFRREFMVPRRLAFNVLFYGAHLSLFIYGWYSQATNQRLAG 45
fPp-NoxA        ---------------MGESWFRREFLAPRRLIFNVLFFGLQVSFFSYGWWAQATNNKLAA 45
Pg-NoxA         ------------MTDCEKCYTCAIQDNKLKELHRGLEGAPESEVEQCHWPNKASGLVSHR 48
Bd-NoxA         ----------------------MVEITARLVAFHVLFWGFHIALFVYGFFKQKNDPELQV 38
Mg-NoxB         ----------------------WMVNEGYRRFFVFVFMILHAMVFAFGFVNYAVKDNLQR 38
Fg-NoxB         ----------------------WMINEGYRRFFVFVFMILHALIFSFACVHYAQKESLET 38
Cc-NoxB         ----------------KQRWDVWMINEGGRQLFFGTWIFLHLLVAVFGFMHYQLKDNSEG 44
Lb-NoxB         ------MKKPVEPKTLREKFSIWMINEGGRQLFFGTWIFLHLLVAVFGFMNYQLKDNLVD 54
fPp-NoxB        ----------------IDRWRLWMINEGGKRLFFTFFILLHVLVIVFGFLYYDTSDDLKG 44
Pg-NoxB         ----------------TTRFKSWMVNEGSRKFFVWTWIFIHIITFVFAFLNFLLKDNLTQ 44
Bd-NoxB         ----------------MVTFNNWMVNEGRSRLFFALFIASQLGYFAWSYYQLWTSPTLVT 44
Hs-Nox2         -------------------MGNWAVNEGLSIFVILVWLGLNVFLFVWYYRVYDIPPKFFY 41
Hs-Nox3         ------------------MMGCWILNEGLSTILVLSWLGINFYLFIDTFYWYEEEESFHY 42
Hs-Nox1         -------------------MGNWVVNHWFSVLFLVVWLGLNVFLFVDAFLKYEKADKYYY 41
Lg-Nox2         -------------------MGEWIVNELPKWIVVTVWLLINTGIFIGTFFSYKNDIEYFY 41
Nv-Nox2A        --------------------MANLGNDVARYIVLIIWMGINIYLFIDVYYKLATTKENMY 40
Nv-Nox2B        ---------------------------MNFYFSQIIWAVINIILFATTYHNYGVTKKYTY 33
Ci-Nox2         ---------------MNARLNGFLVNEFPKYIVFLLWLGLNGFLFGYYYNFYNTKKTFFY 45
Sp-Nox2A        -------------------MGDKFLNEGLKYFFLLLWLAANVAYWVVTFLVYEQGPQYFY 41
Sp-Nox2B        ----------------------------------AAWAIVNLIIWLVTFFKYMDNANYIY 26
Mb-Nox2         ---------MQFWWQQGLTFRQWFVNDGPMYTFVLLWMCANVGLFFYTFSFYTQD-KYNY 50
Hs-Nox4         ---------------MAVSWRSWLANEGVKHLCLFIWLSMNVLLFWKTFLLYNQGPEYHY 45
Nv-Nox4         -----------------------------YFFFQVLWTGPFIVIFWQTYNKYSNCPEYFY 31
Lg-Nox4         -------------MAYQVSVASWIRRHGQKYFLLIVWICGCLFLFYRTFYYFKDDPSFFY 47
Dd-NoxA         -----------MRLPTKEEIQRYWVNEGNKLILVILYTLGNIAAFVYTFVHYYNSPAFEV 49
Dd-NoxB         -----------------------WHR-GISTYIMLFYIALNIGVGVHMFYNMYHSDIFKF 36
Ng-NoxA         -------------FTNRHEFMNYWSAEGGQKLVVVLIWVILNLFLFTEAFVRYYYALLLVN 47
Os-rboh-C       ------------VRHYWQQFMYFLEENWKRSWVMTLWISICIALFIWKFIQYRNRAVFGI 48
Os-rboh-G       ------------LRRGLRRLSYFMEDNWKRVWVMALWLAINAGLFTWKFMAYKRHPTFDV 48
At-rboh-D       ---------------WSEKIKYFILDNWQRLWIMMLWLGICGGLFTYKFIQYKNKAAYGV 45
Os-rboh-A       ------------IRKISTSLSYYFEDNWKRLWVLALWIGIMAGLFTWKFMQYRNRYVFDV 48
Os-rboh-B       ------------IRKISTKLSYYLEDNWKRLWVLALWIGIMAGLFIWKFIQYRHRYVFNV 48
Os-rboh-D       ------------VRKAAARVRVAAEENWRRAWVVALWFAAMASLFVWKFVQYRRTPAFRV 48
Os-rboh-H       ------------PRRAAARAQVAAEENWRRAWVLALWFAAMAGLFAWKFVQYRRTPAFRV 48
Os-rboh-E       ------------LKRHVSRTVDFVHENWKRIWLVALWLAVNVGLFAYKFEQYERRAAFQV 48
Os-rboh-F       ------------MQRRFNKTVDFIHENWKRIWVLSLWAILNIALFMYKFVQYSRRDAFQV 48
mPp-rboh-A      ------------IIKLAEKCVHFFLDNWKRIWILALWMSVMAALFAWKFLQYRDRSSWWI 48
mPp-rboh-C      ------------MMGVAEKSAYFLLDNCKRIWILALWISVMVGLFAWKFLQYRNRSAWWV 48
mPp-rboh-B      -----------RIRSFAEKTRNCFQDNWKRLWILALWMMAMTGLFIWKFFQYREHAAFPI 49
mPp-rboh-D      ------------VQDATGKAKHFLHDYWKRLWILALWVMAMTGLFTWKFLQYRHHEAYAV 48
Ps-rboh-B       ------------PIRPKSKFGAWYSANKLRIWWLLFYAILNNIAFWVKWFSYDVDP---A 45
Ps-rboh-C       -------------------------DNKLRIWWLLFYFIVNNIAFWVKWFMYEVDP---A 32
Ps-rboh-A       ---------------RWYRINKFCRRYKAEIFWLTLYFLLMVAVFIAKASRFAFDP---A 42
Hs-Duox1        ----------------------FIENYRRHIGCVAVFYAIAGGLFLERAYYYAFAAHHTG 38
Hs-Duox2        ----------------------FVENYRRHIVCVAIFSAICVGVFADRAYYYGFASPPSD 38
Lg-Duox1        ------------FQTKIYEITRYIENYRLHIFWFILFNLVTLGIFIERAYYYSIEREHAG 48
Lg-Duox2        -------------QEKIYAITKYIEIYRLHIFWVLLYTLVTCGIFIERAYYYAEGREHAG 47
Lg-Duox3        -----------PFKAKVKVIKHFIENYRQHIFFLVLVFGIVLGLFAERFYYYTVEREHSG 49
Dp-Duox         ------------ILRKWDELTTFLEANRQNIFYLFVFYVVTIALFVDRFIHYSFMSEHTD 48
Cb-Duox         ------------PMTLVHQVCAFLETYRQHVFIVFCFVAINLVLFFERFWHYRYMTENRD 48
Mg-NoxC         ----------------FRRVRAYWAVHGPEIAFLGIVVGLQLGLGIWQCHKYASGEQYQA 44
Fg-NoxC         ----------------WRRIRSYWAVHGPEIVFLGVVISMQLAFGIWQLVKYQTTPGYRA 44
```

```
Cm-NoxD2        ----------------LDLWNERVGESGLFFSLLVAYWCSQAWFFVYGVKYALSLALPSD 44
Cm-NoxD1        -----------------QIWNIHLVENGLWYSIVLLFFCAMSWFFAYGVRYVIVDLRPSG 43
Pt-NoxD         ----------------WAKSEAFFFNQGANLAFLGLMLALNILMLVWGTYEFTGTRFVTD 44
An-Fre1         MLLRISFLAIFVPAMAKQKSFDDAVSSLDADCLCPIYTALSQYTFSGTPAGGSYNHSISV 60
Sc-Fre1         ---------MVRTRVLFCLFISFFATVQSSATLISTSCISQAALYQFGCSSKSKSCYCKN 51


Dp-Nox5A        --------PAYVIAR-ACGQVLNFNCAFVVVL-MLHHCITWIRQTKLAV----------- 83
Dp-Nox5B        --------VFYILAR-CGGQALNFNCAFILVL-MLRHCITLLRQIGCGY----------- 83
Hs-Nox5         --------ASVMVAK-GCGQCLNFDCSFIAVL-MLRRCLTWLRATWLAQ----------- 72
Lg-Nox5A        --------VYIIIAR-GCGMCLNFNCAFILMT-MLRKTITYIRTFKNLH----------- 82
Lg-Nox5B        --------NAIIIAR-GSGMCLNFNCMFVLVL-MLRKCITWLRTWQYVYC---------- 83
Dd-NoxC         EL----FGPGVYITR-IAAQLIEFNAAIILMT-MCKQLFTMIRNTKFKF----------- 87
Mg-NoxA         LN--TLTFSVWISRG--AGLVLSVDGMLILLP-VCRTIMRWIRP---------------- 84
Fg-NoxA         LN--TLKFSVWISRG--AGLVLSVDCMLILLP-VCRTVMRWVRP---------------- 88
Cc-NoxA         LN--TLQWSVWVSRG--AGLVLALDGGLILIP-MLRNIIRVVRP---------------- 84
Lb-NoxA         LN--SLKYSVWTSRG--AGLVLAFDGGLILLP-MLRNIIRIIRP---------------- 84
fPp-NoxA        LN--ALQWSVWVSRG--AGLVLAFLAACLLLP-MLRNCIRVIRP---------------- 84
Pg-NoxA         SN--GIGVPGSHEDGSLKGFHVCMGRRLMPLSRWVRTFYTSLQK---------------- 90
Bd-NoxA         LN--SIGPSVTISRG--AGLVLGVDCAALLIP-VCRNIVRFFRSS--------------- 78
Mg-NoxB         AR--DTFGPTFMIAR-AAALVLHFDVALILFP-VCRTFISLARQTPLN------------ 82
Fg-NoxB         SR--QTFGFTFIIAR-SAALVLHVDVAIILFP-VCRTLISLLRQTPLN------------ 82
Cc-NoxB         AR--SVFGITFPIAR-TAALVLHVDVIFILLP-VCRNFISLLRRTPLN------------ 88
Lb-NoxB         AR--ALFGVTFTIAR-TAALILHIDVIFILLP-VCRNFVSLLRRTPLN------------ 98
fPp-NoxB        AR--KTFGITYPIAR-SAALVLHVEVAFILLP-ICRNFISLLRRTPLN------------ 88
Pg-NoxB         AR--ATFNITYPIAR-ASALVLHVDVAFILLP-ICRNFITLLRRSALN------------ 88
Bd-NoxB         FR--TVLQHGLPMAR-AAANVINLDCGIILF----------------------------- 72
Hs-Nox2         TR--KLLGSALALAR-APAACLNFNCMLILLP-VCRNLLSFLRGSSAC------------ 85
Hs-Nox3         TR--VILGSTLAWAR-ASALCLNFNCMLILIP-VSRNLISFIRGTSIC------------ 86
Hs-Nox1         TR--KILGSTLACAR-ASALCLNFNSTLILLP-VCRNLLSFLRGTCSF------------ 85
Lg-Nox2         LR--LIVGDALCFAR-ASAACLNFNCFLILLP-VCRNFISFLRGTCAR------------ 85
Nv-Nox2A        LN--KLVKFGLALAR-APAMVLNFNCMLILLT-MCRNLLSFIRGSCK------------- 83
Nv-Nox2B        LR--EVIKNGLPIAR-GAAMVLNFNCMLILLS-MCRNLNSLIRRHCKST----------- 78
Ci-Nox2         TR--VLLGPALALAR-APAACLNLNCLLVLLP-VCRNLLSLFRKACMC------------ 89
Sp-Nox2A        IR--RITGVGLSIAK-AAGAALNLNSMIILLP-ICRNLISFFRGSCATNTL--------- 88
Sp-Nox2B        TK--YLMKNGLPVAR-ASAACLNFNSMLILFP-VCRNMISYLRGSCESTKF--------- 73
Mb-Nox2         LR--RIVHFGLPVAR-GAASVLNLNCALVLLP-VCRNLVNFCRGIFES------------ 94
Hs-Nox4         LH--QMLGLGLCLSR-ASASVLNLNCSLILLP-MCRTLLAYLRGS-QKVPS--------- 91
Nv-Nox4         LH--NMLGNSLSWSR-GAAAVLWFSCSLMLLP-MCRNLLAFVRNT-LCKSS--------- 77
Lg-Nox4         VR--QILGVSMCISR-GTASILNLNCGLILLP-TCRCILTFITYLNSKVRI--------- 94
Dd-NoxA         VG------YGVCFAR-GCAQLLKLNCALILVP-VLRNLLSFLRGTFLN------------ 89
Dd-NoxB         LG------LSFCFSR-TAARLINLNSAVILLP-VLRNFLSWLRGTIVN------------ 76
Ng-NoxA         ITGVTVVPFYVALAR-GFGQLLNFNCALLILP-TMRTLLNVFRSLKFG------------ 93
Os-rboh-C       MG------YCVTTAK-GAAETLKFNMALVLLP-VCRNTITWIRSKTQVGA---------- 90
Os-rboh-G       MG------YCVCVAK-GGAETTKFNMALILLP-VCRNTITWLRSRTKLGA---------- 90
At-rboh-D       MG------YCVCVAK-GGAETLKFNMALILLP-VCRNTITWLRNKTKLGT---------- 87
Os-rboh-A       MG------YCVTTAK-GAAETLKLNMAIILLP-VCRNTITWLRS-TRAAR---------- 89
Os-rboh-B       MG------YCVTTAK-GAAETLKLNMAIILLP-VCRNTITWLRN-TRAAR---------- 89
Os-rboh-D       MG------YCLPTAK-GAAETLKLNMALVLLP-VCRNTLTWLRS-SWARF---------- 89
Os-rboh-H       MG------YCLPTAK-GAAETLKLNMALVLLP-VCRNTLTWLRS-SWARF---------- 89
Os-rboh-E       MG------HCVCVAK-GAAEVLKLNMALILLP-VCRNTLTTLRS-TALSH---------- 89
Os-rboh-F       MG------YCVCIAK-GAAETLKLNMAVILLP-VCRNTLTRLRS-TALSK---------- 89
mPp-rboh-A      MG------DCLCVAK-GAAETLKLNMALILLP-VCRNTLTRLRS-TRLWK---------- 89
mPp-rboh-C      MG------DCLCVAK-GAAETLKLNMALILLP-VCRNTITRLRS-TRLRR---------- 89
mPp-rboh-B      MG------NCLCVAK-GAAETLKLNMALVLLP-VCRNTLTRLRS-TRLGK---------- 90
mPp-rboh-D      MG------ECLCVAK-GAAETLKLNMALILLP-VCRNMLTRLRS-TRLGK---------- 89
Ps-rboh-B       IG------WGLRIAR-ANAQVAMVNCVFVLLP-MCRSITQVMKRSRILWR---------- 87
Ps-rboh-C       IG------WGLRIAR-ANAQVAMLNCVFVLLP-MCRSITQVMKRSKFLWR---------- 74
Ps-rboh-A       VG------NCPRVAK-GFAEICLVNTMFVLLP-MCRNFVTGLRTLPVVVN---------- 84
Hs-Duox1        ITD-TTR-VGIILSR-GTAASISFMFSYILLT-MCRNLITFLRETFLNR----------- 83
Hs-Duox2        IAQ-TTL-VGIILSR-GTAASVSFMFSYILLT-MCRNLITFLRETFLNR----------- 83
Lg-Duox1        LRR-LAG-YGVSVTR-GAASVQMFLYVSLLVT-MSRNTLTFFRETFLHR----------- 93
Lg-Duox2        LRR-LSGAWTTAMIR-GSASVIMFTYVSLLVT-MCRNTITLLRETVLHR----------- 93
Lg-Duox3        LMR-LMS-YGISITR-GAAAAMSFTFSLLLLT-MCRNTITYLRSTFLNM----------- 94
```

```
Dp-Duox         LRH-IMG-VGIAITR-GSAAALSFCYSLLLLT-MSRNLLTKLKEFSVQQ----------- 93
Cb-Duox         LRR-VMG-AGIAITR-GAAGALSFCMALILLT-VCRNIITLLRETVISQ----------- 93
Mg-NoxC         AFG-----WGVALAK-LCAGALYPTFFFLILS-MSRYFSTFLRRSYHLSR---------- 87
Fg-NoxC         AFG-----WGVVMAK-TCAGALYPTFFFLILS-MSRYFSTWLRRSYHISR---------- 87
Cm-NoxD2        WSR-----YMLIFGR-GFGYVATFNALLLPIT-VNRTLTNILYRLPLYD----------- 86
Cm-NoxD1        ILR-----YAGSIAR-GMGFACTFCSLVLPLT-INRTLATVLYRTPLYD----------- 85
Pt-NoxD         SD---ILRVTLPIAR-AGGRVVTWNAALIFLS-GSKYLWTWLRQSPLHLG---------- 89
An-Fre1         TAFFAEESYIQTLTNFDSICNSTVEIASMYASARARCDGNSPAASVSFWQE--------- 111
Sc-Fre1         IN---WLGSVTACAYENSKSNKTLDSALMKLASQCSSIKVYTLEDMKNIYLNASNYLRAP 108


Dp-Nox5A        ---------FLPLDQH-VYLHKLCG----------------------------------- 98
Dp-Nox5B        ---------FLPLDLH-VYLHKVCG----------------------------------- 98
Hs-Nox5         ---------VLPLDQN-IQFHQLMG----------------------------------- 87
Lg-Nox5A        ---------FLPLDQH-IEMHKLTG----------------------------------- 97
Lg-Nox5B        ---------ILPLDQH-ILFHKTTG----------------------------------- 98
Dd-NoxC         ---------LFPVDKY-MTFHKLIG----------------------------------- 102
Mg-NoxA         -----KIRFI-PLDEN-IWFHRQIA----------------------------------- 102
Fg-NoxA         -----KIRFL-PLDEN-LWMHRQLA----------------------------------- 106
Cc-NoxA         -----KLTWLFPADEN-IWFHRQVA----------------------------------- 103
Lb-NoxA         -----KLTWAFPADEN-IWFHRQVA----------------------------------- 103
fPp-NoxA        -----KVAFLFPADEN-IWFHRQAA----------------------------------- 103
Pg-NoxA         -----PYAPFKTEDRM-ARLSRQVA----------------------------------- 109
Bd-NoxA         -----FLNKYIPIDSN-LYFHKWLA----------------------------------- 97
Mg-NoxB         --------GIIQFDKN-ITFHITTA----------------------------------- 98
Fg-NoxB         --------GILQFDKN-ITFHIVTA----------------------------------- 98
Cc-NoxB         --------DIIPFDKN-ITFHKATA----------------------------------- 104
Lb-NoxB         --------DIIPFDKN-ITLHKATA----------------------------------- 114
fPp-NoxB        --------SYIPFDKN-ITFHKAVA----------------------------------- 104
Pg-NoxB         --------QVIPFEKN-ITFHKFTG----------------------------------- 104
Bd-NoxB         -----------TFDKN-ITFHIWIA----------------------------------- 85
Hs-Nox2         ----CSTRVRRQLDRN-LTFHKMVA----------------------------------- 105
Hs-Nox3         ----CRGPWRRQLDKN-LRFHKLVA----------------------------------- 106
Hs-Nox1         ----CSRTLRKQLDHN-LTFHKLVA----------------------------------- 105
Lg-Nox2         ----CHR-ARRQLHRQ-ITYHKYIA----------------------------------- 104
Nv-Nox2A        ----CCSFMLRLLDKHHVTFHKYIA----------------------------------- 104
Nv-Nox2B        ----CMAPVIRVLDKS-ITFHKYIA----------------------------------- 98
Ci-Nox2         ----CPRRIRRVLDKN-IKFHRMCA----------------------------------- 109
Sp-Nox2A        ----CRRSVRRQLDKN-LTFHKTVA----------------------------------- 108
Sp-Nox2B        ----SRRNLRRQLDKN-ITFHKLIA----------------------------------- 93
Mb-Nox2         -----KRSIRRLFDKN-ILFHKWCA----------------------------------- 113
Hs-Nox4         ------RRTRRLLDKS-RTFHITCG----------------------------------- 109
Nv-Nox4         ------RRLRRLLDKH-IWFHKACA----------------------------------- 95
Lg-Nox4         ------STIRVILESC-KGFHKVCA----------------------------------- 112
Dd-NoxA         --------NYVPFDKN-IVFHKLIA----------------------------------- 105
Dd-NoxB         --------NYIPIDKH-LNFHKLCA----------------------------------- 92
Ng-NoxA         --------SIFPLDKN-LVFHRYLA----------------------------------- 109
Os-rboh-C       ---------VVPFNDN-INFHKV------------------------------------- 103
Os-rboh-G       ---------VIPFNDN-INFHKV------------------------------------- 103
At-rboh-D       ---------VVPFDDS-LNFHKV------------------------------------- 100
Os-rboh-A       ---------ALPFDDN-INFHKT------------------------------------- 102
Os-rboh-B       ---------ALPFDDN-INFHKT------------------------------------- 102
Os-rboh-D       ---------FVPFDDN-ITFHKM------------------------------------- 102
Os-rboh-H       ---------FVPFDDS-ITFHKVESSSGFKWAPPGRSNQCSVAGEPAGTEDPRPGRLRVK 139
Os-rboh-E       ---------VIPFDDN-INFHKV------------------------------------- 102
Os-rboh-F       ---------VVPFDDN-INFHKV------------------------------------- 102
mPp-rboh-A      ---------IIPFDDN-LDFHKAS------------------------------------ 103
mPp-rboh-C      ---------IIPFDDN-LDFHK-------------------------------------- 101
mPp-rboh-B      ---------IIPFDDN-LDFHKET------------------------------------ 104
mPp-rboh-D      ---------LIPFDEN-LDFHKASL----------------------------------- 104
Ps-rboh-B       ---------YIPFDDS-IAFHKIAG----------------------------------- 102
Ps-rboh-C       ---------IIPFDDH-IAFHKISG----------------------------------- 89
Ps-rboh-A       ---------HLPIDHH-IEFHKICG----------------------------------- 99
Hs-Duox1        ---------YVPFDAA-VDFHRLIA----------------------------------- 98
```

```
Hs-Duox2        ---------YVPFDAA-VDFHRWIA----------------------------------- 98
Lg-Duox1        ---------FIPFDAF-HAMHKYTA----------------------------------- 108
Lg-Duox2        ---------FIPFDSA-VAFHKYIA----------------------------------- 108
Lg-Duox3        ---------FIPFDSH-ISFHKVIA----------------------------------- 109
Dp-Duox         ---------YIPLDSH-IQFHKICA----------------------------------- 108
Cb-Duox         ---------YIPFDSA-IAFHKIVA----------------------------------- 108
Mg-NoxC         ---------FINWDLS-QEFHIKIS----------------------------------- 102
Fg-NoxC         ---------FFNWDLS-QEFHIRIS----------------------------------- 102
Cm-NoxD2        ---------IFSVARWLPDLHAIIA----------------------------------- 102
Cm-NoxD1        ---------LLCVERWIPDMHTIVG----------------------------------- 101
Pt-NoxD         ----------FPIDNVMPYYHKMIA----------------------------------- 104
An-Fre1         ----VCDVDISSLETNRSTTYTSMSIVDPDVNTTASTG----TIKNPVLLSESYYRRAYK 163
Sc-Fre1         EKSDKKTVVSQPLMANETAYHYYYEENYG------------------------------- 137

Dp-Nox5A        -----------------------FTIIFFGTLHTAMHMINF----SNELVSPVT------ 125
Dp-Nox5B        -----------------------GVVVVLSAVHTLMHLINFPLNIADKVESPVT------ 129
Hs-Nox5         -----------------------YVVVGLSLVHTVAHTVNF------VLQAQAE------ 112
Lg-Nox5A        -----------------------IMIAVFTVIHTLAHIGNA--------VVVAE------ 120
Lg-Nox5B        -----------------------IMIAVHSLIHTMGHIGNA--------VIVSE------ 121
Dd-NoxC         -----------------------YTLIIASFLHTIGWIVGMA-----VATGKPD------ 128
Mg-NoxA         -----------------------YAMLIFSITHTAAHYVNFFNVERLQIRA--------- 130
Fg-NoxA         -----------------------YSMLLFTCLHTGAHYVNFYNVEITQIRP--------- 134
Cc-NoxA         -----------------------YSMAFWSVVHTTAHYVNFINVERTQIRK--------- 131
Lb-NoxA         -----------------------YSLAFWAMVHTTAHYVNFINVERTQIRK--------- 131
fPp-NoxA        -----------------------YCLAFWSMVHTTAHYVNFINVERTQVRQ--------- 131
Pg-NoxA         -----------------------YTTLFFTAIHTTAHYVNMFHVETTQIRP--------- 137
Bd-NoxA         -----------------------YSMLFFSLLHTNAHYTNFFFVETKLPSLG-------- 126
Mg-NoxB         -----------------------WSIVFFSWVHTVAHWNNFAQIAAQ------------- 122
Fg-NoxB         -----------------------WSIVFWSWVHTIAHWNNFAQVAIK------------- 122
Cc-NoxB         -----------------------WSIVIGSAVHTLAHMVNFTKLALS-IPN--------- 131
Lb-NoxB         -----------------------WSIVAGTVVHVVAHMVNFYKLAMADTDA--------- 142
fPp-NoxB        -----------------------WGICLFTFIHIAAHMVNFARLAFADPLA--------- 132
Pg-NoxB         -----------------------FALAFFSAIHILAHMVNFGQLAVR------------- 128
Bd-NoxB         -----------------------YSIAFWTFVHVIAHYFNYNNVRIA------------- 109
Hs-Nox2         -----------------------WMIALHSAIHTIAHLFNVEWCVNARVN---------- 132
Hs-Nox3         -----------------------YGIAVNATIHIVAHFFNLERYHWSQSE---------- 133
Hs-Nox1         -----------------------YMICLHTAIHIIAHLFNFDCYSRSRQA---------- 132
Lg-Nox2         -----------------------YMICLQTAIHIAAHCFDFEFLIEAYDS---------- 131
Nv-Nox2A        -----------------------YMICLQTAIHCIAHIFNVEFLILAWQ----------- 130
Nv-Nox2B        -----------------------YTICFFTIVHVGAHCYNFENLIDSWS----------- 124
Ci-Nox2         -----------------------YMIVLMTLIHYFAHCFNVDFFTSAYQSKILATDTPAI 146
Sp-Nox2A        -----------------------YMIVVWTIVHVVAHAFNFRNLYNHYRCVTT------- 138
Sp-Nox2B        -----------------------YAIGFFVILHVGAHCFNLQNLYNG-RKATS------- 122
Mb-Nox2         -----------------------YVICVFASIHICAHFFNVNNLVEDGTYG--------- 141
Hs-Nox4         -----------------------VTICIFSGVHVAAHLVNALNFSVNYSED--------- 137
Nv-Nox4         -----------------------ITTIIAAVVHTVAHLINGKRFSENYSTD--------- 123
Lg-Nox4         -----------------------ITIILSSVIHVIGHIFNALKYSLYYNYQ--------- 140
Dd-NoxA         -----------------------WVICFATFGHVMAHFNNFR-LYQDITPQ--------- 132
Dd-NoxB         -----------------------FMLFCCTIIHCVGHYISFKKINDDVLKI--------- 120
Ng-NoxA         -----------------------YWIVVCVIGHTLGHYLNYACCWQLYN----------- 135
Os-rboh-C       ---------------------IAAGVAVGVALHAGAHLTCDFPRLLHAS----------- 131
Os-rboh-G       ---------------------VAGGVVVGVALHGVTHLTCDFPRLLHAS----------- 131
At-rboh-D       ---------------------IASGIVVGVLLHAGAHLTCDFPRLIAAD----------- 128
Os-rboh-A       ---------------------IAAAIVVGIILHAGNHLVCDFPRLIKSS----------- 130
Os-rboh-B       ---------------------IAAAIVVGVILHGGLHLVCDFPRLIGSS----------- 130
Os-rboh-D       ---------------------IATAIVVGITLHAGNHLACDFPRLIASG----------- 130
Os-rboh-H       APVIQKSPRPTGLPLSCGATIIATAIALGICTHAGTHLACDFPRLIGSS----------- 188
Os-rboh-E       ---------------------IAATIAAATAVHTLAHVTCDFPRLINCP----------- 130
Os-rboh-F       ---------------------IALTIAIGAATHTLAHVTCDFPRLVSCP----------- 130
mPp-rboh-A      -------------------QIVAGGMAAGVFLHVGCHATCDLPRFVNAD----------- 133
mPp-rboh-C      --------------------IVAGGIAAGVILHATCHITCDIPRFVEAD----------- 130
mPp-rboh-B      -----------------------GGIAAGVFIHGVCHITCDIPKFVESG----------- 130
mPp-rboh-D      --------------LPDPEHCIAAAIAVGVFVHGTLHITCDIPKFVNCD----------- 139
```

```
Ps-rboh-B       -----------------------SVLLTAGLIHTIAHVFNEIYLYLVATPEEIK------ 133
Ps-rboh-C       -----------------------SVLLIAGLIHTIAHVFNEIYLYLIATPDEVK------ 120
Ps-rboh-A       -----------------------VVLLLAFLGHTAAWLV--IVVYVRTVPLAVW------ 128
Hs-Duox1        -----------------------STAIPVAVLHSVGHVVNVYLFSISPLSV--------- 126
Hs-Duox2        -----------------------MAAVVLAILHSAGHAVNVYIFSVSPLSL--------- 126
Lg-Duox1        -----------------------LLALIFTAMHIIGHGINLFHISTQASSD--------- 136
Lg-Duox2        -----------------------VLAMIGTIVHIFGHAVNLYCVVTQPPQD--------- 136
Lg-Duox3        -----------------------WTALFFSGLHVIGYSFNFYHLATQPTKF--------- 137
Dp-Duox         -----------------------LTAFFFSMLHTVGHVVNFYHVSTQPIEH--------- 136
Cb-Duox         -----------------------LFAAFWATLHTVGHCVNFYHVGTQSQEG--------- 136
Mg-NoxC         -----------------------IVALVLASLHALGHLSGTFNWGSRPERQ--------- 130
Fg-NoxC         -----------------------CVAILLATLHAIGHLTGSFVHGSDPANE--------- 130
Cm-NoxD2        -----------------------RWFTIAGWIHGILLSVAYGVGTLPFRGG--------- 130
Cm-NoxD1        -----------------------TSLAATGWIHGIAQIVNYAASVFVFRGG--------- 129
Pt-NoxD         ----------------------WTIIFMGCVVHTIPQVINYATKELRIEGG--------- 133
An-Fre1         TCVIHDKALGQDIRFGWGLMGYWVAILVFGMAQKILSLWSARRALDSGRDAEAN------ 217
Sc-Fre1         ----------IHLNLMRSQWCAWGLVFFWVAVLTAATILNILKRVFGKNIMANS------ 181


Dp-Nox5A        ------------------------------NQTHSTEEWLFTTIPGLFGLIPGVAN-LTG 154
Dp-Nox5B        ------------------------------KETHSAIEWLFTTEPGLFGLVPGLAN-LTG 158
Hs-Nox5         ------------------------------ASPFQFWELLLTTRPG-IGWVHGSAS-PTG 140
Lg-Nox5A        ------------------------------DFGVTVWEFIFTTKAN-IGWVLGFAP-LSG 148
Lg-Nox5B        ------------------------------TNNITAVEVIFTTKAN-LGWLLGSAP-ITG 149
Dd-NoxC         ------------------------------NIFYDCLAPHFKFRPTVWEMIFNSLPGVTG 158
Mg-NoxA         ---------------------------------QTAVQIH-----------YAQPGGATG 146
Fg-NoxA         ---------------------------------VTALQIH-----------YAQPGGITG 150
Cc-NoxA         ---------------------------------QTAQMIH-----------YTQPGGITG 147
Lb-NoxA         ---------------------------------QTALQIH-----------YTQPGGITG 147
fPp-NoxA        ---------------------------------EYALQIH-----------YTQAGGITG 147
Pg-NoxA         ---------------------------------EKAIEIM-----------YSETGPLTG 153
Bd-NoxA         ---------------------------------LKAWMIH-----------YLAWSGATG 142
Mg-NoxB         --------------------------QKLG--IYGWLLAN-----------FVSGPGWTG 143
Fg-NoxB         --------------------------YNLG--IYGWLLAN-----------FVSGPGWTG 143
Cc-NoxB         --------------------------MTTGQRIGAFMAAN-----------FATGPGLTG 154
Lb-NoxB         --------------------------KTTGQRVLAFLEAN-----------FNTGPGVTG 165
fPp-NoxB        --------------------------KTPGERFVVWLIAN-----------FTTGPGVTG 155
Pg-NoxB         --------------------------TQTG--IVGFIGAN-----------FLTGPGATG 149
Bd-NoxB         ----------------------------LG---VSAEYLS-----------LVSGPGLTG 127
Hs-Nox2         -NSDPYSVALSELGDRQNE----SYLNFARKRIKNPEGGLYLA---------VTLLAGITG 179
Hs-Nox3         -EAQGLLAALSKLGNTPNE----SYLNPVRTFPTNTTTEL-----------LRTIAGVTG 177
Hs-Nox1         -TDGSLASILSSLSHDEKKGG--SWLNPIQSRNTTVEYVT-----------FTSIAGLTG 178
Lg-Nox2         -PTKS-IQAITNLDTSNNG----TWLNPVRVQGTDATREV-----------FKTIAGVSG 174
Nv-Nox2A        -KGG-IYTKLCQLEDVGNE----TYINPIRDPNADPITVL-----------WTIVSGVTG 173
Nv-Nox2B        -KENEIDAKLSQLD--GAD----NWVNPIR--SEMSVGKR-----------GSKFAGVTG 164
Ci-Nox2         IQQKKLIAKLIQIGNNGNE----TYLNPIR-KSVFSVGAV-----------FLLTGGWTG 190
Sp-Nox2A        -DNDELCEGISAIGRKFKAKPEDNWLNPIQGAKTLPAGLGLIEQA------LIPIAGWSG 191
Sp-Nox2B        -EDDWLANRLSQP--SFDLNP----FKTIRSSD--VSGLGVIGPG------LSLLAGWTG 167
Mb-Nox2         --------------------------RILRDGTPMSQEEVL----------FTTVAGGTG 165
Hs-Nox4         ---------------------------FVELNAARYRDEDPRKLL------FTTVPGLTG 164
Nv-Nox4         ---------------------------HPPLNFAKNRDQDPLEFV------MLSVAGFTG 150
Lg-Nox4         ---------------------------YPEVNGASYPNQNPIFII------LWTVSGVTG 167
Dd-NoxA         --------------------------EYKRILGIDYPNLTPIKYA------FATLAGWTG 160
Dd-NoxB         --------------------------DDGKSVAGDYLNININNFPDEKYLFFKSVPGITG 154
Ng-NoxA         --------------------------SARTPLEACWWNK----------------YGTTG 153
Os-rboh-C       ----------------------------DAQYELMKP-FFGEKRPPNYWWFVKGTEGWTG 162
Os-rboh-G       ----------------------------DAAYEPMKK-YFGQTRIPDYWWFVRGVEGITG 162
At-rboh-D       ----------------------------EDTYEPMEK-YFGD-QPTSYWWFVKGVEGWTG 158
Os-rboh-A       ----------------------------DEKYAPLGQ-YFGEIKPT-YFTLVKGVEGITG 160
Os-rboh-B       ----------------------------EEKYAPLGK-YFGETKPT-YLTLVKGVEGITG 160
Os-rboh-D       ----------------------------PEEYRLVAD-AFGPEKPT-YVGLLSGVEGITG 160
Os-rboh-H       ----------------------------REEYELLLSGFFGASRPT-YRGLLAGVEGVTG 219
Os-rboh-E       ----------------------------SDKFMATLGPNFGYRQPT-YADLLESAPGVTG 161
Os-rboh-F       ----------------------------RDKFEATLGPYFNYVQPT-YSSLVASTPGWTG 161
```

```
mPp-rboh-A      ----------------------------KETFFKHLGDHFDT-QPT-YSDILNMSVGYSG 163
mPp-rboh-C      ----------------------------KEKFFKYLGDDFDF-QPT-YSDILRMSVGYSG 160
mPp-rboh-B      ----------------------------DDKFFKYLGDEFEK-HPT-YANIAVMPVAITG 160
mPp-rboh-D      ----------------------------DEKFFEALGDQFDK-HPT-YADIAVTPVAITG 169
Ps-rboh-B       ----------------------------RSIFVTRHVSSFVNGERPPFITMLQSLPVWTG 165
Ps-rboh-C       ----------------------------RSIFVTRHVSTFVNGERPPFTTMLQALPVWTG 152
Ps-rboh-A       ----------------------------K--QSRYHHLAFVRDEN--LLLFALRVPIWTG 156
Hs-Duox1        ---------------------------LSCLFPGLFHDDGSELPQKYYWWFFQTVPGLTG 159
Hs-Duox2        ---------------------------LACIFPNVFVNDGSKLPQKFYWWFFQTVPGMTG 159
Lg-Duox1        ---------------------------LNCYFREYFR--ATDVLATFHYWAFTTITGITG 167
Lg-Duox2        ---------------------------VSCLFREYFR--GSHEIATFHYWAYQTITGLAG 167
Lg-Duox3        ---------------------------LCIFDSIVFR---ADKLPTFSFWLFGNMTGFTG 167
Dp-Duox         ---------------------------LRCLSKEISLP--SDYKPTITYWLFQTLTGLTG 167
Cb-Duox         ---------------------------LACLFQEAFFG--SNFLPSISYWFYGTITGLTG 167
Mg-NoxC         --------------------------------DAVGVLLGEDQVPRPYSAYVSSLPGITG 158
Fg-NoxC         --------------------------------DAVAEALGPDKVPRPYIDYVRSLPGFTG 158
Cm-NoxD2        -------------------------------FLPFSNTIP------------TTMVFVTG 147
Cm-NoxD1        -------------------------------LFTGPAALP------------ATMLFVTG 146
Pt-NoxD         --------------------------------PIWLWGDG----------LATKQLLVTG 151
An-Fre1         -AVSGPIECKQIPTPLSSISYALRTYIVVPASFTPIFSHHQRLLYGHSIPKRLDFLIVSG 276
Sc-Fre1         ----------------------VKKSLIYPSVYKDYNERTFYLWKRLPFNFTTRGKGLVV 219


Dp-Nox5A        WGLCVILGVMIPCSLPFVRKTG-------------------------------------- 176
Dp-Nox5B        WALVGILIIMGLCSLPCVRKSG-------------------------------------- 180
Hs-Nox5         VALLLLLLLMFICSSSCIRRSG-------------------------------------- 162
Lg-Nox5A        VILDVILIVMIICSMPFIRRSG-------------------------------------- 170
Lg-Nox5B        VLLCPILIVMVICSMSFVRRSG-------------------------------------- 171
Dd-NoxC         FIMISFLIIMAILSLKIIRKS--------------------------------------- 179
Mg-NoxA         HMMLLCMLLMYTTAHH-RIRQQ-------------------------------------- 167
Fg-NoxA         HIMLLCMLLMFTSAHA-RIRQQ-------------------------------------- 171
Cc-NoxA         HFMLLIMVVMYTTAHQ-KIRKQ-------------------------------------- 168
Lb-NoxA         HFMLLIMVLMYSTAHQ-KIRQQ-------------------------------------- 168
fPp-NoxA        HFMLLCMVLMYTTAHH-KVRQQ-------------------------------------- 168
Pg-NoxA         HIMLFIMVLMYTTAST-KIRTQ-------------------------------------- 174
Bd-NoxA         HIMLIIMFFMYTSAKS-DVKTK-------------------------------------- 163
Mg-NoxB         YVMLIALMGMVFTSVE-KPRRA-------------------------------------- 164
Fg-NoxB         YVMLIALMGMVLTSME-KPRRA-------------------------------------- 164
Cc-NoxB         WIMWLALGIMVWFAIE-KRRRA-------------------------------------- 175
Lb-NoxB         WIMTVSLGIMVFFASE-KRRRA-------------------------------------- 186
fPp-NoxB        WIMTAALAIMVWYAVE-KRRRA-------------------------------------- 176
Pg-NoxB         WIMTLSLGIIVWYARE-KPRRA-------------------------------------- 170
Bd-NoxB         QVISVSFFLIFTSAME-AVRRK-------------------------------------- 148
Hs-Nox2         VVITLCLILIITSSTK-TIRRS-------------------------------------- 200
Hs-Nox3         LVISLALVLIMTSSTE-FIRQA-------------------------------------- 198
Hs-Nox1         VIMTIALILMVTSATE-FIRRS-------------------------------------- 199
Lg-Nox2         VIITLCLILIVSSSTE-TIRRW-------------------------------------- 195
Nv-Nox2A        AVITLALILMLSSSTE-LIRRS-------------------------------------- 194
Nv-Nox2B        AVITLCLVVMVSSSTE-LIRRS-------------------------------------- 185
Ci-Nox2         VIITLSLFFMVTSSLE-FIRRS-------------------------------------- 211
Sp-Nox2A        AVLTLALILMFSSATE-FIRRS-------------------------------------- 212
Sp-Nox2B        AVLALTYILMFTSATE-FIR-Y-------------------------------------- 187
Mb-Nox2         VGITVPLILMVTTASQ-QIRRS-------------------------------------- 186
Hs-Nox4         VCMVVVLFLMITASTY-AIRVS-------------------------------------- 185
Nv-Nox4         MGMMLVLLIMIAASTP-IVRNR-------------------------------------- 171
Lg-Nox4         LLMALILVLIVTSSYK-HIRDS-------------------------------------- 188
Dd-NoxA         HVVCIVMVLMYTSAVESIRRP--------------------------------------- 181
Dd-NoxB         HIMLLILILIVSSSMWRIRRP--------------------------------------- 175
Ng-NoxA         NVLCIVMFIMYAASAKSYRRTK-------------------------------------- 175
Os-rboh-C       VVMVVLMAIAFTLAQPWFRRNK---------------------------------LKDSN 189
Os-rboh-G       VIMVVLMAIAYTLAHPWFRRSK---------------------------------LSDSN 189
At-rboh-D       IVMVVLMAIAFTLATPWFRRNK---------------------------------LNLPN 185
Os-rboh-A       VIMVVCMIIAFTLATRWFRRSL---------------------------------VKLPR 187
Os-rboh-B       VIMLVCMIIAFTLATRWFRRSL---------------------------------VKLPK 187
```

```
Os-rboh-D       VAMVVLMTVSFTLATHPFRKGEKGGS-----GGGAAATV------------LPTVARLPS 203
Os-rboh-H       IVMVVLMVVSFTLATRPLRKRE--------------------------------APRLPF 247
Os-rboh-E       ILMIIIMSFSFTLATHSFRRSV---------------------------------VKLPS 188
Os-rboh-F       ILMILIMSFSFTLATHSFRRSV---------------------------------VKLPS 188
mPp-rboh-A      VIMLVIMIIAFLLATHWFRRSL---------------------------------VKLPW 190
mPp-rboh-C      LIMVVLMILAFLLATHWFRQSL---------------------------------VKLPW 187
mPp-rboh-B      ILMVVFMIIAFLLATHWFRRSM---------------------------------VKLPW 187
mPp-rboh-D      ILMVVLMAIAMLLATHWIRRSL---------------------------------VKFPW 196
Ps-rboh-B       VILLVITCISFPLAAIPKFRQG-------------------------------------- 187
Ps-rboh-C       VILLVITCISFPLAAIPKFRQG-------------------------------------- 174
Ps-rboh-A       VAMLLCAAIAAPLC-LEKVRRG-------------------------------------- 177
Hs-Duox1        VVLLLILAIMYVFASHHFRRRS-------------------------------------- 181
Hs-Duox2        VLLLLVLAIMYVFASHHFRRRS-------------------------------------- 181
Lg-Duox1        VILTLIIIVMYVFATEYGRRHL-------------------------------------- 189
Lg-Duox2        VVVTIVIFIMYVFATQFARRNH-------------------------------------- 189
Lg-Duox3        VLLVVVLCIMYVFATQTARSHI-------------------------------------- 189
Dp-Duox         VLLFIVMIIIFVFAHPIIRKKA-------------------------------------- 189
Cb-Duox         IALVTVMCIIYVFALPCFIKRA-------------------------------------- 189
Mg-NoxC         LTALGLFYTLALLSMPQVRRWN-------------------------------------- 180
Fg-NoxC         ITALGLFWILCLLSIPQVRRWN-------------------------------------- 180
Cm-NoxD2        CALMAFLLAIVLLSLGSVRRRV-------------------------------------- 169
Cm-NoxD1        VVLMVLLVPLLGFGVETVRRKW-------------------------------------- 168
Pt-NoxD         IFLFWIFAGFFMTTLERVRRTTWG------------------------------------ 175
An-Fre1         FWILCVLLACVNYNGAIRTSTMPQRN-----WHYTSDRTGILAYACLPFLWLFTGRNNIF 331
Sc-Fre1         LIFVILTILSLSFGHNIKLPHPYDRPRWRRSMAFVSRRADLMAIALFPVVYLFGIRNNPF 279


Dp-Nox5A        ------YFELFYWTHF-LHYPFWALLILHGPH---------------------------- 201
Dp-Nox5B        ------SFEVFYWTHL-LYIPFWVLLLIHGPN---------------------------- 205
Hs-Nox5         ------HFEVFYWTHL-SYLLVWLLLIFHGPN---------------------------- 187
Lg-Nox5A        ------HFQVFYWTHI-LYVPFWILCILHATN---------------------------- 195
Lg-Nox5B        ------HFEIFYYTHM-LYIPFWILCIIHASN---------------------------- 196
Dd-NoxC         ------NFELFYYSHH-LFIGFYVLLILHGTMGWIRPPT--------------------- 211
Mg-NoxA         ------SFETFWYTHH-LFIPFFLGLYTHTVGCFVRDTVE-------------------- 200
Fg-NoxA         ------SFETFWYTHH-LFIPFFLGLYTHTVGCFVRDTPE-------------------- 204
Cc-NoxA         ------CFEAFWYTHH-LAFFFMLGLYTHATGCFVRDSVH-------------------- 201
Lb-NoxA         ------CFEAFWYTHH-LAFFFMLGLYTHATGCFVRDTVD-------------------- 201
fPp-NoxA        ------CFEAFWYTHH-LAFFFFIALWTHADGCFVRDSTN-------------------- 201
Pg-NoxA         ------CFEAFWYTHH-LAFFWALCLYTHAAGCFVRGALP-------------------- 207
Bd-NoxA         ------NFEYFWYTHH-LFVPFYFCLFFHSFGCFVKSST--------------------- 195
Mg-NoxB         ------NYERFWYTHH-FFIVFFFFWSIHGAFCMIQPDFA-------------------- 197
Fg-NoxB         ------NFERFWYTHH-MFIVFFFFWSIHGAFCMIQPDVA-------------------- 197
Cc-NoxB         ------HFERFWYSHH-LFIVFFINWQLHGMFCMIKPDRP-------------------- 208
Lb-NoxB         ------HFERFWYSHH-LFVVFFINWQLHGMFCMIKPDRP-------------------- 219
fPp-NoxB        ------NFEKFWYTHH-LFIVFFLNWQLHGMFCMIQPDRE-------------------- 209
Pg-NoxB         ------KFERFWYSHH-LFIVFFSAWQLHGMFCMIQPDRP-------------------- 203
Bd-NoxB         ------YFEIFWFTHH-LFLVFFGALLMHGSFCFIKGDSG-------------------- 181
Hs-Nox2         ------YFEVFWYTHH-LFVIFFIGLAIHGAERIVRGQTA-------------------- 233
Hs-Nox3         ------SYELFWYTHH-VFIVFFLSLAIHGTGRIVRGQTQ-------------------- 231
Hs-Nox1         ------YFEVFWYTHH-LFIFYILGLGIHGIGGIVRGQTE-------------------- 232
Lg-Nox2         ------YFELFWYTHH-LFIIFLIGFVIHGIQGIIRHQTN-------------------- 228
Nv-Nox2A        ------YFEVFWFNHH-CFVIFYIGLVLHGVQGIIRYQS--------------------- 226
Nv-Nox2B        ------YFEVFWYSHH-LFIIFFAGLVAHGCGEILRYQT--------------------- 217
Ci-Nox2         ------YFEVFWFTHH-LFIVFYGFLVVHGISMQVRGQTP-------------------- 244
Sp-Nox2A        ------YFETFWITHH-LFIVYFAMLLAHGVGGIIRSQT--------------------- 244
Sp-Nox2B        ------YFETFWLTHH-LFVIYYAMLMTHGMGGVVKYQT--------------------- 219
Mb-Nox2         ------YFELFWYTHH-LFVVFYVCLCLHGYSGFVERQDNPDTYPISLDRGVCTSRDDVL 239
Hs-Nox4         ------NYDIFWYTHN-LFFVFYMLLTLHVSGGLLKYQTNLDTHPPGC------------ 226
Nv-Nox4         ------SYEVFWFTHH-AFIAFYLMLAVHGLGGVIKHQTNLAAHTPGCK----------- 213
Lg-Nox4         ------NYEVFYYTHR-LIFIFYGLLLIHAVK---------------------------- 213
Dd-NoxA         ------MFEGFWYTHH-LFVVFFGLLVVHGLHSILEP----------------------- 211
Dd-NoxB         ------MFEIFWYVHH-LFIPFYILLCFHGYSKILKKD---------------------- 206
Ng-NoxA         ------NFTVFWYAHH-LFVVFFVLLLVHG------------------------------ 198
Os-rboh-C       PLKKMTGFNAFWFTHH-LFVIVYTLLFVHGTCLYLSRKWY-------------------- 228
```

```
Os-rboh-G       PLKRLSGFNMFWYSHH-LFVIVYIAFVVHGVCLYINRTWW-------------------- 228
At-rboh-D       FLKKLTGFNAFWYTHH-LFIIVYALLIVHGIKLYLTKIWY-------------------- 224
Os-rboh-A       PFDKLTGFNAFWYSHH-LFIIVYIALIVHGECLYLIHVWY-------------------- 226
Os-rboh-B       PFDKLTGFNAFWYSHH-LFIIVYISLVIHGEWLYLIRIWY-------------------- 226
Os-rboh-D       PFNRLAGFNAFWYSHH-LLGIVYALLIAHGYFLFLVRRWY-------------------- 242
Os-rboh-H       PLGHLAGFNAFWYSHH-LLIVVYLLLLVHGWFMFLVTKWH-------------------- 286
Os-rboh-E       PLHHLAGFNAFWYAHH-LLVLAYVLLVVHSYFIFLTREWY-------------------- 227
Os-rboh-F       PLHHLAGFNAFWYAHH-LLVIAYILLVLHSYFIFLTKQWY-------------------- 227
mPp-rboh-A      PFHRLMGFNAFWYSHH-LFVIVYALLLLHGTKLLLPSPWH-------------------- 229
mPp-rboh-C      PFHRLTGFNAFWYSHH-LFAIVYAFLLLHGSKLLLPNSIL-------------------- 226
mPp-rboh-B      PLQRLTGFNAFWYSHH-LFVIVYALLMVHSIKLLLAGPWY-------------------- 226
mPp-rboh-D      PLHRLTGFNTFWYSHH-LFVIVYALLLVHSIKLLLAGSWY-------------------- 235
Ps-rboh-B       ------KFNVFWYSHM-LFGPFLLVLCFHGACSWLAR----------------------- 217
Ps-rboh-C       ------KFNLFWYSHM-LFGPFLVVLAFHGATSWLAR----------------------- 204
Ps-rboh-A       ------KFNLFWVSHM-LFIPFLVLMAFHGFARWVAA----------------------- 207
Hs-Duox1        -------FRGFWLTHH-LYILLYVLLIIHGSFALIQL----------------------- 210
Hs-Duox2        -------FRGFWLTHH-LYILLYALLIIHGSYALIQL----------------------- 210
Lg-Duox1        -------FNAFWFTHN-FYVFLYIFLILHGIGRLVQD----------------------- 218
Lg-Duox2        -------FTAFWLTHS-LYLTVYPLTILHGIGVLVQA----------------------- 218
Lg-Duox3        -------FNLFWLTHK-LFIIMYVLVILHGASIIVQK----------------------- 218
Dp-Duox         -------YNFFWMTHS-LYIVLYILSVLHGLGRLTAE----------------------- 218
Cb-Duox         -------YHAFRLTHL-LNIAFYALTILHGLPKLLDS----------------------- 218
Mg-NoxC         -------YEVFQLAHL-LMFPIIGLLAAHGTAQLLQYAMFG------------------- 213
Fg-NoxC         -------YEVFQLGHL-LMFPIIGLMMAHGTAALLQWPMFG------------------- 213
Cm-NoxD2        -------YRVFWASHVPLAILTYAALVFHGLRGGR------------------------- 197
Cm-NoxD1        -------FRLFWWTHRPIAVLVYICLVFHGLRGGR------------------------- 196
Pt-NoxD         -------FRLFWVAHMISITCVLPLLIIHGTIRGK------------------------- 203
An-Fre1         LWATNFDIQSFSTFHRHIAWACTLLAIVHSIGYSIILADYEDAYHEAWSHKPWYMG---- 387
Sc-Fre1         IPITGLSFSTFNFYHKWSAYVCFMLAVVHSIVMTASGVKRGVFQSLVRKFYFRWG----- 334
                          *   *             *

Dp-Nox5A        ------------------------------------------------------------
Dp-Nox5B        ------------------------------------------------------------
Hs-Nox5         ------------------------------------------------------------
Lg-Nox5A        ------------------------------------------------------------
Lg-Nox5B        ------------------------------------------------------------
Dd-NoxC         ------------------------------------------------------------
Mg-NoxA         --------------------PHSPFAGDEY----------------------WNHCIGYL 218
Fg-NoxA         --------------------AFSPFAGDEF----------------------WEHCIGYL 222
Cc-NoxA         --------------------PNLTSTFPFYS---------------------TENCIGYL 220
Lb-NoxA         --------------------PDYIPTFPFYS---------------------TEHCLGYL 220
fPp-NoxA        --------------------AAYIDTFPFYN---------------------AEFCLGYE 220
Pg-NoxA         --------------------DHK------------------------------AQCLGYN 217
Bd-NoxA         ----------------------------------------------------TGKCKGYN 203
Mg-NoxB         --------------------PFCMS---------------------------FGTSAIGV 210
Fg-NoxB         --------------------PFCTS---------------------------IGSSAIGV 210
Cc-NoxB         --------------------PYCS------------------------------FNTIGV 218
Lb-NoxB         --------------------PYCS------------------------------SNTIGV 229
fPp-NoxB        --------------------PFCT------------------------------WNSIGV 219
Pg-NoxB         --------------------PYCS------------------------------FNQIGV 213
Bd-NoxB         --------------------DPCR--------------------------------GGPM 189
Hs-Nox2         -----------------ESLAVHNITVCE-------QKISEWG-KIKECPIPQFAGNPPM 268
Hs-Nox3         -----------------DSLSLHNITFCR-------DRYAEWQ-TVAQCPVPQFSGKEPS 266
Hs-Nox1         -----------------ESMNESHPRKCA-------ESFEMWDDRDSHCRRPKFEGHPPE 268
Lg-Nox2         -----------------VSS--HNPEKCY-------TRHDEWG-TSPDCKIPQFAGSSPK 261
Nv-Nox2A        ------------------NVDKHDPEIC--------MNHTLWYNHPKCMEPPKFVPFGMN 260
Nv-Nox2B        ------------------NMDKHDPDVCRK-----QENLATWGVKDPCRVLPEFEAAGAM 254
Ci-Nox2         -----------------QSLTVHDPIRCS------TIDPATWSQNN-CP-TPVFAGSPPM 279
Sp-Nox2A        ------------------NLDRHDVVFCS-------ENLDVWGPTSAQCEDPVFKDGSAA 279
Sp-Nox2B        ------------------NVDEHDPVECM-------VDEETFDQCVID-NPPLFAGTPGA 253
Mb-Nox2         VSNGCASWNAVVSALANQNSSLTEPNLCIPGGVDVGDEEDAFCCPCRLVTQAVLQRGQAA 299
Hs-Nox4         ISLNR---TSSQNISLPEYFSEHFHEPFPEGFSKP-AEFTQHKFVKICMEEPRFQANFPQ 282
Nv-Nox4         VPLNDSMDTPFNMTSLPLCREAEFAPDQPQVYHKSNTQVYQKPYHTVYCK--CYHTGIPQ 271
Lg-Nox4         -----------------------------------------------------------E 214
```

```
Dd-NoxA         ----------------------------------------------------------TS 213
Dd-NoxB         ----------------------------------------------------------PQ 208
Ng-NoxA         ----------------------------------------------------------KQ 200
Os-rboh-C       ---------------------------------------------------------KKT 231
Os-rboh-G       ---------------------------------------------------------KQT 231
At-rboh-D       ---------------------------------------------------------QKT 227
Os-rboh-A       ---------------------------------------------------------RRT 229
Os-rboh-B       ---------------------------------------------------------KRT 229
Os-rboh-D       ---------------------------------------------------------LKT 245
Os-rboh-H       ---------------------------------------------------------QRT 289
Os-rboh-E       ---------------------------------------------------------KKT 230
Os-rboh-F       ---------------------------------------------------------NRT 230
mPp-rboh-A      ---------------------------------------------------------ERT 232
mPp-rboh-C      ---------------------------------------------------------ERS 229
mPp-rboh-B      ---------------------------------------------------------KRT 229
mPp-rboh-D      ---------------------------------------------------------KRT 238
Ps-rboh-B       ----------------------------------------------------------SS 219
Ps-rboh-C       ----------------------------------------------------------CS 206
Ps-rboh-A       ----------------------------------------------------------PQ 209
Hs-Duox1        ----------------------------------------------------------PR 212
Hs-Duox2        ----------------------------------------------------------PT 212
Lg-Duox1        ----------------------------------------------------------PL 220
Lg-Duox2        ----------------------------------------------------------PI 220
Lg-Duox3        ----------------------------------------------------------PL 220
Dp-Duox         ----------------------------------------------------------PN 220
Cb-Duox         ----------------------------------------------------------PK 220
Mg-NoxC         ------------------------------------------------------------
Fg-NoxC         ------------------------------------------------------------
Cm-NoxD2        ----------------------------------------------------------LW 199
Cm-NoxD1        ----------------------------------------------------------PW 198
Pt-NoxD         ----------------------------------------------------------PI 205
An-Fre1         ---------VIAIIAMSVLLIHSVTWLRRKWYEVFLLSHIILAIVIIYALFQHTRPDGPQ 438
Sc-Fre1         -----------IVATILMSIIIFQSEKVFRNRGYEIFLLIHKAMNIMFIIAMYYHCHTLG 383

Dp-Nox5A        FWYWLVGPGALFIILEKLLRLRNRMTGKGRTFINYAILLPSS------------------ 243
Dp-Nox5B        FWYWFIGPGILFFIEG-TGRFRLRVTGKGRTFISSALLLPSR------------------ 246
Hs-Nox5         FWKWLLVPGILFFLEK---AIGLAVSRMAAVCIMEVNLLPSK------------------ 226
Lg-Nox5A        FWYWFIIPGIIFIIESLNRSKYIKKAAYGATYITNVYLLPYR------------------ 237
Lg-Nox5B        FWKWFIAPGLIFFVESILRSKPMKLATHGRTFIKEVNLLPSG------------------ 238
Dd-NoxC         FWKWFIVPGFFYTVDR----SFRLFKRTHRVEVLDYCLKNER------------------ 249
Mg-NoxA         GWRWELWTGGFYLLER-----------LYREIRARRETKITR-VVRHPYGEFFFFLFFLL 266
Fg-NoxA         GWRWELWTGGAYLLER-----------LWREVRARRSTKITR-VVRHPY----------- 259
Cc-NoxA         SWRFIIWPGIIYFGER-----------VWREIRARRATRLSK-VLVHPSG---------- 258
Lb-NoxA         SWRFIIWPGIIYFGER-----------VWREIRARRATRLSK-VLVHPSG---------- 258
fPp-NoxA        SWRFTIWPGIIYFGER-----------VWREVRARRATRLSK-VLVHPSG---------- 258
Pg-NoxA         SVYVTVWSGLAYFCDR-----------VFREIRGRGRSEISA-VLIHPSG---------- 255
Bd-NoxA         TNYGTVPIFCVYIAER-----------LLREYRARLPTVLSK-VIFHSGN---------- 241
Mg-NoxB         FWQFWMYGGFVYMAER-----------IAREIRGKHKTYISK-VIQHPSN---------- 248
Fg-NoxB         FWQFWMYSGFCYLAER-----------IAREVRGRHRTFISK-VIQHPSN---------- 248
Cc-NoxB         FWRYWLVGGVIWIWER-----------ILREIRSRHRTHIHK-VIQHPSS---------- 256
Lb-NoxB         FWRYWLIGGVIWISER-----------VLREIRSRHRTYVSK-VIQHPSN---------- 267
fPp-NoxB        FWRYWLTGGVIFTFER-----------ILREVRSRHRTYISK-VIQHPSN---------- 257
Pg-NoxB         FWKYWLVGGTIFIWER-----------VLREVRSRHKTYISK-VIQHPSN---------- 251
Bd-NoxB         FWKFWVGSAAFYLIER-----------LWREISGRRKTYIFK-VVQHPSK---------- 227
Hs-Nox2         TWKWIVGPMFLYLCER-----------LVRFWRSQQKVVITK-VVTHPFK---------- 306
Hs-Nox3         AWKWILGPVVLYACER-----------IIRFWRFQQEVVITK-VVSHPSG---------- 304
Hs-Nox1         SWKWILAPVILYICER-----------ILRFYRSQQKVVITK-VVMHPSK---------- 306
Lg-Nox2         SWIWILIPVIIFIIER-----------GIRFYRSLQQVVITK-VVKHPSN---------- 299
Nv-Nox2A        TWRWVVGPMFLYCIER-----------LIRFVRSHQAIQIIK-VVKHPSN---------- 298
Nv-Nox2B        TWKIVLLPMVIYFLER-----------CLRFWRSMQQVKVVKVVVKHPSR---------- 293
Ci-Nox2         TWKWVIAPMVLYVIER-----------IIRLVRFNQQVEVLK-VIKHPSR---------- 317
Sp-Nox2A        SYKWVSGPLFIYLLER-----------TIRFWRSCQTVTLTK-VVKHQSK---------- 317
Sp-Nox2B        SWKWCVTPLCVYFLER-----------ILRMIRTWPDVTIVQ-VVQHQSK---------- 291
```

```
Mb-Nox2        TWVWVIGPLILYILER-----------LYRWYKSQTRRLRILKVVKHKDS---------- 338
Hs-Nox4        TWLWISGPLCLYCAER-----------LYRYIRSNKPVTIIS-VMSHPSD---------- 320
Nv-Nox4        SWKLLILPLSIYLIDR-----------LIRVVRGYQEVTVIK-VVNHPCG---------- 309
Lg-Nox4        SWVWIVVPLILYIVDG-----------IIRIFN-RKTTTITK-ITAYTGD---------- 251
Dd-NoxA        FWKWVIGPCALYIVER-----------LIRLLRSKKTTMLIQ-ARIHPSR---------- 251
Dd-NoxB        SWMWIIAPFILYSIER-----------LIRIARSKKRVILEK-AIMHPSK---------- 246
Ng-NoxA        FWAWFFIPMALYALER-----------VMRNLRGSEMTIVKR-VHCLASR---------- 238
Os-rboh-C      TWMYLAVPVVLYVSER-----------ILRLFRSH--DAVGIQKVAVYPG---------- 268
Os-rboh-G      TWMYLAIPILLYAGER-----------IFRALRSHGFTTVRIEKVAIYPG---------- 270
At-rboh-D      TWMYLAVPILLYASER-----------LLRAFRSS-IKPVKMIKVAVYPG---------- 265
Os-rboh-A      TWMYLSVPVCLYVGER-----------ILRFFRSG-SYSVRLLKVAIYPG---------- 267
Os-rboh-B      TWMYLAVPVCLYVGER-----------TLRFFRSG-SYSVRLLKVAIYPG---------- 267
Os-rboh-D      TWMYISVPLMLYVGER-----------MLRALRSN-AYAVKILKVCLLPG---------- 283
Os-rboh-H      TWMYIAVPLMLYVGER-----------TLRAFRSK-AYAVKILKVCLLPG---------- 327
Os-rboh-E      TWMYLIVPVLFYACER-----------TIRKVREN-NYRVSIVKAAIYPG---------- 268
Os-rboh-F      TWMFLAVPVLFYSCER-----------TIRRVRES-SYGVTVIKAAIYPG---------- 268
mPp-rboh-A     TWMYIAVPLLLYAGER-----------LLRMYRTN-SSKVDVIKAAIYSG---------- 270
mPp-rboh-C     TWIYIAVPLVLYAGER-----------FLRMYRTN-SSKVDVIKAAIYTG---------- 267
mPp-rboh-B     TWMYIAIPLLLYAGER-----------MLRLYRTN-SSKVEIVKAAIYTG---------- 267
mPp-rboh-D     IWMYTAVPLVLYASER-----------FLRLYRTN-YSKVEVVKAAVYTG---------- 276
Ps-rboh-B      SYIWITPPFLIYLIER-----------RFRYAKMF--AAPVRIMEAMELD---------- 256
Ps-rboh-C      AYIWITPPFLMYLIER-----------RFRYAKMF--AAPVRIMEAMELD---------- 243
Ps-rboh-A      AHYWVLPPIVIYLVEK-----------RYRMTQMF--GGQTQIAHVQLSK---------- 246
Hs-Duox1       FHIFFLVPAIIYGGDK-----------LVSLSRKKVEISVVKAELLPSG----------- 250
Hs-Duox2       FHIYFLVPAIIYGGDK-----------LVSLSRKKVEISVVKAELLPSG----------- 250
Lg-Duox1       FPYFFLGPLVVFVLDK-----------MVSLSRNKVEIVVKKAEILPSG----------- 258
Lg-Duox2       FPYYLFGPLVLFVFDK-----------LASLSRNRVEIRVKKVSILPSD----------- 258
Lg-Duox3       FFAYFIGPAALFTLDK-----------MISLSRKKTEINIVKAINLPSD----------- 258
Dp-Duox        FWVFLIGPAIVYTLDK-----------IISLRTKFMGLDIIETVLLPSD----------- 258
Cb-Duox        FGYYVLGPVIIFVIDR-----------IIGLMQYYKKLEIVSAEILPSD----------- 258
Mg-NoxC        --YWLAVPTILVLTER-----------LVRVGTGFHRIPASLKVLDDETV---------- 250
Fg-NoxC        --YFLAFPTLLVLVER-----------TVRVGLGFHRIKATMKVLDKETV---------- 250
Cm-NoxD2       SVYFFGLPVVLYILDR-----------LYHSFAAAFTPHRVLSVELSHKNS--------- 239
Cm-NoxD1       TVYFFAGPVFLYIIDR-----------LYQLSRATVTPRRILVLSLAQANS--------- 238
Pt-NoxD        LMYTASLPIAIYIVDS----------FMRRMMYKTVQAGVVRFQAFGGEGQGGGE----- 250
An-Fre1        WNPYLRTVVAIWMFDR--------IFRIARIAYCNLQIRFSELQISLPSSSVTYNPDS-- 488
Sc-Fre1        WMGWIWSMAGILCFDR-----------FCRIVRIIMNGGLKTATLSTTDDS--------- 423


Dp-Nox5A       -----------------VTHLVCRKPP-NFNFHA----GDYVVLNIPAIAS------YEW 275
Dp-Nox5B       -----------------VTHLVIRKPE-NFNFNP----GDYVFVKIPAITA------SEW 278
Hs-Nox5        -----------------VTHLLIKRPP-FFHYRP----GDYLYLNIPTIAR------YEW 258
Lg-Nox5A       -----------------VIHLVISRPP-KWRYRP----GDYVFIQIPAIAK------YEW 269
Lg-Nox5B       -----------------VTHLVISRPP-NFDFKP----GDYLFIQIPMLAK------YEW 270
Dd-NoxC        -----------------VINLTFSKPP-SFDYKP----GQYLLINVPHISK------LQW 281
Mg-NoxA        FGPSMVGFSVLIRDSADVVEIQFNKPS--FKYKA----GQWLFLQVPSVSK------YQW 314
Fg-NoxA        ----------------DVVEIQFNKPS--FKYKA----GQWLFLQVPSLSK------YQW 291
Cc-NoxA        -----------------AMELRIVKP---FKYTA----GQWLFLQVPELSR------FQW 288
Lb-NoxA        -----------------AMELRIIKPS--FKYTA----GQWLFIQIPELSR------FQW 289
fPp-NoxA       -----------------AMELRIVKPS--FKYVA----GQWLFIQVPEVSK------FQW 289
Pg-NoxA        -----------------TVEIRMLKAG--FKYVP----GQWIFFQMPEVSR------FQW 286
Bd-NoxA        -----------------TMELRIEKPS--FQYMP----GQYLFLNIPSISA------FQW 272
Mg-NoxB        -----------------VCEIQIKKEH--TKTRA----GQYIFFCCPEVSV------WQY 279
Fg-NoxB        -----------------VCEIQMKKEH--TKTRA----GQYIFLCCPAVSL------WQY 279
Cc-NoxB        -----------------VMEVQIKKEK--TTTRA----GQYIFLSCPEISY------FQW 287
Lb-NoxB        -----------------VMELQIKKEK--TTTRA----GQYIFLSCPEISY------FQW 298
fPp-NoxB       -----------------VMELQIKKEK--TTTRA----GQYIFLSCPEISY------FQW 288
Pg-NoxB        -----------------VCEVQIKKEK--TTTRA----GQYIFLNCPEVSY------WQW 282
Bd-NoxB        -----------------VVEVQIKKNG--WKMQA----GQYIFICCPEIGL------FEW 258
Hs-Nox2        -----------------TIELQMKKKG--FKMEV----GQYIFVKCPKVSK------LEW 337
Hs-Nox3        -----------------VLELHMKKRG--FKMAP----GQYILVQCPAISS------LEW 335
Hs-Nox1        -----------------VLELQMNKRG--FSMEV----GQYIFVNCPSISL------LEW 337
Lg-Nox2        -----------------VFELQIRKKS--FYADP----GQYIFLHCPSISL------LEW 330
Nv-Nox2A       -----------------VIEIKMKKAG--FRPEV----GQYIFLQCPKISK------LEW 329
```

```
Nv-Nox2B        -----------------VVEIQMKKPG--FVCEA----GQYVFLQVPKISQ------LEW 324
Ci-Nox2         -----------------VLEIQMRKNG--FFAEV----GQYVFIMCPQLSQ------LEW 348
Sp-Nox2A        -----------------VIELQMKKKG--FKMEA----GQYIFLKCPSISH------VQW 348
Sp-Nox2B        -----------------VIELRMKKQG--FKMLP----GQYIFLKCSPISK------VQW 322
Mb-Nox2         -----------------VPVMEIQFQK--VPTKA----GQYVFINCPKINS------LEW 369
Hs-Nox4         -----------------VMEIRMVKEN--FKARP----GQYITLHCPSVSA------LEN 351
Nv-Nox4         -----------------VIELHMKKSG--FYAEP----GQFVYVRCHSVAR------FEW 340
Lg-Nox4         -----------------VIELQLKCDG--LKAIP----GQYVLLKCPRISL------FEW 282
Dd-NoxA         -----------------VIEVRMKTER--FKYKP----GQYLFLNCPTIAQ------NEW 282
Dd-NoxB         -----------------VLELRMKRDNDNFNFKP----GQYLYLNCPSIAY------HEW 279
Ng-NoxA         -----------------VIHLELEKPS--FRYES----GQYCFLNCPMISQ------HEW 269
Os-rboh-C       ----------------NVLALYMSKPP-GFRYR----SGQYIFIKCTAVSP------YEW 301
Os-rboh-G       ----------------NVIAIHMTKPH-GFKYK----SGQYIYVNCG------------- 296
At-rboh-D       ----------------NVLSLHMTKPQ-GFKYK----SGQFMLVNCRAVSP------FEW 298
Os-rboh-A       ----------------NVLTLQMSKPP-TFRYK----SGQYMFVQCPAVSP------FEW 300
Os-rboh-B       ----------------NVLTLQMSKPP-TFRYK----SGQYMFVQCPAVSP------FEW 300
Os-rboh-D       ----------------NVLTITMSKPY-GFRYR----SGQYIFLQCPTISP------FEW 316
Os-rboh-H       ----------------NVLTITMSKPY-GFRYR----SGQYIFLQCPTISP------FEW 360
Os-rboh-E       ----------------NVLSLHMKKPP-GFKYK----SGMYLFVKCPDVSP------FEW 301
Os-rboh-F       ----------------NVLSIHMNKPS-SFKYK----SGMYMFVKCPDVSP------FEW 301
mPp-rboh-A      ----------------NVLAIHMSKPE-GFKYK----SGMYLFLQCPEISS------FEW 303
mPp-rboh-C      ----------------NVLAIHMSKPE-GFKYK----SGMYLFLQCPEISS------FEW 300
mPp-rboh-B      ----------------NVLAIHITKPQ-GFKYK----SGMYLFLQCPQISS------FEW 300
mPp-rboh-D      ----------------NVLAIHMTKPA-GFKYK----SGMYLFLQCPAISS------FEW 309
Ps-rboh-B       ----------------GTVALFMEKPR-RFVYR----PGMYMFVNCPLISS------HEW 289
Ps-rboh-C       ----------------STVALFIEKPR-RFVYR----PGMYLYINCPQISS------HEW 276
Ps-rboh-A       ----------------EAVAVFMRKPK-AFSKRQRFLPGMYVFVNVPTISK------FEW 283
Hs-Duox1        -----------------VTHLRFQRPQ-GFEYKS----GQWVRIACLALGT------TEY 282
Hs-Duox2        -----------------VTYLQFQRPQ-GFEYKS----GQWVRIACLALGT------TEY 282
Lg-Duox1        -----------------VTNLVFKRPL-NFDYKS----GQWVRIACLDLGQ------SEY 290
Lg-Duox2        -----------------VTELVFRRPA-NFDYRS----GQWVRIACLKLGK------GEY 290
Lg-Duox3        -----------------VTMLEFKRPP-KFEYKS----GQWVRIACLSHGS------NEY 290
Dp-Duox         -----------------VLKVKFYRPP-NFKYLS----GQWIRLSCTGVKP------EEF 290
Cb-Duox         -----------------IIYIEFRRPR-TFQYKS----GQWITVSSPSISCTF----NES 292
Mg-NoxC         -----------------ELRATIPSER-IWKYQA----GQWAYLQVPTISM------WQW 282
Fg-NoxC         -----------------EVTAIIPSER-LWKYKA----GQYIFLQVPKISF------FQW 282
Cm-NoxD2        ----------------NVVRLVLERRG--RKFVA----GQYFKLAWKFHVCSHLAVAGEW 277
Cm-NoxD1        ----------------NIVKLSVERRN--AVYLP----GQYFKIMIIDTRKGCGMPADAW 276
Pt-NoxD         ----------------KVVELILRSSA--YKYRP----GQYAEIQIPELSR------YEW 282
An-Fre1         ----------------DLIMIDTEAPP-HLALKP----GQHYFISQPLSVH-----CLES 522
Sc-Fre1         ----------------NVIKISVKKPK-FFKYQVG--AFAYMYFLSPKSAWFYS---FQS 461


Dp-Nox5A        HPFTISSAPELP-------DLIWFHIRGVGGWTNKLYEYFKQEQIKQY-----EFEHHSL 323
Dp-Nox5B        HPFTISSAPELP-------DVMWLHIRCAGGWTNKLYDYFEREQAKLCLKQSAQNQQHNG 331
Hs-Nox5         HPFTISSAPEQK-------DTIWLHIRSQGQWTNRLYESFK------------------- 292
Lg-Nox5A        HPFTISSAPEQE-------GTFWLHIRSAGHWTNKLYDYFDSYDPNTY------------ 310
Lg-Nox5B        HPFTISSAPEME-------GFIWLHIRSAGYWTKSLYDFFEKYGSDQR------------ 311
Dd-NoxC         HPFTMTSSP-LE-------DKIYVHIRVTGNWTKKLFRWLSIKKQLQQ------------ 321
Mg-NoxA         HPFTITSCPYD--------PYVSVHVRQVGDFTKALGDATGAGAAQAKLYEG-------- 358
Fg-NoxA         HPFTITSCPFD--------PYVSVHVRQVGDFTRELGDALGAGAAQAKLYDD-------- 335
Cc-NoxA         HPFTITSAPED--------PYVSVHIRQVGDFTNALGERLGVGPAAVASMTKAAVKGAEK 340
Lb-NoxA         HPFTITSAPED--------PYVSIHIRQVGDFTRGLGDRLGVGPSDKMSG---------- 331
fPp-NoxA        HPFTITSAPED--------PYVSVHIRQVGDFTQALGDRVGAGPSVVAAMTKAAMIGSEK 341
Pg-NoxA         HPFTISSAPDD--------PYISIHVRQVGDFTKAVGTRLGATPQLMATLNQPSE----- 333
Bd-NoxA         HPFTISSSPEE--------GFVSIHIRIVGDWTKNAAKMLGCYEQDIEKR---------- 314
Mg-NoxB         HPFTLTSAPEE--------DYISIHMRVVGDFTRGVSKALGCDWDRK---GDASKVV--- 325
Fg-NoxB         HPFTLTSAPEE--------DYISIHMRCVGDFTKELAKSLGCDWSKKKDAGDASKVV--- 328
Cc-NoxB         HPFTLTSAPEE--------DFISVHIRVAGDWTTAFSKALGCDFERK---KKGDD----- 331
Lb-NoxB         HPFTLTSAPEE--------DYISVHIRVVGDFTQALAKAMGCDFEKK---GKEETPA--- 344
fPp-NoxB        HPFTLTSAPEE--------DYISVHIRIVGDFTRELAAAVGCDFDSK---EKGVEA---- 333
Pg-NoxB         HPFTLTSAPEE--------DYISVHIRCVGDFTMEFAEALGCDFSRN---KEKSN----- 326
Bd-NoxB         HPFTLTSSPHE--------EFLSIHIRVVGDWTEKFAERVGCRFGGS------------- 297
Hs-Nox2         HPFTLTSAPEE--------DFFSIHIRIVGDWTEGLFNACGC--DKQE------------ 375
```

```
Hs-Nox3          HPFTLTSAPQE--------DFFSVHIRAAGDWTAALLEAFGA--EGQA------------ 373
Hs-Nox1          HPFTLTSAPEE--------DFFSIHIRAAGDWTENLIRAFEQ--QYS------------- 374
Lg-Nox2          HPFTLTSAPGE--------ETITVHVRRVGDWTEKLAKSCHV--DEGE------------ 368
Nv-Nox2A         HPFTLTSAPEE--------DTFSVHIRIVGDWTGDLAKLCGY--KGQK------------ 367
Nv-Nox2B         HPFTLTSAPEE--------DYFSLHIRVVGNWTTDLANQLG---AGNQ------------ 361
Ci-Nox2          HPFTLTSAPEE--------DYFSIHVRIVGDWTTGLSKVLGADEAGNE------------ 388
Sp-Nox2A         HPFTLTSAPEE--------DHFSVHIRVVGDWTRDLFKAMGA--DKPE------------ 386
Sp-Nox2B         HPFTLTSAPEE--------DYFSLHIRRVGDWTDELAVKMGA--DQAE------------ 360
Mb-Nox2          HPITLTSCPEL--------DYVSVHIRLVGDWTTKLADACGF--YEDN------------ 407
Hs-Nox4          HPFTLTMCPTETK------ATFGVHLKIVGDWTERFRD-LLLPPSSQDSEI--------- 395
Nv-Nox4          HPFTLTKCPSSKD------DSFSIHIKRTGDWTKSLSDQKPLLAALDDSGS--------- 385
Lg-Nox4          HPYSITKCPDKSS------SGFNLCLRHKGDWSGNIHK--------AFNTE--------- 319
Dd-NoxA          HPFTITSAPEE--------DFVSCHINVVGNWTGKLSTLLNPDKKMGIVQEN-------- 326
Dd-NoxB          HPFTITSAPDD--------PFISVHINIVGNWTRKLFKLLNPDNKLGLIQED-------- 323
Ng-NoxA          HPFTISSAPEE--------EFLQFHIRCVGDWTNTLMDVFNPAQRP-TVEIN-------- 312
Os-rboh-C        HPFSITSAPGD--------DYLSVHIRTRGDWTSRLRTVFSEACRPPTEGESGLLRADLS 353
Os-rboh-G        -----------------------------------------EICRPPMNGQSGLLRADCM 315
At-rboh-D        HPFSITSAPGD--------DYLSVHIRTLGDWTRKLRTVFSEVCKPPTAGKSGLLRADGG 350
Os-rboh-A        HPFSITSAPGD--------DYLSIHVRQLGDWTRELKRVFAAACEPPAGGKSGLLRADET 352
Os-rboh-B        HPFSITSAPGD--------DYLSIHVRQLGDWTRELKRVFSAACEPPVGGKSGLLRADET 352
Os-rboh-D        HPFSITSAPGD--------DYLSVHIRTNGDWTQELKRIFVENYFSPHLNRRASFSELGA 368
Os-rboh-H        HPFSITSAPGD--------DYISVHIQTRGDWTQELKRIFVENYFVPSVPRRASFGALGM 412
Os-rboh-E        HPFSITSAPGD--------DYLSVHIRTLGDWTTELRNLFGKACEAQVTSKKATLSRLET 353
Os-rboh-F        HPFSITSAPGD--------DYLSVHIRTLGDWTTELRNLFGKACEAQVSSKKATLARLET 353
mPp-rboh-A       HPFSITSAPED--------PFLSVHIRTLGDWTGEMMKIFADACGGRMRLQTVNNYGFIL 355
mPp-rboh-C       HPFSITSAPED--------PFLSVHIRTLGDWTAELKRIFSDACGGRMRLQTVNNYG--L 350
mPp-rboh-B       HPFSITSAPGD--------PFLSVHIRTLGDWTAEMKKIFSEACGGRTCLQTINNYG--L 350
mPp-rboh-D       HPFSITSAPDD--------PFLSVHIRTLGDWTTEMRKIFSDSLGGKTRLQAINDYG--L 359
Ps-rboh-B        HPFTISSAPGD--------NYISVHIRVCGDWTQALARVIADCHERKVL----------- 330
Ps-rboh-C        HPFTISSAPGD--------NYISIHIRVCGDWTDAMARVIADCHACNLQ----------- 317
Ps-rboh-A        HPFTISSAPED--------KFISLHIQRSGDWTRALYDNLQQHQASRVEDQGSPVTS--- 332
Hs-Duox1         HPFTLTSAPHE--------DTLSLHIRAAGPWTTRLREIYSAPTGDRC------------ 322
Hs-Duox2         HPFTLTSAPHE--------DTLSLHIRAVGPWTTRLREIYSSPKGNGC------------ 322
Lg-Duox1         HPFTLTSAPQE--------ENLSLHIRAVGPWTTNIRRVYDINNIVSE------------ 330
Lg-Duox2         HPFTLTSAPHE--------ENLSLHIRAVGPWTKNLRKIYEFYSIGN------------- 329
Lg-Duox3         HPFTLTSAPHE--------DTLKVHIRALGPWTWNIRQTFDLENLKD------------- 329
Dp-Duox          HSFTLTSAPHE--------NFLSCHIKAQGPWTWKLRNYFDPSNFNPK------------ 330
Cb-Duox          HAFSIASSPQD--------ETVKLYIKAVGPWTWKLRSELLR-AQNTG------------ 331
Mg-NoxC          HPFTISVCVGK---------EMRMHIKTDGNWTGRLRDLAKDAPQG-------------- 319
Fg-NoxC          HPFTVSFCRGN---------KMMLHIKTDGNWTAKLRELGGDS----------------- 316
Cm-NoxD2         HPFTVASSPLLDK------DRIIFFIAATGKWTNALRALALQQHQQQHDENLEHASSTSP 331
Cm-NoxD1         HPFTAASAPSCDS------DRITFYIAAVGKWTRRLHSLA--------EAAVAEAATTTA 322
Pt-NoxD          HPFTIASAPNSD-------NTVSFCIKALGRWTNGLFDLADTFQGGDVEEPK-------- 327
An-Fre1          HPFALGAYAQASSRSLKEKSRLTFYIRPYNGWTKTLRDRCSRAKN--------------- 567
Sc-Fre1          HPFTVLSERHRDPNNP---DQLTMYVKANKGITRVLLSKVLSAPNHTVD----------- 507


Dp-Nox5A         RPASPFVSSDQSAFQTNVSFGNQSATIASAKNATENQSPFGWTKTYRPYNPQISITLSEV 383
Dp-Nox5B         GAGEPACRHCQRILGNDASFSTQN-IMKGVRRLTRTFSNKNPVDKYDQLES-CRMTLRPL 389
Hs-Nox5          -ASDPLGR--------------------GSKRLSRSVTMRKSQRSSK------------- 318
Lg-Nox5A         -QYGTFNKGFG------------LDVMEAGGTIEEGTYRRLSSTGNTD------------ 345
Lg-Nox5B         -RKSTITGQIN------------KAFHDGLDKIERG-IKEVSTSFTHR------------ 345
Dd-NoxC          -------------------------QQQLYNNIKQQNVLPDGSNFIIN------------ 344
Mg-NoxA          ------------------------------------------------------------
Fg-NoxA          ------------------------------------------------------------
Cc-NoxA          ------------------------------------------------------------
Lb-NoxA          ------------------------------------------------------------
fPp-NoxA         ------------------------------------------------------------
Pg-NoxA          ------------------------------------------------------------
Bd-NoxA          ------------------------------------------------------------
Mg-NoxB          ------------------------------------------------------------
Fg-NoxB          ------------------------------------------------------------
Cc-NoxB          ------------------------------------------------------------
Lb-NoxB          ------------------------------------------------------------
```

```
fPp-NoxB      ------------------------------------------------------------
Pg-NoxB       ------------------------------------------------------------
Bd-NoxB       ------------------------------------------------------------
Hs-Nox2       ------------------------------------------------------------
Hs-Nox3       ------------------------------------------------------------
Hs-Nox1       ------------------------------------------------------------
Lg-Nox2       ------------------------------------------------------------
Nv-Nox2A      ------------------------------------------------------------
Nv-Nox2B      ------------------------------------------------------------
Ci-Nox2       ------------------------------------------------------------
Sp-Nox2A      ------------------------------------------------------------
Sp-Nox2B      ------------------------------------------------------------
Mb-Nox2       ------------------------------------------------------------
Hs-Nox4       ------------------------------------------------------------
Nv-Nox4       ------------------------------------------------------------
Lg-Nox4       ------------------------------------------------------------
Dd-NoxA       ------------------------------------------------------------
Dd-NoxB       ------------------------------------------------------------
Ng-NoxA       ------------------------------------------------------------
Os-rboh-C     KGITD------------------------------------------------------- 358
Os-rboh-G     S--ME------------------------------------------------------- 318
At-rboh-D     D----------------------------------------------------------- 351
Os-rboh-A     T----------------------------------------------------------- 353
Os-rboh-B     T----------------------------------------------------------- 353
Os-rboh-D     TE---------------------------------------------------------- 370
Os-rboh-H     AE---------------------------------------------------------- 414
Os-rboh-E     TVVADAQT---------------------------------------------------- 361
Os-rboh-F     TIIADGLK---------------------------------------------------- 361
mPp-rboh-A    VGHLTYRS---------------------------------------------------- 363
mPp-rboh-C    SGELTL------------------------------------------------------ 356
mPp-rboh-B    SGELTL------------------------------------------------------ 356
mPp-rboh-D    SGELTL------------------------------------------------------ 365
Ps-rboh-B     ------------------------------------------------------------
Ps-rboh-C     ------------------------------------------------------------
Ps-rboh-A     ------------------------------------------------------------
Hs-Duox1      ------------------------------------------------------------
Hs-Duox2      ------------------------------------------------------------
Lg-Duox1      ------------------------------------------------------------
Lg-Duox2      ------------------------------------------------------------
Lg-Duox3      ------------------------------------------------------------
Dp-Duox       ------------------------------------------------------------
Cb-Duox       ------------------------------------------------------------
Mg-NoxC       ------------------------------------------------------------
Fg-NoxC       ------------------------------------------------------------
Cm-NoxD2      TPKSLATEAMPHAVD--------------------------------------------- 346
Cm-NoxD1      DGLDLAPERICMTT---------------------------------------------- 336
Pt-NoxD       ------------------------------------------------------------
An-Fre1       ------------------------------------------------------------
Sc-Fre1       ------------------------------------------------------------


Dp-Nox5A      STSESPSGAQSVAHTGYRYMRHPPEIINLPVPVEDPRNDDLPHAMWKKFNRKKGPRDTFG 443
Dp-Nox5B      KNSDQLNGAPDDPSSSITFNRAQSPALNR-DPYDEGILRDKQHHRYPHHHQQPRAMSILV 448
Hs-Nox5       ------------------------------------------------------------
Lg-Nox5A      ------------------------------------------------------------
Lg-Nox5B      ------------------------------------------------------------
Dd-NoxC       ------------------------------------------------------------
Mg-NoxA       ------------------------------------------------------------
Fg-NoxA       ------------------------------------------------------------
Cc-NoxA       ------------------------------------------------------------
Lb-NoxA       ------------------------------------------------------------
fPp-NoxA      ------------------------------------------------------------
Pg-NoxA       ------------------------------------------------------------
Bd-NoxA       ------------------------------------------------------------
```

```
Mg-NoxB         ------------------------------------------------------------
Fg-NoxB         ------------------------------------------------------------
Cc-NoxB         ------------------------------------------------------------
Lb-NoxB         ------------------------------------------------------------
fPp-NoxB        ------------------------------------------------------------
Pg-NoxB         ------------------------------------------------------------
Bd-NoxB         ------------------------------------------------------------
Hs-Nox2         ------------------------------------------------------------
Hs-Nox3         ------------------------------------------------------------
Hs-Nox1         ------------------------------------------------------------
Lg-Nox2         ------------------------------------------------------------
Nv-Nox2A        ------------------------------------------------------------
Nv-Nox2B        ------------------------------------------------------------
Ci-Nox2         ------------------------------------------------------------
Sp-Nox2A        ------------------------------------------------------------
Sp-Nox2B        ------------------------------------------------------------
Mb-Nox2         ------------------------------------------------------------
Hs-Nox4         ------------------------------------------------------------
Nv-Nox4         ------------------------------------------------------------
Lg-Nox4         ------------------------------------------------------------
Dd-NoxA         ------------------------------------------------------------
Dd-NoxB         ------------------------------------------------------------
Ng-NoxA         ------------------------------------------------------------
Os-rboh-C       ------------------------------------------------------------
Os-rboh-G       ------------------------------------------------------------
At-rboh-D       ------------------------------------------------------------
Os-rboh-A       ------------------------------------------------------------
Os-rboh-B       ------------------------------------------------------------
Os-rboh-D       ------------------------------------------------------------
Os-rboh-H       ------------------------------------------------------------
Os-rboh-E       ------------------------------------------------------------
Os-rboh-F       ------------------------------------------------------------
mPp-rboh-A      ------------------------------------------------------------
mPp-rboh-C      ------------------------------------------------------------
mPp-rboh-B      ------------------------------------------------------------
mPp-rboh-D      ------------------------------------------------------------
Ps-rboh-B       ------------------------------------------------------------
Ps-rboh-C       ------------------------------------------------------------
Ps-rboh-A       ------------------------------------------------------------
Hs-Duox1        ------------------------------------------------------------
Hs-Duox2        ------------------------------------------------------------
Lg-Duox1        ------------------------------------------------------------
Lg-Duox2        ------------------------------------------------------------
Lg-Duox3        ------------------------------------------------------------
Dp-Duox         ------------------------------------------------------------
Cb-Duox         ------------------------------------------------------------
Mg-NoxC         ------------------------------------------------------------
Fg-NoxC         ------------------------------------------------------------
Cm-NoxD2        ------------------------------------------------------------
Cm-NoxD1        ------------------------------------------------------------
Pt-NoxD         ------------------------------------------------------------
An-Fre1         ------------------------------------------------------------
Sc-Fre1         ------------------------------------------------------------

Dp-Nox5A        PETLRAHKTNINLNYIQLCLSKYFMYKRKKNIIQGESSVRDNRPNGITTTSDAIPDPKQM 503
Dp-Nox5B        PIEETPGKSKTGSEVRKRATPRASYAPESMRKKPAQFTSRDENRSAHQPNSEKP----HS 504
Hs-Nox5         -------GSEILLEKHKFCN---------------------------------------- 331
Lg-Nox5A        -------QLKTTKPMSKIVR---------------------------------------- 358
Lg-Nox5B        -------QSKGTYLFLRKIT---------------------------------------- 358
Dd-NoxC         --------NNNNIDQIDLEIG--------------------------------------- 357
Mg-NoxA         ---------------------------------------------------VDPMGMYEV 367
Fg-NoxA         ---------------------------------------------------VDPMGMYEV 344
Cc-NoxA         ----------------------------------------------D-GPSLSRGDFVEI 353
```

```
Lb-NoxA         ---------------------------------------------------MSRGDYVEL 340
fPp-NoxA        ----------------------------------------------DDSVYGTRGDFVEL 355
Pg-NoxA         ------------------------------------------------FSVEDCGEFHDI 345
Bd-NoxA         ------------------------------------------------------------
Mg-NoxB         ----------------------------------------------------GVNGENPD 333
Fg-NoxB         ----------------------------------------------------GLTGREAE 336
Cc-NoxB         ----------------------------------------------------GAVAKPAA 339
Lb-NoxB         ----------------------------------------------------GGKVVGTN 352
fPp-NoxB        ----------------------------------------------------GGKLIGTN 341
Pg-NoxB         ------------------------------------------------------AGRPTV 332
Bd-NoxB         -----------------------------------------------------------S 298
Hs-Nox2         ------------------------------------------------------------
Hs-Nox3         ------------------------------------------------------------
Hs-Nox1         ------------------------------------------------------------
Lg-Nox2         ------------------------------------------------------------
Nv-Nox2A        ------------------------------------------------------------
Nv-Nox2B        ------------------------------------------------------------
Ci-Nox2         ------------------------------------------------------------
Sp-Nox2A        ------------------------------------------------------------
Sp-Nox2B        ------------------------------------------------------------
Mb-Nox2         ------------------------------------------------------------
Hs-Nox4         ------------------------------------------------------------
Nv-Nox4         ------------------------------------------------------------
Lg-Nox4         ------------------------------------------------------------
Dd-NoxA         -----------------------------------------------------------V 327
Dd-NoxB         -----------------------------------------------------------L 324
Ng-NoxA         -----------------------------------------------------------K 313
Os-rboh-C       ------------------------------------------------------------
Os-rboh-G       ------------------------------------------------------------
At-rboh-D       ------------------------------------------------------------
Os-rboh-A       ------------------------------------------------------------
Os-rboh-B       ------------------------------------------------------------
Os-rboh-D       ------------------------------------------------------------
Os-rboh-H       ------------------------------------------------------------
Os-rboh-E       ------------------------------------------------------------
Os-rboh-F       ------------------------------------------------------------
mPp-rboh-A      ------------------------------------------------------------
mPp-rboh-C      ------------------------------------------------------------
mPp-rboh-B      ------------------------------------------------------------
mPp-rboh-D      ------------------------------------------------------------
Ps-rboh-B       ------------------------------------------------------------
Ps-rboh-C       ------------------------------------------------------------
Ps-rboh-A       ------------------------------------------------------------
Hs-Duox1        ------------------------------------------------------------
Hs-Duox2        ------------------------------------------------------------
Lg-Duox1        ------------------------------------------------------------
Lg-Duox2        ------------------------------------------------------------
Lg-Duox3        ------------------------------------------------------------
Dp-Duox         ------------------------------------------------------------
Cb-Duox         ------------------------------------------------------------
Mg-NoxC         ------------------------------------------------------------
Fg-NoxC         ------------------------------------------------------------
Cm-NoxD2        ---------------------------------------------------------KSS 349
Cm-NoxD1        ------------------------------------------------------------
Pt-NoxD         ------------------------------------------------------------
An-Fre1         ------------------------------------------------------------
Sc-Fre1         ------------------------------------------------------------


Dp-Nox5A        VEGVNIYLPLEITMDGPYGTPSSHIFRAQH-AVLIAAGIGVTPYASILQSIMHRYYASLQ 562
Dp-Nox5B        VEGVHIYYPLEIAIDGPYGTPSSHIFRAQH-AVLVAAGIGVTPFASILQSIMHRYYVSRQ 563
Hs-Nox5         ---------IKCYIDGPYGTPTRRIFASEH-AVLIGAGIGITPFASILQSIMYRHQKRKH 381
Lg-Nox5A        ---------VKIYIDGPFGTSAREIFDTEH-AVLVGSGIGVTPFASILQSILHRFQSATR 408
Lg-Nox5B        ---------AEVYLDGPYGTSTREIFQTEH-AVLIGSGIGITPYASILQSIIYRFNAITR 408
```

```
Dd-NoxC        ------LKPFRINIDGPFGSSSQYALKQKQ-VILVGAGIGVSPMASLLKDISLKKQRLQL 410
Mg-NoxA        ALQN-GQQMPMLRIDGPYGAPAEDVFENEI-AVLIGTGIGVTPWASILKNIWHLRNGPNP 425
Fg-NoxA        ALQN-GDQMPALRIDGPYGAPAEDVFENEI-AVLIGTGIGVTPWAAILKNIWHLRNSPNP 402
Cc-NoxA        DPATMSITLPQVRIDGPYGAPAEDVFNNEV-AVLVGAGIGVTPFASILKHIWYRQKKGKL 412
Lb-NoxA        DPAGSSIALPSVRIDGPYGAPAEDVFGAEV-AILIGAGIGVTPFASILKHIWYRQKKGKL 399
fPp-NoxA       DSG--ARPLPTVRIDGPYGAPAEDVFNVEV-AVLVGAGIGVTPFASILKHIWYRQKKGNL 412
Pg-NoxA        TTIR-SRDLPLVRIDGPYGSPAQDVFKCEV-AILIGAGIGVTPFSSILKNIYYMQAQGKL 403
Bd-NoxA        ------MDLPEIRIDGPYGAPAEDLYNYKV-AVLVGAGIGVTPAASLLKSVWYRYYR-KA 366
Mg-NoxB        VDPALKRVLPRVYVDGPFGSASEDVFKYEI-AVLCGAGIGVTPFASILKSIWYRMNYPQK 392
Fg-NoxB        IDPAIRRVLPRVYVDGPFGSASEDVFKYEV-SVLVGAGIGVTPFASILKSIWYRMNYPQK 395
Cc-NoxB        VPPPINRPLPRVMVDGPFGSASEDFLKYET-VLLVGGGIGVTPFASILKHIWYRMNNLTD 398
Lb-NoxB        VNPSVNRLLPRVMVDGPFGSASEDFLKYET-VLLVGAGIGVTPFASILKSIWYRMNNFNN 411
fPp-NoxB       TNPPVNRVLPRVMVDGPFGSASEDFLNYET-VLLVGAGIGVTPFASILKSIWYRMNNLNN 400
Pg-NoxB        LPPATNRVLPRVMVDGPFGSASEDVFKFEV-VMLVGGGIGVTPFASVLKSIWYKLNFPSA 391
Bd-NoxB        DNMPAPDTLPYVMVDGPYGSASEDVFDYEA-AVLVGAGIGVTPFASILKTIWFRINNPTR 357
Hs-Nox2        --FQDAWKLPKIAVDGPFGTASEDVFSYEV-VMLVGAGIGVTPFASILKSVWYKYCN--N 430
Hs-Nox3        --LQEPWSLPRLAVDGPFGTALTDVFHYPV-CVCVAAGIGVTPFAALLKSIWYKCSE--A 428
Hs-Nox1        -------PIPRIEVDGPFGTASEDVFQYEV-AVLVGAGIGVTPFASILKSIWYKFQC--A 424
Lg-Nox2        --FQEAWKMPKIAIDGPYGTSTEDCFRFDV-AVLIGTGIGVTPFASVLRHVWKKYST--R 423
Nv-Nox2A       --LQSVNEMPRLALDGPFGTASIDVFKYGV-GMYIGAGIGVTPFASVLKSIWYRYNE--N 422
Nv-Nox2B       --QIAIDQMPRIAVDGPYGTASTDVFRYEV-VMCIGAGIGVTPFASILKSIWYRHNQ--D 416
Ci-Nox2        --VQPSWKMPRLAIDGPFGTASEDVFNYPV-AICVGSGIGVTPFASLLKSVWYKNLN--P 443
Sp-Nox2A       --QQSQDELARVAVDGPFGTASIDIFKYQV-AICVGAGIGVTPFASILKSIWLKSVN--N 441
Sp-Nox2B       --PLSITQLPRVQVDGPFGTSCTDIFDYDV-VMCVSAGIGVTPYASTLKSIWISSRQ--N 415
Mb-Nox2        --PKVGSELPYICIDGPFGTASEDMYHYPV-AMLIGAGIGVTPFASLLKELYFRKSNPSA 464
Hs-Nox4        LPFIQSRNYPKLYIDGPFGSPFEESLNYEV-SLCVAGGIGVTPFASILN----TLLDDWK 450
Nv-Nox4        LVAAPSVRSAVLSVDGPYGSPCMDVEEYRV-SMCIATGIGVTPFAALITRIRSQIIHAQR 444
Lg-Nox4        LHYEDIVRSPELKVDGPYSSPLCEMNQSKL-AVCIAGGIGVTPFISFLLKLR------YK 372
Dd-NoxA        LKSPD-GKP-ILRIDGPFGAASEEVFKYKQ-VILVGAGIGVTPFASILKHIKYQMARTYN 384
Dd-NoxB        KSTQNRGKRRILKIDGPFGAPAENFFKYRN-LVLIGAGIGVTPFSSILRHLKNQNDKQTN 383
Ng-NoxA        PTTPD-GTDYLIRVDGPFGTCAEYCFDFEY-VMLIAAGIGVTPYSSLLKHFKFRLDAAAS 371
Os-rboh-C      ----EKARFPKLLVDGPYGAPAQDYREYDV-LLLIGLGIGATPLISIVKDVLN---HIQG 410
Os-rboh-G      ----HHSRFPKLLIDGPYGAPAQDYWKYDV-LLLIGLGIGATPLISIVKDVLN---HIYD 370
At-rboh-D      ----GNLPFPKVLIDGPYGAPAQDYKKYDV-VLLVGLGIGATPMISILKDIIN---NMKG 403
Os-rboh-A      -----KKILPKLLIDGPYGSPAQDYSKYDV-LLLVGLGIGATPFISILKDLLNNIIKMEE 407
Os-rboh-B      -----KKALPKLLIDGPYGSPAQDYSKYDV-LLLVGLGIGATPFISILKDLINSIIKMEE 407
Os-rboh-D      -----PRSLPRLLVDGPYGAPAQDFRNYDV-LLLVGLGIGATPFISILRDLLNN-IKLAE 423
Os-rboh-H      -----QKSPPRLLVDGPYGAPAQDFRNYDV-LLLVGLGIGATPFISILRDLLNN-IKLAD 467
Os-rboh-E      ----EDTRFPKVLIDGPYGAPAQNYKKYDI-LLLIGLGIGATPFISILKDLLN---NIKS 413
Os-rboh-F      ----EETCFPKVFIDGPFGAPAQNYKKYDI-LLLIGLGIGATPFISILKDLLN---NIKS 413
mPp-rboh-A     ----CCHRFPKLYIDGPYGAPAQDYLKYDV-LLLVGLGIGATPFISILKDMLH---HTRN 415
mPp-rboh-C     -----AARFPKLYIDGPYGAPAQDYLNYDV-LLLVGLGIGATPFISILKDLLH---HTIN 407
mPp-rboh-B     -----AARFPKLFVDGPYGAPAQDYLKYDV-LLLVGLGIGATPFISILKDMLH---HTR- 406
mPp-rboh-D     -----APRFPKLYIDGPYGAPAQDYLKYDV-LLLVGLGIGATPFISILKDMLH---HSRN 416
Ps-rboh-B      --------YPDIHLDGPVGAPTQDYHRYKT-VICVGGGIGVTPFASILKDVVHLWEANRC 381
Ps-rboh-C      --------YPDVYLDGPVGAPTQDYHRYKT-VICIGGGIGVTPFASILKDVVHLWEDNRC 368
Ps-rboh-A      -------PYPTVFLDGPIGAPAQDYSRYRE-VVLIGAGIGVTPFASILRSIMHQWESYRC 384
Hs-Duox1       ------ARYPKLYLDGPFGEGHQEWHKFEV-SVLVGGGIGVTPFASILKDLVFKSSVS-- 373
Hs-Duox2       ------AGYPKLYLDGPFGEGHQEWHKFEV-SVLVGGGIGVTPFASILKDLVFKSSLG-- 373
Lg-Duox1       ------KGFPKLYLDGPFGEGHQDWYRYPV-AVLVGGGIGVTPFASILKDLVQKSKMK-- 381
Lg-Duox2       ------KPLPLLYVDGPFGEGHQDWFTFDV-AVLVGGGIGVTPFASILKDIAFKSKAG-- 380
Lg-Duox3       ------NPYPKLFLDGPYGAGQQDWYQYEV-SLLVGAGIGVTPYASILKDFVHMTSIKNT 382
Dp-Duox        ------DMDPKIQLDGPFGGGNQDWYKFEV-AVMVGGGIGVTPYASILNDLVFGTSTNRY 383
Cb-Duox        ------SPYPLIHLKGPYGDGNQEWMNYEV-AIMVGGGIGVTPYASTLNDLVQLTASDSF 384
Mg-NoxC        -----QEVDIEIGINGPFGAPAQRFYDFNH-TILVGAGIGLTPFSGILADLQAKEDRLHG 373
Fg-NoxC        -----GESEIEVGINGPFGAPAQRFYDFNH-SIIIGAGIGVTPFSGILADLQYNDDLDHG 370
Cm-NoxD2       ITTCLPINRRWILLSGPYGAPAQSHQNFAH-QLLIGTGVGASPMFSIVQELCG-RATPCD 407
Cm-NoxD1       ------IEAYRVLLAGPYGAPAQSHLHFPY-QLLIGSGVGATPMVSILREICSARAPAKL 389
Pt-NoxD        -----APLSMTVNLRGPFGAPAQNYLNYRH-LVVIGSGIGVTPLLSVWAYLVRATKGLVK 381
An-Fre1        ------TIQPLLLLEGPYGHTAP-LHTFDT-VLLIAGGTGIAATLPYILDYAARLDSQAT 619
Sc-Fre1        ---------CKIFLEGPYGVTVPHIAKLKRNLVGVAAGLGVAAIYPHFVECLRLPSTDQL 558
                              **                    * *

Dp-Nox5A       KCPNCRYSWANQ------------------------------------------------ 574
```

```
Dp-Nox5B       SCPRCHHSWVSQ------------------------------------------------ 575
Hs-Nox5        TCPSCQHSWIEG------------------------------------------------ 393
Lg-Nox5A       TCPKCQHSFYD------------------------------------------------- 419
Lg-Nox5B       TCPKCHHCFNEP------------------------------------------------ 420
Dd-NoxC        LNQGDQIALEQSKN---------------------------------------------- 424
Mg-NoxA        PTR--------------------------------------------------------- 428
Fg-NoxA        PRR--------------------------------------------------------- 405
Cc-NoxA        GS---------------------------------------------------------- 414
Lb-NoxA        GS---------------------------------------------------------- 401
fPp-NoxA       QS---------------------------------------------------------- 414
Pg-NoxA        GL---------------------------------------------------------- 405
Bd-NoxA        SMP--------------------------------------------------------- 369
Mg-NoxB        K----------------------------------------------------------- 393
Fg-NoxB        K----------------------------------------------------------- 396
Cc-NoxB        AK---------------------------------------------------------- 400
Lb-NoxB        SK---------------------------------------------------------- 413
fPp-NoxB       SK---------------------------------------------------------- 402
Pg-NoxB        SKQGS------------------------------------------------------- 396
Bd-NoxB        A----------------------------------------------------------- 358
Hs-Nox2        ATN--------------------------------------------------------- 433
Hs-Nox3        QTP--------------------------------------------------------- 431
Hs-Nox1        DHN--------------------------------------------------------- 427
Lg-Nox2        RNE--------------------------------------------------------- 426
Nv-Nox2A       PSD--------------------------------------------------------- 425
Nv-Nox2B       LEN--------------------------------------------------------- 419
Ci-Nox2        EHE--------------------------------------------------------- 446
Sp-Nox2A       SAS--------------------------------------------------------- 444
Sp-Nox2B       FCT--------------------------------------------------------- 418
Mb-Nox2        YPS--------------------------------------------------------- 467
Hs-Nox4        PYK--------------------------------------------------------- 453
Nv-Nox4        PPR--------------------------------------------------------- 447
Lg-Nox4        VGK--------------------------------------------------------- 375
Dd-NoxA        TTP--------------------------------------------------------- 387
Dd-NoxB        ADEN-------------------------------------------------------- 387
Ng-NoxA        GQAP-------------------------------------------------------- 375
Os-rboh-C      E----GSVGTTEPE---------------------------------------------- 420
Os-rboh-G      DPESAASPHTTNGG---------------------------------------------- 384
At-rboh-D      P-----DRDSDIEN---------------------------------------------- 412
Os-rboh-A      EEDASTDLYPPMGR---------------------------------------------- 421
Os-rboh-B      EEEASGDLYPPIGR---------------------------------------------- 421
Os-rboh-D      ELMDLAMETSRSED---------------------------------------------- 437
Os-rboh-H      ELMDLAMETSRSDD---------------------------------------------- 481
Os-rboh-E      N----EEVESIHGS---------------------------------------------- 423
Os-rboh-F      N----GDVQSTHDA---------------------------------------------- 423
mPp-rboh-A     DSSGHSYPVLT------------------------------------------------- 426
mPp-rboh-C     ESLGHSDPSLTPDL---------------------------------------------- 421
mPp-rboh-B     ---YDLS----------------------------------------------------- 410
mPp-rboh-D     DSVSDLSSSPDLN----------------------------------------------- 429
Ps-rboh-B      PNCS-------------------------------------------------------- 385
Ps-rboh-C      LNCN-------------------------------------------------------- 372
Ps-rboh-A      PQCG-------------------------------------------------------- 388
Hs-Duox1       CQVFC------------------------------------------------------- 378
Hs-Duox2       SQMLC------------------------------------------------------- 378
Lg-Duox1       VKFPC------------------------------------------------------- 386
Lg-Duox2       IKINC------------------------------------------------------- 385
Lg-Duox3       YKVKC------------------------------------------------------- 387
Dp-Duox        SGVAC------------------------------------------------------- 388
Cb-Duox        HKVRC------------------------------------------------------- 389
Mg-NoxC        GPTQKLQEQAEKGGDVRGSTSADAPDGRRIGTSREAEAMPQRTDVDMQAPTSGDQSNTTL 433
Fg-NoxC        GPNHEVDHHR------HDSEATAIPQAARRSDSSSSDEATTSDNVPETPTRQGSVGPDLI 424
Cm-NoxD2       N----------------------------------------------------------- 408
Cm-NoxD1       N----------------------------------------------------------- 390
Pt-NoxD        KVYFVFYG---------------------------------------------------- 389
An-Fre1        KTT--------------------------------------------------------- 622
```

```
Sc-Fre1         QHK--------------------------------------------------------- 561

Dp-Nox5A        --------------------MPDSIMNLKKVDFFWINRDQRSFEWFVHVLSQLEMEQAEM 614
Dp-Nox5B        --------------------MPDSIMNLKKVDFFWINREQRSFEWFVEVLSQLEVEQAEM 615
Hs-Nox5         --------------------VQDN-MKLHKVDFIWINRDQRSFEWFVSLLTKLEMDQAEE 432
Lg-Nox5A        --------------------QRGPGMKIKRVDFIWINRDQKNFEWFVRMLTDIEVEQA-N 458
Lg-Nox5B        --------------------LAGSMMKLKKVDFVWINRDQRAFEWFTSLLTQLELEQVKN 460
Dd-NoxC         -----EIT------------TKFGLGNLEKVHFFWLNRDQHSFQWFEDLLIDISTNGN-- 465
Mg-NoxA         ---------------------------LRRVEFLWVCKDTSSFEWFQTLLSSLEQQSTDA 461
Fg-NoxA         ---------------------------LRRVEFIWVCKDTGSFEWFQTLLSSLEEQSNEA 438
Cc-NoxA         ---------------------------LRRVEFFWVCRDAPSFGWFQSLLQEVEAAQAD- 446
Lb-NoxA         ---------------------------LRRVEFFWVCRDAPSFGWFQSLLQEVEAAQADR 434
fPp-NoxA        ---------------------------LKRVEFFWVCRDAPSFGWFQTLLQEVEEAQVD- 446
Pg-NoxA         ---------------------------LRKVQFIWINKEITSFSWFKTLLKNLEDIQHD- 437
Bd-NoxA         ---------------------------LKKVYFVWINRDKEAFGWFQSLLASLEETIPR- 401
Mg-NoxB         -------------------------TRLAKVYFFWICRDFGSFEWFRSLLLAIE----AQ 424
Fg-NoxB         -------------------------TRLSKVYFFWICRDFDSFEWFRSLLLAVE----AQ 427
Cc-NoxB         ------------------------PTRLSKVYFTWVIKDFGTAEWFHSLLHAIE----EE 432
Lb-NoxB         ------------------------PTRLSKVYFTWVIRDFGVAEWFHSLLHAIE----EQ 445
fPp-NoxB        ------------------------PTRLSKVYFTWVIRDFGSAEWFHSLLQAIE----EQ 434
Pg-NoxB         ------------------------PIRLQKVYFFWVCRDFDSFEWFKSLLSAIE----EQ 428
Bd-NoxB         -------------------------VPLKKVYFFWICRDKDAFEWFQDLLSTIE----DE 389
Hs-Nox2         -------------------------LKLKKIYFYWLCRDTHAFEWFADLLQLLESQMQER 468
Hs-Nox3         -------------------------LKLSKVYFYWICRDARAFEWFADLLLSLETRMSEQ 466
Hs-Nox1         -------------------------LKTKKIYFYWICRETGAFSWFNNLLTSLEQEMEEL 462
Lg-Nox2         -------------------------LQLKYVHFYWVCPSTSSFEWLQELLNSLESQMAEM 461
Nv-Nox2A        -------------------------LNIKKVYFFWICNDTNAFEWFTDLLKILEEKMIDS 460
Nv-Nox2B        -------------------------LRVKKVYFYWICRDTFAFEWFSDLLKHIEIQMDEM 454
Ci-Nox2         -------------------------MVLKKVYFFWICPETHAFEWFGDLLKYLERQLTEI 481
Sp-Nox2A        -------------------------LKLKKVYFFWICPDTNAFEWFSTLLDSIDTHFTEQ 479
Sp-Nox2B        -------------------------LHLKRMYFYWICRDTHAFEWFVELLSSLELILRQI 453
Mb-Nox2         -------------------------FKTQKVYFYWMCPGFDAWGWFASLLIDLEDKLEQL 502
Hs-Nox4         ---------------------------LRRLYFIWVCRDIQSFRWFADLLCMLHNKFWQE 486
Nv-Nox4         ---------------------------PHRLYFVWICREVGALQWFADLIHETSRQLWEL 480
Lg-Nox4         ---------------------------LKKLYFIWIVRDLSHLAWFMEDILLSH-QLLER 407
Dd-NoxA         --------------------------LIDKVHFYWICRDRNSFEWFSGLIGELEMEN-H- 419
Dd-NoxB         ------------------------HLKINKIYFIWISRQKNSFQWFTDILAELENDERI- 422
Ng-NoxA         ------------------------PLKIRRASFYWINRDEGSWEWFSDILNQLETEN--- 408
Os-rboh-C       -----SS-----------SKAKKKPFMTKRAYFYWVTREEGSFEWFRGVMNEVSEKDK-- 462
Os-rboh-G       -----GA-----------AAAARRAFMTKRVYFYWCTREEGSFEWFRGVMNEVADRDAG- 427
At-rboh-D       -----NN-----------SNNNSKGFKTRKAYFYWVTREQGSFEWFKGIMDEISELDE-- 454
Os-rboh-A       -----NKPHVDLGTLMTITSRPKKILKTTNAYFYWVTREQGSFDWFKGVMNEIADLDQ-- 474
Os-rboh-B       -----NKAHVDLDTLMRITSKPKRVLKTTNAYFYWVTREQGSFDWFKGVMNEIAELDQ-- 474
Os-rboh-D       -----SAN---SFSVSTASSNKKRAYRTSRAHFYWVTREPLSFEWFKGVMNEVAEMDK-- 487
Os-rboh-H       -----SAN---SFSVSTASSNKRRAYRTSRAHFYWVTREPGSFEWFKGVMNEVAEMDK-- 531
Os-rboh-E       -----EIG-------------SFKNNGPGRAYFYWVTREQGSFEWFKGVMNDVAESDH-- 463
Os-rboh-F       -----ELGC------------TFKSNGPGRAYFYWVTREQGSFEWFKGVMNDVAESDH-- 464
mPp-rboh-A      ----------------------TKAKRKPKAYFYWITREQGSFDWFRGVMREVEEIDN-- 462
mPp-rboh-C      -----RPM------ESPRTRSKKKAKRNPKAYFYWMTREQGSFDWFRGVMREVEEIDH-- 468
mPp-rboh-B      ------------------DPVMKPCDRES-SYFYWVTREQGSFDWFRGIMREVEEIDN-- 449
mPp-rboh-D      --------------PTGSPPPKKKAKRDPKAYFYWVTREQGSFDWFRGIMREVEEIDN-- 473
Ps-rboh-B       ------------------HVRHPGSFKIQKLYFHWVTRGQESLSWFEETMNQIAEMDR-- 425
Ps-rboh-C       ------------------HVRHPSSFQIQKLYFHWVTRGQESLSWFEGTMKQIAQMDR-- 412
Ps-rboh-A       ------------------HVRFPPSFQLRKIYFYWVMREQEALTWFTNTMNQLSQMDA-- 428
Hs-Duox1        ----------------------------KKIYFIWVTRTQRQFEWLADIIREVEENDHQ- 409
Hs-Duox2        ----------------------------KKIYFIWVTRTQRQFEWLADIIQEVEENDHQ- 409
Lg-Duox1        ----------------------------QKVYFLWVTRTQKSFEWMTDIIREVELGDVN- 417
Lg-Duox2        ----------------------------KKVYFVWITRSQKHFEWLIDIIRDVEEQDVL- 416
Lg-Duox3        ----------------------------QKLYFIWVTGSQRHFEWLIDILREVEEIDER- 418
Dp-Duox         ----------------------------KKVYFLWTCPSHRHFEWFIDVLRDIESKDVT- 419
Cb-Duox         ----------------------------QKVYFLWVCPSHKNYEWFVDVLKNVENQDRR- 420
Mg-NoxC         QETADADSDSASSISRPSSSFSSFASDYRRVDFHWMVRDRNHLLWISELLNTVSRSQAWH 493
Fg-NoxC         NKEKQPQADKAG----------SFAEDYRRVDFHWMVRERNYLLWLSDLLNDVSMSQDWH 474
```

```
Cm-NoxD2        ---------------------------IRTVTFVWVVRYVEDLWFLEKLYELASQQDASD 441
Cm-NoxD1        ---------------------------VFSLTFVWVVREYADLWGIQGLFDLA--QTWTP 421
Pt-NoxD         -----------------------AFIALHSVRIFHIFYATARPPHTEEPTRGVDAIHSVT 426
An-Fre1         -----------------------------RLHLIWSARQRDVFTTVLSDELSHVLQCEG- 652
Sc-Fre1         --------------------------------FYWIVNDLSHLKWFENELQWLKEKSCEV 589


Dp-Nox5A        DSALG----RFLDLHMYITSALKKTDLKAVGLQMALDLLYAE------------------ 652
Dp-Nox5B        GSALG----RFLDLHMYITSALKKTDLKAVGLQMALDLIHAK------------------ 653
Hs-Nox5         A-QYG----RFLELHMYMTSALGKNDMKAIGLQMALDLLANK------------------ 469
Lg-Nox5A        YDKFG----RIIHMHMYMTSALSKTDMKGLGLQMALELLHTK------------------ 496
Lg-Nox5B        GSHLD----KILNLHMYMTAAVQKADMKGIGLQVALDLIHEK------------------ 498
Dd-NoxC         --SNL----PKISINTFNTRVFPKNDVRVFMLWNGLDKLFKA------------------ 501
Mg-NoxA         AGLPGGNGVEFLKIHSYLTQKLDMDTTQNIVLNSVGAAL--------------------- 500
Fg-NoxA         ARMPGSTGVEFLKIHTYLTQKLDIDTAQNIVLNSVGSQM--------------------- 477
Cc-NoxA         --------PNFLRINIYLTQKINEDMLWNIAVNDAGADY--------------------- 477
Lb-NoxA         E--LHVLAANFLRINVYLTQKISEDMLWNIAVNDAGAEY--------------------- 471
fPp-NoxA        --------PNFLRINIYLTQKIGEDMLWNIALNDAGAEY--------------------- 477
Pg-NoxA         --------YSFLRMDMYLTGSMDEDTISNVVLNTGAHKF--------------------- 468
Bd-NoxA         ---------SFLEIHTYLTGNLAVDDIQNILLN-SDMDV--------------------- 430
Mg-NoxB         DVD------NRIEIHTYLTAKIKADDATNIMINDANAD---------------------- 456
Fg-NoxB         DLD------HRIEIHTYLTARIKADDATNIMINDANAD---------------------- 459
Cc-NoxB         DTQ------NRIEINIYLTAKLKEDEVNNIIVQDVGAE---------------------- 464
Lb-NoxB         DTQ------GRIEINIYLTAKIKEDDMNNIILQDVGAE---------------------- 477
fPp-NoxB        DTQ------NRIEINIYLTAKIKEDDMNNIIVQDVGAE---------------------- 466
Pg-NoxB         DVD------RRVELHTYITQKLKDDDINNIIVSDVGGN---------------------- 460
Bd-NoxB         NIS------NFLEIHTYLTQKLKISEVKNIVINDGEDG---------------------- 421
Hs-Nox2         NNA------GFLSYNIYLT-GWDESQANHFAVHHDEE----------------------- 498
Hs-Nox3         GKT------HFLSYHIFLT-GWDENQALHIALHWDEN----------------------- 496
Hs-Nox1         GKV------GFLNYRLFLT-GWDSNIVGHAALNFDKA----------------------- 492
Lg-Nox2         GSA------NFLSHNIYLTSGWDSNQAKNIVLHNDEE----------------------- 492
Nv-Nox2A        GNA------GFLEYNIYLTRGWGANMARDIYLREDEV----------------------- 491
Nv-Nox2B        NMP------GFIQINIFLT-GWDKKLANQVVMERSAD----------------------- 484
Ci-Nox2         GRQ------DLIEYHIYLTRGWDHKQAKAIYAHEEDT----------------------- 512
Sp-Nox2A        GKP------DFLKYYIYLSRGWNNTQAKNIYLQEEQE----------------------- 510
Sp-Nox2B        DKE------HLLSYSIYLTRGWDYTQAKNIFMQEDRE----------------------- 484
Mb-Nox2         GVP------DFLEIRVFTTRGWSQDDAAKIMLQEDESGDS--------------------- 536
Hs-Nox4         NRP------DYVNIQLYLSQTD-GIQKIIG--EKYHALN--------------------- 516
Nv-Nox4         NRP------DFLTCLFYITSKG-QKEKICINHTQSSWFD--------------------- 512
Lg-Nox4         KFP------NSLEVQFYVTNSDSTTEQRHFSEDSLNFIQ--------------------- 440
Dd-NoxA         --------NNFLEIHPYLTGALSAQEIRDVMYGD-------------------------- 445
Dd-NoxB         --------DSILEIHIFLTGALELDDYAKIKN---------------------------- 446
Ng-NoxA         --------PEFFDIHTYMTGMLKADDVKKIMFTSSEYQTQSNTAGNVQQSGKVDFMVRAL 460
Os-rboh-C       --------DGVIELHNHCSSVYQEGDARSALIVMLQELQHAKK----------------- 497
Os-rboh-G       --------RELIELHNHCTSVYEEGDARSALVTMLQALHHAKN----------------- 462
At-rboh-D       --------EGIIELHNYCTSVYEEGDARVALIAMLQSLQHAKN----------------- 489
Os-rboh-A       --------RNIIEMHNYLTSVYEEGDARSALITMLQALNHAKN----------------- 509
Os-rboh-B       --------RNIIEMHNYLTSVYEEGDARSALITMLQALNHAKN----------------- 509
Os-rboh-D       --------KGVIELHNYLTSVYEERDARTTLLSMVQALNHAKH----------------- 522
Os-rboh-H       --------KGVIELHNYLTSVYEERDARSTLLSMVQALNHAKH----------------- 566
Os-rboh-E       --------NNIIEMHNYLTSVYEEGDARSALIAMVQSLQHAKN----------------- 498
Os-rboh-F       --------DNVIEMHNYLTSVYEEGDARSALIAMVQSLQHAKN----------------- 499
mPp-rboh-A      --------KRSIEMHNYLTSVYEEGDARSTLVMMLQALHHAKN----------------- 497
mPp-rboh-C      --------KGSIEMHNYLTSVYEEGDARSTLVIMLQALHHAKN----------------- 503
mPp-rboh-B      --------KESIEMHNYLTSVYEEGDARSTLVTMLQSLYHAKN----------------- 484
mPp-rboh-D      --------KELIEMHNYLTSVYEEGDARSTLVTMLQSLHHAKN----------------- 508
Ps-rboh-B       --------DNVIETHQYLST--LKGSENTSQLKMFQEFVHEQT----------------- 458
Ps-rboh-C       --------DNVIETHQYLSTVVLGGEENTSQLKMFQEFVHEQT----------------- 447
Ps-rboh-A       --------DNRLEIHNFFSS--VKNEEVIAPLQALQNFIHDTE----------------- 461
Hs-Duox1        ---------DLVSVHIYITQLAEKFDLRTTMLYICERHFQKVLN---------------- 444
Hs-Duox2        ---------DLVSVHIYVTQLAEKFDLRTTMLYICERHFQKVLN---------------- 444
Lg-Duox1        ---------NLVSVHIFITQFQQKFDLRTTMLYICERHFQKVAG---------------- 452
Lg-Duox2        ---------EIVNVHIFITQFTQKYDLRTTMLYICERNFQKVEN---------------- 451
Lg-Duox3        ---------GMVSIDIFITQFFQNFDLRTAMLYVFEEHFQKMTGG--------------- 454
```

```
Dp-Duox         ---------NVLEMHIFITQFFHKFDLRTTMLYICENHFQRISK---------------- 454
Cb-Duox         ---------GILETHIFVTQLFHKFDLRTTMLYICEKHFRATNAG--------------- 456
Mg-NoxC         HRHDAP-GEYHLDIRMQTHVTQKRKNVSTHVYRWLLEQHRTPEHP--------------- 537
Fg-NoxC         REHED---KPHLDIRINTHVTAKQKKISTHVYRWLLEMHRTDEHP--------------- 516
Cm-NoxD2        PCR------PRLRIHVHITRLGHDDADPVSSRLPANCGEILQFHCGR------------- 482
Cm-NoxD1        DLI------PRVRVHIFLTR-GEIPASILEAGQSTKS--ILQFHQGR------------- 459
Pt-NoxD         GIWVAKKYEDMSFAAPSLVRTLPGLSKVFSLRLFATRDKEED------------------ 468
An-Fre1         -----------VTISFFCTGGDPMSPIEIEAIDKEVAIN--------------------- 680
Sc-Fre1         S-----------VIYTGSSVEDTNSDESTKGFDDKEESE--------------------- 617


Dp-Nox5A        ------------------------------------------------EKRDLVTG--LK 662
Dp-Nox5B        ------------------------------------------------DKRDLVTG--LK 663
Hs-Nox5         ------------------------------------------------EKKDSITG--LQ 479
Lg-Nox5A        ------------------------------------------------EHRDVLTG--LR 506
Lg-Nox5B        ------------------------------------------------EQRDLLTG--LR 508
Dd-NoxC         ------------------------------------------------QGLDPTTN--LP 511
Mg-NoxA         ---------------------------------------------------DPLTE--LK 507
Fg-NoxA         ---------------------------------------------------DPLTE--LQ 484
Cc-NoxA         ---------------------------------------------------DPLTL--LR 484
Lb-NoxA         ---------------------------------------------------DPLTL--LR 478
fPp-NoxA        ---------------------------------------------------DPLTL--LR 484
Pg-NoxA         ---------------------------------------------------DSLTG--LK 475
Bd-NoxA         ---------------------------------------------------DPLTE--LQ 437
Mg-NoxB         --------------------------------------------------KDAITG--LR 464
Fg-NoxB         --------------------------------------------------KDTITG--LR 467
Cc-NoxB         --------------------------------------------------KDAITS--LR 472
Lb-NoxB         --------------------------------------------------KDAITS--LR 485
fPp-NoxB        --------------------------------------------------KDAITS--LR 474
Pg-NoxB         --------------------------------------------------RDAITQ--LR 468
Bd-NoxB         --------------------------------------------------RDAITG--LK 429
Hs-Nox2         --------------------------------------------------KDVITG--LK 506
Hs-Nox3         --------------------------------------------------TDVITG--LK 504
Hs-Nox1         --------------------------------------------------TDIVTG--LK 500
Lg-Nox2         --------------------------------------------------RDAVTG--LQ 500
Nv-Nox2A        --------------------------------------------------EDPITR--LR 499
Nv-Nox2B        --------------------------------------------------RDPITG--LF 492
Ci-Nox2         --------------------------------------------------HDVITG--LE 520
Sp-Nox2A        --------------------------------------------------IDAITG--LR 518
Sp-Nox2B        --------------------------------------------------IDAVTG--LR 492
Mb-Nox2         ------------------------------------------------IVRDAETGRALR 548
Hs-Nox4         ------------------------------------------------------------
Nv-Nox4         ------------------------------------------------------------
Lg-Nox4         ------------------------------------------------------------
Dd-NoxA         ------------------------------------------------EEKDLITGFT-- 455
Dd-NoxB         ------------------------------------------------AQKCHITNLH-- 456
Ng-NoxA         HKYDAQSSDEISLDRNDIVVVSEQDESGWWTGTNTTTKQTGLFPGNFVVKVDHVTKMADS 520
Os-rboh-C       -------------------------------------------------GVDILS--GTS 506
Os-rboh-G       -------------------------------------------------GVDVVS--GTR 471
At-rboh-D       -------------------------------------------------GVDVVS--GTR 498
Os-rboh-A       -------------------------------------------------GVDIVS--GTK 518
Os-rboh-B       -------------------------------------------------GVDIVS--GTK 518
Os-rboh-D       -------------------------------------------------GVDIVS--GTR 531
Os-rboh-H       -------------------------------------------------GVDIVS--GTR 575
Os-rboh-E       -------------------------------------------------GVDIVS--GSR 507
Os-rboh-F       -------------------------------------------------GVDIVS--GSK 508
mPp-rboh-A      -------------------------------------------------GVDLVS--GTR 506
mPp-rboh-C      -------------------------------------------------GVDLVS--GTR 512
mPp-rboh-B      -------------------------------------------------GVDLVS--GTR 493
mPp-rboh-D      -------------------------------------------------GVDVVS--GTR 517
Ps-rboh-B       -------------------------------------------------GKDFVSGLSTK 469
Ps-rboh-C       -------------------------------------------------GKDFVSGMDTK 458
Ps-rboh-A       -------------------------------------------------GQDIISGLQTR 472
Hs-Duox1        --------------------------------------------------RSLFTG--LR 452
```

```
Hs-Duox2        --------------------------------------------------RSLFTG--LR 452
Lg-Duox1        --------------------------------------------------QSLFTG--LR 460
Lg-Duox2        --------------------------------------------------KSLFTG--LR 459
Lg-Duox3        --------------------------------------------------KSVYTG--LK 462
Dp-Duox         --------------------------------------------------RSMFTG--LK 462
Cb-Duox         --------------------------------------------------MSMFTG--LH 464
Mg-NoxC         --------------------------------------------------ASPITG--LI 545
Fg-NoxC         --------------------------------------------------ASPLTG--LL 524
Cm-NoxD2        ------------------------------------PDFGEYVAELVNEDHDTTTP--LA 504
Cm-NoxD1        ------------------------------------PRFETFIEDLL----DLRDP--YE 477
Pt-NoxD         -----------------------------------------------LVHRNPLADHGPD 481
An-Fre1         ----------------------------------------------------TTSN---- 684
Sc-Fre1         ----------------------------------------------------------IT 619


Dp-Nox5A        TRTNAGRPNWDK-VFQKLIDEDKGN--------------------------VTVFYCGPP 695
Dp-Nox5B        TRTNAGRPNWDK-IFQKLVDEDKGK--------------------------VTVFYCGPP 696
Hs-Nox5         TRTQPGRPDWSK-VFQKVAAEKKGK--------------------------VQVFFCGSP 512
Lg-Nox5A        TRTHAGRPNFDE-LFGEIKQNKAGK--------------------------VKVFLCGPQ 539
Lg-Nox5B        TRTKAGRPDFNE-LFKSIAAQKHGK--------------------------VKVFFCGAP 541
Dd-NoxC         FKTHWGRPNWDT-IFQYYSKKYSGES-------------------------ISVFCCGPS 545
Mg-NoxA         SRTNFGRPNFAK-LFASMRDGIMDRT-------------YLSGLEGSMKTTVGVYFCGPS 553
Fg-NoxA         SRTNFGRPDFPR-LFTTMRNGILDRT-------------YLNGLESHIRTTVGVYFCGPS 530
Cc-NoxA         SRTMFGRPDWNS-IYSRIRQAVEGGQ-------------YIPGATAQLKTKVGTYFCGPS 530
Lb-NoxA         SRTMFGRPDWMT-IFGQMKQAIEGGQ-------------YLPGSTSQLKTKVATYFCGPG 524
fPp-NoxA        TRTMFGRPDWKN-IYARMKQAIEMGQ-------------YLPGTKEQLKTKVGTYFCGPG 530
Pg-NoxA         SQTHFGKPHWKKDVFDPIRKAIHSGD-------------WYERDISG-TTKVGCFYCGPR 521
Bd-NoxA         SRTHYGRPAWSQ-LLNGIKLNVAD--------------------VRDPQVEVGIFYCGPG 476
Mg-NoxB         APTNFGRPNWDM-IFRGIRKLHT-------------------------PAEAGVFFCGPK 498
Fg-NoxB         SPTNFGRPNWDM-IFRGIRKIHS-------------------------PAEAGVFYGGPK 501
Cc-NoxB         APTHFGRPNWDR-VFGSLCEKHP-------------------------ETDVGVFFCGPA 506
Lb-NoxB         APTHFGRPNWDR-VFGSIAEKHP-------------------------ETDVGVFFCGPA 519
fPp-NoxB        APTHYGRPNWDR-VFSSIAEKHP-------------------------ETDVGVFFCGPA 508
Pg-NoxB         SPTHYGRPNWDR-IFNSVRETHP-------------------------ATDVGVFFCGPG 502
Bd-NoxB         SRTQYGRPNWDQ-IFEALRVKHR-------------------------ATDIGVFFCGPK 463
Hs-Nox2         QKTLYGRPNWDN-EFKTIASQHP-------------------------NTRIGVFLCGPE 540
Hs-Nox3         QKTFYGRPNWNN-EFKQIAYNHP-------------------------SSSIGVFFCGPK 538
Hs-Nox1         QKTSFGRPMWDN-EFSTIATSHP-------------------------KSVVGVFLCGPR 534
Lg-Nox2         HKTHYGRPQWDK-IFSDLAVFHK-------------------------GMKLGVFFCGPK 534
Nv-Nox2A        QKTRFGRPEWNK-IFNDIGNKHP-------------------------KTDIGVFFCGPK 533
Nv-Nox2B        ARTKYGRPEWAK-IFNEVAEAHN-------------------------QTSVGVFFCGPS 526
Ci-Nox2         QKTNYGRPNWDE-IFSKTARDYP-------------------------NTHIGVFFCGVA 554
Sp-Nox2A        QKTHYGRPKWDS-NFKMIAEENPG------------------RVS-------SVFFCGPK 552
Sp-Nox2B        QKTHYGRPKWDS-NFSYIAEKNP-------------------RVSRIWNATIGVFFCGPK 532
Mb-Nox2         HKMNFGRPNWDS-EFTSVANTHA-------------------------GNNIGLFFCGPK 582
Hs-Nox4         SRLFIGRPRWKL-LFDEIAKYNR-------------------------GKTVGVFCCGPN 550
Nv-Nox4         ARLTHGRPDWFQ-IFRRVSQENP-------------------------KTCVGVFYCGAR 546
Lg-Nox4         PRTMFHRPDWAN-VLSSVSHLHC-------------------------RKTVDVFVCGPK 474
Dd-NoxA         TPTQFGRPKWDE-IFADHALRYAEK-------------------------DVGVFFCGPK 489
Dd-NoxB         SKTLFGRPNFRS-IFNQLTQLHQRE-------------------------KIGVFYCGNK 490
Ng-NoxA         SKRNFGRPNWDA-EFSDIRRYVEKNS------------------SGAKKPNVGVFVCGPG 561
Os-rboh-C       VKTHFARPNWRS-VFKKVAVSHE-------------------------NQRVGVFYCGEP 540
Os-rboh-G       VRTHFARPSWRD-VFKRVAVNHQ-------------------------GQRVGVFFCGDQ 505
At-rboh-D       VKSHFAKPNWRQ-VYKKIAVQHP-------------------------GKRIGVFYCGMP 532
Os-rboh-A       VRTHFARPNWRK-VLSKISSKHP-------------------------YAKIGVFYCGAP 552
Os-rboh-B       VRTHFARPNFKK-VLSKIASKHP-------------------------YAKIGVFYCGAP 552
Os-rboh-D       VRTHFARPNWKE-VFTRIASKHP-------------------------NSTVGVFYCGAP 565
Os-rboh-H       VRTHFARPNWKE-VFTRIASKHP-------------------------NSTVGVFYCGKP 609
Os-rboh-E       IRTHFARPNWRK-VFSDLANAHK-------------------------NSRIGVFYCGSP 541
Os-rboh-F       IRTHFARPNWRK-VFSDLANAHQ-------------------------NSRIGVFYCGSP 542
mPp-rboh-A      ARTHFARPNWKS-VFSGLTATHQ-------------------------DKRIGVFYCGPA 540
mPp-rboh-C      ARTHFARPNWKS-VFSKLAATHQ-------------------------EKRIGVFYCGPV 546
mPp-rboh-B      ARTHFARPNWKD-VFANLTFTHP-------------------------EKRIGVFYCGPA 527
mPp-rboh-D      ARTHFARPNWKN-VFTNMADTHP-------------------------NKRIGVFYCGPA 551
```

```
Ps-rboh-B       QLTHFGRPDWDK-VFSEAKANHP-------------------------GEEVGVFYCGPH 503
Ps-rboh-C       QLTHFGRPDWER-VFSDASAKHP-------------------------GEEVGVFCCGPH 492
Ps-rboh-A       QRTRFGRPDWNA-ELSRVAQNHRRLE--------------PLEDGDGEREEIGVFFCGPK 517
Hs-Duox1        SITHFGRPPFEP-FFNSLQEVHPQV------------------------RKIGVFSCGPP 487
Hs-Duox2        SITHFGRPPFEP-FFNSLQEVHPQV------------------------RKIGVFSCGPP 487
Lg-Duox1        ATTHFGRPKFQD-FLLSLAHEHDGV------------------------PQVGVFSCGPP 495
Lg-Duox2        AVTHFGRPKMED-FLHSLHHEYPEV------------------------ERFGVFSCGPG 494
Lg-Duox3        ATTHFGRPQLNN-IMTAVNRAHPMT------------------------RKIGVFSCGPP 497
Dp-Duox         AVNHFGRPDMTS-FLKFVQKKHSYV------------------------SKIGVFSCGPR 497
Cb-Duox         AKNHFGRPNFKT-FFQFIQNEHKEQ------------------------SEIGVFSCGPN 499
Mg-NoxC         NPTQFGRPDFVSILDRHYDDMRKYKAGLVARASRGGGGEDDAASVADDEVKVGVFFCGTP 605
Fg-NoxC         NPTHFGRPDFDLILDEHYEEMLKFRASKRTSTR----NKEDENYEEDEELKVGVFYCGAP 580
Cm-NoxD2        GRGKPGAPASIR-RFQRTRAFSN-------------------------KVHHGIFFCGGR 538
Cm-NoxD1        FERLFGNIIQVK-QFNRVSGFSN-------------------------DVQRGIFFCGNP 511
Pt-NoxD         HILTAGRPDWDALLAEALEKAHTSHP---------------------EGDAVGVFFCGAP 520
An-Fre1         IHFCNGRPDIRGAVENEAEIARECS------------------------TRLGVLCSGPG 720
Sc-Fre1         VECLNKRPDLKELVRSEIKLSELEN------------------------NNITFYSCGPA 655
                                                                         *

Dp-Nox5A        ELSKVLKKKC------------------NKFGFNFHKEIF------------- 717
Dp-Nox5B        QLAKELRKKC------------------NEFDFGFSKEIF------------- 718
Hs-Nox5         ALAKVLKGHC------------------EKFGFRFFQENF------------- 534
Lg-Nox5A        ALAKDLKEKS------------------DKFGFDFTKENF------------- 561
Lg-Nox5B        VVGKAIKEHC------------------AKFKFGFRKENF------------- 563
Dd-NoxC         QLSKELYEKCRYYTCL----------KTGGTKFYFHKENF------------- 575
Mg-NoxA         AAARDIKAACKTASVN-------------EVEFRFWKEHF------------- 580
Fg-NoxA         AAARDIKLACKAATVP-------------DVDFRFWKEHF------------- 557
Cc-NoxA         VLAKAIKEATIKNTNA-------------NVEFSFAKEHF------------- 557
Lb-NoxA         ALAKAIKDATVSHTNS-------------NVEFTFAKEHF------------- 551
fPp-NoxA        VMAKAIKEACVASTTP-------------NINFTFAKEHF------------- 557
Pg-NoxA         PLAKTLEQECRQATTD-------------KVKFEFHKERSVDSSTFMNPLECA 561
Bd-NoxA         ALAKVIKKHADQASDN-------------RVKFVLRKEHF------------- 503
Mg-NoxB         GLGSTLHIFCNKYS-------------EPDFAFVWGKENF------------- 525
Fg-NoxB         GLGSSLHTYCNKYT-------------EPGFSFVWGKENF------------- 528
Cc-NoxB         PLSKTLHSMCNKYS------------TPKGTRFFYGKENF------------- 534
Lb-NoxB         VLSKQLHQMSNKYS------------DPKGTRFFFGKENF------------- 547
fPp-NoxB        VLSKQLHISCNKYS------------SPSGTKFFFGKENF------------- 536
Pg-NoxB         PLGHSLHLQCNKWTGG----------TDNDTRFFWGKENF------------- 532
Bd-NoxB         VLSRTLHHTCNKWTEA----------TEDGTRFYYGKENF------------- 493
Hs-Nox2         ALAETLSKQSISNSES----------GPRGVHFIFNKENF------------- 570
Hs-Nox3         ALSRTLQKMCHLYSSA----------DPRGVHFYYNKESF------------- 568
Hs-Nox1         TLAKSLRKCCHRYSSL----------DPRKVQFYFNKENF------------- 564
Lg-Nox2         SLSTSLHSLCNKHS-T----------ESVGTRFYYNKENF------------- 563
Nv-Nox2A        ALSHTLHKMS-NAHSR----------DGDGAKFYYNKENF------------- 562
Nv-Nox2B        GLSHELHKMC-TQSCD----------ETKGVRFFYNKENF------------- 555
Ci-Nox2         ALSAKLHKMS-NKHS------------GGGVYFHYNKENF------------- 581
Sp-Nox2A        ALSSVLHENANKFTSL----------TPDGAKFFYNKENF------------- 582
Sp-Nox2B        SLSTILHQSCNKHTSD----------ESDGTRFVYYKENF------------- 562
Mb-Nox2         VLSSQLHVTCNKFTSER---------AAEGTKFYYNKENF------------- 613
Hs-Nox4         SLSKTLHKLSNQNNSY-------------GTRFEYNKESFS------------ 578
Nv-Nox4         GPSSMLRRCCQRMYKN-------------GASFVFNKEVSA------------ 574
Lg-Nox4         QLIYDVRKITLKCCNK-------------TNRFLLFEESF------------- 501
Dd-NoxA         LLSKSLYKASTHYTKT------------TTCRFHYNKENF------------- 517
Dd-NoxB         ALGKNIIKNCNKFNGK------------NNCHLIFHKENF------------- 518
Ng-NoxA         GLSKQVVYSYAVDKSKN-----------STVQFVFHKENF------------- 589
Os-rboh-C       VLVPQLRQLSADFT------------HKTNTRFDFHKENF------------- 568
Os-rboh-G       ALTPELRRLAQDFS------------HKTTTKFVFHKENF------------- 533
At-rboh-D       GMIKELKNLALDFS------------RKTTTKFDFHKENF------------- 560
Os-rboh-A       VLAQELSKLCHEFN------------GKCTTKFEFHKEHF------------- 580
Os-rboh-B       VLAQELSDLCHDFN------------GRCTSKFEFHKEHF------------- 580
Os-rboh-D       TLAKELKTLSHEMS------------HRTGTRFHFHKEYF------------- 593
Os-rboh-H       TLAKELKKLSLDMS------------HKTTTRFHFHKEYF------------- 637
Os-rboh-E       TLTKQLKDLSKEFS------------QTTTTRFHFHKENF------------- 569
Os-rboh-F       TLTKMLRDLSLEFS------------QTTTTRFHFHKENF------------- 570
```

```
mPp-rboh-A      ALANELENLSRSYT------------QTSSTKFSFHKENF------------- 568
mPp-rboh-C      TLANELENLSRTYT------------QKSSTKFSFHKENF------------- 574
mPp-rboh-B      ALVNELETLSRTYT------------KESSTKFSFHKENF------------- 555
mPp-rboh-D      SLVNELKTLSKAFT------------KKS-TKFFFHKENF------------- 578
Ps-rboh-B       ALEEILDNTCKRYSSS----------DPNGTIFDFHSEKFS------------ 534
Ps-rboh-C       ELEEVLANVCKKYTSS----------KADGTVFDFHSEKFA------------ 523
Ps-rboh-A       PLGNVIDEQCARLNQT----------VPD-VEFAFHSENF------------- 546
Hs-Duox1        GMTKNVEKACQLINRQ------------DRTHFSHHYENF------------- 515
Hs-Duox2        GMTKNVEKACQLVNRQ------------DRAHFMHHYENF------------- 515
Lg-Duox1        PMTSNVDKACSSLNKL------------EGPTYVHHFENF------------- 523
Lg-Duox2        PMTNSVQAACTLLNSI------------EGPTYCHHFENF------------- 522
Lg-Duox3        GVTKGVERACVDASKS------------TKAIFEHHYENF------------- 525
Dp-Duox         PLTKSIMSACEEVNQVR-----------KLPYFIHHFENFG------------ 527
Cb-Duox         NLNEKIAEGCAEANRQR-----------DAPSFAHRFETF------------- 528
Mg-NoxC         IVGEILADRCKALTARGRHDGS-------KIEYHFMIEVFN------------ 639
Fg-NoxC         VVGEILADKCRELTLRGWQDGI---HELGRIEWLVNYDGYDDHIN-------- 622
Cm-NoxD2        SVVRSVHQSIRAVAAARAA------RGLPQRNVFFMKENF------------- 572
Cm-NoxD1        ALGASVRRAMHKVQRQPER------LGT-KKPILFIQERF------------- 544
Pt-NoxD         AIARTLQRTAHKVTAQHHYTVRRATGTACRCRVLVHKENF------------- 560
An-Fre1         MMADECRLAVYEAMRR------------GCRGIRYFEEAFTW----------- 750
Sc-Fre1         TFNDDFRNAVVQGIDS-----------SLKIDVELEEESFTW----------- 686
```
