# Supplementary material for: Molecular evolution of Phox-related regulatory subunits for NADPH oxidase enzymes
Source: BMC Evol Biol. 2007 Sep 27;7:178. doi: 10.1186/1471-2148-7-178 (PMC2121648; doi:10.1186/1471-2148-7-178)
Supplement: Additional file 7 — Amino acid sequences of putative Nox regulatory subunits of L. gigantea, N. vectensis, M. brevicollis. Amino acid sequences of putative regulatory subunit proteins of L. gigantea (p47phox-, p67phox-, p22phox-like proteins), N. vectensis (p47phox-, p22phox-like proteins), and M. brevicollis (p47phox-, p22phox-like proteins) are provided. [file 1471-2148-7-178-S7.doc]

**Additional File 7**

# Amino acid sequences of putative Nox regulatory subunits of *L. gigantean,* *N. vectensis,* and *M. brevicollis.* The following naming was used to describe species of the sequences: gastropod snail-limpet-Lg (*L. gigantea*), sea anemone-Nv (*N. vectensis*), choanoflagellate-Mb (**M. brevicollis)**. Sequences are obtained from the indicated server: the DOE Joint Genome Institute (JGI) database (see Method section). The predicted choanoflagellate Mb-p47*phox* of JGI nucleotide database and protein database were obtained by TBLASTN and BLASTP searches, respectively.

>snail-Lg-p47*phox*: JGI database jgi|Lotgi1|123646|e_gw1.44.220.1

MFLIYLSIRDDTYITFSIQTYIKFLSVFDSTHITFYIRRLCYIFQVYVIFVTWSDSGTYVVYRRYSRFFDFQSALLDKFPIEGGSIDPESRIIPFLPGKIIFGRSHIRDVAVKRLGPINDYCKAVIALPPKISQCEEVLDFFEVETDDLDPPKAEEKKKKDEGAKKAENISDPKTLTQYRVTCDYEKQDRGEIDLEAGMIVEVAEKSETGWWFVNSDDAQGWVPSTYLEPADGSTTDNVVLRAQPGQEEKFICIEMFQSNGNDEISLEKGAVVEVLEKNLSGWWLVRHQGKEGFAPATYLNKTEDQFAVSLAKRKSLDVEIITNLSDISNIMKGDSPRNSLASTETPKPVTEMYSKVKSRSLERGGNIKPPPRQSMRMVSLSFTPSPSHKTSHYVTIAEFEDTVGDGLSFKEGQSVEIVEESDSGWWMVKLNGKSGWVPSAYLVERRKDAPVAAPPVPPHAASKRASRVIYKTCALFMAENEGELGFDEGETVDVMDKTDAEWWLVRIGNEEGWAPRSYIEQVEIEDTRGKPPAPPKGKPSIQFPLPAR

>snail-Lg-p67*phox*: JGI database jgi|Lotgi1|235403|estExt_fgenesh2_pg.C_sca_640024

MSLKQNIILWAEAVKLYEDGDLIECLEQLNKVNVSAKIRYNQGIVNSRLGNSDKAIEYLSQALQLDSFMSVAYFQRGLLFFDLHRYNEAEKDLEDSLINLRGSMVIDYKQLGLYTQLHLAQVLYNLAVIKKFKGDVNGFELYSTKFYNNPFNTELFSSVNTKLFNTVVNLRPLYNIFCPPKGYVSNLLKKDFLGKSEVVSSVEDNDFVLRREQKSRSNPSTPVVVRKNHLDVQTHRMAVTPPPLRPPPRLKSDAIPNDHVREPDSNVHPKPFSQLQIPQRTVPIPLSKVQAISKERNNSKSVIINDNGSQGQSSIKQFSPIEIPQKHSVSSTPVPVSTVITKKVVPVRPPPFIKDCDRKLKPERPPPPRFK

>snail-Lg-p22*phox*: JGI database jgi|Lotgi1|178875|fgenesh2_pm.C_sca_44000013

MGKIEWAMWANEQAIASSAVVLLGGIIAVAGQFPNWPFGAYGIVAGILVALLEYPRSKRQNGKCVERKFQRPLSRVVQCCGLITRNYFVRFVFYLLLTVPCCFILPTLLGGMCFFITSIIYFVAAIKGEEWKPVLEDYKPSQGPTVIEAPRHPPPRHPPTHNPNRASEV

>sea anemone-Nv-p47*phox*: JGI database jgi|Nemve1|174469|estExt_gwp.C_3070071

MTKRTIKDVKIVDVQKRKIPSKHYVYVISVTWSDGSVNVVYRRYSKFFDLQTKLLEEFPDEGGVKDPSARVLPFLPGKILFGRSHVRDVAVKRKEPIQEYCSKLVELPPKISQSGLVLKFFEPKPEDIELPLEKKKKKKKTADVISDPVSLEQYVAIADYQKQNRNEITMVAGDIVEVIDKNENGWWFVNLDEEQGWVPAAYLESVDGHSDDAAPIEGPTVEVGQYITTTSHKAELDDEITFETGVIVSVIQKNFDGWWLIRYQDKEGWAPAMYLRKPDPSQLHAAQAVGGLPSKDRVGAVTAPAAVRPGVNPVTDRCKKTINFKAETETVVTRKRKAHSVNVYGKGIEAMPALKSPDAEYFTVGEFRALGIQSGLDFSKGASVEVLDKNPNGWWYGKIDGNEGWIPSSYLGKREKSVKKKATTSPKPARFQAQELRTKPKPVMPDIPDTGSKPKPARPPVSKKITIIPNNELSNSFQNDLKSALRSAGPSAHGVEEVYVAKSDYEDKSDGENWAPANFLEMKSPEYAKQDKPAKHVYVALASYHDEDDDAISFDEGDEMEVVQQDDSGWWLVKIADKSGWAPSNFLKQL

>sea anemone-Nv-p22*phox*: JGI database jgi|Nemve1|245452|estExt_fgenesh1_pg.C_1600042

MGHTEWAMWANEQGVITSFVLALGGTLGVAGMFKKYEFGAYSIALGVLVFLIEYPRGKRKQGKKTQERSYQRCLTRLVSGLGIVGRNYYIRFVFYLLSCIPCCFFLPTILGGFSLFFTSIIYLCAALSGEKWQPCLDSKADRTPGINPSHHPTVPPPRNPRSQSLDNLGVSSSSVNRV

>choanoflagellate-Mb-p47*phox*-JGI protein database: JGI database jgi|Monbr1|17272|estExt_gwp_gw1.C_20623

MAKFVKNVTVIDYQRRRAPSKHYVYVITVVWSDGSEVTIYRRYSQLFEFHTSLLDRFPEAAGATGEERIIPFLPGKKIFGRSHTHKVAQSRAKPIDEYLKVLISLPAELSRCDLALELFEATNTDIAPPSEQERELPSRYTLNPPSPCAPGSRALLPVMVLDQYRAVADYTKQDRKELSFKTGDIFEVVEKNDNGWWFVSNDSASQGWVPATFLEPLDGELPLLSIFMHEKYITTAAYAASSDDEIGYEKGVVVRVLEKKLDGWWQVEYQGKVGWTPGTFLKRIEVCQ

>choanoflagellate-Mb-p47*phox*-JGI nucleotide database: JGI database fgenesh2_pg.scaffold_2000609, protein ID 22402

MAKFVKNVTVIDYQRRRAPSKHYVYVITVVWSDGSEVTIYRRYSQLFEFHTSLLDRFPEAAGATGEERIIPFLPGKKIFGRSHTHKVAQSRAKPIDEYLKVLISLPAELSRCDLALELFEATNTDIAPPSEQERERRPTGIFKKLSKSDDSGKEEREISVGDVMVLDQYRAVADYTKQDRKELSFKTGDIFEVVEKNDNGWWFVSNDSASQDEKYITTAAYAASSDDEIGYEKGVVVRVLEKKLDGWWQVEYQGKVGWTPGTFLKRIENSNAGPTTLKSTPASSTAATNGGVVPPAKPPAPQLRNEKEPPPRRESIRRPVSIHSKGPFAEIERARQIAAQNSGTKPAKPAVPALPKRNAASTNSGNDAVTIYTCTRKCDKQDDSGIALPAGARVELLEKSETGWYYVKYAGREGWAPADALQEENGVKATATSGAGVGPTVPRKVDTRPSVAPQSSAGVGAIAAALAQRASTMATGTASKPLKPTKPATPGKPATPSKLATPARSSGPKSDGATPPTLPLRPTMSGGTKPAKPVKPAKPSTPDASSKPAPVKPAKPSTPDTPSKPARPTMSGVKPKPSLPGRPEPAANKVPAKPARPGRPDPASSVGVAVAASPASNAKKARAKSDFEGQPGESISLKAGEIVTVEEEAATGWWFVIKADKSEGWAPADYLDLTGSNSDSQPGAPAKPAKPSKPSKPSMPAKPSRPGSNEASSNLRDDEFLVLDTAYNADGEGELSLRPREKVTVLEKATEWWFCRNAQGQEGWAPANYLSKR

>choanoflagellate-Mb-p22*phox* (partial): JGI database jgi|Monbr1|3080|gw1.8.321.1

GVLVLFVEWPRSARLKGSTIPRLYQYSIANIVDKLGPVARNYFARAIFWLGASIPCFFVFPAIAGALTLAVGALVYFLAAFKGEVWVKLEPQKERARGKVYEAPTRAPPR
