# Supplementary material for: Molecular evolution of Phox-related regulatory subunits for NADPH oxidase enzymes
Source: BMC Evol Biol. 2007 Sep 27;7:178. doi: 10.1186/1471-2148-7-178 (PMC2121648; doi:10.1186/1471-2148-7-178)
Supplement: Additional file 8 — Alignments of putative regulatory subunit proteins of L. gigantea(p47phox-, p67phox-, p22phox-like proteins), N. vectensis (p47phox-, p22phox-like proteins), and M. brevicollis (p47phox-, p22phox-like proteins). Alignment of p67phox-like protein (L. gigantea and H. sapiens), p47phox-like protein (L. gigantea, N. vectensis, M. brevicollis, S. purpuratus, and H. sapiens), p22phox-like proteins (L. gigantea, N. vectensis, M. brevicollis, and H. sapiens) are provided. [file 1471-2148-7-178-S8.pdf]

Additional File 8

Alignment of characteristic domains of putative regulatory subunit proteins of *L. gigantean* (p47*phox*-, p67*phox*-, p22*phox*-like proteins), *N. vectensis* (p47*phox*-, p22*phox*-like proteins), and *M. brevicollis* (p47*phox*-, p22*phox*-like proteins). The following naming was used to describe species of the sequences: Lg (*L. gigantea*), Nv (*N. vectensis*), Mb (*M. brevicollis*). Human (Hs, *H. sapiens*) and sea urchin (Sp, *S. purpuratus*) sequences were used as references.

Alignment of PX, bis-SH3 domains, and predicted AIR-like region of p47*phox*-like sequences. *Gray boxes* indicate the predicted domains corresponding to the PX (residues 3-132) and bis-SH3 (residues 151-285) domains of human p47*phox*. *Yellow boxes* indicate the predicted motif corresponding to the crucial residues of the AIR (residues 299-305 of human p47*phox*) containing a putative phosphorylation site by PKC (*bold letter*) consistent with a PKC substrate consensus motif (R/K-x-S/T-x-R/K). Polybasic region of *N. vectensis* is indicated by *underline.*

```
                                             PX-domain
Hs-p47phox    ---------------------MGDTFIRHIALLGFEKRFVPSQHYVYMFLVKWQDLSEKV 39
Nv-p47phox    ---------------------MTKRTIKDVKIVDVQKRKIPSKHYVYVISVTWSDGSVNV 39
Sp-p47phox    ---------------------MGKRTVVNANVTDIEKRREPTKHYVYIIHVTWSDGSVNV 39
Lg-p47phox    MFLIYLSIRDDTYITFSIQTYIKFLSVFDSTHITFYIRRLCYIFQVYVIFVTWSDSGTYV 60
Mb-p47phox    ----------------------MAKFVKNVTVIDYQRRRAPSKHYVYVITVVWSDGSEVT 38

Hs-p47phox    VYRRFTEIYEFHKTLKEMFPIEAGAINPENRIIPHLPAPKWFDG---QRAAENRQGTLTE 96
Nv-p47phox    VYRRYSKFFDLQTKLLEEFPDEGGVKDPSARVLPFLPGKILFGRSHVRDVAVKRKEPIQE 99
Sp-p47phox    VYRRYSTFFDFQNKLLSKFPEEAGANNPSSRCIPFLPGKKLFGRSHIREVALKRLSPIDE 99
Lg-p47phox    VYRRYSRFFDFQSALLDKFPIEGGSIDPESRIIPFLPGKIIFGRSHIRDVAVKRLGPIND 120
Mb-p47phox    IYRRYSQLFEFHTSLLDRFPEAAGATG-EERIIPFLPGKKIFGRSHTHKVAQSRAKPIDE 97

Hs-p47phox    YCGTLMSLPTKISRCPHLLDFFKVRPDDLKLPTDNQTKKPETYLMPKDGKSTATDITG-- 154
Nv-p47phox    YCSKLVELPPKISQSGLVLKFFEPKPEDIELPLEKK--KKKKKTADV--ISDPV------ 149
Sp-p47phox    YCTALVKLPGKISDSKEVINFFTPTPEDVSPPSPDGGGESTRGRADIGNISEPI------ 153
Lg-p47phox    YCKAVIALPPKISQCEEVLDFFEVETDDLDPPKAEEKKKKDEGAKKAENISDPK------ 174
Mb-p47phox    YLKVLISLPAELSRCDLALELFEATNTDIAPPSEQERERRPTGIFKKLSKSDDSGKEERE 157
```

```
                   bis-SH3 domain
Hs-p47phox    -----PIILQTYRAIANYEKTSGSEMALSTGDVVEVVEKSESGWWFCQMKAKRGWIPASF 209
Nv-p47phox    -------SLEQYVAIADYQKQNRNEITMVAGDIVEVIDKNENGWWFVNLDEEQGWVPAAY 202
Sp-p47phox    -------QAEQYIVVADYKKQQKNEVELTAGDLVEVFEKNDNGWWFVTVHDQHGWAPGTF 206
Lg-p47phox    -------TLTQYRVTCDYEKQDRGEIDLEAGMIVEVAEKSETGWWFVNSDDAQGWVPSTY 227
Mb-p47phox    ISVGDVMVLDQYRAVADYTKQDRKELSFKTGDIFEVVEKNDNGWWFVSNDSAS------- 210

Hs-p47phox    LEPLDSPDETEDP--EPNYAGEPYVAIKAYTAVEGDEVSLLEGEAVEVIHKLLDGWWVIR 267
Nv-p47phox    LESVDGHSDDAAPIEGPTVEVGQYITTTSHKAELDDEITFETGVIVSVIQKNFDGWWLIR 262
Sp-p47phox    LQNPDGQEEEDEETLIPGNDE-SYITNNAYQGQAEDEISFETGVVVTVIQKSLDGWWKVS 265
Lg-p47phox    LEPADGSTTDNVVLRAQPGQEEKFICIEMFQSNGNDEISLEKGAVVEVLEKNLSGWWLVR 287
Mb-p47phox    -------------------QDEKYITTAAYAASSDDEIGYEKGVVVRVLEKKLDGWWQVE 251

Hs-p47phox    KDDVTGYFPSMYLQK--------------------------------------------- 282
Nv-p47phox    YQDKEGWAPAMYLRKPD-PS---QLHAAQAVG-------GLPSKDRVG-AVTAPAAVRPG 310
Sp-p47phox    YQGKQGWAPATFLQIYKGPSGVTPKHPTQSIGNVMLLKSGSDSKPKPGPGPGSSGRPQPP 325
Lg-p47phox    HQGKEGFAPATYLNKTEDQFAV-SLAKRKSLDVEIITNLSDISNIMKGDSPRNSLASTET 346
Mb-p47phox    YQGKVGWTPGTFLKRIENSNAG--------------------------PTTLKSTPASST 284

                                   predicted AIR-like region
Hs-p47phox    -------SGQDVSQAQRQIKRGAPPRRSSIRNVHSIHQRSRKRLSQDAYRRNSV------ 329
Nv-p47phox    VNPVTDRCKKTINFKAETETVVTRKRKAHSVNVYGKGIEAMPALKSP------------- 357
Sp-p47phox    VQPRDEPGQLYSNYDAE--VKPTPPRRATVKKSVRRGGVRQTKPKLKK------------ 373
Lg-p47phox    PKPVTEMYSKVKSRSLERGGNIKPPPRQSMRMVSLSFTPSPSHK---------------- 390
Mb-p47phox    AATNGGVVPPAKPPAPQLRNEKEPPPRRESIRRPVSIHSKGPFAEIERAR---------- 334
```

Alignment of TRP and AD-like regions of p67*phox*-like sequences

Gray and *yellow boxes* indicate predicted motifs corresponding to the tetratricopeptide repeat (TPR, residues 6-153) and activation domain (AD, 196-210 residues) of human p67*phox*. *Red letters* indicate the amino acid residues corresponding to the two residues (D67 and R102 of human p67*phox*) crucial for binding to Rac.

```
                TPR
Lg-p67phox    MSLKQNIILWAEAVKLYEDGDLIECLEQLNKVN-VSAKIRYNQGIVNSRLGNSDKAIEYL 59
Hs-p67phox    MSLVEAISLWNEGVLAADKKDWKGALDAFSAVQDPHSRICFNIGCMYTILKNMTEAEKAF 60

Lg-p67phox    SQALQLDSFMSVAYFQRGLLFFDLHRYNEAEKDLEDSLINLRGSMVIDYKQLGLYTQLHL 119
Hs-p67phox    TRSINRDKHLAVAYFQRGMLYYQTEKYDLAIKDLKEALIQLRGNQLIDYKILGLQFKLFA 120

Lg-p67phox    AQVLYNLAVIKKFKG---DVNGFELYSTKFYNNPFNTELFSSVNTKLFNTVVN--LRPLY 174
Hs-p67phox    CEVLYNIAFMYAKKEEWKKAEEQLALATSMKSEPRHSKIDKAMECVWKQKLYEPVVIPVG 180
```

```
                          predicted AD
Lg-p67phox    NIFCPPKGYVSNLLKKDFLGKSEVVSSVEDND---------------------------- 206
Hs-p67phox    KLFRPNERQVAQLAKKDYLGKATVVASVVDQD---------------------------- 212
```

Alignment of p22*phox*-like sequence

Gray boxes indicate the amino acid sequences corresponding to the polybasic region (basic, residues 55-61 of human p22*phox*), predicted 1st and 2nd transmenbrane segments (TM1-TM2, residues 91-120 of human p22*phox*). Proline-rich region (PRR, residues 151-164 of human p22*phox*) are indicated by *yellow boxes*.

```
                                                                    basic
Lg-p22phox    MGKIEWAMWANEQAIASSAVVLLGGIIAVAGQFPNWPFGAYGIVAGILVALLEYPRSKRQ 60
Nv-p22phox    MGHTEWAMWANEQGVITSFVLALGGTLGVAGMFKKYEFGAYSIALGVLVFLIEYPRGKRK 60
Hs-p22phox    MGQIEWAMWANEQALASGLILITGGIVATAGRFTQWYFGAYSIVAGVFVCLLEYPRGKRK 60
Mb-p22phox    ---------------------------------------------GVLVLFVEWPRSARL 15

                                     TM1-TM2
Lg-p22phox    NGK-CVERKFQRPLSRVVQCCGLITRNYFVRFVFYLLLTVPCCFILPTLLGGMCFFITSI 119
Nv-p22phox    QGKKTQERSYQRCLTRLVSGLGIVGRNYYIRFVFYLLSCIPCCFFLPTILGGFSLFFTSI 120
Hs-p22phox    KGS-TMERWGQKYMTAVVKLFGPFTRNYYVRAVLHLLLSVPAGFLLATILGTACLAIASG 119
Mb-p22phox    KGS-TIPRLYQYSIANIVDKLGPVARNYFARAIFWLGASIPCFFVFPAIAGALTLAVGAL 74

                                     PRR
Lg-p22phox    IYFVAAIKGEEWKPVLEDYKP-SQGPTVIEAPRHPPPRHPPTHNPN-------------- 164
Nv-p22phox    IYLCAALSGEKWQPCLDSKADRTPGINPSHHPTVPPPRNPRSQSLDNLGV---------- 170
Hs-p22phox    IYLLAAVRGEQWTPIEPKPRERPQIGGTIKQPPSNPPPRPPAEARKKPSEEEAAVAAGGP 179
Mb-p22phox    VYFLAAFKGEVWVKLEPQKER---ARGKVYEAPTRAPPR--------------------- 110

Lg-p22phox    -RASEV---------- 169
Nv-p22phox    -SSSSVNRV------- 178
Hs-p22phox    PGGPQVNPIPVTDEVV 195
Mb-p22phox    ----------------
```
