# Supplementary material for: Molecular evolution of Phox-related regulatory subunits for NADPH oxidase enzymes
Source: BMC Evol Biol. 2007 Sep 27;7:178. doi: 10.1186/1471-2148-7-178 (PMC2121648; doi:10.1186/1471-2148-7-178)
Supplement: Additional file 9 — Amino acid sequences of Fre of an amoeboflagellate. Amino acid sequences of predicted Fre proteins of the amoeboflagellate Naegleria gruberi are provided. [file 1471-2148-7-178-S9.doc]

**Additional File 9**

**Amino acid sequences of predicted Fre proteins of amoeboflagellate.** Gene classification was determined by phylogenetic taxonomy and comparison of sequences with the 68 residues conserved in all Nox that previously identified (1) and other Fre sequences (e.g. *Aspergillus nidulans* Fre1 protein, GenBank No. AAN61438). Five Fre proteins of *Naegleria gruberi* (amoeboflagellate-Ng) are shown. The sequences were obtained from the DOE Joint Genome Institute (JGI) database (http://genome.jgi-psf.org/euk_home.html).

Reference

1. Kawahara, T., Quinn, M.T., Lambeth, J.D. Molecular evolution of the reactive oxygen-generating NADPH oxidase (Nox/Duox) family of enzymes. BMC Evol Biol. (2007) 7:109.

>amoeboflagellate-Ng-Fre1: JGI database jgi|Naegr1|51918|fgeneshHS_pg.scaffold_50000036

MNIYWKFHNKVTGLLQDDDLIEIAIVAKTKGWLAIGLDFNKTGMTQADCYIGFYDQTNSRPVLYDFWLPG

KSIPKNDSLFLGQDDILQLTGSLKDGTTTLKFLRKIDTGDIHADHPIRNRTIGVSYAFNINTIDVTQKHT

THGDFFINFVTQKGALNLAPAPLIQFHIIATASVAILMAAIGLSYTFISMKEKNRVISLVFHTKFTGLIK

NPILGSIFNPILELTIGEITLILFHLMLTAVWFCFGFFNSTSANVGKGFAYAIIINLTATILPITRYSAF

VFVFGISFERAVKYHKWVARLTLLIATIHGILMTVHLADNGSLGNILTASANDYILFGLIAWIAMVIMTI

FALEPIRRKFWELFKVIHIILAPVVLVMSSIHAKGWERTLPVLGIAMGLLVIDHLLRIIFGYIMPTKVIK

MEYEEKTGITTMVFEKPTISMWYMESLGMGKFVYVYIPGVSFFQSHPFTISSYSKLDSSIEFTCHVKNLG

RGWTKKLADFAMKRKYSAASIFARVEGPYGKLSVDLNTYSTVVLIAGGIGVTPINAIYNEITSNKVNASK

QNIYVLWTMKDEHMIQLFPNLTQPSSQVKQSFYITGENHKQVNESDTSYQYHYNMRPNFGKFFHMVSETV

GSDNSVAVVVCGPTQMNVDVYNAARRVGREERVNFHVHRETFEL

>amoeboflagellate-Ng-Fre2: JGI database jgi|Naegr1|46125|fgeneshHS_pg.scaffold_5000073

MMMFRYTGSIDIKDMYLATNSRSAPIQDTIDNLIFKDGGVSGSDLHVIFRRYLNTGDSSRDKIIRNETMR

FSYAYSLSSNDFQSYHDKTGKFTTNLLLIATNSSSGGTTTGTTNNLYNFHLIVTGIMFVIVLSLGIVFTF

IPVLVKPNLLTDLILYRRFCKVSTNKYLGGMINSLADLTIGEVLIVSSYWLILAAWAVYGGISASSFPAG

RAFAYVNVWNFSLILFPLSRYSVLLALFGISFERAIKFHKWLGRSTHFFVTVHFLAMLIENAMINNAAYL

WSMSTINYPLLGFISWILLSILVMGTFEPIRRKLWELFYGSHIILAVLVIGLSIAHGRGWITLLPYMAAS

ILLYLIDLLFRWVFGFGIPTKVVGISYNEDCSVTTVTFQKRFLTFLNIGGKPGKGSFIFVYLPSVSPFEY

HPFTVSSYKELDKKGTYEFTCHVKNHHGKGYGNKLANLAKSNPDVSRLFARVEGAFGNLSIPIQHYQTVI

LISGGIGITFVHSLLDGLVQRGEDKSKKIHLWWSFRHPSMLDLFPTIKNNSSIEKELFLTGKDTEMGSHS

HSDIKMSRMDVKQLLEKVKSNTNDSYVGVFVCGPTQLISTVQNAIFDVNGGGTRFHLHKEVFEF

>amoeboflagellate-Ng-Fre3: JGI database jgi|Naegr1|75169|fgeneshNG_pg.scaffold_77000085

MDAWMVAKPQSPVSDASDDILQRNGGYNSATGETHIKWTRFVKTGDSHDVIFTNAVMKTSWALHSTSTGMVRHTSTGKGPSLNFLSAGYATGEYGVLPSQPVVSDGGSSSSNSTCSLQTGYSQSILTQWVNSADYPTANY

KLVANYGTSDSLKLYWKVLTNPNDANVPIIEFGLIATGIYGYASIGFNHGTNSGMDTADTMFGYVDSSGN

PVILDTYMQGNLQAPTLDSSQDFISKNGGLRSVSGNYELHLKWTRLVNTKDSLDHLFTNAQISISWAIAK

TSTSMEYVHNYRGKNVMINFLQTATLAHLAAPPGSELATCGSSTGNIKDGSISMNSLTKLANSYRWHLIA

TAFICGLLVIGGFLLSVIGPRVNPNLFLDLILYRRLTKQVNLKFIGIYINTILDLTLGEAVVISCYWILV

AIWFAFGYVNATSAEAGKAFASVCVFNFGLILFPVTRYSVLQVLFGISFDRAIKYHRWLGILQWFFVTAH

GIAMVVHYNASGLYLVDVTSPTFPILGIIAWFFMTILWLMTFPVIRRKLWEVFLISHVILSILIVILSII

HGSGYINLLPYMALSILLYLFDVFLRVVFGFGIPTKIDSIKYDEKSQVTTITMRKPFLTFGSNPSMGHFI

FLYIPAVSKYQHHPITVSSCKEIDGGMIKKKELEFTVHVKNFGSGWSKQVAKIAQEKAQSANELICRVEG

AYGSLSVPLLRYKTIVMFGGGIGITPVHSIYSHLLEKNIQDKTVYTCWSIPTTDMIQVFPQLLETNGNKN

LKPNIFISKMKEENSQQTAFIHKGRANPKQFLEQVKKEMKERGELNAYVGVLVCGPEQLVTSVSNAIWDA

NERNGIRFHLHKEVFTW

>amoeboflagellate-Ng-Fre4: JGI database jgi|Naegr1|47775|fgeneshHS_pg.scaffold_14000124

MNTITVSTNEYPSSVQTDMLSFYWKFHTQQTGIIQGDDIIEMAIVAKTRGWLSIGLDFNKTGMNQADCFI

GFFDQENNLPILYDDWMPGKSIPKNDSIFGGTNDILQVTGSFVNGVTTLKFIRKVDTGDVLADKPIRNAT

VGISWAINSNSIDVTKVHSSYGQFNINLMTHVGILNLIKQPLIQFHLIATGSVALLMTLAGMSYVFISRK

EKNFFISLVFHTKFSNLIRNEFIGSWLNPILELTIGEITLIIIHLMLTSVWFCFGYFNNSSPILSKIGKA

FGYASVITLTTTILPITRYSAFVFIFGISFERAIKYHKWCSLLTLLVTTLHGIFMAIPMGMKGNLERLVS

VRANGYILFGTIAWIAMVIMSLFSIEIIRRKCWELFKVVHVILAPVVLVFSSIHARGWERTLPVLGVAIG

LLLVDYLLRFVFGYVLPTKVVKMEYDEKCQVTKIVFEKSNLGMWSTESLGMAKFVHVCIPGVSLFQSHPL

TISSYEKLPQSVEFTCHVKNNGKGWSKDVADFAVKRKCCASSIFARVEGPYGKLSLDVNSYSNVVLIAGG

IGVTPINAIFEELTNNSKHALQKNIFVIWTMKDENLMTMFPSLLERYSNVKQSFYVTSSASNSLKINSSD

AYNNRVHYNSRPNFVEYLTLISETVGCFDNSAAIIVCGPNQMNIDVYNASRKVAKESGVHFHIHRETFEM

>amoeboflagellate-Ng-Fre5: JGI database jgi|Naegr1|69148|fgeneshNG_pg.scaffold_32000174

MSTGDSQDATFKNSNVYVSWAYHSSSTSIRSTHTAYDRNIAINFLSVSPTETYSTGMINSGTSNNNNGGG

NNNGGNDDNEGDNDDDHDSGDSKKHLYHLYALGIAVFSLMVLGIITTILPASVKSNIFTHFLLYRKVSKV

IPYDFPLVSYVNQFLDLTLGELVIILVYAGLNVIWFVYGYLESQKVSSAFAKVIVFNYIFLFIPVTRYSV

LQALFGISFERSIKFHKWIGVMVFLSATAHGIVEFIEFKDNIPFMFSVEDGYPLLGIIAWLALGLLLLFS

MEPIRRKLYELFLVFHIPLTFITVTFSIIHGEGWINLLPYMAFSIILMVIDWTLRAIIGFGLHTQLVHIS

YDEESEVTTCVFEKRFLTLFHNPNHAHFVFVYIPQVSIYQLHPMTISSCEKLADSNHYRFTCHIKRMEGG

SWSTKLANLAKSNKPIDSYHGVRVEGAYGNLSIPFFNDSLLSRSEDKKRTIVFIVAGIGATPANAILTAL

EDPKYNEPTPYKVYVNLTSRNEAILKHFRILVQHKPNVDCTFYITGTKPSKSIEMLEKGQSTGIDHSTDR

RFIRGSRPNYVDYLNGVKAQVNQSKSLPYVSVFACGPSSMMNSVHNAVWSCSDMDCRFELHKEEFEF
