# Supplementary material for: Molecular evolution of Phox-related regulatory subunits for NADPH oxidase enzymes
Source: BMC Evol Biol. 2007 Sep 27;7:178. doi: 10.1186/1471-2148-7-178 (PMC2121648; doi:10.1186/1471-2148-7-178)
Supplement: Additional file 10 — Amino acid sequences of SH3PXD2 proteins. Amino acid sequences vertebrate and urochordate SH3 and PX domains 2 (SH3PXD2) proteins are provided. Alignments of PX and bis-SH3 domains of SH3PXD2, p47phox, NOXO1, p47phox-like proteins are shown. [file 1471-2148-7-178-S10.doc]

**Additional File 10**

**Amino acid sequences of SH3 and PX domains 2A and 2B (SH3PXD2a and SH3PDX2b) proteins.** We used the following naming: Hs (*H. sapiens*), Cf (*C. familliaris*), Rn (*R. norvegicus*), Mm (*M. musculus*), Gg (*G. gallus*), Xt (*X. tropicalis*), Dr (*D. rerio*), Tr (*T. rubripes*), Tn (*T. nigroviridis*), Ol (*O. latipes*)*,* Ci (*C. intestinalis*), Sp (*S. purpuratus*), Lg (*L. gigantea*), Nv (*N. vectensis*), Mb (M. brevicollis). Sequences are available from the indicated servers: http://www.ncbi.nlm.nih.gov/ (GenBank^TM^ numbering genes), http://www.ensembl.org (Ensembl numbering genes).

>Hs-SH3PXD2a: GenBank No. NP_055446

MLAYCVQDATVVDVEKRRNPSKHYVYIINVTWSDSTSQTIYRRYSKFFDLQMQLLDKFPIEGGQKDPKQRIIPFLPGKILFRRSHIRDVAVKRLKPIDEYCRALVRLPPHISQCDEVFRFFEARPEDVNPPKEDYGSSKRKSVWLSSWAESPKKDVTGADATAEPMILEQYVVVSNYKKQENSELSLQAGEVVDVIEKNESGWWFVSTSEEQGWVPATYLEAQNGTRDDSDINTSKTGEEEKYVTVQPYTSQSKDEIGFEKGVTVEVIRKNLEGWWYIRYLGKEGWAPASYLKKAKDDLPTRKKNLAGPVEIIGNIMEISNLLNKKASGDKETPPAEGEGHEAPIAKKEISLPILCNASNGSAVGVPDRTVSRLAQGSPAVARIAPQRAQISSPNLRTRPPPRRESSLGFQLPKPPEPPSVEVEYYTIAEFQSCISDGISFRGGQKAEVIDKNSGGWWYVQIGEKEGWAPASYIDKRKKPNLSRRTSTLTRPKVPPPAPPSKPKEAEEGPTGASESQDSPRKLKYEEPEYDIPAFGFDSEPELSEEPVEDRASGERRPAQPHRPSPASSLQRARFKVGESSEDVALEEETIYENEGFRPYAEDTLSARGSSGDSDSPGSSSLSLTRKNSPKSGSPKSSSLLKLKAEKNAQAEMGKNHSSASFSSSITINTTCCSSSSSSSSSLSKTSGDLKPRSASDAGIRGTPKVRAKKDADANAGLTSCPRAKPSVRPKPFLNRAESQSQEKMDISTLRRQLRPTGQLRGGLKGSKSEDSELPPQTASEAPSEGSRRSSSDLITLPATTPPCPTKKEWEGPATSYMTCSAYQKVQDSEISFPAGVEVQVLEKQESGWWYVRFGELEGWAPSHYLVLDENEQPDPSGKELDTVPAKGRQNEGKSDSLEKIERRVQALNTVNQSKKATPPIPSKPPGGFGKTSGTPAVKMRNGVRQVAVRPQSVFVSPPPKDNNLSCALRRNESLTATDGLRGVRRNSSFSTARSAAAEAKGRLAERAASQGSDSPLLPAQRNSIPVSPVRPKPIEKSQFIHNNLKDVYVSIADYEGDEETAGFQEGVSMEVLERNPNGWWYCQILDGVKPFKGWVPSNYLEKKN

>Mm-SH3PXD2a: GenBank No. NP_032044

MLAYCVQDATVVDVEKRRSPSKHYVYIINVTWSDSTSQTIYRRYSKFFDLQMQLLDKFPIEGGQKDPKQRIIPFLPGKILFRRSHIRDVAVKRLKPIDEYCRALVRLPPHISQCDEVFRFFEARPEDVNPPKEDYGSSKRKSVWLSSWAESPKKDVTGADTNAEPMILEQYVVVSNYKKQENSELSLQAGEVVDVIEKNESGWWFVSTSEEQGWVPATYLEAQNGTRDDSDINTSKTGEVSKRRKAHLRRLDRRWTLGGMVNRQHSREEKYVTVQPYTSQSKDEIGFEKGVTVEVIRKNLEGWWYIRYLGKEGWAPASYLKKAKDDLPTRKKNLAGPVEIIGNIMEISNLLNKKASGDKEAPAEGEGSEAPITKKEISLPILCNASNGSALAIPERTTSKLAQGSPAVARIAPQRAQISSPNLRTRPPPRRESSLGFQLPKPPEPPSVEVEYYTIAEFQSCISDGISFRGGQKAEVIDKNSGGWWYVQIGEKEGWAPASYIDKRKKPNLSRRTSTLTRPKVPPPAPPSKPKEAEENPVGACESQGSPLKVKYEEPEYDVPAFGFDSEPEMNEEPSGDRGSGDKHPAQPRRISPASSLQRAHFKVGESSEDVALEEETIYENEGFRPYTEDTLSARGSSGDSDSPGSSSLSLAVKNSPKSDSPKSSSLLKLKAEKNAQAELGKNQSNISFSSSVTISTTCSSSSSSSSLSKNNGDLKPRSASDAGIRDTPKVGTKKDPDVKAGLASCARAKPSVRPKPVLNRAESQSQEKMDISSLRRQLRPTGQLRGGLKGSRSEDSELPPQMASEGSRRGSADIIPLTATTPPCVPKKEWEGQGATYVTCSAYQKVQDSEISFPEGAEVHVLEKAVSGWWYVRFGELEGWAPSHYLVAEENQQPDTASKEGDTGKSSQNEGKSDSLEKIEKRVQALNTVNQSKRATPPIPSKPPGGFGKTSGTVAVKMRNGVRQVAVRPQSVFVSPPPKDNNLSCALRRNESLTATDSLRGVRRNSSFSTARSAAAEAKGRLAERAASQGSESPLLPTQRKGIPVSPVRPKPIEKSQFIHNNLKDVYISIADYEGDEETAGFQEGVSMEVLEKNPNGWWYCQILDEVKPFKGWVPSNYLEKKN

>Rn-SH3PXD2a: GenBank No. XP_001069452

MLAYCVQDATVVDVEKRRNPSKHYVYIINVTWSDSTSQTIYRRYSKFFDLQMQLLDKFPIEGGQKDPKQRIIPFLPGKILFRRSHIRDVAVKRLKPINEYCRALVRLPPHISQCDEVFRFFEARPEDVNPPKEDYGSSKRKSVWLSSWAESPKKDVTGADTNAEPMILEQYVVVSNYKKQENSELSLQAGEVVDVIEKNESGWWFVSTSEEQGWVPATYLEAQNGTRDDSDINTSKTGEVSKRRKAHLRRLDRRWTLGGMVNRQHSREEKYVTVQPYTSQSKDEIGFEKGVTVEVIRKNLEGWWYIRYLGKEGWAPASYLKKAKDDLPTRKKNLAGPVEIIGSIMEISNLLNKKASGDKEAPVEGEGSEAPISKKEISLPILCNASNGSASGIPERTTSKLAQGSPAVARIAPQRAQISSPNLRTRPPPRRESSLGFQLPKPPEPPSVEVEYYTIAEFQSCISDGISFRGGQKAEVIDKNSGGWWYVQIGEKEGWAPASYIDKRKKPNLSRRTSTLTRPKVPPPAPPGKPKETEENPAGACENQGSPLKLKYEEPEYDIPAFGFDSEPELNEEPTEDRGSGDKHPAQPRRTSPASSLQRAHFKVGESSEDVALEEETIYENEGFRPYTEDTLSARGSSGDSDSPGNSSLSLAMKNSPKSDSPKSSSLLKLKAEKNAQAELGKNQSNISFSSSVTIGTTSSSSSSSSSLSKNSGDLKPRSASDAGIRDSPKGGTKKDPDVKTGLASCVRAKPSVRPKPVLNRAESQSQEKMDISSLRRQLRPTGQLRGGLKGSRSEDSELPPQTASEGSRRGSADIIPLPATTPPCVPKKEWEGQGTAYVTCSAYQKVQDSEISFPAGAEVHVLEKVESGWWYVRFGELEGWAPSHYLVPEENQQPDPDSKEPDTVKSTQNEGKSDSLEKIEKRVQALNTVNQSKRATPPIPSKPPGGFGKTSGTVAVKMRNGVRQVAVRPQSVFVSPPPKDNNLSCALRRNESLTATDSVRGVRRNSSFSTARSAAAEAKGRLAERAASQGSESPLLPTQRNGIPVSPVRPKPIEKSQFIHNNLKDVYVSIADYEGDEETAGFQEGVSMEVLERNPNGWWYCQILDEVKPFKGWVPSNYLEKKN

>Cf-SH3PXD2a (partial): GenBank No. XP_544004

MNPRNSPKPFIPDEGHVVQTGRGGSGYVSVAGGQVYIINVTWSDSTSQTIYRRYSKFFDLQMQLLDKFPIEGGQKDPKQRIIPFLPGKILFRRSHIRDVAVKRLKPIDEYCRALVRLPPHISQCDEVFRFFEARPEDVNPPKEDYGGSKRKSVWLSSWAESPKKDATGADTNAEPMILEQYVVVSNYKKQENSELSLQAGEVVDVIEKNESGWWFVSTSEEQGWVPATYLEAQNGTRDDSDINTSKTGEVSKRRKAHLRRLDRRWTLGGMVNRQHSREEKYITVQPYTSQSKDEIGFEKGVTVEVIRKNLEGWWYIRYLGKEGWAPASYLKKAKDELPARKKNLAGPVEIIGNIMEISNLLNKKTSGDKETPPAEGEGPEAPITKKEISLPILCNASNGSALGVPERSISKLAQGSPAVARIAPQRAQISSPNLRTRPPPRRESSLGFQLPKPPEPPSVEVEYYTIAEFQSCISDGISFRGGQKAEVIDKNSGGWWYVQIGEKEGWAPASYIDKRKKPNLSRRTSTLTRPKVPPPAPPSKPKEAEEGPVGSTESQDSPLKLKYEEPEYDIPAFGFDSEPELSEEPTEDSGSGDRRPAQSHKPSPASSLQRARFRVGGSSEDVALEEETIYENEGFRPCAEDTLSARRSSRDSDSPGGSPLSLARKNSPKSGSPKSSSLLKLKAEKNAQAELGKNHSSASFSSSITINTTCSSSSSSSSLSKNSGDLKPRSASDAGIRGTPTVGPKRDADLKAGPTSCARAKPSVRPKPFLSRAESQSQERMDISTLRRQLRPTGQLRGGLKGSKSEDSELPPQTGFEGPGEGSRRGSADLITPPAATPPCPAKKGREGQATSYTTCSAYQKVQDSEISFPAGVEVQVLEKLGSGWWYVRFGELEGWAPSHYLVLGEDEQPDSSGKEPDPGSAKSKQNEGKSDSLEKIEKRVQALNTVNQNKRATPPIPSKPPGGFSKTSAAGAVKMRNGVRQVAVRPQSVFVSPPPKDNNLSCALRRNESLTATDGLRGVRRNSSFSTARSAAAEAKSRLAERAASQGSDPPLLPAQRNGIPVSPVRPKPIEKSQFIHNNLKDVYVSIADYEGDEETAGFQEGVSMEVLERNPNGWWYCQILDGAKPFKGWVPSNYLEKKN

>Gg-SH3PXD2a: GenBank No. XP_421741

MSSNIYKTFITTEKYKVYVDEKQSAMCCYYPKQSGWQLQARGFHWRSLYAWGFLKVYIINVTWSDLTSQIIYRRYSKFFDLQMQLLDKFPIEGGQKDPKQRIIPFLPGKILFRRSHVRDVAVKRLKPIDEYCRALVRLPPHISQCDEVFRFFEARPEDLNPPKEDYGSSKRKSVWMSSLSETPKKSVAPGADASSEPMILEQYVVVSNYEKQENSEISLQAGEVVDVIEKNESGWWFVSTAEEQGWVPATYLESQNGTRDDSDINTSKTGEEEKYVTIQPYASQGKDEIGFEKGVTVEVIQKNLEGWWYIRYLGKEGWAPASYLKKAKDDLPSRKKNLTGPVEIIGNIMEISNLLNKKSTDKETQAENEASETHITKKEISLPILCNDSNGNAMMTPDKQASKLAQGSPAIARIAPQRAQISSPNLRTRPPPRRESSLGFQLPKPPEPPSVEVEYYTIAEFQSCISDGISFRGGQKAEVIEKNSGGWWYVQIGEKEGWAPASYIDKRKKPNLNRRTSTLTRPKVPPPAPPSKPKDSEEGTTGMVSSGDTQDSPHKLKYEEPEYDVPTFGFDSECEVNESHREEGTPEKRLFQPLKPSPVSSLQKAKFKVGESSEDVANEEETIYENEGFRRYVEDALSNKESSGDSDSQKSSSLSLIRRNSPCSISPKSSFMKPKMEKDIQPDVGKCHYSRSEETKPRSASDVGLRSMPRTGMKKEPGQSPSIRAKPIVRPKPFLNKADSQSQEKMDISTLRRQLRPTGQLRGGLKGSRSEESESPPHTASENHDDHVRRGSADLITAPSRGIFCSSHPAKTEQENDTRCFYVTTDSYQKVQDSEICFPAGVEVEVLEKQASGWWYLKYGDMEGWAPSHYLALPDNQQETTSVETDASFARNRKNENKSNSLEKIEKRVQALNTINQSKRATPPIPSKPPGGFSKTSGPVKMRNGVRQLAVRPQSVFVSPPPKDNNLSCSLRRNESLTATDHLRNVRRNSSFSNARSQPGDVKGKQPERSGSEGSETTSNLPTQRNGIPVSTVRPKPIEKSQFIHNNLKDIYVSIADYEGDEETAGFQEGVCMEVLERNPNGWWYCQIMNGVKPFKGWVPSNYLEKKN

>Xt-SH3PXD2a: Ensembl No. **ENSXETP00000024885**

MLSFWVLDVSVQGVQKRRNPSKHYVYIINVTWSDSTSQVIYRRYSKFFDLQMQVLDKFPIEAGQKDPKQRIIPFLPGKILFRRSHIRDVAVKRLKPIDEYCRALVKLPAHISRCEEVLRFFEARPDDLDPPKEDYGSSKRKSVWMSSLCEIPKADPKGADGNSEPIILDQYVVVSNYEKQENSEISLKTGELVDVIEKNESGWWFVSTTEEQGWVPATYLDSQGGTKDDSEINTSKSGDVTKRRKAHLRRLDRRWTLGGIVNRQQSREEKYITVQPYTSQGKDEIGFEKGDTVEVIQKNLEGWWFIKYQGKEGWAPASYLKKAKDDIPCRKKNLTGPVEIIGNIMEISNLLNKKTPNDKESQGGNDSEEQTITKKEISLPILCNDSNGNSMMSPEKQIPKVTPGSPAVARIAPQRSEISSPNLRTKPPPRRESSLGFQLPKPPEPPSVEVEYYTIAEFQSCISDGISFRGGQKAEVIEKNSGGWWFVQIGEKEGWAPSSYIDKRKKPNLNRRTSTLTRPKVPPPAPPVKQKDSEDGINPLTCASTEPKDSPSKQIYEEPEYDVPAFGFDLEVELNVTNQSHGEELIQENLFQPSRPSPVSSLQRPKFRVGEFSEDVTNEEETIYENEGFRPLVEEALSSKESSGDSDSQKSSVTIQRNSPTAVSSKPPLLKYKSERNVQQETKNVSFSTSEEIKPRSASDVGLRAVPKVSTKKDAEQKQAVVPSTRAKPTVRPKPFLSKADSQNQDKMDISSLRRQLRPTGQLRSGLKGSKSEESENPPKTALENADSKPRRRSVDLTSNTSQTSSNNDKSEESAEQKSFYTATSTYQKVLDSEISFPVGAKIEVLDKQDSGWWYIKYKDSEGWAPSHFLEEVDNKKADTSIAESESTKIKKNENKSNSLEKIEKRVQALNTINQSKRATPPVPSRPPGGFSKPPGPGSNVVKLRNGVKQVTVRPQSVFVSAPPKDNNISCNLRRNESLSATDHLRGVRRNSSFNTVRSQPNQTKISSSAKPDGDMSESETSIRPSQKNGIPVSTVRPKPIEKSQFIHNNLKDIYISIADYEGDDETVGFQEGVSMEVLEKNPNGWWYCQILDGGKPFKGWVPSNYLEKKN

>Dr-SH3PXD2a: GenBank No. XP_690504

MAVYRGQATGRWQGRVGRGSAGRWLTACFFPSPAGLDSSEPMVLEQYVVVANYERQENSEISLKAGETVDVIEKSESGWWFVSTAEEQGWVPATYLDSQSGTRDDLDLGTSRSGEVTKRRKAHLKRLDRRWTLGGIVNRQQSREEKYVSVQAYASQGKDEIGFEKGVTVEVIQKNLEGWWYIRYQGKEGWAPASYLKKLKDDLSPRKKTLTGPVEIIGNIMEISNLLNKKAVSEKDIQTDGEATTPERHISKSEISLPMPYAPEAGVAPTVVTALGMNSGSSATLQENKSRAEPGSPAIARVAPHRVEIGSPNLRQKPPPRRDANLAFQLPKPPEAPTVEAEYYTIAEFQSSISDGISFRGGQKADVIEKNSGGWWYVQIGDTEGWAPSSYIDKRKKPNLSRRTSTLTRPKVPPPAPPVKKQDSEEGPSLGGSASKAPESPQRVYEEPEYDVPALGFDSELDCNPPKPKTHNSPKPEPRKFEIKSNPAAAERIAQAGKASPLLKVMTSPLRKRNSLENINKEEVIYENEGFRFSSDDFASGCDSHTPRSLTLGRKPFGSSSGGGKPLRKVSPDLNRSHSLGRAERHSSKLFSDESARNPKREPVMRKDVEIRIGQSPLARPKPVVRPKPLLTKSEPQSPERMDISSIRRHLRPTGSLRQGAIRAMRGEDSETASVVSSEDSTSSRSTSDLSSVYSKGSRGGESDHESVLFRTTDAYERAQESELSFPAGVEVEVLEKQESGWWFVRWGSDEGWVPTFYLEPIKHTHNVGIQESRDSPLVDLGSTNKSNSLEKNEQRVQALNNLNQQNLRSMSNPSPPIPSKPPGGFSKPTAMLNGSSVRMRNGVRQAAVRPQSVFVSPPQPLKETNIHTGSLRRNESLGAGDHLRSTGGVRRNSSFTAVRPQPVTDVRVRAGTTITAPAGSSSPLIAQRNGIPISTVRPKPIEKMQLIHNNLREVYVSIADYRGDEETMGFSEGTSLEVLEKNPNGWWYCQVLDGLQGRKGWVPSNYLERKK

>Tr-SH3PXD2a-1: Ensembl No. **SINFRUP00000137657**

VLDVKVVDVEKRRSPSKHYVYLINVTYSDNTSHIVYRRYSKFFDLQMQILDKFPIEGGQKDPKKRIIPFLPGKILFRRSHVRDVAMKRLRFIDDYCRALVRLPPQISQSEEVLRFFETKAEDINPPVDRSRGSTAGRWLTACFSPPGIESSEPMVLEQYVAVANYERQENSEINLKAGETVDVIEKSESGWWFVSTAEEQGWVPATYLDSQNATRDDLDLGTFRTGEVTKRRKAHLKRLDRRWTLGGIVNRQQSREEKYVTLQPYTSQGKDEVTFEKGVIVEVIQKNLEGWWFIRYLGKEGWAPASYLKKVKDDFSPRKKTMTGPVEIIGNIMEISNLLQKKSSSEKDIQTDGEGSTTPERHTSKNEISVPTPFSSEINAETGRRLSTTRDTNSPCLGIAASAALSENKARGDPGSPAVARVAPHRVEIGSPNLRQKPPPRRDTNLGFHLPKPPEPPAVEAEYYTIADFQSSISDGISFRGGQKADVIEKNPGGWWYVQIGEKEGWAPSSYIDKRKKPNLSRRSSTLTRPKVPPPAPPVKKQDSEEAPTSDNLPFKASDPPSRPVYEEPEYDVPPTGCEREPDTNPVKNERNPDVKTTSGVSDKYHSSPAFTKAAPPVCKAPSGVTHQRASFRSVEEVSKAECIYENQNFRRGAGSERNLVKGCSEPNSPKSYHSSTVPRRPSGTSPLAGRPIKTMTPETNRRSQTLGRRADLSCRSHDSSPHSSSDELSRAPKKTFSQGVEQRLGQSPSTRPKPSVRPKPLLAKSEAQSPERMDMSSLRRQLRPTSQFQHGLKPSRGEDSETASVISSEDSMCSRSTSDLSSVYSKGSRCDSDVEGPNLYRSLDAYKKVQDSEVSFPAGVEVEVLEKEQSGWWYIRWGSEEGWAPSYYLESVRQVGDAGSGGGKSDSLEKNEQNVLTLNNINIQGRNQQHRGLKRNTPPIPSKPPGGFSKPSGMVNGGVRMRNGVRQVAVRPQSVFVTTTQSAKDSHYMTGSLRRNDSLSRSDHYGSGSATLGVRRNASFSTVRPHVVVESHTRPVERSGVGSSGSSLSTGNVQDALARANQRNGIPVSAVRPKPIEKNQLIHNNLGRDVYVSIADYCGDEETMGFTEGTCLEVLERNPNGWWYCQVQDSLIPRKGWVPSNYLERK

>Tr-SH3PXD2a-2: Ensembl No. SINFRUP00000154137

VLDVNVVDVQKRRNPSKHYVYLINVTYSDSSSHVIYRRYSKFFELQMQILDKFPIEGGQKDPKKRIIPFLPGKVLFRRSHIRDVAVRRLKHLDNYCKALMKLPSQISQSEEVLKFFETKLDDLNPPTDRSRGPMAGSWLTACFFPTGPGLDASDPMLLEQYVVVASYEKQEPAEISLQAGEVVDVIEKSESGWWFVSTAEEQGWVPATYLNSHSGTRDDLDLGASKAGEVTKRHKAHLKRLDRRWTLGGVISRQQSREEKYVTVQSYTSEGKDEIAFEKGAVVEVIQKNLEGWWFIRYQDKEGWAPASYLKKMKDDLSPRKKAVTGPVEIIGNIMEISNLLNKKALSEKDVQTEGVPESPQAARKEISLPIPCAESSPASNPQEEKSKVEPASPAVARIAPHRVEIGSPVLRQKPPPRRDATLGFQLPSPPEPPTVEAEYYTIAEFQSCISDGITFSGGQKAEVIEKNSGGWWYVQIGEKEGWAPCSYIDKRKKPNLNRRTSTLCRPKVPPPAPPVKKQDSVETAPPSSPGSEAPESPVYQGRPVYEEPEYDVPAIGDLDLESEFEFLRGESSLVDGKNEDTSSEKGSHMSSKPSPASSLHSASFKMGESFEDGHDAGGEAEGDEECIYENDGFRPFKETPERQCSRDSSSSRTSVFSESSKTAGGGWRAGGSKFKGDSNGSSFSNKFEEGTGPKSALAESQNKREQEESKPVSSTLSSSKLKPVVRPKPQLAKTSSSEQMDISSLRRQLRPTGQLKNSIKMKNEDSETASVISSEDSFSSQSTSDLSSIYSKGSRGDSDLEGCAVYRTTDPYEKVQESELSFPAGVEVEVLEKQESGWWYVRWGDTEGWAPTYYLEPLRQQDDFAGSESEGSPSKPGSLSKSNSLEKNEQRVQALNNINQNLKKVTPPIPSKPPGGLSKPISFFGSRKQNSAKQQVVRPQSVLISAPIMDPPSSLGALRRNESLNSTDHPRVSPTVRRNASFGTAPRGLVANNLALPSRNRSGTGSSESLGLGSVKNSLPVSTVKPKPHIIHNNLREIYVSIADYHGDEETMGFPEGTSLEVLDRNPNGWWYCKILDNGKQRKGWVPSNYLERKH

>Tn-SH3PXD2a (partial): Ensembl No. **GSTENT00026428001**

MIGIESSEPMVLEQYVAVANYERQENSEINLKAGETVDVIEKSESGWWFVSTAEEQGWVRATYLDSQNATRDDLDRGTFRTGEEEKYVTLQSYTSQGKDEVSFEKGVTVEVIQKNLEGWWFIRYLGKEGWAPASYLKKVKDDFSPRKKTMTGPVEIIGNIMEISNLLQKKSVSEKDIQTDGEGSTTPERHISKNEISMPMPFSSEINAETGRRLSTTRDTNSPCLGIAASAALSETKARGDPGSPAVARVAPHRVEIGSPNLRQKPPPRRDANLGFHLPKPPEPPAVEAEYYTIADFQSSISDGISFRGGQKADVIEKNPGGWWYVQIGEMEGWAPSSYIDKRKKPNLSRRSSTLTRPKVPPPAPPVKKQDSEEAPASDSSSFKASDPPSRPVYEEPEYDVPPVGCEKESDTDSVKNERNVDVKISSGVSDKNHSSPSFTKASPPICKVPSVITYQRAPFRSVEEVSKAECIYENENFRRSERNLVKGSSEPNSPKSYHSSTVPRRPSGAAPFAGRPVKTVTPEMNRRSQTLGRRMEVCCRSHDGSPHSSSDELSRGPKKAFSQDVEQRIGQSPSTRPKPSVRPKPLLTKSEPQSPERMDMSSLRRQLRPTSQFQHGLKPPRGDDSETASVVSSEGSVCSRSTSDLSSVYSKGSRGDSDAEGPNLYRSVDTYKKVQDSEVSFPAGVEVEVLEKEESGWWYIRWGSEEGWAPSYYLEPVRQVGDAGSGGSKSNSLEKNKQNVLTLNNINIQGRNQQHQGLKRNTPPIPSKPPGGFSKPSGMVNGGVRLRNGVRQVAVRPQSVFVTTSQSAKDSHYMTGSLRRNDSLGRSDHYGSGSATLGVRRNASFSTVRPHVVVESQTRPVERSGISSSGSSLSTGNVQDVLTRVNQRNGIPVSAVRPKPIEKNQLIHNNLGRDVYVSIADYCGDEETMGFTEGTCLEVLERNPNGWWYCQVQDSLIPRKGWVPSNYLERKK

>Hs-SH3PXD2b: GenBank No. NP_001017995.1

MPPRRSIVEVKVLDVQKRRVPNKHYVYIIRVTWSSGSTEAIYRRYSKFFDLQMQMLDKFPMEGGQKDPKQRIIPFLPGKILFRRSHIRDVAVKRLIPIDEYCKALIQLPPYISQCDEVLQFFETRPEDLNPPKEEHIGKKKSGGDQTSVDPMVLEQYVVVANYQKQESSEISLSVGQVVDIIEKNESGWWFVSTAEEQGWVPATCLEGQDGVQDEFSLQPEEEEKYTVIYPYTARDQDEMNLERGAVVEVIQKNLEGWWKIRYQGKEGWAPASYLKKNSGEPLPPKPGPGSPSHPGALDLDGVSRQQNAVGREKELLSSQRDGRFEGRPVPDGDAKQRSPKMRQRPPPRRDMTIPRGLNLPKPPIPPQVEEEYYTIAEFQTTIPDGISFQAGLKVEVIEKNLSGWWYIQIEDKEGWAPATFIDKYKKTSNASRPNFLAPLPHEVTQLRLGEAAALENNTGSEATGPSRPLPDAPHGVMDSGLPWSKDWKGSKDVLRKASSDMSASAGYEEISDPDMEEKPSLPPRKESIIKSEGELLERERERQRTEQLRGPTPKPPGVILPMMPAKHIPPARDSRRPEPKPDKSRLFQLKNDMGLECGHKVLAKEVKKPNLRPISKSKTDLPEEKPDATPQNPFLKSRPQVRPKPAPSPKTEPPQGEDQVDICNLRSKLRPAKSQDKSLLDGEGPQAVGGQDVAFSRSFLPGEGPGRAQDRTGKQDGLSPKEISCRAPPRPAKTTDPVSKSVPVPLQEAPQQRPVVPPRRPPPPKKTSSSSRPLPEVRGPQCEGHESRAAPTPGRALLVPPKAKPFLSNSLGGQDDTRGKGSLGPWGTGKIGENREKAAAASVPNADGLKDSLYVAVADFEGDKDTSSFQEGTVFEVREKNSSGWWFCQVLSGAPSWEGWIPSNYLRKKP

>Mm-SH3PXD2b: GenBank No. NP_796338.2

MPPRRSIVEVKVLDVQKRRVPNKHYVYIIRVTWSSGATEAIYRRYSKFFDLQMQMLDKFPMEGGQKDPKQRIIPFLPGKILFRRSHIRDVAVKRLIPIDEYCKALIQLPPYISQCDEVLQFFETRPEDLNPPKEEHIGKKKSGNDPTSVDPMVLEQYVVVADYQKQESSEISLSVGQVVDIIEKNESGWWFVSTAEEQGWVPATCLEGQDGVQDEFSLQPEEEEKYTVIYPYTARDQDEMNLERGAVVEVVQKNLEGWWKIRYQGKEGWAPASYLKKNSGEPLPPKLGPSSPAHSGALDLDGVSRHQNAMGREKELLNNQRDGRFEGRLVPDGDVKQRSPKMRQRPPPRRDMTIPRGLNLPKPPIPPQVEEEYYTIAEFQTTIPDGISFQAGLKVEVIEKSLSGWWYIQMEDKEGWAPATFIDKYKKTSSASRPNFLAPLPHEMTQLRLGDAAATENNTGPEAVGPSRPLPEAPHGAVDSGMLWSKDWKGGKEAPRKASSDLSASTGYEEISDPTQEEKPSLPPRKESIIKSEEELLERERQKMEPLRGSSPKPPGMILPMIPAKHAPLARDSRKPEPKLDKSKFPLRNDMGLECGHKVLAKEVKKPNLRPISRSKAELSEEKVDPTSQNLFMKSRPQVRPKPTPSPKTEPAQSEDHVDIYNLRSKLRPAKSQEKALLDGESHHAAGSHDTALSRSFLPGEGPGHGQDRSGRQDGLSPKETPCRAPPRPAKTTDPGPKNVPVPVQEATLQQRPVVPPRRPPPPKKTSSSPLSCRPLPEVRGAQREESRVAPAAGRALLVPPKAKPFLSNSSVGQDDMRGKGGLGPRVTGKVGETREKAASFLNADGPKDSLYVAVANFEGDEDTSSFQEGTVFEVREKNSSGWWFCQVLSGAPSWEGWIPSNYLRKKP

>Rn-SH3PXD2b: GenBank No. XP_001063612.1

MPPRRSIVEVKVLDVQKRRVPNKHYVYIIRVTWSSGATEAIYRRYSKFFDLQMQMLDKFPVEGGQKDPKQRIIPFLPGKILFRRSHIRDVAVKRLIPIDEYCKALIQLPPYISQCDEVLQFFETRPEDLNPPKEEHIGKKKSGSDPTSVDPMVLEQYVVVANYQKQESSEISLSVGQVVDIIEKNESGWWFVSTAEEQGWVPATCLEGQDGVQDEFSLQPEEEEKYTVIYPYTARDQDEMNLERGVVVEVIQKNLEGWWKIRFQGKEGWAPASYLKKSSGEPLPPKLGPSSAHSGALDLDGVSRQQNAVGREKELLNNQRDGRFEGRLAPDGDVKQRSPKMRQRPPPRRDMTIPRGLNLPKPPIPPQVEEEYYTIAEFQTTIPDGISFQAGLKVEVIEKSLSGWWYIQMEDKEGWAPATFIDKYKKTSSASRPNFLAPLPHEMTQLRLGEAAATENNTGPEAVGPSRPLPEAPHGAVDSGMLWSKDWKGGKEAPRKASSDLSASTGYEEISDPTQEEKPSLPPRKESIIKSEEELLERERQKMEPHRGSSPKPPGVILPMIPAKHAPLARDSKKPEPKPDKSKFPLRNDMGLECGHKVLAKEVKKPNLRPISRSKAELPEEKVEPNPQNLFLKSRPQVRPKPTPSPKTEPAQGEDQVDIYNLRSKLRPAKSQEKALLDGESHHAAGGHDTALGRSFLPGEGPGRGQDRSGRQDGLSPKETPCRAPPRPAKTTDPGPKNVPVPVQEATQQRPVVPPRRPPPPKKTSSSPLSCRPLPEVRGSQREESRAIPASGRALLVPPKAKPFLSNSSVGQDDIRGKGGLGPRIAGKVGETREKAASFLNADGPKDSLYVAVANFEGDEDTSSFQEGTVFEVREKNSSGWWFCRVLSGAPSWEGWIPSNYLRKKP

>Gg-SH3PXD2b : GenBank No. XP_425197

MGPLEVYIIKVTWSNGSTEVIYRRYSKFFDLQMQMLDKFPMEGGQKDPKQRIIPFLPGKILFRRSHIRDVAVKRLIPIDEYCKALIQLPPYISQCEEVLQFFETRPDDLTPPKEEPIGKKKSGADSASVDPLVLEQYVVVANYQKQESSEISLCVGQLVDIIEKNESGWWFVSTSEEQGWVPATCLEAQDGVQDELSMQPDEEEKYTVIYPYTARDQDEMNLDKGAVVVVIQKNLEGWWKIRYQGQEGWAPASYLKKGNGEMFSQKLGSGSSAHSCALDLDGISRQQAVTSREKDGRFDNRPLPNADIRRKSPKMRQRPPPRRDLTIPRGLNLPKPPVPPQVEEEYYTIADFQTTIPDGISFQAGMKVEVIEKNLSGWWYIQIEEKEGWAPATFIDKYKKTSNASRPNFLAPLPSEMAQLRLGDAAAESSATEEATGPCRPLPEAPPNGMDCGMKRAKDWKGKEATESGDLAFTCGYEEISDRDVEEKPSLPPRKESIIKSEGELLERQRPPPKPPGMILPMIPPKQSAAPKDSKKPELKPEKGKLFQLKNEMGLECGHKVSAKEVKKPNLRPIVKPTKPKAEPVEDKPEPITQNPFLKSRPQIKPKPAAAPRTDPPPADDKLDICSLRSKLRPAKCPEKPPEQDTAASESSCSTPAVAPEASGRFQERPSVENKALPKSPPGPAVAPAAREPTPQRPVVPPRRPPPPKKTTSPVAGPVPEARASPLPGRPMLVPPKARPFLSAAIQDEAKVKSSVGPKVISKAVERGEGRERTSAPFSNPDVSKEALYVAVADFEGDEETNSFREGTLFEVREKNSSGWWFCKVLTGGPCWEGWIPSNYLRKKP

>Dr-SH3PXD2b: Ensembl No. ENSDARG00000021377

MPRRTVLEVTVQDVQKRRNPNKHYVYIIKVAWSDGSTEVIFRRYSKFFDLQMELLDKFPVEGGQKDPKRRIIPFLPGKILFRRSHIRDVAMKRLKPINEYCRALIQLPVYISQCEEVRVFFETRPEDLNPPKEEPSGKKKSGGDSSSADPLLLDQYVAVTDYEKQESSEISLYVGQVVEVIEKNESGWWFVSTEDAQGWVPATCLEAQDDPDDFSLPAEEEEKYTAIYPYSARDQDEIDLERGMTVEVIQKNLEGWWKIRYQGKEGWAPASYLKKADILSQKMAAGAPVHASTNDLDVACKQQNANKENKENQRDRFSPFSDSKRKVGARQRPPPRRDLTIPRGVNLPKPPVPPQVEEEYYTIADFQTTIPDGISFQAGLKVEVIEKNSSGWWYIQIEDKEGWAPVTFIDKYKKTSSASRPNFLAPLPGEMEQLKLEDTSSNSTNSEHTWSKPLPDEPSSNSDLSTRSKLREWKPNAAKSSSHFSGPLPPPPSSPTAEEKPALPPRRESINKSLELEDKPKAELSKPLPPKPPVPGVIAPLVTPKAAPLKPDKPPEMKKDDKNKQ

>Cf-SH3PXD2b: GenBank No. XP_546237.2

VYIIRVTWSSGSTEAIYRRYSKFFDLQMQMLDKFPMEGGQKDPKQRIIPFLPGKILFRRSHIRDVAVKRLIPIDEYCKALIQLPPYISQCDEVLQFFETRPEDLNPPKEEHVGKKKSGGDLTSVDPMVLEQYVVVADYQKQESSEISLSVGQVVDIIEKNESGWWFVSTAEEQGWVPATCLEGQDGMQDEFSLQPEEEEKYTVIYPYTARDQDEMNLERGAVVEVIQKNLEGWWKIRYQGKEGWAPASYLKKSSGEPLPPKPGTGSPAHTGILDLDGLSRQQSSVGRDRELLNNQRDGRFEGRPVPDGDIKQRSPKMRQRPPPRRDMTIPRGLHLPKPPVPPQVEEEYYTIAEFQTTIPDGISFQAGLKVEVIEKNLSGWWYIQIEDKEGWAPATFIDKYKKTSNASRPNFLAPLPNEVTQLHLGDAAAMENNTGSEAIGPSRPLPDAPHGAMDSGMPWSKDWKGGKEVPRKASSDMSSCAGYEEISSPDLEEKPSLPPRKESIIKSEGELQERERQRMEQLRGSSPKPPGMILPMIPAKHTPPARDGRRSEPKPDKGKLLQLKNEMGLECGHKVLAKEVKKPNLRPISKSKADLPEEKPEGIPQNPFLKSKPQVRPKPAPSPRTEPPQGEDQVDICNLRSKLRPAKSQEKPLLDGEGSQDVACSRSFLPGEGPGRTQDRTGKQDGLSPKEVPCRAPPKPVKTADPVPKNVPTPLQEASPQRPVVLPRRPPPPKKTSSSRPLPEVRGPQREASEGKAAPAPGRALLVPPKAKPFLSNSSGGHDDMRGKGGLGPWLVGKIGENREKVAAAPFPSADGSKDSLYVAVANFEGDKDTSSFQEGTVFEVREKSSSGWWFCQVLSGAPSWEGWIPSNYLKKKP

>Ci-SH3PXD2: Ensembl No. ENSCING00000012164

MSQIPKRLITAVNVNRAEKRRVPSKHYVYVIEVSWDDGSDTVIFRRYSKFFDLQISLLETFPKEGGMKDPSTRIIPFLPGKILFRRSNIRDVAMKRVLSIGEYCKDLIKLPSYIVQHSLILDFFETKPEDLKPPESESTSRASKRNSLDISQPILPESYVVVQDYIKTQPKELNARVGEVVEVMDKHENGWWFVSTEDGEQGWVPGVYLGKPDGKSENLVIKQDQLGQGELYLTTTQYNGEDSEVSFNTGVLVEVLQKNLEGWWFVSYNGKQGWAPASYLTKPPESVTAISLSKKLSSPVKNDSSIPSSHSCTSLSSQSSSNAGDTSSPSHKRLSGVGIKRPSLKPQVPPPPPPQHSDDVQYIAMHSFDGKIPHGVAFNIDDPVTVLSKSPGWWYVEVNGNEGWAPETYISKTTKPKPTPARPQPPSFPKKTELQRASHTKVTRSSSLRQDKPAVMDRVEHTYSNDTNFRNFKPKPTLPKKPLKPAVNNQKPTFNAGNGQTPLPN
