# Supplementary material for: Molecular evolution of Phox-related regulatory subunits for NADPH oxidase enzymes
Source: BMC Evol Biol. 2007 Sep 27;7:178. doi: 10.1186/1471-2148-7-178 (PMC2121648; doi:10.1186/1471-2148-7-178)
Supplement: Additional file 11 — Alignment of vertebrate p47phox, vertebrate NOXO1, p47phox-like protein, and chordate SH3PXD2 proteins. Alignments of SH3PXD2a and 2b of H. sapiens, C. familliaris, R. norvegicus, M. musculus, G. gallus, X. tropicalis, D. rerio, T. rubripes, T. nigroviridis, O. latipes, and C. intestinalis with other p47phox family proteins are shown. [file 1471-2148-7-178-S11.pdf]

Additional File 11

Alignment of vertebrate p47*phox*, vertebrate NOXO1, chordate SH3PXD2 proteins, and 47*phox*-like proteins. *Gray boxes* indicate the predicted domains corresponding to the PX (residues 3-132) and bis-SH3 (residues 151-282) domains of human p47*phox*. *Blue boxes* indicate the predicted C-terminal SH3 domains of human SH3PXD2a and 2b. *Red letters* indicate the characteristic amino acid residues of human p47*phox* PX domain (Arg-42, Pro-73, and Arg-90). Abbreviations: Hs (*H. sapiens*), Cf (*C. familliaris*), Rn (*R. norvegicus*), Mm (*M. musculus*), Gg (*G. gallus*), Xt (*X. tropicalis*), Dr (*D. rerio*), Tr (*T. rubripes*), Tn (*T. nigroviridis*), Ol (*O. latipes*), Ci (*C. intestinalis*), Sp (*S. purpuratus*), Lg (*L. gigantea*), Nv (*N. vectensis*), Mb (*M. brevicollis*).

```
                                    PX-domain
Hs-p47phox      ---------------------MGDTFIRHIALLGFEKRFVPSQHY--------------- 24
Mm-p47phox      ---------------------MGDTFIRHIALLGFEKRFIPSQHY--------------- 24
Rn-p47phox      ---------------------MGDTFIRHIALLGFEKRFVPSQHY--------------- 24
Cf-p47phox      ---------------------MGDTFIRHIALLGFEKRFVPSQHY--------------- 24
Gg-p47phox      ---------------------VGDTFIRHIELLRYEKRFFPSQHY--------------- 24
Xt-p47phox      ---------------------MTEPHIRHIQLLGFEKRFIPSQHY--------------- 24
Tn-p47phox      ---------------------MADTYVRHVQLLGFEKRFFPSQHY--------------- 24
Tr-p47phox      ---------------------MAETYVRHVQLLGFEKRFFPSQHY--------------- 24
Ol-p47phox      ---------------------MEDVYVRHVELLGIEKRFFPTQHF--------------- 24
Dr-p47phox      ---------------------MAETYVRHVELLGFEKRFFPSQHY--------------- 24
Mm-SH3PXD2a     ---------------------MLAYCVQDATVVDVEKRRSPSKHY--------------- 24
Rn-SH3PXD2a     ---------------------MLAYCVQDATVVDVEKRRNPSKHY--------------- 24
Hs-SH3PXD2a     ---------------------MLAYCVQDATVVDVEKRRNPSKHY--------------- 24
Gg-SH3PXD2a     ----------------MSSNIYKTFITTEKYKVYVDEKQSAMCCYYPKQSGWQLQARGFH 44
Xt-SH3PXD2a     ---------------------MLSFWVLDVSVQGVQKRRNPSKHY--------------- 24
Dr-SH3PXD2a     ------------------------------------------------------------
```

Tr-SH3PXD2a1	--------------------------VLDVKVVDVEKRRSPSKHY---------------	19
Tr-SH3PXD2a2	--------------------------VLDVNVVDVQKRRNPSKHY---------------	19
Hs-SH3PXD2b	--------------------MPPRRSIVEVKVLDVQKRRVPNKHY---------------	25
Cf-SH3PXD2b	--	
Mm-SH3PXD2b	--------------------MPPRRSIVEVKVLDVQKRRVPNKHY---------------	25
Rn-SH3PXD2b	--------------------MPPRRSIVEVKVLDVQKRRVPNKHY---------------	25
Gg-SH3PXD2b	--MGPLE---------------	5
Dr-SH3PXD2b	---------------------MPRRTVLEVTVQDVQKRRNPNKHY---------------	24
Lg-p47phox-like	MFLIYLSIRDDTYITFSIQTYIKFLSVFDSTHITFYIRRLCYIFQ---------------	45
Ci-SH3PXD2	------------------MSQIPKRLITAVNVNRAEKRRVPSKHY---------------	27
Ci-p47phox	---------------------MVNRTLKSVKVIDIEKRRLPSKHY---------------	24
Sp-p47phox	---------------------MGKRTVVNANVTDIEKRREPTKHY---------------	24
Nv-p47phox-like	---------------------MTKRTIKDVKIVDVQKRKIPSKHY---------------	24
Mb-p47phox-like	-----------------------MAKFVKNVTVIDYQRRAPSKHY---------------	23
Hs-NOXO1	--------------MAGPRYPVSVQGAALVQIKRLQT-----------------------	23
Cf-NOXO1	--------------MAGSRHPVSVRAAALVQTGRLQT-----------------------	23
Mm-NOXO1	--------------MASPRHPVSAHAVALVQMDRLQT-----------------------	23
Rn-NOXO1	--------------MASPRHPVSAHAVALVQMERLQT-----------------------	23
Gg-NOXO1	--	
Xt-NOXO1	--	
Dr-NOXO1	---------------------LIKLFIRRVEHRNMDINIHSSPPQ---------------	24
Ol-NOXO1	--------------------PADTHMSLSVSAPGSDKWLLPSDDRKY-------------	27
Tn-NOXO1	--------------METQRYPISARLVGVLHKEKSKVGAHKSQVVLFHARFNQQLCPLQ-	45

Hs-p47phox	-----------VYMFLVKWQDLSEKVVYRRFTEIYEFHKTLKEMFPIEAGAINPENRIIP	73
Mm-p47phox	-----------VYMFLVKWQDLSEKVVYRKFTEIYEFHKMLKEMFPIEAGEIHTENRVIP	73
Rn-p47phox	-----------VYMFLVKWQDLSEKVVYRKFTEIYEFHKMLKEMFPIEAGEIHTENRVIP	73
Cf-p47phox	-----------VYMFLVKWHDLSEKVVYRRFTEIYEFHKMLKEMFPIEAGDINPENRIIP	73
Gg-p47phox	-----------VYMFLVKWNDLSEKLIYRRFTDIYEFHKALKEMFPIESGDINAENRIIP	73
Xt-p47phox	-----------VYMFMVKWQDLTEKLVYRKFTEIYEFHKSLKEMFPIEAGDISKEHRTIP	73
Tn-p47phox	-----------VYMLLVKWSDLTEKLIYRTYPEIYTFHKALKEMFPIEAGKIEKRDRIIP	73
Tr-p47phox	-----------VYMLLVKWSDLTEKLIYRTYPEIYTFHKSLKEMFPIEAGKIEKRDRIIP	73
Ol-p47phox	-----------VYMLMVKWSDQSEKLIYRSYPEIHTFHKSLKDMFPIEAGQIEAKDRIIP	73
Dr-p47phox	-----------VYMLLVKWSDQSEKLVYRRYPEVHTLHKTLKEMFPIEAGDIDEKDRIIP	73
Mm-SH3PXD2a	-----------VYIINVTWSDSTSQTIYRRYSKFFDLQMQLLDKFPIEGGQKDPKQRIIP	73
Rn-SH3PXD2a	-----------VYIINVTWSDSTSQTIYRRYSKFFDLQMQLLDKFPIEGGQKDPKQRIIP	73
Hs-SH3PXD2a	-----------VYIINVTWSDSTSQTIYRRYSKFFDLQMQLLDKFPIEGGQKDPKQRIIP	73
Gg-SH3PXD2a	WRSLYAWGFLKVYIINVTWSDLTSQIIYRRYSKFFDLQMQLLDKFPIEGGQKDPKQRIIP	104
Xt-SH3PXD2a	-----------VYIINVTWSDSTSQVIYRRYSKFFDLQMQVLDKFPIEAGQKDPKQRIIP	73
Dr-SH3PXD2a	------------------------MAVYR--------------------GQATG------	10
Tr-SH3PXD2a1	-----------VYLINVTYSDNTSHIVYRRYSKFFDLQMQILDKFPIEGGQKDPKKRIIP	68
Tr-SH3PXD2a2	-----------VYLINVTYSDSSSHVIYRRYSKFFELQMQILDKFPIEGGQKDPKKRIIP	68
Hs-SH3PXD2b	-----------VYIIRVTWSSGSTEAIYRRYSKFFDLQMQMLDKFPMEGGQKDPKQRIIP	74
Cf-SH3PXD2b	-----------VYIIRVTWSSGSTEAIYRRYSKFFDLQMQMLDKFPMEGGQKDPKQRIIP	49
Mm-SH3PXD2b	-----------VYIIRVTWSSGATEAIYRRYSKFFDLQMQMLDKFPMEGGQKDPKQRIIP	74
Rn-SH3PXD2b	-----------VYIIRVTWSSGATEAIYRRYSKFFDLQMQMLDKFPVEGGQKDPKQRIIP	74
Gg-SH3PXD2b	-----------VYIIKVTWSNGSTEVIYRRYSKFFDLQMQMLDKFPMEGGQKDPKQRIIP	54
Dr-SH3PXD2b	-----------VYIIKVAWSDGSTEVIFRRYSKFFDLQMELLDKFPVEGGQKDPKRRIIP	73
Lg-p47phox-like	-----------VYVIFVTWSDSGTYVVYRRYSRFFDFQSALLDKFPIEGGSIDPESRIIP	94
Ci-SH3PXD2	-----------VYVIEVSWDDGSDTVIFRRYSKFFDLQISLLETFPKEGGMKDPSTRIIP	76
Ci-p47phox	-----------VYLIEIKWSDGSLCTVGRRFSAFFMMHMTLLEKFPLEGGQKDPSRRILP	73
Sp-p47phox	-----------VYIIHVTWSDGSVNVVYRRYSTFFDFQNKLLSKFPEEAGANNPSSRCIP	73
Nv-p47phox-like	-----------VYVISVTWSDGSVNVVYRRYSKFFDLQTKLLEEFPDEGGVKDPSARVLP	73

```
Mb-p47phox-like     -----------VYVITVVWSDGSEVTIYRRYSQLFEFHTSLLDRFPEAAGATGEE-RIIP 71
Hs-NOXO1            ------------FAFSVRWSDGSDTFVRRSWDEFRQLKKTLKETFPVEAGLLRRSDRVLP 71
Cf-NOXO1            ------------FAFSVCWSDGSDTFVRRSWAEFKELHKTLKEAFPVEAGLLRRSDRILP 71
Mm-NOXO1            ------------FAFSVCWSDNSDTFVRRSWDEFRQLQKTLKKTFPVEAGLLRRSEQVLP 71
Rn-NOXO1            ------------FAFSVCWSDNSDTFARRSWEEFRQLQKTLKKIFPVEAGLLQRSERVLP 71
Gg-NOXO1            ------------YMMFVSWSDQNNILIYRTLEEFKRFHKELKRKFPIESGSLRRSDRTIP 48
Xt-NOXO1            ------------------WSDHNEILIYRTFEDFKKLNRQLKKKFPLEAGLFRKSDNLLP 42
Dr-NOXO1            -----------LYMTTVLWSDGNEITVYRSLEDFKKMHRQLKKKFPPSN-PFKRSARIVP 72
Ol-NOXO1            -----------TFMVSGIWSDGSEIIIYRSFKDFKKFHDQLKKQFPNLT-PFRKEDRMLP 75
Tn-NOXO1            -----------MYMTSVLWSDHNEIVVYRTFQDFRKMH-KLKRSGKKKS-PTR------- 85
                                         *

Hs-p47phox          HLPAPK-----WFDGQRAAE---NRQGTLTEYCGTLMSLPTKISRCPHLLDFFKVRPDDL 125
Mm-p47phox          HLPAPR-----WFDGQRAAE---SRQGTLTEYFNGLMGLPVKISRCPHLLDFFKVRPDDL 125
Rn-p47phox          HLPAPR-----WYDGQRAAE---SRQGTLTEYFNSLMGLPMKISRCPHLLNFFKVRPDDL 125
Cf-p47phox          HLPAPR-----WFDGQRAAE---SRQGTLTEYYNTLMGLPVKISRCPQLLDFFRVRPDDL 125
Gg-p47phox          HLPAPK-----WFDGQRSTQ---SRQGTLAEYCYTLVNLPHKISRCRHVVSFFEVRPDDM 125
Xt-p47phox          HLPAPK-----WFDGLRSTE---NRQVTLSDYFSSLLSLPPKISRCPHVLNFFQVRSDDV 125
Tn-p47phox          SLSAPP-----WLDSQKSTE---TRQTSLSDYCQALVNLPPHISRCTHLTSLFKVRPEDE 125
Tr-p47phox          SLSAPP-----WLDSQKSTE---TRQTTLSDYCHSLVNLPPHISRCTHLTGFFTVRPEDE 125
Ol-p47phox          KLPAPR-----WLESQKSRE---NRKTTLVDYCHLLVSLPPHISRCKELSNFFKVRPEDE 125
Dr-p47phox          TLPAPK-----WLDNQKTTE---TRQATLAEYCRSLLNLPANISRCQLIRDFFKMRPEDE 125
Mm-SH3PXD2a         FLPGKI-----LFRRSHIRDVAVKRLKPIDEYCRALVRLPPHISQCDEVFRFFEARPEDV 128
Rn-SH3PXD2a         FLPGKI-----LFRRSHIRDVAVKRLKPINEYCRALVRLPPHISQCDEVFRFFEARPEDV 128
Hs-SH3PXD2a         FLPGKI-----LFRRSHIRDVAVKRLKPIDEYCRALVRLPPHISQCDEVFRFFEARPEDV 128
Gg-SH3PXD2a         FLPGKI-----LFRRSHVRDVAVKRLKPIDEYCRALVRLPPHISQCDEVFRFFEARPEDL 159
Xt-SH3PXD2a         FLPGKI-----LFRRSHIRDVAVKRLKPIDEYCRALVKLPAHISRCEEVLRFFEARPDDL 128
Dr-SH3PXD2a         --------------------------------------RWQGRVG---------------- 17
Tr-SH3PXD2a1        FLPGKI-----LFRRSHVRDVAMKRLRFIDDYCRALVRLPPQISQSEEVLRFFETKAEDI 123
Tr-SH3PXD2a2        FLPGKV-----LFRRSHIRDVAVRRLKHLDNYCKALMKLPSQISQSEEVLKFFETKLDDL 123
Hs-SH3PXD2b         FLPGKI-----LFRRSHIRDVAVKRLIPIDEYCKALIQLPPYISQCDEVLQFFETRPEDL 129
Cf-SH3PXD2b         FLPGKI-----LFRRSHIRDVAVKRLIPIDEYCKALIQLPPYISQCDEVLQFFETRPEDL 104
Mm-SH3PXD2b         FLPGKI-----LFRRSHIRDVAVKRLIPIDEYCKALIQLPPYISQCDEVLQFFETRPEDL 129
Rn-SH3PXD2b         FLPGKI-----LFRRSHIRDVAVKRLIPIDEYCKALIQLPPYISQCDEVLQFFETRPEDL 129
Gg-SH3PXD2b         FLPGKI-----LFRRSHIRDVAVKRLIPIDEYCKALIQLPPYISQCEEVLQFFETRPDDL 109
Dr-SH3PXD2b         FLPGKI-----LFRRSHIRDVAMKRLKPINEYCRALIQLPVYISQCEEVRVFFETRPEDL 128
Lg-p47phox-like     FLPGKI-----IFGRSHIRDVAVKRLGPINDYCKAVIALPPKISQCEEVLDFFEVETDDL 149
Ci-SH3PXD2          FLPGKI-----LFRRSNIRDVAMKRVLSIGEYCKDLIKLPSYIVQHSLILDFFETKPEDL 131
Ci-p47phox          FLPGKI-----LFKRSHTRDVTLKRLGSISEYCESLLLLPEHISQCDTILRFFETSSSDI 128
Sp-p47phox          FLPGKK-----LFGRSHIREVALKRLSPIDEYCTALVKLPGKISDSKEVINFFTPTPEDV 128
Nv-p47phox-like     FLPGKI-----LFGRSHVRDVAVKRKEPIQEYCSKLVELPPKISQSGLVLKFFEPKPEDI 128
Mb-p47phox-like     FLPGKK-----IFGRSHTHKVAQSRAKPIDEYLKVLISLPAELSRCDLALELFEATNTDI 126
Hs-NOXO1            KLL-----DAPLLGRVGRTSRGLARLQLLETYSRRLLATAERVARSPTITGFFAPQPLDL 126
Cf-NOXO1            KLP-----DTSLLVRGGRTGRGLARLRLLDTYTRALLAAAEQLSRSAVLTGFFEPQPVDL 126
Mm-NOXO1            KLP-----DAPLLTRRGHTGRGLVRLRLLDTYVQALLATSEHILRSSALHGFFVPKPLDL 126
Rn-NOXO1            KLPGQACRNAPLLTRRGHTGRGLLRLRLLETYVRSLLATSQHIVTSSTLNSFFAPKPLDL 131
Gg-NOXO1            RFKDING----KQKKSGKINRSLERLKLLETYTQELLKVDAKISQGEDVIQFFKAQTQDL 104
Xt-NOXO1            KLKDVP-----IFRKNRTTNRFIERLRLLEKYSQELLRTDGKISQCDLVLKFFTPSNNDL 97
Dr-NOXO1            EFKGN---------KWSGSK-SVLRMKALEEYCGQLLKSDAQVCRSSELIQFLLPKAHDL 122
Ol-NOXO1            KFNGKAR----RSLKQKGSKKSVKQMEFLESYCDKLLKCDPNVTQSSEVTRFFTPKDQDL 131
Tn-NOXO1            ---------------------SLVRLKFLQKYCNELLSCEPRVSQSADLIQFFHPNAQDL 124

                                                  bis-SH3 domains
Hs-p47phox          KLPTD-NQTKKPETYLMPKD-GKS------TATDITGPIILQTYRAIANYEKTSGSE--M 175
```

Mm-p47phox	KLPTD-SQAKKPETYLVPKD-GKN------NVADITGPIILQTYRAIADYEKSSGTE--M	175
Rn-p47phox	KLPND-SQVKKPETYLTAKD-GKN------NVADITGPIILQTYRAIADYEKGSKTE--M	175
Cf-p47phox	KLPTD-SQVKKPETYLVPKD-GKS------SVTDITGPIILQTYRAIADFEKTSSSQ--M	175
Gg-p47phox	NPVTD-SQIRKPEVFLLPKD-AKK------NTSDITGPIVLQTYRAIADYEKSSKSE--M	175
Xt-p47phox	NPVANNTNGRKPETFLLKVDTAKK------NVSDITGPIILQSYRVIADYEKNSKSE--L	177
Tn-p47phox	NPAAP-NTLKRNETFVVSRDLARG------NASEISGPIILDMYRAIADYTKTTKYE--I	176
Tr-p47phox	NPPSP-NILKRNETFVVSKDLARG------NVSEISGPIILDMYRAIADYTKTTKYE--I	176
Ol-p47phox	NPPAP-NITKRNQTFVVSKEPAQG------TAAEISGPIILDTYRVIADFEKTSKHE--L	176
Dr-p47phox	TPPAP-HPYKRNETFIMSTNRVRSN-----TTSEITGPIILETYRVIADYSKSSKYE--L	177
Mm-SH3PXD2a	NPPKEDYGSSKRKSVWLSSWAESPKKDVT-GADTNAEPMILEQYVVVSNYKKQENSE--L	185
Rn-SH3PXD2a	NPPKEDYGSSKRKSVWLSSWAESPKKDVT-GADTNAEPMILEQYVVVSNYKKQENSE--L	185
Hs-SH3PXD2a	NPPKEDYGSSKRKSVWLSSWAESPKKDVT-GADATAEPMILEQYVVVSNYKKQENSE--L	185
Gg-SH3PXD2a	NPPKEDYGSSKRKSVWMSSLSETPKKSVAPGADASSEPMILEQYVVVSNYEKQENSE--I	217
Xt-SH3PXD2a	DPPKEDYGSSKRKSVWMSSLCEIPKADPK-GADGNSEPIILDQYVVVSNYEKQENSE--I	185
Dr-SH3PXD2a	-------RGS-AGRWLTACFFPSPA-----GLDSS-EPMVLEQYVVVANYERQENSE--I	61
Tr-SH3PXD2a1	NPPVDRSRGSTAGRWLTACFSPP-------GIESS-EPMVLEQYVAVANYERQENSE--I	173
Tr-SH3PXD2a2	NPPTDRSRGPMAGSWLTACFFPTGP-----GLDAS-DPMLLEQYVVVASYEKQEPAE--I	175
Hs-SH3PXD2b	NPPKEEHIGKKKSGGDQT----------------SVDPMVLEQYVVVANYQKQESSE--I	171
Cf-SH3PXD2b	NPPKEEHVGKKKSGGDLT----------------SVDPMVLEQYVVVADYQKQESSE--I	146
Mm-SH3PXD2b	NPPKEEHIGKKKSGNDPT----------------SVDPMVLEQYVVVADYQKQESSE--I	171
Rn-SH3PXD2b	NPPKEEHIGKKKSGSDPT----------------SVDPMVLEQYVVVANYQKQESSE--I	171
Gg-SH3PXD2b	TPPKEEPIGKKKSGADSA----------------SVDPLVLEQYVVVANYQKQESSE--I	151
Dr-SH3PXD2b	NPPKEEPSGKKKSGGDSS----------------SADPLLLDQYVAVTDYEKQESSE--I	170
Lg-p47phox-like	DPPKAEEKKKKDEGAKKAEN--------------ISDPKTLTQYRVTCDYEKQDRGE--I	193
Ci-SH3PXD2	K-PPESESTSRASKRNSLD---------------ISQPILPESYVVVQDYIKTQPKE--L	173
Ci-p47phox	ARDTKEKTQSTAVSQIIQD---------------ITGPIELETYIAIADYKAEAKTQ--I	171
Sp-p47phox	SPPSPDGGGESTRGRADIGN--------------ISEPIQAEQYIVVADYKKQQKNE--V	172
Nv-p47phox-like	ELPLEK--KKKKKKTADV----------------ISDPVSLEQYVAIADYQKQNRNE--I	168
Mb-p47phox-like	APPSEQERERRPTGIFKKLSKSDDSGKEE-REISVGDVMVLDQYRAVADYTKQDRKE--L	183
Hs-NOXO1	EPALPPGSRVILPTPEEQPLSRA-------AGRLSIHSLEAQSLRCLQPFCTQDTRDRPF	179
Cf-NOXO1	EPVLPPGSLVILPTPEE-PHRRP-------PHSPAICSLEAQSLRCLQPFSTQDTQGWPF	178
Mm-NOXO1	EPMLPPGSLVILPTPEE-PLSQP-------RGSLDIHSLEAQSIPCVQPFHTLDIRDRPF	178
Rn-NOXO1	EPMLPPGSLVILPTPEE-PLSQP-------IGSLAIHSLEAQSMRCLQPFHTLDTKDRPF	183
Gg-NOXO1	DPCFPEDSVVIMPSEIGGEKKKEVQ----QQQLSITYPQVSQSYRCIETFETKDTKNKTF	160
Xt-NOXO1	NPKFPENSLVMMTSDSKDQKEQKKP----LPEAPAIHPIVSQQYICMEDYETKDTKNRPF	153
Dr-NOXO1	NADFAKNCIVIMPSDVTLGSSKA------ESNSGVTQPFVTETYRCIANYETKDTKNRPF	176
Ol-NOXO1	QPDFTKNSSLLLVYGR-PGALRS------SGAGNVTHPFVTQTYRCVAPYETRDTKNRPF	184
Tn-NOXO1	EPEFSKNRQEEVKAEAGHGSVG-----------NVTQPFVTVTYRCVSQYETKDTKNKPF	173

Hs-p47phox	ALSTGDVVEVVEKSESGWWFCQMKA-KRGWIPASFLEPLDSPDETEDPEPNYAG------	228
Mm-p47phox	TVATGDVVDVVEKSESGWWFCQMKT-KRGWVPASYLEPLDSPDEAEDPDPNYAG------	228
Rn-p47phox	TVATGDVVDVVEKSESGWWFCQMKT-KRGWVPASYLEPLDSPDEAEDPDPNYAG------	228
Cf-p47phox	ALATGDVVDVVEKSESGWWFCQTKT-KRGWVPASYLEPLDSPDEAEDPEPNYEG------	228
Gg-p47phox	AVKAGDAVDVVEKSETGWWFCQLKT-KRGWVPAAYLEPMDGPDESEEQEPNYAG------	228
Xt-p47phox	AAKNGDVVEIVEKSENGWWFCQLRN-KRGWMPAAYLEPLDGPDESEEQDPNYEG------	230
Tn-p47phox	NLLAGDQVEIVEKNQNGWWFCQMDS-KRGWVPASYLEPLDGPEESEEADPDYEGS-----	230
Tr-p47phox	NLHAGDQVEIVEKNQNGWWFCQCDS-KRGWVPASYLEPLDGPEESEEAEPDYGGSP----	231
Ol-p47phox	NLHDGDLVEILEKNSNGWWFCQCEA-KRGWVPASYLEPLDGPEEAEEAEPDYEG------	229
Dr-p47phox	TLKMGDMVDIVEKSPNGWWFCQCES-RRGWVPASYLEPLDGADESEEPEPNYAG------	230
Mm-SH3PXD2a	SLQAGEVVDVIEKNESGWWFVSTSE-EQGWVPATYLEAQNGTRDDSDINTSKTGEVSKRR	244
Rn-SH3PXD2a	SLQAGEVVDVIEKNESGWWFVSTSE-EQGWVPATYLEAQNGTRDDSDINTSKTGEVSKRR	244
Hs-SH3PXD2a	SLQAGEVVDVIEKNESGWWFVSTSE-EQGWVPATYLEAQNGTRDDSDINTSKTGE-----	239
Gg-SH3PXD2a	SLQAGEVVDVIEKNESGWWFVSTAE-EQGWVPATYLESQNGTRDDSDINTSKTGE-----	271

```
Xt-SH3PXD2a         SLKTGELVDVIEKNESGWWFVSTTE-EQGWVPATYLDSQGGTKDDSEINTSKSGDVTKRR 244
Dr-SH3PXD2a         SLKAGETVDVIEKSESGWWFVSTAE-EQGWVPATYLDSQSGTRDDLDLGTSRSGEVTKRR 120
Tr-SH3PXD2a1        NLKAGETVDVIEKSESGWWFVSTAE-EQGWVPATYLDSQNATRDDLDLGTFRTGEVTKRR 232
Tr-SH3PXD2a2        SLQAGEVVDVIEKSESGWWFVSTAE-EQGWVPATYLNSHSGTRDDLDLGASKAGEVTKRH 234
Hs-SH3PXD2b         SLSVGQVVDIIEKNESGWWFVSTAE-EQGWVPATCLEGQDGVQDEFSLQPE--------- 221
Cf-SH3PXD2b         SLSVGQVVDIIEKNESGWWFVSTAE-EQGWVPATCLEGQDGMQDEFSLQPE--------- 196
Mm-SH3PXD2b         SLSVGQVVDIIEKNESGWWFVSTAE-EQGWVPATCLEGQDGVQDEFSLQPE--------- 221
Rn-SH3PXD2b         SLSVGQVVDIIEKNESGWWFVSTAE-EQGWVPATCLEGQDGVQDEFSLQPE--------- 221
Gg-SH3PXD2b         SLCVGQLVDIIEKNESGWWFVSTSE-EQGWVPATCLEAQDGVQDELSMQPD--------- 201
Dr-SH3PXD2b         SLYVGQVVEVIEKNESGWWFVSTED-AQGWVPATCLEAQD-DPDDFSLPAE--------- 219
Lg-p47phox-like     DLEAGMIVEVAEKSETGWWFVNSDD-AQGWVPSTYLEPADGSTTDNVVLRAQPG------ 246
Ci-SH3PXD2          NARVGEVVEVMDKHENGWWFVSTEDGEQGWVPGVYLGKPDGKSENLVIKQDQLG------ 227
Ci-p47phox          SLHSGETVEVVEKSESGWWLVCNTYGSNGWVPGAYLEKEDGSEEDLVTEKAAVG------ 225
Sp-p47phox          ELTAGDLVEVFEKNDNGWWFVTVHD-QHGWAPGTFLQNPDGQEEEDEETLIPG------- 224
Nv-p47phox-like     TMVAGDIVEVIDKNENGWWFVNLDE-EQGWVPAAYLESVDGHSDDAAPIEGPT------- 220
Mb-p47phox-like     SFKTGDIFEVVEKNDNGWWFVSNDS----------------------------------- 208
Hs-NOXO1            QAQAQESLDVLLRHPSGWWLVENEDRQTAWFPAPYLEE---AAPGQGREGGPSLGS---- 232
Cf-NOXO1            HARAQEVIDVLLRHPSGWWLVANEEQQMAWFPAPYLEE---AAP--DREG-TTLRS---- 228
Mm-NOXO1            HTKAQEILDILLRHPSGWWLVENKDQQVAWFPAPYLEE---VATCQGQESGLALQG---- 231
Rn-NOXO1            HTQAKEILDILLRHPSGWWLVENKDQQTAWFPAPYLEE---IATGQGQESGMAVQG---- 236
Gg-NOXO1            KVAKKEIVEVLLKDMTGWWLVENADKQIAWFPASYLEQ---ISAHKDIQNVESSDE---- 213
Xt-NOXO1            KVKRHELVGVLIKENTGWWLVENEEKHLAWFPAPYLKD---VDNSEDTDSG-TSED---- 205
Dr-NOXO1            KVEVDETVDVLIKDQKGWWLVENESKHLAWFPAPYLERAEMADDGPDEMDNESFQS---- 232
Ol-NOXO1            KVAVDEKLDVLIKDPAGWWLVESENKRLAWFPAPYLE----VLDGEDEDDEGNLG----- 235
Tn-NOXO1            KVAADEKVDVLIKDKAGWWLVENEEKRMAWFPAPYLEK---LEEDGDEDDTDGTR----- 225
                                    ***

Hs-p47phox          ------------------------EPYVAIKAYTAVEGDEVSLLEGEAVEVIHKLLDGWW 264
Mm-p47phox          ------------------------EPYVTIKAYAAVEEDEMSLSEGEAIEVIHKLLDGWW 264
Rn-p47phox          ------------------------EPYVTIKAYAAVEEDEVSLSEGEAIEVIHKLLDGWW 264
Cf-p47phox          ------------------------EPYVTIKAYTAEMEDEMSLQEGEAIEVIHKLLDGWW 264
Gg-p47phox          ------------------------ELYVVQKSYTAVEEDELTLKEGDTIEVIHKLLDGWW 264
Xt-p47phox          ------------------------DLHITTKDYSGELDDELSLQEGENVEVIHKLLDGWW 266
Tn-p47phox          ------------------------ELFITIKAYKAEQEDEISLDLGESIEVIHKLLDGWW 266
Tr-p47phox          -----------------------CELYITIKAYKAEQEDEITLDLGESIEVIHKLLDGWW 268
Ol-p47phox          ------------------------ELHVTTNAYKAEQDDEISLDLGETVEVIHKLLDGWW 265
Dr-p47phox          ------------------------ELYKTTRGYKAVEQDEMTLEAGVIIEVIHKLLDGWW 266
Mm-SH3PXD2a         KAHLRRLDRRWTLGGMVNRQHSREEKYVTVQPYTSQSKDEIGFEKGVTVEVIRKNLEGWW 304
Rn-SH3PXD2a         KAHLRRLDRRWTLGGMVNRQHSREEKYVTVQPYTSQSKDEIGFEKGVTVEVIRKNLEGWW 304
Hs-SH3PXD2a         -----------------------EEKYVTVQPYTSQSKDEIGFEKGVTVEVIRKNLEGWW 276
Gg-SH3PXD2a         -----------------------EEKYVTIQPYASQGKDEIGFEKGVTVEVIQKNLEGWW 308
Xt-SH3PXD2a         KAHLRRLDRRWTLGGIVNRQQSREEKYITVQPYTSQGKDEIGFEKGDTVEVIQKNLEGWW 304
Dr-SH3PXD2a         KAHLKRLDRRWTLGGIVNRQQSREEKYVSVQAYASQGKDEIGFEKGVTVEVIQKNLEGWW 180
Tr-SH3PXD2a1        KAHLKRLDRRWTLGGIVNRQQSREEKYVTLQPYTSQGKDEVTFEKGVIVEVIQKNLEGWW 292
Tr-SH3PXD2a2        KAHLKRLDRRWTLGGVISRQQSREEKYVTVQSYTSEGKDEIAFEKGAVVEVIQKNLEGWW 294
Hs-SH3PXD2b         ----------------------EEEKYTVIYPYTARDQDEMNLERGAVVEVIQKNLEGWW 259
Cf-SH3PXD2b         ----------------------EEEKYTVIYPYTARDQDEMNLERGAVVEVIQKNLEGWW 234
Mm-SH3PXD2b         ----------------------EEEKYTVIYPYTARDQDEMNLERGAVVEVVQKNLEGWW 259
Rn-SH3PXD2b         ----------------------EEEKYTVIYPYTARDQDEMNLERGVVVEVIQKNLEGWW 259
Gg-SH3PXD2b         ----------------------EEEKYTVIYPYTARDQDEMNLDKGAVVVVIQKNLEGWW 239
Dr-SH3PXD2b         ----------------------EEEKYTAIYPYSARDQDEIDLERGMTVEVIQKNLEGWW 257
Lg-p47phox-like     ----------------------QEEKFICIEMFQSNGNDEISLEKGAVVEVLEKNLSGWW 284
Ci-SH3PXD2          ----------------------QGELYLTTTQYNGE-DSEVSFNTGVLVEVLQKNLEGWW 264
Ci-p47phox          ----------------------QGTWYVATSHYDATSNDEISFPMGAALEVLQVNLEGWW 263
```

```
Sp-p47phox          ---------------------NDE-SYITNNAYQGQAEDEISFETGVVVTVIQKSLDGWW 262
Nv-p47phox-like     ---------------------VEVGQYITTTSHKAELDDEITFETGVIVSVIQKNFDGWW 259
Mb-p47phox-like     --------------------ASQDEKYITTAAYAASSDDEIGYEKGVVVRVLEKKLDGWW 248
Hs-NOXO1            ----------------------SGPQFCASRAYESSRADELSVPAGARVRVLETSDRGWW 270
Cf-NOXO1            ----------------------SGSQFCASQAYESSHADELSVPAGARVSVLETSDRGWW 266
Mm-NOXO1            ----------------------SGRQFCTTQAYEGSRSDELSVPSGARVHVLETSDRGWW 269
Rn-NOXO1            ----------------------SGRQFCATQAYEGSRPDELSVPSGARVHVLETSDRGWW 274
Gg-NOXO1            ----------------------EGSLYFVMRAYEAQKADELSLNKGVVVEVVRRSDNGWW 251
Xt-NOXO1            ----------------------EGVLYYAAKAYEAMNSDEVSITVGVLVEVIEKSNNGWW 243
Dr-NOXO1            ----------------------AGVFYVATKAYKATNSDELSVELGSVLEVLQKSDNGWW 270
Ol-NOXO1            -----------------------GSLYCAVRSYSTKKNDEVPLSIGSVVEVLRKSDDGWW 272
Tn-NOXO1            ------------------------TLYLTAKNYKASKGDEISVAVGAVVEVLQKSDSGWW 261
                                                           *     *    *      ***

Hs-p47phox          VIRKDDVTGYFPSMYLQKSGQ--DVSQAQRQIK-RGAPPRRSSIRNVHSIHQRSRKRLSQ 321 -36
Mm-p47phox          VVRKGDITGYFPSMYLQKAGE--EITQAQRQIRGRGAPPRRSTIRNAQSIHQRSRKRLSQ 322 -37
Rn-p47phox          VVRKGDITGYFPSMYLQKAGE--EITQAQRQIRSRGAPPRRSTIRNAQSIHQRSRKRLSQ 322 -37
Cf-p47phox          VVRKDDITGYFPSMYLQKSGQ--DAAQAHRQIKSRGAPPRRSSIRNAHSIHQRSRKRLSQ 322 -37
Gg-p47phox          VIRKDETTGYYPSMYLQKSGE--VNSPEKSGLRNHNIPPRRSTIRNAKSIHNKGRKQISQ 322 -37
Xt-p47phox          VVRKGSITGYFPAMYLQKSGE--TAPANENPSKRKGLPPRRSTISNANSIHKKERKQISQ 324 -37
Tn-p47phox          VVRKGEGTGYFPSMFLQKASKRAQAEAARNHLQGQKPPPRRSTIRNAKSIHNKSRQRLSQ 326 -39
Tr-p47phox          VVRKGEQMGYFPSMFLQKANKREQSESSRANVQGHKPPPRRSTIRNAKSIHNKSRQRLSQ 328 -39
Ol-p47phox          VVRKGDETGHFPSMFLTKASKRIQT--LRTNLHGQRPPPRRSTIKNAKSIHNRSRQRLSQ 323 -37
Dr-p47phox          VVRKGEETGFYPSMFLCRTGEKKEVDAERDVVRRATPPPRRSTIRNAQSIHSTVRRRISQ 326 -39
Mm-SH3PXD2a         YIRYLGKEGWAPASYLKKAKDDLPT--RKKNLAGPVEIIGNIMEISNLLNKKASGDKEAP 362 -37
Rn-SH3PXD2a         YIRYLGKEGWAPASYLKKAKDDLPT--RKKNLAGPVEIIGSIMEISNLLNKKASGDKEAP 362 -37
Hs-SH3PXD2a         YIRYLGKEGWAPASYLKKAKDDLPT--RKKNLAGPVEIIGNIMEISNLLNKKASGDKETP 334 -
Gg-SH3PXD2a         YIRYLGKEGWAPASYLKKAKDDLPS--RKKNLTGPVEIIGNIMEISNLLNKKST-DKETQ 365
Xt-SH3PXD2a         FIKYQGKEGWAPASYLKKAKDDIPC--RKKNLTGPVEIIGNIMEISNLLNKKTPNDKESQ 362
Dr-SH3PXD2a         YIRYQGKEGWAPASYLKKLKDDLSP--RKKTLTGPVEIIGNIMEISNLLNKKAVSEKDIQ 238
Tr-SH3PXD2a1        FIRYLGKEGWAPASYLKKVKDDFSP--RKKTMTGPVEIIGNIMEISNLLQKKSSSEKDIQ 350
Tr-SH3PXD2a2        FIRYQDKEGWAPASYLKKMKDDLSP--RKKAVTGPVEIIGNIMEISNLLNKKALSEKDVQ 352
Hs-SH3PXD2b         KIRYQGKEGWAPASYLKKNSGEPLP--PKPGPGSPSHPGALDLDGVSRQQNAVGREKELL 317
Cf-SH3PXD2b         KIRYQGKEGWAPASYLKKSSGEPLP--PKPGTGSPAHTGILDLDGLSRQQSSVGRDRELL 292
Mm-SH3PXD2b         KIRYQGKEGWAPASYLKKNSGEPLP--PKLGPSSPAHSGALDLDGVSRHQNAMGREKELL 317
Rn-SH3PXD2b         KIRFQGKEGWAPASYLKKSSGEPLP--PKLGPSS-AHSGALDLDGVSRQQNAVGREKELL 316
Gg-SH3PXD2b         KIRYQGQEGWAPASYLKKGNGEMFS--QKLGSGSSAHSCALDLDGISRQQAVTSREK--- 294
Dr-SH3PXD2b         KIRYQGKEGWAPASYLKK--ADILS--QKMAAGAPVHASTNDLDVACKQQNANKENKE-- 311
Lg-p47phox-like     LVRHQGKEGFAPATYLNKTEDQFAVS-LAKRKSLDVEIITNLSDISNIMKGDSPRNSLAS 343
Ci-SH3PXD2          FVSYNGKQGWAPASYLTKPPESVTAISLSKKLSSPVKNDSSIPSSHSCTSLSSQSSSNAG 324
Ci-p47phox          LARYNSNEGWVPGSYLEK----------SRRTYS-WATDTAPTESVPGAAESVKKSTLA- 311
Sp-p47phox          KVSYQGKQGWAPATFLQIYKGPSGV--TPKHPTQSIGNVMLLKSGSDSKPKPGPGPGSSG 320
Nv-p47phox-like     LIRYQDKEGWAPAMYLRKPD-PS-----QLHAAQAVG-------GLPSKDRVG-AVTAPA 305
Mb-p47phox-like     QVEYQGKVGWTPGTFLKRIENSNAGPTTLKSTPASSTAATNGGVVPPAKPPAPQLRNEKE 308
Hs-NOXO1            LCRYGDRAGLLPAVLLRPE----------------------------------------- 289
Cf-NOXO1            LCRFRGRSGLLPAVLLQPE----------------------------------------- 285
Mm-NOXO1            LCRYNGRTGLLPAMSLQPE----------------------------------------- 288
Rn-NOXO1            LCRYNGQTGLLPAVLLQPE----------------------------------------- 293
Gg-NOXO1            LIRYNGRKGYMPSMCLQAYKNPHHRLQT-------IMNSGLHISTPNLCSPSPALQP--L 302
Xt-NOXO1            LIR--------------------------------------------------------- 246
Dr-NOXO1            IVRYNRKAGYVPSMYLQPHNNPRILLKS--------TQKEISRSTLDLAQLQHPQT---- 318
Ol-NOXO1            LIRFNGKVGYIPAMYLQPYNNPRTGLHG--------QHNKIHTSTLNLSTMKDQQVPPSI 324
Tn-NOXO1            LIRYQGKVGYVPTLCLQPYNRPQVRLNGAAFPGLQQQSNKLSSSRGNLLQLPSAGRSPSP 321
```

```
Mm-p47phox          ------------------------------------------------------------
Rn-p47phox          ------------------------------------------------------------
Cf-p47phox          ------------------------------------------------------------
Hs-p47phox          ------------------------------------------------------------
Gg-p47phox          ------------------------------------------------------------
Xt-p47phox          ------------------------------------------------------------
Tn-p47phox          ------------------------------------------------------------
Tr-p47phox          ------------------------------------------------------------
Ol-p47phox          ------------------------------------------------------------
Dr-p47phox          ------------------------------------------------------------
Mm-SH3PXD2a         ---AEGEGSEAPITKKEISLPILCNASNG--------------------SALAIPERTTS 399
Rn-SH3PXD2a         ---VEGEGSEAPISKKEISLPILCNASNG--------------------SASGIPERTTS 399
Hs-SH3PXD2a         P--AEGEGHEAPIAKKEISLPILCNASNG--------------------SAVGVPDRTVS 372
Gg-SH3PXD2a         ---AENEASETHITKKEISLPILCNDSNG--------------------NAMMTPDKQAS 402
Xt-SH3PXD2a         ---GGNDSEEQTITKKEISLPILCNDSNG--------------------NSMMSPEKQIP 399
Dr-SH3PXD2a         TDGEA-TTPERHISKSEISLPMPYAPEAGVAP--TVVTALGMNS-----GSSATLQENKS 290
Tr-SH3PXD2a1        TDGEGSTTPERHTSKNEISVPTPFSSEINAETGRRLSTTRDTNSPCLGIAASAALSENKA 410
Tr-SH3PXD2a2        TEGVP-ESPQ--AARKEISLPIPCAESS----------------------PASNPQEEKS 387
Hs-SH3PXD2b         --------------------------------------------------SSQRDGRFEG 327
Cf-SH3PXD2b         --------------------------------------------------NNQRDGRFEG 302
Mm-SH3PXD2b         --------------------------------------------------NNQRDGRFEG 327
Rn-SH3PXD2b         --------------------------------------------------NNQRDGRFEG 326
Gg-SH3PXD2b         ------------------------------------------------------DGRFDN 300
Dr-SH3PXD2b         --------------------------------------------------------NQRD 315
Lg-p47phox-like     ------------------------------------------------------TETPKP 349
Ci-SH3PXD2          ------------------------------------------------------------
Ci-p47phox          ------------------------------------------------------------
Sp-p47phox          --------------------------------------------------------RPQP 324
Nv-p47phox-like     --------------------------------------------------------AVRP 309
Mb-p47phox-like     ----------------------------------------------------PPPRRESI 316
Hs-NOXO1            ------------------------------------------------------------
Cf-NOXO1            ------------------------------------------------------------
Mm-NOXO1            ------------------------------------------------------------
Rn-NOXO1            ------------------------------------------------------------
Gg-NOXO1            R----------------------------------------------------------- 303
Xt-NOXO1            ------------------------------------------------------------
Dr-NOXO1            ------------------------------------------------------------
Ol-NOXO1            P----------------------------------------------------------- 325
Tn-NOXO1            Q----------------------------------------------------------- 322


Mm-p47phox          -------------------DTYRRNSVRFLQQRR-------RPGRPGPQSTDG-TKDN-- 353
Rn-p47phox          -------------------DTYRRNSVRFLQQRR-------RPARPGPQSPD--SKDN-- 352
Cf-p47phox          -------------------DTYRRNSV---------------PTQPG------------- 335
Hs-p47phox          -------------------DAYRRNSVRFLQQRR-------RQARPGPQSPGSPLEEE-- 353
Gg-p47phox          -------------------ETYRRNSKKYMQNRR-------NMRGNLQNKDIISEKNE-- 354
Xt-p47phox          -------------------DTYRRNSKKYLKQR---------QSIVDTKSPIITEENK-- 354
Tn-p47phox          -------------------DAYRRNSRRYLQQKGGQRDLQNKYARTAAKSPLQERKNQGN 367
Tr-p47phox          -------------------DTYRRNSRRYLQQKGGQLVKPDPYPRNVAKSPLRERRNQGN 369
Ol-p47phox          -------------------EAYRRNSRRYLQQKGGRLASPHRSSRGSGKSPLTERKNHDN 364
Dr-p47phox          -------------------DSYRKQSRRFLQQRG----RLNSHSRIGTRSPLQERRTNKN 363
Mm-SH3PXD2a         KLAQGSPAVARIAPQRAQISSPNLRTRPPPRRESSL--GFQLPKPPEPPSVEVEYYTIAE 457
Rn-SH3PXD2a         KLAQGSPAVARIAPQRAQISSPNLRTRPPPRRESSL--GFQLPKPPEPPSVEVEYYTIAE 457
```

```
Hs-SH3PXD2a       RLAQGSPAVARIAPQRAQISSPNLRTRPPPRRESSL--GFQLPKPPEPPSVEVEYYTIAE 430  (3rd SH3)
Gg-SH3PXD2a       KLAQGSPAIARIAPQRAQISSPNLRTRPPPRRESSL--GFQLPKPPEPPSVEVEYYTIAE 460
Xt-SH3PXD2a       KVTPGSPAVARIAPQRSEISSPNLRTKPPPRRESSL--GFQLPKPPEPPSVEVEYYTIAE 457
Dr-SH3PXD2a       RAEPGSPAIARVAPHRVEIGSPNLRQKPPPRRDANL--AFQLPKPPEAPTVEAEYYTIAE 348
Tr-SH3PXD2a1      RGDPGSPAVARVAPHRVEIGSPNLRQKPPPRRDTNL--GFHLPKPPEPPAVEAEYYTIAD 468
Tr-SH3PXD2a2      KVEPASPAVARIAPHRVEIGSPVLRQKPPPRRDATL--GFQLPSPPEPPTVEAEYYTIAE 445
Hs-SH3PXD2b       RPVPDG---------DAKQRSPKMRQRPPPRRDMTIPRGLNLPKPPIPPQVEEEYYTIAE 378  (3rd SH3)
Cf-SH3PXD2b       RPVPDG---------DIKQRSPKMRQRPPPRRDMTIPRGLHLPKPPVPPQVEEEYYTIAE 353
Mm-SH3PXD2b       RLVPDG---------DVKQRSPKMRQRPPPRRDMTIPRGLNLPKPPIPPQVEEEYYTIAE 378
Rn-SH3PXD2b       RLAPDG---------DVKQRSPKMRQRPPPRRDMTIPRGLNLPKPPIPPQVEEEYYTIAE 377
Gg-SH3PXD2b       RPLPNA---------DIRRKSPKMRQRPPPRRDLTIPRGLNLPKPPVPPQVEEEYYTIAD 351
Dr-SH3PXD2b       RFSPFS---------DSKRKVG-ARQRPPPRRDLTIPRGVNLPKPPVPPQVEEEYYTIAD 365
Lg-p47phox-like   VTEMYS-------KVKSRSLERGGNIKPPPRQSMRM---VSLSFTPSPSHKTSHYVTIAE 399
Ci-SH3PXD2        -----------------DTSSPSHKRLSGVGIKRPSLKPQVPPPPPPQHSDDVQYIAMHS 367
Ci-p47phox        ------------------LVKPPPKRAT---IRRTLKVTRGQDSIKEKHDEDNLYITLFD 350
Sp-p47phox        PVQPRDEPGQLYSNYDAEVKPTPPRRATVKKSVRRG---GVRQTKPKLKK-VMEHYTTDS 380
Nv-p47phox-like   GVNPVTDRCKKTINFKAETETVVTRKRKAHSVNVYG---KGIEAMPALKSPDAEYFTVGE 366
Mb-p47phox-like   RRPVSIHSKGPFAEIERARQIAAQNSGTKPAKPAVPALPKRNAASTNSGNDAVTIIYTCTR 376
Hs-NOXO1          ------------------------GLGALLSGTGFR----GGDDPAGEARGF-------- 313
Cf-NOXO1          ------------------------GLGALLSGPGLHREANSKEDRGGEAQRT-------- 313
Mm-NOXO1          ------------------------GLGSLLGRPGFP-------DSAGADK---------- 307
Rn-NOXO1          ------------------------GLGSLLGRPGLP-------DSGGADK---------- 312
Gg-NOXO1          -------------DSTARDCTSGDGSDEDLESDSSSLSSGSAPSGVLSWKPD-------- 342
Xt-NOXO1          ------------------------------------------------------------
Dr-NOXO1          -------------LQDS-------RLRELSRSQGNLLLQPAETD---IMDKQ-------- 347
Ol-NOXO1          -------------RRDSPSSDSSTSLREEAQGDAPRLQEDSQSSGEAASDRR-------- 364
Tn-NOXO1          -------------QPHADGRQRSHSLNALLETLPAQPARGAAPDTGTPPSPQQAPPPVIR 369


Mm-p47phox        ---------------------------PSTPRVKPQPAVPPRPSSDLILHRCTESTKRKL 386
Rn-p47phox        ---------------------------PSTPRAKPQPAVPPRPSSDLILHRCTESTKRKL 385
Cf-p47phox        ---------------------------------KPQPAVPPRPSADLILHRCSESTKRKL 362
Hs-p47phox        ---------------------------RQTQRSKPQPAVPPRPSADLILNRCSESTKRKL 386
Gg-p47phox        ---------------------------QEENKSKAQPAVPPRPSKDLIMNRCTESTRRKI 387
Xt-p47phox        ---------------------------EEESKSKPQPAIPPRPSKELILDRCSENTKSKI 387
Tn-p47phox        I-------------------PEESGTASEGEPKKEAPVVPPRPSPELILQRCSDNTRKKI 408
Tr-p47phox        I-------------------PEESSTISENEGKREAPVIPPRPSPELILQRCSDNTRKKI 410
Ol-p47phox        I-------------------PELHG--SETETKRETPVIPPRPSPELILERCTANTCKKV 403
Dr-p47phox        I-------------------EKSSAPQAEDEDK-SVPVVPPRPSPQLILERCTENTSKRM 403
Mm-SH3PXD2a       F-------------------QSCISDG-ISFRGGQKAEVIDKNSGGWWYVQIGEKEGWAP 497
Rn-SH3PXD2a       F-------------------QSCISDG-ISFRGGQKAEVIDKNSGGWWYVQIGEKEGWAP 497
Hs-SH3PXD2a       F-------------------QSCISDG-ISFRGGQKAEVIDKNSGGWWYVQIGEKEGWAP 470
Gg-SH3PXD2a       F-------------------QSCISDG-ISFRGGQKAEVIEKNSGGWWYVQIGEKEGWAP 500
Xt-SH3PXD2a       F-------------------QSCISDG-ISFRGGQKAEVIEKNSGGWWFVQIGEKEGWAP 497
Dr-SH3PXD2a       F-------------------QSSISDG-ISFRGGQKADVIEKNSGGWWYVQIGDTEGWAP 388
Tr-SH3PXD2a1      F-------------------QSSISDG-ISFRGGQKADVIEKNPGGWWYVQIGEKEGWAP 508
Tr-SH3PXD2a2      F-------------------QSCISDG-ITFSGGQKAEVIEKNSGGWWYVQIGEKEGWAP 485
Hs-SH3PXD2b       F-------------------QTTIPDG-ISFQAGLKVEVIEKNLSGWWYIQIEDKEGWAP 418
Cf-SH3PXD2b       F-------------------QTTIPDG-ISFQAGLKVEVIEKNLSGWWYIQIEDKEGWAP 393
Mm-SH3PXD2b       F-------------------QTTIPDG-ISFQAGLKVEVIEKSLSGWWYIQMEDKEGWAP 418
Rn-SH3PXD2b       F-------------------QTTIPDG-ISFQAGLKVEVIEKSLSGWWYIQMEDKEGWAP 417
Gg-SH3PXD2b       F-------------------QTTIPDG-ISFQAGMKVEVIEKNLSGWWYIQIEEKEGWAP 391
Dr-SH3PXD2b       F-------------------QTTIPDG-ISFQAGLKVEVIEKNSSGWWYIQIEDKEGWAP 405
Lg-p47phox-like   F-------------------EDTVGDG-LSFKEGQSVEIVEESDSGWWMVKLNGKSGWVP 439
```

```
Ci-SH3PXD2          F-------------------DGKIPHG-VAFNIDDPVTVLSKSP-GWWYVEVNGNEGWAP 406
Ci-p47phox          F-------------------DSSIEDG-LSFKAGQIVKVIEQSDNGWWLATLNGAEGWVP 390
Sp-p47phox          F-------------------QGAAGEGSISFESGQKVEVLEENDGGWWYVKMNGQEGWAP 421
Nv-p47phox-like     F-------------------RALGIQSGLDFSKGASVEVLDKNPNGWWYGKIDGNEGWIP 407
Mb-p47phox-like     K-------------------CDKQDDSGIALPAGARVELLEKSETGWYYVKYAGREGWAP 417
Hs-NOXO1            ----------------------PEPSQATAPPP----TVPTRPSPGAIQSRCCTVTRRAL 347
Cf-NOXO1            ----------------------PEACQATTLSP----SVPARPPLSAIRSRCCSVTRRAL 347
Mm-NOXO1            ------------------------VAEDRTIPP----VVPTRPCMSAIQSRCCSITRRAL 339
Rn-NOXO1            ------------------------VTEGRTVPP----VVPTRPCMSAIQSRCCSITRRAV 344
Gg-NOXO1            --------------------LSRSLPEVEQAVP-------MRPSAHEILQRCSTVTKRAV 375
Xt-NOXO1            ------------------------------------------------------------
Dr-NOXO1            --------------------KSDPCLNKMPSTP----KVPPRPAVQEILTRCTTVTRKNM 383
Ol-NOXO1            --------------------STNSSGFDESTGP----RVPPRPKAEEILTRCTTMTRKAA 400
Tn-NOXO1            PQYPRGQNDAQRLRSQHLQGSRNPQGSTQTPGPGDPHQVHHHHPQECQQRRSVAHPTRDT 429

Mm-p47phox          TSAV-------------------------------------------------------- 390
Rn-p47phox          TSAV-------------------------------------------------------- 389
Cf-p47phox          ASSV-------------------------------------------------------- 366
Hs-p47phox          ASAV-------------------------------------------------------- 390
Gg-p47phox          ------------------------------------------------------------
Xt-p47phox          ------------------------------------------------------------
Tn-p47phox          S----------------------------------------------------------- 409
Tr-p47phox          SIHKSSSASTSKPGQA-------------------------------------------- 426
Ol-p47phox          SIHRSQSGSSSQNQDQ-------------------------------------------- 419
Dr-p47phox          SMQEA------------------------------------------------------- 408
Mm-SH3PXD2a         ASYIDKRKKPNLSRRTSTLTRPKVPPPAPPSKPKEAEENPVGAC----ESQGS---PLKV 550
Rn-SH3PXD2a         ASYIDKRKKPNLSRRTSTLTRPKVPPPAPPGKPKETEENPAGAC----ENQGS---PLKL 550
Hs-SH3PXD2a         ASYIDKRKKPNLSRRTSTLTRPKVPPPAPPSKPKEAEEGPTGAS----ESQDS---PRKL 523
Gg-SH3PXD2a         ASYIDKRKKPNLNRRTSTLTRPKVPPPAPPSKPKDSEEGTTGMVSS-GDTQDS---PHKL 556
Xt-SH3PXD2a         SSYIDKRKKPNLNRRTSTLTRPKVPPPAPPVKQKDSEDGINPLTCASTEPKDS---PSKQ 554
Dr-SH3PXD2a         SSYIDKRKKPNLSRRTSTLTRPKVPPPAPPVKKQDSEEGPSLGGSA-SKAPES---PQR- 443
Tr-SH3PXD2a1        SSYIDKRKKPNLSRRSSTLTRPKVPPPAPPVKKQDSEEAPTSDNLP-FKASDP---PSRP 564
Tr-SH3PXD2a2        CSYIDKRKKPNLNRRTSTLCRPKVPPPAPPVKKQDSVETAPPSSPG-SEAPESPVYQGRP 544
Hs-SH3PXD2b         ATFIDKYKK------TSNASRPNFLAPLPHEVTQLRLGEAAALENNTGSEATG---PSRP 469
Cf-SH3PXD2b         ATFIDKYKK------TSNASRPNFLAPLPNEVTQLHLGDAAAMENNTGSEAIG---PSRP 444
Mm-SH3PXD2b         ATFIDKYKK------TSSASRPNFLAPLPHEMTQLRLGDAAATENNTGPEAVG---PSRP 469
Rn-SH3PXD2b         ATFIDKYKK------TSSASRPNFLAPLPHEMTQLRLGEAAATENNTGPEAVG---PSRP 468
Gg-SH3PXD2b         ATFIDKYKK------TSNASRPNFLAPLPSEMAQLRLGDAAAESSAT-EEATG---PCRP 441
Dr-SH3PXD2b         VTFIDKYKK------TSSASRPNFLAPLPGEMEQLKLEDTSSNSTNS---EHT---WSKP 453
Lg-p47phox-like     SAYLVERRK----------DAPVAAPPVPP------------------------------ 459
Ci-SH3PXD2          ETYISKTTKP-----KPTPARPQPPSFPKKTELQRASHTKVTRSSSLRQDKPA------- 454
Ci-p47phox          SSYLESKTT------EAEPARNSNPGFSAKMVLPKIEETGAGRSYDPGQAKQK------- 437
Sp-p47phox          SNYIEKRE--VSSRINGKPSLGNLDEETGEDKVPPVLPARPAFGSGGSGAFKP------- 472
Nv-p47phox-like     SSYLGKREKSVKKKATTSPKPARFQAQELRTKPKPVMPDIPDTGSKPKPARPP------- 460
Mb-p47phox-like     ADALQEENGVKATATSGAGVGPTVPRKVDTRPSVAPQSSAGVGAIAAALAQRAS------ 471
Hs-NOXO1            E-RRPRRQGRPRGCVDSVPHPTTEQ----------------------------------- 371
Cf-NOXO1            ASKYPPRAGQ-------------------------------------------------- 357
Mm-NOXO1            GQEQGTRVPR-------------------------------------------------- 349
Rn-NOXO1            GQEQRTQVPP-------------------------------------------------- 354
Gg-NOXO1            QQSA-------------------------------------------------------- 379
Xt-NOXO1            ------------------------------------------------------------
Dr-NOXO1            Q----------------------------------------------------------- 384
Ol-NOXO1            LATKTR------------------------------------------------------ 406
```

```
Tn-NOXO1            ESVKLPLARFSCSFRLHA------------------------------------------ 447

Mm-p47phox          ------------------------------------------------------------
Rn-p47phox          ------------------------------------------------------------
Cf-p47phox          ------------------------------------------------------------
Hs-p47phox          ------------------------------------------------------------
Gg-p47phox          ------------------------------------------------------------
Xt-p47phox          ------------------------------------------------------------
Tn-p47phox          ------------------------------------------------------------
Tr-p47phox          ------------------------------------------------------------
Ol-p47phox          ------------------------------------------------------------
Dr-p47phox          ------------------------------------------------------------
Mm-SH3PXD2a         KYEEPEYDVPAFG-FDSEPEMNEE--------PSGDRGSGDKHPAQPRRISPASSLQRAH 601
Rn-SH3PXD2a         KYEEPEYDIPAFG-FDSEPELNEE--------PTEDRGSGDKHPAQPRRTSPASSLQRAH 601
Hs-SH3PXD2a         KYEEPEYDIPAFG-FDSEPELSEE--------PVEDRASGERRPAQPHRPSPASSLQRAR 574
Gg-SH3PXD2a         KYEEPEYDVPTFG-FDSECEVNES--------HREE-GTPEKRLFQPLKPSPVSSLQKAK 606
Xt-SH3PXD2a         IYEEPEYDVPAFG-FDLEVELNVTN-------QSHGEELIQENLFQPSRPSPVSSLQRPK 606
Dr-SH3PXD2a         VYEEPEYDVPALG-FDSELDCNPPKPKTHNSPKPEP-RKFEIKSNPAAAERIAQAGKASP 501
Tr-SH3PXD2a1        VYEEPEYDVPPTG-CEREPDTNPVKNERNPDVKTTSGVSDKYHSSPAFTKAAPPVCKAPS 623
Tr-SH3PXD2a2        VYEEPEYDVPAIGDLDLESEFEFLRGESSLVDGKNEDTSSEKGSHMSSKPSPASSLHSAS 604
Hs-SH3PXD2b         LPDAPHGVMDSGLPWSKDWKGSKD---------VLRKASSDMSASAGYEEISDPDMEEKP 520
Cf-SH3PXD2b         LPDAPHGAMDSGMPWSKDWKGGKE---------VPRKASSDMSSCAGYEEISSPDLEEKP 495
Mm-SH3PXD2b         LPEAPHGAVDSGMLWSKDWKGGKE---------APRKASSDLSASTGYEEISDPTQEEKP 520
Rn-SH3PXD2b         LPEAPHGAVDSGMLWSKDWKGGKE---------APRKASSDLSASTGYEEISDPTQEEKP 519
Gg-SH3PXD2b         LPEAPPNGMDCGMKRAKDWKG-KE---------AT--ESGDLAFTCGYEEISDRDVEEKP 489
Dr-SH3PXD2b         LPDEPSS--NSDLSTRSKLREWKP---------NAAKSSSHFSGPLP-PPPSSPTAEEKP 501
Lg-p47phox-like     --------------------------------------------HAASKRASRVIYKTCA 475
Ci-SH3PXD2          ---------------------------------VMDRVEHTYSNDTNFRNFKPKPTLPKK 481
Ci-p47phox          ---------------------------------LIG-------------------SPLKL 445
Sp-p47phox          --------------------------------------VAKKSTPPPVDRSNSPSFTNRK 494
Nv-p47phox-like     --------------------------------------VSKKITIIPNNELSN-SFQN-- 479
Mb-p47phox-like     ---------------------------------TMATGTASKPLKPTKPATPGKPATPSK 498
Hs-NOXO1            ------------------------------------------------------------
Cf-NOXO1            ------------------------------------------------------------
Mm-NOXO1            ------------------------------------------------------------
Rn-NOXO1            ------------------------------------------------------------
Gg-NOXO1            ------------------------------------------------------------
Xt-NOXO1            ------------------------------------------------------------
Dr-NOXO1            ------------------------------------------------------------
Ol-NOXO1            ------------------------------------------------------------
Tn-NOXO1            ------------------------------------------------------------

Mm-p47phox          ------------------------------------------------------------
Rn-p47phox          ------------------------------------------------------------
Cf-p47phox          ------------------------------------------------------------
Hs-p47phox          ------------------------------------------------------------
Gg-p47phox          ------------------------------------------------------------
Xt-p47phox          ------------------------------------------------------------
Tn-p47phox          ------------------------------------------------------------
Tr-p47phox          ------------------------------------------------------------
Ol-p47phox          ------------------------------------------------------------
Dr-p47phox          ------------------------------------------------------------
```

```
Mm-SH3PXD2a        FKVGE-------SSEDVALEEETIYENEGFRPYTEDTLSARGSSGDSDSPGSSSLSLAVK 654
Rn-SH3PXD2a        FKVGE-------SSEDVALEEETIYENEGFRPYTEDTLSARGSSGDSDSPGNSSLSLAMK 654
Hs-SH3PXD2a        FKVGE-------SSEDVALEEETIYENEGFRPYAEDTLSARGSSGDSDSPGSSSLSLTRK 627
Gg-SH3PXD2a        FKVGE-------SSEDVANEEETIYENEGFRRYVEDALSNKESSGDSDSQKSSSLSLIRR 659
Xt-SH3PXD2a        FRVGE-------FSEDVTNEEETIYENEGFRPLVEEALSSKESSGDSDSQKSS--VTIQR 657
Dr-SH3PXD2a        LLKVMTSPLRKRNSLENINKEEVIYENEGFR---FSSDDFASGCDS-HTPR---SLTLGR 554
Tr-SH3PXD2a1       GVTHQRASFR---SVEEVSKAECIYENQNFRRGAGSERNLVKGCSEPNSPKSYHSSTVPR 680
Tr-SH3PXD2a2       FKMGESFEDGHDAGGEAEGDEECIYENDGFR---PFKETPERQCSRDSSSSRTSVFSESS 661
Hs-SH3PXD2b        SLPPR--------KESIIKSEGELLERERERQRTEQLRGPTPKPPGVILPMMPAKHIPPA 572
Cf-SH3PXD2b        SLPPR--------KESIIKSEGELQERER--QRMEQLRGSSPKPPGMILPMIPAKHTPPA 545
Mm-SH3PXD2b        SLPPR--------KESIIKSEEELLERER--QKMEPLRGSSPKPPGMILPMIPAKHAPLA 570
Rn-SH3PXD2b        SLPPR--------KESIIKSEEELLERER--QKMEPHRGSSPKPPGVILPMIPAKHAPLA 569
Gg-SH3PXD2b        SLPPR--------KESIIKSEGELLERQR----------PPPKPPGMILPMIPPKQSAAP 531
Dr-SH3PXD2b        ALPPR--------RESINKS-LELEDKPK--AELSKPLPPKPPVPGVIAPLVTPKAAPLK 550
Lg-p47phox-like    LFMAE------------NEGELGFDEGET------------------------------- 492
Ci-SH3PXD2         PLKPA-----------VNNQKPTFNAGNGQTPLPN------------------------- 505
Ci-p47phox         KLEGK-----------ERNTPFQLQAK--------------------------------- 461
Sp-p47phox         AVTNN-----------GRGGGDGVRRGSG------------------------------- 512
Nv-p47phox-like    -------------------DLKSALRSAG------------------------------- 489
Mb-p47phox-like    LATPAR--------SSGPKSDGATPPTLPLRPTMSGGTKPAKPVKPAKPSTPDASSKPAP 550
Hs-NOXO1           ------------------------------------------------------------
Cf-NOXO1           ------------------------------------------------------------
Mm-NOXO1           ------------------------------------------------------------
Rn-NOXO1           ------------------------------------------------------------
Gg-NOXO1           ------------------------------------------------------------
Xt-NOXO1           ------------------------------------------------------------
Dr-NOXO1           ------------------------------------------------------------
Ol-NOXO1           ------------------------------------------------------------
Tn-NOXO1           ------------------------------------------------------------


Mm-p47phox         ------------------------------------------------------------
Rn-p47phox         ------------------------------------------------------------
Cf-p47phox         ------------------------------------------------------------
Hs-p47phox         ------------------------------------------------------------
Gg-p47phox         ------------------------------------------------------------
Xt-p47phox         ------------------------------------------------------------
Tn-p47phox         ------------------------------------------------------------
Tr-p47phox         ------------------------------------------------------------
Ol-p47phox         ------------------------------------------------------------
Dr-p47phox         ------------------------------------------------------------
Mm-SH3PXD2a        NSPKSDSPKSSSLLKLKAEKNAQAELGKNQSNISFSSSVTISTTC--SSSSSSSSLSKNN 712
Rn-SH3PXD2a        NSPKSDSPKSSSLLKLKAEKNAQAELGKNQSNISFSSSVTIGTTS--SSSSSSSSLSKNS 712
Hs-SH3PXD2a        NSPKSGSPKSSSLLKLKAEKNAQAEMGKNHSSASFSSSITINTTCCSSSSSSSSSLSKTS 687
Gg-SH3PXD2a        NSPCSISPKSS-FMKPKMEKDIQPDVGKCHYSR--------------------------S 692
Xt-SH3PXD2a        NSPTAVSSKPP-LLKYKSERNVQQETKNVSFST--------------------------S 690
Dr-SH3PXD2a        KPFGSSSGGGKPLRKVSPDLN-RSHSLG------------------------------RA 583
Tr-SH3PXD2a1       RPSGTSPLAGRPIKTMTPETNRRSQTLGRRADLSC-----------------------RS 717
Tr-SH3PXD2a2       KTAGGGWRAGG--SKFKGDSNGSSFSNK------------------------------FE 689
Hs-SH3PXD2b        RDSRRPEPKPDKSRLFQLKNDMGLECG--------------------------------- 599
Cf-SH3PXD2b        RDGRRSEPKPDKGKLLQLKNEMGLECG--------------------------------- 572
Mm-SH3PXD2b        RDSRKPEPKLDKSK-FPLRNDMGLECG--------------------------------- 596
Rn-SH3PXD2b        RDSKKPEPKPDKSK-FPLRNDMGLECG--------------------------------- 595
Gg-SH3PXD2b        KDSKKPELKPEKGKLFQLKNEMGLECG--------------------------------- 558
```

```
Dr-SH3PXD2b         PDKPPEMKKDDKNKQ--------------------------------------------- 565
Lg-p47phox-like     ------------------------------------------------------------
Ci-SH3PXD2          ------------------------------------------------------------
Ci-p47phox          ------------------------------------------------------------
Sp-p47phox          ------------------------------------------------------------
Nv-p47phox-like     ------------------------------------------------------------
Mb-p47phox-like     VKPAKPSTPDTPSKPARPTMSG-------------------------------------- 572
Hs-NOXO1            ------------------------------------------------------------
Cf-NOXO1            ------------------------------------------------------------
Mm-NOXO1            ------------------------------------------------------------
Rn-NOXO1            ------------------------------------------------------------
Gg-NOXO1            ------------------------------------------------------------
Xt-NOXO1            ------------------------------------------------------------
Dr-NOXO1            ------------------------------------------------------------
Ol-NOXO1            ------------------------------------------------------------
Tn-NOXO1            ------------------------------------------------------------


Mm-p47phox          ------------------------------------------------------------
Rn-p47phox          ------------------------------------------------------------
Cf-p47phox          ------------------------------------------------------------
Hs-p47phox          ------------------------------------------------------------
Gg-p47phox          ------------------------------------------------------------
Xt-p47phox          ------------------------------------------------------------
Tn-p47phox          ------------------------------------------------------------
Tr-p47phox          ------------------------------------------------------------
Ol-p47phox          ------------------------------------------------------------
Dr-p47phox          ------------------------------------------------------------
Mm-SH3PXD2a         GDLKPRSASDAGIRDTPKV-----GTKKDPDVKAGLASCARAKPSVRPKPVLN--RAESQ 765
Rn-SH3PXD2a         GDLKPRSASDAGIRDSPKG-----GTKKDPDVKTGLASCVRAKPSVRPKPVLN--RAESQ 765
Hs-SH3PXD2a         GDLKPRSASDAGIRGTPKV-----RAKKDADANAGLTSCPRAKPSVRPKPFLN--RAESQ 740
Gg-SH3PXD2a         EETKPRSASDVGLRSMPRT-----GMKKEP----GQSPSIRAKPIVRPKPFLN--KADSQ 741
Xt-SH3PXD2a         EEIKPRSASDVGLRAVPKV-----STKKDAEQKQAVVPSTRAKPTVRPKPFLS--KADSQ 743
Dr-SH3PXD2a         ERHSSKLFSDESARNPKREP----VMRKDVEIRIGQSPLARPKPVVRPKPLLT--KSEPQ 637
Tr-SH3PXD2a1        HDSSPHSSSDELSRAPKK------TFSQGVEQRLGQSPSTRPKPSVRPKPLLA--KSEAQ 769
Tr-SH3PXD2a2        EGTGPKSALAESQN--KREQ----EESKPVSSTLSSS---KLKPVVRPKPQLA--KTS-- 736
Hs-SH3PXD2b         HKVLAKEVKKPNLRPISK---SKTDLPEEKPDATPQNPFLKSRPQVRPKPAPSPKTEPPQ 656
Cf-SH3PXD2b         HKVLAKEVKKPNLRPISK---SKADLPEEKPEGIPQNPFLKSKPQVRPKPAPSPRTEPPQ 629
Mm-SH3PXD2b         HKVLAKEVKKPNLRPISR---SKAELSEEKVDPTSQNLFMKSRPQVRPKPTPSPKTEPAQ 653
Rn-SH3PXD2b         HKVLAKEVKKPNLRPISR---SKAELPEEKVEPNPQNLFLKSRPQVRPKPTPSPKTEPAQ 652
Gg-SH3PXD2b         HKVSAKEVKKPNLRPIVKPTKPKAEPVEDKPEPITQNPFLKSRPQIKPKPAAAPRTDPPP 618
Dr-SH3PXD2b         ------------------------------------------------------------
Lg-p47phox-like     ------------------------------------------------------------
Ci-SH3PXD2          ------------------------------------------------------------
Ci-p47phox          ------------------------------------------------------------
Sp-p47phox          ------------------------------------------------------------
Nv-p47phox-like     ------------------------------------------------------------
Mb-p47phox-like     -------------------------------------VKPKPSLPGRPEPAANKVPAKPA 595
Hs-NOXO1            ------------------------------------------------------------
Cf-NOXO1            ------------------------------------------------------------
Mm-NOXO1            ------------------------------------------------------------
Rn-NOXO1            ------------------------------------------------------------
Gg-NOXO1            ------------------------------------------------------------
Xt-NOXO1            ------------------------------------------------------------
```

```
Dr-NOXO1            ------------------------------------------------------------
Ol-NOXO1            ------------------------------------------------------------
Tn-NOXO1            ------------------------------------------------------------

Mm-p47phox          ------------------------------------------------------------
Rn-p47phox          ------------------------------------------------------------
Cf-p47phox          ------------------------------------------------------------
Hs-p47phox          ------------------------------------------------------------
Gg-p47phox          ------------------------------------------------------------
Xt-p47phox          ------------------------------------------------------------
Tn-p47phox          ------------------------------------------------------------
Tr-p47phox          ------------------------------------------------------------
Ol-p47phox          ------------------------------------------------------------
Dr-p47phox          ------------------------------------------------------------
Mm-SH3PXD2a         SQEKMDISSLRRQLRPTGQLRGG-LKGSRSEDSELPPQMASE----GSRRGSADIIPLTA 820
Rn-SH3PXD2a         SQEKMDISSLRRQLRPTGQLRGG-LKGSRSEDSELPPQTASE----GSRRGSADIIPLPA 820
Hs-SH3PXD2a         SQEKMDISTLRRQLRPTGQLRGG-LKGSKSEDSELPPQTASEAPSEGSRRSSSDLITLPA 799
Gg-SH3PXD2a         SQEKMDISTLRRQLRPTGQLRGG-LKGSRSEESESPPHTASENHDDHVRRGSADLITAPS 800
Xt-SH3PXD2a         NQDKMDISSLRRQLRPTGQLRSG-LKGSKSEESENPPKTALENADSKPRRRSVDLTSNTS 802
Dr-SH3PXD2a         SPERMDISSIRRHLRPTGSLRQGAIRAMRGEDSETASVVSSE--DSTSSRSTSDLSSVYS 695
Tr-SH3PXD2a1        SPERMDMSSLRRQLRPTSQFQHG-LKPSRGEDSETASVISSE--DSMCSRSTSDLSSVYS 826
Tr-SH3PXD2a2        SSEQMDISSLRRQLRPTGQLKNS--IKMKNEDSETASVISSE--DSFSSQSTSDLSSIYS 792
Hs-SH3PXD2b         GEDQVDICNLRSKLRPAKSQDKSLLDGEGPQAVGGQDVAFSR------------------ 698
Cf-SH3PXD2b         GEDQVDICNLRSKLRPAKSQEKPLLDGEGS-----QDVACSR------------------ 666
Mm-SH3PXD2b         SEDHVDIYNLRSKLRPAKSQEKALLDGESHHAAGSHDTALSR------------------ 695
Rn-SH3PXD2b         GEDQVDIYNLRSKLRPAKSQEKALLDGESHHAAGGHDTALGR------------------ 694
Gg-SH3PXD2b         ADDKLDICSLRSKLRPAKCPEKPPEQDTAAS-----ESSCST------------------ 655
Dr-SH3PXD2b         ------------------------------------------------------------
Lg-p47phox-like     ------------------------------------------------------------
Ci-SH3PXD2          ------------------------------------------------------------
Ci-p47phox          ------------------------------------------------------------
Sp-p47phox          ------------------------------------------------------------
Nv-p47phox-like     ------------------------------------------------------------
Mb-p47phox-like     RPGRPDPASSVGVAVAASPASNAKKARAKSDFEGQPGESISLK----------------- 638
Hs-NOXO1            ------------------------------------------------------------
Cf-NOXO1            ------------------------------------------------------------
Mm-NOXO1            ------------------------------------------------------------
Rn-NOXO1            ------------------------------------------------------------
Gg-NOXO1            ------------------------------------------------------------
Xt-NOXO1            ------------------------------------------------------------
Dr-NOXO1            ------------------------------------------------------------
Ol-NOXO1            ------------------------------------------------------------
Tn-NOXO1            ------------------------------------------------------------

Mm-p47phox          ------------------------------------------------------------
Rn-p47phox          ------------------------------------------------------------
Cf-p47phox          ------------------------------------------------------------
Hs-p47phox          ------------------------------------------------------------
Gg-p47phox          ------------------------------------------------------------
Xt-p47phox          ------------------------------------------------------------
Tn-p47phox          ------------------------------------------------------------
Tr-p47phox          ------------------------------------------------------------
```

```
Ol-p47phox        ------------------------------------------------------------
Dr-p47phox        ------------------------------------------------------------
Mm-SH3PXD2a       T---TPPCVPKKEWEG-QGATYVTCSAYQKVQDSEISFPEGAEVHVLEKAVSGWWYVRFG 876
Rn-SH3PXD2a       T---TPPCVPKKEWEG-QGTAYVTCSAYQKVQDSEISFPAGAEVHVLEKVESGWWYVRFG 876
Hs-SH3PXD2a       T---TPPCPTKKEWEG-PATSYMTCSAYQKVQDSEISFPAGVEVQVLEKQESGWWYVRFG 855   (4th SH3)
Gg-SH3PXD2a       RGIFCSSHPAKTEQENDTRCFYVTTDSYQKVQDSEICFPAGVEVEVLEKQASGWWYLKYG 860
Xt-SH3PXD2a       Q---TSSNNDKSEESAEQKSFYTATSTYQKVLDSEISFPVGAKIEVLDKQDSGWWYIKYK 859
Dr-SH3PXD2a       K-------GSRGGESDHESVLFRTTDAYERAQESELSFPAGVEVEVLEKQESGWWFVRWG 748
Tr-SH3PXD2a1      K-------GSRCDSDVEGPNLYRSLDAYKKVQDSEVSFPAGVEVEVLEKEQSGWWYIRWG 879
Tr-SH3PXD2a2      K-------GSRGDSDLEGCAVYRTTDPYEKVQESELSFPAGVEVEVLEKQESGWWYVRWG 845
Hs-SH3PXD2b       --------------------------------------------SFLPGEGPGRAQDRTG 714
Cf-SH3PXD2b       --------------------------------------------SFLPGEGPGRTQDRTG 682
Mm-SH3PXD2b       --------------------------------------------SFLPGEGPGHGQDRSG 711
Rn-SH3PXD2b       --------------------------------------------SFLPGEGPGRGQDRSG 710
Gg-SH3PXD2b       --------------------------------------------PAVAPEASGRFQERPS 671
Dr-SH3PXD2b       ------------------------------------------------------------
Lg-p47phox-like   -------------------------------------------VDVMDKTDAEWWLVRIG 509
Ci-SH3PXD2        ------------------------------------------------------------
Ci-p47phox        ------------------------------------------------------------
Sp-p47phox        ----------------------------------------GVGAGGNMMAALKKQLEKSS 532
Nv-p47phox-like   ----------------------------------------PSAHGVEEVYVAKSDYEDKS 509
Mb-p47phox-like   ---------------------------------------AGEIVTVEEEAATGWWFVIKA 659
Hs-NOXO1          ------------------------------------------------------------
Cf-NOXO1          ------------------------------------------------------------
Mm-NOXO1          ------------------------------------------------------------
Rn-NOXO1          ------------------------------------------------------------
Gg-NOXO1          ------------------------------------------------------------
Xt-NOXO1          ------------------------------------------------------------
Dr-NOXO1          ------------------------------------------------------------
Ol-NOXO1          ------------------------------------------------------------
Tn-NOXO1          ------------------------------------------------------------


Mm-p47phox        ------------------------------------------------------------
Rn-p47phox        ------------------------------------------------------------
Cf-p47phox        ------------------------------------------------------------
Hs-p47phox        ------------------------------------------------------------
Gg-p47phox        ------------------------------------------------------------
Xt-p47phox        ------------------------------------------------------------
Tn-p47phox        ------------------------------------------------------------
Tr-p47phox        ------------------------------------------------------------
Ol-p47phox        ------------------------------------------------------------
Dr-p47phox        ------------------------------------------------------------
Mm-SH3PXD2a       E-LEGWAPSHYLVAEENQQPDTASKEGDTG--KSSQNEGKSDSLEKIEKRVQALNTVNQS 933
Rn-SH3PXD2a       E-LEGWAPSHYLVPEENQQPDPDSKEPDTV--KSTQNEGKSDSLEKIEKRVQALNTVNQS 933
Hs-SH3PXD2a       E-LEGWAPSHYLVLDENEQPDPSGKELDTVPAKGRQNEGKSDSLEKIERRVQALNTVNQS 914
Gg-SH3PXD2a       D-MEGWAPSHYLALPDNQQ-ETTSVETDASFARNRKNENKSNSLEKIEKRVQALNTINQS 918
Xt-SH3PXD2a       D-SEGWAPSHFLEEVDNKKADTSIAESEST--KIKKNENKSNSLEKIEKRVQALNTINQS 916
Dr-SH3PXD2a       S-DEGWVPTFYLEPIKHTH-NVGIQESRDSPLVDLGSTNKSNSLEKNEQRVQALNNLNQQ 806
Tr-SH3PXD2a1      S-EEGWAPSYYLESVRQVG-DAGS------------GGGKSDSLEKNEQNVLTLNNINIQ 925
Tr-SH3PXD2a2      D-TEGWAPTYYLEPLRQQD-DFAGSESEGSP-SKPGSLSKSNSLEKNEQRVQALNNINQN 902
Hs-SH3PXD2b       K-QDGLSPKEISCRAPPRP------------AKTTDPVSKSVPVPLQEAP-QQRPVVPPR 760
Cf-SH3PXD2b       K-QDGLSPKEVPCRAPPKP------------VKTADPVPKNVPTPLQEAS-PQRPVVLPR 728
Mm-SH3PXD2b       R-QDGLSPKETPCRAPPRP------------AKTTDPGPKNVPVPVQEATLQQRPVVPPR 758
```

```
Rn-SH3PXD2b         R-QDGLSPKETPCRAPPRP------------AKTTDPGPKNVPVPVQEAT-QQRPVVPPR 756
Gg-SH3PXD2b         V-ENKALPK-----SPPGP------------AVAP---------AAREPT-PQRPVVPPR 703
Dr-SH3PXD2b         ------------------------------------------------------------
Lg-p47phox-like     N-EEGWAPRSYIEQVEIED---------------------------------TRG----- 530
Ci-SH3PXD2          ------------------------------------------------------------
Ci-p47phox          ------------------------------------------------------------
Sp-p47phox          V-GESSSPAIPARPGVAPK----------------------------------------- 550
Nv-p47phox-like     D-GENWAPAN-FLEMKSPE----------------------------------------- 526
Mb-p47phox-like     DKSEGWAPADYLDLTGSNS----------------------------------------- 678
Hs-NOXO1            ------------------------------------------------------------
Cf-NOXO1            ------------------------------------------------------------
Mm-NOXO1            ------------------------------------------------------------
Rn-NOXO1            ------------------------------------------------------------
Gg-NOXO1            ------------------------------------------------------------
Xt-NOXO1            ------------------------------------------------------------
Dr-NOXO1            ------------------------------------------------------------
Ol-NOXO1            ------------------------------------------------------------
Tn-NOXO1            ------------------------------------------------------------


Mm-p47phox          ------------------------------------------------------------
Rn-p47phox          ------------------------------------------------------------
Cf-p47phox          ------------------------------------------------------------
Hs-p47phox          ------------------------------------------------------------
Gg-p47phox          ------------------------------------------------------------
Xt-p47phox          ------------------------------------------------------------
Tn-p47phox          ------------------------------------------------------------
Tr-p47phox          ------------------------------------------------------------
Ol-p47phox          ------------------------------------------------------------
Dr-p47phox          ------------------------------------------------------------
Mm-SH3PXD2a         ---------KRATPPIPSKPPGGFGKTSGT---VAVKMRNGVRQVAVRPQSVFVSPP--- 978
Rn-SH3PXD2a         ---------KRATPPIPSKPPGGFGKTSGT---VAVKMRNGVRQVAVRPQSVFVSPP--- 978
Hs-SH3PXD2a         ---------KKATPPIPSKPPGGFGKTSGT---PAVKMRNGVRQVAVRPQSVFVSPP--- 959
Gg-SH3PXD2a         ---------KRATPPIPSKPPGGFSKTSG-----PVKMRNGVRQLAVRPQSVFVSPP--- 961
Xt-SH3PXD2a         ---------KRATPPVPSRPPGGFSKPPGPG-SNVVKLRNGVKQVTVRPQSVFVSAP--- 963
Dr-SH3PXD2a         ----NLRSMSNPSPPIPSKPPGGFSKPTAMLNGSSVRMRNGVRQAAVRPQSVFVSPPQPL 862
Tr-SH3PXD2a1        GRNQQHRGLKRNTPPIPSKPPGGFSKPSGMVNGG-VRMRNGVRQVAVRPQSVFVTTTQSA 984
Tr-SH3PXD2a2        --------LKKVTPPIPSKPPGGLSKPISFFGSR---KQNSAKQQVVRPQSVLISAP--I 949
Hs-SH3PXD2b         ------------RPPPPKKTSSSS---RPLPEVRGPQCEGHESRAAPTPGRALLVPP--- 802
Cf-SH3PXD2b         ------------RPPPPKKTSSS----RPLPEVRGPQREASEGKAAPAPGRALLVPP--- 769
Mm-SH3PXD2b         ------------RPPPPKKTSSSPLSCRPLPEVRGAQRE--ESRVAPAAGRALLVPP--- 801
Rn-SH3PXD2b         ------------RPPPPKKTSSSPLSCRPLPEVRGSQRE--ESRAIPASGRALLVPP--- 799
Gg-SH3PXD2b         ------------RPPPPKKTTSPVAG--PVPEAR----------ASPLPGRPMLVPP--- 736
Dr-SH3PXD2b         ------------------------------------------------------------
Lg-p47phox-like     ------------KPPAPPK---------------------------GKPSIQFPLPAR-- 549
Ci-SH3PXD2          ------------------------------------------------------------
Ci-p47phox          ------------------------------------------------------------
Sp-p47phox          ------------------------------------------------------------
Nv-p47phox-like     ------------------------------------------------------------
Mb-p47phox-like     ----------DSQPGAPAKPAKPSKPSK-------------------------------- 696
Hs-NOXO1            ------------------------------------------------------------
Cf-NOXO1            ------------------------------------------------------------
Mm-NOXO1            ------------------------------------------------------------
Rn-NOXO1            ------------------------------------------------------------
```

```
Gg-NOXO1            ------------------------------------------------------------
Xt-NOXO1            ------------------------------------------------------------
Dr-NOXO1            ------------------------------------------------------------
Ol-NOXO1            ------------------------------------------------------------
Tn-NOXO1            ------------------------------------------------------------


Mm-p47phox          ------------------------------------------------------------
Rn-p47phox          ------------------------------------------------------------
Cf-p47phox          ------------------------------------------------------------
Hs-p47phox          ------------------------------------------------------------
Gg-p47phox          ------------------------------------------------------------
Xt-p47phox          ------------------------------------------------------------
Tn-p47phox          ------------------------------------------------------------
Tr-p47phox          ------------------------------------------------------------
Ol-p47phox          ------------------------------------------------------------
Dr-p47phox          ------------------------------------------------------------
Mm-SH3PXD2a         PKDNNLSCALRRNESLTATDSLRG------VRRNSSFSTARSAAAEAKGRLA-------- 1024
Rn-SH3PXD2a         PKDNNLSCALRRNESLTATDSVRG------VRRNSSFSTARSAAAEAKGRLA-------- 1024
Hs-SH3PXD2a         PKDNNLSCALRRNESLTATDGLRG------VRRNSSFSTARSAAAEAKGRLA-------- 1005
Gg-SH3PXD2a         PKDNNLSCSLRRNESLTATDHLRN------VRRNSSFSNARSQPGDVKGKQP-------- 1007
Xt-SH3PXD2a         PKDNNISCNLRRNESLSATDHLRG------VRRNSSFNTVRSQPNQTKISSS-------- 1009
Dr-SH3PXD2a         KETNIHTGSLRRNESLGAGDHLRS---TGGVRRNSSFTAVRPQPVTDVRVR--------- 910
Tr-SH3PXD2a1        KDSHYMTGSLRRNDSLSRSDHYGSGSATLGVRRNASFSTVRPHVVVESHTRPVERSGVGS 1044
Tr-SH3PXD2a2        MDPPSSLGALRRNESLNSTDHPRV---SPTVRRNASFGTAPRGLVANNLALP-------- 998
Hs-SH3PXD2b         KAKPFLSNSLGGQDDTRGKGSLGP------------------------------------ 826
Cf-SH3PXD2b         KAKPFLSNSSGGHDDMRGKGGLGP------------------------------------ 793
Mm-SH3PXD2b         KAKPFLSNSSVGQDDMRGKGGLGP------------------------------------ 825
Rn-SH3PXD2b         KAKPFLSNSSVGQDDIRGKGGLGP------------------------------------ 823
Gg-SH3PXD2b         KARPFLS--AAIQDEAKVKSSVGP------------------------------------ 758
Dr-SH3PXD2b         ------------------------------------------------------------
Lg-p47phox-like     ------------------------------------------------------------
Ci-SH3PXD2          ------------------------------------------------------------
Ci-p47phox          ------------------------------------------------------------
Sp-p47phox          ------------------------------------------------------------
Nv-p47phox-like     ------------------------------------------------------------
Mb-p47phox-like     ------------------------------------------------------------
Hs-NOXO1            ------------------------------------------------------------
Cf-NOXO1            ------------------------------------------------------------
Mm-NOXO1            ------------------------------------------------------------
Rn-NOXO1            ------------------------------------------------------------
Gg-NOXO1            ------------------------------------------------------------
Xt-NOXO1            ------------------------------------------------------------
Dr-NOXO1            ------------------------------------------------------------
Ol-NOXO1            ------------------------------------------------------------
Tn-NOXO1            ------------------------------------------------------------


Mm-p47phox          ------------------------------------------------------------
Rn-p47phox          ------------------------------------------------------------
Cf-p47phox          ------------------------------------------------------------
Hs-p47phox          ------------------------------------------------------------
Gg-p47phox          ------------------------------------------------------------
Xt-p47phox          ------------------------------------------------------------
```

```
Tn-p47phox        ------------------------------------------------------------
Tr-p47phox        ------------------------------------------------------------
Ol-p47phox        ------------------------------------------------------------
Dr-p47phox        ------------------------------------------------------------
Mm-SH3PXD2a       -ERAASQGSESP--LLPTQRKGIPVSPVRPKPIEKSQFIHNNL-KDVYISIADYEGDE-E 1079
Rn-SH3PXD2a       -ERAASQGSESP--LLPTQRNGIPVSPVRPKPIEKSQFIHNNL-KDVYVSIADYEGDE-E 1079
Hs-SH3PXD2a       -ERAASQGSDSP--LLPAQRNSIPVSPVRPKPIEKSQFIHNNL-KDVYVSIADYEGDE-E 1060 (5th SH3)
Gg-SH3PXD2a       -ERSGSEGSETTS-NLPTQRNGIPVSTVRPKPIEKSQFIHNNL-KDIYVSIADYEGDE-E 1063
Xt-SH3PXD2a       -AKPDGDMSESETSIRPSQKNGIPVSTVRPKPIEKSQFIHNNL-KDIYISIADYEGDD-E 1066
Dr-SH3PXD2a       AGTTITAP-AGSSSPLIAQRNGIPISTVRPKPIEKMQLIHNNL-REVYVSIADYRGDE-E 967
Tr-SH3PXD2a1      SGSSLSTGNVQDALARANQRNGIPVSAVRPKPIEKNQLIHNNLGRDVYVSIADYCGDE-E 1103
Tr-SH3PXD2a2      -SRNRSGTGSSESLGLGSVKNSLPVSTVKPKP----HIIHNNL-REIYVSIADYHGDE-E 1051
Hs-SH3PXD2b       ----------------WGTG---KIGENREKAAAASVPNADGLKDSLYVAVADFEGDK-D 866 (4th SH3)
Cf-SH3PXD2b       ----------------WLVG---KIGENREKVAAAPFFPSADGSKDSLYVAVANFEGDK-D 833
Mm-SH3PXD2b       ----------------RVTG---KVGETREK--AASFLNADGPKDSLYVAVANFEGDE-D 863
Rn-SH3PXD2b       ----------------RIAG---KVGETREK--AASFLNADGPKDSLYVAVANFEGDE-D 861
Gg-SH3PXD2b       ----------------KVISKAVERGEGRER-TSAPFSNPDVSKEALYVAVADFEGDE-E 800
Dr-SH3PXD2b       ------------------------------------------------------------
Lg-p47phox-like   ------------------------------------------------------------
Ci-SH3PXD2        ------------------------------------------------------------
Ci-p47phox        ------------------------------------------------------------
Sp-p47phox        --------------------------------LSKPTAQPYCNRDAAYITTSSYVNDEND 578
Nv-p47phox-like   --------------------------------YAKQDKP----AKHVYVALASYHDEDDD 550
Mb-p47phox-like   -----------------------PSMPAKPSRPGSNEASSNLRDDEFLVLDTAYNADGEG 733
Hs-NOXO1          ------------------------------------------------------------
Cf-NOXO1          ------------------------------------------------------------
Mm-NOXO1          ------------------------------------------------------------
Rn-NOXO1          ------------------------------------------------------------
Gg-NOXO1          ------------------------------------------------------------
Xt-NOXO1          ------------------------------------------------------------
Dr-NOXO1          ------------------------------------------------------------
Ol-NOXO1          ------------------------------------------------------------
Tn-NOXO1          ------------------------------------------------------------


Mm-p47phox        ---------------------------------------------
Rn-p47phox        ---------------------------------------------
Cf-p47phox        ---------------------------------------------
Hs-p47phox        ---------------------------------------------
Gg-p47phox        ---------------------------------------------
Xt-p47phox        ---------------------------------------------
Tn-p47phox        ---------------------------------------------
Tr-p47phox        ---------------------------------------------
Ol-p47phox        ---------------------------------------------
Dr-p47phox        ---------------------------------------------
Mm-SH3PXD2a       TAGFQEGVSMEVLEKNPNGWWYCQILDEVKPFKGWVPSNYLEKKN 1124
Rn-SH3PXD2a       TAGFQEGVSMEVLERNPNGWWYCQILDEVKPFKGWVPSNYLEKKN 1124
Hs-SH3PXD2a       TAGFQEGVSMEVLERNPNGWWYCQILDGVKPFKGWVPSNYLEKKN 1105
Gg-SH3PXD2a       TAGFQEGVCMEVLERNPNGWWYCQIMNGVKPFKGWVPSNYLEKKN 1108
Xt-SH3PXD2a       TVGFQEGVSMEVLEKNPNGWWYCQILDGGKPFKGWVPSNYLEKKN 1111
Dr-SH3PXD2a       TMGFSEGTSLEVLEKNPNGWWYCQVLDGLQGRKGWVPSNYLERKK 1012
Tr-SH3PXD2a1      TMGFTEGTCLEVLERNPNGWWYCQVQDSLIPRKGWVPSNYLERK- 1147
Tr-SH3PXD2a2      TMGFPEGTSLEVLDRNPNGWWYCKILDNGKQRKGWVPSNYLERKH 1096
Hs-SH3PXD2b       TSSFQEGTVFEVREKNSSGWWFCQVLSGAPSWEGWIPSNYLRKKP 911
```

```
Cf-SH3PXD2b         TSSFQEGTVFEVREKSSSGWWFCQVLSGAPSWEGWIPSNYLKKKP 878
Mm-SH3PXD2b         TSSFQEGTVFEVREKNSSGWWFCQVLSGAPSWEGWIPSNYLRKKP 908
Rn-SH3PXD2b         TSSFQEGTVFEVREKNSSGWWFCRVLSGAPSWEGWIPSNYLRKKP 906
Gg-SH3PXD2b         TNSFREGTLFEVREKNSSGWWFCKVLTGGPCWEGWIPSNYLRKKP 845
Dr-SH3PXD2b         ---------------------------------------------
Lg-p47phox-like     ---------------------------------------------
Ci-SH3PXD2          ---------------------------------------------
Ci-p47phox          ---------------------------------------------
Sp-p47phox          GLSFEEGQRVEVIKKDDSGWWSVRIGNT----EGWVPNTFLEKI- 618
Nv-p47phox-like     AISFDEGDEMEVVQQDDSGWWLVKIADK----SGWAPSNFLKQL- 590
Mb-p47phox-like     ELSLRPREKVTVLEKATEWWFCR----NAQGQEGWAPANYLSKR- 773
Hs-NOXO1            ---------------------------------------------
Cf-NOXO1            ---------------------------------------------
Mm-NOXO1            ---------------------------------------------
Rn-NOXO1            ---------------------------------------------
Gg-NOXO1            ---------------------------------------------
Xt-NOXO1            ---------------------------------------------
Dr-NOXO1            ---------------------------------------------
Ol-NOXO1            ---------------------------------------------
Tn-NOXO1            ---------------------------------------------
```
