# Supplementary material for: Molecular evolution of Phox-related regulatory subunits for NADPH oxidase enzymes
Source: BMC Evol Biol. 2007 Sep 27;7:178. doi: 10.1186/1471-2148-7-178 (PMC2121648; doi:10.1186/1471-2148-7-178)
Supplement: Additional file 12 — Amino acid sequences and alignment of predicted fungal NOXR partner: Bem1, Cdc24, CBSn-PB1. Amino acid sequences Bem1, Cdc24, CBSn-PB1 of A. nidulans, M. grisea and F. graminearum are provided. Alignments of each gene family are shown. [file 1471-2148-7-178-S12.pdf]

Additional File 12

Amino acid sequences and alignment of predicted fungal NOXR partner: Bem1, Cdc24, CBSn-PB1. To describe species of sequences, we used the following naming: Mg (*Magnaporthe grisea*), Fg (*Fusarium graminearum*), An (*Aspergillus nidulans*). Sequences are available from GenBank^{TM} (http://www.ncbi.nlm.nih.gov/) by indicated accession numbers. *Gray boxes* indicate predicted characteristic domains. *Underlines* in alignment of Bem1 and Cdc24 protein sequences indicate serine-rich region (SRR) and PRR, respectively.

Bem1 ortholog

>An-Bem1： GenBank No. XP664634

MKALRRSLKGEKDSKPHHHHQHHHQQHHHLSITPKSAVAILPPKKVIKALYDYQPEPGNTQELAFSKGDFFHVISREDDSDWYEACNPLIPTAR
GLVPVTFFEVIGKTQRDSSGSIDLGKKKEPHDSGFADRATSSGSEFGSGSSKSYQSHPAFPRTSMMGKAGGGAMVYGVVQYDFQAERPDELDAK
AGEAIIVIAQSNPEWFVAKPIGRLGGPGLIPVSFIELRDMQSGQAVADPLEAVRRAGVPKVEEWKKMTAEYKNSSISLGKIETTGSMQSVTSGM
ERMSVRQSADHVSHMSQNGHANAYHNRNASKASIAPSTHQQPPPHSQQPLVAPIAASIPRYCFDNDKYWYIIEVKMEDGRCWELSRYYHDFYDF
QIALLTQFEEEAGNRGKPRTLPFMPGPVAHVTDAISDGRRHNLDEYIKKLLAMPPHITRCALVRQLFAPRPGDFEIDPSAFGEDARFSGGSQHS
SGHELSPSASRQSSQQQNGPSERSSHSHSHSHSHSHQRSQTSSSIPNGKPPMVRQQSSLTQVSTSSGGALKVKVNFQDDLIAIRVPSDINVQQL
KEKLMDRLKINDEIVVQYKDEASGAYVDLISDSDLDTAIQRNSKLTLYVGLA

>Mg-Bem1： GenBank No. XP363776

MKALRRSIKGDKDKQPQISIAPKSAVAIVPPKKVIRALYDYEAQSTQELSFSRGDFFHVIGRENDTDWYEACNPALPDARGLVPVAFFQALGRT
ERDSAQSDTSVTRTSAPVPSKNPDHDSGYAETSAPVPSTPGFSNRDSKSGKSGAMVYGIVMYDFQAERADELQAQAGEAIIVIAQSNPEWFVAK
PIGRLGGPGLIPVSFIEIRDMASNTPVPDPEGAVRRAGIPRVEEWKKMAAEYKNSSITLGKFEVGGAPQQQAIEQGMDRMSLQPTDSRSSQGQL
NGKLQQAMQPQNAYPTPKPSTQLYAPVQASIPRYCFAEEKYWFVIEAILEDGRKYELSRYYEDFYDFQIALLTEFPAEAGNTGTQKRTLPYMPG
PVNYVTDAITEGRLHNLDAYVKNLLAQPTYISRCNLVKQFFAPREGDYEMDANGEEDEYRMSGGSQQSSPDSPNGGISQQSSRNNLSSGGYNNN
NLAPSQQRGLSAQPPMMRQASSLSQPSQASLSPGIPQAGAFMKVKLSYNGDLIAIRVPSDIQFRELFDKITERLRIQPGEEVQLSYKDDITNGK
LALMNDQDLIYAMQRNEKLLFYHRYSNSFDR

>Fg-Bem1: GenBank No. XP385437

MKALRRSIKGDKDKGPVTPKSAVAIVPPKKVIRALYDYEARSSQELSFSRGDFFHVIGRENDDDWYEACNPALPDARGLVPVTFFQALGRTERD

SAQSDGGQPPSPPKNPDHDSGYSESPAMHPAPAVMSPTTNSAPQGHQRNSKSVGKTGAMVYGIVMYDFAAERADELEAKAGEAIIVIAQSNPEW

FVAKPIGRLGGPGLIPVSFVEIRDMASDKAIQNPGDAIKRAGVPKVEEWKKMAAEYKNSSITLGKFEGGGPGQPGQGIEQGMERMSIQQQQHSR

QPSQNGTQSGYASQPRTSQQPQPHQSQVQKPAAPLHAPLEARIPRYCFAEDKYWFVIEAVLEDGRQWELSRYYEDFYDFQIALLTEFPAEAGNT

GTQKRTLPYMPGPVNYVTDAITEGRLHNLDAYVKNMLNQPPYISRCNLVRQFFAPREGDYEIDPNDNENEYRLSQGSQLSSMESPAGGSQNNLN

GSGYGLAPSHPDMQTQASSLSQPSQASLGGGMQAPSQPPSAMKIKMYFNGDLIAIRVPTDISFQALYDKICDRLKIPANEEIQLFYKDEPTGDK

PSMISDNDLDFALQRNEKLLLYVEAV

```
                                                             SH3-N
Mg-XP363776     MKALRRSIKGDKDKQPQ------------ISIAPKSAVAIVPPKKVIRALYDYEAQ--ST 46
Fg-XP385437     MKALRRSIKGDKDKGP---------------VTPKSAVAIVPPKKVIRALYDYEAR--SS 43
An-XP664634     MKALRRSLKGEKDSKPHHHHQHHHQQHHHLSITPKSAVAILPPKKVIKALYDYQPEPGNT 60
                ******* ** **  *                 ******* ****** *****

Mg-XP363776     QELSFSRGDFFHVIGRENDTDWYEACNPALPDARGLVPVAFFQALGRTERDSAQSDTSVT 106
Fg-XP385437     QELSFSRGDFFHVIGRENDDDWYEACNPALPDARGLVPVTFFQALGRTERDSAQSDGGQP 103
An-XP664634     QELAFSKGDFFHVISREDDSDWYEACNPLIPTARGLVPVTFFEVIGKTQRDSSGS----- 115
                *** ** ******* ** * ********  * ******* **   * * ***  *
                                                                          SH3-C
Mg-XP363776     RTSAPVPSKNPDHDSGYAETSAPVPSTPGFS----------NRDSKS-GKSG--AMVYGI 153
Fg-XP385437     ----PSPPKNPDHDSGYSESPAMHPAPAVMSPTTNSAPQGHQRNSKSVGKTG--AMVYGI 157
An-XP664634     ---IDLGKKKEPHDSGFADRATSSGSEFGSGSSKSYQSHPAFPRTSMMGKAGGGAMVYGV 172
                        *   ****                                ** *  *****

Mg-XP363776     VMYDFQAERADELQAQAGEAIIVIAQSNPEWFVAKPIGRLGGPGLIPVSFIEIRDMASNT 213
Fg-XP385437     VMYDFAAERADELEAKAGEAIIVIAQSNPEWFVAKPIGRLGGPGLIPVSFVEIRDMASDK 217
An-XP664634     VQYDFQAERPDELDAKAGEAIIVIAQSNPEWFVAKPIGRLGGPGLIPVSFIELRDMQSGQ 232
                * *** *** *** * ********************************** * *** *

Mg-XP363776     PVPDPEGAVRRAGIPRVEEWKKMAAEYKNSSITLGKFEVGGAPQQ-QAIEQGMDRMSLQP 272
Fg-XP385437     AIQNPGDAIKRAGVPKVEEWKKMAAEYKNSSITLGKFEGGGPGQPGQGIEQGMERMSIQQ 277
An-XP664634     AVADPLEAVRRAGVPKVEEWKKMTAEYKNSSISLGKIETTGSMQS---VTSGMERMSVRQ 289
                    *  *  *** * ******* ******** *** *  *  *       ** ***

Mg-XP363776     --TDSR--SSQGQLNGKLQQ--AMQPQNAYPTP----KPSTQLYAPVQASIPRYCFAEEK 322
Fg-XP385437     Q-QHSRQPSQNGTQSGYASQ--PRTSQQPQPHQSQVQKPAAPLHAPLEARIPRYCFAEDK 334
An-XP664634     SADHVSHMSQNGHANAYHNRNASKASIAPSTHQQPPPHSQQPLVAPIAASIPRYCFDNDK 349
                        *  *                              * **  * ******   *
                PX
Mg-XP363776     YWFVIEAILEDGRKYELSRYYEDFYDFQIALLTEFPAEAGNTGTQKRTLPYMPGPVNYVT 382
Fg-XP385437     YWFVIEAVLEDGRQWELSRYYEDFYDFQIALLTEFPAEAGNTGTQKRTLPYMPGPVNYVT 394
An-XP664634     YWYIIEVKMEDGRCWELSRYYHDFYDFQIALLTQFEEEAGNRG-KPRTLPFMPGPVAHVT 408
                **  **   ****  ****** *********** *  **** *   **** *****  **
                                                                       SRR
Mg-XP363776     DAITEGRLHNLDAYVKNLLAQPTYISRCNLVKQFFAPREGDYEMDANGEEDEYRMSGGSQ 442
Fg-XP385437     DAITEGRLHNLDAYVKNMLNQPPYISRCNLVRQFFAPREGDYEIDPNDNENEYRLSQGSQ 454
An-XP664634     DAISDGRRHNLDEYIKKLLAMPPHITRCALVRQLFAPRPGDFEIDPSAFGEDARFSGGSQ 468
                ***  ** **** * *  *  *  * ** ** * **** ** * *        * * ***
```

```
Mg-XP363776       QSS--PDSPNGGISQQSSRNNLSSGGYNNNNLAPSQQRGLSAQPPMMRQASSLSQPSQAS 500
Fg-XP385437       LSS--MESPAGG-----SQNNLNGSGYG---LAPS-------HPDMQTQASSLSQPSQAS 497
An-XP664634       HSSGHELSPSASRQSSQQQNGPSERSSHSHSHSHSHQRSQTSSSIPNGKPPMVRQQSS 528
                   **    **          *              *                      * *

                                 PB1
Mg-XP363776       LSPGIPQAG---AFMKVKLSYNGDLIAIRVPSDIQFRELFDKITERLRIQPGEEVQLSYK 557
Fg-XP385437       LGGGMQAPSQPPSAMKIKMYFNGDLIAIRVPTDISFQALYDKICDRLKIPANEEIQLFYK 557
An-XP664634       LTQVSTSSG---GALKVKVNFQDDLIAIRVPSDINVQQLKEKLMDRLKIN--DEIVVQYK 583
                  *              * *     ******** **    *  *   ** *    *    **

Mg-XP363776       DDITNGKLALMNDQDLIYAMQRNEKLLFYHRYSNSFDR 595
Fg-XP385437       DEPTGDKPSMISDNDLDFALQRNEKLLLYVEAV----- 590
An-XP664634       DEASGAYVDLISDSDLDTAIQRNSKLTLYVGLA----- 616
                  *           * **  * *** **  *
```

Cdc24 ortholog

>Mg-Cdc24 :GenBank No. XP364852

MNQASRRQPGLNAPRDTKLAGSVQSNDVNQQPLLPTPAPKPPDQSLPGPSRRSASENIGVGHPTCPVAFTPPNALAMAYAPLLRTNTTPIFPSS

TGGVARLQGTMGSTGPAVGIPPLRASQLSGTTLHNSTTSLSSLASASTVVPAQNGGQVVATSNIINQKADASRSLYQICMSLKQRLAQVPGFEE

YMSLMDQWEAEDPEGGIVESVWKLLRMGTPLIVIFNLLKPENPMSFDPTPDAKKAKMYIYKFAEQCKNELGIEDIFTISDLLKNDTTGFVKVTT

VVNQVLDIAQTRGMLIQQQPYPEDDMPDSGKSKMSYRDYIVRELVDTERKYVQDLENLHDLKKALTERGIVTGDVAYQIFGNIDAILDFQRRFL

IRVETTNSMPGEVQQWGSPFVAMEASFDPIYTPFIANQRKAALIAQSEFDKIKSIEHPVACDFNTLDGFLLKPMQRLVKYPLLLKDLLKKSEDD

KVKDDLSLGIAAAERTLQKANEAVDRDLLDDALEDLKTRVDDWKNHQVDQFGSLLLHGVHTVLTGKSESERDYEIYLFECILLCCKEISSTKSK

DKKDKTKSTGPKIRNKGAKLQLKGRIFMTNVTEVLSFAKPGSYTVQIWWKGDPGVENFIIKFQNEEMMKKWAAGLDQQRKASGSQSATSPDQPT

AEFAWMRSQSAVENPYAQTQPEEEEEYEFGAIPQQSQQYQAMAGTMPRMNSTASIRGRSQTGESSQSIASIARGPPPRFPLPQPPQPLSVQTQL

QGGNSPGFRPGDSYFSPIGESPVSSRTSTASNVFPGSAGYGSFKGVTGQPYPGMWDGPIVDQNRYTAPAMPRAPSRDGPSPVNAYGMSGANGRN

PRGPSMPVMASHSAQGASQQSRSRSYSTPDIQGQGPPGMRRTPGGSQSNVPAVPGIPAHLHAAHERHDSNIPRSNTGSPALSLRNDLPMRSNTQ

SPGVQRDRLMQQGGGYSGGTLAQFPMQPVYPRGGTPNPPGSGHTPAPLNLSADMGRTVSPPLGTGTPQPPNTAGLMSPDQGGGIMPTQLKVKVN

YDTSNYMTLVAAYNITYRSLVDRIDAKLARFTNSSISRGNLRLRYRDEESDFVSIVCDEDVQMAFTDGRNGDRDMYNGGVGEVELFCVGIGGE

>Fg-Cdc24 :GenBank No. XP390087

MNHAPRASQLSGSTAYTGSSASLSSLATATTITPTNGGPVHATANIINQKADASRSLYQICISLKQRLAQVPGFGPYLDELDPTDPVDPLWNLF

RSGYPLLLIYNALRPNEELKVDDSSSANEAKKSKIAIFKFVQACMKELEIPSTQSFVITDLMGTDTSGFVKVTQVVNYVLDRAEERGYLMQAQP

DVESNEPTGGQMTYRDHIIRELVDTERKYVQDLENLHDLKKTLEQQGEIPGDTLHQIFFNINAILDFQRRFLIRVETTNSMPALNQRWGAPFVH

YEDALIDIYQPFIANQRKAAQVANQVFDKIQRSSHPVAADYNTLDGFLLKPMQRLVKYPLLLKDLNKKTEDDEVKMDLTTGCEAAERVLSKANE

AVNRDLLDEAVEDLTSRVEDWKSHKVEQFGKLLLHGVYGVITGKTDQEKDYEIYLFESILLCCKEISSSKSKDKKDKLRSGGPKARNKSAKLQL

KGRIFMTNVTDIVSFTKPGSHSVQIWWKGDPGVENFIIKFLNEETLKKWVNTLETQRKHNVPRQSTNSDTLSTDFAWTRDQVAGLENPYLKEND

DEDDEDIGPATAPAGFPGVTHPMSLGPRTASSNNLRARAGTGESSASLAGMVRAPPPRFPLPAPPGSLSLQTQPNGAHSPSAWAGDSYFSPVTE

SPASTRTSTTSGMFSTPQYGFPKSATPNPQQWEDANGNRYTAPAMPRAPSRDGPSPNPARNPRGPSLPAMSSSSQAALAAQQRNRSYSTPDING

PGMPRTRQPSHGNIPAVPGIPQHLHPGHNPNLSRDQTGSPRNDQPTRAQTNSPGAQRERMHKPTGSVGGSMNHFPAQPVHPRSITPGGGNQILR

VDAAAANSRTVSPALGTATMPPPSANPLSPEIPLPTQLKVRVNCESGNYVTLVVAFNITYQSLVDRIDAKLARFTTSSISKSMLKLRYRDEDGD

YVAIEGDDDIQIAFMEWREGVRNMYSGGVGEIELFCVGDTA

>An-Cdc24: GenBank No. XP663196

MADTAGLNPGGPVAEDNIINRRGNEGIYQSCVNLKKRLAEVPGFEPYLREMEEEDLAQGNSDPVASLWNYLRHGYPLLAIYNASDPGAPLEIDT

SKVPEARRPKAATFKFLQAAIQEMAFPQQECFLITDLYGENTIGFTKVIKMVNRVLDILEIQGQLKKPSDTAMAAPAAGRKLTKREHILKELLE

TERDYVHHLQNLQALKKELEDTGALTGDASHQIFLNLNNLLDFSQRFLIRLEQHYARPEEQQNWGELFIQHEEAFRQYEPFIANQMRCDKTCQK

EWDKIQAAPRSPDLQQMVAQPATLNGFFVKPFQRLTKYPLMLSELRKQIEDPDLQADISRAIDSIQSVLDAANDAIDKEQLAAAFVELDERVDD

WKALKIETFGELLRFGTFTVIKNDNNKDSEREYHIYLFERILLCCKDINPNKQKSRLVGGSKDKPNTSKGKPRLVLKGRIYMANVTDIVWLQKP

GSYRIQIFWKGDPGVVDNFIIRYQNEDTMRKWYKDINTQREIQAEQRSARNTGTSDSEFTYMKSLSNIPNPYQQEYDVEEQSTKEAAFFSEFPM

SRNASSTSLRTRSATGGSGSSGPPLSTSRPRYPAMPDSTLSVHTQFPGGSMSPGERNGNSYFSPTESARSSSQSAGYPYNRQVTPVTPWGDDNN

RYTAPALSRATSRDGSNSGYFNGAPPNGRSAQRPSLPPMSGSNQSSNSASQRMRSASSPDIHHHNPESRRYMGVHTMQTVDNVPVPPIPAHMAN

MKAPVNRSQNNSPTNQSLPIRTNTSHAFHEPQYSDGRAAAPLSDQPTSPLSHEPEEEPFMPTQLKAKVNFDENYVTLVISSNIGFRTLTDRVDA

KLARFTNRSIGSKTVRLRYQDEDGDFVTIDSDEAVQLAFVEWKEQHREELARGQVGEIQLFCQPIEN

```
Mg-XP364852     MNQASRRQPGLNAPRDTKLAGSVQSNDVNQQPLLPTPAPKPPDQSLPGPSRRSASENIGV 60
Fg-XP390087     ------------------------------------------------------------
An-XP663196     ------------------------------------------------------------


Mg-XP364852     GHPTCPVAFTPPNALAMAYAPLLRTNTTPIFPSSTGGVARLQGTMGSTGPAVGIPPLRAS 120
Fg-XP390087     ----------------MNHAP------------------------------------RAS 8
An-XP663196     ------------------------------------------------------------


Mg-XP364852     QLSGTTLHN-STTSLSSLASASTVVPAQNGGQVVATSNIINQKADASRSLYQICMSLKQR 179
Fg-XP390087     QLSGSTAYTGSSASLSSLATATTITPT-NGGPVHATANIINQKADASRSLYQICISLKQR 67
An-XP663196     -----------------MADTAGLNPG----GPVAEDNIINRRGNEG--IYQSCVNLKKR 37
                                  *      *        *  ****         ** *  ** *
                                    CDC24
Mg-XP364852     LAQVPGFEEYMSLMDQWEAEDPEGGIVESVWKLLRMGTPLIVIFNLLKPENPMSFDPTP- 238
Fg-XP390087     LAQVPGFGPYLDELDPTDPVDP-------LWNLFRSGYPLLLIYNALRPNEELKVDDSSS 120
An-XP663196     LAEVPGFEPYLREMEEEDLAQGNSDPVASLWNYLRHGYPLLAIYNASDPGAPLEIDTSKV 97
                ** ****  *                    *   * * **  * *   *      *

Mg-XP364852     --DAKKAKMYIYKFAEQCKNELGIED--IFTISDLLKNDTTGFVKVTTVVNQVLDIAQTR 294
Fg-XP390087     ANEAKKSKIAIFKFVQACMKELEIPSTQSFVITDLMGTDTSGFVKVTQVVNYVLDRAEER 180
An-XP663196     P-EARRPKAATFKFLQAAIQEMAFPQQECFLITDLYGENTIGFTKVIKMVNRVLDILEIQ 156
                   *   *    **      *        * * **    * ** **   ** ***
                                        RhoGEF
Mg-XP364852     GMLIQQQPYPEDDMPDSGKSKMSYRDYIVRELVDTERKYVQDLENLHDLKKALTERGIVT 354
Fg-XP390087     GYLMQAQPDVESNEPTGG--QMTYRDHIIRELVDTERKYVQDLENLHDLKKTLEQQGEIP 238
An-XP663196     GQLKKP-SDTAMAAPAAG-RKLTKREHILKELLETERDYVHHLQNLQALKKELEDTGALT 214
                * *           *  *      *  *  **  *** **  * **  *** *   *

Mg-XP364852     GDVAYQIFGNIDAILDFQRRFLIRVETTNSMPGEVQQWGSPFVAMEASFDPIYTPFIANQ 414
Fg-XP390087     GDTLHQIFFNINAILDFQRRFLIRVETTNSMPALNQRWGAPFVHYEDALIDIYQPFIANQ 298
An-XP663196     GDASHQIFLNLNNLLDFSQRFLIRLEQHYARPEEQQNWGELFIQHEEAFR-QYEPFIANQ 273
                **   *** *    ***  ***** *     *   * **  *    *      * ******
```

```
Mg-XP364852     RKAALIAQSEFDKIKSIE-----HPVACDFNTLDGFLLKPMQRLVKYPLLLKDLLKKSED 469
Fg-XP390087     RKAAQVANQVFDKIQRSS-----HPVAADYNTLDGFLLKPMQRLVKYPLLLKDLNKKTED 353
An-XP663196     MRCDKTCQKEWDKIQAAPRSPDLQQMVAQPATLNGFFVKPFQRLTKYPLMLSELRKQIED 333
                           ***                 ** **  ** *** **** *  * *  **

Mg-XP364852     DKVKDDLSLGIAAAERTLQKANEAVDRDLLDDALEDLKTRVDDWKNHQVDQFGSLLLHGV 529
Fg-XP390087     DEVKMDLTTGCEAAERVLSKANEAVNRDLLDEAVEDLTSRVEDWKSHKVEQFGKLLLHGV 413
An-XP663196     PDLQADISRAIDSIQSVLDAANDAIDKEQLAAAFVELDERVDDWKALKIETFGELLRFGT 393
                     *           *  ** *     *  *   *  ** ***       ** **  *

Mg-XP364852     HTVLTG--KSESERDYEIYLFECILLCCKEISSTKSKDKKDKTKSTGPKIRNKGAKLQLK 587
Fg-XP390087     YGVITG--KTDQEKDYEIYLFESILLCCKEISSSKSKDKKDKLRSGGPKARNKSAKLQLK 471
An-XP663196     FTVIKNDNNKDSEREYHIYLFERILLCCKDINPNKQKSRLVGGSKDKPNTSKGKPRLVLK 453
                  *         *  * ***** ****** *   * *          *        * **

Mg-XP364852     GRIFMTNVTEVLSFAKPGSYTVQIWWKGDPG-VENFIIKFQNEEMMKKWAAGLDQQRKAS 646
Fg-XP390087     GRIFMTNVTDIVSFTKPGSHSVQIWWKGDPG-VENFIIKFLNEETLKKWVNTLETQRKHN 530
An-XP663196     GRIYMANVTDIVWLQKPGSYRIQIFWKGDPGVVDNFIIRYQNEDTMRKWYKDINTQREIQ 513
                *** * ***      ****   ** ****** * ****   **    **      **

Mg-XP364852     G-SQSATSPDQPTAEFAWMRSQSA-VENPYAQTQPEEEEE-YEFGAIPQQSQQYQAMAGT 703
Fg-XP390087     V-PRQSTNSDTLSTDFAWTRDQVAGLENPYLKENDDEDDEDIGPATAPAGFPGVTHPMSL 589
An-XP663196     AEQRSARNTGTSDSEFTYMKSLSN-IPNPYQQEYDVEEQS-TKEAAFFSEFP-------- 563
                               *           ***      *

Mg-XP364852     MPRMNSTASIRGRSQTGESSQSIASIARGPPPRFPLPQPPQPLSVQTQLQGG-NSPGFRP 762
Fg-XP390087     GPRTASSNNLRARAGTGESSASLAGMVRAPPPRFPLPAPPGSLSLQTQPNGA-HSPSAWA 648
An-XP663196     MSRNASSTSLRTRSATGGSGSSGPPLSTSRP-RYP-AMPDSTLSVHTQFPGGSMSPGERN 621
                  *  *    * *  ** *  *        * * *   *   **  **  *   **

Mg-XP364852     GDSYFSPIGESPVSSRTSTASNVFPGSAGYGSFKGVTGQPYPGMWDGPIVDQNRYTAPAM 822
Fg-XP390087     GDSYFSPVTESPASTRTSTTSGMFS-TPQYGFPKSAT--PNPQQWED--ANGNRYTAPAM 703
An-XP663196     GNSYFSPT----ESARSSSQS------AGYPYNRQVT---PVTPWGD---DNNRYTAPAL 665
                * *****      * * *  *        *      *       *       *******

Mg-XP364852     PRAPSRDGPSPVNAYGMSGANGRNPRGPSMPVMASHSAQGASQQSRSRSYSTPDIQGQGP 882
Fg-XP390087     PRAPSRDGPSPN--------PARNPRGPSLPAMSSSSQAALAAQQRNRSYSTPDING--- 752
An-XP663196     SRATSRDGSNSGYFNG-APPNGRSAQRPSLPPMSGSNQSSNSASQRMRSASSPDIHHHNP 724
                 ** ****              *    ** * *            * ** * ***

Mg-XP364852     PGMRRTPGGSQSNVPAVPGIPAHLHAAHERHDSNIPRSNTGSPALSLRNDLPMRSNTQSP 942
Fg-XP390087     PGMPRTRQPSHGNIPAVPGIPQHLHPGHN---PNLSRDQTGSP----RNDQPTRAQTNSP 805
An-XP663196     ESRRYMGVHTMQTVDNVPVPPIPAHMANMK--APVNRSQNNSP---TNQSLPIRTNTS-- 777
                                **  *   *           *    **        * *  *

Mg-XP364852     GVQRDRLMQQGGGYSGGTLAQFPMQPVYPRGGTPNPPGSGHTPAPLNLSADMGRTVSPPL 1002
Fg-XP390087     GAQRERMHKPTG-SVGGSMNHFPAQPVHPRSITP---GGGNQILRVDAAAANSRTVSPAL 861
An-XP663196     -----HAFHEPQYSDGRAAAPLSDQPTSPLSHEP-------------------------- 806
                               *        **  *    *
                                         PB1
Mg-XP364852     GTGTPQPPNTAGLMSPDQGGGIMPTQLKVKVNYDTSNYMTLVAAYNITYRSLVDRIDAKL 1062
Fg-XP390087     GTATMPPP-SANPLSPEIP---LPTQLKVRVNCESGNYVTLVVAFNITYQSLVDRIDAKL 917
An-XP663196     ---EEEP--------------FMPTQLKAKVNFD-ENYVTLVISSNIGFRTLTDRVDAKL 848
```

```
                       *                *****  **    ** ***   **    * ** ****

Mg-XP364852      ARFTNSSISRGNLRLRYRDEESDFVSIVCDEDVQMAFTDGRNGDRDMYN-GGVGEVELFC 1121
Fg-XP390087      ARFTTSSISKSMLKLRYRDEDGDYVAIEGDDDIQIAFMEWREGVRNMYS-GGVGEIELFC 976
An-XP663196      ARFTNRSIGSKTVRLRYQDEDGDFVTIDSDEAVQLAFVEWKEQHREELARGQVGEIQLFC 908
                 ****  **      *** **  * * *  *   * **       *     * ***  ***

Mg-XP364852      VGIGGE 1127
Fg-XP390087      VGDTA- 981
An-XP663196      QPIEN- 913
```

CBSn-PB1 protein

>Mg-CBSn-PB1: GenBank No. XP361856

MSASRQKQTKRDEAIRRKMENDLAKKKHVTGRARHSRKAPPGTVLALKPSQALQIKPGTTVAEAAQLMAAKREDCVLVTDDDDRIAGIFTAKDL
AFRVVGAGLKATNVTIAEIMTKNPLCARTDTSATDALDLMVRKGFRHLPVMDENQDISGILDITKCFYDAMEKLERAYSSSRKLYDALEGVQSE
LGASQPQQIIQYVEALRTKMSGPTLESVLNGMPPTTVGVKTSVKEAAQMMKENHTTAVLVQDQGQITGIFTSKDVVLRVIAPGLDPGNCSVVRV
MTPHPDFAPMDMSIQAALRKMHDGHYLNLPVMNDSGEIVGMVDVLKLTYATLEQINTMSSGDNEGPAWNKFWLSLDNETESMVSGDGSHHHSHS
HIHHRSVMSPDLTRERLDSVAPGDSASHAGVESPGHSIVQHSPELPVSEIPFPFKFKAPSGRVHRLQVIASHGVAAFITNVTSKLGGEIEAVGG
APEVTEDGQVRGGFALSYLDNEGDSVSITADQDLLEAILLARQSGRDKVDLFVHDPEKPPVAVAAPAAVPPAPSEPVSVPIPTPPASSVARERR
RRKSPASDESDEEEESEDDGSSTLRRPRRHQRAAAAQQEPLIAGVPNELLLPGAIVTLAVVIVGVFTISRISSNNR

>Fg-CBSn-PB1: GenBank No. XP386485

MSGHSHNTHRSTPNKGRGAVPFANSPSGGGGGGSSNIPRPVLEPTPPAETGSSFSASRQKQSKRDEAIRKKLENDLSKKKHLTSRARHSRKAPP
GTVLALKPSQALQIKPNTTVSEAAQLMAAKREDCVLVTDDDDRIAGIFTAKDLAFRVVGAGAKASAVTIAEIMTKNPLCARTDTSATDALDLMV
RKGFRHLPVMDENQDISGVLDITKCFYDAMEKLERAYSSSRKLYDALEGVQSELGSTQPQQIIQYVEALRSKMSGPTLETVLNGVPPTTVSVRT
SVREAAQLMKENRTTAVLVQDQGAITGIFTSKDVVLRVIAPGLDPANCSVVRVMTPHPDFAPMDMTLQAALRKMHDGHYLNLPVMNDGGEIVGM
VDVLKLTYATLDQINAMSNNNDEGPAWNKFWLSLDAETESMMSGEGSQAQHTNLGSRLTSPDMVRDRLNDTVAPGDSASHVGMESPPRSILPDV
LEHQLPEELPFPFKFKAPSGRVHRMKIVATDGIEAFVEAIASKLGAEADNIGGVPDVEDGKIVGSGFALSFLDDEGDSISITADHDLLEAVILA
RQAHHDKVDLFVHDPEKPPVSAADPRHPATPSVSTGAGLRERRKWWPEEEEEEDDDDSEDEHPARRRKSRAAHTHAHAHEEQIIAGVPNELILP
GAIVTLAVAIVGVFTIARLTSR

>An-CBSn-PB1: GenBank No. XP663320

MSSTPTFRGTTSHRTVGRGRLPDFEGGSSASHIPRPRPESSSTITSHNPHTPSSDIGSSTMSAASSRQRQNQSKRDEAIRRKLEADLNKKRSNP
ARANRTRKAPPGTVLALKPSSALQIKPSTTIAEAAQLMAAKREDCVLVTDDDDRIAGIFTAKDLAFRVVGAGLKARDITVSEIMTKNPLCARTD
TSATDALDLMVRKGFRHLPVMDENQDISGVLDITKCFYDAMEKLERAYSSSRKLYDALEGVQSELGSSQPQQIIQYVEALRSKMSGPTLESVLD
GMPPTTVSVRTTVKEAAALMKEHHTTALLVQDQGSITGIFTSKDIVLRVIAPGLDPATCSVVRVMTPHPDFAPSDMSIQAALRKMHDGHYLNLP
VMNEGGEIVGMVDVLKLTYATLEQINSMSTQDDEGPAWNKFWLSMDHESDSMVSGSQSHQPHRSIVNPESPKASFDARDSVLPNESASHHGGDE
HSEFHHGELSPFPFKFKAPSGRVHRVNILPAAGIAELVAQVTAKLGPEVEAVGGAASCADGVLSNTGYALSYVDNEGDTVSITTDQDLVDAVYI
ARHARRDKVDLFVHDPAQPPVIPAPVEPAPVKPVEVKTPASDDQLSEESPIPKPRATQAYPAHPPEEQLIAGVPNDLLLPGAIVTLAAVIAGVF
ILSRATSR

```
MG-XP361856     ------------------------------------------------------------
Fg-XP386485     MSGHSHNTHRSTPNKGRGAVPFANSPSGGGGGGSSNIPRPVLEP----------TPPAET 50
An-XP663320     MS--STPTFRGTTSHR--TVGRGRLPDFEGGSSASHIPRPRPESSSTITSHNPHTPSSDI 56

                                                                       (CBS)n
MG-XP361856     ----MSAS----RQKQTKRDEAIRRKMENDLAKKKHVTGRARHSRKAPPGTVLALKPSQA 52
Fg-XP386485     G-SSFSAS----RQKQSKRDEAIRKKLENDLSKKKHLTSRARHSRKAPPGTVLALKPSQA 105
An-XP663320     GSSTMSAASSRQRQNQSKRDEAIRRKLEADLNKKRSNPARANRTRKAPPGTVLALKPSSA 116
                     **     ** * ******* * * ** **     **   ************** *

MG-XP361856     LQIKPGTTVAEAAQLMAAKREDCVLVTDDDDRIAGIFTAKDLAFRVVGAGLKATNVTIAE 112
Fg-XP386485     LQIKPNTTVSEAAQLMAAKREDCVLVTDDDDRIAGIFTAKDLAFRVVGAGAKASAVTIAE 165
An-XP663320     LQIKPSTTIAEAAQLMAAKREDCVLVTDDDDRIAGIFTAKDLAFRVVGAGLKARDITVSE 176
                ***** **  **************************************** **   *  *

MG-XP361856     IMTKNPLCARTDTSATDALDLMVRKGFRHLPVMDENQDISGILDITKCFYDAMEKLERAY 172
Fg-XP386485     IMTKNPLCARTDTSATDALDLMVRKGFRHLPVMDENQDISGVLDITKCFYDAMEKLERAY 225
An-XP663320     IMTKNPLCARTDTSATDALDLMVRKGFRHLPVMDENQDISGVLDITKCFYDAMEKLERAY 236
                ***************************************** ******************

MG-XP361856     SSSRKLYDALEGVQSELGASQPQQIIQYVEALRTKMSGPTLESVLNGMPPTTVGVKTSVK 232
Fg-XP386485     SSSRKLYDALEGVQSELGSTQPQQIIQYVEALRSKMSGPTLETVLNGVPPTTVSVRTSVR 285
An-XP663320     SSSRKLYDALEGVQSELGSSQPQQIIQYVEALRSKMSGPTLESVLDGMPPTTVSVRTTVK 296
                ******************  ************* ******** ** * ***** * * *

MG-XP361856     EAAQMMKENHTTAVLVQDQGQITGIFTSKDVVLRVIAPGLDPGNCSVVRVMTPHPDFAPM 292
Fg-XP386485     EAAQLMKENRTTAVLVQDQGAITGIFTSKDVVLRVIAPGLDPANCSVVRVMTPHPDFAPM 345
An-XP663320     EAAALMKEHHTTALLVQDQGSITGIFTSKDIVLRVIAPGLDPATCSVVRVMTPHPDFAPS 356
                ***  ***  *** ****** ********* ***********  ***************

MG-XP361856     DMSIQAALRKMHDGHYLNLPVMNDSGEIVGMVDVLKLTYATLEQINTMSSGDNEGPAWNK 352
Fg-XP386485     DMTLQAALRKMHDGHYLNLPVMNDGGEIVGMVDVLKLTYATLDQINAMSNNNNDEGPAWNK 405
An-XP663320     DMSIQAALRKMHDGHYLNLPVMNEGGEIVGMVDVLKLTYATLEQINSMSTQDDEGPAWNK 416
                **  *******************  ***************** *** **    *******

MG-XP361856     FWLSLDNETESMVSGDGSHHHSHSHIHHRSVMSPDLTRERL-DSVAPGDSASHAGVESPG 411
Fg-XP386485     FWLSLDAETESMMSGEGSQAQ-HTNLGSR-LTSPDMVRDRLNDTVAPGDSASHVGMESPP 463
An-XP663320     FWLSMDHESDSMVSGSQSHQPHRSIVNPE---SPKASFDAR-DSVLPNESASHHGGD--- 469
                **** * *  ** **  *              **        * * *  **** *
                                  PB1
MG-XP361856     HSIVQHSPELPV-SEIPFPFKFKAPSGRVHRLQVIASHGVAAFITNVTSKLGGEIEAVGG 470
Fg-XP386485     RSILPDVLEHQLPEELPFPFKFKAPSGRVHRMKIVATDGIEAFVEAIASKLGAEADNIGG 523
An-XP663320     -----EHSEFHHGELSPFPFKFKAPSGRVHRVNILPAAGIAELVAQVTAKLGPEVEAVGG 524
                        *        **************       *          *** *    **

MG-XP361856     APEVTEDGQVRGGFALSYLDNEGDSVSITADQDLLEAILLARQSGRDKVDLFVHDPEKPP 530
Fg-XP386485     VPDVEDGKIVGSGFALSFLDDEGDSISITADHDLLEAVILARQAHHDKVDLFVHDPEKPP 583
An-XP663320     AASCADGVLSNTGYALSYVDNEGDTVSITTDQDLVDAVYIARHARRDKVDLFVHDPAQPP 584
                            * ***  * ***  *** * **  *   **    **********  **

MG-XP361856     VAVAAPAAVPPAPSEPVSVPIPTPPASSVARERRRRKS-PASDESDEEEESEDDGSSTLR 589
Fg-XP386485     VSAADPRHP------------ATPSVSTGAGLRERRKWWPEEEEEEDDDDSEDEHPARRR 631
An-XP663320     VIPAP----------------VEPAPVKPVEVKT-----PASDDQLSEE-------SPIP 616
                *  *                   *               *
```

```
MG-XP361856       RPRRHQRA-AAAQQEPLIAGVPNELLLPGAIVTLAVVIVGVFTISRISSNNR 640
Fg-XP386485       KSRAAHTH-AHAHEEQIIAGVPNELILPGAIVTLAVAIVGVFTIARLTSR-- 680
An-XP663320       KPRATQAYPAHPPEEQLIAGVPNDLLLPGAIVTLAAVIAGVFILSRATSR-- 666
                    *      *    *  ****** * *********  * ***   *  *
```
