# Supplementary material for: Molecular evolution of Phox-related regulatory subunits for NADPH oxidase enzymes
Source: BMC Evol Biol. 2007 Sep 27;7:178. doi: 10.1186/1471-2148-7-178 (PMC2121648; doi:10.1186/1471-2148-7-178)
Supplement: Additional file 13 — Amino acid sequences of NOXR, Bem1, Cdc24, and CBSn-PB1 proteins of other fungi than M. grisea, F. graminearum, and A. nidulans. Amino acid sequences Bem1, Cdc24, and CBSn-PB1 proteins of S. cerevisiae, C. albicans, Y. lipolytica, S. pombe, C. cinerea, L. bicolor, P. placenta, P. graminis, P. blakesleeanus, and B. dendrobatidis are provided. [file 1471-2148-7-178-S13.doc]

**Additional File 13**

**Amino acid sequences of NOXR, Bem1, Cdc24, and CBSn-PB1 orthologs of other fungi than *M. grisea*, *F. graminearum,* and *A. nidulans.***

Sequences were obtained from the indicated servers: http://www.ncbi.nlm.nih.gov/ (GenBank^TM^), http://genome.jgi-psf.org/euk_home.html (Doe Joint Genome Institute), http://www.broad.mit.edu/cgi-bin/annotation/fgi/blast_page.cgi (the Broad Institute Fungal Genome Initiative database). The following naming was used to describe species of sequences: Sc (*Saccharomyces cerevisiae*), Ca (*Candida albicans*), Yl (*Yarrowia lipolytica*), Sp (*Schizosaccharomyces pombe*), Cc (*Coprinopsis cinerea*), Lb (*Laccaria bicolor*), Pp (*Postia placenta*), Pg (*Puccinia graminis*), Pb (*Phycomyces blakesleeanus*), and Bd (*Batrachochytrium dendrobatidis*). Fungus-Bd-Cdc24-like protein (FGI database No. BDEG_01878.1) shown here contains rhoGEF motif, but does not possess Cdc24 motif and PB1 domains. Therefore it is unclear whether the gene belongs to Cdc24 family.

>fungus-Yl-NOXR: GenBank^TM^ No.XP_501089

MLTLQQVVTWLKGVELYSKGDYAPALKQFNQLINSPEYSGEVSKVAHNIAAIYYKMEDHERATKWFVTALQKDPYLCITHFLFGQCLFEQEKYSEAAKEWDKCLEYMRGNTWINYQPLGLNFTLYSCEVMFNKSMAKIYSGDILPGVSDLSRAGKEHKWSADHNRIDKAYIYFNAIKDSLHRATHTTKQSQNDGLQRSKSLSRSKSQNLSRSKSLARSKSMKHKQQQLQDNIRNRSSMGFGANYTNTFDVSQAEKEAAKQLAQQSSGNVGPVGLGIRQSPSMRGSPANRPSPNFNNNGGTPNLGRSQTLGGGGHTQSSQLHRSQTTGHAHSVAEHPHSSANVHRSQSTRAPVTGTSTTEVDLPSTVKMARMNETYKIYQISSEFCLFSIHDNRLKNIVGCEQMASLLRGKDKEISSGGGVVGASSPPPEDSTEFSFVESVGRSDDVDDIYDIDDYYGTPPASTGQRSANQSVNRSGGSIKSNAATVATGSPPARSKPTPIDVDLAREMRKVSFDSSLSTPKLNKLVDKIQGTTRDSVSSTDSIHAPKATKHRVQINYIRPVTAKVCSFVAEECSSPDTPITPIPVKKKAVKDYSTRSPYPDSVASSSYEDETTSWSKSLPLLPNDHAAHKTDQRLRELRERDRREIRDGFIDRDTGMTPPQSHQFDPTNTVAPGRSRNTKFLTVDLDDPNHQGNNGRSKSPGSPMSPFILVT

>fungus-Cc-NOXR: GenBank^TM^ No.EAU91521

MSLKAELETWAAALKAYDDQDFDKALQLFSEIADNSKIHTNIGLIYATLGEHEEAVKHFIEATGMDQYLAVAYFQCGVSNFLLQRYELALKDFEEALLHLRQNQTMNVSIDDGLNSNYEQLGLQFKLYSAEVLFNMGLSLIFMGQIEMGLQNFEAARREKATEDHNVIDEAIRDQGRGYTVFSIPVGVLYRPSEKKLKNAVQKDYMGKAKLVASSDPDDKITTFVGSARLKMGISPAGIYIDRPDIDTPAGAAVSRSATVPASSYPASRPAESIRSAGLERSKTTINVPSNARALTSPQSLSPSSRLPPVSSAGALGRSNTQITPGRPSPSAGMGGPSRVRHPSFPDRIHADMTKADLPDVPAPERITEFYDDYLDSYEDQPPVPQLSAPPTADRVATWARQTPAYPAGVARSTSRSAPNSQYAPSSYGGGSMRRKPTRRATSRQQNRIGSTYEEEEEGYGSGDYDDGPFEMNLIRVKLHYQDETRGMTLAPDLTFNEFIVKITAKFNKSFSGLGLKFKDEDGGKVTLRDESDYELAIETARENAKGRAEGKLEICSSLYYGVQYKRKLGEKLVVLRAVHSRKANQLCLDSNMGLSGRKVKQRIGNDPRNLSWADDAARFGSNYLSRFGWDASKGLGAEGEGRTSHLKVAQKLDMLGIGAAHQKDPNGIAWKQNRDFENLLKRLNAAGGEGGAEVEVKTEVSGFVSSTKEVQVEVKKEEKEKEDEMDVDEKKERKKEKKEKKDKEKKKDKDKEGKRKRSEVDGDEESGSQKKMKTEEASPTPQSSDSTPSEQPKKAFVPRHRAHRARAIAAKSISMKSSTHISEILGIAPESSTTSAAASGSISPAPPDQGKLTVISEDVPELEKITTSTKSVADYFKERLLAKANAKAQSSGSSASTPQPSATKDEEMDEAEEKPRGGLGFARVQWESQESTSVQSDTLRMGLSKFSSLMSSTFLSSTFSSSTSESTSPATEEKKETGEEDEKETEEQRKERKRLKKEEKARKKAEKEKRKAAKAEAATSEAKGGDAEDAGKEREKESKKKKKSKKEKKT

>fungus-Lb-NOXR: JGI database jgi|Lacbi1|338858|estExt_fgenesh3_pg.C_50277)

MSLKAELETWAAALKAYDEEDFEKSLDLFSRIADSSKILTNIGLIYATLGEHEAAVQRFIEATNFDQYLAVAYFQCGVSNFLLARYELAYKDFEEALLYLRGNQAMYVVFMVEPYLPYSSIPAHRNYEQLGLKFRLFSAEVLFNKGLAQIYMGRAQEGLADMEEARREKATDEHNVIDDAIQERGEGYTVFSIPVGVLYRPSEKKLKNSMQKDYMGKAKLVAASDPNDIFTTFTGSTRLKQGISPSGVFIDRPDIESAVIPSVTRSASVPSSTAPSRQPADGVRLAGVERSRTAMNPPQSARLASNKGPPPRPPMSAGASIGRSNTNITPSRPSPNATIGGPVRGLSVRRPGNASPGNSSPPRAPPKDDARLTEFYDDYLDSYGGDAPIPPIAQPGPDRIAAWARTNANPNYPLVRSGSRSAPTSQYTPSSYGGGGSLRRKVTKRNNPRAPSRVQSTYEEEEEGYVSGEYEDGPFELTLIRVKLHYQDDTRGMTLTPDTPFADFMDKVTAKFGKQINGLGLKFKDEDGGKVTLRDESDYELAIETARESAKGKPEAKFFDSFSLTHCFLIEMGLGGRKIKQRIPDDPRNLSWADDAARFGSNYLSKFGWDASQGLGVDGEGRTSHIKVSHKLDMLGIGAAHQKDPNGIAWKQNKDFENLLKRLNENATEIVDGKEEQDVVKEETPKKKRKQKGDEEKDTKKKRRKTEDSSPSADEVVPDSLPADPAPVRVAPRYRAHRARAIAAKSITSKSAAAISEILGIAPSSGLSVTTTQTTGKLTSLTDDATIEKLTTSTKSVADYFKEKLLVRATQSESTTPISEQDAYDLPRSGLGSGRARFEIQEDDSEMGARRVGLSKFSSLMSSQFLAATSTLSTPPEKTQSEDVATTQLDMVEEEQTSEEKRKKKKGDKDEKKEKKSKDKVTRTEVKDPDTLQTASLDDVDRKARKAQRKAEKKARKAAENNLH

>fungus-Pp-NOXR: JGI database jgi|Pospl1|127073|estExt_fgenesh3_pg.C_330066

MSLKAELETWASALKAYDEENFDKSLQLFSEIGDSSKILTNMGLIYATLGEHDAAVEQFTAATQLDQYLAVAYFQCGVSNFLLGRYETSLRDFDDALLYLRGNGDINYEQLGLKFKLYSAEVLFNKGLAHIYMGQVDQGLAELQEARKEKVTPEHSVIDDAIADRGDGYTVFSIPVGVLYRPAEKKLKNAKTKDYMGKAKLIATEDASEAFTEFTGVARMRQGVSPTGATDFGPGGLQRAATVSNPTPPPKSDDYVRSAALQRANTTINVSVDRFRSNPASPASPATSPGSGPPSAPALSRNASFAGRMQPTRGLSIRKPGTDAPPPSTRGMRTTEFYDSYLDSYADAPSALPPVPEARPAPAAVRRAPSRARAAPQSTYSNGSVRRRPTRRGTVRSRYEEEEEEGYGSGEYEYEREEDGFVKIRVKLHYADDVRGMALSPGMPWEEFLERVTGKFGRELDGLGMKFKDEDGGMVSLRDEMDYELAIETAREQAKGKPEGRLEIWCTDE

>fungus-Pg-NOXR: FGI database PGTG_05816.2

MSLKAEIETWATALELYDQQNFDDALAAFEDIADSSKILFNIGLIYATLGEHEVAIENFRTATSLDQYLAVAYFQCGVSNFLLGMYEEAVKDFEDALMYLRGNLTIDYSQLGLKFRLYSCEILFNKGLSLIYQGAEAEGMIDLQEARKEKQTAEHSVIDDAIADRAEGYTVFSIPVGVLYRPAMSKVKNLASKNYLGQAKLVAATDASETFVGFTGSARAAAKTTTTGPVMSTPIEQAPMPPAPGMLRRGTVATAGSGMGDGPLGPAGMLRRGTSAAITPMMPSSSSGDSPTVGIIRRGTTAARIESTAARPAPVPTNNDLRRRPSEAGSTARLNRSATASSGSKPTSRLAPMAEQREPEKSPRYGSRTRGSLGTESYEDYEDNEPAVPSRSGGPSEDRTTNWARNLSTSGSSSGRNRDPPAVRRPPPANSPYDDPPSDAEYPETVMSGGYAEMTKIRVKLHYGSDTRGMLSHAMIRGFLGKSDQEVFLKSRSSGDEIQGREGSMVSIMDDDDWESAVETARSYAKGRAEGKVEIWVEDAGFL

>fungus-Pb-NOXR: JGI database jgi|Phybl1|79899|estExt_fgeneshPB_pg.C_350109

MEFKLELDQWLNACAAFDSKDYETAVQSFLSMADNAKMHFNIGLIFAIEDDHQRALAAYSRAISMDAYFAVAYFQKGVSQFVMNNMEAAMHDFDNAYQRLRGNEMINYTQLGLAFRLYACEVLFNRGVCQLYLGKIDAGLTDLYHAQKAKMTEEHDVIDQAVRDRGRGYSVFSIPPGVLYRPSEARMRQLQNANMFAAVDKLSLPNKNFKTALTSIQRNNSILLPDARFQKRVPGAQAAPRQVQQPPVISSLAEHGMRAIARESSRPKMATHIYTDQLPPPTPVSSVSSHYPRKQNTRQYSPIERQRNDSNAGSFSTEEWADSPHPSISSTSSFSSSLRQRADGRRVDSGFESTHEERYSSSSGRSSTKSHKANRYSPPPVPPIPHNASYEYGDTSANYGNFDLDEMYGSLPTIDTQDNKQDRVRPRLNSNNTAPLEDDRRGNDQINNNDYSNIRAHPPQPTASVSTPTLKEGSINTKIRVKVHYTDTRILLVPHTITFNELLGRVREKFGAPPSTRLQYKDEEEEMVLMIDDDDLHLVRQTSRQKHGDFGVEKIEIWCVT

>fungus-Bd-NOXR: FGI database BDEG_00032.1

MALKEQLAQWSKALDYWDTNDFFGAYEQFVSFADFAKIHYNVGMCAMRTNNLDEAIAAFSRAITLDEYLSVAYMQIGICQYHMGEFDEALANFEESFKMLRGNFFIDYTQLGMEFQVFACHAIFNIALCHLQRGDTERGVRYISEAIKAVPVDSDFVPDIADIEEAERLGENAADTIMPYEVPPNLVFRPQEDNLRNAERVDYLGQSKVVASVNATDNYTGFSGKLTRDLTLLSRSKKKDANSGAAKEAALKSPASFSAAQTTIAQSNTIRVNRKPSVTSISREPIQEGYEQTITMSRSNSATTSRKYIPRRLSSNNISANTIPGASRVPGTLSRAPSAPLSREGSGSGQQLEFPLRTLRSAGGSSRMGLGDTQNNVDTAAERLNSLTINNNDLPASPAIKLTTSNRSNVSTDSIKVKCHYIESRFIVVPINISFRDLRERVSAKFGVQGLLSLKYKDEEEFMTMMVNDDDLDEAVAVSAQGRLELWCFLLDPPK

>fungus-Sc-Bem1: GenBank^TM^ NP_009759

MLKNFKLSKRDSNGSKGRITSADISTPSHDNGSVIKHIKTVPVRYLSSSSTPVKSQRDSSPKNRHNSKDITSPEKVIKAKYSYQAQTSKELSFMEGEFFYVSGDEKDWYKASNPSTGKEGVVPKTYFEVFDRTKPSSVNGSNSSSRKVTNDSLNMGSLYAIVLYDFKAEKADELTTYVGENLFICAHHNCEWFIAKPIGRLGGPGLVPVGFVSIIDIATGYATGNDVIEDIKSVNLPTVQEWKSNIARYKASNISLGSVEQQQQQSITKPQNKSAKLVDGELLVKASVESFGLEDEKYWFLVCCELSNGKTRQLKRYYQDFYDLQVQLLDAFPAEAGKLRDAGGQWSKRIMPYIPGPVPYVTNSITKKRKEDLNIYVADLVNLPDYISRSEMVHSLFVVLNNGFDREFERDENQNNIKTLQENDTATFATASQTSNFASTNQDNTLTGEDLKLNKKLSDLSLSGSKQAPAQSTSGLKTTKIKFYYKDDIFALMLKGDTTYKELRSKIAPRIDTDNFKLQTKLFDGSGEEIKTDSQVSNIIQAKLKISVHDI

>fungus-Ca-Bem1: GenBank^TM^ EAL04135

MIKTFRKSKRSSSNSSSPKKTISRVSSTSSNQTSHDGILQSPKKVIRALYDYEPQGPGELKFFKGDFFHVLDDVDDELHKEAEANGWIEATNPMTQLKGMVPISYFEIFDRSRPTVTASSNSFTNSIDIQHQHQQGIHNGTGNRNLNQTLYAVTLYEFKAERDDELDIMPNENLIICAHHDYEWFIAKPINRLGGPGLVPVSYVKIIDLLNPNSHYTSIDTSRRSQVIQVINGFNIPTVEQWKNQTAKYQASTVPLGSISGSGTPPTSANSQYFDNHTMTSNRSSSGSSISIIEASVDSYQLDHGRYQYSITARLNNGRIRYLYRYYQDFYDLQVKLLELFPYEAGRIENSKRIIPSIPGPLINVNDSISKLRREKLDYYLSNLIALPSHISRSEEVLKLFDVLDNGFDRETDAINKRFSKPISQKSNSHQDRLSQYSNFNVLQQQQQQQQQQQYAHHSRGSDNSPTNESSGSNLINSSSHNDSSLSSSPPPPQTVTTTNTTITTDSSSKQPKVKVKFYFDDDIFVLLIPTNLRLQDLKSKLFKRLELDITYKYEKPDQQQKPTSESIHLFLKNDFEDFLIENETSNNNNSEIDFENEIIKEKLGEFEVNDDEKFQSILFDKCKLMVLVY

>fungus-Yl-Bem1: GenBank^TM^ AAM09809

MIKGIRRSLKGEKTTSSGSANSLVSDKNPLHTNPPKMVIRALYDYEPQGPGELSFCKGDFFHVISNEQDSEWFEAFNPLRNVRGMVPVPYFEVLGKKEGITSPDPNEALLPEERKALHRSSQSKHAQHSPQHVLANGLDVSQSMQNLSLGGASGSGGHTREPSMAGASDRSSQGKSTPTLHGVVLYDFQAERPDELQASKGDNIIIQAQSNHEWFVAKPVGRLGGPGLIPASFIEIRDIVTGKAVVDVESALRASGLPKVEEWKRMAADYKASSIALGNFDDPQQHSQQQALQHALRGQQTLQEHQVQQEHQTLQERQSLQEQQALREQQALRENYAAPATGPLVVAASVESFALHSGRYWYLVNAELEDGSMRRLCRYYQDFYVFQINLLGVFEEAAGHNDQPRLLPFMPGPLTYVNDSISMQRRHNLDEYVRKLMALPAYISRSVIMQELFALRPGDVAAAGPTDDLPQPLTPETESFDAAQAEARAKGIHPKSLGPTNVSSSSVSYSEGTEESRPVSTASGLMGPPEPTGLPGGGHQRMTSVSTVGPTPDNPAVNTPQSSSNSVKVKVFYQDDLIAIRVPSDIDYATLHDRLCERLRVETLSLLYKADDGSRVVISNDEDLGAAVYGKNKLVLYAS

>fungus-Sp-Bem1: GenBank^TM^ CAA93608

MLKIKRTWKTHSRILDKDPFSIEPPRKVIRALYDYTARKATEVSFAKGDFFHVIGRENDKAWYEVCNPAAGTRGFVPVSHFEEIGKTVKSERDSDGSGQISFTDLTTNSSTTRSSISELHSGSQPLFGIVQFDFAAERPDELEAKAGEAIIIIARSNHEWLVAKPIGRLGGPGLIPLSFIQLRDLKTGAVIKDVSEAVLRISCIPRVEDWKRAAADYKKSSIPLGKFSDGETQTMPSLSPSTENLQINNDVTYQAATDNSSTFPGSVANELTPLQTLESRTASIASKNKKDMSSEPTVVAAMVENYMIRDDQYWYLVRAVMSDGKHRNLCRYYEDFFNFQTKFLELFPNEAGRGDERRVIPYMPGPVDDVNELISSQRAMDLDVYLKEMCRLPARLLENELVKLFFLPLDGDVESPHPTSTMPEALPREPLSFSLPEKAPEKATNISIPESAPTTAGSTCKVKVRLGDETFALRVPSDISFEDFCERLTNKLGECEHLSYRDTNANKVLPLNNVDDLRKACSQESGVLLFAERRRF

>fungus-Cc-Bem1: GenBank^TM^ EAU84301

MKSLRKSLNGNKDSSKLQISTPLPIPTMSKPPSAILPPQKVIRANSSYKSQAPQELSFQKGDFFYVLKDAEGSAWYEAHNPVTGARGIVPRAMFEEFGKNAPPVRHSQFSTPGGGGFKSMHLPGSGSAPSPPTPKHQVYYAIVMHDFSAERPDELDAKRGDAITVVAQSNREWFVAKPIGKLGRPGLIPVSFVEIHDPTTGAPIVDVDALIDRGDLPKVEDWKRAMLNYKQNSISLGVIDPPSRGGPPTSPFLSTMAEPMDPPREPEPPRPQTPECLPEGILLSADVVSFHYEMEEYWFRVDAIFQPYSSPGSGQLPPAKQLILFRVYEDFYEFQVTLLRTFPREAGQQPPHPRVLPYMPGPAKDVDDALTATRREELDEYIHGLCALSRSGYRHILEHEVLRQFLALKPGDVDSEIDPRVDEIDALFGEYPEDDYDREYAQEIADQVGRMKVSDSGHHSDGSDYEDEGYAASPQHRPYDRHPYGQTAPVNGRHSREESLRVQAHQNHQRAGSTSSFHTHQTQHTQSYASHSRNHSSERISPISRDSYSNGQSRWTDHTQPTPTTANSFSSASRTRSQTATSVNLNTPPISAANPQTAFVKIKIFDRVADDLIAIRVHPKVSHHELMEKVQARLGSELRVLKYRDSLTNTFVGLDTDEELRAWMEGTDKHVLYAD

>fungus-Lb-Bem1: JGI database jgi|Lacbi1|151542|gww1.41.113.1

MKSLRKSLNGNKDSSSKLQISTPVPIPTISKPPSAVLPPQKVIRALFNYRSQAPQELSFSKGDFFYVLRDADDVGTWYEAHNPVTGARGIVPRNMFEEFNKNIAXTFFDSALRLWYLIPSPLRVRTSQVGPRTALPSPSRPESSIPTTPKTQVFYAIVLHDFVAERADELDAKRGDAITVVAQSNREWFVAKPIGRLGRPGLIPVSFVEIHDPSTGKAIPDVQGLMDRGELPKVEDWKRAMLNYKQNSIALGVIDPPSRGSVQSNSYSPREDYQSPPQPQAASEPARAQSPDILPEGILLSADVVSFHYEMEEYWFHQNVLPPAKQLILFRVYNDFYDFQVSLLDNFPREAGREPPHPRTLPFMPGPAQDVDDALTSTRRGELDEYIHGLCALGKSGSRNILEHLVVRKFLALKPGDMGRMRVQDDQRSDGSDYEDEGYAPSLQQKAYDRGRHPYVQGSERSSQDNTFRLQGQNHQRNNASTPSTASFRSSQAMSSRSHSHSHSTTTNLNNPPISAANPQTAFVKIKIFDRVSDDLIAIRVHPKVTHSELMDKVQARLGGEVGNLRYRDSMTNTFVGLDSNEELRSWMEGTDKHVLYA

>fungus-Pp-Bem1: JGI database jgi|Pospl1|126313|estExt_fgenesh3_pg.C_890008

MKTLRKSLNGHKDNSPSITTPLPRLSKPSGAVLPPSKVIKALQTYKANAPQELSFEKGDFFHVLNDVNNEGVWYEAHNPATGARGLVPCHMFEEFGRGPSKPRVADAGAADRPLSTVKQQAFYAIVLHDFTAERADELDAKAGDTITVVAQSNREWFVAKPIGRLGRPGLIPVSFVEVRDPATGLAVQDIMALIDGGALPRVEEWKRAMLNYKANSISLGVLEESVAANFAKAQATKQMPPLPTATPPPPSQPQPVKQKPPSPISRPISPKLLPEGILLSADVKSFHFEMEEYWFRIHAVFQPYDPSDSHSLPPAKQLVLFRSYNDFYDFQVEILNAFPHEAGRPDVNTRILPYMPGPSDQVDNEVTAARRIELDDYLHKLCELQLYSRYILEDKLIREFLALKPGDAEVEVEPRMKEIAALARAPDANGQAHESDNVDPEYGVTQISQLRISGEANGQGSDGSDYGDGDVMNSPYDRDSSAYVYGEKEPSRMSTEQTYNTTQPLRPRSRTNGHERTENGISLYGRSSPGPVAALRYDSYHAHSQSRSSVASSQEPSPVSTRASHAGSTATSRTSGSGRSRSQSNATYNPPISASNPQIAFMKIKIFDKSAEDIVAVRVHPRVTYAQLLEKASQRLNCHVERLRYLDSETRQFIDLETDEDLRFWLDDTEKHLLNKRRPLTPTRPHRKMTETDNASAAVDAMIRGALDLDDHGISDDSIQLSNDSSASLSQEDNDLRDDDKALEWHEVIELQAFSERKAWIEEKITFLGKMPPIEVFAGLDAVRSSAVEVPGLPTRAELEEWLAEHDRIEKETEIFDSGELRKLKKFTKAAAQRNLSPEDTDLIELTLTTIYELDKLFHLLRDRSDNLDLLGIRLTWEERRMAAWSELRSVLSDIREFLASRARWTPNAYDSVPVEDELMPPEPETKRRNSVVSINSTFSESSSNLPPLVLSRTSRYKLAESLSRNAAQFASRVSSLRHSKIAGAGKALDKLIDHSRRPVPDELLDEQDKLEDHGINEMEDVGKFVMTVVMQWKKADEIYVETMKDKSAAQTLLEEIEVATSSHPTSRQDVAFLSRATALAKRLAMRDNPASSGTTFPLPTHLLFPDQSGSNEEVIRMLSSELAAAGEHVRKAEQCAKEYHTSLEAVKRVETACKAASDLSVQLESIVQRLKDGVPSDAGDGTPPDLSTEACLEGSRHASFLTVLPSILQELQDADASVDGLLPGARVAMLHLDRPGVDPQFKLEAGSNIDRLIQLRREAAQTRDEVTARTSALNDIKRTWTSMGGLAKDLTSVQDDIAAAAERQIWHQQVRQHDAPPTPESPVTHLPTPTISPASVLATLDGLATRLEEKVSTPLSAVAPAMGESLNKYLTECSTALSLLLDHTRYLTTTWVSMQNQASTMGAVRDDFHGLQVQTEELKLRFDDAIEGVLSGTLTGDELLLTEGGLSSQLKHAEESVQSFIDDLPRRVTFVAPAHSEPHSSPFPKGPRLTSVEFNLAMVQHAAAAELPVDLSHLDQTVRSDSNAYSMMLSGALKLLEYKATHFQSAKAAKIVDAALASVVSKVRRVADVVTSVQDSMDRPDEQQNSERLGKLSAMVNEVLQVEVQEIARSFSPVRGLMHRLRTLPGVSEITAADGIVASRQRALEDAEAQFATWKENAATLHEQIILALQAERARQIERLRLQEEREQAAAEERARLAREEAETLAKAELDRIEQERRETEERARREQEELEERERLEAENRAKEERSIPQGIGLILGSSELLEPVQETDEHSMTREIYGPEAKDDLFGLQVLPVTADPLSQHTSILRPQILALRKRLRSLHINDVVRPSQKSDLSLPEDEYRKRMDKELLAIVSGVDVLPPSIIDDPLTNAELHSLRTEVKASVDLMPTVHKLADLAIALRNCDDALSDLLEHIDSYPSAPLGMLSSSHTSDPTLPPESQLSARIAFTKSLVDTMTTQYSAVSGDPRAVSEHDRIVQTWMELEAMASDRVNGTKSRPNSVMSSGRSSRASAVSTAAKAAPTKKAAGYSRLSVGGSDARYLAPPPPNRRTTSGSSTGTRPRSTSRVSSISSNRSVSGPTPNVSSATLHGSTFASRQRTTSVTSGAPMKTPLKQPHLASSKASSRPRSNTNTTPRTASPAFSETSSRSISRSGLNASVSSVARSSWSRAPRQSFPALLRSPPKNKAVPTAKKPYIANPKNKLDVAVGDVINSLPVNINVEVVADTWKDQSGKYWIGDQDPKLCFCRILRSQTVMVRVGGGWSELSRFIKDHFADAFRILPESPRLGSREEKWISSATLSQAAESIALSNPKTPEVVSSHLPSFALSTPSGTSPKSIKTVSSPGSPLTPLQFLRRADRESPVPRAETPTRPPRNTAAKPARPPVWRP

>fungus-Pg-Bem1: FGI database PGTG_07653.2

MKVLRRSLHKEKDRTSTSSPTKPDFNSHHLPPLSAKHNSKQQQQQQQQQQQQQQQQPTQPFASLAAGNHNLTNPRSIGKPPTLVIKAITAYKSNRIVELSFQKGDFFHVVGERDDHEGSWFEASNPATGARGIVPKDCFELFGRKQNELPSTTPTATAFPPRSSAPGYQSSANNPPGSPLLVRTPLSAEPFRQSHPNSKTQPLYGIVQHDFVAERPDELDAKRGEPIIVIAQSNHEWFVAKPIGRLGGPGLIPVSFVEVQDVNTGKALAPNQVQDLIRSSVVPAVEEWKKATAAYKGNSIPLGKFDFAATSPPMPSATNGGSFNHPTSSRSASTSHTRQPSGAGYDRPPSGRGPQHGSWHGAGGDPSAYDHSRQSSANTPWNAPTNSYSNSRQSPTQQGRDYQDEPYPPQSGPEGDGYATVDELRERYGVVVHASVESFHFEQGHYWFHLRAHFSRISEDGRDEQTTVLVLYRLYEDFYDFQTALIETFPDQAEDEDGAPNLPRMPGPSDNVDELVCAQRVEDLSTYLHELCELPLYIRESELVYEFLGPREGDVELEGDPGNLGLDDRSPTEVEGEVVEYLQRMDSSRGEDGRLADLNESISRLSTGSNGPLEAHHQYQHHRRQSSQASQPSHHPSRMSKGSTGPAGYHQRGQRSQDLRPFSSSTTSSEGFSSHLPSTHTSTSSVPVGSRVTSLTPAGPGEHPGSANHNNPSTGFLRIKIYQRQTDDLIAIRAPTGVQFLELLQRIQERVGAEVRSIRFRDESGVAYSTGGSVPINANNGARLIGIDNDGDLDRWINSGSRLVLYVD

>fungus-Pb-Bem1A: JGI database jgi|Phybl1|1985|gw1.9.31.1

SLAAPKKIIKALYDYQAQGPDELSFSEGDFYHVTARENDSKWFEACNPATNTKGLVPVAFFQVLDKAERNLAVAQPLNDLNIKSDSGFSDSADTMLPTPSKKTSHLYGVVLYDFQAERSDELNAKEGEPIIVIAQSNPEWFVAKPIGRLGGPGLIPVSFVQVRDAVSGQIITNVSNLRQTSSNFIPNVEEWKKMTQGYEASSIPLGRIDKQPQQQLQTQQQSQSQFQSPIQDTINEGRSVKSGSSCPTHRPLSEEYLSRHNSTSRSRTATADGTQHTQAVVVSAVIDSFILEGDQYWFVVYTRLSNNTHRILYRLYEDFYDFQINLLNDYPVEAGKAKRERILPYMPGPLAVIDQEITAERQRDLNTYCKDLLGLPRYISEGDLVQVQLFGIHEGDIETDHDPRSDPAMFTQQPQQSVSQPMPGAIVGGAYYDTIKIKIVHKDDIFAIKVPANTTLDVLRDRIHDRLGFNVQLNYKDEITGESMELSEERDMEEA

>fungus-Pb-Bem1B: JGI database jgi|Phybl1|80336|estExt_fgeneshPB_pg.C_530017

MNLKEKVKPPRSLRSTRLSVVKNKISTPINFQQSAITPKIVAKALFDYKAQTQREISFHQGDYFHVDKTDNPHWFEAFNPVTHAKGIVPVNYFQILEKNDTPNHLGDPTRTETQLPPVKKMQPLYGVVLYDFQAERFDELDARAGESILVIAQSNVEWFVAKPIGRLGGPGLIPVSFVEIRDALTGHTITSSQPSNPLPRVEEWKRMTMGHDNPEKLHNPSSSALYYAPNTIDTFSRIPTHNSVNTLLGSSNAGSGAGGSASASASAGAIITTAEQIAQEIGNLSIGPRDNSRDNTRSRVVAATVDSYVPEGDQFWFIVFAQMSDRRHRVLYRLYEALNVHAFVCVLGGLRTDFYDFQVTLLQDYPIEAGKTGKPRILPFMPGPQTEVDERLTAKRQTELNIYCQELFQLPRYLVESPLVQDVLFVLHEGDVEIDYDPRTGAPRPESVELSHSILIPEAFGSSTMNTFTPFGRNSNPIKDISQTNTATATATATASTTTTAAKNAIGSSNLIKVKIAYKEEMFAIKVPIDCSFQKLHDRVQERLGGSNVRLRYKDQTQGESLPLETESDIAEAFESSLKIGKLILYATPL

>fungus-Bd-Bem1: FGI database BDEG_05366.1

MSPELPHRGHLTVAPVRGSSHPASIFNLPPKKVVRANANYTARFAGELSFAKGDFFYVINERPDGLNYEVINPIQRIRGIVPTSHFASLDKVQQEANARNPDYNGDDQNNYGYTDDGYDDSYNNHHTSDPYGQPPNNNYMGVNHGRSPLSPASPGSNQAHGISASGSGRGDKQLWSVLVQRSEQRDDGLWWFTVELKMTDNSSNILFRTYDDFWILQVSLLNHFPSESGRLDQPRTIPFLPTPTRTLTPEQAQQRRHQLDSYMQELIKLPSHIMNAPSVKRFLMVRSGDLDTPMNIRFDVSETLLDLLTEYQEEADLRIKLVLGEEIICWKEPDYISFEELMYHAEERLEFRFQALMYKDECEQLIPLRSNTDLRLLVTTLSKKLTFYGSKKSSNRLNGRTSDPASTVRTASQLTMQLESPANSNTTVPTLNIADSVNSTTGAVQVSENRVNQDTVPVGSLSASAGQGIRMARSLPRTIPSPPTGLLRDNTTVVPATIPESIMSTSIPRRQSVSDNAGASSYHLSLKINRQSSPSHHSQFRPFATTSTLESPARSALDASQPLSSLKPFISGSMNARRDGFPINTRPLSSGSPASISHLRHYQAMQRRSSSCSPNKTGSHGSNTDSEIGDVLTEDEVALMISEHLVNGDEMGGSEVGGGTASDRDRRSSSRLDGEGLDGDHRMSGMSFGSVSHGLLGGDVTRNIYRWKETRDIERQRRNSEPDLLTIPTSPSGFEIARASTLMEPGMFRRQFLQNKAQRERKRPPMFITRNFIDFLALYGVYGGDVYPSDDDEDTDSNATGFAIDSEDLVDEEMGRGGRSERLTYEEVGRNHHDGRFARNQTCIGTDAYHDECDYDHARTSETALLLAQSGLGTNGIATVPGDGGNTVGVHGTTSSSHTGTSESKAFFMLLKAFVGTGVLFLPKGFLNGGLGFSMVLLVVLGYLTLHCMILLVDTSRSLGGKSFGDIGGHIYGPYMRQLVLASIAISQMGFCCAYFIFVGQNLRDLLMVSSGCRIIWPDWVFILIQLAVYIPLSWVRRIKNFGITSLIADVFILLGLGYIFMYDLSVIGQTGIKPTAWINIESFSLFVGTAMFAFEGICLILPIAESMQHPQKFSSVLSWCILLIGTIFITIGTLGYMSFGDQIETVLFLNLPQNPLVNSIQFFYAVAIMLSFPLTIYPVIRITEQKLFGHYSRTGKSSPVVKWQKNLYRAVLACMLGVISWAGSTSLDKVVSLVGCFACIPLSFIYPALFHLHITTSWWARVTDWMLVGFGTVAMVYTTFVTLEQWAINGPDTPRDRCHDAMGTQLLELPSFGLW

>fungus-Mg-Cdc24: GenBank^TM^ No. XP364852

MNQASRRQPGLNAPRDTKLAGSVQSNDVNQQPLLPTPAPKPPDQSLPGPSRRSASENIGVGHPTCPVAFTPPNALAMAYAPLLRTNTTPIFPSSTGGVARLQGTMGSTGPAVGIPPLRASQLSGTTLHNSTTSLSSLASASTVVPAQNGGQVVATSNIINQKADASRSLYQICMSLKQRLAQVPGFEEYMSLMDQWEAEDPEGGIVESVWKLLRMGTPLIVIFNLLKPENPMSFDPTPDAKKAKMYIYKFAEQCKNELGIEDIFTISDLLKNDTTGFVKVTTVVNQVLDIAQTRGMLIQQQPYPEDDMPDSGKSKMSYRDYIVRELVDTERKYVQDLENLHDLKKALTERGIVTGDVAYQIFGNIDAILDFQRRFLIRVETTNSMPGEVQQWGSPFVAMEASFDPIYTPFIANQRKAALIAQSEFDKIKSIEHPVACDFNTLDGFLLKPMQRLVKYPLLLKDLLKKSEDDKVKDDLSLGIAAAERTLQKANEAVDRDLLDDALEDLKTRVDDWKNHQVDQFGSLLLHGVHTVLTGKSESERDYEIYLFECILLCCKEISSTKSKDKKDKTKSTGPKIRNKGAKLQLKGRIFMTNVTEVLSFAKPGSYTVQIWWKGDPGVENFIIKFQNEEMMKKWAAGLDQQRKASGSQSATSPDQPTAEFAWMRSQSAVENPYAQTQPEEEEEYEFGAIPQQSQQYQAMAGTMPRMNSTASIRGRSQTGESSQSIASIARGPPPRFPLPQPPQPLSVQTQLQGGNSPGFRPGDSYFSPIGESPVSSRTSTASNVFPGSAGYGSFKGVTGQPYPGMWDGPIVDQNRYTAPAMPRAPSRDGPSPVNAYGMSGANGRNPRGPSMPVMASHSAQGASQQSRSRSYSTPDIQGQGPPGMRRTPGGSQSNVPAVPGIPAHLHAAHERHDSNIPRSNTGSPALSLRNDLPMRSNTQSPGVQRDRLMQQGGGYSGGTLAQFPMQPVYPRGGTPNPPGSGHTPAPLNLSADMGRTVSPPLGTGTPQPPNTAGLMSPDQGGGIMPTQLKVKVNYDTSNYMTLVAAYNITYRSLVDRIDAKLARFTNSSISRGNLRLRYRDEESDFVSIVCDEDVQMAFTDGRNGDRDMYNGGVGEVELFCVGIGGE

>fungus-Fg-Cdc24: GenBank^TM^ No. XP390087

MNHAPRASQLSGSTAYTGSSASLSSLATATTITPTNGGPVHATANIINQKADASRSLYQICISLKQRLAQVPGFGPYLDELDPTDPVDPLWNLFRSGYPLLLIYNALRPNEELKVDDSSSANEAKKSKIAIFKFVQACMKELEIPSTQSFVITDLMGTDTSGFVKVTQVVNYVLDRAEERGYLMQAQPDVESNEPTGGQMTYRDHIIRELVDTERKYVQDLENLHDLKKTLEQQGEIPGDTLHQIFFNINAILDFQRRFLIRVETTNSMPALNQRWGAPFVHYEDALIDIYQPFIANQRKAAQVANQVFDKIQRSSHPVAADYNTLDGFLLKPMQRLVKYPLLLKDLNKKTEDDEVKMDLTTGCEAAERVLSKANEAVNRDLLDEAVEDLTSRVEDWKSHKVEQFGKLLLHGVYGVITGKTDQEKDYEIYLFESILLCCKEISSSKSKDKKDKLRSGGPKARNKSAKLQLKGRIFMTNVTDIVSFTKPGSHSVQIWWKGDPGVENFIIKFLNEETLKKWVNTLETQRKHNVPRQSTNSDTLSTDFAWTRDQVAGLENPYLKENDDEDDEDIGPATAPAGFPGVTHPMSLGPRTASSNNLRARAGTGESSASLAGMVRAPPPRFPLPAPPGSLSLQTQPNGAHSPSAWAGDSYFSPVTESPASTRTSTTSGMFSTPQYGFPKSATPNPQQWEDANGNRYTAPAMPRAPSRDGPSPNPARNPRGPSLPAMSSSSQAALAAQQRNRSYSTPDINGPGMPRTRQPSHGNIPAVPGIPQHLHPGHNPNLSRDQTGSPRNDQPTRAQTNSPGAQRERMHKPTGSVGGSMNHFPAQPVHPRSITPGGGNQILRVDAAAANSRTVSPALGTATMPPPSANPLSPEIPLPTQLKVRVNCESGNYVTLVVAFNITYQSLVDRIDAKLARFTTSSISKSMLKLRYRDEDGDYVAIEGDDDIQIAFMEWREGVRNMYSGGVGEIELFCVGDTA

>fungus-An-Cdc24: GenBank^TM^ No. XP663196

MADTAGLNPGGPVAEDNIINRRGNEGIYQSCVNLKKRLAEVPGFEPYLREMEEEDLAQGNSDPVASLWNYLRHGYPLLAIYNASDPGAPLEIDTSKVPEARRPKAATFKFLQAAIQEMAFPQQECFLITDLYGENTIGFTKVIKMVNRVLDILEIQGQLKKPSDTAMAAPAAGRKLTKREHILKELLETERDYVHHLQNLQALKKELEDTGALTGDASHQIFLNLNNLLDFSQRFLIRLEQHYARPEEQQNWGELFIQHEEAFRQYEPFIANQMRCDKTCQKEWDKIQAAPRSPDLQQMVAQPATLNGFFVKPFQRLTKYPLMLSELRKQIEDPDLQADISRAIDSIQSVLDAANDAIDKEQLAAAFVELDERVDDWKALKIETFGELLRFGTFTVIKNDNNKDSEREYHIYLFERILLCCKDINPNKQKSRLVGGSKDKPNTSKGKPRLVLKGRIYMANVTDIVWLQKPGSYRIQIFWKGDPGVVDNFIIRYQNEDTMRKWYKDINTQREIQAEQRSARNTGTSDSEFTYMKSLSNIPNPYQQEYDVEEQSTKEAAFFSEFPMSRNASSTSLRTRSATGGSGSSGPPLSTSRPRYPAMPDSTLSVHTQFPGGSMSPGERNGNSYFSPTESARSSSQSAGYPYNRQVTPVTPWGDDNNRYTAPALSRATSRDGSNSGYFNGAPPNGRSAQRPSLPPMSGSNQSSNSASQRMRSASSPDIHHHNPESRRYMGVHTMQTVDNVPVPPIPAHMANMKAPVNRSQNNSPTNQSLPIRTNTSHAFHEPQYSDGRAAAPLSDQPTSPLSHEPEEEPFMPTQLKAKVNFDENYVTLVISSNIGFRTLTDRVDAKLARFTNRSIGSKTVRLRYQDEDGDFVTIDSDEAVQLAFVEWKEQHREELARGQVGEIQLFCQPIEN

>fungus-Sc-Cdc24: GenBank^TM^ No. NP_009359

MAIQTRFASGTSLSDLKPKPSATSISIPMQNVMNKPVTEQDSLFHICANIRKRLEVLPQLKPFLQLAYQSSEVLSERQSLLLSQKQHQELLKSNGANRDSSDLAPTLRSSSISTATSLMSMEGISYTNSNPSATPNMEDTLLTFSMGILPITMDCDPVTQLSQLFQQGAPLCILFNSVKPQFKLPVIASDDLKVCKKSIYDFILGCKKHFAFNDEELFTISDVFANSTSQLVKVLEVVETLMNSSPTIFPSKSKTQQIMNAENQHRHQPQQSSKKHNEYVKIIKEFVATERKYVHDLEILDKYRQQLLDSNLITSEELYMLFPNLGDAIDFQRRFLISLEINALVEPSKQRIGALFMHSKHFFKLYEPWSIGQNAAIEFLSSTLHKMRVDESQRFIINNKLELQSFLYKPVQRLCRYPLLVKELLAESSDDNNTKELEAALDISKNIARSINENQRRTENHQVVKKLYGRVVNWKGYRISKFGELLYFDKVFISTTNSSSEPEREFEVYLFEKIIILFSEVVTKKSASSLILKKKSSTSASISASNITDNNGSPHHSYHKRHSNSSSSNNIHLSSSSAAAIIHSSTNSSDNNSNNSSSSSLFKLSANEPKLDLRGRIMIMNLNQIIPQNNRSLNITWESIKEQGNFLLKFKNEETRDNWSSCLQQLIHDLKNEQFKARHHSSTSTTSSTAKSSSMMSPTTTMNTPNHHNSRQTHDSMASFSSSHMKRVSDVLPKRRTTSSSFESEIKSISENFKNSIPESSILFRISYNNNSNNTSSSEIFTLLVEKVWNFDDLIMAINSKISNTHNNNISPITKIKYQDEDGDFVVLGSDEDWNVAKEMLAENNEKFLNIRLY

>fungus-Yli-Cdc24: GenBank^TM^ No. XP_501845

MNGSSRRLSPPNDNLAHQQMLHQEINNLLPSSPYTKTPQQRKSSSQSTPSLSTLSPSTQNSTYPSTPRLRSMTPTMSMSSVQSTAPTSVAPMPVANLVMNRQADSEKSLYNMCKTMRDRFHDIEQVRPFLEDSEMSSIVPITHLPPSIPLDPVTQLWSLFRLGAPLCILFNMLQPRTPLTVDPHAAANRASIDVRTCKRSVYDFVQGCKAELGYSDDELFTISNLFSENTGDLIKVMRTVSMVVETLEARHLLPPKTAPITAPATRDARYNVVQELLQTERKYVQDLEILHDYQNRLLEAEILNPDTIHLMFLNVSALVDFQRRVLVGIEHVCSLPVEEQRFGALFLNMEAGFHIYEPYALNQKASSDLAVQEAPKLTALAHIIEPTYELQAFLIKPIQRICKYPLLLRELVKVSSSDHPYASELLDGLEAIKRVTNSVNETQRRVENVAIIQDLCERIDSWKSYNLEDFGDLLYMDHLTIAKESGEYEYLVYLFEAIILCCKETRDKKGKTMSLPSKKPQKTVLSLKGRIYIANVTNMISSTENGYSLTLTYKGDGYGSMTMRFRHEEQLRQWDATIKQLHDWYNQDSYESHFSPLVESPISSRNSQQSSHLSEDLDRLTLGNRSLSGPVPKLGPRQHSTTSINGMHGANGMNHSSVSIASSNGGGMSRARSASTPGFDDGSMRPSNIKVRVQYFQDVFTLIVPAKVTYDQLIERVERKVRLCGKVVPAPLRIKYQDEDGDMVTIHSDEDIAMALEQKNLEQLNIWVA

>fungus-Sp-Cdc24: GenBank^TM^ No. CAB11037

MAYFQDRKTSSRSLPSYINHSTQNLVGPRKDETNLSEYMKLRLLQSPSQVIYNLENTVSLYRRCLNLRKRLMDISELAAFFDSIHREALNSSFKILEFKDIEFDDPVTEIWLFCRLGYPLCALFNCLPVKQKLEVNSSVSLENTNVCKASLYRFMLMCKNELGLTDAALFSISEIYKPSTAPLVRALQTIELLLKKYEVSNTTKSSSTPSPSTDDNVPTGTLNSLIASGRRVTAELYETELKYIQDLEYLSNYMVILQQKQILSQDTILSIFTNLNEILDFQRRFLVGLEMNLSLPVEEQRLGALFIALEEGFSVYQVFCTNFPNAQQLIIDNQNQLLKVANLLEPSYELPALLIKPIQRICKYPLLLNQLLKGTPSGYQYEEELKQGMACVVRVANQVNETRRIHENRNAIIELEQRVIDWKGYSLQYFGQLLVWDVVNVCKADIEREYHVYLFEKILLCCKEMSTLKRQARSISMNKKTKRLDSLQLKGRILTSNITTVVPNHHMGSYAIQIFWRGDPQHESFILKLRNEESHKLWMSVLNRLLWKNEHGSPKDIRSAASTPANPVYNRSSSQTSKGYNSSDYDLLRTHSLDENVNSPTSISSPSSKSSPFTKTTSKDTKSATTTDERPSDFIRLNSEESVGTSSLRTSQTTSTIVSNDSSSTASIPSQISRISQVNSLLNDYNYNRQSHITRVYSGTDDGSSVSIFEDTSSSTKQKIFDQPTTNDCDVMRPRQYSYSAGMKSDGSLLPSTKHTSLSSSSTSTSLSVRNTTNVKIRLRLHEVSLVLVVAHDITFDELLAKVEHKIKLCGILKQAVPFRVRLKYVDEDGDFITITSDEDVLMAFETCTFELMDPVHNKGMDTVSLHVVVYF

>fungus-Ca-Cdc24: GenBank^TM^ No. AAO25556

MEHPPAALRTFSTQSTSSLNSVSTVSSSRIVSSGPVNINNFNKPSTPKDHLFYRCESLKRKLQKIPGMEPFLNQAFNQAEQLSEQQALALAQERSNGNGHSNGKRHQSLDGAMNRLSVGSDSSSIQGSLTRMATNASTSSLISGMPNNNTLFTFTAGVLPANISVDPATHLWKLFQQGAPFCVLINHILPDSQIPVVSSDDLRICKKSVYDFLIAVKTQLNFDDENMFTISNVFSDNAQDLIKIIDVINKLLAEYSDASDSGGGDEDVNMDVQITDERSKVFREIIETERKYVQDLELMCKYRQDLIEAENLSSEQIHLLFPNLNEIIDFQRRFLNGLECNINVPIRYQRIGSVFIHASLGPFNAYEPWTIGQLTAIDLINKEAANLKKSSSLLDPGFELQSYILKPIQRLCKYPLLLKELIKTSPEYSKQDPHGSSSSTSFNELLVAKTAMKELANQVNEAQRRAENIEHLEKLKERVGNWRGFNLDAQGELLFHGQVGVKDAENEKEYVAYLFEKIVFFFTEIDDNKKSDKQEKKSKFSTRKRSTSSNLSSSTTNLLESINNSRKDNTLPLELKGRVYISEIYNISAPNTPGSTLIISWSGRKESGSFTLRYRSEEARNQWEKCLRDLKTNEMNKQIHKKLRDSDSSFNTDDSAIYDYTGISTSPVNQSTQQQYYDHRGSHSSRHHSSSSTLSMMKNNRVKSGDLSRISSTSTTLDSFSNNLNGSPNTTNPSLMSSDATKTIPTFDVAIKLLYKSTELSEPLIVNAQIEYNDLLQKIISQIITSNLVADDVNISRLRYKDDEGDFVNLNSDDDWGLVLDMLTSEDFYQTSSNEKRSVTVWVS

>fungus-Cc-Cdc24: GenBank^TM^ No.EAU91134

MASQAAARKKSIISSQNLQIETPVAGNTLLNKSASQATSLYQQCASLRSRLMRIRGFSYYFNLAAESTDDRQSTDPVTQLWDIFSLGISLCYIFDQLDQNFNKINNSQFNPEKYEANPDKERKHAIALFAMQVRNNQVMQAIPGLEPFAITDLWDRKSTDGLVKVINSVTAIVEYLPEDVFEQAPTSPPTLSAHESSDSLHHDSLPAGPAPPTNAREARRIHITREILETERKYVEGLEIMQKYATQLAQQNIIDQDTIHLLFPNLNNLLNFQRKFLIRFESTLEQPWSEQRWGQHFVDCEDEFAVYEPYCSNFTNATELLVANEQNLAALNGFISVSELKAFHITPIQRVCKYPLFFGDLIKVATGDDTYPHLEGLKQGAEAAHRITAKINEAHRRADNEQIVKSLAKRITDWKGHHLENFGELLLEDVFVVTKSDIDREYHVFLFEKIILCCKEALQQPPNGRGKNAKNNSILKKQPAPLPLATGGQSIAQRDTPLLLKGRIFLGNVTQALSVPARASTASGIPAHFPLQVWWKGDDDLEFFTLRCKREDQMRQWESTINRLIKEAAQRRASERPHGPISRVAHHSPIVRVPPTDPHSHFSSQGSVVSSSAYSSQASSAVSARSSSRSTAPYHYESSHTSYSSSGPQGYPPHEGFDRDDEEDDLEDYPPASTSYSGRGTPMGNRRPTALSMPGERSESLHGYERPRAQTESNDGPTIAQWRNNVAGPRA

>fungus-Lb-Cdc24: JGI database jgi|Lacbi1|313963|eu2.Lbscf0005g05520

MATVAGRKKSVISTAGLTIDTPVATNGLLNKAASQSTSLYQQCSALRSRLMLIRGFPHYFNLATPPDSRQSTDPVTQLWDLFALGVPLCYIFDLLPAEAGFNKINNSSFTADYDANPDRAQKRAIALFAMQIRTDEVTQKIPGCELFTVTDLWDRKLTDGLVKVINTVTAIVDHLQPDVFEESPPSPPTLSSHDSSDSLLDALPQPNGKEAARNNIVREMVETERKFVQDLEIMQVRVFVPLMSLALPIQPAGYVQKYSNALSQANLMDQDTIHLLFPNLNKLLNFQRKFLIRFESTAELPWQDQRWGQHFLESEDEFVVYEPYCANYTNATELMLANEESLVPLNHLINVKGELPAFLIKPIQRVCKYPLLLDASSPTTYQHFDELKRGSDAAKRITDKINEAQRRAENEQTVKSLHYRIDDWKGHHLENFGELLLDDIFVVTKSDIDREYHVFLFEKIILCCKEAPLLPQNGRNKVGKNNSILKKQGTPPPLTLPGGVGQPQKKTTPLLLKGRIFLGNVTQAVPVSARISTPSGVPPHFPLAVWWKGDDDLEFFTLRCRREEQMRQWEAQINRLIKEAAQRKASDRPTMSGIARIAMTNSINPSSASRFGNSSTYSNGAPVSIHHQPSISSSIRSNRPVNSYNHDDASSNAGSMYSAGPQGYPPHDGFDFEPEDEEDYEDYPPVSAANYPASGRGTPMGGPPGARRNPNALSMPLEREPVAGYDRPRAQTESTDGLTMAQWRNGLPQPPSNQPSPAGALRPITPRMNSNVSANSYTSDASFGNSSVPLPLKSSSRPVLRSQASSNRLKNGYDASYNSGIPTPAVNSSDYRTVRAPTPTGGLTAPLMNRSRSASQPTAYVPKTAVPPPMPSYNSHWSTHDRSNHLNNNKRGSGSSQSTGDSSEYSPNSSSPVTPFGSSESSLGGVAIRNSRTQPYESAPALNGHHNVKVKVHFHEDIFVIQVSRATEYEDLVEKVGRKIRLCGPRRDDGPLRVKYKDEDGDMVSLGSTEDVQMAFEQGGQVTLFVT

>fungus-Pp-Cdc24: JGI database jgi|Pospl1|128494|estExt_fgenesh3_pg.C_2160016

MASAAGRKKSVISATTPTIESPIANNTLLNKAASQSTSLYQQCSALRTRLLQVQDFPEWFTVSSPPDSSRRSTDPVTQLWDCFALGVPLCYLFNLLPAPFSPINIDTDPKSFDATNEKTKKRSIALFSMQIKQLEGCEQFTVTELWDRNSTDGFVKVVNNVINLVRCLPDEVFVEPQLSSPHLASAQQSTDSLDADGAAPPPERDGGARYNIVRELVETERKYVQDLEVMQKYSIAASQMNVMDQETLQRLFPGLNKLLNFQRKFLIKLESIAELSPNDQSWGVPFMENEEEFAVYEPYCALYTSASDIMLQEMQNLTALDNVLSKSELPAFLIKPVQRICKYPLLLESLLKAVKTTDYPFIEELKLGVAAAKRVTDKINEAQRRAENLATVEALKGRVEDWKGHHISQFGSLLLDDIFTVTKSEVDREYHVFLFEKIILCCKEYLVPSANGRKVGKSNSILKKPPVPPPLMLPGAGPSKKRNTPLLLKGRIFLANVHLAEPKISAGQYSLAVYWRGDDDTEFFTLRCRNEEQLKMWETQLNRLIQENINRRMSDRNPQRLTQMTPSSAAAMQQRPPPVAYNQEKTYSTFSQSTVYSAYGATSPYGAPGRMGRHPYAAAGDEPPASANGSYSNGFGHGPQGYPPHDGFDVDLDEDYEEYPLPNYPPSGRATPAGSRRSDMIGFSQSARTSSHSSIPSAMSSHTSLYPGRPSISRSAMSYNVETDSASSSTTTRSALRSQFSSTRLKGAYDQAESRPNASSRQSAPAAPSRSRSASQPTAYIPPTVPPPLPTSIPSSVQWSTRAQSTVGNGHNKRGSGSSESTGESSDYSPHSSGSPITPFGSSDTSLAGMSASRHSRSQQHVDQVVTSAANELSRPVKVKVHFHEDIFVIQVPRSTEFDQLVEKVGKKIRLCGPNRRDDGPLRVKYKDEDGDLVSLGSTEDVQMAFESFRPGNLVTLYVQ

>fungus-Pg-Cdc24: FGI database PGTG_04844.2

MTTNPPPTPSTSSFNYQNPFSSSTRSSTTTTHTMTTTTTSASSHTSLNQHQSANLSIDMPIATSSVVNKTANRSASLYQNCLETRALLRRVPGFEAGPWITETKNPNSLLSVTQTLEIFRLGSPLCFLYNAILNPPLSPPELANLPPLPPGKTWQNLKPLKVLWCPREDLKGCQKSAAHFIMALKTELGWGTEIDEIQENGLMVNMLYDVNTNATVKVISNVLRLLHLLKALGVLLPPSEDELVPHSNELNVAMDDRARVVKEILESERKYVQDLEVLQNYQRALLQREILPSDQIRALFINLDSLVDFQRRFLIGVEANARLPPDEQRFGHLFHSFEDSFSCYEPFCANFASASQLAQDENAALTQLVKHTSPNGAYYDELKEGLASITRVTDAVNETTRQRENELAVNDLKERVEDWKGHELVTFGQLVLEDTFTVVKNESEREYHVYLFERIILCCKEVGVKLDKKASKQSMSLKKNSQINNSNVNGGKRKNTQQLKGRIFIANVMSATPRPVPPEEQLKKWQAAITKAVDDAVERRRRQVHHLSSGSRSKMNSPLSQFPNTPQSEMGQPPNSASYQHHPVSHPPRIPEHPYAHSGHGNGGGSISSRTFEDEEESYQTDAESSVNGRNTPSGSRRTTGHRSMPAERSEASFGPGRARAQTDDAASANLAQWRNQMPNSAIPPVPRSGQLTPASSDAYLPNSLRSSTSSKPLRHQPSHEWGAGTINGSNFYRVDDRSHLPTSGPSGHPLHARHNEPQLLPSNSTGVPMFRSRSASSPNIHQSPDFSQQWNSGLLNPVPSVPSLPSGSHSSSSTSRPALPLHNQVQIGGHIKQRSSDSSAAALTDRSSTESQRLSGGVGGGNGVGGRSNLPLSNPMAQTAHGGPPAGGGGLNPRYSSGQAGLTGHSAASSLITSRSSGSLVVVQGGSEGVGGSRYGAADQLVGGSHPSKPLGGGGGSSSTSSTMKSSLTATHNPNNNNNNNNGPTSIRFKLRAGEDTYVIVTLSTITYHELIEKIYKKLRNCGVYKDSSARLKIRYEDEEGDLILICNDEDVSMAIEWMRSVGISHLLLFVD

>fungus-Pb-Cdc24: JGI database jgi|Phybl1|22653|e_gw1.19.182.1

MPSCLIESPQKPIQGQSLYHTCLSVLDRLALVNGFQQFLDSPVQKTQLLNTDPVTKLWNLCQLGTPLYTLFNALDPKEPLTLEYNHNLNQANNAKAIVYHFLVSCRKELDFAEEDLFGITDIFLDDTNGFVKVVNTITKILQILQDRGVISAKSPNRNSDPNAPKDTRDKIVLELLETERKYVQDMELLQASTKEHKIYIPYNILTKFFYVFIQSYMRELQIQKILSPDTVHYLFANLNALVDFQRRFLIQLEDIAEKPAQDQQLGLLFVQTEDAFIVYEPYCSNYYAAQDLVKEKAAELQKLDHVLSPTHQLLSMLIKPIQRICKYPLLMSSLLKSTNPSWQSYPQLIQGSEAIRRVAEKVNETQRKQENLQLVQDLKKRMDDWKGPPIDSHGALLLQEKLMVTVNDLDREHLILLFEKAILICKELKEGSKNRLTKTNTIMKKKRRASIQAKGTISVNRITHILNKSTASGGKYYSSSNSNSVQQFTLKFRNEEQLKQWEAVIDKTRMNIQSTSSSTPLLPTPSQSTSDKTSFIDSLDDDDDDESTKKFIRPIASSSSRLSELANIPLSLLLSSTSTSTSTSTSSASSGSSYFPPSLLSPVTLTGSQDTLKVKLTYNEGVYCIVTPHQIKFSDLMERVEKKIKIVANLKPNAVLRLKYRDEDGDHITISSDDDVQMAFESRGQNTVNLFVTL

>fungus-Bd-Cdc24-like protein: FGI database BDEG_01878.1

MANQVAVADDTIPYGAVIEPPSIVRNLSTPTLAHSSSHRSQRSIANSESMHEVSIVYPALLSQVAMAFKSRVSLDTHLKDLLEYTDSFFGRDGVDVLCYIIKTSDRNLSLLLGRALDAQGFFHDVTYTHRLRDSPHELYKFESWESEDTKTRDLDLLPHGVFTLLTDCYVPTCTRDQLCYSITCPRRIEQRTRLHRQANMLLSRHYSKGSINEKAIGTLWSTSVSKEIVASVSDKERLRQEVIFEIINGEREFVEDLDIMTKVFIQPLRERNIIEPERKEKFIQDVFLNITELHTINSKLLRKLLIRQKENPVVDKIGDIFINIANEFYPYVEYGAKQVYAKNLLDEEKASNLDFVKFLKECERLPALRKLPLESFLARPTTRMGRYPLLLKPVMEKSAETHPDRTLIPQALLSIREVLASINVEAGKADNIVKLSRLDRQLQFDEGEKEDLRLNDEGRTIVRDGKLMLKRSGNDMELSVFLFDHMFLITRKKENGHYKVARKPIPLELLTFRLDKPPRAGGDLVSTATSKAATLYGVAAQKPNSISHKHTHSGSNPMALTDSASRTFPIFISHLGQSGGQYTLLASSQADRQSWCDAIEKQKTILSERKKRFEIVSLVSSGFPVSNRINCSISYLDRLILGTDFGLFVGTEYPTSTDLLTELNENRFVKVIDLERIVQVDVLPNNDNLLVLSDKTLLSFPLQVLNISEVDTTGAGYKGKKIGSHVSFFKQGICAERTLVCAVKSTALTATIKVLEPVGLGVNNLRGKIGKLFRATNDALRVYKEFYIPTESKSIHYLKSKLCVGCAKGFEIVDLESLNTQGLLDPTDESLDFVLKKETVRPISIFRVRDGDFFLCYNEFGFYIDRLGRRAKTDWLVQWAGAPTAFSFAPPYIIAFEPSFIEIRHVDTGELQQIIQTNNLRALNADSDALHCAMDLMDDMQQVFRLQPIDQFRLR

>fungus-An-CBSnPB1: GenBank^TM^ No. XP663320

MSSTPTFRGTTSHRTVGRGRLPDFEGGSSASHIPRPRPESSSTITSHNPHTPSSDIGSSTMSAASSRQRQNQSKRDEAIRRKLEADLNKKRSNPARANRTRKAPPGTVLALKPSSALQIKPSTTIAEAAQLMAAKREDCVLVTDDDDRIAGIFTAKDLAFRVVGAGLKARDITVSEIMTKNPLCARTDTSATDALDLMVRKGFRHLPVMDENQDISGVLDITKCFYDAMEKLERAYSSSRKLYDALEGVQSELGSSQPQQIIQYVEALRSKMSGPTLESVLDGMPPTTVSVRTTVKEAAALMKEHHTTALLVQDQGSITGIFTSKDIVLRVIAPGLDPATCSVVRVMTPHPDFAPSDMSIQAALRKMHDGHYLNLPVMNEGGEIVGMVDVLKLTYATLEQINSMSTQDDEGPAWNKFWLSMDHESDSMVSGSQSHQPHRSIVNPESPKASFDARDSVLPNESASHHGGDEHSEFHHGELSPFPFKFKAPSGRVHRVNILPAAGIAELVAQVTAKLGPEVEAVGGAASCADGVLSNTGYALSYVDNEGDTVSITTDQDLVDAVYIARHARRDKVDLFVHDPAQPPVIPAPVEPAPVKPVEVKTPASDDQLSEESPIPKPRATQAYPAHPPEEQLIAGVPNDLLLPGAIVTLAAVIAGVFILSRATSR

>fungus-Mg-CBSnPB1: GenBank^TM^ No. XP361856

MSASRQKQTKRDEAIRRKMENDLAKKKHVTGRARHSRKAPPGTVLALKPSQALQIKPGTTVAEAAQLMAAKREDCVLVTDDDDRIAGIFTAKDLAFRVVGAGLKATNVTIAEIMTKNPLCARTDTSATDALDLMVRKGFRHLPVMDENQDISGILDITKCFYDAMEKLERAYSSSRKLYDALEGVQSELGASQPQQIIQYVEALRTKMSGPTLESVLNGMPPTTVGVKTSVKEAAQMMKENHTTAVLVQDQGQITGIFTSKDVVLRVIAPGLDPGNCSVVRVMTPHPDFAPMDMSIQAALRKMHDGHYLNLPVMNDSGEIVGMVDVLKLTYATLEQINTMSSGDNEGPAWNKFWLSLDNETESMVSGDGSHHHSHSHIHHRSVMSPDLTRERLDSVAPGDSASHAGVESPGHSIVQHSPELPVSEIPFPFKFKAPSGRVHRLQVIASHGVAAFITNVTSKLGGEIEAVGGAPEVTEDGQVRGGFALSYLDNEGDSVSITADQDLLEAILLARQSGRDKVDLFVHDPEKPPVAVAAPAAVPPAPSEPVSVPIPTPPASSVARERRRRKSPASDESDEEEESEDDGSSTLRRPRRHQRAAAAQQEPLIAGVPNELLLPGAIVTLAVVIVGVFTISRISSNNR

>fungus-Fg-CBSnPB1: GenBank^TM^ No. XP386485

MSGHSHNTHRSTPNKGRGAVPFANSPSGGGGGGSSNIPRPVLEPTPPAETGSSFSASRQKQSKRDEAIRKKLENDLSKKKHLTSRARHSRKAPPGTVLALKPSQALQIKPNTTVSEAAQLMAAKREDCVLVTDDDDRIAGIFTAKDLAFRVVGAGAKASAVTIAEIMTKNPLCARTDTSATDALDLMVRKGFRHLPVMDENQDISGVLDITKCFYDAMEKLERAYSSSRKLYDALEGVQSELGSTQPQQIIQYVEALRSKMSGPTLETVLNGVPPTTVSVRTSVREAAQLMKENRTTAVLVQDQGAITGIFTSKDVVLRVIAPGLDPANCSVVRVMTPHPDFAPMDMTLQAALRKMHDGHYLNLPVMNDGGEIVGMVDVLKLTYATLDQINAMSNNNDEGPAWNKFWLSLDAETESMMSGEGSQAQHTNLGSRLTSPDMVRDRLNDTVAPGDSASHVGMESPPRSILPDVLEHQLPEELPFPFKFKAPSGRVHRMKIVATDGIEAFVEAIASKLGAEADNIGGVPDVEDGKIVGSGFALSFLDDEGDSISITADHDLLEAVILARQAHHDKVDLFVHDPEKPPVSAADPRHPATPSVSTGAGLRERRKWWPEEEEEEDDDDSEDEHPARRRKSRAAHTHAHAHEEQIIAGVPNELILPGAIVTLAVAIVGVFTIARLTSR

>fungus-Sp-CBSnPB1: GenBank^TM^ CAB11262

MTVGTLSVVSSTASDTASHVSDTRKRQYQRDEALRKKIISELGKKSGNFESPVRKIRRNGEPGTVDSAALDPALTVHMQSLVTETAQLMAAKRQNCVLVVDDDEQLAGIVTATDIATRCVGAGLNARQTLIADIMSTSPLCITSDTRFDDALLLMIEHKFRHLPVVSDGGPDGSAGDEGDVIGIINMRACLREPLNRIARQQEAAQKLVEALEGAQEEIENKSVSGNTNSSSVSGNHAAEFLEYVESLKKKASGLEIMSLIDSSEEPFLVGTRTTVAEATESMARSGVSAVLVMDNGAVSGVFTAHDVVLRVLAAGLDPYRSSVIRVMTPHPDCALASLRVSTALERMIEGKFSNLPVVDESDAIIGMLSLFHLATAIEQTPEEEEEVFDQAENDAGIEPSNGFEDQQQQLLGNSNEVVENYDVNPPLPLNPLPSNTQQSESTYEYSARQLPKPPVQAWQNENLSSNNKPQEYVGVENDYNFSNNPPTAMSEQSFHPSVSQKPMDTPENGSNSFAASPYLQPYNSASQLAPSYVGSLPQYHGNPSFVEQALQDLVQPTDSASQIFPLNPQSPSQFTIKYRSIAGRVHRLRLDGINSVSDLRTAVEEREKEQLVTLTYIDDEGDVVELVSDSDLREAILLARRRGLPRLEVRGVAAFTNHLESSHPPISTVDSSIGSASVVEKGVANSIVDIHQPTAKADKGNSKKPIYIGIVSSSIVILAVSMWYLRRKR

>fungus-Yl-CBSnPB1: GenBank^TM^ CAG81162

MSRNTPSRFSNYSGTSGTQSPIDSLTESRKRQSKKDEAIRRKLETDLSKRSHVRPKPTKKIVSGTVMALKPSPAVTIKPKTSVSEAAQLMSAKRENCVLVIEDDNISGIFTAKDLAFKVVGSGLDASVTTVDQIMTRNPLYATTNTSATEGLNLMVNKGFRHLPVMDENNEVSGILDITKCYHEAMEKLERAYQSSRKLYDALEGVQTELGSSQPAQIINYVEALKQRMEGPDLESVLDGTPPTYVDVRTTVFEAASLMKQNHTTAVLVTDHDQVKGIFTSKDVVLRVIAAGLDPKNCSVIRVMTPHPDVAPQNMSIQVALRTMHEGRYLNLPVMGPNAELVGVVDVLKLTYATLEQINTMSTGDSEGPAWNKFWMSLDDGSEAGSETNSHNRIMSPVSVSSPEVSHSELAQFGVGEVGPSDSISTDGYDRPGSNAAGMIATMPFQFKFKSPSGRNHRLTVVPNAGVTALRTSISDKLSVGDVELVTQNYAISYIDDEGDIVAITSDQDLLDAVSITRRAGLEKADLFIHHPDNEVKPVVTQKLEDSVEIPVKAAAAPAPEIIPGLSNDLLLPGAVAVVAAACAVAFSLGRAR

>fungus-Ca-CBSnPB1: GenBank^TM^ EAK97372

MAPPTSLDSRQRQSKRDDAIRKRIENDLRKKKASTSLSTRRSRGAPGTVLWLKPGEPIICKPTATVYEVAQLMTARRENCVLVVNEIGELLGIFTAKDVAFRIVGSGLNATQVTIDTIMTKNPICANAADPAGDALNLMVERGFRHLPVLDEKSQIVGVLDITKSYAQQMEKLERMHSSSKKLHEALDSVHNEIGVGEQPHHVFQYFETLKNKMNGPTLEDALDANTVPTYVNVKASVHEATMLMKENRTTAVLVKDTNEQVAGIFTSKDVVLRVIAAGLDPKKCSVVRVMTPQPDVAPIGLPVQDALRKMFDGHYLNLPVVANEGDIIGVVEVLKLTHITLNQIKQLETSETSNGSTAVDSTNEGPAWNKFWTSLDNTDGDTESAHSDSLMETSRGSATAPDITPSEFHSFNVDIKPSDSVSHVNTSPLKASSFKMTSSMTVDEIPFIFKFKSPGIEGRVHRITLKASDGIIKLRELMNEKLHDKDFAFLNVPKPDGNSSTQETYAISYVDDEGDVVSITSDSDLAECIRINLNLQNEKADLYLHNPHEPAPIENIKSIHKSAKNRHGNADNGLIFGIPNEILIPSALAVLGASIIVGFTLSRK

>fungus-Cc-CBSnPB1: GenBank^TM^ EAU93401

MSTVSGYSMHSETRKKTNRRDEAIRKKIESELARKRTISTHQPQPPSRSRRGKNAPTKGTVAALKPSPALTVPENITVAEASQLCAAKRTDCVLVVDEEEGLSGIFTAKDLAYRVTAEGLDPHSTPVAQIMTRNPMVTRDTTSATEALQLMVSRHFRHLPVCNEDGNVVGLLDITKVFHEALGKVERSSAASEQLFNAMAGVQSELGGVGSNPQAAAMLAWAEKLREKTALPDLTTVMDSRTHPATVGPKTTVRDVAKLMKERRTTAVCVMEPPGPGTPHPRIAGIFTSKDVVLRVIAAGLDAGRCSVVRVMTPHPDTAPPTMTVHDALKKMHNGHYLNLPVVEEDGRLVAIVDVLKLTYATLEQMNAMSGEAAASDNEGGPMWGRFFDSLGQDDTESVFSGSHATGSVLPGSMHLAQSPHSEVHPNDSASVVDDDNGSMLSYSRKKGVSVPLGSAPVQPDDGTYVFKFRTPSGRTHRFQSRHDDIQLLREIVAGKLAIDPFFTEFTGNPGDEVPDPNDFHLSYTDDDGDSVLMTTDSDVADAVTIARNHNQQRVVLFIQGGKGWAEAGDKSEAKAQEVSAAALKEVKEVEKAEEEAEDTPLVQTPPKATTPPTRGVVHNPQEEVFGIPKDLLLPASIGALAVVIIDGEWVKPLDR

>fungus-Lb-CBSnPB1: JGI database jgi|Lacbi1|228583|e_gwh1.1.177.1

MSSLSFQSDPRKKQSKRDEAIRKKIESELSRKRTISTTQGQRSKRGVGKATPAKGTVAALKPSPALTVPENITVAEASQLCAAKRTDCVLVVDDEEGLSGIFTAKDLAYRVTAEGLDPHTTPVSQIMTRNPMVTRDSTSATEALQLMVSRHFRHLPVCNEDGNVVGLLDITKVFHEALGKVERSSAASEQLFSAMAGVQSELGGAVGSNPQAAAMLAWAEKLREKTALPDLTTVMDSRTQPATVGPKTTVREVAKLMKERRTTAVCVMETAGPASPGLPAGSRIAGIFTSKDVVLRVIAAGLDAGRCSVVRVMTPHPDTAPPTMSVHDALKKMHNGHYLNLPVIEEDGRLVAIVDVLKLTYATLEQMNAMAGGDTTSTEAEGGPMWGRFFDSIGHEDTESLFSGSHVTRDHRSFSSINDLHLQQSPHSEVHPNDSASVIDDDPVSVLDGYPRHKGLNIPSGAAPIPVDDGTYVFKFRTPSGRTHRFQARHDDVEHLREIVSGKLATDPFFTEFQPKNEDSPKPDPIDFHLSYTDADGDTVLITSDDDITDAVKIARTAGTDRVVLFVQGGKGWAEAGADKSEAKAAAVTAAAQKEVKEVEKAEEIIVVTPPPVEAPVAQTPPVQTHRPRAPPPAEEVFGIPKDLLLPASIGALAVVIIGIFTISRLSSPSHY

>fungus-Pp-CBSnPB1: JGI database jgi|Pospl1|122378|estExt_Genewise1.C_2090018

MSTVSASVAETRKKQTKRDEAIRKKIEGELSRKRTISTTQHTSSRRGHKPNVQKGTVAALKPSPALTVPENITVSEASQLCAAKRTDCVLVVDDDEGLSGIFTAKDLAYRVTAEGLDPHVTQVSTIMTRNPMVTRDTTSATEALQLMVQRHFRHLPVCNEEGNVVGLLDITKVFHEALDKVERSSSASEKLYSALAGVQSELGTGLGANPQAAAMLSYVESLREKTALPDLTSVMDSRTQPATVGPKTTVKEVAKLMKERRTTAVCVMEAPTHAPHAVAAEPPKIAGIFTSKDIVLRVIAAGLDATRCSVVRVMTPHPDTAPPDMTCHDALKKMHIGHYLNLPVVEADGRLVAIVDVLKLTYATLEQMNSMTTTENAGENEEGGPMWGRFFDSLGGDDTESALSGSHFGTDVRSRHLSRSMSHTVHSPEVHPNDSASMVDEQEEHSVLEGYSSKQGEPLPSIVGPPVPLPAVDDGTYVFKFRTPSGRTHRFQARHDNVENLRDIVAGKLATDPFFTNYKTADPSEPPPDPTDFLLSYTDADGDIVLITSDTDVSDAVKIARAAGADRVVLYIQGGKGWADVMAEKSAAQAAEVAAAAQAETKEVEKAESVLPEPVSNPPPPPVHVVEDETVMGIPKDLLLPASIGALAAVILGVFTISRLSR

>fungus-Pg-CBSnPB1: FGI database PGTG_04250.2

MSRIPLPIQVNSTTPTQHQHQHQHQQHSPNSLMTSSQRSANRSGLPRPNSIYSDDPALTSSTSPSSHRNTSFQSSLSETRQRQGRKDEAIRKKIENELQRKRPGQKRTNNQPAGSSGGPRPKRQAGTVSALRPLPALTVPDNITVADASQLCAAKRTDCVLVVDEDEHLCGIFTAKDLAFRVIGDGMDPRTTPVSAIMTRNPMVTRDTTSATEALTTMVTRGFRHLPVCNDEGDVIGLLDITKVFHESLEKLERAYGSSQKLYNAIEGVQSEFGSGGRGTTPGAVNPLMAYVEALRNKMSFPDLGSILDARTSAATVGVKTSVKEAAVLMREHHTTAVCVMESDGRRIAGIFTSKDIVLRVIAAGLDARTCSVVRVMTPHPDTALPSLSIQEALRKMHDGHYLNLPVVDEAGQLQGCVDVLKLTYATLEQVNSISSEVTNDDSGGPVWNRFFASFGQAGSVDEDNTSIVSGSQLAHSETGPHTPRHKTGHDGQHPGLETPGSELAPNDSASVVQEDYESALAGRSTLQSVTSSQPFTGMVPLDYDDGTYLFKFIAPSGSAHRFQARYDSYDFVREIVVGKIESDPFIADHPSNATTIKPDAQDFQLAYLDDENDLVLITSDRDVTDAVNQAKKQRKDRVVLHLRGGKSWGDSELSKKKAAEVYSTRLKAVTEAEEIENEELEERHSQAGSRDRLSTTRSSKKNAHNQTPVDPTLVAGVPKEFILPAAIGFLGVVILGVFLASKPGH

>fungus-Pb-CBSnPB1-A: JGI database jgi|Phybl1|22406|e_gw1.17.257.1

MLPKPSRSRLPAIATARAETPDVANNGRQRQTKKDEAIRKKLEQELSKKRTGTTRVRQTRKIAGTVSALRPAQALTVKENMLVIEASQLMAAKRSDCVLVVDEDDHLSGIFTAKDLAYRVVAESLDARHTPVSQIMTPGPMCVTADTSATDALNLMVTRGFRHLPVCNEEGDIFGLLDITKCLYEALDKMERAFGSSRKLYDALEGVEREWSGSPVQLAQYMETLRDKMSCPDLNTVLDGSLPAQVSVKVNVRDVARLMKEYHTTAVLVSDREGLAGIFTTKDVVLRVIAAGLNPENCSVVRVMTPHPDTASPHMSIIDALRQMHDGHYLNLPVLEDGRVVGIVDVLKLTYATLEQINSIQGADGEGPMWSRFWDSFGATEHAENESQLSDPTSHHHHLPASQHPMEAISPEPSISFSQLHGFPEISPNESASMVAHNEDNRSANSSHNTRPTKTSGNGGDGSFAFKFTTKGGKTHRIASTPKHSQLLELVRQKVLSEHVPSAAEGEWLSISYLDDEDDEVLITSDADVQDAVRLARKLGQDRVKLFAHDSTSVTAHTAEPPAPVISLSPPSDVSSKTMRQEDDDTSSMTSRSTRKRSSKRKSSRRNESEEPEEYESNTGFPQELILPASIAFLGVVILGVFAFSRVSPKHR

>fungus-Pb-CBSnPB1-B: JGI database jgi|Phybl1|14430|e_gw1.1.956.1

ALRKKLENELSKKRGGSRSLRKHAQRVAGTVSALRPAQALTVKENMLVMEAAQLLAAKRCDCVLVVDEDDHLSGIFTAKDIAYRVVAEELDASHTTVATIMTRGPMCVTSDTSATDALNLMVTRGFRHLPVCNEEGDIFGLLDITKCLYEALDKMERAYGSSRKLYDALEGVEREWSSSPIQLVQYMEALRDRMSCPDLHSVLDGSPPPEVDVKVNVREIAKMMRELHTTAVLVTNREGLAGIFTTKDIVLRVVAAGLHPEKCSVVRVMTPNPDTASPQTSIMDALRKMNDGHYLNLPVMDGHIIVGMVDVLKLTYATLEQMNSIQGNDGEGPMWSRFWDSFGSTDHTETESQLSDPMLSHHPSTNMHPAMHAISPEPSLSYTHLQGYAEILPNESASMVATNDDALSTISSRNIRTHGRDDGTFSFKFTSLSGRTHRFVAPLDSYSQLFDSVLQKVATEHLTLTGKRAGEGAADPSAEWLSISYMDDENDEVLMSSDADVQDAVMLARKVGQSRVKLFIHDAMIESIAAEEAELIDNNDDGEEDTAEEEETEEEYIRKSSKKRSHRRHHYDDGFPRDLLLPAAIVFLGVVVVGVFAISRISPRHR

>fungus-Bd-CBSnPB1-B: FGI database BDEG_01776.1

MKRNSASSASGQKHDTRRTRVGTVSSLRPAPAIIIIETARIIQAAQLMAAKRTDAVLVVGEDGALAGILTDKDIAYRVVAEGLDIRTTPVSSVMTRDPIAVYDKGSRNEALNIMVSRRFRHLPVISETGGGNDDDDNEFDEAAGGTSVVGLLDITKCVFERIDDLERKVNEDLNIISAMEALERRGTVAAEHVGVVRQHHGCPDVGFVLTQTIGGQLEHGTVPEVSIKSSVRDAARIMKAYHTTAVLVIGNSNDDEQIGGIFTTKDIVLRVIAASLDPMTTSVVRVMTPHPDYVLASTSILDALKKLNTGHYLHLPVVDGGVPIGLVDVMTLTISMLTYLMTKDLGTQEGSISEDGPLWNKFWNSTFAGSTIETESDRLSQTSDSRPSGSIHYQPSSSSQHATEMSNRQSLQLQRTLSPHPDEYSSMLSRSQIQNDDPNMFTFKLKDMTRTGNGKVFRFSSRFNSLSEVYAQVCLKTGAKSLIFEAAATSGTTHSMRSEHLDLETKDGEIVRICYMDDENDVVHLESDKDVEEAVLMSRRLGLTRLMIYLGEPLLQNAVTDSQYSSGTATPNVGHHGQVVRYEDSHQSVVSEQSLVEGGNSRINPVVVYNPINGGQGRDPPASIVDYLKDAPLPVNIAISAGIIVVASYLIMKLQR
