# Supplementary material for: Molecular evolution of Phox-related regulatory subunits for NADPH oxidase enzymes
Source: BMC Evol Biol. 2007 Sep 27;7:178. doi: 10.1186/1471-2148-7-178 (PMC2121648; doi:10.1186/1471-2148-7-178)
Supplement: Additional file 14 — Alignments of fungal NOXR, Bem1, Cdc24, and CBSn-PB1 amino acid sequences. Alignments of fungal NOXR, Bem1, Cdc24, and CBSn-PB1 of A. nidulans, M. grisea, F. graminearum S. cerevisiae, C. albicans, Y. lipolytica, S. pombe, C. cinerea, L. bicolor, P. placenta, P. graminis, P. blakesleeanus, and B. dendrobatidis are provided. [file 1471-2148-7-178-S14.pdf]

Additional File 14

Alignments of fungal NOXR, Bem1, Cdc24, and CBSn-PB1 amino acid sequences.

To describe species of sequences, we used the following naming: Mg (*Magnaporthe grisea*), Fg (*Fusarium graminearum*), An (*Aspergillus nidulans*), Sc (*Saccharomyces cerevisiae*), Ca (*Candida albicans*), Yl (*Yarrowia lipolytica*), Sp (*Schizosaccharomyces pombe*), Cc (*Coprinopsis cinerea*), Lb (*Laccaria bicolor*), Pp (*Postia placenta*), Pg (*Puccinia graminis*), Pb (*Phycomyces blakesleeanus*), and Bd (*Batrachochytrium dendrobatidis*). *Gray boxes* indicate predicted PB1 domains. *Red* and *blue letters* indicate the amino acid residues corresponding to the K355 and K382 of PB1 domains of human p67*phox* and four acidic amino acid residues (D289, D291, D293 and D302) of OPCA motif of human p40*phox* PB1 domain, respectively. *Asterisks* indicate identical residues. PB1 domains were identified by Pfam search program (http://www.sanger.ac.uk/Software/Pfam/search.shtml).

Alignment of fungal NOXR proteins

```
Fg-NOXR         MSLKQEIETWVAALGRYDNNEFEEALNEFGKIGDT-------SKILFNMGVIHATLGEHE 53
An-NOXR         MSLKQEIETWVQALEHFDNQEYDLALRSFAAIADT-------SKILFNCGVIYATLGEHE 53
Mg-NOXR         MSLKAELETWAAALKAYDEEDFEKSLDLFSRIADS-------SKILTNIGLIYATLGEHE 53
Lb-NOXR         MSLKAELETWAAALKAYDEEDFEKSLDLFSRIADS-------SKILTNIGLIYATLGEHE 53
Cc-NOXR         MSLKAELETWAAALKAYDDQDFDKALQLFSEIADN-------SKIHTNIGLIYATLGEHE 53
Pp-NOXR         MSLKAELETWASALKAYDEENFDKSLQLFSEIGDS-------SKILTNMGLIYATLGEHD 53
Pg-NOXR         MSLKAEIETWATALELYDQQNFDDALAAFEDIADS-------SKILFNIGLIYATLGEHE 53
Pb-NOXR         MEFKLELDQWLNACAAFDSKDYETAVQSFLSMADN-------AKMHFNIGLIFAIEDDHQ 53
Bd-NOXR         MALKEQLAQWSKALDYWDTNDFFGAYEQFVSFADF-------AKIHYNVGMCAMRTNNLD 53
Yl-NOXR         MLTLQQVVTWLKGVELYSKGDYAPALKQFNQLINSPEYSGEVSKVAHNIAAIYYKMEDHE 60
                *        *                  *              *   *

Fg-NOXR         KAVESYQRAIRLDQYLAVAYFQQGVSNFLLGDFEEALANFNDTLLYLRGN---AMI---- 106
An-NOXR         KAVECYQGAVGLDQYLAIAYFQEGVSNFLLGDFEEALANFNDTLLYLRGN---TYI---- 106
Mg-NOXR         AAVQRFIEATNFDQYLAVAYFQCGVSNFLLARYELAYKDFEEALLYLRGN---QAI---- 106
Lb-NOXR         AAVQRFIEATNFDQYLAVAYFQCGVSNFLLARYELAYKDFEEALLYLRGN---QAMYVVF 110
Cc-NOXR         EAVKHFIEATGMDQYLAVAYFQCGVSNFLLQRYELALKDFEEALLHLRQN---QTMNVSI 110
Pp-NOXR         AAVEQFTAATQLDQYLAVAYFQCGVSNFLLGRYETSLRDFDDALLYLRGN---GDI---- 106
Pg-NOXR         VAIENFRTATSLDQYLAVAYFQCGVSNFLLGMYEEAVKDFEDALMYLRGN---LTI---- 106
Pb-NOXR         RALAAYSRAISMDAYFAVAYFQKGVSQFVMNNMEAAMHDFDNAYQRLRGNEMIN------ 107
Bd-NOXR         EAIAAFSRAITLDEYLSVAYMQIGICQYHMGEFDEALANFEESFKMLRGN---------- 103
Yl-NOXR         RATKWFVTALQKDPYLCITHFLFGQCLFEQEKYSEAAKEWDKCLEYMRGN---------- 110
                 *      *   * *        *                       * *
```

```
Fg-NOXR       ---------------DYAQLGLLFKLYSCEVLFNRGLCYIYLQQMDAGMQDFSYAVKE-K 150
An-NOXR       ---------------DYEQLGLKFRLYSCEVLFNRGLCYIYLQQIGPGMQDLEYASKE-K 150
Mg-NOXR       ---------------NYEQLGLKFRLFSAEVLFNKGLAQIYMGRAQEGLADMEEARRE-K 150
Lb-NOXR       MVEPYLPYSSIPAHRNYEQLGLKFRLFSAEVLFNKGLAQIYMGRAQEGLADMEEARRE-K 169
Cc-NOXR       DDG---------LNSNYEQLGLQFKLYSAEVLFNMGLSLIFMGQIEMGLQNFEAARRE-K 160
Pp-NOXR       ---------------NYEQLGLKFKLYSAEVLFNKGLAHIYMGQVDQGLAELQEARKE-K 150
Pg-NOXR       ---------------DYSQLGLKFRLYSCEILFNKGLSLIYQGAEAEGMIDLQEARKE-K 150
Pb-NOXR       ----------------YTQLGLAFRLYACEVLFNRGVCQLYLGKIDAGLTDLYHAQKA-K 150
Bd-NOXR       ------------FFIDYTQLGMEFQVFACHAIFNIALCHLQRGDTERGVRYISEAIKA-V 150
Yl-NOXR       ------------TWINYQPLGLNFTLYSCEVMFNKSMAKIYSGDILPGVSDLSRAGKEHK 158
                              *  **  *        **             *      *

Fg-NOXR       VVEDHNVIDDAINEQAEGY--------TVFSIPVGVVYRP---NEAKVRNLKTKDYLGKA 199
An-NOXR       YSRSR--------LTPQGY--------TVFSIPVGVVYRP---NEAKVKNLKTKDYLGKS 191
Mg-NOXR       ATDEHNVIDDAIQERGEGY--------TVFSIPVGVLYRP---SEKKLKNSMQKDYMGKA 199
Lb-NOXR       ATDEHNVIDDAIQERGEGY--------TVFSIPVGVLYRP---SEKKLKNSMQKDYMGKA 218
Cc-NOXR       ATEDHNVIDEAIRDQGRGY--------TVFSIPVGVLYRP---SEKKLKNAVQKDYMGKA 209
Pp-NOXR       VTPEHSVIDDAIADRGDGY--------TVFSIPVGVLYRP---AEKKLKNAKTKDYMGKA 199
Pg-NOXR       QTAEHSVIDDAIADRAEGY--------TVFSIPVGVLYRP---AMSKVKNLASKNYLGQA 199
Pb-NOXR       MTEEHDVIDQAVRDRGRGY--------SVFSIPPGVLYRP---SEARMRQLQNANMFAAV 199
Bd-NOXR       PVDSDFVPDIADIEEAERLGENAADTIMPYEVPPNLVFRP---QEDNLRNAERVDYLGQS 207
Yl-NOXR       WSADHNRIDKAYIYFNAIKDSLHRATHTTKQSQNDGLQRSKSLSRSKSQNLSRSKSLARS 218
                                                    *

Fg-NOXR       RLVAASDRANAFTGFAGSEIKNAGKLEVKDDRPADNISFAATNLVKPGLSSR-RQQSEPP 258
An-NOXR       RVIAANRLSTPAD---------------TSQRSVDSVPFATSHLVQKNLTSRSRQQSEPP 236
Mg-NOXR       KLVAASDPNDIFTTFTGSTR-------LKQGISPSGVFIDRPDIESAVIPSVTRSASVPS 252
Lb-NOXR       KLVAASDPNDIFTTFTGSTR-------LKQGISPSGVFIDRPDIESAVIPSVTRSASVPS 271
Cc-NOXR       KLVASSDPDDKITTFVGSAR-------LKMGISPAGIYIDRPDIDTPAGAAVSRSATVPA 262
Pp-NOXR       KLIATEDASEAFTEFTGVAR-------MRQGVSPTGATDFGP-------GGLQRAATVSN 245
Pg-NOXR       KLVAATDASETFVGFTGSAR-------AAAKTTTTGPVMSTPIEQAPMPPAPGMLRRGTV 252
Pb-NOXR       DKLSLPNKNFKTALTSIQRNNSILLPDARFQKRVPGAQAAPRQVQQPPVISSLAEHGMRA 259
Bd-NOXR       KVVASVNATDNYTGFSG--------------KLTRDLTLLSRSKKKDANSGAAKEAALKS 253
Yl-NOXR       KSMKHKQQQLQDNIRNRSSMG--------FGANYTNTFDVSQAEKEAAKQLAQQSSGNVG 270

Fg-NOXR       NGRNVFPPTPPPENERPSRAASVRN----------QKPQLAK--------LNIQQAEPN- 299
An-NOXR       MHRNLFPPTPPPDADKASLSSTGSNGTVRAQPGKAQRPPKLD--------LDRPGAQPAG 288
Mg-NOXR       STAPSRQPADGVRLAGVERSRTAMNPPQSAR-LASNKGPPPR------PPMSAGASIGRS 305
Lb-NOXR       STAPSRQPADGVRLAGVERSRTAMNPPQSAR-LASNKGPPPR------PPMSAGASIGRS 324
Cc-NOXR       SSYPASRPAESIRSAGLERSKTTINVPSNARALTSPQSLSPSSR---LPPVSSAGALGRS 319
Pp-NOXR       PTPPP-KSDDYVRSAALQRANTTIN--VSVDRFRSNPASPAS-------PATSPGSGPPS 295
Pg-NOXR       ATAGSGMGDGPLGPAGMLRRGTSAAITPMMPSSSSGDSPTVG-------IIRRGTTAAR- 304
Pb-NOXR       IARESSRPKMATHIYTDQLPPPTPVSSVSSHYPRKQNTRQYSPIERQRNDSNAGSFSTEE 319
Bd-NOXR       PASFSAAQTTIAQSNTIRVNRKPSVTSISREPIQEGYEQTIT--------MSRSNSATTS 305
Yl-NOXR       PVGLGIRQSPSMRGSPANRPSPNFNNNGGTPNLGRSQTLGGG-------GHTQSSQLHRS 323

Fg-NOXR       RRYEKAASPAD-------ARRPMPRSASTTRTPLQRE---PPPLQLRPKQ---------- 339
An-NOXR       RSTTDLTAPEK-------PRLGTIRTASEPRGQSRQPRGYAPERHVRSSDGYGHRRGASD 341
Mg-NOXR       NTNITPSRPSPNATIGGPVRGLSVRRPGNASPGNSSPPRAPPKDDARLTEFYDDYLDSYG 365
Lb-NOXR       NTNITPSRPSPNATIGGPVRGLSVRRPGNASPGNSSPPRAPPKDDARLTEFYDDYLDSYG 384
Cc-NOXR       NTQITPGRPSPSAGMGGPSRVRHPSFPDRIHADMTKADLPDVPAPERITEFYDDYLDSYE 379
Pp-NOXR       APALS--RNASFAGRMQPTRGLSIRKPGTDAP-------PPSTRGMRTTEFYDSYLDSYA 346
Pg-NOXR       -IESTAARPAP-----VPTNNDLRRRPSEAGSTARLNRSATASSGSKPTSRLAPMAEQRE 358
Pb-NOXR       WADSPHPSISSTSSFSSSLRQRADGRRVDSGFESTHEERYSSSSGRSSTKSHKANRYSPP 379
Bd-NOXR       RKYIPRRLSSN--------NISANTIPGASRVPGTLSRAPSAPLSREGSG---------- 347
Yl-NOXR       QTTGHAHSVAEHPHSSANVHRSQSTRAPVTGTSTTEVDLPSTVKMARMNETYKIYQISSE 383

Fg-NOXR       --IPEETGSPEDVYAMYSATDGYRNSRGSAGSRRLRPQQYSEEED----ASDYEGTINEN 393
An-NOXR       HGFGVSNGHSDDAYGMYGEARAMTLANG---GRPFQQQGYIDEEEEYGSSPCDEDLVPDA 398
Mg-NOXR       GDAPIPPIAQP-GPDRIAAWARTNANPNYPLVRSGSRSAPTSQYTPSSYGGGGSLRRKVT 424
```

```
Lb-NOXR        GDAPIPPIAQP-GPDRIAAWARTNANPNYPLVRSGSRSAPTSQYTPSSYGGGGSLRRKVT 443
Cc-NOXR        DQPPVPQLSAPPTADRVATWARQTPAYPAGVARSTSRSAPNSQYAPSSYGGG-SMRRKPT 438
Pp-NOXR        DAP----SALPPVPEARPAPAAVRRAP------SRARAAPQSTYS------NGSVRRRPT 390
Pg-NOXR        PEKSPRYGSRTRGSLGTESYEDYEDNEPAVPSRSGGPSEDRTTNWARNLSTSGSSSGRNR 418
Pb-NOXR        PVPPIPHNASYEYGDTSANYGNFDLDEMYGSLPTIDTQDNKQDRVRPRLNSNNTAPLEDD 439
Bd-NOXR        --SGQQLEFPLRTLRSAGGSSRMGLGDTQNNVDTAAERLNSLTINNNDLPASPAIKLTTS 405
Yl-NOXR        FCLFSIHDNRLKNIVGCEQMASLLRGKDKEISSGGGVVGASSPPPEDSTEFSFVESVGRS 443

                                                         PB1 domain
Fg-NOXR        DF-EMIGQRRGPGSVS---GSRNSRR--------TEVTKIRVKVH-ADEVKLIMITPDTR 440
An-NOXR        SF-ELMGSRPRARSCSRGPARGYSRR--------PEVRRFRVKVHSFEDTRYILIPPTIE 449
Mg-NOXR        KR-NNPRAPSRVQSTYEEEEEGYVSG--EYEDGPFELTLIRVKLHYQDDTRGMTLTPDTP 481
Lb-NOXR        KR-NNPRAPSRVQSTYEEEEEGYVSG--EYEDGPFELTLIRVKLHYQDDTRGMTLTPDTP 500
Cc-NOXR        RR-ATSRQQNRIGSTYEEEEEGYGSG--DYDDGPFEMNLIRVKLHYQDETRGMTLAPDLT 495
Pp-NOXR        RR-GTVR--SRYE---EEEEEGYGSGEYEYEREEDGFVKIRVKLHYADDVRGMALSPGMP 444
Pg-NOXR        DP-PAVRRPPPANSPYDDPPSDAEYPETVMSGGYAEMTKIRVKLHYGSDTRGMLS--HAM 475
Pb-NOXR        RRGNDQINNNDYSNIRAHPPQPTASVSTPTLKEGSINTKIRVKVHYTDTR-ILLVPHTIT 498
Bd-NOXR        NR------------------------------SNVSTDSIKVKCHYIESR-FIVVPINIS 434
Yl-NOXR        DDVDDIYDIDDYYGTPPASTGQRSANQSVNRSGGSIKSNAATVATGSPPARSKPTPIDVD 503

Fg-NOXR        FETLSDKVRDKFNIKRR-FKIKVKDDDMPNGDMITVGDQDDLEMVIDSVKDEARKQRTET 499
An-NOXR        FAEFETRIREKFGFQMA-LKIKMQDE----GDMITMVDQEDLDLLLMASREIARREGSEM 504
Mg-NOXR        FADFMDKVTAKFGKQINGLGLKFKDED---GGKVTLRDESDYELAIETARESAKGKPEG- 537
Lb-NOXR        FADFMDKVTAKFGKQINGLGLKFKDED---GGKVTLRDESDYELAIETARESAKGKPEAK 557
Cc-NOXR        FNEFIVKITAKFNKSFSGLGLKFKDED---GGKVTLRDESDYELAIETARENAKGRAEGK 552
Pp-NOXR        WEEFLERVTGKFGRELDGLGMKFKDED---GGMVSLRDEMDYELAIETAREQAKGKPEG- 500
Pg-NOXR        IRGFLGKSDQEVFLKSRSSGDEIQGRE---GSMVSIMDDDDWESAVETARSYAKGRAEG- 531
Pb-NOXR        FNELLGRVREKFGAPPS-TRLQYKDEE---EEMVLMIDDDDLHLVRQTSRQKHGDFGVE- 553
Bd-NOXR        FRDLRERVSAKFG-VQGLLSLKYKDEE---EFMTMMVNDDDLDEAVAVSAQG-------- 482
Yl-NOXR        LAREMRKVSFDSSLSTPKLNKLVDKIQGTTRDSVSSTDSIHAPKATKHRVQINYIRPVT- 562

Fg-NOXR        G-------------------------------------------------------KMEI 504
An-NOXR        G-------------------------------------------------------KMEI 509
Mg-NOXR        --------------------------------------------------------RLEL 541
Lb-NOXR        FFDSFSLTH--------------------------CFLIEMGLGGRKIKQRIPDDPRNLS 591
Cc-NOXR        LEICSSLYYGVQYKRKLGEKLVVLRAVHSRKANQLCLDSNMGLSGRKVKQRIGNDPRNLS 612
Pp-NOXR        --------------------------------------------------------RLEI 504
Pg-NOXR        --------------------------------------------------------KVEI 535
Pb-NOXR        --------------------------------------------------------KIEI 557
Bd-NOXR        --------------------------------------------------------RLEL 486
Yl-NOXR        -------------------------------------------------------AKVCS 567

Fg-NOXR        WILQL------------------------------------------------------- 509
An-NOXR        WVEERRMI---------------------------------------------------- 517
Mg-NOXR        WCMDL------------------------------------------------------- 546
Lb-NOXR        WADDAARFGSNYLSKFGWDASQGLGVDGEGRTSHIKVSHKLDMLGIGAAHQKDPNGIAWK 651
Cc-NOXR        WADDAARFGSNYLSRFGWDASKGLGAEGEGRTSHLKVAQKLDMLGIGAAHQKDPNGIAWK 672
Pp-NOXR        WCTDE------------------------------------------------------- 509
Pg-NOXR        WVEDAGFL---------------------------------------------------- 543
Pb-NOXR        WCVT-------------------------------------------------------- 561
Bd-NOXR        WCFLLDPPK--------------------------------------------------- 495
Yl-NOXR        FVAEECSSPDTPITPIPVKKKAVKDYSTRSPYPDSVASSSYEDETTSWSKSLPLLPNDHA 627

Fg-NOXR        ------------------------------------------------------------
An-NOXR        ------------------------------------------------------------
Mg-NOXR        ------------------------------------------------------------
Lb-NOXR        QNKDFENLLKRLNEN----------------------------------ATEIVDGKEEQ 677
Cc-NOXR        QNRDFENLLKRLNAAGGEGGAEVEVKTEVSGFVSSTKEVQVEVKKEEKEKEDEMDVDEKK 732
Pp-NOXR        ------------------------------------------------------------
Pg-NOXR        ------------------------------------------------------------
```

```
Pb-NOXR       ------------------------------------------------------------
Bd-NOXR       ------------------------------------------------------------
Yl-NOXR       AHKTDQRLRELR----------------------------------ERDRREIRDGFIDR 653

Fg-NOXR       ------------------------------------------------------------
An-NOXR_       ------------------------------------------------------------
Mg-NOXR       ------------------------------------------------------------
Lb-NOXR       DVVKEETPKKKRKQKGDEEKDTKKKR------------RKTEDSSPSADEVVPDSLPADP 725
Cc-NOXR       ERKKEKKEKKDKEKKKDKDKEGKRKRSEVDGDEESGSQKKMKTEEASPTPQSSDSTPSEQ 792
Pp-NOXR       ------------------------------------------------------------
Pg-NOXR       ------------------------------------------------------------
Pb-NOXR       ------------------------------------------------------------
Bd-NOXR       ------------------------------------------------------------
Yl-NOXR       DTGMTPPQSHQFDPTNTVAPGRSRNTKFLTVDLDDPNHQGNNGRSKSPGSPMSPFILVT- 712

Fg-NOXR       ------------------------------------------------------------
An-NOXR       ------------------------------------------------------------
Mg-NOXR       ------------------------------------------------------------
Lb-NOXR       APVRVAPRYRAHRARAIAAKSITSKSAAAISEILGIAPSSGLSVTTTQ--------TTGK 777
Cc-NOXR       PKKAFVPRHRAHRARAIAAKSISMKSSTHISEILGIAPESSTTSAAASGSISPAPPDQGK 852
Pp-NOXR       ------------------------------------------------------------
Pg-NOXR       ------------------------------------------------------------
Pb-NOXR       ------------------------------------------------------------
Bd-NOXR       ------------------------------------------------------------
Yl-NOXR       ------------------------------------------------------------

Fg-NOXR       ------------------------------------------------------------
An-NOXR       ------------------------------------------------------------
Mg-NOXR       ------------------------------------------------------------
Lb-NOXR       LTSLTDDA-TIEKLTTSTKSVADYFKEKLLVRA-------------TQSESTTPISEQDA 823
Cc-NOXR       LTVISEDVPELEKITTSTKSVADYFKERLLAKANAKAQSSGSSASTPQPSATKDEEMDEA 912
Pp-NOXR       ------------------------------------------------------------
Pg-NOXR       ------------------------------------------------------------
Pb-NOXR       ------------------------------------------------------------
Bd-NOXR       ------------------------------------------------------------
Yl-NOXR       ------------------------------------------------------------

Fg-NOXR       ------------------------------------------------------------
An-NOXR       ------------------------------------------------------------
Mg-NOXR       ------------------------------------------------------------
Lb-NOXR       YDLPRSGLGSGRARFEIQEDDSEMG-ARRVGLSKFSSLMSSQFLAATSTLSTPPEKTQSE 882
Cc-NOXR       EEKPRGGLGFARVQWESQESTSVQSDTLRMGLSKFSSLMSSTFLS--STFSSSTSESTSP 970
Pp-NOXR       ------------------------------------------------------------
Pg-NOXR       ------------------------------------------------------------
Pb-NOXR       ------------------------------------------------------------
Bd-NOXR       ------------------------------------------------------------
Yl-NOXR       ------------------------------------------------------------

Fg-NOXR       ------------------------------------------------------------
An-NOXR       ------------------------------------------------------------
Mg-NOXR       ------------------------------------------------------------
Lb-NOXR       DVATTQLDMVEEEQTSEEKRKKKKGDKDEKKEKKSKDKVTRTEVKDPDTLQTASLDDVDR 942
Cc-NOXR       ATEEKKETGEEDEKETEEQRKERKRLKKEEKARKKAEKEKRKAAKAEAATSEAKGGDAED 1030
Pp-NOXR       ------------------------------------------------------------
Pg-NOXR       ------------------------------------------------------------
Pb-NOXR       ------------------------------------------------------------
Bd-NOXR       ------------------------------------------------------------
Yl-NOXR       ------------------------------------------------------------
```

```
Fg-NOXR          ----------------------
An-NOXR          ----------------------
Mg-NOXR          ----------------------
Lb-NOXR          KARKAQRKAEKKARKAAENNLH 964
Cc-NOXR          AGKEREKESKKKKKSKKEKKT- 1051
Pp-NOXR          ----------------------
Pg-NOXR          ----------------------
Pb-NOXR          ----------------------
Bd-NOXR          ----------------------
Yl-NOXR          ----------------------
```

Alignment of fungal Bem1 proteins

```
Sc-Bem1          ----MLKNFKLSKRDSNGSKGRITSADIS---------TPSHDNGSVIKHIKTVPVRYLS 47
Ca-Bem1          ----MIKTFRKSKRSSSNS---------------------------------SSPKKTIS 23
Mg-Bem1          -----MKALRRSIKGDKDKQPQ-------------------------------------- 17
Fg-Bem1          -----MKALRRSIKGDKDKGP--------------------------------------- 16
An-Bem1          -----MKALRRSLKGEKDSKPHHHHQ---------------------------------- 21
Yl-Bem1          ----MIKGIRRSLKGEKTTSSGSAN----------------------------------- 21
Sp-Bem1          -----MLKIKRTWKTHS------------------------------------------- 12
Cc-Bem1          -----MKSLRKSLNGNKDSS---------------------------------------- 15
Lb-Bem1          -----MKSLRKSLNGNKDSSS--------------------------------------- 16
Pp-Bem1          -----MKTLRKSLNGHKDNS---------------------------------------- 15
Pg-Bem1          -----MKVLRRSLHKEKDRTSTSSPTKPDFNSHHLPPLSAKHNSKQQQQQQQQQQQQQQQ 55
Pb-Bem1A         ------------------------------------------------------------
Pb-Bem1B         MNLKEKVKPPRSLRSTRLSVVKN------------------------------------- 23
Bd-Bem1          ------MSPELPHRGHLTVAPVR------------------------------------- 17

Sc-Bem1          SSSTPVKSQRDSSPKNRHNSKDITSPEKVIKAKYSYQAQ--TSKELSFMEGEFFYVSGDE 105
Ca-Bem1          RVSS------TSSNQTSHDG-ILQSPKKVIRALYDYEPQ--GPGELKFFKGDFFHVLDDV 74
Mg-Bem1          ------------ISIAPKSAVAIVPPKKVIRALYDYEAQ--STQELSFSRGDFFHVIGRE 63
Fg-Bem1          --------------VTPKSAVAIVPPKKVIRALYDYEAR--SSQELSFSRGDFFHVIGRE 60
An-Bem1          ----HHHQQHHHLSITPKSAVAILPPKKVIKALYDYQPEPGNTQELAFSKGDFFHVISRE 77
Yl-Bem1          ------------SLVSDKNPLHTNPPKMVIRALYDYEPQ--GPGELSFCKGDFFHVISNE 67
Sp-Bem1          -------------RILDKDPFSIEPPRKVIRALYDYTAR--KATEVSFAKGDFFHVIGRE 57
Cc-Bem1          ---KLQISTPLPIPTMSKPPSAILPPQKVIRANSSYKSQ--APQELSFQKGDFFYVLKDA 70
Lb-Bem1          ---KLQISTPVPIPTISKPPSAVLPPQKVIRALFNYRSQ--APQELSFSKGDFFYVLRDA 71
Pp-Bem1          ------PSITTPLPRLSKPSGAVLPPSKVIKALQTYKAN--APQELSFEKGDFFHVLNDV 67
Pg-Bem1          QPTQPFASLAAGNHNLTNPRSIGKPPTLVIKAITAYKSN--RIVELSFQKGDFFHVVGER 113
Pb-Bem1A         ----------------------SLAAPKKIIKALYDYQAQ--GPDELSFSEGDFYHVTARE 37
Pb-Bem1B         -----------KISTPINFQQSAITPKIVAKALFDYKAQ--TQREISFHQGDYFHVDKTD 70
Bd-Bem1          -------------GSSHPASIFNLPPKKVVRANANYTAR--FAGELSFAKGDFFYVINER 62
                                          *     *   *        *  *  *    *

Sc-Bem1          KD----------WYKASNPST-GKEGVVPKTYFEVFDRTKPSSVNG-------------- 140
Ca-Bem1          DDELHKEAEANGWIEATNPMT-QLKGMVPISYFEIFDRSRPTVTAS-------------- 119
Mg-Bem1          NDTD--------WYEACNPALPDARGLVPVAFFQALGRTERDSAQSDTSVTRTS------ 109
Fg-Bem1          NDDD--------WYEACNPALPDARGLVPVTFFQALGRTERDSAQSDGGQP--------- 103
An-Bem1          DDSD--------WYEACNPLIPTARGLVPVTFFEVIGKTQRDSSGS-------------- 115
Yl-Bem1          QDSE--------WFEAFNPLR-NVRGMVPVPYFEVLGKKEGITSPDPNEALLPEERKALH 118
Sp-Bem1          NDKA--------WYEVCNPAA-GTRGFVPVSHFEEIGKTVKS------------------ 90
Cc-Bem1          EGS--------AWYEAHNPVT-GARGIVPRAMFEEFGKNAPPVRHS-------------- 107
Lb-Bem1          DDVG-------TWYEAHNPVT-GARGIVPRNMFEEFNKNIAXTFFDSALRLWYL------ 117
Pp-Bem1          NNEG-------VWYEAHNPAT-GARGLVPCHMFEEFGR---------------------- 97
Pg-Bem1          DDHEG------SWFEASNPAT-GARGIVPKDCFELFGRKQNELPSTTPTATAFP------ 160
Pb-Bem1A         NDSK--------WFEACNPAT-NTKGLVPVAFFQVLDKAERN------------------ 70
Pb-Bem1B         N-PH--------WFEAFNPVT-HAKGIVPVNYFQILEKNDTP------------------ 102
Bd-Bem1          PDGLN--------YEVINPIQ-RIRGIVPTSHFASLDKVQQEANARNPDYNGDDQNNYGY 113
```

```
                                  **      * **   *

Sc-Bem1         ----------------------SNSSSRKVTNDSLNMG---------------------- 156
Ca-Bem1         ----------------------SNSFTNSIDIQHQHQQG-----------IHNGTGNRNL 146
Mg-Bem1         APVPSKNPDH----------DSGYAETSAPVPSTPGFS----------NRDSKS-GKSG- 147
Fg-Bem1         -PSPPKNPDH----------DSGYSESPAMHPAPAVMSPTTNSAPQGHQRNSKSVGKTG- 151
An-Bem1         IDLGKKKEPH----------DSGFADRATSSGSEFGSGSSKSYQSHPAFPRTSMMGKAGG 165
Yl-Bem1         RSSQSKHAQHSPQHVLANGLDVSQSMQNLSLGGASGSGGHTREPSMAGASDRSSQGKST- 177
Sp-Bem1         -ERDSDGSGQ-----------ISFTDLTTNSSTT---------------RSSISELHSGS 123
Cc-Bem1         ----------------------QFSTPGGGGFKSMHLP----------GSGSAPSPPTPK 135
Lb-Bem1         --IPS---------------PLRVRTSQVGPRTALPSP----------SRPESSIPTTPK 150
Pp-Bem1         -----------------------------GPSKPRVAD----------AGAADRPLSTVK 118
Pg-Bem1         --PRSSAPGYQ---------SSANNPPGSPLLVRTPLS----------AEPFRQSHPNSK 199
Pb-Bem1A        ----------------------------LAVAQPLNDLNIKSDSGFSDSADTMLPTPSKK 102
Pb-Bem1B        ----------------------------NHLGDPT-------------RTETQLP-PVKK 120
Bd-Bem1         TDDGYDDSYNN----HHTSDPYGQPPNNNYMGVNHGRSPLSPASPGSNQAHGISASGSGR 169


Sc-Bem1         --SLYAIVLYDFKAEKAD-------ELTTYVGENLFICAHHNCEWFIAKPIG-------- 199
Ca-Bem1         NQTLYAVTLYEFKAERDD-------ELDIMPNENLIICAHHDYEWFIAKPIN-------- 191
Mg-Bem1         -AMVYGIVMYDFQAERAD-------ELQAQAGEAIIVIAQSNPEWFVAKPIG-------- 191
Fg-Bem1         -AMVYGIVMYDFAAERAD-------ELEAKAGEAIIVIAQSNPEWFVAKPIG-------- 195
An-Bem1         GAMVYGVVQYDFQAERPD-------ELDAKAGEAIIVIAQSNPEWFVAKPIG-------- 210
Yl-Bem1         -PTLHGVVLYDFQAERPD-------ELQASKGDNIIIQAQSNHEWFVAKPVG-------- 221
Sp-Bem1         -QPLFGIVQFDFAAERPD-------ELEAKAGEAIIIIARSNHEWLVAKPIG-------- 167
Cc-Bem1         HQVYYAIVMHDFSAERPD-------ELDAKRGDAITVVAQSNREWFVAKPIG-------- 180
Lb-Bem1         TQVFYAIVLHDFVAERAD-------ELDAKRGDAITVVAQSNREWFVAKPIG-------- 195
Pp-Bem1         QQAFYAIVLHDFTAERAD-------ELDAKAGDTITVVAQSNREWFVAKPIG-------- 163
Pg-Bem1         TQPLYGIVQHDFVAERPD-------ELDAKRGEPIIVIAQSNHEWFVAKPIG-------- 244
Pb-Bem1A        TSHLYGVVLYDFQAERSD-------ELNAKEGEPIIVIAQSNPEWFVAKPIG-------- 147
Pb-Bem1B        MQPLYGVVLYDFQAERFD-------ELDARAGESILVIAQSNVEWFVAKPIG-------- 165
Bd-Bem1         GDKQLWSVLVQRSEQRDDGLWWFTVELKMTDNSSNILFRTYDDFWILQVSLLNHFPSESG 229
                                  *       **                 *


Sc-Bem1         RLGGPGLVPVGFVSIIDIATG--------YATGNDVIEDIKSVNL--PTVQEWKSNIARY 249
Ca-Bem1         RLGGPGLVPVSYVKIIDLLNPNSHYTSIDTSRRSQVIQVINGFNI--PTVEQWKNQTAKY 249
Mg-Bem1         RLGGPGLIPVSFIEIRDMASNT------PVPDP---EGAVRRAGI--PRVEEWKKMAAEY 240
Fg-Bem1         RLGGPGLIPVSFVEIRDMASDK------AIQNP---GDAIKRAGV--PKVEEWKKMAAEY 244
An-Bem1         RLGGPGLIPVSFIELRDMQSGQ------AVADP---LEAVRRAGV--PKVEEWKKMTAEY 259
Yl-Bem1         RLGGPGLIPASFIEIRDIVTGK------AVVDV---ESALRASGL--PKVEEWKRMAADY 270
Sp-Bem1         RLGGPGLIPLSFIQLRDLKTGA------VIKDV--SEAVLRISCI--PRVEDWKRAAADY 217
Cc-Bem1         KLGRPGLIPVSFVEIHDPTTGA------PIVD--V-DALIDRGDL--PKVEDWKRAMLNY 229
Lb-Bem1         RLGRPGLIPVSFVEIHDPSTGK------AIPD--V-QGLMDRGEL--PKVEDWKRAMLNY 244
Pp-Bem1         RLGRPGLIPVSFVEVRDPATGL------AVQD--I-MALIDGGAL--PRVEEWKRAMLNY 212
Pg-Bem1         RLGGPGLIPVSFVEVQDVNTGK------ALAPNQV-QDLIRSSVV--PAVEEWKKATAAY 295
Pb-Bem1A        RLGGPGLIPVSFVQVRDAVSGQ------IITNVSN-LRQTSSNFI--PNVEEWKKMTQGY 198
Pb-Bem1B        RLGGPGLIPVSFVEIRDALTGH------TITSSQ------PSNPL--PRVEEWKRMTMGH 211
Bd-Bem1         RLDQPRTIPFLPTPTRTLTPEQAQQRRHQLDSYMQELIKLPSHIMNAPSVKRFLMVRSGD 289
                  *  *   *                                      * *

Sc-Bem1         K---------ASNISLGSVE---------------------------------------- 260
Ca-Bem1         Q---------ASTVPLGSISGSGTPPT--------------------------------- 267
Mg-Bem1         K---------NSSITLGKFEVGGAPQQ--------------------------------- 258
Fg-Bem1         K---------NSSITLGKFEGGGPGQPG-------------------------------- 263
An-Bem1         K---------NSSISLGKIETTGSMQS--------------------------------- 277
Yl-Bem1         K---------ASSIALGNFDDPQQHSQQ-------------------------------- 289
Sp-Bem1         K---------KSSIPLGKFSD-GETQTM-------------------------------- 235
Cc-Bem1         K---------QNSISLGVIDPPSRG----------------------------------- 245
Lb-Bem1         K---------QNSIALGVIDPPSRG----------------------------------- 260
Pp-Bem1         K---------ANSISLGVLEESVAAN---------------------------------- 229
Pg-Bem1         K---------GNSIPLGKFDFAATSPPMPSATNGGSFNHPTSSRSASTSHTRQPSGAGYD 346
Pb-Bem1A        ---------EASSIPLGRIDKQPQQQLQTQ------------------------------ 219
```

```
Pb-Bem1B       DNPEKLHNPSSSALYYAPNTIDTFSRIPTH------------------------------ 241
Bd-Bem1        LDTPMNIRFDVSETLLDLLTEYQEEADLRIKLVLG------------------------- 324
                                          (PB1 domain of Bd-Bem1)

Sc-Bem1        ----------------------------QQQQQSITKPQNKSAKLVDG------------ 280
Ca-Bem1        ----------------------------SANSQYFDNHTMTSNRSSSGS----------- 288
Mg-Bem1        ----------------------QAIEQGMDR-MSLQP--TDSR--SSQGQLNGKLQQ--A 289
Fg-Bem1        ----------------------QGIEQGMER-MSIQQQ-QHSRQPSQNGTQSGYASQ--P 297
An-Bem1        ------------------------VTSGMER-MSVRQSADHVSHMSQNGHANAYHNRNAS 312
Yl-Bem1        ----------------------QALQHALRGQQTLQEHQVQQEHQTLQERQSLQEQQ--A 325
Sp-Bem1        ----------------------PSLSPSTENLQINNDVTYQAATDNSSTFPGSVANELTP 273
Cc-Bem1        ---------------------------GPPTSPFLS--TMAEPMDPPREPEPP-----RP 271
Lb-Bem1        ---------------------------SVQSNSYSPREDYQSPPQPQAASEPA-----RA 288
Pp-Bem1        ----------------------FAKAQATKQMPPLPTATPPPPSQPQPVKQKPPSPISRP 267
Pg-Bem1        RPPSGRGPQHGSWHGAGGDPSAYDHSRQSSANTPWNAPTNSYSNSRQSPTQQGRDYQDEP 406
Pb-Bem1A       -----------------------QQSQSQFQSPIQDTINEGRSVKSGSSCPTHRPLSEEY 256
Pb-Bem1B       -----------------------NSVNTLLGSSNAGSGAGGSASASASAGAIITTAEQIA 278
Bd-Bem1        -EEIICWKEPDYISFEELMYHAEERLEFRFQALMYKDECEQLIPLRSNTDLRLLVTTLSK 383

Sc-Bem1        ----------------------ELLVKASVESFGLEDEKYWFLVCCELS----------- 307
Ca-Bem1        ---------------------SISIIEASVDSYQLDHGRYQYSITARLN----------- 316
Mg-Bem1        MQPQNAYPTP------KPSTQLYAPVQASIPRYCFAEEKYWFVIEAILE----------- 332
Fg-Bem1        RTSQQPQPHQSQV--QKPAAPLHAPLEARIPRYCFAEDKYWFVIEAVLE----------- 344
An-Bem1        KASIAPSTHQQPP--PHSQQPLVAPIAASIPRYCFDNDKYWYIIEVKME----------- 359
Yl-Bem1        LREQQALREN-----YAAPATGPLVVAASVESFALHSGRYWYLVNAELE----------- 369
Sp-Bem1        LQTLESRTASIASKNKKDMSSEPTVVAAMVENYMIRDDQYWYLVRAVMS----------- 322
Cc-Bem1        QTPECLPEG--------------ILLSADVVSFHYEMEEYWFRVDAIFQPYSSPGSGQLP 317
Lb-Bem1        QSPDILPEG--------------ILLSADVVSFHYEMEEYWFHQN------------VLP 322
Pp-Bem1        ISPKLLPEG--------------ILLSADVKSFHFEMEEYWFRIHAVFQPYDPSDSHSLP 313
Pg-Bem1        YPPQSGPEGDGYATVDELRERYGVVVHASVESFHFEQGHYWFHLRAHFSRIS--EDGRDE 464
Pb-Bem1A       LSRHNSTSRSRTATADGTQHTQAVVVSAVIDSFILEGDQYWFVVYTRLS----------- 305
Pb-Bem1B       QEIGNLSIGPRDNSRD---NTRSRVVAATVDSYVPEGDQFWFIVFAQMS----------- 324
Bd-Bem1        KLTFYGSKKSSNRLNGRTSDPASTVRTASQLTMQLESPANSNTTVPTLN-------IADS 436
                                          *

Sc-Bem1        NGKTRQLKRYYQ----------------DFYDLQVQLLDAFPAEAGKLRDAGGQWSKRIM 351
Ca-Bem1        NGRIRYLYRYYQ----------------DFYDLQVKLLELFPYEAGRIENS-----KRII 355
Mg-Bem1        DGRKYELSRYYE----------------DFYDFQIALLTEFPAEAG-----NTGTQKRTL 371
Fg-Bem1        DGRQWELSRYYE----------------DFYDFQIALLTEFPAEAG-----NTGTQKRTL 383
An-Bem1        DGRCWELSRYYH----------------DFYDFQIALLTQFEEEAG-----NRG-KPRTL 397
Yl-Bem1        DGSMRRLCRYYQ----------------DFYVFQINLLGVFEEAAG-----HND-QPRLL 407
Sp-Bem1        DGKHRNLCRYYE----------------DFFNFQTKFLELFPNEAG-----RGD-ERRVI 360
Cc-Bem1        PAKQLILFRVYE----------------DFYEFQVTLLRTFPREAG-----QQPPHPRVL 356
Lb-Bem1        PAKQLILFRVYN----------------DFYDFQVSLLDNFPREAG-----REPPHPRTL 361
Pp-Bem1        PAKQLVLFRSYN----------------DFYDFQVEILNAFPHEAG-----RPDVNTRIL 352
Pg-Bem1        QTTVLVLYRLYE----------------DFYDFQTALIETFPDQAE-----DEDGAP-NL 502
Pb-Bem1A       NNTHRILYRLYE----------------DFYDFQINLLNDYPVEAG-----KAK-RERIL 343
Pb-Bem1B       DRRHRVLYRLYEALNVHAFVCVLGGLRTDFYDFQVTLLQDYPIEAG-----KTG-KPRIL 378
Bd-Bem1        VNSTTGAVQVSE----------------NRVNQDTVPVGSLSASAGQG-IRMARSLPRTI 479
                                                           *

Sc-Bem1        PYIPG--------------PVPYVTNSITKKR----KEDLNIYVADLVNL----PDYISR 389
Ca-Bem1        PSIPG--------------PLINVNDSISKLR----REKLDYYLSNLIAL----PSHISR 393
Mg-Bem1        PYMPG--------------PVNYVTDAITEGR----LHNLDAYVKNLLAQ----PTYISR 409
Fg-Bem1        PYMPG--------------PVNYVTDAITEGR----LHNLDAYVKNMLNQ----PPYISR 421
An-Bem1        PFMPG--------------PVAHVTDAISDGR----RHNLDEYIKKLLAM----PPHITR 435
Yl-Bem1        PFMPG--------------PLTYVNDSISMQR----RHNLDEYVRKLMAL----PAYISR 445
Sp-Bem1        PYMPG--------------PVDDVNELISSQR----AMDLDVYLKEMCRL----PARLLE 398
Cc-Bem1        PYMPG--------------PAKDVDDALTATR----REELDEYIHGLCALSRSGYRHILE 398
Lb-Bem1        PFMPG--------------PAQDVDDALTSTR----RGELDEYIHGLCALGKSGSRNILE 403
Pp-Bem1        PYMPG--------------PSDQVDNEVTAAR----RIELDDYLHKLCELQLYS-RYILE 393
Pg-Bem1        PRMPG--------------PSDNVDELVCAQR----VEDLSTYLHELCEL----PLYIRE 540
Pb-Bem1A       PYMPG--------------PLAVIDQEITAER----QRDLNTYCKDLLGL----PRYISE 381
```

```
Pb-Bem1B        PFMPG--------------PQTEVDERLTAKR----QTELNIYCQELFQL----PRYLVE 416
Bd-Bem1         PSPPTGLLRDNTTVVPATIPESIMSTSIPRRQSVSDNAGASSYHLSLKINRQSSPSHHSQ 539
                *  *               *                      *

Sc-Bem1         SEMVHSLFVVLNNGFDREFER--------------------------------------- 410
Ca-Bem1         SEEVLKLFDVLDNGFDRETD---------------------------------------- 413
Mg-Bem1         CNLVK-QFFAPREGDYEMD----------------------------------------- 427
Fg-Bem1         CNLVR-QFFAPREGDYEID----------------------------------------- 439
An-Bem1         CALVR-QLFAPRPGDFEID----------------------------------------- 453
Yl-Bem1         SVIMQ-ELFALRPGDVAAAG---------------------------------------- 464
Sp-Bem1         NELVK-LFFLPLDGDVESP----------------------------------------- 416
Cc-Bem1         HEVLR-QFLALKPGDVDSEIDPRVDEIDALFGEYPED----DYDREYAQEIADQVGRMKV 453
Lb-Bem1         HLVVR-KFLALKPGD---------------------------------------MGRMRV 423
Pp-Bem1         DKLIR-EFLALKPGDAEVEVEPRMKEIAALARAPDANGQAHESDNVDPEYGVTQISQLRI 452
Pg-Bem1         SELVY-EFLGPREGDVELEGDPGNLGLDDRSPTEVEG---------------EVVEYLQR 584
Pb-Bem1A        GDLVQVQLFGIHEGDIETD----------------------------------------- 400
Pb-Bem1B        SPLVQDVLFVLHEGDVEID----------------------------------------- 435
Bd-Bem1         FRPFATTSTLESPARSALDASQPLSSLKPFISGSMNAR------------------RDGF 581

Sc-Bem1         -----------DENQNNIKTLQENDTATFATASQTSNFASTNQ----------------- 442
Ca-Bem1         -----------AINKRFSKPISQKSNSHQDRLSQYSNFNVLQQQQQQQ------------ 450
Mg-Bem1         ----------ANGEEDEYRMSGGSQQSS--PDSPNGGISQQSSRNNL------------- 462
Fg-Bem1         ----------PNDNENEYRLSQGSQLSS--MESPAGG-----SQNNL------------- 469
An-Bem1         ----------PSAFGEDARFSGGSQHSSGHELSPSASRQSSQQQNGP------------- 490
Yl-Bem1         --PTDDLPQPLTPETESFDAAQAEARAKGIHPKSLGPTNVSSSSVSY------------- 509
Sp-Bem1         --------------HPTSTMPEALPREP-------------------------------- 430
Cc-Bem1         SDSG--HHSDGSDYEDEGYAASPQHRPYDR--HPYGQTAPVNGRHSR-----EESL---- 500
Lb-Bem1         QDD---QRSDGSDYEDEGYAPSLQQKAYDRGRHPYVQGS---ERSSQ-----DNTF---- 468
Pp-Bem1         SGEANGQGSDGSDYGD----GDVMNSPYDRDSSAYVYGEKEPSRMST-----EQTYNTTQ 503
Pg-Bem1         MDSSRGEDGRLADLNESISRLSTGSNGPLEAHHQYQHHRRQSSQASQPSHHP-------- 636
Pb-Bem1A        -----------HDPRSDP-----------------------AMFT--------------- 411
Pb-Bem1B        -----------YDPRTGAPRPESVELSHSILIPEAFGSSTMNTFTPF------------- 471
Bd-Bem1         PINTRPLSSGSPASISHLRHYQAMQRRSSSCSPNKTGSHGSNTDSEIGDVLTEDEVALMI 641

Sc-Bem1         ------------------DNTLT----GEDLKLNKKLSDLSLS----------------- 463
Ca-Bem1         ------QQQQYAHHSRGSDNSPTNESSGSNLINSSSHNDSSLS----------------- 487
Mg-Bem1         ------SSGGYNNNNLAPSQQRGLSAQPPMMRQASSLSQPSQA----------------- 499
Fg-Bem1         ------NGSGYG---LAPS-------HPDMQTQASSLSQPSQA----------------- 496
An-Bem1         ------SERSSHSHSHSHSHSHQRSQTSSSIPNGKPPMVRQQS----------------- 527
Yl-Bem1         ------SEGTEESRPVSTASGLMGPPEPTGLPGGGHQRMTSVS----------------- 546
Sp-Bem1         --------------------------LSFSLPEKAPEKATNIS----------------- 447
Cc-Bem1         ------RVQAHQNHQRAGSTSSFHTHQTQHTQSYAS-HSRNHSSERISPISRDSYSNGQS 553
Lb-Bem1         ------RLQGQNHQRNNASTPSTASFRSSQAMSSRS-HSHSHS----------------- 504
Pp-Bem1         PLRPRSRTNGHERTENGISLYGRSSPGPVAALRYDSYHAHSQS--------RSSVASSQE 555
Pg-Bem1         --------SRMSKGSTGPAGYHQRGQRSQDLRPFSSSTTSSEG----------------- 671
Pb-Bem1A        ------------------QQPQQSVSQPMPGAIVGGAYYDT------------------- 434
Pb-Bem1B        ---------------GRNSNPIKDISQTNTATATATATASTTT----------------- 499
Bd-Bem1         SEHLVNGDEMGGSEVGGGTASDRDRRSSSRLDGEGLDGDHRMSG---------------- 685

                                                              PB1 domain
Sc-Bem1         ----------------------GSKQAP---------AQSTSGLKTTKIKF--YYKDDIF 490
Ca-Bem1         --------------------SSPPPPQTVTTTNTTITTDSSSKQPKVKVKF--YFDDDIF 525
Mg-Bem1         ----------------------------SLS---PGIPQAG---AFMKVKL--SYNGDLI 523
Fg-Bem1         ----------------------------SLG---GGMQAPSQPPSAMKIKM--YFNGDLI 523
An-Bem1         ----------------------------SLT-----QVSTSSG-GALKVKV--NFQDDLI 551
Yl-Bem1         --------------------------TVGPTPDNPAVNTPQSSSNSVKVKV--FYQDDLI 578
Sp-Bem1         ----------------------------IPE-----SAPTTAG-STCKVKV--RLGDETF 471
Cc-Bem1         RWTDHTQPTPTTANSFSSASRTRSQTATSVNLNTPPISAANPQTAFVKIKIFDRVADDLI 613
Lb-Bem1         ---------------------------TTTNLNNPPISAANPQTAFVKIKIFDRVSDDLI 537
Pp-Bem1         PSPVSTRASHAGSTATSRTSGSGRSRSQSNATYNPPISASNPQIAFMKIKIFDKSAEDIV 615
Pg-Bem1         -FSSHLPSTHTSTSSVPVGSRVTSLTPAGPGEHPGSANHNNPSTGFLRIKIYQRQTDDLI 730
Pb-Bem1A        ----------------------------------------------IKIKIVHK--DDIF 446
```

```
Pb-Bem1B        ------------------------------------TAAKNAIGSSNLIKVKIAYKEEMF 523
Bd-Bem1         ------MSFGSVSHGLLGGDVTRNIYRWKETRDIERQRRNSEPDLLTIP(KR)SPSGFEIARA 739

Sc-Bem1         ALMLKGDTTYKELRSKIAPRIDTDNFKLQTKLFDG----SGEEIKTDSQVSNIIQAKLKI 546
Ca-Bem1         VLLIPTNLRLQDLKSKLFKRLELDITYKYEKPDQQQKPTSESIHLFLKNDFEDFLIENET 585
Mg-Bem1         AIRVPSDIQFRELFDKITERLRIQPGEEVQLSYKDDITNGKLALMNDQDLIYAMQRNEKL 583
Fg-Bem1         AIRVPTDISFQALYDKICDRLKIPANEEIQLFYKDEPTGDKPSMISDNDLDFALQRNEKL 583
An-Bem1         AIRVPSDINVQQLKEKLMDRLKIN--DEIVVQYKDEASGAYVDLISDSDLDTAIQRNSKL 609
Yl-Bem1         AIRVPSDIDYATLHDRLCERLRVET---LSLLYKAD-DGSRVVISNDEDLGAAVYGKNKL 635
Sp-Bem1         ALRVPSDISFEDFCERLTNKLGECE----HLSYRDTNANKVLPLNNVDDLRKACSQESGV 527
Cc-Bem1         AIRVHPKVSHHELMEKVQARLGSELR---VLKYRDSLTNTFVGLDTDEELRAWMEGTDKH 670
Lb-Bem1         AIRVHPKVTHSELMDKVQARLGGEVG---NLRYRDSMTNTFVGLDSNEELRSWMEGTDKH 594
Pp-Bem1         AVRVHPRVTYAQLLEKASQRLNCHVE---RLRYLDSETRQFIDLETDEDLRFWLDDTEKH 672
Pg-Bem1         AIRAPTGVQFLELLQRIQERVGAEVR---SIRFRDE-SGVAYSTGGSVPINANNGARLIG 787
Pb-Bem1A        AIKVPANTTLDVLRDRIHDRLG-FNVQ---LNYKDEITGESMELSEERDMEEA------- 495
Pb-Bem1B        AIKVPIDCSFQKLHDRVQERLGGSNVR---LRYKDQTQGESLPLETESDIAEAFESSLK- 579
Bd-Bem1         STLMEPGMFRRQFLQNKAQRERKRPPMFITRNFIDFLALYGVYGGDVYPSDDDEDTDSNA 799

Sc-Bem1         SVHDI------------------------------------------------------- 551
Ca-Bem1         SNNNNSEIDFENEIIKEKLGEFEVNDDEKFQSILFDKCKLMVLVY--------------- 630
Mg-Bem1         LFYHRYSNSFDR------------------------------------------------ 595
Fg-Bem1         LLYVEAV----------------------------------------------------- 590
An-Bem1         TLYVGLA----------------------------------------------------- 616
Yl-Bem1         VLYAS------------------------------------------------------- 639
Sp-Bem1         LLFAERRRF--------------------------------------------------- 536
Cc-Bem1         VLYAD------------------------------------------------------- 675
Lb-Bem1         VLYA-------------------------------------------------------- 598
Pp-Bem1         LLNKRRPLTPTRPHRKMTETDNASAAVDAMIRGALDLDDHGISDDSIQLSNDSSASLSQE 732
Pg-Bem1         IDNDGDLDRWINSGSRLVLYVD-------------------------------------- 808
Pb-Bem1A        ------------------------------------------------------------
Pb-Bem1B        IGKLILYATPL------------------------------------------------- 590
Bd-Bem1         TGFAIDSEDLVDEEMGRGGRSERLTYEEVGRNHHDGRFARNQTCIGTDAYHDECDYDHAR 859

Sc-Bem1         ------------------------------------------------------------
Ca-Bem1         ------------------------------------------------------------
Mg-Bem1         ------------------------------------------------------------
Fg-Bem1         ------------------------------------------------------------
An-Bem1         ------------------------------------------------------------
Yl-Bem1         ------------------------------------------------------------
Sp-Bem1         ------------------------------------------------------------
Cc-Bem1         ------------------------------------------------------------
Lb-Bem1         ------------------------------------------------------------
Pp-Bem1         DNDLRDDDKALEWHEVIELQAFSERKAWIEEKITFLGKMPPIEVFAGLDAVRSSAVEVPG 792
Pg-Bem1         ------------------------------------------------------------
Pb-Bem1A        ------------------------------------------------------------
Pb-Bem1B        ------------------------------------------------------------
Bd-Bem1         TSETALLLAQSGLGTNGIATVPGDGGNTVGVHGTTSSSHTGTSESKAFFMLLKAFVGTGV 919

Sc-Bem1         ------------------------------------------------------------
Ca-Bem1         ------------------------------------------------------------
Mg-Bem1         ------------------------------------------------------------
Fg-Bem1         ------------------------------------------------------------
An-Bem1         ------------------------------------------------------------
Yl-Bem1         ------------------------------------------------------------
Sp-Bem1         ------------------------------------------------------------
Cc-Bem1         ------------------------------------------------------------
Lb-Bem1         ------------------------------------------------------------
Pp-Bem1         LPTRAELEEWLAEHDRIEKETEIFDSGELRKLKKFTKAAAQRNLSPEDTDLIELTLTTIY 852
Pg-Bem1         ------------------------------------------------------------
Pb-Bem1A        ------------------------------------------------------------
```

```
Pb-Bem1B        ------------------------------------------------------------
Bd-Bem1         LFLPKGFLNGGLGFSMVLLVVLGYLTLHCMILLVDTSRSLGGKSFGDIGGHIYGPYMRQL 979

Sc-Bem1         ------------------------------------------------------------
Ca-Bem1         ------------------------------------------------------------
Mg-Bem1         ------------------------------------------------------------
Fg-Bem1         ------------------------------------------------------------
An-Bem1         ------------------------------------------------------------
Yl-Bem1         ------------------------------------------------------------
Sp-Bem1         ------------------------------------------------------------
Cc-Bem1         ------------------------------------------------------------
Lb-Bem1         ------------------------------------------------------------
Pp-Bem1         ELDKLFHLLRDRSDNLDLLGIRLTWEERRMAAWSELRSVLSDIREFLASRARWTPNAYDS 912
Pg-Bem1         ------------------------------------------------------------
Pb-Bem1A        ------------------------------------------------------------
Pb-Bem1B        ------------------------------------------------------------
Bd-Bem1         VLASIAISQMGFCCAYFIFVGQNLRDLLMVSSGCRIIWPDWVFILIQLAVYIPLSWVRRI 1039

Sc-Bem1         ------------------------------------------------------------
Ca-Bem1         ------------------------------------------------------------
Mg-Bem1         ------------------------------------------------------------
Fg-Bem1         ------------------------------------------------------------
An-Bem1         ------------------------------------------------------------
Yl-Bem1         ------------------------------------------------------------
Sp-Bem1         ------------------------------------------------------------
Cc-Bem1         ------------------------------------------------------------
Lb-Bem1         ------------------------------------------------------------
Pp-Bem1         VPVEDELMPPEPETKRRNSVVSINSTFSESSSNLPPLVLSRTSRYKLAESLSRNAAQFAS 972
Pg-Bem1         ------------------------------------------------------------
Pb-Bem1A        ------------------------------------------------------------
Pb-Bem1B        ------------------------------------------------------------
Bd-Bem1         KNFGITSLIADVFILLGLGYIFMYDLSVIGQTGIKPTAWINIESFSLFVGTAMFAFEGIC 1099

Sc-Bem1         ------------------------------------------------------------
Ca-Bem1         ------------------------------------------------------------
Mg-Bem1         ------------------------------------------------------------
Fg-Bem1         ------------------------------------------------------------
An-Bem1         ------------------------------------------------------------
Yl-Bem1         ------------------------------------------------------------
Sp-Bem1         ------------------------------------------------------------
Cc-Bem1         ------------------------------------------------------------
Lb-Bem1         ------------------------------------------------------------
Pp-Bem1         RVSSLRHSKIAGAGKALDKLIDHSRRPVPDELLDEQDKLEDHGINEMEDVGKFVMTVVMQ 1032
Pg-Bem1         ------------------------------------------------------------
Pb-Bem1A        ------------------------------------------------------------
Pb-Bem1B        ------------------------------------------------------------
Bd-Bem1         LILPIAESMQHPQKFSSVLSWCILLIGTIFITIGTLGYMSFGDQIETVLFLNLPQNPLVN 1159

Sc-Bem1         ------------------------------------------------------------
Ca-Bem1         ------------------------------------------------------------
Mg-Bem1         ------------------------------------------------------------
Fg-Bem1         ------------------------------------------------------------
An-Bem1         ------------------------------------------------------------
Yl-Bem1         ------------------------------------------------------------
Sp-Bem1         ------------------------------------------------------------
Cc-Bem1         ------------------------------------------------------------
Lb-Bem1         ------------------------------------------------------------
Pp-Bem1         WKKADEIYVETMKDKSAAQTLLEEIEVATSSHPTSRQDVAFLSRATALAKRLAMRDNPAS 1092
Pg-Bem1         ------------------------------------------------------------
Pb-Bem1A        ------------------------------------------------------------
```

```
Pb-Bem1B        ------------------------------------------------------------
Bd-Bem1         SIQFFYAVAIMLSFPLTIYPVIRITEQKLFGHYSRTGKSSPVVKWQKNLYRAVLACMLGV 1219

Sc-Bem1         ------------------------------------------------------------
Ca-Bem1         ------------------------------------------------------------
Mg-Bem1         ------------------------------------------------------------
Fg-Bem1         ------------------------------------------------------------
An-Bem1         ------------------------------------------------------------
Yl-Bem1         ------------------------------------------------------------
Sp-Bem1         ------------------------------------------------------------
Cc-Bem1         ------------------------------------------------------------
Lb-Bem1         ------------------------------------------------------------
Pp-Bem1         SGTTFPLPTHLLFPDQSGSNEEVIRMLSSELAAAGEHVRKAEQCAKEYHTSLEAVKRVET 1152
Pg-Bem1         ------------------------------------------------------------
Pb-Bem1A        ------------------------------------------------------------
Pb-Bem1B        ------------------------------------------------------------
Bd-Bem1         ISWAGSTSLDKVVSLVGCFACIPLSFIYPALFHLHITTSWWARVTDWMLVGFGTVAMVYT 1279

Sc-Bem1         ------------------------------------------------------------
Ca-Bem1         ------------------------------------------------------------
Mg-Bem1         ------------------------------------------------------------
Fg-Bem1         ------------------------------------------------------------
An-Bem1         ------------------------------------------------------------
Yl-Bem1         ------------------------------------------------------------
Sp-Bem1         ------------------------------------------------------------
Cc-Bem1         ------------------------------------------------------------
Lb-Bem1         ------------------------------------------------------------
Pp-Bem1         ACKAASDLSVQLESIVQRLKDGVPSDAGDGTPPDLSTEACLEGSRHASFLTVLPSILQEL 1212
Pg-Bem1         ------------------------------------------------------------
Pb-Bem1A        ------------------------------------------------------------
Pb-Bem1B        ------------------------------------------------------------
Bd-Bem1         TFVTLEQWAINGPDTPRDRCHDAMGTQLLELPSFGLW----------------------- 1316

Sc-Bem1         ------------------------------------------------------------
Ca-Bem1         ------------------------------------------------------------
Mg-Bem1         ------------------------------------------------------------
Fg-Bem1         ------------------------------------------------------------
An-Bem1         ------------------------------------------------------------
Yl-Bem1         ------------------------------------------------------------
Sp-Bem1         ------------------------------------------------------------
Cc-Bem1         ------------------------------------------------------------
Lb-Bem1         ------------------------------------------------------------
Pp-Bem1         QDADASVDGLLPGARVAMLHLDRPGVDPQFKLEAGSNIDRLIQLRREAAQTRDEVTARTS 1272
Pg-Bem1         ------------------------------------------------------------
Pb-Bem1A        ------------------------------------------------------------
Pb-Bem1B        ------------------------------------------------------------
Bd-Bem1         ------------------------------------------------------------

Sc-Bem1         ------------------------------------------------------------
Ca-Bem1         ------------------------------------------------------------
Mg-Bem1         ------------------------------------------------------------
Fg-Bem1         ------------------------------------------------------------
An-Bem1         ------------------------------------------------------------
Yl-Bem1         ------------------------------------------------------------
Sp-Bem1         ------------------------------------------------------------
Cc-Bem1         ------------------------------------------------------------
Lb-Bem1         ------------------------------------------------------------
Pp-Bem1         ALNDIKRTWTSMGGLAKDLTSVQDDIAAAAERQIWHQQVRQHDAPPTPESPVTHLPTPTI 1332
Pg-Bem1         ------------------------------------------------------------
Pb-Bem1A        ------------------------------------------------------------
```

```
Pb-Bem1B        ------------------------------------------------------------
Bd-Bem1         ------------------------------------------------------------

Sc-Bem1         ------------------------------------------------------------
Ca-Bem1         ------------------------------------------------------------
Mg-Bem1         ------------------------------------------------------------
Fg-Bem1         ------------------------------------------------------------
An-Bem1         ------------------------------------------------------------
Yl-Bem1         ------------------------------------------------------------
Sp-Bem1         ------------------------------------------------------------
Cc-Bem1         ------------------------------------------------------------
Lb-Bem1         ------------------------------------------------------------
Pp-Bem1         SPASVLATLDGLATRLEEKVSTPLSAVAPAMGESLNKYLTECSTALSLLLDHTRYLTTTW 1392
Pg-Bem1         ------------------------------------------------------------
Pb-Bem1A        ------------------------------------------------------------
Pb-Bem1B        ------------------------------------------------------------
Bd-Bem1         ------------------------------------------------------------

Sc-Bem1         ------------------------------------------------------------
Ca-Bem1         ------------------------------------------------------------
Mg-Bem1         ------------------------------------------------------------
Fg-Bem1         ------------------------------------------------------------
An-Bem1         ------------------------------------------------------------
Yl-Bem1         ------------------------------------------------------------
Sp-Bem1         ------------------------------------------------------------
Cc-Bem1         ------------------------------------------------------------
Lb-Bem1         ------------------------------------------------------------
Pp-Bem1         VSMQNQASTMGAVRDDFHGLQVQTEELKLRFDDAIEGVLSGTLTGDELLLTEGGLSSQLK 1452
Pg-Bem1         ------------------------------------------------------------
Pb-Bem1A        ------------------------------------------------------------
Pb-Bem1B        ------------------------------------------------------------
Bd-Bem1         ------------------------------------------------------------

Sc-Bem1         ------------------------------------------------------------
Ca-Bem1         ------------------------------------------------------------
Mg-Bem1         ------------------------------------------------------------
Fg-Bem1         ------------------------------------------------------------
An-Bem1         ------------------------------------------------------------
Yl-Bem1         ------------------------------------------------------------
Sp-Bem1         ------------------------------------------------------------
Cc-Bem1         ------------------------------------------------------------
Lb-Bem1         ------------------------------------------------------------
Pp-Bem1         HAEESVQSFIDDLPRRVTFVAPAHSEPHSSPFPKGPRLTSVEFNLAMVQHAAAAELPVDL 1512
Pg-Bem1         ------------------------------------------------------------
Pb-Bem1A        ------------------------------------------------------------
Pb-Bem1B        ------------------------------------------------------------
Bd-Bem1         ------------------------------------------------------------

Sc-Bem1         ------------------------------------------------------------
Ca-Bem1         ------------------------------------------------------------
Mg-Bem1         ------------------------------------------------------------
Fg-Bem1         ------------------------------------------------------------
An-Bem1         ------------------------------------------------------------
Yl-Bem1         ------------------------------------------------------------
Sp-Bem1         ------------------------------------------------------------
Cc-Bem1         ------------------------------------------------------------
Lb-Bem1         ------------------------------------------------------------
Pp-Bem1         SHLDQTVRSDSNAYSMMLSGALKLLEYKATHFQSAKAAKIVDAALASVVSKVRRVADVVT 1572
Pg-Bem1         ------------------------------------------------------------
Pb-Bem1A        ------------------------------------------------------------
```

```
Pb-Bem1B      ------------------------------------------------------------
Bd-Bem1       ------------------------------------------------------------

Sc-Bem1       ------------------------------------------------------------
Ca-Bem1       ------------------------------------------------------------
Mg-Bem1       ------------------------------------------------------------
Fg-Bem1       ------------------------------------------------------------
An-Bem1       ------------------------------------------------------------
Yl-Bem1       ------------------------------------------------------------
Sp-Bem1       ------------------------------------------------------------
Cc-Bem1       ------------------------------------------------------------
Lb-Bem1       ------------------------------------------------------------
Pp-Bem1       SVQDSMDRPDEQQNSERLGKLSAMVNEVLQVEVQEIARSFSPVRGLMHRLRTLPGVSEIT 1632
Pg-Bem1       ------------------------------------------------------------
Pb-Bem1A      ------------------------------------------------------------
Pb-Bem1B      ------------------------------------------------------------
Bd-Bem1       ------------------------------------------------------------

Sc-Bem1       ------------------------------------------------------------
Ca-Bem1       ------------------------------------------------------------
Mg-Bem1       ------------------------------------------------------------
Fg-Bem1       ------------------------------------------------------------
An-Bem1       ------------------------------------------------------------
Yl-Bem1       ------------------------------------------------------------
Sp-Bem1       ------------------------------------------------------------
Cc-Bem1       ------------------------------------------------------------
Lb-Bem1       ------------------------------------------------------------
Pp-Bem1       AADGIVASRQRALEDAEAQFATWKENAATLHEQIILALQAERARQIERLRLQEEREQAAA 1693
Pg-Bem1       ------------------------------------------------------------
Pb-Bem1A      ------------------------------------------------------------
Pb-Bem1B      ------------------------------------------------------------
Bd-Bem1       ------------------------------------------------------------

Sc-Bem1       ------------------------------------------------------------
Ca-Bem1       ------------------------------------------------------------
Mg-Bem1       ------------------------------------------------------------
Fg-Bem1       ------------------------------------------------------------
An-Bem1       ------------------------------------------------------------
Yl-Bem1       ------------------------------------------------------------
Sp-Bem1       ------------------------------------------------------------
Cc-Bem1       ------------------------------------------------------------
Lb-Bem1       ------------------------------------------------------------
Pp-Bem1       EERARLAREEAETLAKAELDRIEQERRETEERARREQEELEERERLEAENRAKEERSIPQ 1752
Pg-Bem1       ------------------------------------------------------------
Pb-Bem1A      ------------------------------------------------------------
Pb-Bem1B      ------------------------------------------------------------
Bd-Bem1       ------------------------------------------------------------

Sc-Bem1       ------------------------------------------------------------
Ca-Bem1       ------------------------------------------------------------
Mg-Bem1       ------------------------------------------------------------
Fg-Bem1       ------------------------------------------------------------
An-Bem1       ------------------------------------------------------------
Yl-Bem1       ------------------------------------------------------------
Sp-Bem1       ------------------------------------------------------------
Cc-Bem1       ------------------------------------------------------------
Lb-Bem1       ------------------------------------------------------------
Pp-Bem1       GIGLILGSSELLEPVQETDEHSMTREIYGPEAKDDLFGLQVLPVTADPLSQHTSILRPQI 1812
Pg-Bem1       ------------------------------------------------------------
Pb-Bem1A      ------------------------------------------------------------
```

```
Pb-Bem1B        ------------------------------------------------------------
Bd-Bem1         ------------------------------------------------------------

Sc-Bem1         ------------------------------------------------------------
Ca-Bem1         ------------------------------------------------------------
Mg-Bem1         ------------------------------------------------------------
Fg-Bem1         ------------------------------------------------------------
An-Bem1         ------------------------------------------------------------
Yl-Bem1         ------------------------------------------------------------
Sp-Bem1         ------------------------------------------------------------
Cc-Bem1         ------------------------------------------------------------
Lb-Bem1         ------------------------------------------------------------
Pp-Bem1         LALRKRLRSLHINDVVRPSQKSDLSLPEDEYRKRMDKELLAIVSGVDVLPPSIIDDPLTN 1872
Pg-Bem1         ------------------------------------------------------------
Pb-Bem1A        ------------------------------------------------------------
Pb-Bem1B        ------------------------------------------------------------
Bd-Bem1         ------------------------------------------------------------

Sc-Bem1         ------------------------------------------------------------
Ca-Bem1         ------------------------------------------------------------
Mg-Bem1         ------------------------------------------------------------
Fg-Bem1         ------------------------------------------------------------
An-Bem1         ------------------------------------------------------------
Yl-Bem1         ------------------------------------------------------------
Sp-Bem1         ------------------------------------------------------------
Cc-Bem1         ------------------------------------------------------------
Lb-Bem1         ------------------------------------------------------------
Pp-Bem1         AELHSLRTEVKASVDLMPTVHKLADLAIALRNCDDALSDLLEHIDSYPSAPLGMLSSSHT 1932
Pg-Bem1         ------------------------------------------------------------
Pb-Bem1A        ------------------------------------------------------------
Pb-Bem1B        ------------------------------------------------------------
Bd-Bem1         ------------------------------------------------------------

Sc-Bem1         ------------------------------------------------------------
Ca-Bem1         ------------------------------------------------------------
Mg-Bem1         ------------------------------------------------------------
Fg-Bem1         ------------------------------------------------------------
An-Bem1         ------------------------------------------------------------
Yl-Bem1         ------------------------------------------------------------
Sp-Bem1         ------------------------------------------------------------
Cc-Bem1         ------------------------------------------------------------
Lb-Bem1         ------------------------------------------------------------
Pp-Bem1         SDPTLPPESQLSARIAFTKSLVDTMTTQYSAVSGDPRAVSEHDRIVQTWMELEAMASDRV 1992
Pg-Bem1         ------------------------------------------------------------
Pb-Bem1A        ------------------------------------------------------------
Pb-Bem1B        ------------------------------------------------------------
Bd-Bem1         ------------------------------------------------------------

Sc-Bem1         ------------------------------------------------------------
Ca-Bem1         ------------------------------------------------------------
Mg-Bem1         ------------------------------------------------------------
Fg-Bem1         ------------------------------------------------------------
An-Bem1         ------------------------------------------------------------
Yl-Bem1         ------------------------------------------------------------
Sp-Bem1         ------------------------------------------------------------
Cc-Bem1         ------------------------------------------------------------
Lb-Bem1         ------------------------------------------------------------
Pp-Bem1         NGTKSRPNSVMSSGRSSRASAVSTAAKAAPTKKAAGYSRLSVGGSDARYLAPPPPNRRTT 2052
Pg-Bem1         ------------------------------------------------------------
Pb-Bem1A        ------------------------------------------------------------
```

```
Pb-Bem1B      ------------------------------------------------------------
Bd-Bem1       ------------------------------------------------------------

Sc-Bem1       ------------------------------------------------------------
Ca-Bem1       ------------------------------------------------------------
Mg-Bem1       ------------------------------------------------------------
Fg-Bem1       ------------------------------------------------------------
An-Bem1       ------------------------------------------------------------
Yl-Bem1       ------------------------------------------------------------
Sp-Bem1       ------------------------------------------------------------
Cc-Bem1       ------------------------------------------------------------
Lb-Bem1       ------------------------------------------------------------
Pp-Bem1       SGSSTGTRPRSTSRVSSISSNRSVSGPTPNVSSATLHGSTFASRQRTTSVTSGAPMKTPL 2112
Pg-Bem1       ------------------------------------------------------------
Pb-Bem1A      ------------------------------------------------------------
Pb-Bem1B      ------------------------------------------------------------
Bd-Bem1       ------------------------------------------------------------

Sc-Bem1       ------------------------------------------------------------
Ca-Bem1       ------------------------------------------------------------
Mg-Bem1       ------------------------------------------------------------
Fg-Bem1       ------------------------------------------------------------
An-Bem1       ------------------------------------------------------------
Yl-Bem1       ------------------------------------------------------------
Sp-Bem1       ------------------------------------------------------------
Cc-Bem1       ------------------------------------------------------------
Lb-Bem1       ------------------------------------------------------------
Pp-Bem1       KQPHLASSKASSRPRSNTNTTPRTASPAFSETSSRSISRSGLNASVSSVARSSWSRAPRQ 2172
Pg-Bem1       ------------------------------------------------------------
Pb-Bem1A      ------------------------------------------------------------
Pb-Bem1B      ------------------------------------------------------------
Bd-Bem1       ------------------------------------------------------------

Sc-Bem1       ------------------------------------------------------------
Ca-Bem1       ------------------------------------------------------------
Mg-Bem1       ------------------------------------------------------------
Fg-Bem1       ------------------------------------------------------------
An-Bem1       ------------------------------------------------------------
Yl-Bem1       ------------------------------------------------------------
Sp-Bem1       ------------------------------------------------------------
Cc-Bem1       ------------------------------------------------------------
Lb-Bem1       ------------------------------------------------------------
Pp-Bem1       SFPALLRSPPKNKAVPTAKKPYIANPKNKLDVAVGDVINSLPVNINVEVVADTWKDQSGK 2232
Pg-Bem1       ------------------------------------------------------------
Pb-Bem1A      ------------------------------------------------------------
Pb-Bem1B      ------------------------------------------------------------
Bd-Bem1       ------------------------------------------------------------

Sc-Bem1       ------------------------------------------------------------
Ca-Bem1       ------------------------------------------------------------
Mg-Bem1       ------------------------------------------------------------
Fg-Bem1       ------------------------------------------------------------
An-Bem1       ------------------------------------------------------------
Yl-Bem1       ------------------------------------------------------------
Sp-Bem1       ------------------------------------------------------------
Cc-Bem1       ------------------------------------------------------------
Lb-Bem1       ------------------------------------------------------------
Pp-Bem1       YWIGDQDPKLCFCRILRSQTVMVRVGGGWSELSRFIKDHFADAFRILPESPRLGSREEKW 2292
Pg-Bem1       ------------------------------------------------------------
Pb-Bem1A      ------------------------------------------------------------
```

```
Pb-Bem1B     ------------------------------------------------------------
Bd-Bem1      ------------------------------------------------------------

Sc-Bem1      ------------------------------------------------------------
Ca-Bem1      ------------------------------------------------------------
Mg-Bem1      ------------------------------------------------------------
Fg-Bem1      ------------------------------------------------------------
An-Bem1      ------------------------------------------------------------
Yl-Bem1      ------------------------------------------------------------
Sp-Bem1      ------------------------------------------------------------
Cc-Bem1      ------------------------------------------------------------
Lb-Bem1      ------------------------------------------------------------
Pp-Bem1      ISSATLSQAAESIALSNPKTPEVVSSHLPSFALSTPSGTSPKSIKTVSSPGSPLTPLQFL 2352
Pg-Bem1      ------------------------------------------------------------
Pb-Bem1A     ------------------------------------------------------------
Pb-Bem1B     ------------------------------------------------------------
Bd-Bem1      ------------------------------------------------------------

Sc-Bem1      ----------------------------------
Ca-Bem1      ----------------------------------
Mg-Bem1      ----------------------------------
Fg-Bem1      ----------------------------------
An-Bem1      ----------------------------------
Yl-Bem1      ----------------------------------
Sp-Bem1      ----------------------------------
Cc-Bem1      ----------------------------------
Lb-Bem1      ----------------------------------
Pp-Bem1      RRADRESPVPRAETPTRPPRNTAAKPARPPVWRP 2407
Pg-Bem1      ----------------------------------
Pb-Bem1A     ----------------------------------
Pb-Bem1B     ----------------------------------
Bd-Bem1      ----------------------------------
```

Alignment of fungal Cdc24 proteins

```
Mg-Cdc24     MNQASRRQPGLNAPRDTKLAGSVQSNDVNQQPLLPTPAPKPPDQSLPGPSRRSASENIGV 60
Fg-Cdc24     ------------------------------------------------------------
An-Cdc24     ------------------------------------------------------------
Sp-Cdc24     ------------------------------------------------------------
Pb-Cdc24     ------------------------------------------------------------
Cc-Cdc24     ------------------------------------------------------------
Lb-Cdc24     ------------------------------------------------------------
Pp-Cdc24     ------------------------------------------------------------
Yl-Cdc24     ------------------------------------------------------------
Pg-Cdc24     ------------------------------------------------------------
Ca-Cdc24     ----------------------------------------------------MEHPPAAL 8
Sc-Cdc24     ------------------------------------------------------------

Mg-Cdc24     GHPTCPVAFTPPNALAMAYAPLLRTNTTPIFPSSTGGVARLQGTMGSTGPAVGIPPLRAS 120
Fg-Cdc24     ----------------MNHAP------------------------------------RAS 8
An-Cdc24     ------------------------------------------------------------
Sp-Cdc24     --------------------------------------------------MAYFQDRKTS 10
Pb-Cdc24     ------------------------------------------------------------
Cc-Cdc24     ------------------------------------------------------------
Lb-Cdc24     ------------------------------------------------------------
Pp-Cdc24     ------------------------------------------------------------
```

```
Yl-Cdc24        -MNGSSRRLSPPNDNLAHQQMLHQEINNLLPSSPYTKTPQQRKSSSQSTPSLSTLSPSTQ 59
Pg-Cdc24        ---------------------------------MTTNPPPTPSTSSFNYQNPFSSSTRSS 27
Ca-Cdc24        RTFSTQSTSSLNSVSTVSSSRIVSSGPVNINNFNKPSTPKDHLFYRCESLKRKLQKIPGM 68
Sc-Cdc24        MAIQTRFASGTSLSDLKPKPSATSISIPMQNVMNKPVTEQDSLFHICANIRKRLEVLPQL 60

Mg-Cdc24        QLSGTTLHN-STTSLSSLASASTVVPAQNGGQVVATSNIINQKADASRSLYQICMSLK-- 177
Fg-Cdc24        QLSGSTAYTGSSASLSSLATATTITPT-NGGPVHATANIINQKADASRSLYQICISLK-- 65
An-Cdc24        -----------------MADTAGLNPG----GPVAEDNIINRRGNEG--IYQSCVNLK-- 35
Sp-Cdc24        SRSLPSYINHSTQNLVGPRKDETNLSEYMKLRLLQSPSQVIYNLENTVSLYRRCLNLR-- 68
Pb-Cdc24        ----------------------------MPSCLIESPQKPIQ----GQSLYHTCLSVL-- 26
Cc-Cdc24        ----------MASQAAARKKSIISSQNLQIETPVAGNTLLNKSASQATSLYQQCASLR-- 48
Lb-Cdc24        -----------MATVAGRKKSVISTAGLTIDTPVATNGLLNKAASQSTSLYQQCSALR-- 47
Pp-Cdc24        -----------MASAAGRKKSVISATTPTIESPIANNTLLNKAASQSTSLYQQCSALR-- 47
Yl-Cdc24        NSTYPSTPRLRSMTPTMSMSSVQSTAPTSVAPMPVANLVMNRQADSEKSLYNMCKTMR-- 117
Pg-Cdc24        TTTTHTMTTTTTSASSHTSLNQHQSANLSIDMPIATSSVVNKTANRSASLYQNCLETR-- 85
Ca-Cdc24        EPFLNQAFNQAEQLSEQQALALAQERSNGNGHSNGKRHQSLDGAMNRLSVGSDSSSIQGS 128
Sc-Cdc24        KPFLQLAYQSSEVLSERQSLLLSQKQHQELLKSNGANRDSSDLAPTLRSSSISTATSLMS 120

Mg-Cdc24        -QRLAQVPGFEEYMSLMDQW----------EAEDPEGGIVESVWKLLRMGTPLIVIFNLL 226
Fg-Cdc24        -QRLAQVPGFGPYLDELDPT----------DPVDP-------LWNLFRSGYPLLLIYNAL 107
An-Cdc24        -KRLAEVPGFEPYLREMEEE----------DLAQGNSDPVASLWNYLRHGYPLLAIYNAS 84
Sp-Cdc24        -KRLMDISELAAFFDSIHREALNSSFKILEFKDIEFDDPVTEIWLFCRLGYPLCALFNCL 127
Pb-Cdc24        -DRLALVNGFQQFLDSP-----------VQKTQLLNTDPVTKLWNLCQLGTPLYTLFNAL 74
Cc-Cdc24        -SRLMRIRGFSYYFNLAAES----------TDDRQSTDPVTQLWDIFSLGISLCYIFDQL 97
Lb-Cdc24        -SRLMLIRGFPHYFNLATP-----------PDSRQSTDPVTQLWDLFALGVPLCYIFDLL 95
Pp-Cdc24        -TRLLQVQDFPEWFTVSSPP----------DSSRRSTDPVTQLWDCFALGVPLCYLFNLL 96
Yl-Cdc24        -DRFHDIEQVRPFLEDSEMSSIVP---ITHLPPSIPLDPVTQLWSLFRLGAPLCILFNML 173
Pg-Cdc24        -ALLRRVPGFEAGPWITETK------------NPNSLLSVTQTLEIFRLGSPLCFLYNAI 132
Ca-Cdc24        LTRMATNASTSSLISGMPNNNTLFTFTAGVLPANISVDPATHLWKLFQQGAPFCVLINHI 188
Sc-Cdc24        MEGISYTNSNPSATPNMEDTLLTFS--MGILPITMDCDPVTQLSQLFQQGAPLCILFNSV 178
                                                                 *

Mg-Cdc24        K---------------------PENPMSFDPTP---DAKKAKMYIYKFAEQCKN-----E 257
Fg-Cdc24        R---------------------PNEELKVDDSSSANEAKKSKIAIFKFVQACMK-----E 141
An-Cdc24        D---------------------PGAPLEIDTSKVP-EARRPKAATFKFLQAAIQ-----E 117
Sp-Cdc24        P---------------------VKQKLEVNSSVSLENTNVCKASLYRFMLMCKN-----E 161
Pb-Cdc24        D---------------------PKEPLTLEYNHNLNQANNAKAIVYHFLVSCRK-----E 108
Cc-Cdc24        --D--------------QNFNKINNSQFNPEKYEANPDKERKHAIALFAMQVRNNQ--VM 139
Lb-Cdc24        PAE--------------AGFNKINNSSFTAD-YDANPDRAQKRAIALFAMQIRTDE--VT 138
Pp-Cdc24        P----------------APFSPIN-IDTDPKSFDATNEKTKKRSIALFSMQIK------- 132
Yl-Cdc24        QP-----------------RTPLTVDPHAAANRASIDVRTCKRSVYDFVQGCKA-----E 211
Pg-Cdc24        LNPPLSPPELANLPPLPPGKTWQNLKPLKVLWCPREDLKGCQKSAAHFIMALKTELGWGT 192
Ca-Cdc24        LP------------------------DSQIPVVSSDDLRICKKSVYDFLIAVKT-----Q 219
Sc-Cdc24        KP------------------------QFKLPVIASDDLKVCKKSIYDFILGCKK-----H 209
                                                               *

Mg-Cdc24        LGIED--IFTISDLLKN-DTTGFVKVTTVVNQVLDIAQTRGMLIQQQPYPEDDMPDSGKS 314
Fg-Cdc24        LEIPSTQSFVITDLMGT-DTSGFVKVTQVVNYVLDRAEERGYLMQAQPDVESNEPTGG-- 198
An-Cdc24        MAFPQQECFLITDLYGE-NTIGFTKVIKMVNRVLDILEIQGQLKKP-SDTAMAAPAAG-R 174
Sp-Cdc24        LGLTDAALFSISEIYKP-STAPLVRALQTIELLLKKYEVSNTTKSSSTPSPSTDDNVPTG 220
Pb-Cdc24        LDFAEEDLFGITDIFLD-DTNGFVKVVNTITKILQILQDR-GVISAKSPNRNSDPNAPKD 166
Cc-Cdc24        QAIPGLEPFAITDLWDRKSTDGLVKVINSVTAIVEYLPEDVFEQAPTSPPTLS-AHESSD 198
Lb-Cdc24        QKIPGCELFTVTDLWDRKLTDGLVKVINTVTAIVDHLQPDVFEESPPSPPTLS-SHDSSD 197
Pp-Cdc24        -QLEGCEQFTVTELWDRNSTDGFVKVVNNVINLVRCLPDEVFVEPQLSSPHLASAQQSTD 191
Yl-Cdc24        LGYSDDELFTISNLFSE-NTGDLIKVMRTVSMVVETLEARHLLPPKTAPITAPATRDAR- 269
Pg-Cdc24        EIDEIQENGLMVNMLYDVNTNATVKVISNVLRLLHLLKALGVLLPPSEDELVPHSNELNV 252
Ca-Cdc24        LNFDDENMFTISNVFSD-NAQDLIKIIDVINKLLAEYSDASDSGGGDEDVNMDVQITDER 278
Sc-Cdc24        FAFNDEELFTISDVFAN-STSQLVKVLEVVETLMNSSPTIFPSKSKTQQIMNAENQHRHQ 268

Mg-Cdc24        KMSYR-----------------DYIVRELVDTERKYVQDLENLHD--------------- 342
```

```
Fg-Cdc24        QMTYR-----------------DHIIRELVDTERKYVQDLENLHD--------------- 226
An-Cdc24        KLTKR-----------------EHILKELLETERDYVHHLQNLQA--------------- 202
Sp-Cdc24        TLNSLIASG-------------RRVTAELYETELKYIQDLEYLSN--------------- 252
Pb-Cdc24        TR--------------------DKIVLELLETERKYVQDMELLQASTKEHKIYIPYNILT 206
Cc-Cdc24        SLHHDSLPAGPAPPTNAREARRIHITREILETERKYVEGLEIMQ---------------- 242
Lb-Cdc24        SLL-DALPQ-----PNGKEAARNNIVREMVETERKFVQDLEIMQVRVFVPLMSLALPIQP 251
Pp-Cdc24        SLDADGAAP----PPERDGGARYNIVRELVETERKYVQDLEVMQ---------------- 231
Yl-Cdc24        ----------------------YNVVQELLQTERKYVQDLEILHD--------------- 292
Pg-Cdc24        AMDDR-----------------ARVVKEILESERKYVQDLEVLQN--------------- 280
Ca-Cdc24        S----------------------KVFREIIETERKYVQDLELMCK--------------- 301
Sc-Cdc24        PQQSSKKHN-----------EYVKIIKEFVATERKYVHDLEILDK--------------- 302
                                           *    *

Mg-Cdc24        ---------LKKALTERGIVTGDVAYQIFGNIDAILDFQRRFLIRVETTNSMPGEVQQWG 393
Fg-Cdc24        ---------LKKTLEQQGEIPGDTLHQIFFNINAILDFQRRFLIRVETTNSMPALNQRWG 277
An-Cdc24        ---------LKKELEDTGALTGDASHQIFLNLNNLLDFSQRFLIRLEQHYARPEEQQNWG 253
Sp-Cdc24        ---------YMVILQQKQILSQDTILSIFTNLNEILDFQRRFLVGLEMNLSLPVEEQRLG 303
Pb-Cdc24        KFFYVFIQSYMRELQIQKILSPDTVHYLFANLNALVDFQRRFLIQLEDIAEKPAQDQQLG 266
Cc-Cdc24        --------KYATQLAQQNIIDQDTIHLLFPNLNNLLNFQRKFLIRFESTLEQPWSEQRWG 294
Lb-Cdc24        A---GYVQKYSNALSQANLMDQDTIHLLFPNLNKLLNFQRKFLIRFESTAELPWQDQRWG 308
Pp-Cdc24        --------KYSIAASQMNVMDQETLQRLFPGLNKLLNFQRKFLIKLESIAELSPNDQSWG 283
Yl-Cdc24        ---------YQNRLLEAEILNPDTIHLMFLNVSALVDFQRRVLVGIEHVCSLPVEEQRFG 343
Pg-Cdc24        ---------YQRALLQREILPSDQIRALFINLDSLVDFQRRFLIGVEANARLPPDEQRFG 331
Ca-Cdc24        ---------YRQDLIEAENLSSEQIHLLFPNLNEIIDFQRRFLNGLECNINVPIRYQRIG 352
Sc-Cdc24        ---------YRQQLLDSNLITSEELYMLFPNLGDAIDFQRRFLISLEINALVEPSKQRIG 353
                                            *        *    *   *         *  *

Mg-Cdc24        SPFVAMEASFDPIYTPFIANQRKAALIAQSEFDKIKSIE-----HPVACDFNTLDGFLLK 448
Fg-Cdc24        APFVHYEDALIDIYQPFIANQRKAAQVANQVFDKIQRSS-----HPVAADYNTLDGFLLK 332
An-Cdc24        ELFIQHEEAFR-QYEPFIANQMRCDKTCQKEWDKIQAAPRSPDLQQMVAQPATLNGFFVK 312
Sp-Cdc24        ALFIALEEGFS-VYQVFCTNFPNAQQLIIDNQNQLLKVA------NLLEPSYELPALLIK 356
Pb-Cdc24        LLFVQTEDAFI-VYEPYCSNYYAAQDLVKEKAAELQKLD------HVLSPTHQLLSMLIK 319
Cc-Cdc24        QHFVDCEDEFA-VYEPYCSNFTNATELLVANEQNLAALN------GFISVS-ELKAFHIT 346
Lb-Cdc24        QHFLESEDEFV-VYEPYCANYTNATELMLANEESLVPLN------HLINVKGELPAFLIK 361
Pp-Cdc24        VPFMENEEEFA-VYEPYCALYTSASDIMLQEMQNLTALD------NVLSKS-ELPAFLIK 335
Yl-Cdc24        ALFLNMEAGFH-IYEPYALNQKASSDLAVQEAPKLTALA------HIIEPTYELQAFLIK 396
Pg-Cdc24        HLFHSFEDSFS-CYEPFCANFASASQLAQDENAALTQLVK-------------------- 370
Ca-Cdc24        SVFIHASLGPFNAYEPWTIGQLTAIDLINKEAANLKKSS------SLLDPGFELQSYILK 406
Sc-Cdc24        ALFMHSKHFFK-LYEPWSIGQNAAIEFLSSTLHKMRVDESQ---RFIINNKLELQSFLYK 409¥
                  *          *

Mg-Cdc24        PMQRLVKYPLLLKDLLK-----------KSEDDKVKDDLSLGIAAAERTLQKANEAVDRD 497
Fg-Cdc24        PMQRLVKYPLLLKDLNK-----------KTEDDEVKMDLTTGCEAAERVLSKANEAVNRD 381
An-Cdc24        PFQRLTKYPLMLSELRK-----------QIEDPDLQADISRAIDSIQSVLDAANDAIDKE 361
Sp-Cdc24        PIQRICKYPLLLNQLLK----------GTPSGYQYEEELKQGMACVVRVANQVNETRRIH 406
Pb-Cdc24        PIQRICKYPLLMSSLLK----------STNPSWQSYPQLIQGSEAIRRVAEKVNETQRKQ 369
Cc-Cdc24        PIQRVCKYPLFFGDLIKV--------ATGDDTYPHLEGLKQGAEAAHRITAKINEAHRRA 398
Lb-Cdc24        PIQRVCKYPLLLD-------------ASSPTTYQHFDELKRGSDAAKRITDKINEAQRRA 408
Pp-Cdc24        PVQRICKYPLLLESLLK---------AVKTTDYPFIEELKLGVAAAKRVTDKINEAQRRA 386
Yl-Cdc24        PIQRICKYPLLLRELVK----------VSSSDHPYASELLDGLEAIKRVTNSVNETQRRV 446
Pg-Cdc24        ---------------------------HTSPNGAYYDELKEGLASITRVTDAVNETTRQR 403
Ca-Cdc24        PIQRLCKYPLLLKELIKTSPEYSKQDPHGSSSSTSFNELLVAKTAMKELANQVNEAQRRA 466
Sc-Cdc24        PVQRLCRYPLLVKELLAE-----------SSDDNNTKELEAALDISKNIARSINENQRRT 458
                                                                     *

Mg-Cdc24        LLDDALEDLKTRVDDWKNHQVDQFGSLLLHGVHTVLTG--KSESERDYEIYLFECILLCC 555
Fg-Cdc24        LLDEAVEDLTSRVEDWKSHKVEQFGKLLLHGVYGVITG--KTDQEKDYEIYLFESILLCC 439
An-Cdc24        QLAAAFVELDERVDDWKALKIETFGELLRFGTFTVIKNDNNKDSEREYHIYLFERILLCC 421
Sp-Cdc24        ENRNAIIELEQRVIDWKGYSLQYFGQLLVWDVVNVCKA----DIEREYHVYLFEKILLCC 462
Pb-Cdc24        ENLQLVQDLKKRMDDWKGPPIDSHGALLLQEKLMVTVN----DLDREHLILLFEKAILIC 425
Cc-Cdc24        DNEQIVKSLAKRITDWKGHHLENFGELLLEDVFVVTKS----DIDREYHVFLFEKIILCC 454
Lb-Cdc24        ENEQTVKSLHYRIDDWKGHHLENFGELLLDDIFVVTKS----DIDREYHVFLFEKIILCC 464
```

```
Pp-Cdc24        ENLATVEALKGRVEDWKGHHISQFGSLLLDDIFTVTKS----EVDREYHVFLFEKIILCC 442
Yl-Cdc24        ENVAIIQDLCERIDSWKSYNLEDFGDLLYMDHLTIAKE----SGEYEYLVYLFEAIILCC 502
Pg-Cdc24        ENELAVNDLKERVEDWKGHELVTFGQLVLEDTFTVVKN----ESEREYHVYLFERIILCC 459
Ca-Cdc24        ENIEHLEKLKERVGNWRGFNLDAQGELLFHGQVGVKDA----ENEKEYVAYLFEKIVFFF 522
Sc-Cdc24        ENHQVVKKLYGRVVNWKGYRISKFGELLYFDKVFISTTNSSSEPEREFEVYLFEKIIILF 518
                        *  *   *        * *                        ***

Mg-Cdc24        KEISSTKSKDKKDKTKS----------------------TGPKIRNKGAKLQLKGRIFMT 593
Fg-Cdc24        KEISSSKSKDKKDKLRS----------------------GGPKARNKSAKLQLKGRIFMT 477
An-Cdc24        KDINPNKQKSRLVGGSK----------------------DKPNTSKGKPRLVLKGRIYMA 459
Sp-Cdc24        KEMS-TLKRQARSISMN-------------------------KKTKRLDSLQLKGRILTS 496
Pb-Cdc24        KELKEGSKNRLTKTNTI-------------------------MKKKRRASIQAKGTISVN 460
Cc-Cdc24        KEALQQPPNGRGKNAKNNSILKKQ--P-----APLPLATGGQSIAQRDTPLLLKGRIFLG 507
Lb-Cdc24        KEAPLLPQNGRNKVGKNNSILKKQGTP-----PPLTLPGGVGQPQKKTTPLLLKGRIFLG 519
Pp-Cdc24        KEYLVPSANGR-KVGKSNSILKKPPVP-----PPLMLP-GAGPSKKRNTPLLLKGRIFLA 495
Yl-Cdc24        KETRDKKGKTMSLPSKK----------------------------PQKTVLSLKGRIYIA 534
Pg-Cdc24        KEVGVKLDKKASKQSMSLKKNSQIN-------------NSNVNGGKRKNTQQLKGRIFIA 506
Ca-Cdc24        TEIDDNKKSDKQEKKSKFSTRKRSTSSNLSSSTTNLLESINNSRKDNTLPLELKGRVYIS 582
Sc-Cdc24        SEVVTKKSASSLILKKK----------------------SSTSASISASNITDNNGSPHH 556


Mg-Cdc24        NVTEVLSFAKPGS----------------------YTVQIWWKGDPG-VENFIIKFQNEE 630
Fg-Cdc24        NVTDIVSFTKPGS----------------------HSVQIWWKGDPG-VENFIIKFLNEE 514
An-Cdc24        NVTDIVWLQKPGS----------------------YRIQIFWKGDPGVVDNFIIRYQNED 497
Sp-Cdc24        NITTVVPNHHMGS----------------------YAIQIFWRGDPQ-HESFILKLRNEE 533
Pb-Cdc24        RITHILN-KSTAS----------------------GGKYYSSSNSNS-VQQFTLKFRNEE 496
Cc-Cdc24        NVTQALSVPARAST--------------ASGIPAHFPLQVWWKGDDD-LEFFTLRCKRED 552
Lb-Cdc24        NVTQAVPVSARIST--------------PSGVPPHFPLAVWWKGDDD-LEFFTLRCRREE 564
Pp-Cdc24        NVHLAEPKIS----------------------AGQYSLAVYWRGDDD-TEFFTLRCRNEE 532
Yl-Cdc24        NVTNMISSTENG-----------------------YSLTLTYKGDGY--GSMTMRFRHEE 569
Pg-Cdc24        NVMSATPRPVPPEEQLKKWQAAITKAVDDAVERRRRQVHHLSSGSRSKMNSPLSQFPNTP 566
Ca-Cdc24        EIYNISAPNTPGS-----------------------TLIISWSGRKE-SGSFTLRYRSEE 618
Sc-Cdc24        SYHKRHSNSSSSN-----------------------NIHLSSSSAAAIIHSSTNSSDNNS 593


Mg-Cdc24        MMKKWAAGLDQQRKASG-SQSATS------PDQPTAEFAWMRSQSA-VENPYAQTQPEEE 682
Fg-Cdc24        TLKKWVNTLETQRKHNV-PRQSTN------SDTLSTDFAWTRDQVAGLENPYLKENDDED 567
An-Cdc24        TMRKWYKDINTQREIQAEQRSARN------TGTSDSEFTYMKSLSN-IPNPYQQEYDVEE 550
Sp-Cdc24        SHKLWMSVLNRLLWKNEHGSPKDI---------RSAASTPANPVYNRSSSQTSKGYNSSD 584
Pb-Cdc24        QLKQWEAVIDKTRMNIQS----------------TSSSTPLLPTPSQSTS---------- 530
Cc-Cdc24        QMRQWESTINRLIKEAAQRRASER-----PHGP-ISRVAHHSPIVRVPPTDP-HSHFSSQ 605
Lb-Cdc24        QMRQWEAQINRLIKEAAQRKASDR-----PTMSGIARIAMTNSINPSSASRFGNSSTYSN 619
Pp-Cdc24        QLKMWETQLNRLIQENINRRMSDRNPQRLTQMTPSSAAAMQQRPPPVAYNQEKTYSTFSQ 592
Yl-Cdc24        QLRQWDATIKQLHDWYNQDSYESH-----------FSPLVESPISSRNSQQSSHLSEDLD 618
Pg-Cdc24        QSEMGQPPNSASYQHHPVSHPPRIP---EHPYAHSGHGNGGGSISSRTFEDEEESYQTDA 623
Ca-Cdc24        ARNQWEKCLRDLKTNEMNKQIHKKLRDSDSSFNTDDSAIYDYTGISTSPVNQSTQQQYYD 678
Sc-Cdc24        NNSSSSSLFKLSANEPKLDLRGRIMIMNLNQIIPQNNRSLNITWESIKEQGNFLLKFKNE 653


Mg-Cdc24        EE-YEFGAIPQQSQQYQAMAGTMPRMNSTASIRGRSQTGESSQSIASIARGPPPRFPLPQ 741
Fg-Cdc24        DEDIGPATAPAGFPGVTHPMSLGPRTASSNNLRARAGTGESSASLAGMVRAPPPRFPLPA 627
An-Cdc24        QS-TKEAAFFSEFP--------MSRNASSTSLRTRSATGGSGSSGPPLSTSRP-RYP-AM 599
Sp-Cdc24        YDLLRTHSLDENVNSPTSISSPSSKSSPFTKTTSKDTKSAT------------------- 625
Pb-Cdc24        -----------------------------DKTSFIDSLDDD------------------- 542
Cc-Cdc24        GSVVSSSAYSS-QASSAVSARSSSRSTAPYHYESSHTSYSS---SGPQGYPPHEGFDRD- 660
Lb-Cdc24        GAPVSIHHQPS-ISSSIRSNRPVN-SYNHDDASSNAGSMYS---AGPQGYPPHDGFDFEP 674
Pp-Cdc24        STVYSAYGATSPYGAPGRMGRHPYAAAGDEPPASANGSYSNGFGHGPQGYPPHDGFDVD- 651
Yl-Cdc24        RLTLGNRSLSGPVPKLGPRQHSTTSINGMHGANGMNHSSVS------------------- 659
Pg-Cdc24        ESSVNGRNTPSGSRRTTGHRSMPAERSEASFGPGRARAQTDDAASANLAQWRNQMPNSAI 683
Ca-Cdc24        HRGSHSSRHHSSSSTLSMMKNNRVKSGDLSRISSTSTTLDS------------------- 719
Sc-Cdc24        ETRDNWSSCLQQLIHDLKNEQFKARHHSSTSTTSSTAKSSS--------------MMSPT 699


Mg-Cdc24        PPQPLSVQTQLQGG-NSPGFRPGDSYFSPIGESPVSSRTSTASNVFPGSAGYGSFKGVTG 800
```

```
Fg-Cdc24        PPGSLSLQTQPNGA-HSPSAWAGDSYFSPVTESPASTRTSTTSGMFS-TPQYGFPKSAT- 684
An-Cdc24        PDSTLSVHTQFPGGSMSPGERNGNSYFSPT----ESARSSSQS------AGYPYNRQVT- 648
Sp-Cdc24        TTDERPSDFIRLNSE---ESVGTSSLRT--------SQTTSTIVSNDSSSTASIPSQISR 674
Pb-Cdc24        DDDESTKKFIRPIAS---SSSRLSELAN--------IPLSLLLSSTSTSTSTSTSSASS- 590
Cc-Cdc24        DEEDDLEDYPPASTS---YSGRGTPMGN-----RRPTALSMPGERSESLHGYERPRAQTE 712
Lb-Cdc24        EDEEDYEDYPPVSAANYPASGRGTPMGGPPGARRNPNALSMPLER-EPVAGYDRPRAQTE 733
Pp-Cdc24        -LDEDYEEYPLPNYP---PSGRATPAGS-----RRSDMIGFSQSARTSSHSS-IPSAMSS 701
Yl-Cdc24        IASSNGGGMSRARSASTPGFDDGSMRPS-------------------------------- 687
Pg-Cdc24        PPVPRSGQLTPASSDAYLPNSLRSSTSSKPLRHQPSHEWGAGTINGSNFYRVDDRSHLPT 743
Ca-Cdc24        -FSNNLNGSPNTTNPSLMSSDATKTIPT-------------------------------- 746
Sc-Cdc24        TTMNTPNHHNSRQTHDSMASFSSSHMKRVSDVLPKRRTTSSSFESEIKSISENFKNSIPE 759

Mg-Cdc24        QPYPGMWDGPIVDQNRYTAPAMPRAPSRDGPSPVNAYGMSGANGRNPRGPSMPVMASHSA 860
Fg-Cdc24        -PNPQQWED--ANGNRYTAPAMPRAPSRDGPSPN--------PARNPRGPSLPAMSSSSQ 733
An-Cdc24        --PVTPWGD---DNNRYTAPALSRATSRDGSNSGYFNG-APPNGRSAQRPSLPPMSGSNQ 702
Sp-Cdc24        ISQVNSLLNDYNYNRQSHITR--------------------------------------- 695
Pb-Cdc24        ------------------------------------------------------------
Cc-Cdc24        SNDGPTIAQWRNNVAGPRA----------------------------------------- 731
Lb-Cdc24        STDGLTMAQWRNGLPQPPSNQPSPAGALRPITPRMNSNVSANSYTSDASFGNSSVPLPLK 793
Pp-Cdc24        HT---SLYPGRPSISRSAMS----------------YNVETDSASS-------------S 729
Yl-Cdc24        ------------------------------------------------------------
Pg-Cdc24        SGPSGHPLHARHNEPQLLPSNSTGVPMFRSRSASSPNIHQSPDFSQQWNSGLLNPVPSVP 803
Ca-Cdc24        ------------------------------------------------------------
Sc-Cdc24        ------------------------------------------------------------

Mg-Cdc24        QGASQQSRSRSYSTPDIQGQGPPGMRRTPGGSQSNVPAVPGIPAHLHAAHERH------- 913
Fg-Cdc24        AALAAQQRNRSYSTPDING---PGMPRTRQPSHGNIPAVPGIPQHLHPGHN--------- 781
An-Cdc24        SSNSASQRMRSASSPDIHHHNPESRRYMGVHTMQTVDNVPVPPIPAHMANMK-------- 754
Sp-Cdc24        ------------------------------------------------------------
Pb-Cdc24        ------------------------------------------------------------
Cc-Cdc24        ------------------------------------------------------------
Lb-Cdc24        SSSRPVLRSQASSNRLKNGYDASYNSGIPTPAVNSSDYRTVRAPTPTGGLTAP------- 846
Pp-Cdc24        TTTRSALRSQFSSTRLKGAYDQAESR------PNASSRQSAPAAP--------------- 768
Yl-Cdc24        ------------------------------------------------------------
Pg-Cdc24        SLPSGSHSSSSTSRPALPLHNQVQIGGHIKQRSSDSSAAALTDRSSTESQRLSGGVGGGN 863
Ca-Cdc24        ------------------------------------------------------------
Sc-Cdc24        ------------------------------------------------------------

Mg-Cdc24        ---------DSNIPRSNTGSPALSLRNDLPMRSNTQSPGVQRDRLMQQGGGYSGGTLAQF 964
Fg-Cdc24        ----------PNLSRDQTGSP----RNDQPTRAQTNSPGAQRERMHKPTG-SVGGSMNHF 826
An-Cdc24        ----------APVNRSQNNSP---TNQSLPIRTNTS-------HAFHEPQYSDGRAAAPL 794
Sp-Cdc24        ----------------------------------------VYSGTDDGSSVSIFEDTSSS 715
Pb-Cdc24        ------------------------------------------------------------
Cc-Cdc24        ------------------------------------------------------------
Lb-Cdc24        ---------LMNRSRSASQPTAYVPKTAVPPPMPSYNS--HWSTHDRSNHLN-NNKRGSG 894
Pp-Cdc24        -----------SRSRSASQPTAYIPPTVPPPLPTSIPSSVQWSTRAQSTVGNGHNKRGSG 817
Yl-Cdc24        ------------------------------------------------------------
Pg-Cdc24        GVGGRSNLPLSNPMAQTAHGGPPAGGGGLNPRYSSGQAGLTGHSAASSLITSRSSGSLVV 923
Ca-Cdc24        ------------------------------------------------------------
Sc-Cdc24        ------------------------------------------------------------

Mg-Cdc24        PMQPVYPRGGTPNPPGSGHTPAPLNLSADMGRTVSPPLGTGTPQPPNTAGLMSPDQGGGI 1024
Fg-Cdc24        PAQPVHPRSITP---GGGNQILRVDAAAANSRTVSPALGTATMPPP-SANPLSPEIP--- 879
An-Cdc24        SDQPTSPLSHEP-----------------------------EEEP--------------F 811
Sp-Cdc24        TKQKIFDQPTTNDCDVMRPRQYSYSAGMKSDGSLLPSTKHTSLSSS-----STSTSLSVR 770
Pb-Cdc24        ------------------------------GSSYFPPSLLSPVTLT-----GSQD----- 610
Cc-Cdc24        ------------------------------------------------------------
Lb-Cdc24        SSQSTGDSSEYSPNSS-SPVTPFGSSESSLGGV-AIRNSRTQPYES-----APALNGH-- 945
Pp-Cdc24        SSESTGESSDYSPHSSGSPITPFGSSDTSLAGMSASRHSRSQQHVD-----QVVTSAANE 872
Yl-Cdc24        ------------------------------------------------------------
```

```
Pg-Cdc24        VQGGSEGVGGSRYGAADQLVGGSHPSKPLGGGGGSSSTSSTMKSSLTATHNPNNNNNNNN 983
Ca-Cdc24        ------------------------------------------------------------
Sc-Cdc24        ------------------------------------------------------------
```

```
                  PB1 domain
Mg-Cdc24        MPTQLKVKVNYDT-------SNYMTLVAAYNITYRSLVDRIDAKLARFTNSSISRG-NLR 1076
Fg-Cdc24        LPTQLKVRVNCES-------GNYVTLVVAFNITYQSLVDRIDAKLARFTTSSISKS-MLK 931
An-Cdc24        MPTQLKAKVNFD--------ENYVTLVISSNIGFRTLTDRVDAKLARFTNRSIGSK-TVR 862
Sp-Cdc24        NTTNVKIRLRLH--------EVSLVLVVAHDITFDELLAKVEHKIKLCGILKQAVPFRVR 822
Pb-Cdc24        ---TLKVKLTYN--------EGVYCIVTPHQIKFSDLMERVEKKIKIVANLKPNA--VLR 657
Cc-Cdc24        ------------------------------------------------------------
Lb-Cdc24        --HNVKVKVHFH--------EDIFVIQVSRATEYEDLVEKVGRKIRLCGP-RRDDG-PLR 993
Pp-Cdc24        LSRPVKVKVHFH--------EDIFVIQVPRSTEFDQLVEKVGKKIRLCGPNRRDDG-PLR 923
Yl-Cdc24        ---NIKVRVQYF--------QDVFTLIVPAKVTYDQLIERVERKVRLCGKVVPAP---LR 733
Pg-Cdc24        GPTSIRFKLRAG--------EDTYVIVTLSTITYHELIEKIYKKLRNCGVYKDSSA-RLK 1034
Ca-Cdc24        --FDVAIKLLYKST------ELSEPLIVNAQIEYNDLLQKIISQIITSNLVADDVN-ISR 797
Sc-Cdc24        --SSILFRISYNNNSNNTSSSEIFTLLVEKVWNFDDLIMAINSKISNTHNNNISP--ITK 815
```

```
Mg-Cdc24        LRYRDEESDFVSIVCDEDVQMAFTDGRNGDRDMYN-GGVGEVELFCVGIGGE 1127
Fg-Cdc24        LRYRDEDGDYVAIEGDDDIQIAFMEWREGVRNMYS-GGVGEIELFCVGDTA- 981
An-Cdc24        LRYQDEDGDFVTIDSDEAVQLAFVEWKEQHREELARGQVGEIQLFCQPIEN- 913
Sp-Cdc24        LKYVDEDGDFITITSDEDVLMAFETCTFELMDPVHNKGMDTVSLHVVVYF-- 872
Pb-Cdc24        LKYRDEDGDHITISSDDDVQMAFE-----------SRGQNTVNLFVTL---- 694
Cc-Cdc24        ----------------------------------------------------
Lb-Cdc24        VKYKDEDGDMVSLGSTEDVQMAFE-------------QGGQVTLFVT----- 1027
Pp-Cdc24        VKYKDEDGDLVSLGSTEDVQMAFES----------FRPGNLVTLYVQ----- 960
Yl-Cdc24        IKYQDEDGDMVTIHSDEDIAMALEQ-----------KNLEQLNIWVA----- 769
Pg-Cdc24        IRYEDEEGDLILICNDEDVSMAIEWMR--------SVGISHLLLFVD----- 1073
Ca-Cdc24        LRYKDDEGDFVNLNSDDDWGLVLDMLTSEDFYQTSSNEKRSVTVWVS----- 844
Sc-Cdc24        IKYQDEDGDFVVLGSDEDWNVAKEMLAE--------NNEKFLNIRLY----- 854
```

Alignment of fungal CBSn-PB1 proteins

```
Mg-CBSn-PB1        ------------------------------------------------------------
Fg-CBSn-PB1        -------MSGHSHNTHRSTPNKGRGAVPFANSPSGGGGGGSSNIPRPVLEP--------- 44
An-CBSn-PB1        -------MS--STPTFRGTTSHR--TVGRGRLPDFEGGSSASHIPRPRPESSSTITSHNP 49
Yl-CBSn-PB1        --------------------------MSRNTPSRFSNYSGTSGTQSP------------- 21
Ca-CBSn-PB1        ------------------------------------------------------------
Sp-CBSn-PB1        --------------------------MTVGTLSVVSSTASDT------------------ 16
Cc-CBSn-PB1        ------------------------------------------------------------
Lb-CBSn-PB1        ------------------------------------------------------------
Pp-CBSn-PB1        ------------------------------------------------------------
Pg-CBSn-PB1        MSRIPLPIQVNSTTPTQHQHQHQHQQHSPNSLMTSSQRSANRSGLPRPNSIYSDDPALTS 60
Pb-CBSn-PB1A       ----------------------------------------------------MLPKPSRS 8
Pb-CBSn-PB1B       ------------------------------------------------------------
Bd-CBSn-PB1        ------------------------------------------------------------
```

```
Mg-CBSn-PB1        -----------MSAS----RQKQTKRDEAIRRKMENDLAKKKH---------VTGRARHS 36
Fg-CBSn-PB1        -TPPAETG-SSFSAS----RQKQSKRDEAIRKKLENDLSKKKH---------LTSRARHS 89
An-CBSn-PB1        HTPSSDIGSSTMSAASSRQRQNQSKRDEAIRRKLEADLNKKRS---------NPARANRT 100
Yl-CBSn-PB1        --------IDSLTES----RKRQSKKDEAIRRKLETDLSKRSH---------VRPKP--T 58
Ca-CBSn-PB1        ------MAPPTSLDS----RQRQSKRDDAIRKRIENDLRKKKA---------STSLSTRR 41
Sp-CBSn-PB1        --------ASHVSDT----RKRQYQRDEALRKKIISELGKKSGNF-------ESPVRKIR 57
Cc-CBSn-PB1        ------MSTVSGYSMHSETRKKTNRRDEAIRKKIESELARKR-TISTHQPQPPSRSRRGK 53
Lb-CBSn-PB1        ------MSSLS---FQSDPRKKQSKRDEAIRKKIESELSRKR-TISTTQGQRSKRG-VGK 49
Pp-CBSn-PB1        ------MSTVS--ASVAETRKKQTKRDEAIRKKIEGELSRKR-TISTTQHTSSRRG--HK 49
Pg-CBSn-PB1        STSPSSHRNTSFQSSLSETRQRQGRKDEAIRKKIENELQRKRPGQKRTNNQPAGSSGGPR 120
Pb-CBSn-PB1A        RLPAIATARAETPDVANNGRQRQTKKDEAIRKKLEQELSKKR---------TGTTRVR-Q 58
```

```
Pb-CBSn-PB1B         ----------------------------ALRKKLENELSKKR---------GGSRSLRKH 23
Bd-CBSn-PB1          -----------------------------MKRNSASSASGQK---------------HDT 16


Mg-CBSn-PB1          RKAPPGTVLALKPSQALQIKPGTTVAEAAQLMAAKREDCVLVTDDDDRIAGIFTAKDLAF 96
Fg-CBSn-PB1          RKAPPGTVLALKPSQALQIKPNTTVSEAAQLMAAKREDCVLVTDDDDRIAGIFTAKDLAF 149
An-CBSn-PB1          RKAPPGTVLALKPSSALQIKPSTTIAEAAQLMAAKREDCVLVTDDDDRIAGIFTAKDLAF 160
Yl-CBSn-PB1          KKIVSGTVMALKPSPAVTIKPKTSVSEAAQLMSAKRENCVLVIEDDN-ISGIFTAKDLAF 117
Ca-CBSn-PB1          SRGAPGTVLWLKPGEPIICKPTATVYEVAQLMTARRENCVLVVNEIGELLGIFTAKDVAF 101
Sp-CBSn-PB1          RNGEPGTVDSAALDPALTVHMQSLVTETAQLMAAKRQNCVLVVDDDEQLAGIVTATDIAT 117
Cc-CBSn-PB1          NAPTKGTVAALKPSPALTVPENITVAEASQLCAAKRTDCVLVVDEEEGLSGIFTAKDLAY 113
Lb-CBSn-PB1          ATPAKGTVAALKPSPALTVPENITVAEASQLCAAKRTDCVLVVDDEEGLSGIFTAKDLAY 109
Pp-CBSn-PB1          PNVQKGTVAALKPSPALTVPENITVSEASQLCAAKRTDCVLVVDDDEGLSGIFTAKDLAY 109
Pg-CBSn-PB1          PKRQAGTVSALRPLPALTVPDNITVADASQLCAAKRTDCVLVVDEDEHLCGIFTAKDLAF 180
Pb-CBSn-PB1A         TRKIAGTVSALRPAQALTVKENMLVIEASQLMAAKRSDCVLVVDEDDHLSGIFTAKDLAY 118
Pb-CBSn-PB1B         AQRVAGTVSALRPAQALTVKENMLVMEAAQLLAAKRCDCVLVVDEDDHLSGIFTAKDIAY 83
Bd-CBSn-PB1          RRTRVGTVSSLRPAPAIIIIETARIIQAAQLMAAKRTDAVLVVGEDGALAGILTDKDIAY 76
                          ***                     **  * *   ***        ** *  * *


Mg-CBSn-PB1          RVVGAGLKATNVTIAEIMTKNPLCARTDTSATDALDLMVRKGFRHLPVMDE--------- 147
Fg-CBSn-PB1          RVVGAGAKASAVTIAEIMTKNPLCARTDTSATDALDLMVRKGFRHLPVMDE--------- 200
An-CBSn-PB1          RVVGAGLKARDITVSEIMTKNPLCARTDTSATDALDLMVRKGFRHLPVMDE--------- 211
Yl-CBSn-PB1          KVVGSGLDASVTTVDQIMTRNPLYATTNTSATEGLNLMVNKGFRHLPVMDE--------- 168
Ca-CBSn-PB1          RIVGSGLNATQVTIDTIMTKNPICANAADPAGDALNLMVERGFRHLPVLDE--------- 152
Sp-CBSn-PB1          RCVGAGLNARQTLIADIMSTSPLCITSDTRFDDALLLMIEHKFRHLPVVSDGGPDGSAGD 177
Cc-CBSn-PB1          RVTAEGLDPHSTPVAQIMTRNPMVTRDTTSATEALQLMVSRHFRHLPVCNEDG------- 166
Lb-CBSn-PB1          RVTAEGLDPHTTPVSQIMTRNPMVTRDSTSATEALQLMVSRHFRHLPVCNEDG------- 162
Pp-CBSn-PB1          RVTAEGLDPHVTQVSTIMTRNPMVTRDTTSATEALQLMVQRHFRHLPVCNEEG------- 162
Pg-CBSn-PB1          RVIGDGMDPRTTPVSAIMTRNPMVTRDTTSATEALTTMVTRGFRHLPVCNDEG------- 233
Pb-CBSn-PB1A         RVVAESLDARHTPVSQIMTPGPMCVTADTSATDALNLMVTRGFRHLPVCNEEG------- 171
Pb-CBSn-PB1B         RVVAEELDASHTTVATIMTRGPMCVTSDTSATDALNLMVTRGFRHLPVCNEEG------- 136
Bd-CBSn-PB1          RVVAEGLDIRTTPVSSVMTRDPIAVYDKGSRNEALNIMVSRRFRHLPVISETGGGNDDDD 136
                                      *   *            *  *    ******

Mg-CBSn-PB1          --------NQDISGILDITKCFYDAMEKLERAYSSSRKLYDALEGVQSELGA-------- 191
Fg-CBSn-PB1          --------NQDISGVLDITKCFYDAMEKLERAYSSSRKLYDALEGVQSELGS-------- 244
An-CBSn-PB1          --------NQDISGVLDITKCFYDAMEKLERAYSSSRKLYDALEGVQSELGS-------- 255
Yl-CBSn-PB1          --------NNEVSGILDITKCYHEAMEKLERAYQSSRKLYDALEGVQTELGS-------- 212
Ca-CBSn-PB1          --------KSQIVGVLDITKSYAQQMEKLERMHSSSKKLHEALDSVHNEIGVG------- 197
Sp-CBSn-PB1          --------EGDVIGIINMRACLREPLNRIARQQEAAQKLVEALEGAQEEIENKSVSGNTN 229
Cc-CBSn-PB1          ----------NVVGLLDITKVFHEALGKVERSSAASEQLFNAMAGVQSELGG-------- 208
Lb-CBSn-PB1          ----------NVVGLLDITKVFHEALGKVERSSAASEQLFSAMAGVQSELGGA------- 205
Pp-CBSn-PB1          ----------NVVGLLDITKVFHEALDKVERSSSASEKLYSALAGVQSELGTG------- 205
Pg-CBSn-PB1          ----------DVIGLLDITKVFHESLEKLERAYGSSQKLYNAIEGVQSEFGSGG------ 277
Pb-CBSn-PB1A         ----------DIFGLLDITKCLYEALDKMERAFGSSRKLYDALEGVEREWSGS------- 214
Pb-CBSn-PB1B         ----------DIFGLLDITKCLYEALDKMERAYGSSRKLYDALEGVEREWSSS------- 179
Bd-CBSn-PB1          NEFDEAAGGTSVVGLLDITKCVFERIDDLERKVNEDLNIISAMEALERRGTVA------- 189
                                  *                *          *

Mg-CBSn-PB1          ------SQPQQIIQYVEALRTKMSGPTLESVLNG--------MPPTTVGVKTSVKEAAQM 237
Fg-CBSn-PB1          ------TQPQQIIQYVEALRSKMSGPTLETVLNG--------VPPTTVSVRTSVREAAQL 290
An-CBSn-PB1          ------SQPQQIIQYVEALRSKMSGPTLESVLDG--------MPPTTVSVRTTVKEAAAL 301
Yl-CBSn-PB1          ------SQPAQIINYVEALKQRMEGPDLESVLDG--------TPPTYVDVRTTVFEAASL 258
Ca-CBSn-PB1          ------EQPHHVFQYFETLKNKMNGPTLEDALDAN-------TVPTYVNVKASVHEATML 244
Sp-CBSn-PB1          SSSVSGNHAAEFLEYVESLKKKASGLEIMSLIDSS-------EEPFLVGTRTTVAEATES 282
Cc-CBSn-PB1          -VGSNPQAA-AMLAWAEKLREKTALPDLTTVMDSR-------THPATVGPKTTVRDVAKL 259
Lb-CBSn-PB1          -VGSNPQAA-AMLAWAEKLREKTALPDLTTVMDSR-------TQPATVGPKTTVREVAKL 256
Pp-CBSn-PB1          -LGANPQAA-AMLSYVESLREKTALPDLTSVMDSR-------TQPATVGPKTTVKEVAKL 256
Pg-CBSn-PB1          -RGTTPGAVNPLMAYVEALRNKMSFPDLGSILDAR-------TSAATVGVKTSVKEAAVL 329
Pb-CBSn-PB1A         --------PVQLAQYMETLRDKMSCPDLNTVLDG--------SLPAQVSVKVNVRDVARL 258
Pb-CBSn-PB1B         --------PIQLVQYMEALRDRMSCPDLHSVLDG--------SPPPEVDVKVNVREIAKM 223
Bd-CBSn-PB1          ------------AEHVGVVRQHHGCPDVGFVLTQTIGGQLEHGTVPEVSIKSSVRDAARI 237
                                                                   *     *
```

```
Mg-CBSn-PB1        MKENHTTAVLVQDQ------------GQITGIFTSKDVVLRVIAPGLDPGNCSVVRVMTP 285
Fg-CBSn-PB1        MKENRTTAVLVQDQ------------GAITGIFTSKDVVLRVIAPGLDPANCSVVRVMTP 338
An-CBSn-PB1        MKEHHTTALLVQDQ------------GSITGIFTSKDIVLRVIAPGLDPATCSVVRVMTP 349
Yl-CBSn-PB1        MKQNHTTAVLVTDH------------DQVKGIFTSKDVVLRVIAAGLDPKNCSVIRVMTP 306
Ca-CBSn-PB1        MKENRTTAVLVKDTN-----------EQVAGIFTSKDVVLRVIAAGLDPKKCSVVRVMTP 293
Sp-CBSn-PB1        MARSGVSAVLVMDN------------GAVSGVFTAHDVVLRVLAAGLDPYRSSVIRVMTP 330
Cc-CBSn-PB1        MKERRTTAVCVMEPPGPGTP-----HPRIAGIFTSKDVVLRVIAAGLDAGRCSVVRVMTP 314
Lb-CBSn-PB1        MKERRTTAVCVMETAGPASPGLPA-GSRIAGIFTSKDVVLRVIAAGLDAGRCSVVRVMTP 315
Pp-CBSn-PB1        MKERRTTAVCVMEAPTHAPHAVAAEPPKIAGIFTSKDIVLRVIAAGLDATRCSVVRVMTP 316
Pg-CBSn-PB1        MREHHTTAVCVMESDG----------RRIAGIFTSKDIVLRVIAAGLDARTCSVVRVMTP 379
Pb-CBSn-PB1A       MKEYHTTAVLVSDREG------------LAGIFTTKDVVLRVIAAGLNPENCSVVRVMTP 306
Pb-CBSn-PB1B       MRELHTTAVLVTNREG------------LAGIFTTKDIVLRVVAAGLHPEKCSVVRVMTP 271
Bd-CBSn-PB1        MKAYHTTAVLVIGNSND--------DEQIGGIFTTKDIVLRVIAASLDPMTTSVVRVMTP 289
                   *      *  *                   * **  * **** *  *     ** *****

Mg-CBSn-PB1        HPDFAPMDMSIQAALRKMHDGHYLNLPVMNDSGEIVGMVDVLKLTYATLEQIN------- 338
Fg-CBSn-PB1        HPDFAPMDMTLQAALRKMHDGHYLNLPVMNDGGEIVGMVDVLKLTYATLDQIN------- 391
An-CBSn-PB1        HPDFAPSDMSIQAALRKMHDGHYLNLPVMNEGGEIVGMVDVLKLTYATLEQIN------- 402
Yl-CBSn-PB1        HPDVAPQNMSIQVALRTMHEGRYLNLPVMGPNAELVGVVDVLKLTYATLEQIN------- 359
Ca-CBSn-PB1        QPDVAPIGLPVQDALRKMFDGHYLNLPVVANEGDIIGVVEVLKLTHITLNQIK------- 346
Sp-CBSn-PB1        HPDCALASLRVSTALERMIEGKFSNLPVVDESDAIIGMLSLFHLATAIEQTPEEEEEVFD 390
Cc-CBSn-PB1        HPDTAPPTMTVHDALKKMHNGHYLNLPVVEEDGRLVAIVDVLKLTYATLEQMN------- 367
Lb-CBSn-PB1        HPDTAPPTMSVHDALKKMHNGHYLNLPVIEEDGRLVAIVDVLKLTYATLEQMN------- 368
Pp-CBSn-PB1        HPDTAPPDMTCHDALKKMHIGHYLNLPVVEADGRLVAIVDVLKLTYATLEQMN------- 369
Pg-CBSn-PB1        HPDTALPSLSIQEALRKMHDGHYLNLPVVDEAGQLQGCVDVLKLTYATLEQVN------- 432
Pb-CBSn-PB1A       HPDTASPHMSIIDALRQMHDGHYLNLPVLE-DGRVVGIVDVLKLTYATLEQIN------- 358
Pb-CBSn-PB1B       NPDTASPQTSIMDALRKMNDGHYLNLPVMD-GHIIVGMVDVLKLTYATLEQMN------- 323
Bd-CBSn-PB1        HPDYVLASTSILDALKKLNTGHYLHLPVVD-GGVPIGLVDVMTLTISMLTYLM------- 341
                    **          **     *    ***               *

Mg-CBSn-PB1        ----------------------------------------------TMSSGD-------- 344
Fg-CBSn-PB1        ----------------------------------------------AMSNNN-------- 397
An-CBSn-PB1        ----------------------------------------------SMSTQD-------- 408
Yl-CBSn-PB1        ----------------------------------------------TMSTGD-------- 365
Ca-CBSn-PB1        ----------------------------------------------QLETSETSNGSTAV 360
Sp-CBSn-PB1        QAENDAGIEPSNGFEDQQQQLLGNSNEVVENYDVNPPLPLNPLPSNTQQSESTYEYSARQ 450
Cc-CBSn-PB1        ----------------------------------------------AMSG-E-----AAA 375
Lb-CBSn-PB1        ----------------------------------------------AMAGGD-----TTS 377
Pp-CBSn-PB1        ----------------------------------------------SMTTTE-----NAG 378
Pg-CBSn-PB1        ----------------------------------------------SISS-------EVT 439
Pb-CBSn-PB1A       ----------------------------------------------SIQG---------- 362
Pb-CBSn-PB1B       ----------------------------------------------SIQG---------- 327
Bd-CBSn-PB1        ----------------------------------------------TKDLGT-----QEG 350

Mg-CBSn-PB1        ---NEGPAWNKFWLSLDNE------TESMVSGDGSHHHS-----HSHIHHRS--VMSPDL 388g
Fg-CBSn-PB1        ---DEGPAWNKFWLSLDAE------TESMMSGEGSQAQ------HTNLGSR---LTSPDM 439
An-CBSn-PB1        ---DEGPAWNKFWLSMDHE------SDSMVSGSQSHQPH-----RSIVNPE-----SPKA 449
Yl-CBSn-PB1        ---SEGPAWNKFWMSLDDG------SE-AGSETNSHNRI-----MSPVSVS-----SPEV 405
Ca-CBSn-PB1        DSTNEGPAWNKFWTSLDNT------DGDTESAHSDSLME-----TSRGSAT-----APDI 404
Sp-CBSn-PB1        LPKPPVQAWQNENLSSNNK------PQEYVGVENDYNFS-----NNPPTAMSEQSFHPSV 499
Cc-CBSn-PB1        SDNEGGPMWGRFFDSLGQD-----DTESVFSGSHATG------SVLPGS----MHLAQSP 420
Lb-CBSn-PB1        TEAEGGPMWGRFFDSIGHE-----DTESLFSGSHVTR------DHRSFSSINDLHLQQSP 426
Pp-CBSn-PB1        ENEEGGPMWGRFFDSLGGD-----DTESALSGSHFGT------DVRSRHLS--RSMSHTV 425
Pg-CBSn-PB1        NDDSGGPVWNRFFASFGQAGSVDEDNTSIVSGSQLAHSETGPHTPRHKTGHDGQHPGLET 499
Pb-CBSn-PB1A       -ADGEGPMWSRFWDSFGAT--EHAENESQLSDPTSHH------HHLPASQHPMEAISPEP 413
Pb-CBSn-PB1B       -NDGEGPMWSRFWDSFGST--DHTETESQLSDPMLSH------HPSTNMHPAMHAISPEP 378
Bd-CBSn-PB1        SISEDGPLWNKFWNSTFAGSTIETESDRLSQTSDSRP------SGSIHYQPSSSSQHATE 404
                           *     *
```

PB1 domain

```
Mg-CBSn-PB1        TRERL--------------DSVAPGDSASHAGVESPGHSIVQHSPELPV-SEIPFPFKFK 433
Fg-CBSn-PB1        VRDRLN-------------DTVAPGDSASHVGMESPPRSILPDVLEHQLPEELPFPFKFK 486
An-CBSn-PB1        SFDAR--------------DSVLPNESASHHGGD--------EHSEFHHGELSPFPFKFK 487
Yl-CBSn-PB1        SHSELAQFGV---------GEVGPSDSISTDGYDRP------GSNAAGMIATMPFQFKFK 450
Ca-CBSn-PB1        TPSEFHSFN----------VDIKPSDSVSHVNTSPLKAS-SFKMTSSMTVDEIPFIFKFK 453
Sp-CBSn-PB1        SQKPMDTPENGSNSFA-ASPYLQPYNSASQLAPSYVGSLPQYHGNPSFVEQALQDLVQPT 558
Cc-CBSn-PB1        HS-EVHPNDSASVVDD-DNGSMLS-YSRKKGVSVP---LGSAP----VQPDDGTYVFKFR 470
Lb-CBSn-PB1        HS-EVHPNDSASVIDD-DPVSVLDGYPRHKGLNIP---SGAAP----IPVDDGTYVFKFR 477
Pp-CBSn-PB1        HSPEVHPNDSASMVDEQEEHSVLEGYSSKQGEPLP---SIVGPPVPLPAVDDGTYVFKFR 482
Pg-CBSn-PB1        PGSELAPNDSASVVQE-DYESALAGRSTLQSVTSSQPFTGMVP----LDYDDGTYLFKFI 554
Pb-CBSn-PB1A       SISFSQLHGFPEISPN-ESASMVAHNEDNRSANSS---HNTRPTKTSGNGGDGSFAFKFT 469
Pb-CBSn-PB1B       SLSYTHLQGYAEILPN-ESASMVATNDDALSTISS---RNIR---THGRD-DGTFSFKFT 430
Bd-CBSn-PB1        MSNRQSLQLQRTLSPHPDEYSSMLSRSQIQNDDPN----MFTFKLKDMTRTGNGKVFRFS 460

Mg-CBSn-PB1        AP--SGRVHRLQVIASHGVAAFITNVTSKLGGE----IEAVGGAPEVTEDGQVRGGFALS 487
Fg-CBSn-PB1        AP--SGRVHRMKIVATDGIEAFVEAIASKLGAE----ADNIGGVPDVEDGKIVGSGFALS 540
An-CBSn-PB1        AP--SGRVHRVNILPAAGIAELVAQVTAKLGPE----VEAVGGAASCADGVLSNTGYALS 541
Yl-CBSn-PB1        SP--SGRNHRLTVVPNAGVTALRTSISDKLS---------VG------DVELVTQNYAIS 493
Ca-CBSn-PB1        SPGIEGRVHRITLKASDGIIKLRELMNEKLHDK----DFAFLNVPKPDGNSSTQETYAIS 509
Sp-CBSn-PB1        DS--ASQIFPLNPQSPSQFTIKYRSIAGRVHRLRLDGINSVSDLRTAVEEREKEQLVTLT 616
Cc-CBSn-PB1        TP--SGRTHRFQSRHDD-IQLLREIVAGKLAID---PFFTEFTG-NPGDEVPDPNDFHLS 523
Lb-CBSn-PB1        TP--SGRTHRFQARHDD-VEHLREIVSGKLATD---PFFTEFQPKNEDSPKPDPIDFHLS 531
Pp-CBSn-PB1        TP--SGRTHRFQARHDN-VENLRDIVAGKLATD---PFFTNYKTADPSEPPPDPTDFLLS 536
Pg-CBSn-PB1        AP--SGSAHRFQARYDS-YDFVREIVVGKIESD---PFIADHPS-NATTIKPDAQDFQLA 607
Pb-CBSn-PB1A       TK--GGKTHR-IASTPK-HSQLLELVRQKVLSE---HVPS----AAEG------EWLSIS 512
Pb-CBSn-PB1B       SL--SGRTHRFVAPLDS-YSQLFDSVLQKVATE---HLTLTGKRAGEGAADPSAEWLSIS 484
Bd-CBSn-PB1        SR--FNSLSEVYAQVCL-KTGAKSLIFEAAATS---GTTHSMRSEHLDLETKDGEIVRIC 514

Mg-CBSn-PB1        YLDNEGDSVSITADQDLLEAILLARQSGRDKVDLFVHDPEKPPVAVAAPAAVPPAPSEPV 547
Fg-CBSn-PB1        FLDDEGDSISITADHDLLEAVILARQAHHDKVDLFVHDPEKPPVSAADPRHP-------- 592
An-CBSn-PB1        YVDNEGDTVSITTDQDLVDAVYIARHARRDKVDLFVHDPAQPPVIPAP------------ 589
Yl-CBSn-PB1        YIDDEGDIVAITSDQDLLDAVSITRRAGLEKADLFIHHPDNE------------------ 535
Ca-CBSn-PB1        YVDDEGDVVSITSDSDLAECIRINLNLQNEKADLYLHNPHEP------------------ 551
Sp-CBSn-PB1        YIDDEGDVVELVSDSDLREAILLARRRGLPRLEVRGVAAFTNHLESS------------- 663
Cc-CBSn-PB1        YTDDDGDSVLMTTDSDVADAVTIARNHNQQRVVLFIQGGKGWAEAG-------------D 570
Lb-CBSn-PB1        YTDADGDTVLITSDDDITDAVKIARTAGTDRVVLFVQGGKGWAEAGA------------D 579
Pp-CBSn-PB1        YTDADGDIVLITSDTDVSDAVKIARAAGADRVVLYIQGGKGWADVMA------------E 584
Pg-CBSn-PB1        YLDDENDLVLITSDRDVTDAVNQAKKQRKDRVVLHLRGGKSWGDSELS-----------K 656
Pb-CBSn-PB1A       YLDDEDDEVLITSDADVQDAVRLARKLGQDRVKLFAHDSTSVTAHTA------------- 559
Pb-CBSn-PB1B       YMDDENDEVLMSSDADVQDAVMLARKVGQSRVKLFIHD--AMIESIA------------- 529
Bd-CBSn-PB1        YMDDENDVVHLESDKDVEEAVLMSRRLGLTRLMIYLGEPLLQNAVTDS-----------Q 563
                     *   *      * *

Mg-CBSn-PB1        SVPIPTPPASSVARERRRRKS-PASDESDEEEESEDDGSSTLRRPRRHQRA-AAAQQEPL 605
Fg-CBSn-PB1        ----ATPSVSTGAGLRERRKWWPEEEEEEDDDDSEDEHPARRRKSRAAHTH-AHAHEEQI 647
An-CBSn-PB1        ----VEPAPVKPVEVKT-----PASDDQLSEE-------SPIPKPRATQAYPAHPPEEQL 633
Yl-CBSn-PB1        ----VKPVVT-----------------------------QKLEDSVEIPVKAAAAPAPEI 562
Ca-CBSn-PB1        -----APIEN-------------------------------IKSIHKSAKNRHGNADNGL 575
Sp-CBSn-PB1        ----HPPISTVDSSIGS---------------------ASVVEKGVANSIVDIHQPTAKA 698
Cc-CBSn-PB1        KSEAKAQEVS--AAALKEVKEVEKAEEEAEDTPLVQTPPKATTPPT----RGVVHNPQEE 624
Lb-CBSn-PB1        KSEAKAAAVT--AAAQKEVKEVEKAEEIIVVTPPPVEAPVAQTPPVQ-THRPRAPPPAEE 636
Pp-CBSn-PB1        KSAAQAAEVA--AAAQAETKEVEKAESVLP-------EPVSNPPPP-----PVHVVEDET 630
Pg-CBSn-PB1        KKAAEVYSTR--LKAVTEAEEIENEELEERHSQAGSRDRLSTTRSSKKNAHNQTPVDPTL 714
Pb-CBSn-PB1A       EPPAPVISLS--PPSDVSSKTMRQEDDDTSSMTSRSTRKRSSKRKSSRRNESEEPEEYES 617
Pb-CBSn-PB1B       AEEAELIDNN--DDG--------EEDTAEEEETEEEYIRKSSKKRSHRRHHYDD------ 573
Bd-CBSn-PB1        YSSGTATPNVGHHGQVVRYEDSHQSVVSEQSLVEGGNSRINPVVVYNPINGGQGRDPPAS 623

Mg-CBSn-PB1        IAGVPNELLLPGAIVTLA-VVIVGVFTISRISSNNR- 640
Fg-CBSn-PB1        IAGVPNELILPGAIVTLA-VAIVGVFTIARLTSR--- 680
An-CBSn-PB1        IAGVPNDLLLPGAIVTLA-AVIAGVFILSRATSR--- 666
Yl-CBSn-PB1        IPGLSNDLLLPGAVAVVA-AACAVAFSLGRAR----- 593
```

```
Ca-CBSn-PB1          IFGIPNEILIPSALAVLG-ASIIVGFTLSRK------ 605
Sp-CBSn-PB1          DKGNSKKPIYIGIVSSSI-VILAVSMWYLRRKR---- 730
Cc-CBSn-PB1          VFGIPKDLLLPASIGALA-VVIIDGEWVKPLDR---- 656
Lb-CBSn-PB1          VFGIPKDLLLPASIGALA-VVIIGIFTISRLSSPSHY 672
Pp-CBSn-PB1          VMGIPKDLLLPASIGALA-AVILGVFTISRLSR---- 662
Pg-CBSn-PB1          VAGVPKEFILPAAIGFLG-VVILGVFLASKPGH---- 746
Pb-CBSn-PB1A         NTGFPQELILPASIAFLG-VVILGVFAFSRVSPKHR- 652
Pb-CBSn-PB1B         --GFPRDLLLPAAIVFLG-VVVVGVFAISRISPRHR- 606
Bd-CBSn-PB1          IVDYLKDAPLPVNIAISAGIIVVASYLIMKLQR---- 656
```
