# Supplementary material for: Molecular evolution of Phox-related regulatory subunits for NADPH oxidase enzymes
Source: BMC Evol Biol. 2007 Sep 27;7:178. doi: 10.1186/1471-2148-7-178 (PMC2121648; doi:10.1186/1471-2148-7-178)
Supplement: Additional file 15 — Amino acid sequences of PB1 domain-containing proteins of D. discoideum and alignment of PB1 domains. Amino acid sequences of PB1 domain-containing proteins of the slime mold amoeba D. discoideum (Dd-PB1-A to -E) and an alignment of the PB1 domians are provided. [file 1471-2148-7-178-S15.pdf]

Additional File 15

Amino acid sequences of PB1 domain-containing proteins of *Dictyostelium discoideum*. Sequences are obtained from the indicated servers: GenBank™ (http://www.ncbi.nlm.nih.gov/)

>Dd-PB1-A: GenBank No. XP_639165
MSWLNNPSQNFVDPFIRIKCILGDDIRIIKFNSNISYGGLMNQLEQDFQCPISIHQYEDYEGDKVTV
KSKDDIMEALTMYFELKALNPTKIISTKFFLKQLPPQSQPLSSSLSPTQSLILNNNNNNSNNNLMN
MNMNMNLNMNSNNSSGSSSSNNIVIPQQQGNRKGSNAPPLRTPPPSPPSSSPKNSPPFLGNNSNTS
INILDFYSLNGSNNNSGGTGSNNNGIGSSSREITPPLRTPPGSPRFALNNSNSNSNSSVNNIPTNNNN
NNHHHIIHQLNLNSPSPPPPLRTPPPSPPSSSPPSSSPQSSFIFEQTDFHFEELSKNNSPKNSNFLNTID
QPHQIYQPHQPHYQQQQQQQQMYNFNQPLSPSPPLQSQQKQQQQQQQQQQQQQQPQQQQQQQ
QQQQQQQQQQQQQFNNSFENGGIKLPRPLSPTFLNINNNIINSNLNNNNNNNNNNNNNNNNN
NNNNNNIIQHTDFPSLIINEHEELISNHNIKWQKGQILGRGGYGSVYLGLNKDTGELFAVKQLEIV
DINSDPKLKNMILSFSKEIEVMRSLRHDNIVRYLGTSLDQSFLSVFLEYIPGGSISSLLGKFGAFSEN
VIKVYTKQILQGLSFLHANSIIHRDIKGANILIDTKGIVKLSDFGCSKSFSGIVSQFKSMQGTPYWM
APEVIKQTGHGRSSDIWSLGCVIVEMATAQPPWSNITELAAVMYHIASSNSIPNIPSHMSQEAFDF
LNLCFKRDPKERPDANQLLKHPFIMNLDDNIQLPTISPTTTLSTNTTNTTATTTTTNNATNSNINQ
QQQQQQQPPTRTQRVSISAGSSNNKRYTPPISTSTSSSSSSILNNFSINIILPINLIILIFREIKPNFVN
TLSRVCKHWKQIIDDDELWNKYCSDRLINKSKFEESITWKSNYIKIYKQQKVWFHNKLNHSTLK
GHDKGVFCVKLIDDQGMVLSGGEDKKLKVWDISGNHHHNHHSGIVGSISKKSGLNIINNNNSNN
SNSSSNSSSSNSRYLFSLKGHSGCIKSVDYQRQSGSDVSRVFTASADFTCKIFSLKTKKTLFTYTN
HQEAVTCINYLGDVENKCITSSLDKTIQLWDAETGSCLSTLRGHTGGIYCVKTDQVATHGNGYN
HLVVSASVDKTSNVWDTRSSSKVRSFTQHTEDVLCCYVFDQKVVTGSCDGTIKLWDIGTGKTIS
TFIPSETRQKNYVWTVQFDQSKIISSGKTGIIRIWDIYNERDSRSIGGHHETIFSLQFNNQKLITGSL
DKLVKIWSID
>Dd-PB1-B: GenBank No. XP_646538
MVNLILKIQHNDDTRRVSMERDPTFLELRKMTVTFFKINSFLIKYFDEDKDLITITSDNDLKEAFSI
ATTSPRTVRLFVSKTEEESSSETINNNNTTPSINNYQNPLSNSVNNNNNNNNNNNDNNTMNLKPLI
DSILANPNIAQLASASAVSCLTPKVHTSVYGIPTTGTDQTQIENLLSNLGSQNWINEIVQNSLSNIF
KPNVNNNNQNQNQSTTTNNNNTTTTTTTSTTKNEEKQKTEKNENMVEHVGITCDGCDSKVFGN
RYKCTVCHDYDLCSECESRGDQVHPTSHPLLKIAQPTPISCSWQHSNAGRSGIPHGFGGGRCTRK
VYAARYLADISIKDGSVIPKGSSFTKTWRLRNDGKTSWPENTTLSFLSGDRFQYQTDIFVPVCQP
GQDIDISVDLVAPTKTGRYTGYWRLSTPEGFGFGQSIWVDIYVIADEDDNKKQQPIIQEEKQQEEE
QKDVVQRLPDSDSEDELLKQEQEKLEEEEEEEEEEKDEPKIEEEADSFCFLPFTPSPFSPFSIQNNV
PASVQVPSEISNSFNIPPVAEQQQQEKINNNTSNNSNNYQLPPLPVVEQEQIIENVQPQVEELPKLE
ELSVNGEQEDIRRKCIGTLVSMGFSNTPNIIEIIKRYNFNINEIIDHLLSNQEQ
>Dd-PB1-C: GenBank No. XP_644298
MNILIKSELENDKRRFRLKECSFSCLCYTLASIYSFYNDMIYSIFYLDNENEWITLASTDDLKESYS
LCPSLIRIKIIVLDLTINNNNNNNNNNNNNNNSCKLLLNNHSSNSNNIYNHSNSSNSNNYSTNNNNNN
NNNNNNNNNNNNNNNNNNNTTNNNNYHNNINNNSNSNNKIIDIASQLLSNDSIRSQMNNLLSGTL
ASFLPEILEKTIPHTIPILVATISSMKAEAEQNNYDEPTFYNNKDKHYKSTDESQHINIKSAGNNIPT
SKSSVELSVNKKSVASNNNNNNNNVNNSSNTSNNNIIINNNNVSKPQPIQNVMSQSSPNLTFTTNN
NNTTCSNINSSTGSMEPMEINPTTQPYSIISSTPASTSLPPLSATTSFLPTSSTTSNNNAGTSNGFTSIT
NSNNNNNNSNNNNNNNNNNSSILSQSVPSVNLINKKPSSPSPILSLFSKSSITPIQTSSISLPSPGISPFS
NPLDGSSLINPETTPSQSSSSFFRPVSPTSISSLENNNTNSNTTVNNNNNNNNDTSPLKRKLSRFSFFS

SILGNKNNNTSTNTTKMDSPEISSTISDISTVDCSTTSSSTNQPNTSVIKLDIEQEKDDQEKQVDDL
QFGLIIEPMPLNVMEDLNNTITNDDNNNNNNNNNNNNNNNNNNINDDDVGDSLVNDINNQNDEK
NKNDIGSGSIVIVEDCISTQNNNDNNNNNNNKNNIISSNSLTVDENDDSGRSSASMDYNETSLNN
NANTTKTTTNHLIINNGLELLSCDDDDEDDEITKEIESVFLKQQQQKLQQQQQQQQQQQQQQQ
QQQQQQQQQQQQQQQQQQQQQQQQQQQQQ
>Dd-PB1-D: GenBank No. XP_638776
MVDQPPKFWSFLGCHGNNSANNILAPPNPTKENNNSSNGSINGYGGNTNNLDSTTLNVTLGSEM
KSITVPKSSTYKDMISTIKDKFGVNSKSTLCIKCENKDGEMFSLASDCHVKKAYNQQPENQPKEL
RLVVKEIPQKKCDMFHNSILSFFSNCSSSNSNTIAQNYTSTQPPPASQSSLQHLATQPVPDYLKPSN
NNKSDFTPPTSSSPSYLTSNNNINNNNNNNNNNNNNNNNNNNNNNNNNNNNNNNNNNNNNNN
LNSNIGTSTNNTNNINLRFSLFQMSNSSTNNISNIKNMGINQNINNSLNRGFFAKNNQTSLCMGSH
NSNLNNNNNNNNNNNNNNNNNNNNNNNNNNNNNNNNNNNNNNNNNNNNNKISTSPKQSNILPP
INTKSNNNNGINNNLSPSQQQHQSQQKLNTTLQHVTSNPQLNMSTSSFMTSASMNSNIGSDTETM
KRNESFDFNMVMEDDSSEPQSQSYQQNQSIINNGTCALLNQNLNDEVEFGDEQQQQEKQKIINN
GKPPTSTPMCREVGSFIQDTTKLFYKLRQTSDPKEMSAIKELICEKIQDSLTTMPWLLGFFPQLKQ
FANQSCPVFNVPPNTPPPSPLMTSPLLEMYQNPTSTFKPSSSVSSSLSSSTSSTTTTTKTSSTVNQAS
VYQVSPNVNINSIQSQLQLQNKIEVDMISKAYQESKKNQKIINEQQQQQQQRPIDFSSQQSSANT
TTDEDSSCSNSNNNNNNNNSKHFLKIAGPTIHKGTTCAFCKFSPIIGNIYECNSCSYAFCELCKTDSA
LQCPSNDPTHKVQMSSLSKQCKKLLKSKASQTGSSSVSASPNRGCPYKRKHPKPTFGVKFLNDIT
LFFGSEVHPNECIVKTWRLLNTGPTLKDCLLVRVCGNTRLSKVPAILIPIVSSGEEFSLSVPIQIPLIP
NKCEEYLVGEYWRICTSDGVYFGDQLWISLVVKNREMISLSDQFNKLSSQCNSTSQQNQQQQQ
QQQQQQQQQQQQQQQQQQQQQQQQQQQQQQQQQQKTTTTTSTSCTCRNNNLISCNCQVNKN
STNFNSCLN
>Dd-PB1-E: GenBank No. XP_635325
MGITYKSNFEGDVRRFSSDHPLTYTRLQDKLVNLYNLYEISFGITYLDDGDNITIADAKDLEEAH
NLLGNEILRLTITRKLNENKNINNSSSSSNNNSNNSNNNNNNIDSLFVQLLSNQFVQQMIPQVMNSL
ATNPALMTSIIQTAGSALRNNGVNNNNNNNNSNNNTNNNNNNNNNNVNSGDNNNNNINCNIKRNS
PHLLNASLGNNFHTHSTDSIVATLKPIIDEENNGNNNNNTTRITLTSSNGNESCNGSFSSSISFEMV
NKESDSDNQYSTSSSTTSSTSATSNSHSLNNRPPSPMENLNKSPSTIVSSSSSSSSSSSSSSTTSSSST
TTTTTTTPLPPPTTTTNNRLSGIPKSIFNILPFTNTTHSSNNNNNNNSNKIEIKPKQDEPSNVNNTLSSS
SKPIATPTTATPTSQQQQQTNSSSPKQDRNMSTSPQQPPSHPHNSPNHLFQHNFSNPYIYQNGNVG
ATQPPPLPQSPFIPQQPVAPLVQQQPLPSAPLSQDQQQQQQQQQQQQQQQQQQQQQQQQQQQ
QQQQQQQQPLPTAPLSQSQEQQQPLSSESIKFIKEALQISESEAIKLFEKYGGDLDKIFSEIKN

Alignment of PB1 domains of Dd-PB1-A to -E

Each PB1 domains were identified by Pfam search program (http://www.sanger.ac.uk/Software/Pfam/search.shtml). Amino acid sequences were trimmed to the length of PB1 domain and were aligned. Dd-p67-like and Fg-Bem1 were used as references. *Red* and *blue letters* indicate the amino acid residues corresponding to K355 and K382 of PB1 domains of human p67*phox* and four acidic amino acid residues (D289, D291, D293 and D302) of OPCA motif of human p40*phox* PB1 domain,

respectively.

```
Dd-PB1C         -MNILIKSELENDKRRFR-LKECSFSCLCYTLASIYSFYNDMIYSIFYLD-NENEWITLAS
Dd-PB1E         -MGITYKSNFEGDVRRFSSDHPLTYTRLQDKLVNLYNLY-EISFGITYLD--DGDNITIAD
Dd-PB1B         MVNLILKIQHNDDTRRVSMERDPTFLELRKMTVTFFKIN---SFLIKYFD-EDKDLITITS
Dd-PB1D         --STTLNVTLGSEMKSITVPKSSTYKDMISTIKDKFGVNSKSTLCIKCEN-KDGEMFSLAS
Dd-PB1A         ---IRIKCILGDDIRIIKFNSNISYGGLMNQLEQDFQCP---ISIHQYED-YEGDKVTVKS
Dd-p67-PB1      --KITLKVFYKDRRLI-QIPVPCNLSTFIQKIELKFEIT---ISDKFSFQ-LDGEENEINS
Fg-Bem1-PB1     --AMKIKMYFNGDLIAIRVPTDISFQALYDKICDRLKIPANEEIQLFYKDEPTGDKPSMIS

Dd-PB1C         TDDLKESYSLCP--------SLIRIKIIVL 80
Dd-PB1E         AKDLEEAHNLLGN-------EILRLTITRK 80
Dd-PB1B         DNDLKEAFSIATT-------SPRTVRLFVS 80
Dd-PB1D         DCHVKKAYNQQPEN------QPKELRLVVK 134
Dd-PB1A         KDDIMEALTMYFELKALNPTKIISTKFFL- 98
Dd-p67-PB1      QVQLDKMICM----------EINEINVKD- 383
Fg-Bem1-PB1     DNDLDFALQR-NE-------KLLLYVEAV- 590
```
