# Supplementary material for: Molecular evolution of Phox-related regulatory subunits for NADPH oxidase enzymes
Source: BMC Evol Biol. 2007 Sep 27;7:178. doi: 10.1186/1471-2148-7-178 (PMC2121648; doi:10.1186/1471-2148-7-178)
Supplement: Additional file 16 — Amino acid sequences of hypothetical p67phox-like (hypo-p67-L) of Arabidopsis thalianaand Oryzae sativa. Sequences of A. thaliana and O. sativa proteins that contain the TPR and PB1 domains (At-hypo-p67-L1 to L4 and Or-hypo-p67-L1) are shown. [file 1471-2148-7-178-S16.doc]

**Additional File 16**

**Amino acid sequences of hypothetical p67*phox*-like (hypo-p67-L) of *Arabidopsis thaliana* and *Oryzae sativa*.** Sequences of *Arabidopsis thaliana* (At) and *Oryzae sativa* (Os) proteins similar to p67*phox* (At-hypo-p67-L1 to L4 and Or-hypo-p67-L1, respectively) are shown. Sequences are available from the GenBank server (http://www.ncbi.nlm.nih.gov/).

>At-hypo-p67-L1 (GenBank No. NP_194935.1)

MGKPTAKKKNPETPKDASGGGGGGGGKSGKTYHRSTSRVFDEDMEIFISRALELKEEGNKLFQKRDHEGAMLSFDKALKLLPKDHIDVAYLRTSMASCYMQMGLGEYPNAISECNLALEASPRYSKALVRRSRCYEALNKLDYAFRDARIVLNMEPGNVSANEIFDRVKKVLVDKGIDVDEMEKDFVDVQPVCAARLKKIVKERLRKSKKKKKSGGKDEELKSPKVVVVDKGDEAEGRNKPKEEKSDKSDIDGKIGGKREEKKTSFKSDKGQKKKSGGNKAGEERKVEDKVVVMDKEVIASEIVDGGGSKKEGATVTRTIKLVHGDDIRWAQLPLDSTVRLVRDVIRDRFPALRGFLIKYRDTEGDLVTITTTDELRLAASTHDKLGSLRLYIAEVNPDQEPTYDGMSNTESTDKVSKRLSSLADNGSVGEYVGSDKASGCFENWIFQFAQLFKNHVGFDSDSYVDLHDLGMKLYTEAMEDAVTGEDAQELFQIAADKFQEMGALALLNWGNVHMSKARKQVCIPEDASREAIIEAVEAAFVWTQNEYNKAAEKYEEAIKVKPDFYEALLALGQEQFEHAKLCWYHALKSKVDLESEASQEVLKLYNKAEDSMERGMQIWEEMEECRLNGISKLDKHKNMLRKLELDELFSEASEEETVEQTANMSSQINLLWGSLLYERSIVEYKLGLPTWDECLEVAVEKFELAGASATDIAVMVKNHCSSESALEGNQFLARIPNSGQVTTQWFSVYNNLRTNAGMGFKIDEIVQAWNEMYDAKRWQMGVPSFRLEPMFRRRAPKLHDILENVFSGPV

> At-hypo-p67-L2 (GenBank No. NP_180101.1)

MGKPTGKKKNNNYTEMPPTESSTTGGGKTGKSFDRSATKSFDDDMTIFINRALELKEEGNKLFQKRDYEGAMFRYDKAVKLLPRDHGDVAYLRTSMASCYMQMGLGEYPNAINECNLALEASPRFSKALLKRARCYEALNKLDFAFRDSRVVLNMEPENVSANEIFERVKKVLVGKGIDVDEMEKNLVNVQPVGAARLRKIVKERLRKKKKKSMTMTNGGNDGERKSVEAVVEDAKVDNGEEVDSGRKGKAIEEKKLEDKVAVMDKEVIASEIKEDATVTRTVKLVHGDDIRWAQLPLDSSVVLVRDVIKDRFPALKGFLIKYRDSEGDLVTITTTDELRLAASTREKLGSFRLYIAEVSPNQEPTYDVIDNDESTDKFAKGSSSVADNGSVGDFVESEKASTSLEHWIFQFAQLFKNHVGFDSDSYLELHNLGMKLYTEAMEDIVTGEDAQELFDIAADKFQEMAALAMFNWGNVHMSKARRQIYFPEDGSRETILEKVEAGFEWAKNEYNKAAEKYEGAVKIKSDFYEALLALGQQQFEQAKLCWYHALSGEVDIESDASQDVLKLYNKAEESMEKGMQIWEEMEERRLNGISNFDKHKELLQKLGLDGIFSEASDEESAEQTANMSSQINLLWGSLLYERSIVEYKLGLPTWDECLEVAVEKFELAGASATDIAVMVKNHCSSDNALEGNYVYL

> At-hypo-p67-L3 (GenBank No. NP_197536.1)

MEKQNEEISTDDAETSQSQLVDDSKVETLDDCVSKVETLDDCVSKVETLDDCVSKAETLADCVSKVETLDDCVSKVKTLDDCVSKVENLDDCVPKVETLDDCVPKVETLDDCVSEVETLDDCVSKAQGLKEEGNKLFQKRDYDGAMFKYGEAIKILPKDHVEVSHVRANVASCYMQLEPGEFAKAIHECDLALSVTPDHNKALLKRARCYEALNKLDLALRDVCMVSKLDPKNPMASEIVEKLKRTLESKGLRINNSVIELPPDYVEPVGASPAALWAKLGKVRVKKTKKSNQVEEKSEGEGEDVEPEKKNNVLAEKGKEKIKMKVKGKQSDKRSDTSKEQEKVIIEEELLVIGVEDVNKDVKFVYSDDIRLAELPINCTLFKLREVVHERFPSLRAVHIKYRDQEGDLVTITTDEELRMSEVSSRSQGTMRFYVVEVSPEQDPFFGRLVEMKKLKITADSFKAKVNGRGGCKVEDWMIEFAHLFKIQARIDSDRCLNLQELGMKLNSEAMEEVVTSDAAQGPFDRAAQQFQEVAARSLLNLGYVHMSGARKRLSLLQGVSGESVSEQVKTAYECAKKEHANAKEKYEEAMKIKPECFEVFLALGLQQFEEARLSWYYVLVSHLDLKTWPYADVVQFYQSAESNIKKSMEVLENLETGKESEPSQAGKTDCLTHEKDLGSSTQNNPAKEAGRLKSWIDILLCAVLYERSIMEYKLDQPFWRESLEAAMEKFELAGTCKDDVVEIISEDYVAGNTLRDIRFHMEEIIQIFDEIYEAKHWTNGIPSDQLEEILKRRAENIFHVPNIAIQRG

>At-hypo-p67-L4 (GenBank No. NP_564794.1)

MGKSGGRKKKSGGSNSNSSQVNSSETSGLSKPSTIVNGGVDFDASIFLKRAHELKEEGNKKFQARDYVGALEQYENGIKLIPKSHPDRAVFHSNRAACLIEMKPIDYESVISECSMALKSQPGFTRALLRRARAFEAVGKFDLAVQDVNVLLGSDPNHKDAGEIFEAVKNCLGPHQDLQSRPFTCSSWCFGCFRRSYCWTWSCLPSRNVHKKGVTSPVGSVSLPNLVMEGLRGRQVVNPVTENGGSVSKGQASRVVLKPVSHSPKGSKVEELGSSSVAVVGKVQEKRIRWRPLKFVYDHDIRLGQMPVNCRFKELREIVSSRFPSSKAVLIKYKDNDGDLVTITSTAELKLAESAADCILTKEPDTDKSDSVGMLRLHVVDVSPEQEPMLVSRKKRRKMEEKPVIEEVISSPTESLSETEINTEKTDKEVEKEKASSSEDPETKELEMDDWLFDFAHLFRTHVGIDPDAHIDLHELGMELCSEALEETVTSEKAQPLFDKASAKFQEVAALAFFNWGNVHMCAARKRIPLDESAGKEVVAAQLQTAYEWVKERYTLAKEKYEQALSIKPDFYEGLLALGQQQFEMAKLHWSYLLAQKIDISGWDPSETLNLFDSAEAKMKDATEMWEKLEEQRMDDLKNPNSNKKEEVSKRRKKQGGDGNEEVSETITAEEAAEQATAMRSQIHLFWGNMLFERSQVECKIGKDGWNKNLDSAVERFKLAGASEADIATVVKNHCSNEAAATEEMRKRYLRLETLYPNKNYDIKSYETKTDSRLMNGCVTGK

>Os-hypo-p67-L1 (GenBank No. BAD31284)

MGKPAAAKKPPASAAVDGDEEVFLELSRELKEEGGRLFNRRDYEGAAFKYDKAVQLLPSGGHADAAAHLRTCVAQCYMRMAPAEHHRAIHECNLALEAAPRYSRALLRRAACFQALDRPDLAWEDVRTVLAWEPANRAAREISDKVRAALEEKGVLVLEKEPVPPPPEHKAVSAKGQGKLKKSHKQCDSAIEGQELIHVEDYEQSEKTELKINGQENGENRAGKEQFDCNVPVKQEIRTDQPEANGVGKHQYHMDDKENKGLDKEGKNGKPGKHSAGKKIRRADAKKQKHSAMEPVHHAEENRHERYTETSVHVKEAMKDLKLVFGEDIRCAQMPANCNLSQLRDIVQNKFPSLKALLIKYKDKEGDLVTITSSDELRWAYSLADLEGPIRLYIVAVDPAQELGVDVVRRRSSFASLEKAYYSMSENGSSRHDDDHNCSIDDWMIQFARLFKNHLGFDSDSYLDLHDLGMRLYYEAMEDTVASEEAQEIFQVAELKFQEMAALALFNWGNVHMASARKRPPLSDDASMECILEQVKVAYEWACAEYAKAGAKYGEAVKTKPDFFEGLIALGQQQFEQAKLCWYYALACKIDMGTEVLGLFNHAEDNMEKGMGMWEGMENTRLRGLSKPSKEKIIFEKMGIDGYMKDMSSDEAFEQASSIRSHVNILWGTILYERSVVEFILGLPSWEESLTVAIEKFKTGGASPADINVMVKNHSANETTQEGLSFKVEEIVQAWNEMYDAKKWRNGVPSFRLEPIFRRRAPKLHHMLEHIHYA
