# Supplementary material for: Molecular evolution of Phox-related regulatory subunits for NADPH oxidase enzymes
Source: BMC Evol Biol. 2007 Sep 27;7:178. doi: 10.1186/1471-2148-7-178 (PMC2121648; doi:10.1186/1471-2148-7-178)
Supplement: Additional file 17 — Alignment of hypothetical p67phox-like (hypo-p67-L) of Arabidopsis thaliana and Oryza sativa. (i) Alignment of four hypo-p67-L1 to -L4 of A. thaliana, (ii) comparison of TPR and AD domain between the A. thaliana and O. sativa proteins (At-hypo-p67-L1-4 and Os-hypo-p67-L1) and human p67phox (1–212 residues) and NOXA1 (1–213 residues) proteins are shown. [file 1471-2148-7-178-S17.pdf]

Additional File 17

Alignment of hypothetical p67*phox*-like (hypo-p67-L) proteins of *Arabidopsis thaliana*. The following names were used to describe species of sequences: At (as *A. thaliana*), Os (*O. sativa*). *Gray* and *yellow boxes* indicate the amino acid sequences of TPR and of PB1 domains, respectively.

```
Athypo-p67-L1        MGKPTAKKKNPE-TPKDASGGGGGGGGKSGKTYHRSTSR--------------------- 38
Athypo-p67-L2        MGKPTGKKKNNNYTEMPPTESSTTGGGKTGKSFDRSATK--------------------- 39
Athypo-p67-L4        MGKSGGRKKKSGGSNSNSSQVNSSETSGLSKP-STIVNG--------------------- 38
Athypo-p67-L3        MEKQNEEISTDDAETSQSQLVDDSKVETLDDCVSKVETLDDCVSKVETLDDCVSKAETLA 60

Athypo-p67-L1        -----------------------------------------------------VFDEDME 45
Athypo-p67-L2        -----------------------------------------------------SFDDDMT 46
Athypo-p67-L4        -----------------------------------------------------GVDFDAS 45
Athypo-p67-L3        DCVSKVETLDDCVSKVKTLDDCVSKVENLDDCVPKVETLDDCVPKVETLDDCVSEVETLD 120

Athypo-p67-L1        IFISRALELKEEGNKLFQKRDHEGAMLSFDKALKLLPKDHIDVAYLRTSMASCYMQMGLG 105
Athypo-p67-L2        IFINRALELKEEGNKLFQKRDYEGAMFRYDKAVKLLPRDHGDVAYLRTSMASCYMQMGLG 106
Athypo-p67-L4        IFLKRAHELKEEGNKKFQARDYVGALEQYENGIKLIPKSHPDRAVFHSNRAACLIEMKPI 105
Athypo-p67-L3        DCVSKAQGLKEEGNKLFQKRDYDGAMFKYGEAIKILPKDHVEVSHVRANVASCYMQLEPG 180

Athypo-p67-L1        EYPNAISECNLALEASPRYSKALVRRSRCYEALNKLDYAFRDARIVLNMEPGNVSANEIF 165
Athypo-p67-L2        EYPNAINECNLALEASPRFSKALLKRARCYEALNKLDFAFRDSRVVLNMEPENVSANEIF 166
Athypo-p67-L4        DYESVISECSMALKSQPGFTRALLRRARAFEAVGKFDLAVQDVNVLLGSDPNHKDAGEIF 165
Athypo-p67-L3        EFAKAIHECDLALSVTPDHNKALLKRARCYEALNKLDLALRDVCMVSKLDPKNPMASEIV 240

Athypo-p67-L1        DRVKKVLVDKGIDVDEMEKDFVDVQPVCAARLKKIVKERLRKSKKKKKSGGKDEELKSPK 225
Athypo-p67-L2        ERVKKVLVGKGIDVDEMEKNLVNVQPVGAARLRKIVKERLR--KKKKKSM---------- 214
Athypo-p67-L4        EAVKNCLG-PHQDLQSRPFTCSSWCFGCFRRSYCWTWSCLPSRNVHKKGV---------- 214
Athypo-p67-L3        EKLKRTLESKGLRINNSVIELPPDYVEPVGASPAALWAKLGKVRVKKTKK---------- 290

Athypo-p67-L1        VVVVDKGDEAEGRNKPKEEK---SDKSDIDGKIGGKREEKKTSFKSDKGQKKKSGGNKAG 282
Athypo-p67-L2        ---TMTNGGNDGERKSVEAV---VEDAKVD-----NGEE------VDSGRK-----GKAI 252
Athypo-p67-L4        ---TSPVGSVSLPNLVMEGL---RGRQVVN-------------PVTENGGSVSKGQASRV 255
Athypo-p67-L3        ----SNQVEEKSEGEGEDVEPEKKNN-----------------VLAEKGKEKIKMKVKGK 329

Athypo-p67-L1        EERKVEDKVVVMDKEVIASEIVDGGGSKKEGATVTRTIKLVHGDDIRWAQLPLDSTVRLV 342
Athypo-p67-L2        EEKKLEDKVAVMDKEVIASEIK-------EDATVTRTVKLVHGDDIRWAQLPLDSSVVLV 305
Athypo-p67-L4        VLKPVSHSPKGSKVEELGSSSVAVVGKVQEKRIRWRPLKFVYDHDIRLGQMPVNCRFKEL 315
Athypo-p67-L3        QSDKRSDTSKEQEKVIIEEELLVIG-----VEDVNKDVKFVYSDDIRLAELPINCTLFKL 384

Athypo-p67-L1        RDVIRDRFPALRGFLIKYRDTEGDLVTITTTDELRLAASTH-------------DKLGSL 389
Athypo-p67-L2        RDVIKDRFPALKGFLIKYRDSEGDLVTITTTDELRLAASTR-------------EKLGSF 352
Athypo-p67-L4        REIVSSRFPSSKAVLIKYKDNDGDLVTITSTAELKLAESAADCILTKEPDTDKSDSVGML 375
Athypo-p67-L3        REVVHERFPSLRAVHIKYRDQEGDLVTITTDEELRMSEVSS-------------RSQGTM 431

Athypo-p67-L1        RLYIAEVNPDQEPTYDGMSNT---ESTDKVSKRLSSLADNGSVGEY--------VGSDKA 438
Athypo-p67-L2        RLYIAEVSPNQEPTYDVIDND---ESTDKFAKGSSSVADNGSVGDF--------VESEKA 401
Athypo-p67-L4        RLHVVDVSPEQEPMLVSRKKRRKMEEKPVIEEVISSPTESLSETEINTEKTDKEVEKEKA 435
Athypo-p67-L3        RFYVVEVSPEQDPFFG----------RLVEMKKLKITADSFKAKVN-----------GRG 470

Athypo-p67-L1        SGC---------FENWIFQFAQLFKNHVGFDSDSYVDLHDLGMKLYTEAMEDAVTGEDAQ 489
Athypo-p67-L2        STS---------LEHWIFQFAQLFKNHVGFDSDSYLELHNLGMKLYTEAMEDIVTGEDAQ 452
Athypo-p67-L4        SSSEDPETKELEMDDWLFDFAHLFRTHVGIDPDAHIDLHELGMELCSEALEETVTSEKAQ 495
Athypo-p67-L3        GCK---------VEDWMIEFAHLFKIQARIDSDRCLNLQELGMKLNSEAMEEVVTSDAAQ 521
```

```
Athypo-p67-L1         ELFQIAADKFQEMGALALLNWGNVHMSKARKQVCIPEDASREAIIEAVEAAFVWTQNEYN 549
Athypo-p67-L2         ELFDIAADKFQEMAALAMFNWGNVHMSKARRQIYFPEDGSRETILEKVEAGFEWAKNEYN 512
Athypo-p67-L4         PLFDKASAKFQEVAALAFFNWGNVHMCAARKRIPLDESAGKEVVAAQLQTAYEWVKERYT 555
Athypo-p67-L3         GPFDRAAQQFQEVAARSLLNLGYVHMSGARKRLSLLQGVSGESVSEQVKTAYECAKKEHA 581

Athypo-p67-L1         KAAEKYEEAIKVKPDFYEALLALGQEQFEHAKLCWYHALKSKVDLESEASQEVLKLYNKA 609
Athypo-p67-L2         KAAEKYEGAVKIKSDFYEALLALGQQQFEQAKLCWYHALSGEVDIESDASQDVLKLYNKA 572
Athypo-p67-L4         LAKEKYEQALSIKPDFYEGLLALGQQQFEMAKLHWSYLLAQKIDISGWDPSETLNLFDSA 615
Athypo-p67-L3         NAKEKYEEAMKIKPECFEVFLALGLQQFEEARLSWYYVLVSHLDLKTWPYADVVQFYQSA 641

Athypo-p67-L1         EDSMERGMQIWEEMEECRLNGISK--------LDKHKNMLRKLELDELFSEASEEETVEQ 661
Athypo-p67-L2         EESMEKGMQIWEEMEERRLNGISN--------FDKHKELLQKLGLDGIFSEASDEESAEQ 624
Athypo-p67-L4         EAKMKDATEMWEKLEEQRMDDLKNPNSNKKEEVSKRRKKQGGDGNEEVSETITAEEAAEQ 675
Athypo-p67-L3         ESNIKKSMEVLENLETGKESEPSQ------------AGKTDCLTHEKDLGSSTQNNPAKE 689

Athypo-p67-L1         TANMSSQINLLWGSLLYERSIVEYKLGLPTWDECLEVAVEKFELAGASATDIAVMVKNHC 721
Athypo-p67-L2         TANMSSQINLLWGSLLYERSIVEYKLGLPTWDECLEVAVEKFELAGASATDIAVMVKNHC 684
Athypo-p67-L4         ATAMRSQIHLFWGNMLFERSQVECKIGKDGWNKNLDSAVERFKLAGASEADIATVVKNHC 735
Athypo-p67-L3         AGRLKSWIDILLCAVLYERSIMEYKLDQPFWRESLEAAMEKFELAGTCKDDVVEIISEDY 749

Athypo-p67-L1         SSESALEGNQFLARIPNSGQVTTQWFSVYNNLRTNAGMGFKIDEIVQAWNEMYDAKRWQM 781
Athypo-p67-L2         SSDNALEGN-------------------YVYL---------------------------- 697
Athypo-p67-L4         SNEAAATEEMR---------------KRYLRLET------------LYPNKNYDIKSYET 768
Athypo-p67-L3         VAGNTLRDIRFH-----------------------------MEEIIQIFDEIYEAKHWTN 780

Athypo-p67-L1         GVPSFRLEPMFRRRAPKLHDILENVFSGPV 811
Athypo-p67-L2         ------------------------------
Athypo-p67-L4         KTDSRLMNGCVTGK---------------- 782
Athypo-p67-L3         GIPSDQLEEILKRRAENIFHVPNIAIQRG- 809
```

Comparison of TPR and AD domain between hypothetical p67*phox*-like protein (hypo-p67-L) of plants and human p67*phox* (1-212 residues) and NOXA1 (1-213 residues) proteins. The following names were used to describe species of sequences: At (as *A. thaliana*), Os (*O. sativa*), and Hs (as *H. sapiens*). *Gray boxes* indicate AD region of human p67*phox* and NOXA1 and predicted AD-like region of At-hypo-p67-L1 and L2. *Red letters* indicate crucial residues in TRP (D67 and R102) and AD regions (V204) of human p67*phox*. *Asterisks* indicated positions of residues that were identified in Figure 5 as conserved residues among all activator proteins (p67phox, NOXA1, and hypo-p67).

```
Hsp67phox             -MSLVEAISLWNEGVLAADKKDWKGALDAFS---------AVQDPHS--RICFNIGCMYTI 49
HsNOXA1               MASLGDLVRAWHLGAQAVDRGDWARALHLFS---------GVPAPPA--RLCFNAGCVHLL 50
Athypo-p67-L1         -IFISRALELKEEGNKLFQKRDHEGAMLSFDKALKLLPKDHIDVAYL--RTSMASCYMQMG 103
Athypo-p67-L2         -IFINRALELKEEGNKLFQKRDYEGAMFRYDKAVKLLPRDHGDVAYL--RTSMASCYMQMG 104
Oshypo-p67-L1         -VFLELSRELKEEGGRLFNRRDYEGAAFKYDKAVQLLPSGGHADAAAHLRTCVAQCYMRMA 80
```

```
Athypo-p67-L3          -DCVSKAQGLKEEGNKLFQKRDYDGAMFKYGEAIKILPKDHVEVSHV--RANVASCYMQLE 103
Athypo-p67-L4          -IFLKRAHELKEEGNKKFQARDYVGALEQYENGIKLIPKSHPDRAVF--HSNRAACLIEMK 178
                                        **                       *

Hsp67phox              LKNMTEAEKAFTRSINRDKHLAVAYFQRGMLYYQTEKYDLAIKDLKEALIQLRGNQLIDYK 110
HsNOXA1                AGDPEAALRAFDQAVTKDTCMAVGFFQRGVANFQLARFQEALSDFWLALEQLRGHAAIDYT 111
Athypo-p67-L1          LGEYPNAISECNLALEASPRYSKALVRRSRCYEALNKLDYAFRDARIVLNMEPGNVSANEI 164
Athypo-p67-L2          LGEYPNAINECNLALEASPRFSKALLKRARCYEALNKLDFAFRDSRVVLNMEPENVSANEI 165
Oshypo-p67-L1          PAEHHRAIHECNLALEAAPRYSRALLRRAACFQALDRPDLAWEDVRTVLAWEPANRAAREI 142
Athypo-p67-L3          PGEFAKAIHECDLALSVTPDHNKALLKRARCYEALNKLDLALRDVCMVSKLDPKNPMASEI 164
Athypo-p67-L4          PIDYESVISECSMALKSQPGFTRALLRRARAFEAVGKFDLAVQDVNVLLGSDPNHKDAGEI 239
                       *          *   *  *                          *    **

Hsp67phox              ILGLQFKLFACEVLYNIAFMYAKKE-EWKKAEEQLALATSMKSEPRHSKIDK-------- 161
HsNOXA1                QLGLRFKLQAWEVLHNVASAQCQLG-LWTEAASSLREAMSKWPEGSLNGLDS-------- 162
Athypo-p67-L1          FDRVKKVLVDKGIDVDEMEKDFVD--VQPVCAARLKKIVKERLRKSKKKKKSGGKDEELK 222
Athypo-p67-L2          FERVKKVLVGKGIDVDEMEKNLVN--VQPVGAARLRKIVKERLRK--KKKKS-------- 213
Oshypo-p67-L1          SDKVRAALEEKGVLVLEKEPVPPPPEHKAVSAKGQGKLKKSHKQCDSAIEGQELIHVEDY 202
Athypo-p67-L3          VEKLKRTLESKGLRINNSVIELPPDYVEPVGASPAALWAKLGKVRVKKTKKS-------- 216
Athypo-p67-L4          FEAVKNCLG-PHQDLQSRPFTCSSWCFGCFRRSYCWTWSCLPSRNVHKKGVTSPVG---- 294
                        *       *

Hsp67phox              -----AMECVWKQKLYEPVVIPVGRLFRPNE----------------------------R 188
HsNOXA1                -----ALDQVQRRGSLPPRQVPRGEVFRPHR----------------------------W 189
Athypo-p67-L1          SPKVVVVDKGDEAEGRNKPKEEKSDKSDIDGKIGGKREEKKTSFKSDKGQKKKSGGNKAG 282
Athypo-p67-L2          ---MTMTNGGNDGE--RKSVEAVVEDAKVD-----NGEE------VDSGRK-----GKAI 252
Oshypo-p67-L1          EQSEKTELKINGQENGENRAGKEQFDCNVPVKQEIRTDQPEANGVGKHQYHMDDKENKGL 262
Athypo-p67-L3          ----NQVEEKSEGE--GEDVEPEKKNNVLAE----KGKEK-----IKMKVK-----GKQS 256
Athypo-p67-L4          --SVSLPNLVMEGLRGRQVVNPVTENGGSVS----KGQAS------------------RV 331
                                     *     * *
                                 AD
Hsp67phox              QVAQLAKKDYLGKATVVASVVDQD---------- 212
HsNOXA1                HLKHLEPVDFLGKAKVVASAIPDD---------- 213
Athypo-p67-L1          EERKVEDKVVVMDKEVIASEIVDGGGSKKEGATV 316
Athypo-p67-L2          EEKKLEDKVAVMDKEVIASEIK-------EDATV 279
Oshypo-p67-L1          DK---EGKNGKPGKHSAGKKIRRADAKKQKHSAM 293
Athypo-p67-L3          DKR--SDTSKEQEKVIIEEELLVIG---VEDV-- 283
Athypo-p67-L4          VLKPVSHSPKGSKVEELGSSSVAVVGKVQEKRIR 364
```
