# Supplementary material for: Molecular evolution of Phox-related regulatory subunits for NADPH oxidase enzymes
Source: BMC Evol Biol. 2007 Sep 27;7:178. doi: 10.1186/1471-2148-7-178 (PMC2121648; doi:10.1186/1471-2148-7-178)
Supplement: Additional file 18 — ID numbers of hypothetical PB1 domain-containing genes of Arabidopsis thaliana,except for hypo-p67-L genes. a total of 27 ID numbers of Arabidopsis thaliana genes are shown. [file 1471-2148-7-178-S18.pdf]

Additional File 18

ID numbers of PB1 domain-containing genes of *Arabidopsis thaliana,* except for hypo-p67-L genes. Genes are identified using NCBI UniGene database ID numbers. The genes are available in NCBI database (http//www.ncbi.nlm.nih.gov/).

1. At.43530 protein kinase family protein
2. At.21300 protein kinase family protein
3. At.48395 protein kinase family protein
4. At.43754 protein kinase family protein
5. At.42436 protein kinase family protein
6. At.29723 CBS domain-containing protein / octicosapeptide/Phox/Bemp1 (PB1) domain-containing protein
7. At.35243 CBS domain-containing protein / octicosapeptide/Phox/Bemp1 (PB1) domain-containing protein
8. At.37545 CBS domain-containing protein / octicosapeptide/Phox/Bemp1 (PB1) domain-containing protein
9. At.71804 CBS domain-containing protein / octicosapeptide/Phox/Bemp1 (PB1) domain-containing protein
10. At.49212 CBS domain-containing protein / octicosapeptide/Phox/Bemp1 (PB1) domain-containing protein
11. At.24692 Ubiquitin-associated (UBA)/TS-N domain-containing protein / octicosapeptide/Phox/Bemp1 (PB1) domain-containing protein
12. At.43367 Octicosapeptide/Phox/Bem1p (PB1) domain-containing protein

13. At.19855 Octicosapeptide/Phox/Bem1p (PB1) domain-containing protein

14. At.27308 Octicosapeptide/Phox/Bem1p (PB1) domain-containing protein

15. At.50254 Octicosapeptide/Phox/Bem1p (PB1) domain-containing protein

16. At.61015 Octicosapeptide/Phox/Bem1p (PB1) domain-containing protein

17. At.27450 Octicosapeptide/Phox/Bem1p (PB1) domain-containing protein

18. At.70293 Octicosapeptide/Phox/Bem1p (PB1) domain-containing protein

19. At.4069 Octicosapeptide/Phox/Bem1p (PB1) domain-containing protein

20. At.35281 Octicosapeptide/Phox/Bem1p (PB1) domain-containing protein

21. At.27500 Octicosapeptide/Phox/Bem1p (PB1) domain-containing protein

22. At.19833 Octicosapeptide/Phox/Bem1p (PB1) domain-containing protein

23. At.53480 Octicosapeptide/Phox/Bem1p (PB1) domain-containing protein

24. At.51764 Octicosapeptide/Phox/Bem1p (PB1) domain-containing protein

25. At.72006 Octicosapeptide/Phox/Bem1p (PB1) domain-containing protein

26. At.71567 Octicosapeptide/Phox/Bem1p (PB1) domain-containing protein

27. At.67350 Octicosapeptide/Phox/Bem1p (PB1) domain-containing protein
